# Streamlined synthetic assembly of α-chiral CAAC ligands and catalytic performance of their copper and ruthenium complexes†

**DOI:** 10.1039/d4sc04278f

**Published:** 2024-07-24

**Authors:** Adrien Madron du Vigné, Nicolai Cramer

**Affiliations:** a Laboratory of Asymmetric Catalysis and Synthesis, Institute of Chemical Sciences and Engineering, École Polytechnique Fédérale de Lausanne (EPFL) 1015 Lausanne Switzerland nicolai.cramer@epfl.ch

## Abstract

The unique electronic and steric parameters of chiral cyclic alkyl amino carbene (CAAC) ligands render them appealing steering ligands for enantioselective transition-metal catalyzed transformations. Due to the lack of efficient synthetic strategies to access particularly attractive α-chiral CAACs assessment and exploitation of their full synthetic potential remain difficult. Herein, we report a streamlined strategy to assemble a library of diastereo- and enantiomerically pure CAAC ligands featuring the notoriously difficult to access α-quaternary stereogenic centers. A tailored Julia–Kocienski olefination reagent allows the Claisen-rearrangement to be leveraged as an expedient route to form the synthetically pivotal racemic α-chiral methallyl aldehydes. Subsequent condensation with chiral amines and further cyclization provided a library of diastereomeric mixtures of the targeted ligand precursors. The CAAC salts as well as their corresponding metal complexes are conveniently separable by standard silica gel flash chromatography closing a long-standing accessibility gap in chiral CAAC ligands with proximal α-chirality. The rapid availability of both diastereomers enables testing of the relevance and synergistic effects of two chiral centers on the ligand in catalytic applications. A broad range of metal complexes with copper, gold, rhodium and ruthenium were obtained and structurally analyzed. The catalytic performances of the corresponding chiral CAAC copper and ruthenium complexes were assessed in enantioselective conjugate borylations and asymmetric ring closing metathesis, displaying selectivities of up 95 : 5 er.

## Abstract 2

A streamlined strategy to diastereo- and enantiomerically pure α,β-chiral CAAC salts and their corresponding transition metal complexes is reported, displaying high enantioselectivities in benchmark asymmetric transformations.

## Graphical abstract

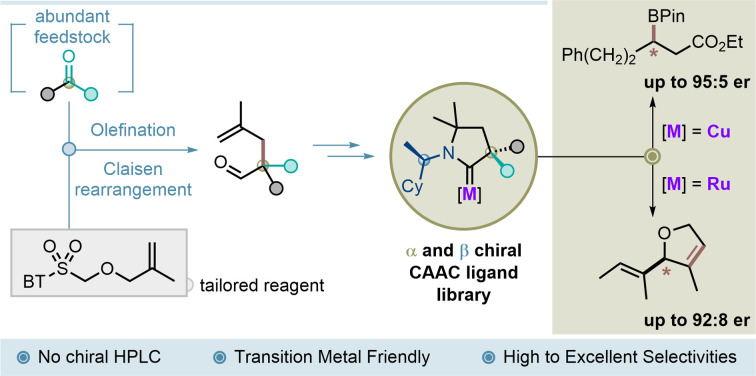

## Introduction

**Scheme 1:**
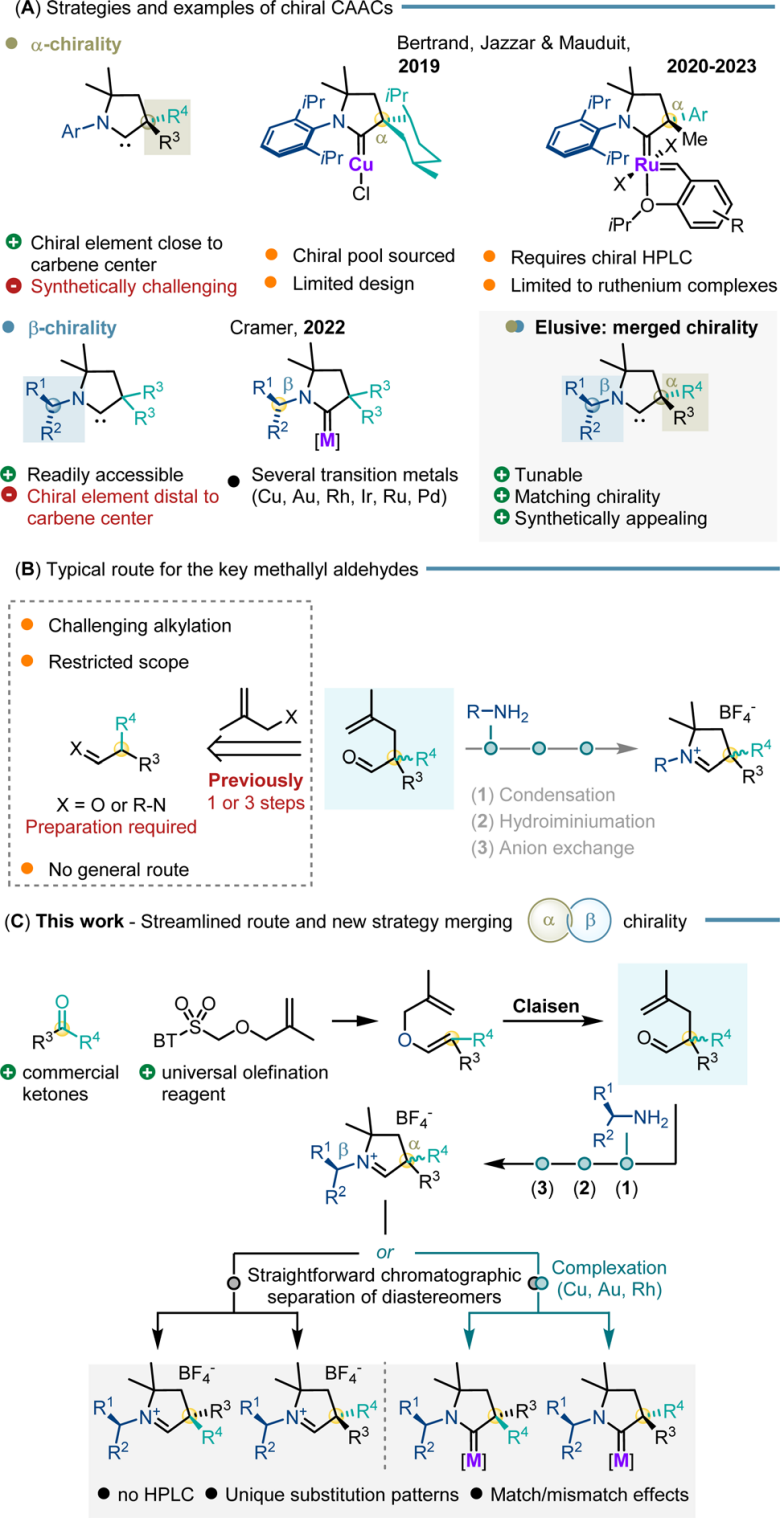
(A) Reported strategies and examples for chiral CAACs; (B) limitations of the current route for methallyl aldehyde key precursors in the CAAC synthesis; (C) streamlined strategy for rapid access to α,β-chiral CAACs.

N*-*Heterocyclic carbenes (NHCs) are an important ligand class for transition metals enabling a broad variety of asymmetric transformations.^1^ The chiral elements of NHCs have been extensively investigated and modulated providing excellent levels of enantioselectivity with a myriad of transition metal complexes.^2^ Introduced by Bertrand in 2005, cyclic alkyl amino carbenes (CAACs) are an intriguing class of NHC ligands.^3^ Compared to the classical NHC framework, one of the heteroatoms adjacent to the carbene carbon atom is switched to an sp^3^-hybridized quaternary carbon atom in CAACs. This change induces profound steric and electronic changes compared to typical NHCs.^4,5^ Notably, they exhibit enhanced sigma-donor and pi-acceptor properties.^6^ As a result, stronger bonds are formed with various main group elements^7^ and transition metals.^8,9^ In this respect, CAACs garnered substantial attention and underwent rapid evolution in the field.^10^ However, despite their great application potential, the development of chiral CAACs for asymmetric catalysis remains severely underdeveloped (Scheme 1A). Bertrand *et al.* reported the two first chiral CAAC complexes for asymmetric catalysis in 2019.^11^ The underlying ligand is based on the chiral-pool approach having one stereogenic element adjacent to the carbene carbon atom. Their selectivity and efficiency were tested in the asymmetric conjugate borylation of unsaturated esters with enantioselectivities of up to 77.5 : 22.5 er. A library of α-chiral CAAC bearing ruthenium complexes displayed selectivities of up to 94 : 6 er in asymmetric ring opening cross metathesis (AROCM). However, the enantiopure complexes had to be resolved by chiral preparative HPLC from the prepared racemate.^12,13^ Independently, we and Bertrand *et al.* reported in 2022 a streamlined chiral CAAC synthesis combining the benefits of functionalized methallyl aldehydes with various primary amines. The chiral primary amines used in our study resulted in CAACs having their stereogenic element at the beta position of the carbene carbon atom. The related copper CAAC catalysts provided improved selectivities of up to 89 : 11 er in asymmetric conjugate borylation.^14^ However, the development of chiral CAACs remains substantially limited and largely restricted to these examples. This restriction primarily stems from the inconvenience and costs of resolution by chiral preparative HPLC as well as the incompatibility of many CAAC metal complexes with chiral chromatographic separation techniques. A general and synthetically attractive method to build the quaternary α-stereogenic center of CAACs suitable for a broad spectrum of transition metals is lacking. This limitation significantly impedes the development of CAACs for catalytic enantioselective transformations and represents a central challenge that needs to be addressed. In this context, methallyl aldehydes emerged as key building blocks shortening the synthesis of CAAC ligands as well as broadening the scope.^14,15^ However, the two main routes to access such aldehydes face significant limitations in general applicability (*i.e.* substrate dependent capricious phase-transfer aldehyde alkylation^15,16^ and transient imine alkylation^17^). Most importantly, both synthetic routes are based on unsymmetrically substituted tertiary aldehydes as key starting materials. Very few aldehydes are commercial and a more desirable substitution pattern requires additional and sometimes tedious preparatory steps (Scheme 1B). Recognizing this limitation, we reasoned that a Claisen rearrangement could allow straightforward access to the pivotal carbene precursors from allyl vinyl ethers.^18^ In turn, synthesis of a single tailored Julia–Kocienski olefination reagent would allow unsymmetrical ketones to be simply used as starting materials. This choice leverages the commercial availability of unsymmetrical ketones that span immense structural diversity (Scheme 1C).

Pairing them with a selection of chiral amines directly leads to a library of carbene precursors with α- and β-stereogenic centers. The formed diastereomers can be separated by simple flash chromatography as their tetrafluoroborate salt carbene precursors or as their CAAC metal complexes. Our approach offers a substantial advantage by providing direct access to CAACs with challenging chirality of the quaternary α-carbon stereogenic centers as well as exploiting unprecedented matched/mismatched effects of multiple stereogenic centers on the CAAC ligands. The catalytic efficiency and inducible enantioselectivity of the corresponding copper and ruthenium complexes were showcased with asymmetric conjugate borylations and ARCMs as selected benchmark transformations.

## Results and discussion

**Scheme 2:**
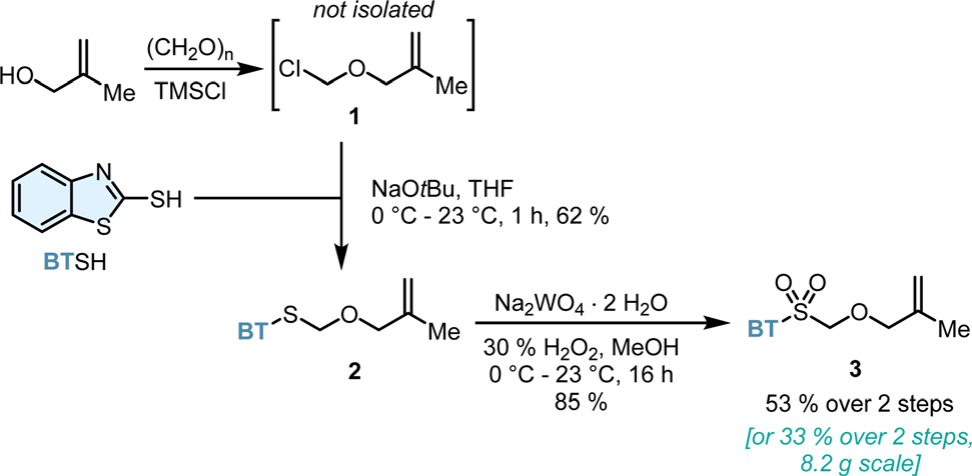
Preparation of Julia–Kocienski olefination reagent 3.

Considering the structural and synthetic challenges in accessing α-chiral CAACs, we began developing a bespoke and universal olefination reagent in order to prepare the pivotal methallyl aldehyde structural unit more efficiently (Scheme 2). In this respect, we envisioned Julia–Kocienski reagent 3 as the platform to perform the desired ketone olefination. Two single reports focused on the preparation of simple α-benzyloxy^19^ or α-naphthylmethyloxy^20^ heteroaryl sulfones and their use for vinyl ether synthesis. To the best of our knowledge, the preparation and olefination use of α-methallyloxy heteroaryl sulfones are surprisingly not yet documented. Reagent 3 was accessed conveniently from widely available and cheap starting materials. Chloromethyl methallyl ether 1 was prepared *in situ* from methallyl alcohol, paraformaldehyde and TMSCl and directly used. Subsequently, thioether 2 was formed from mercaptobenzothiazole and freshly prepared 1. Oxidation of sulfide 2 with aqueous hydrogen peroxide catalyzed by sodium tungstate provided the target sulfone 3 in 53% yield over two steps. The key Julia–Kocienski reagent 3 can be conveniently prepared on a multigram scale and is a stable and free-flowing white solid.

**Scheme 3:**
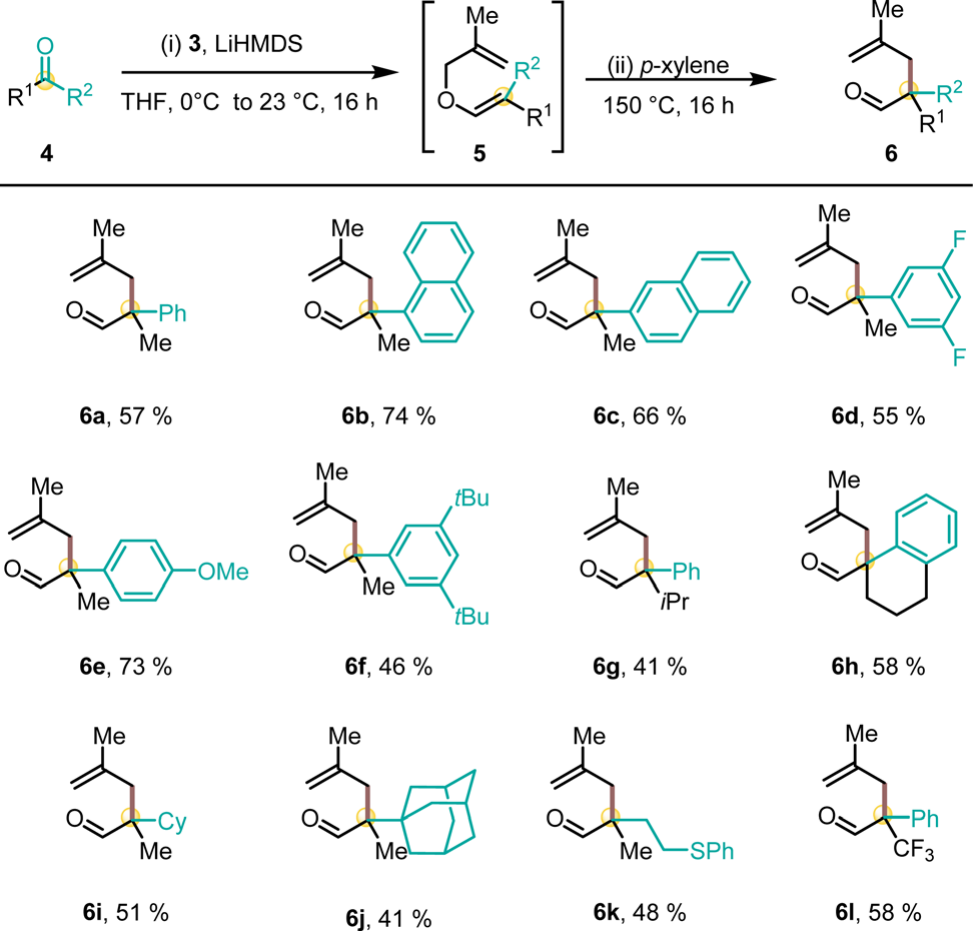
Preparation of methallyl aldehydes 6. Isolated yields over 2 steps.

To leverage the utility of 3 for the synthesis of CAAC ligands, a variety of racemic quaternary methallyl aldehydes were synthesized (Scheme 3). The olefination of a non-symmetrical ketone proceeded smoothly with deprotonated sulfone 3. The resulting crude methallyl vinyl ether 5 underwent Claisen rearrangement yielding aldehyde 6 upon refluxing in *p*-xylene. This approach proved to be synthetically versatile with respect to the substitution pattern of ketone 4. All methallyl vinyl ethers 5 reliably underwent [3,3]-sigmatropic rearrangement. A variety of sterically and electronically diverse aryl-substituents (phenyl (6a), 1-naphthyl (6b), 2-naphthyl (6c), 3,5-di-fluorophenyl (6d) 4-methoxyphenyl (6e) and 3,5-di-*tert*-butylphenyl substituted ketone (6f)) were well tolerated in the olefination–rearrangement sequence. A switch of the methyl substituent at the quaternary carbon atom by an isopropyl group or the generation of a spirocyclic tetrahydronaphthalene (THN) core provided aldehydes (6g) and (6h). Methyl ketones yielded cyclohexyl derived (6i), demanding adamantyl substituted (6j) and sulfide-functionalized aldehyde 6k. Moreover, trifluoro acetophenone was used to access aldehyde 6l in 58% yield.

**Scheme 4:**
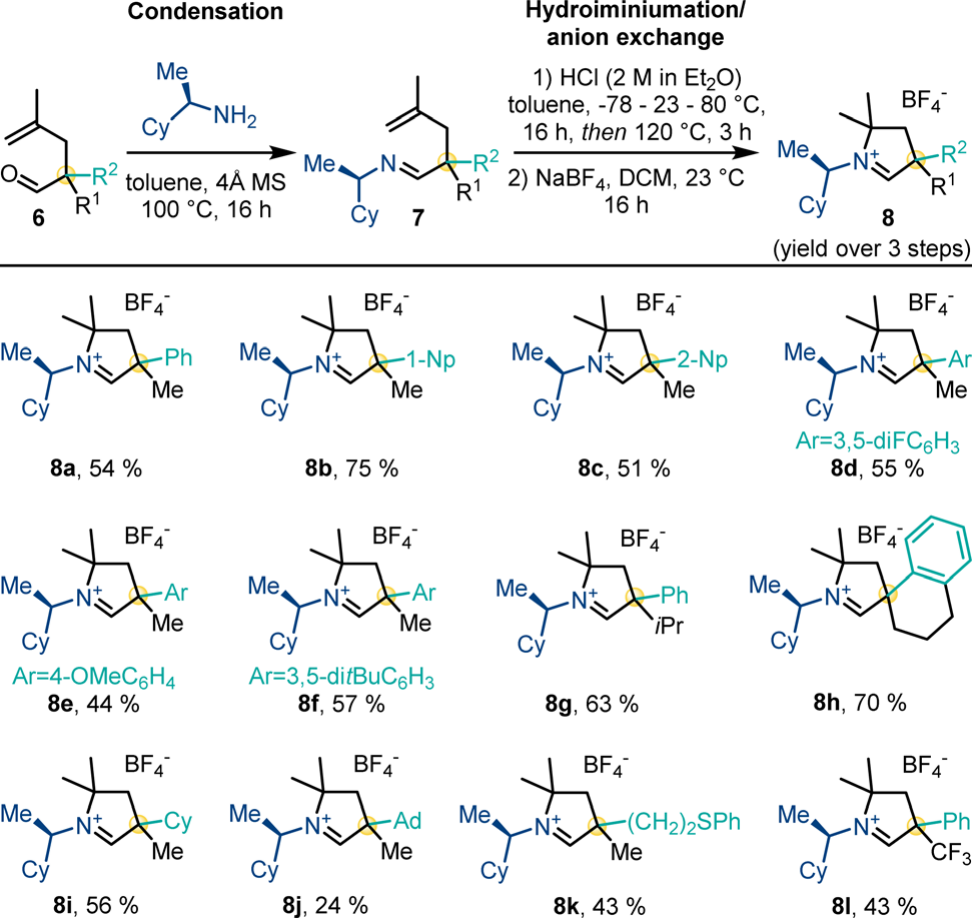
Preparation of the carbene precursor library.

Next, aldehydes 6 were converted to their corresponding carbene precursors 8 by our previously reported procedure (Scheme 4).^14^ The different cyclic iminium tetrafluoroborate salts 8 were generally obtained in good to high overall yields (43–75%) as a 1 : 1 mixture of diastereomers. The process allowed for unprecedented substitution patterns by introducing a sterically demanding adamantyl (8j) as well as an electron-withdrawing trifluoromethyl group (8l) in close proximity to the future carbene center.

**Scheme 5:**
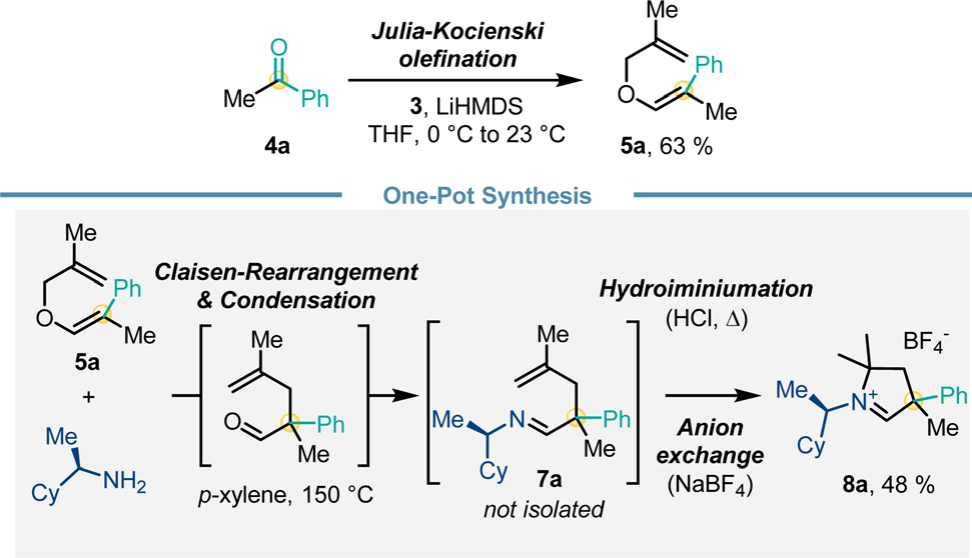
Streamlined one-pot synthesis of carbene precursor 8a.

We further aimed to further enhance convenience and efficiency of the route for the carbene precursor synthesis (Scheme 5). While a full one-pot procedure from 4 to 8 proved to be challenging, we established a proof-of-concept for a one-pot protocol from methallyl vinyl ether 5a. Heating 5a and (*R*)-cyclohexylethylamine triggered Claisen rearrangement and subsequently imine formation giving 7a. Subsequent addition of HCl and heating initiated the hydroiminiumation reaction. Anion exchange with NaBF_4_ provided carbene precursor 8a in 48% yield over 4 steps in a one-pot fashion.

**Scheme 6:**
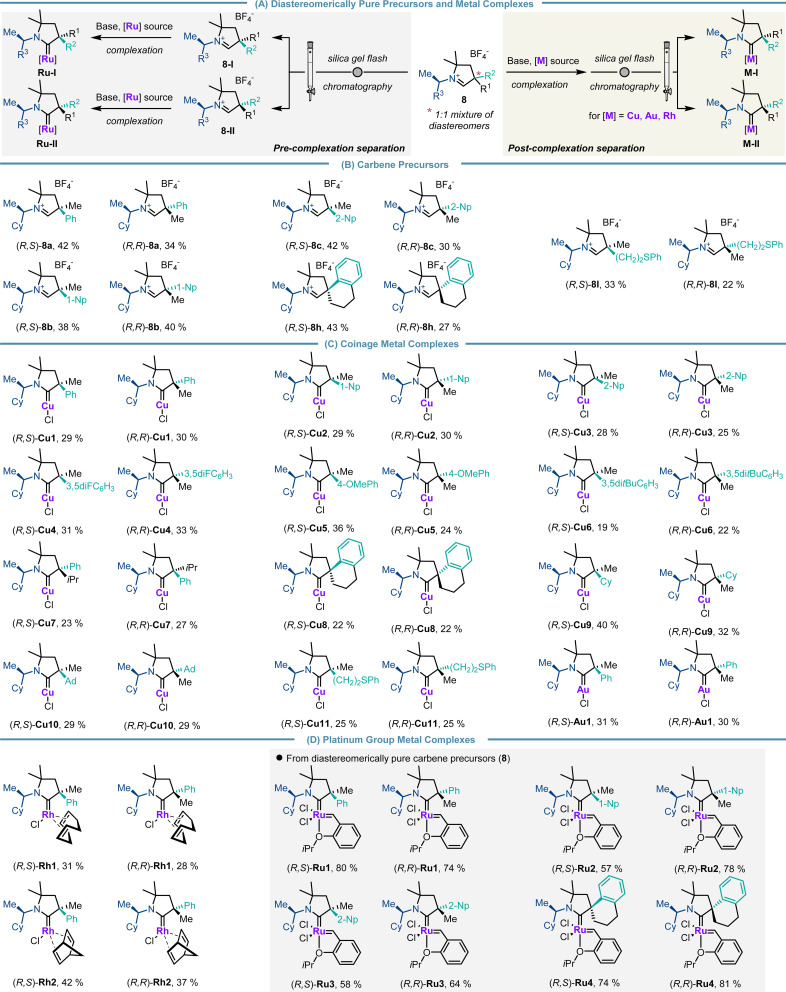
Prepared chiral CAAC precursors and their transition-metal complexes.

Having established a robust and reliable route to access a diverse library of CAAC precursors, we turned our focus to simple separation of the diastereomers of the parent carbene precursors as well as the related downstream CAAC transition metal complexes (Scheme 6A). The use of routine separation by silica gel flash chromatography of diastereomers instead of preparative chiral HPLC for enantiomer resolution is an appreciable simplification. Notably, diastereomerically pure iminiums 8a–8d were consistently obtained in isolated yields ranging from 27% to 43% (note: with the initial 1 : 1 diastereomeric mixture of CAAC precursors the maximal yield for diastereomerically pure precursors is 50%) (Scheme 6B). Conveniently, the diastereomers of CAAC copper (Cu1–Cu11), gold (Au1) and rhodium complexes (Rh1 and Rh2) were also smoothly separated by silica gel flash chromatography. Consistently, both diastereomers were isolated with equal efficiency in yields ranging from 19% to 42% (note: with the 1 : 1 diastereomeric mixture of CAAC precursors the maximal yield for diastereomerically pure complexes is 50%) (Scheme 6C and D). Ruthenium complexes Ru1–Ru4 were prepared utilizing the diastereomerically pure iminium salts as the separation of diastereomers at the metal complex stage was not successful for these examples (Scheme 6d).

**Fig. 1:**
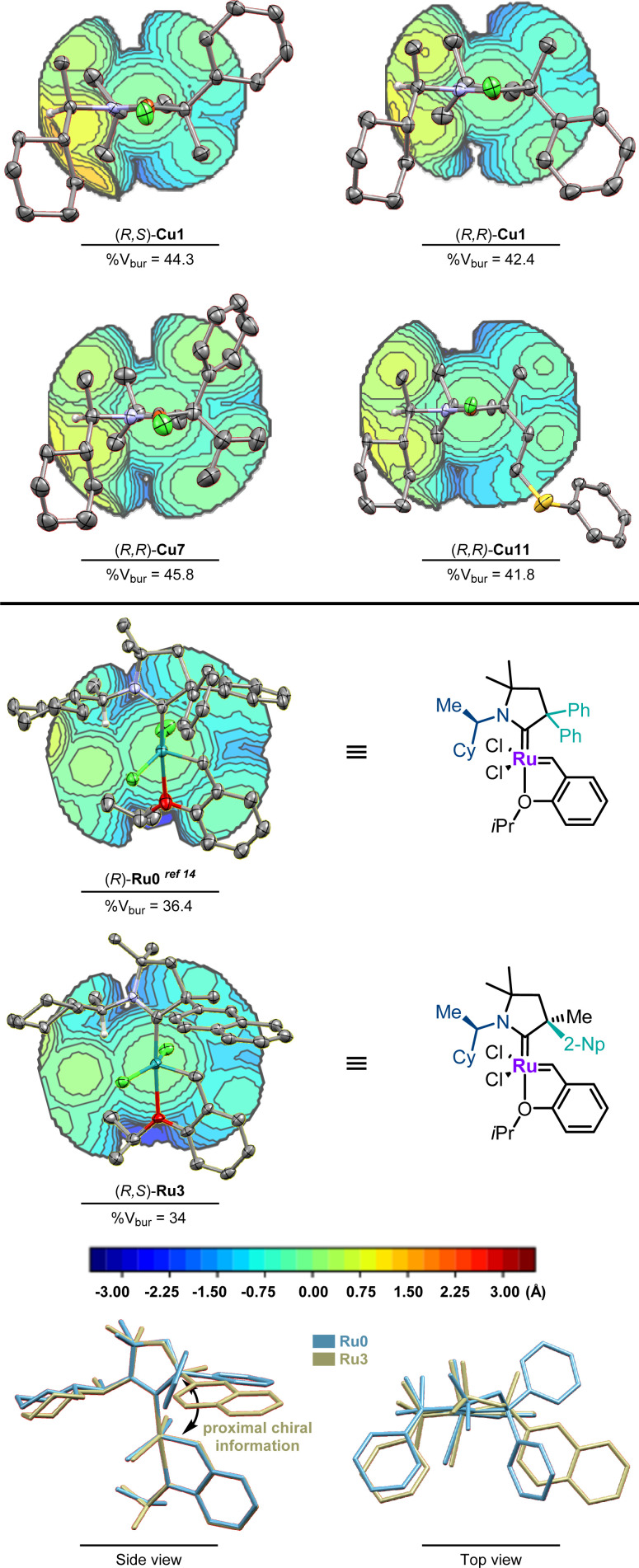
ORTEP plots (50% probability thermal ellipsoids and 30% for Cu7, Ru0 and Ru3, hydrogen atoms are omitted for clarity), topological steric maps of (*R*,*S*)-Cu1 and (*R*,*R*)-Cu1, (*R*,*R*)-Cu7 and Cu11, and (*R*)-Ru0 and (*R*,*S*)-Ru3 and structure overlay of (*R*)-Ru0 and (*R*,*S*)-Ru3. The plotted topological steric maps^23^ and calculated buried volumes (% V_Bur_)^24^ were obtained from SambVca2.1 (ref. 25) (Bondi radii scaled by 1.17, sphere radius 3.5 Å, and mesh spacing 0.1 Å).

The relative and absolute configurations of the CAAC ligands were determined by single-crystal X-ray diffraction analysis of a set of copper and ruthenium complexes (Fig. 1). The diastereomer of (*R*,*S*)-Cu1 adopts a pseudo-*C*_2_-symmetrical geometry placing the largest substituents in diagonally opposed quadrants. In contrast, the (*R*,*R*)-diastereomer of Cu1 displays a crowded southern hemisphere and a rather accessible northern hemisphere. With either two bulky (Cu7) or two small substituents (Cu11) at the chiral α-quaternary stereogenic center, the quadrant distinction becomes less pronounced. Contrasting the pseudo-*C*_2_-symmetrical copper complexes, ruthenium complex (*R*,*S*)-Ru3 shows a single crowded hemisphere minimizing interactions between the cyclohexyl group and both chlorides. The steric parameters were similar to those of α-*gem*-bis-phenyl substituted (*R*)-Ru0.^14^ In archetypical *N*-aryl substituted CAACs, the aromatic ring is placed in proximity to the benzylidene unit forcing the quaternary carbon atom bearing the chiral information away from the metathesis initiating part of the catalyst. Such an “inverted”-CAAC orientation^21,22^ resulting in a proximal chiral environment sitting above the benzylidene bond might be an exploitable structural feature for enantioselective catalysis. The suspected critical relevance of these design features with respect to the ability to induce enantioselectivity in catalytic application was tested next.

In order to evaluate the catalytic performance of the synthesized chiral CAAC ligands, the corresponding copper complexes Cu1–Cu11 were benchmarked in enantioselective conjugate borylation of α,β-unsaturated ester 9 (Table 1). The complexes were grouped as diastereomeric pairs. Notably a clear trend for the matching/mismatching pair of the stereogenic center was observed. The complexes with the (*R*,*S*)-configured CAAC complex (Cu1–Cu5 and Cu8) with an aryl/methyl substitution pattern delivered borylated ester 10 in excellent yields (86–92%) and with good enantioselectivities ranging from 87 : 13 to 91 : 9 er. Complexes with an alkyl/alkyl stereocenter (Cu9 and Cu10) displayed a reduced reactivity, diminished yields and lower selectivities. Reducing the size difference of the substituent of the a-stereocenter by replacing the methyl group with an isopropyl group (Cu7) caused a substantial drop in the observed enantioselectivity. The series of complexes with (*R,R*)-configured CAAC ligands was clearly confirmed to have mismatching stereocenters. In these cases, ester product 10 was consistently formed in substantially inferior enantioselectivities, experimentally supporting the selectivity hypothesis derived from the steric maps. To further assess catalytic activity, the two best performing catalysts Cu2 and Cu8 were tested at a reduced reaction temperature of −20 °C. Under these conditions, catalyst Cu2 provided 10 in 65% yield with an identical enantioselectivity of 89 : 11 er. Catalyst Cu8 maintained its excellent reactivity giving 10 in 95% yield with an improved selectivity of 95 : 5 er.

**Table 1:** Catalytic performance of the chiral CuCAACs in the asymmetric conjugate borylation (ACB) reaction^a^

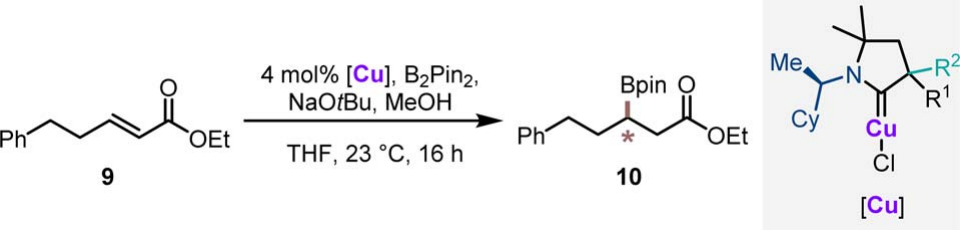			
Entry	[Cu]	% Yield of 10^b^	er^c^
1	(*R*,*S*)-Cu1	86	87 : 13
2	(*R*,*R*)-Cu1	90	62.5 : 37.5
3	(*R*,*S*)-Cu2	92	89 : 11
4^d^	(*R*,*S*)-Cu2	65	89 : 11
5	(*R*,*R*)-Cu2	95	59 : 41
6	(*R*,*S*)-Cu3	92	89 : 11
7	(*R*,*R*)-Cu3	94	62 : 38
8	(*R*,*S*)-Cu4	87	86 : 14
9	(*R*,*R*)-Cu4	94	60 : 40
10	(*R*,*S*)-Cu5	90	88 : 12
11	(*R*,*R*)-Cu5	90	63 : 37
12	(*R*,*S*)-Cu6	89	77 : 23
13	(*R*,*R*)-Cu6	93	79 : 21
14	(*R*,*R*)-Cu7	98	72 : 28
15	(*R*,*S*)-Cu7	97	55 : 45
16	(*R*,*S*)-Cu8	92	91 : 9
**17^d^**	**(*R*,*S*)-Cu8**	**95**	**95 : 5**
18	(*R*,*R*)-Cu8	90	45 : 55
19	(*R*,*S*)-Cu9	63	83 : 17
20	(*R*,*R*)-Cu9	78	55 : 45
21	(*R*,*S*)-Cu10	75	80 : 20
22	(*R*,*R*)-Cu10	99	54 : 46
23	(*R*,*S*)-Cu11	87	69 : 31
24	(*R*,*R*)-Cu11	84	58 : 42

a Reaction conditions: 0.1 mmol 9, 4 mol% [Cu], 20 mol% NaOtBu, 1.1 equiv. B_2_Pin_2_, 2.0 equiv. MeOH, 0.2 M in THF at 23 °C for 16 h.

b Isolated yield.

c Enantiomeric ratio determined by chiral HPLC after oxidation to the corresponding secondary alcohol.

d Reaction was performed at −20 °C.

The CAAC ruthenium complexes Ru1–Ru4 and additionally (*R*)-Ru0 (ref. 14) were evaluated in asymmetric ring closing metathesis (ARCM) of triene 11 as the second benchmark transformation (Table 2). Ru0 provided 12 in 62% yield with an encouraging 73 : 27 er (entry 1). In all cases, the catalysts having (*R*,*S*)-diastereomeric CAAC proved to have the matched pair of stereogenic centers for this transformation (entries 2–5). (*R*,*S*)-Ru3 emerged as the best performer, providing dihydrofuran 12 in 60% yield and 92 : 8 er (entry 4). (*R*,*S*)-Ru4 was not competent in this transformation and seemed prone to decomposition under the reaction conditions. In general, increasing the reaction temperature to 40 °C improved the reaction yields with a very small reduction of the enantioselectivities (entries 6–9). The series of ruthenium complexes having the (*R*,*R*)-configurations at the CAAC ligand clearly displayed mismatching characteristics (entries 10–13). The observed enantioselectivities were moderate. (*R*,*R*)-Ru3 showed the highest selectivity in this series (24 : 76 er) in favor of the enantiomeric product 12. This behavior underlines the dominance of the proximal α-stereogenic center in enantioselection. Noteworthily, (*R*,*S*)-Ru2 and (*R*,*R*)-Ru2 appeared as a 1 : 0.7 and 1 : 1 rotamer mixture in ^1^H-NMR in CD_2_Cl_2_. The negative impact of rotamers for selectivity resonated with the observations previously reported suggesting an impaired catalytic performance due to the formation of rotamers prior to the enantio-determining step.^26^

**Table 2:** Catalytic performance of the chiral RuCAACs in the asymmetric ring closing metathesis^a^

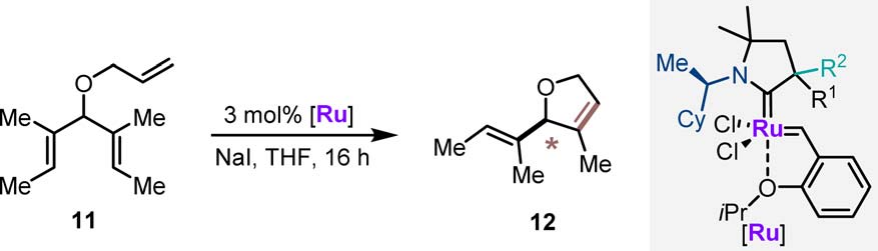					
	Entry	[Ru]	Temp. (°C)	% Yield of 12^c^	er^d^
Match	1^b^	(*R*)-**Ru0**	23	62	27 : 73
	2	(*R*,*S*)-**Ru1**	23	39	89.5 : 10.5
	3	(*R*,*S*)-**Ru2**	23	21	65 : 35
	**4**	**(*R*,*S*)-Ru3**	**23**	**60**	**92** **:** **8**
	5	(*R*,*S*)-**Ru4**	23	<1	n.d.
Match	6	(*R*,*S*)-**Ru1**	**40**	54	88.5 : 11.5
	7	(*R*,*S*)-**Ru2**	**40**	72	65 : 35
	8	(*R*,*S*)-**Ru3**	**40**	79	90 : 10
	9	(*R*,*S*)-**Ru4**	**40**	<3	n. d.
Mismatch	10	(*R*,*R*)-**Ru1**	23	84	34 : 66
	11	(*R*,*R*)-**Ru2**	23	27	45 : 55
	12	(*R*,*R*)-**Ru3**	23	80	24 : 76
	13	(*R*,*R*)-**Ru4**	23	23	45 : 55

a Reaction conditions: 0.05 mmol 11, 3 mol% [Ru], 0.75 equiv. NaI, 0.07 M in THF for 16 h.

b With 5 mol% [Ru], 0.5 M in THF.

c Determined by NMR using 1,3,5-trimethoxybenzene as the internal standard.

d Enantiomeric ratio was determined by chiral GC.

## Conclusions

In summary, we devised a streamlined strategy to assemble a library of diastereo- and enantiomerically pure CAAC ligands featuring notoriously difficult to access α-quaternary stereogenic centers. A universal Julia–Kocienski olefination reagent allowed the Claisen-rearrangement to be leveraged as an expedient strategy forming the racemic α-chiral aldehydes as key intermediates for the CAAC synthesis. Condensation with chiral amines and subsequent cyclization provided diastereomeric mixtures of the targeted ligand precursors. These precursors, as well as their corresponding copper, gold and rhodium complexes, are conveniently separable using standard silica gel flash chromatography to diastereo- and enantiomerically pure materials. The availability of both diastereomers enabled the testing of the synergistic effects of the two chiral centers in catalytic applications. The catalytic performances of the corresponding chiral CAAC copper and ruthenium complexes were evaluated in enantioselective conjugate borylations and asymmetric ring closing metathesis, respectively. Our approach closes a gap in chiral ligand accessibility enabling the synthesis of various chiral CAAC transition metal complexes previously beyond reach. We firmly believe that the outlined streamlined and modular synthetic strategy provides substantial leverage to design further chiral CAACs able to bridge method limitations in challenging catalytic enantioselective transformations.

## Author contributions

A. M. d. V. and N. C. conceived and conceptualized the project. A. M. d. V. designed and performed the experiments. Analysis of all experiments was performed by A. M. d. V. and N. C. Interpretation of the results and writing and revision of the manuscript was performed by all authors. N. C. provided funding and resources.

## Conflicts of interest

There are no conflicts to declare.

## Supplementary Material

SC-015-D4SC04278F-s001

SC-015-D4SC04278F-s002

## Data Availability

Experimental details and characterization data are available free of charge from the ESI† available with this article. Crystallographic data are available at CCDC (see note in ref. 27).
