# Supplementary material for: Streamlined synthetic assembly of α-chiral CAAC ligands and catalytic performance of their copper and ruthenium complexes
Source: Chem Sci. 2024 Jul 24;15(34):13864–71. doi: 10.1039/d4sc04278f (PMC11306997; doi:10.1039/d4sc04278f)
Supplement: SC-015-D4SC04278F-s001 [file SC-015-D4SC04278F-s001.pdf]

Supplementary Information (SI) for Chemical Science.
This journal is © The Royal Society of Chemistry 2024

Streamlined Synthetic Assembly of α-Chiral CAAC Ligands and Catalytic Performance of Their Copper and Ruthenium Complexes

Adrien Madron du Vigné and Nicolai Cramer*

Laboratory of Asymmetric Catalysis and Synthesis, Institute of Chemical Sciences and Engineering, Ecole Polytechnique Fédérale de Lausanne (EPFL), SB-ISIC, BCH4305, 1015 Lausanne, Switzerland

*e-mail: nicolai.cramer@epfl.ch

Supplementary Information

Table of Content:

General methods and material S2
Ketone synthesis S3
Julia-Kocienski reagent preparation S5
Claisen route S8
Diastereomeric carbene precursors synthesis S13
Diastereomerically pure carbene precursors S21
Transition metal complexes S27
Asymmetric Conjugate Borylation (ACB) S47
Asymmetric Ring Closing Metathesis (ARCM) S73
Crystallographic data S84
References S88
NMR Spectra S89

General methods and material. All reactions were carried out under inert atmosphere in oven-dried glassware with magnetic stirring, unless otherwise indicated. Reagents and solvents were purchased from Aldrich/Merck, Acros, Alfa Aesar, Abcr or TCI and used as obtained from the suppliers. $CuCl(SMe_2)$,[1] $AuCl(SMe_2)$,[2] $[Rh(nbd)Cl]_2$[3] and $[Rh(COD)Cl]_2$[3] were prepared according to reported procedures. (*R*)-1-cyclohexylethan-1-amine and Hoveyda-Grubbs Catalyst® M700 were purchased from Aldrich/Merck and used without further purification. Dry diethyl ether (Et_2O), dichloromethane (DCM), toluene and tetrahydrofuran (THF) were purified by an Innovative Technology Solvent Delivery System. Ethyl acetate (EtOAc), dichloromethane (DCM) and pentane used for filtration, transfers, chromatography and recrystallizations were purchased from commercial sources and distilled before use. THF used in complexation and catalytic reactions was degassed via freeze-pump-thaw technique and stored over 4Å molecular sieves in a nitrogen-filled glove box. Column chromatography was performed with Silicycle silica gel SiliaFlash P60 (40-63 μm grade) and SiliaFlash 40A (40-63 μm grade). Analytical thin-layer chromatography was performed with commercial glass plates coated with 0.25 mm silica gel (E. Merck, Kieselgel 60 F254). R_f values reported were measured using a 5 × 2 cm plate. Visualization was accomplished by UV light (254 nm), or by dipping the plate in Cerium Ammonium Molybdate (CAM) or aqueous potassium permanganate ($KMnO_4$) solutions followed by heating. Proton nuclear magnetic resonance (^1H NMR), proton decoupled carbon-13 nuclear magnetic resonance ($^{13}C\ \{^1H\}$ NMR) and fluorine-19 nuclear magnetic resonance (^{19}F NMR) spectra were recorded on a Bruker AVANCEIII-400 spectrometer (400 MHz, ^1H; 101 MHz, ^{13}C; 376 MHz, ^{19}F) or on a Bruker AVANCEII-800 (800 MHz, ^1H; 201 MHz, ^{13}C) spectrometer. Chemical shifts are reported in parts per million (ppm). ^1H NMR spectra are referenced to residual $CHCl_3$ in $CDCl_3$ (s, δ = 7.26 ppm), $CDHCl_2$ in CD_2Cl_2 (t, δ = 5.32 ppm) or C_4D_7HO in THF-d_8 (m, δ = 3.58 ppm and 1.73 ppm) and ^{13}C NMR spectra are referenced to deuterated chloroform (t, δ = 77.16 ppm) or deuterated dichloromethane (p, δ = 53.84 ppm) according to the literature.[4] Splitting patterns are designated as s, singlet; d, doublet; t, triplet; q, quartet; hept, heptuplet; m, multiplet; br, broad. All NMR data were recorded at 298 K. Infrared (IR) data were recorded on an Alpha-P Bruker FT-IR Spectrometer. Absorbance frequencies are reported in reciprocal centimeters (cm^{-1}). HRMS data were acquired on an Agilent LC-MS TOF (Multimode: ESI + APCI) or on a LTQ Orbitrap FTMS instrument (LTQ Orbitrap Elite FTMS, Thermo Scientific, Bremen, Germany) equipped with an Ion Max APPI ionization source with a VUV Kr lamp (Syagen, CA, USA). The sample was diluted in DCM and directly infused into the mass spectrometer. FT-MS spectra were recorded in the 100-1000 *m/z* range in the positive mode with a resolution set to 120,000. Optical rotations were measured at 21 °C on a Polartronic M polarimeter using a 10.0 cm cell with a Na 589 nm filter. Melting points were measured on a Büchi B-540 and are uncorrected. X-ray analysis of compounds (*R*,*S*)- and (*R*,*R*)-**Cu1**, (*R*,*R*)-**Cu7,** (*R*,*R*)-**Cu11,** (*R*,*S*)-**Ru3** and (*R*)-**Ru0** were performed by Dr. R. Scopelliti and Dr. F. Tirani at the EPF Lausanne. HRMS analyses were performed by Dr. L. Menin team at the mass spectroscopy platform of ISIC at the EPF Lausanne.

Ketone synthesis

Alcohol preparation

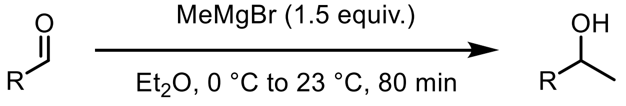

S-I **S-II**

General Procedure (GP1):

In a two-necked round-bottom flask equipped with a magnetic stirrer and nitrogen inlet, the aldehyde (1.0 equiv.) was dissolved in dry diethyl ether (0.3 M). The solution was cooled to 0 °C and MeMgBr (3.0 M, 1.5 equiv.) was added dropwise via syringe. The reaction mixture was stirred at 0 °C for 15 min before being allowed to warm to 23 °C and stirred for 80 min (complete by TLC). The mixture was cooled to 0 °C and quenched with aqueous saturated NH_4Cl (8 mL/mmol) and water (4 mL/mmol). After warming to 23 °C, layers were separated and the aqueous layer was extracted three times with EtOAc (3 × 12 mL/mmol). The combined organic layers were dried over $MgSO_4$, filtered and solvents removed under reduced pressure to afford the desired alcohol, used without further purification.

1-(3,5-di-tert-butylphenyl)ethan-1-ol (S-IIa)

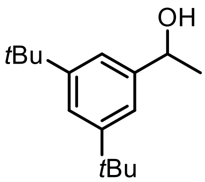

Following **GP1**: 3,5-di-tert-butylbenzaldehyde (1.80 g, 7.82 mmol) gave 1-(3,5-di-tert-butylphenyl)ethan-1-ol **S-IIa** (1.83 g, 7.82 mmol, 99 %) as a white solid. The characterization data matched those previously reported in the literature.[5]

^{1}H NMR (400 MHz, $CDCl_3$) δ 7.36 (t, *J* = 1.9 Hz, 1H), 7.23 (d, *J* = 1.8 Hz, 2H), 4.90 (q, *J* = 6.5 Hz, 1H), 1.78 (s, 1H), 1.52 (d, *J* = 6.5 Hz, 3H), 1.34 (s, 18H).

1-(4-methoxyphenyl)ethan-1-ol (S-IIb)

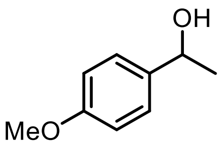

Following **GP1**: 4-methoxybenzaldehyde (501 mg, 3.61 mmol) gave 1-(4-methoxyphenyl)ethan-1-ol **S-IIb** (514 mg, 3.38 mmol, 94 %) as a colorless oil. The characterization data matched those previously reported in the literature.[6]

^{1}H NMR (400 MHz, $CDCl_3$) δ 7.33–7.29 (m, 2H), 6.91–6.87 (m, 2H), 4.86 (qd, *J* = 6.4, 3.5 Hz, 1H), 3.81 (s, 3H), 1.71 (br s, 1H), 1.48 (d, *J* = 6.4 Hz, 3H).

Alcohol oxidation

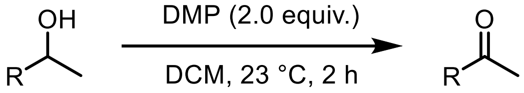

S-II **S-III**

1-(3,5-di-tert-butylphenyl)ethan-1-one (S-IIIa)

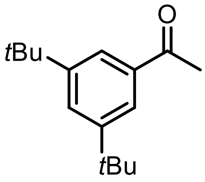

The preparation of 1-(3,5-di-tert-butylphenyl)ethan-1-one was adapted from a literature procedure and the characterization data matched those previously reported.[5]

To a solution of 1-(3,5-di-tert-butylphenyl)ethan-1-ol (355 mg, 1.52 mmol, 1.0 equiv.) in DCM (15 mL, 0.1 M) at 23 °C was added Dess-Martin periodinane (1.39 g, 3.16 mmol, 2.0 equiv.) in two portions separated by 30 min. The reaction was complete by TLC after a total of 1 h reaction time. The reaction mixture was diluted with 5:95 EtOAc/pentane (30 mL) and allowed to stir vigorously 20 min to precipitate a white solid. Filtration through a short plug of silica gel (1:9 EtOAc/pentane) afforded 1-(3,5-di-tert-butylphenyl)ethan-1-one **S-IIIa** (301 mg, 1.30 mmol, 86 %) as a pale-yellow solid.

^{1}H NMR (400 MHz, $CDCl_3$) δ 7.81 (d, *J* = 1.9 Hz, 2H), 7.65 (t, *J* = 1.9 Hz, 1H), 2.61 (s, 3H), 1.36 (s, 18H).

Michael addition

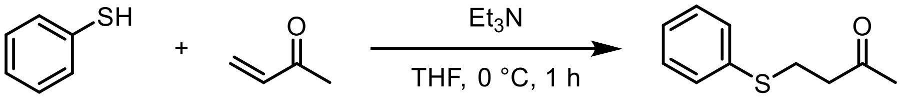

S-IV

4-(phenylthio)butan-2-one (S-IV)

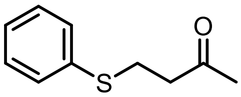

Methyl vinyl ketone (1.0 equiv.) was carefully added dropwise at 0 °C to a mixture of thiophenol (1.0 equiv.) and triethylamine (1.2 equiv.). The mixture was left to stir for 1 h (complete conversion - ^{1}H NMR). The reaction mixture was concentrated under reduced pressure, quantitatively affording 4-(phenylthio)butan-2-one **S-IV** used for the next step without further purification. The characterization data matched those previously reported in the literature.[7]

^{1}H NMR (400 MHz, $CDCl_3$) δ 7.38–7.29 (m, 4H), 7.25–7.21 (m, 1H), 3.17 (t, *J* = 7.3 Hz, 2H), 2.79 (t, *J* = 7.3 Hz, 2H), 2.18 (s, 3H).

Julia-Kocienski reagent preparation

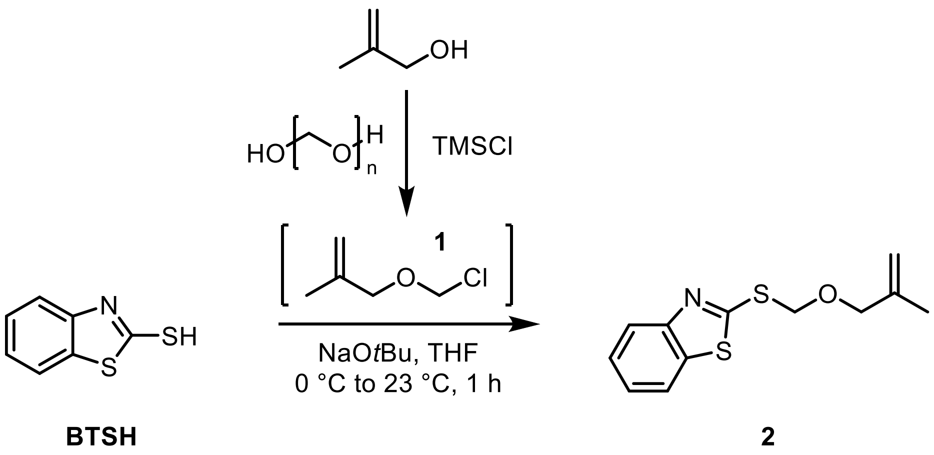

2-((((2-methylallyl)oxy)methyl)thio)benzo[d]thiazole (2)

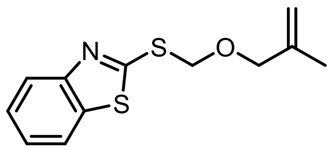

TMSCl (1.78 mL, 13.6 mmol, 1.0 equiv.) was added dropwise at -10 °C to a suspension of paraformaldehyde (406 mg, 13.6 mmol, 1.0 equiv.) in methallyl alcohol (1.0 g, 13.6 mmol, 1.0 equiv.). The suspension was vigorously stirred for 90 min at -10 °C. An aliquot was taken after 75 min for NMR assessment of 3-(chloromethoxy)-2-methylprop-1-ene **1** purity (ca. 75 % over 10 repeats - Figure S1).

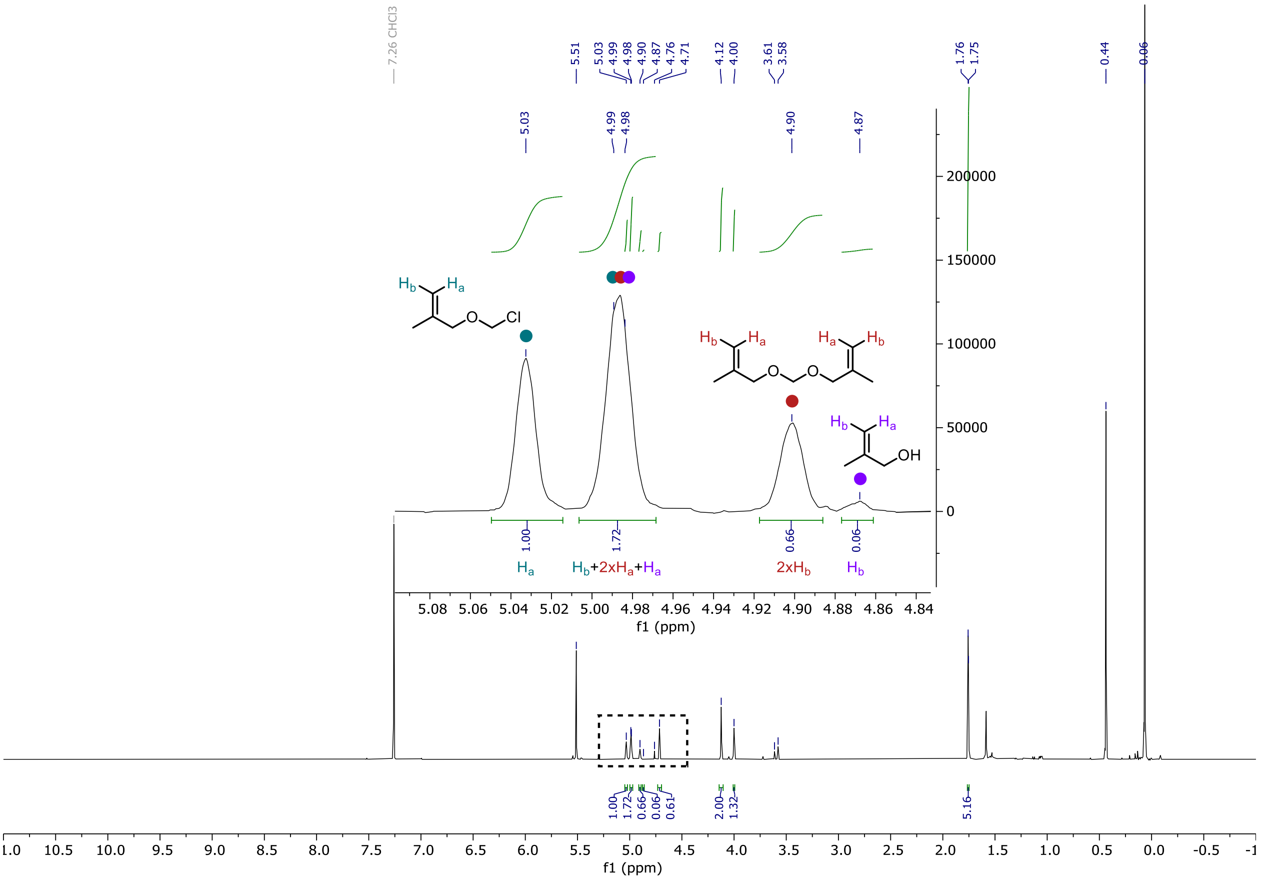

Figure S1. *Reaction mixture aliquot containing 3-(chloromethoxy)-2-methylprop-1-ene* ***1*** *at t = 75 min.*

The reaction mixture containing 3-(chloromethoxy)-2-methylprop-1-ene **1** (1.23 g, 10.2 mmol, 1.2 equiv.) was diluted at -10 °C with THF (10 M) followed by filtration under nitrogen atmosphere over a small pad of THF-wet silica (ca. 1 cm) directly into a separate flask containing a mixture of 2-mercaptobenzothiazole (**BTSH**) (1.42 g, 8.50 mmol, 1.0 equiv.) and NaO*t*Bu (927 mg, 9.35 mmol, 1.1 equiv.) in THF (1 M) at 0 °C. The mixture was warmed up to 23 °C and was left to stir for 1 h. The reaction mixture was filtered over a DCM-wet pad of silica, thoroughly washed with DCM. After removal of the solvents under reduced pressure, the residue was purified by column chromatography (conditioning: 1:9 DCM/pentane, eluent: 2:8 DCM/pentane to 6:4 DCM/pentane by 10 % increment) to afford the desired sulfide **2** (1.33 g, 5.28 mmol, 62 % yield, 96 % purity) as a colorless oil.

^{1}H NMR (400 MHz, $CDCl_3$) δ 7.92 (ddd, *J* = 8.2, 1.2, 0.6 Hz, 1H), 7.78 (ddd, *J* = 8.0, 1.3, 0.7 Hz, 1H), 7.43 (ddd, *J* = 8.3, 7.3, 1.3 Hz, 1H), 7.32 (ddd, *J* = 8.4, 7.3, 1.2 Hz, 1H), 5.55 (s, 2H), 5.03–5.01 (m, 1H), 4.98–4.96 (m, 1H), 4.13 (s, 2H), 1.76 (br s, 3H). **^{13}C NMR** (101 MHz, $CDCl_3$) δ 165.4, 153.1, 140.8, 135.8, 126.3, 124.7, 122.1, 121.1, 114.1, 73.8, 73.1, 19.8. **IR** (ATR, neat) 2973, 2913, 2853, 1456, 1426, 1309, 1239, 1092, 996, 904, 756. **HRMS** (ESI/QTOF) m/z: $[M+H]^+$ calculated for $C_{12}H_{14}NOS_2^+$ 252.0511; found 252.0515. **R_f** = 0.23 (1:1 DCM/pentane, UV, CAM).

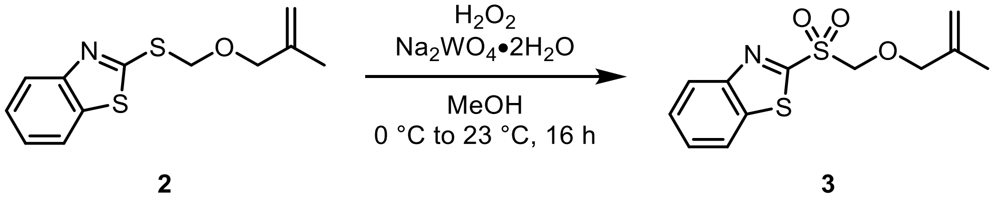

2-((((2-methylallyl)oxy)methyl)sulfonyl)benzo[d]thiazole (3)

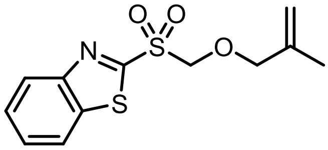

To a solution of sulfide **2** (1.33 g, 5.28 mmol, 1.0 equiv.) in methanol (26 mL, 0.2 M) at 0 °C was added sodium tungstate dihydrate (880 mg, 2.64 mmol, 0.5 equiv.). Hydrogen peroxide 30 % aqueous (2.2 mL, 21.5 mmol, 4 equiv.) was added 5 min later. The reaction was stirred at 23 °C for 16 h. The reaction mixture was quenched with 10 % aqueous sodium metabisulfite (0.57 g/mmol sulfide) at 0 °C and diluted with DCM (50 mL). The mixture was extracted 3 times with DCM (3 × 25 mL). The combined organic layers were dried over $MgSO_4$, filtered and concentrated under reduced pressure. The residue was purified on Biotage Isolera (0 to 30 % EtOAc/hexane over 15 CV, 80 mL/min, 50 g silica gel cartridge) to give the desired sulfone **3** (1.28 g, 4.51 mmol, 85 % yield, 96 % purity) as a white solid.

^{1}H NMR (400 MHz, $CDCl_3$) δ 8.26–8.24 (m, 1H), 8.04–8.01 (m, 1H), 7.67–7.58 (m, 2H), 4.98 (s, 2H), 4.98–4.96 (m, 2H), 4.30 (s, 2H), 1.68 (t, J = 1.2 Hz, 3H). **^{13}C NMR** (101 MHz, $CDCl_3$) δ 164.6, 153.0, 139.8, 137.4, 128.3, 127.8, 125.8, 122.5, 115.4, 83.9, 77.1, 19.4. **IR** (ATR, neat) 2917, 1470, 1334, 1301, 1149, 1114, 1084, 901, 760, 729. **HRMS** (ESI/QTOF) m/z: $[M+Na]^+$ calculated for $C_{12}H_{13}NNaO_3S_2^+$ 306.0229; found 306.0219. **mp** 70.3–72.4 °C. **R$_f$** = 0.35 (2:8 EtOAc/hexane, UV, CAM).

Claisen route

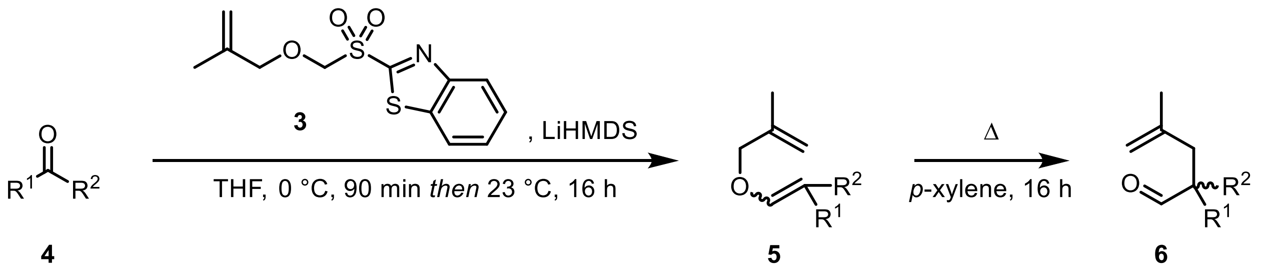

General Procedure (GP2):

To a solution of sulfone **3** (1.0 equiv.) and ketone **4** (1.0 equiv.) in THF (0.085 M) at 0 °C, was added LiHMDS (1.4 equiv., 1 M in THF) dropwise over 2-5 min. The reaction was stirred at 0 °C for 90 min then 23 °C for 16 h. The reaction mixture was quenched with saturated aqueous NH_4Cl, extracted 3 times with EtOAc, washed with brine, dried over $MgSO_4$ and concentrated under reduced pressure. The crude product was passed over a plug of silica (1:9 DCM/pentane) to afford the crude allyl vinyl ether **5** as a colorless to pale-yellow oil (as an inconsequential E/Z mixture) used without further purification.

General Procedure (GP3):

Dry *p*-xylene (0.2 M) was added to the allyl vinyl ether **5** (1.0 equiv.) in a microwave tube whereupon argon or nitrogen was bubbled for 5 min. The tube was sealed and the mixture was heated to 150 °C (oil bath) for 16 h. The reaction mixture was directly purified by column chromatography (conditioning: pentane, eluent: pentane until xylene was out then 1:9 DCM/pentane to 1:1 DCM/pentane by 10 % increment) to afford the desired aldehyde **6**.

(1-((2-methylallyl)oxy)prop-1-en-2-yl)benzene (5a)

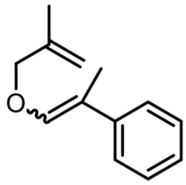

Following **GP2**: acetophenone (444 mg, 3.69 mmol) gave the title product **5a** as a colorless oil (437 mg, 2.32 mmol, 63 %) in an inconsequential 1.42:1 E/Z mixture after filtration over a short pad of silica (1:9 DCM/pentane *then* 2:8 DCM/pentane). **^{1}H NMR** (400 MHz, $CDCl_3$) δ 7.68–7.66 (m, 2H), 7.36–7.26 (m, 6H), 7.20–7.16 (m, 2H), 6.48 (q, J = 1.4 Hz, 1H), 6.18 (q, J = 1.3 Hz, 1H), 5.04–5.02 (m, 2H), 4.96–4.94 (m, 2H), 4.29–4.28 (m, 2H), 4.25–4.24 (m, 2H), 2.04 (d, J = 1.3 Hz, 3H), 1.93 (d, J = 1.3 Hz, 3H), 1.79–1.78 (m, 3H), 1.77–1.76 (m, 3H). **^{13}C NMR** (101 MHz, $CDCl_3$) δ 143.6, 143.1, 141.8, 141.7, 140.8, 138.5, 128.4, 128.0, 127.6, 126.1, 126.0, 125.1, 114.7, 112.8, 112.8, 110.7, 76.5, 76.1, 19.4, 19.4, 18.5, 12.8. **IR** (ATR, neat) 3055, 2973, 2921, 2859, 1650, 1599, 1496, 1443, 1154, 1140, 1076, 903, 759, 694. **HRMS** (ESI/QTOF) m/z: $[M+Ag]^+$ calculated for $C_{13}H_{16}AgO^+$ 295.0247; found 295.0250. **R_f** = 0.51 (1:9 DCM/pentane, CAM).

2,4-dimethyl-2-phenylpent-4-enal (6a)

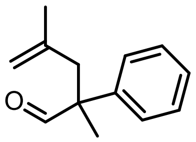

Following **GP2**/**GP3**: acetophenone (124 mg, 1.02 mmol) gave the title product **6a** as a colorless oil (111 mg, 0.59 mmol, 57 % over 2 steps). **^{1}H NMR** (400 MHz, $CDCl_3$) δ 9.55 (s, 1H), 7.40–7.35 (m, 2H), 7.31–7.27 (m, 3H), 4.81 (dq, *J* = 2.9, 1.6 Hz, 1H), 4.62 (dq, *J* = 1.9, 0.9 Hz, 1H), 2.73 (dd, *J* = 13.9, 1.0 Hz, 1H), 2.65 (dd, *J* = 13.9, 1.0 Hz, 1H), 1.47 (s, 3H), 1.40 (dd, *J* = 1.4, 0.8 Hz, 3H). **^{13}C NMR** (101 MHz, $CDCl_3$) δ 202.1, 141.6, 139.9, 128.9, 127.5, 115.6, 53.6, 44.4, 24.3, 18.7. **IR** (ATR, neat) 3074, 2970, 2940, 2804, 2709, 1721, 1445, 894, 759, 698. **HRMS** (Sicrit plasma/LTQ-Orbitrap) m/z: $[M+H]^+$ calculated for $C_{13}H_{17}O^+$ 189.1274; found 189.1274. **R*f*** = 0.47 (1:9 DCM/pentane, CAM).

2,4-dimethyl-2-(naphthalen-1-yl)pent-4-enal (6b)

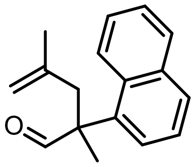

Following **GP2**/**GP3**: 1-acetonaphtone (173 mg, 1.00 mmol) gave the title product **6b** as a colorless oil (175 mg, 0.74 mmol, 74 % over 2 steps). **^{1}H NMR** (400 MHz, $CDCl_3$) δ 9.73 (s, 1H), 7.92–7.88 (m, 1H), 7.87–7.80 (m, 2H), 7.51–7.46 (m, 4H), 4.77 (dq, *J* = 3.0, 1.5 Hz, 1H), 4.54 (dq, *J* = 1.8, 0.9 Hz, 1H), 3.03 (d, *J* = 13.6 Hz, 1H), 2.96 (dd, *J* = 13.7, 0.8 Hz, 1H), 1.59 (s, 3H), 1.11 (dd, *J* = 1.5, 0.8 Hz, 3H). **^{13}C NMR** (101 MHz, $CDCl_3$) δ 204.9, 141.8, 136.5, 134.6, 131.7, 129.7, 129.2, 126.6, 126.4, 125.7, 125.4, 124.6, 115.7, 54.3, 42.9, 24.1, 21.0. **IR** (ATR, neat) 2967, 1720, 1640, 1510, 1459, 1375, 896, 801, 777. **HRMS** (ESI/QTOF) m/z: $[M+H]^+$ calculated for $C_{17}H_{19}O^+$ 239.1430; found 239.1431. **R*f*** = 0.27 (1:9 DCM/pentane, CAM).

2,4-dimethyl-2-(naphthalen-2-yl)pent-4-enal (6c)

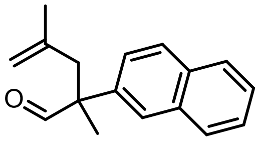

Following **GP2**/**GP3**: 2-acetonaphtone (551 mg, 3.14 mmol) gave the title product **6c** as a colorless oil (491 mg, 2.06 mmol, 66 % over 2 steps). **^{1}H NMR** (400 MHz, $CDCl_3$) δ 9.60 (s, 1H), 7.87–7.82 (m, 3H), 7.75 (d, *J* = 2.0 Hz, 1H), 7.52–7.47 (m, 2H), 7.41 (dd, *J* = 8.7, 2.0 Hz, 1H), 4.82 (dt, *J* = 3.0, 1.6 Hz, 1H), 4.66 (dq, *J* = 2.0, 1.0 Hz, 1H), 2.83 (dd, *J* = 13.9, 1.0 Hz, 1H), 2.79 (dd, *J* = 14.0, 1.0 Hz, 1H), 1.58 (s, 3H), 1.42 (s, 3H). **^{13}C NMR** (101 MHz, $CDCl_3$) δ 202.1, 141.6, 137.3, 133.5, 132.5, 128.6, 128.2, 127.7, 126.5, 126.5, 126.4, 125.4, 115.6, 53.8, 44.2, 24.4, 18.9. **IR** (ATR, neat) 3058, 2969, 2705, 1721, 1643, 1598, 1506, 1455, 1376, 950, 895, 816. **HRMS** (ESI/QTOF) m/z: $[M+H]^+$ calculated for $C_{17}H_{19}O^+$ 239.1430; found 239.1431. **R*f*** = 0.22 (1:9 DCM/pentane, UV, CAM).

2-(3’,5’-difluorophenyl)-2,4-dimethylpent-4-enal (6d)

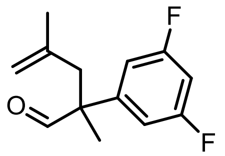

Following **GP2**/**GP3**: 3’,5’-difluoroacetophenone (548 mg, 3.44 mmol) gave the title product **6d** as a colorless oil (422 mg, 1.88 mmol, 55 % over 2 steps). ^{1}**H NMR** (400 MHz, $CDCl_3$) δ 9.52 (s, 1H), 6.86–6.80 (m, 2H), 6.74 (tt, *J* = 8.6, 2.3 Hz, 1H), 4.85–4.83 (m, 1H), 4.63–4.62 (m, 1H), 2.68 (dd, *J* = 14.1, 1.0 Hz, 1H), 2.60 (dd, *J* = 14.0, 1.0 Hz, 1H), 1.45 (s, 3H), 1.45 (s, 3H). ^{13}**C NMR** (101 MHz, $CDCl_3$) δ 200.8, 163.4 (dd, *J* = 249.1, 12.9 Hz), 144.3 (t, *J* = 8.6 Hz), 140.7, 116.2, 110.7 (dd, *J* = 26.3, 7.0 Hz), 103.1 (t, *J* = 25.2 Hz), 53.7 (t, *J* = 1.9 Hz), 44.4, 24.3, 18.9. ^{19}**F NMR** (376 MHz, $CDCl_3$) δ -108.76 (t, *J* = 8.6 Hz). **IR** (ATR, neat) 2974, 2810, 2709, 1726, 1622, 1596, 1433, 1318, 1120, 986, 898, 855, 690. **HRMS** (APCI/QTOF) m/z: $[M+H]^+$ calculated for $C_{13}H_{15}F_2O^+$ 225.1085; found 225.1077. **R*f*** = 0.26 (1:9 DCM/pentane, UV, CAM).

2-(4-methoxyphenyl)-2,4-dimethylpent-4-enal (6e)

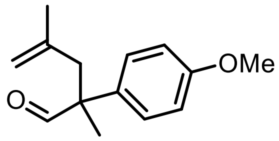

Following **GP2**/**GP3**: 4’-methoxyacetophenone (151 mg, 1.00 mmol) gave the title product **6e** as a colorless oil (160 mg, 0.73 mmol, 73 % over 2 steps). ^{1}**H NMR** (400 MHz, $CDCl_3$) δ 9.48 (s, 1H), 7.20 (d, *J* = 8.8 Hz, 2H), 6.90 (d, *J* = 8.9 Hz, 2H), 4.81–4.79 (m, 1H), 4.62–4.61 (m, 1H), 3.80 (s, 3H), 2.69 (d, *J* = 13.9 Hz, 1H), 2.62 (d, *J* = 13.9 Hz, 1H), 1.44 (s, 3H), 1.41 (s, 3H). ^{13}**C NMR** (101 MHz, $CDCl_3$) δ 202.0, 158.9, 141.8, 131.6, 128.6, 115.4, 114.3, 55.4, 52.9, 44.2, 24.3, 18.7. **IR** (ATR, neat) 2967, 2837, 1719, 1609, 1511, 1460, 1250, 1184, 1032, 896, 828. **HRMS** (ESI/QTOF) m/z: $[M+H]^+$ calculated for $C_{14}H_{19}O_2^+$ 219.1380; found 219.1381. **R*f*** = 0.16 (1:9 DCM/pentane, UV, CAM).

2-(3,5-di-tert-butylphenyl)-2,4-dimethylpent-4-enal (6f)

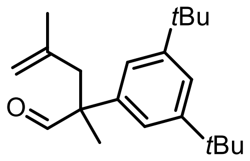

Following **GP2**/**GP3**: 1-(3,5-di-tert-butylphenyl)ethan-1-one (536 mg, 2.24 mmol) gave the title product **6f** as a colorless oil (304 mg, 1.01 mmol, 46 % over 2 steps). ^{1}**H NMR** (400 MHz, $CDCl_3$) δ 9.56 (s, 1H), 7.34 (t, *J* = 1.8 Hz, 1H), 7.10 (d, *J* = 1.7 Hz, 2H), 4.81 (dt, *J* = 3.0, 1.5 Hz, 1H), 4.64 (dq, *J* = 1.7, 0.8 Hz, 1H), 2.73 (dd, *J* = 13.7, 0.9 Hz, 1H), 2.64 (dd, *J* = 13.7, 0.9 Hz, 1H), 1.47 (s, 3H), 1.36 (br s, 3H), 1.32 (s, 18H). ^{13}**C NMR** (101 MHz, $CDCl_3$) δ 202.5, 151.2, 142.0, 138.9, 121.6, 121.3, 115.4, 54.0, 44.5, 35.2, 31.6, 24.1, 18.9. **IR** (ATR, neat) 2965, 1719, 1642, 1446, 1373, 895, 762, 701. **HRMS** (ESI/QTOF) m/z: $[M+H]^+$ calculated for $C_{21}H_{33}O^+$ 301.2526; found 301.2539. **R*f*** = 0.43 (3:7 DCM/pentane, CAM).

2-isopropyl-4-methyl-2-phenylpent-4-enal (6g)

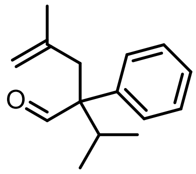

Following **GP2**/**GP3**: 2-methyl-1-phenyl-propane-1-one (150 mg, 0.99 mmol) the title product **6g** as a colorless oil (109 mg, 0.50 mmol, 51 % over 2 steps). **^{1}H NMR** (400 MHz, $CDCl_3$) δ 9.92 (s, 1H), 7.39–7.34 (m, 2H), 7.30–7.26 (m, 1H), 7.24–7.21 (m, 2H), 4.75–4.73 (m, 1H), 4.51 (dq, *J* = 1.9, 1.0 Hz, 1H), 2.73 (s, 2H), 2.44 (hept, *J* = 6.9 Hz, 1H), 1.43 (s, 3H), 0.91 (d, *J* = 7.0 Hz, 3H), 0.83 (d, *J* = 6.8 Hz, 3H). **^{13}C NMR** (101 MHz, $CDCl_3$) δ 205.3, 141.5, 138.1, 128.8, 128.2, 127.0, 115.5, 60.2, 41.5, 33.5, 24.8, 18.6, 17.9. **IR** (ATR, neat) 2965, 1719, 1642, 1446, 1373, 895, 762, 701. **HRMS** (ESI+APCI) m/z: $[M+H]^+$ calculated for $C_{15}H_{21}O^+$ 217.1587; found 217.1576. **R*f*** = 0.38 (1:9 DCM/pentane, CAM).

1-(2-methylallyl)-1,2,3,4-tetrahydronaphthalene-1-carbaldehyde (6h)

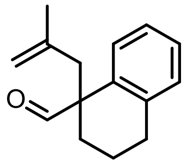

Following **GP2**/**GP3**: tetralone (270 mg, 1.79 mmol) gave the title product **6h** as a colorless oil (224 mg, 1.05 mmol, 58 % over 2 steps). **^{1}H NMR** (400 MHz, $CDCl_3$) δ 9.60 (s, 1H), 7.28–7.26 (m, 1H), 7.22–7.12 (m, 3H), 4.81 (dt, *J* = 3.0, 1.5 Hz, 1H), 4.68 (dt, *J* = 1.9, 1.0 Hz, 1H), 2.78–2.75 (m, 2H), 2.73 (dd, *J* = 14.1, 1.1 Hz, 1H), 2.59 (dd, *J* = 13.9, 1.0 Hz, 1H), 2.13–2.06 (m, 1H), 1.91–1.75 (m, 3H), 1.45 (dd, *J* = 1.5, 0.8 Hz, 3H). **^{13}C NMR** (101 MHz, $CDCl_3$) δ 202.3, 142.0, 138.7, 134.7, 130.0, 128.6, 127.1, 126.4, 115.8, 52.6, 45.1, 30.2, 27.9, 24.2, 19.7. **IR** (ATR, neat) 3072, 2939, 2870, 2713, 1721, 1447, 895, 757, 734. **HRMS** (ESI/QTOF) m/z: $[M+Ag]^+$ calculated for $C_{15}H_{18}AgO^+$ 321.0403; found 321.0405. **R*f*** = 0.21 (1:9 DCM/pentane, UV, CAM).

2-cyclohexyl-2,4-dimethylpent-4-enal (6i)

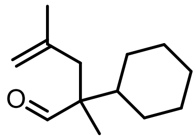

Following **GP2**/**GP3**: 1-cyclohexylethanone (413 mg, 3.11 mmol) gave the title product **6i** as a colorless oil (238 mg, 1.22 mmol, 76 % over 2 steps). **^{1}H NMR** (400 MHz, $CDCl_3$) δ 9.56 (s, 1H), 4.83–4.81 (m, 1H), 4.66–4.66 (m, 1H), 2.44 (d, *J* = 13.8 Hz, 1H), 2.20 (d, *J* = 13.8 Hz, 1H), 1.80–1.73 (m, 3H), 1.70–1.66 (m, 1H), 1.63 (br s, 3H), 1.53–1.49 (m, 2H), 1.26–1.22 (m, 1H), 1.21–1.15 (m, 1H), 1.14–1.09 (m, 1H), 1.07–0.98 (m, 2H), 0.95 (s, 3H). **^{13}C NMR** (101 MHz, $CDCl_3$) δ 208.0, 142.0, 115.2, 52.2, 43.6, 42.7, 28.3, 27.0, 26.9, 26.9, 26.6, 24.6, 14.4. **IR** (ATR, neat) 2926, 2854, 2700, 1723, 1644, 1449, 1396, 1376, 1005, 945, 893, 846, 789. **HRMS** (ESI+APCI) m/z: $[M+H]^+$ calculated for $C_{13}H_{23}O^+$ 195.1743; found 195.1735. **R*f*** = 0.35 (3:7 DCM/pentane, CAM).

2-(adamantan-1-yl)-2,4-dimethylpent-4-enal (6j)

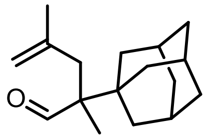

Following **GP2**/**GP3**: 1-adamantyl methyl ketone (471 mg, 2.59 mmol) and two portions of LiHMDS (1.4 equiv., 1 M in THF) separated by 16 h gave the title product **6j** as a white solid (261 mg, 1.06 mmol, 41 % over 2 steps). **^{1}H NMR** (400 MHz, $CDCl_3$) δ 9.83 (s, 1H), 4.78–4.76 (m, 1H), 4.62–4.61 (m, 1H), 2.73 (d, *J* = 13.6 Hz, 1H), 2.15 (d, *J* = 13.5 Hz, 1H), 2.00–1.97 (m, 3H), 1.75–1.67 (m, 6H), 1.64–1.58 (m, 6H), 1.55 (s, 3H), 0.91 (s, 3H). **^{13}C NMR** (101 MHz, $CDCl_3$) δ 209.6, 142.7, 115.0, 53.7, 38.8, 38.3, 37.0, 36.9, 28.8, 24.3, 12.7. **IR** (ATR, neat) 2901, 2849, 2709, 1719, 1644, 1448, 1377, 1311, 1056, 873. **HRMS** (Sicrit plasma/LTQ-Orbitrap) m/z: $[M+H]^+$ calculated for $C_{17}H_{27}O^+$ 247.2056; found 247.2056. **mp** 59.6–62.9 °C. **R*f*** = 0.11 (1:9 DCM/pentane, $KMnO_4$).

2,4-dimethyl-2-(2-(phenylthio)ethyl)pent-4-enal (6k)

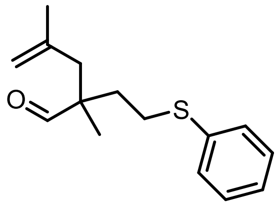

Following **GP2**/**GP3**: 4-(phenylthio)butan-2-one **S-IV** (393 mg, 2.18 mmol) gave the title product **6k** as a colorless oil (243 mg, 0.98 mmol, 48 % over 2 steps), obtained. **^{1}H NMR** (400 MHz, $CDCl_3$) δ 9.53 (s, 1H), 7.33–7.27 (m, 4H), 7.21–7.17 (m, 1H), 4.86–4.84 (m, 1H), 4.68–4.66 (m, 1H), 2.90–2.75 (m, 2H), 2.34 (d, *J* = 14.0 Hz, 1H), 2.19 (d, *J* = 14.1 Hz, 1H), 1.93 (ddd, *J* = 14.1, 11.4, 5.3 Hz, 1H), 1.76 (ddd, *J* = 14.1, 11.5, 5.3 Hz, 1H), 1.65–1.61 (m, 3H), 1.09 (s, 3H). **^{13}C NMR** (101 MHz, $CDCl_3$) δ 205.6, 141.0, 136.0, 129.5, 129.1, 126.3, 115.8, 49.5, 44.5, 35.7, 28.7, 24.4, 18.3. **IR** (ATR, neat) 3075, 2969, 2931, 1724, 1584, 1481, 1439, 898, 739, 691. **HRMS** (APCI/QTOF) m/z: $[M+H]^+$ calculated for $C_{15}H_{21}OS^+$ 249.1308; found 249.1312. **R*f*** = 0.54 (1:1 DCM/pentane, UV, CAM).

4-methyl-2-phenyl-2-(trifluoromethyl)pent-4-enal (6l)

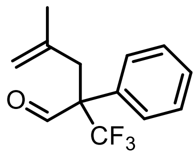

Following **GP2**/**GP3**: 2,2,2-trifluoroacetophenone (342 mg, 1.94 mmol) gave the title product **6l** as a colorless oil (272 mg, 1.12 mmol, 58 % over 2 steps). **^{1}H NMR** (400 MHz, $CDCl_3$) δ 9.79 (q, *J* = 2.4 Hz, 1H), 7.46–7.36 (m, 5H), 4.87–4.86 (m, 1H), 4.68–4.67 (m, 1H), 3.04 (s, 2H), 1.53 (br s, 3H). **^{13}C NMR** (101 MHz, $CDCl_3$) δ 195.0 (q, *J* = 1.7 Hz), 138.8, 131.5, 129.1, 128.9 (q, *J* = 1.2 Hz), 125.4 (q, *J* = 284.9 Hz), 117.4, 62.5 (q, *J* = 22.6 Hz), 38.2 (d, *J* = 1.9 Hz), 24.2. **^{19}F NMR** (376 MHz, $CDCl_3$) δ -67.25. **IR** (ATR, neat) 2854, 1737, 1450, 1213, 1151, 904, 698. **HRMS** (APCI/QTOF) m/z: $[M+H]^+$ calculated for $C_{13}H_{14}F_3O^+$ 243.0991; found 243.0999. **R*f*** = 0.33 (1:9 DCM/pentane, UV, CAM).

Diastereomeric carbene precursors synthesis

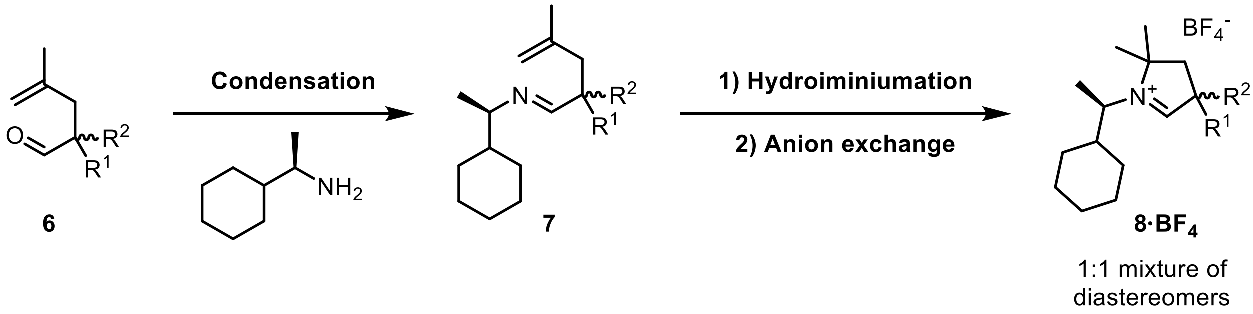

General Procedure (GP4)

Condensation

(R)-(–)-1-cyclohexylethylamine (1.0 equiv.) and aldehyde **6** (1.0 equiv.) were stirred over activated 4 Å molecular sieves in dry toluene (0.8 M) at 100 °C for 16 h. The reaction mixture was filtered over Celite and the filtrate was concentrated under reduced pressure to afford the corresponding crude aldimine **7** used for the next step without further purification.

Hydroiminiumation

A solution of crude aldimine **7** (1.0 equiv.) in toluene (0.3 M) was cooled down to -78 °C. HCl (2 M in Et_2O, 1.0 equiv.) was added dropwise and the mixture was stirred at this temperature for 30 min. The mixture was then left to reach 23 °C and stirred for an additional 30 min. The mixture was then stirred for 16 h at 80 °C. Eventually, the mixture was heated to 110 °C for 3 h. Volatiles were removed under reduced pressure and the residue was triturated in Et_2O and filtered to afford the cyclic iminium chloride **8**·Cl as an off-white/beige solid.

Anion exchange

DCM (0.4 M) was added to the iminium chloride (1.0 equiv.) and $NaBF_4$ (2.5 equiv.). The mixture was stirred at 23 °C for 5 h. The reaction mixture was directly purified by column chromatography (conditioning: DCM, eluent: DCM to 5:95 MeOH/DCM) to afford a 1:1 mixture of diastereomeric tetrafluoroborate CAAC precursor **8**·BF_4.

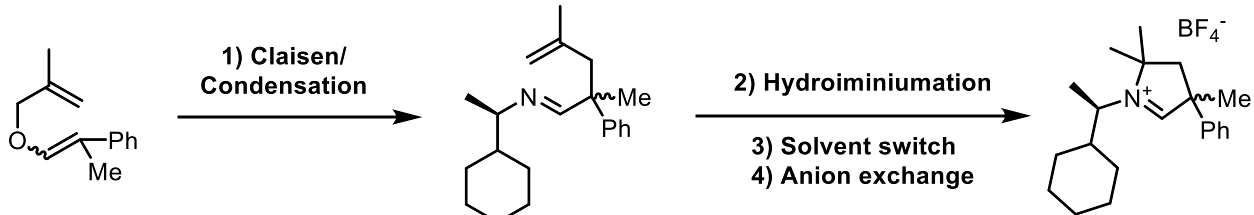

5a **7a** **8a·BF4**

One-Pot Procedure

(R)-(–)-1-cyclohexylethylamine (292 mg, 2.25 mmol, 1.0 equiv.) and allyl vinyl ether **5a** (429 mg, 2.28 mmol, 1.0 equiv.) were stirred in dry *p*-xylene (2.8 mL, 0.8 M) at 150 °C for 16 h in a flask mounted with a Dean-Stark apparatus containing activated 4Å molecular sieves in the water collector.

The reaction mixture was diluted with dry toluene (2.8 mL, 0.4 M) and cooled to -78 °C. HCl (1.2 mL, 2 M in Et_2O, 1.0 equiv.) was added and the mixture stirred at -78 °C for 30 min. The mixture was warmed to 23 °C and stirred for another 30 min whereupon it was heated at 80 °C for 16 h. Finally, the mixture was heated to 110 °C for 3h.

The mixture was concentrated under reduced pressure. $NaBF_4$ (645 mg, 5.75 mmol, 2.5 equiv.) was added and the solvent was exchanged for DCM (0.4 M). The mixture was stirred for 5 h at 23 °C.

The reaction mixture was directly purified by column chromatography (conditioning: DCM, eluent: DCM to 5:95 MeOH/DCM) to afford a mixture of diastereomeric tetrafluoroborate CAAC precursor **8a**·BF_4.

(*R*)-[Me, Cy]$^{Me, Ph}$CAAC(H^+)(BF_4^-) (8a)

IUPAC: 1-((*R*)-1-cyclohexylethyl)-2,2,4-trimethyl-4-phenyl-3,4-dihydro-2H-pyrrol-1-ium tetrafluoroborate

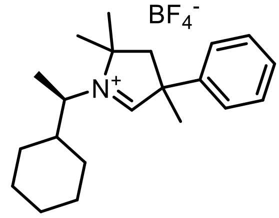

Following **GP4**: 2,4-dimethyl-2-phenylpent-4-enal **6a** (320 mg, 1.70 mmol) and (*R*)-(–)-1-cyclohexylethylamine (218 mg, 1.68 mmol) afforded **8a** (347 mg, 901 µmol, 54 % over 3 steps) as an off-white solid in a 1:1 mixture of diastereomers. **^{1}H NMR** (400 MHz, CD_2Cl_2) δ 9.12 (s, 1H), 9.12 (s, 1H), 7.49–7.44 (m, 4H), 7.40–7.35 (m, 2H), 7.33–7.28 (m, 4H), 3.66–3.55 (m, 2H), 2.80 (d, *J* = 13.9 Hz, 1H), 2.76 (d, *J* = 13.9 Hz, 1H), 2.47 (d, *J* = 13.9 Hz, 1H), 2.45 (d, *J* = 13.9 Hz, 1H), 2.08–2.00 (m, 1H), 1.99–1.83 (m, 5H), 1.82 (s, 3H), 1.82 (s, 3H), 1.81–1.70 (m, 4H), 1.68 (s, 3H), 1.67–1.65 (m, 1H), 1.63 (s, 3H), 1.61 (d, *J* = 6.8 Hz, 3H), 1.57 (d, *J* = 6.7 Hz, 3H), 1.47 (s, 3H), 1.43 (s, 3H), 1.41–1.28 (m, 4H), 1.24–1.10 (m, 3H), 1.08–0.97 (m, 3H), 0.94–0.84 (m, 1H). **^{13}C NMR** (101 MHz, CD_2Cl_2) δ 182.3, 182.0, 141.5, 141.3, 130.2, 130.2, 130.1, 130.1, 128.8, 128.8, 128.8, 128.8, 125.9, 125.8, 79.5, 79.2, 62.6, 62.6, 54.7, 54.5, 50.0, 49.4, 43.3, 42.8, 31.0, 31.0, 29.4, 29.2, 28.3, 27.9, 27.8, 27.7, 27.3, 26.7, 26.1, 26.0, 25.9, 25.9, 25.8, 25.8, 21.7, 20.8. **^{19}F NMR** (CD_2Cl_2, 376 MHz) δ -150.63 (^1J ^{19}F-^{10}B), -150.68 (^1J ^{19}F-^{11}B). **IR** (ATR, neat) 2931, 2856, 1661, 1449, 1381, 1054, 764, 702. **HRMS** (ESI+APCI) m/z: [M-BF_4]$^+$ calculated for $C_{21}H_{32}N^+$ 298.2529; found 298.2545. **R$_f$** = 0.51 (1:9 MeOH/DCM, UV, CAM).

(*R*)-[Me, Cy]$^{Me, 1-Np}$CAAC(H^+)(BF_4^-) (8b)

IUPAC: 1-((*R*)-1-cyclohexylethyl)-2,2,4-trimethyl-4-(naphthalen-1-yl)-3,4-dihydro-2H-pyrrol-1-ium tetrafluoroborate

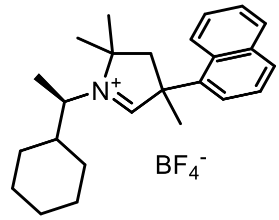

Following **GP4**: 2,4-dimethyl-2-(naphthalen-1-yl)pent-4-enal **6b** (154 mg, 644 µmol) and (*R*)-(–)-1-cyclohexylethylamine (82.4 mg, 634 µmol) afforded **8b** (206 mg, 473 µmol, 75 % over 3 steps) as a beige solid in a 1:1 mixture of diastereomers. **^{1}H NMR** (400 MHz, CD_2Cl_2) δ 9.54 (s, 1H), 9.50 (s, 1H), 7.96–7.91 (m, 4H), 7.87–7.85 (m, 2H), 7.63–7.55 (m, 4H), 7.45–7.40 (m, 2H), 7.23–7.20 (m, 2H), 3.75–3.64 (m, 2H), 3.05 (d, *J* = 13.6 Hz, 1H), 2.99 (d, *J* = 13.8 Hz, 1H), 2.92 (d, *J* = 13.7 Hz, 1H), 2.82 (d, *J* = 13.6 Hz, 1H), 2.19–2.10 (m, 2H), 2.05 (s, 3H), 2.04 (s, 3H), 2.03–1.96 (m, 2H), 1.91–1.83 (m, 3H), 1.80–1.74 (m, 3H), 1.72 (s, 3H), 1.71 (d, *J* = 6.7 Hz, 3H), 1.68 (d, *J* = 6.8 Hz, 3H), 1.66 (s, 3H), 1.65–1.60 (m, 2H), 1.45–1.39 (m, 3H), 1.36 (s, 6H), 1.25–1.08 (m, 5H), 1.04–0.93 (m, 2H). **^{13}C NMR** (101 MHz, CD_2Cl_2) δ 183.5, 182.9, 138.6, 138.3, 135.8, 135.8, 130.6, 130.5, 130.0, 129.9, 129.4, 129.3, 127.0, 126.9, 126.5, 126.5, 125.7, 125.5, 124.6, 124.5, 123.2, 122.9, 80.1, 79.8, 63.1, 62.7, 55.0, 54.6, 50.6, 50.3, 43.5, 42.8, 31.5, 31.2, 29.5, 29.4, 28.5, 28.3, 27.4, 27.3, 27.2, 26.9, 26.1, 26.1, 25.9, 25.9, 25.8, 21.6, 21.0. (1C not resolved). **^{19}F NMR** (CD_2Cl_2, 376 MHz) δ -150.45 (^1J ^{19}F-^{10}B), -150.51 (^1J ^{19}F-^{11}B). **IR** (ATR, neat) 2932, 2856, 1664, 1451, 1388, 1057, 805, 779. **HRMS** (Nanochip-based ESI/LTQ-Orbitrap) m/z: [M-BF_4]$^+$ calculated for $C_{25}H_{34}N^+$ 348.2686; found 348.2680. **R$_f$** = 0.41 (1:9 MeOH/DCM, UV, CAM).

(*R*)-[Me, Cy]$^{Me, 2\text{-}Np}$CAAC(H^+)(BF_4^-) (8c)

IUPAC: 1-((*R*)-1-cyclohexylethyl)-2,2,4-trimethyl-4-(naphthalen-2-yl)-3,4-dihydro-2H-pyrrol-1-ium tetrafluoroborate

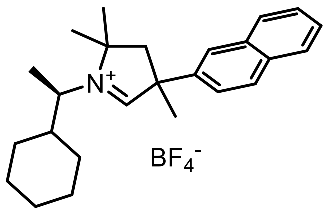

Following **GP4**: 2,4-dimethyl-2-(naphthalen-2-yl)pent-4-enal **6c** (491 mg, 2.06 mmol) and (*R*)-(–)-1-cyclohexylethylamine (267 mg, 2.06 mmol) afforded **8c** (456 mg, 1.05 mmol, 51 % over 3 steps) as a beige solid in a 1:1 mixture of diastereomers. **^{1}H NMR** (400 MHz, CD_2Cl_2) δ 9.31 (s, 1H), 9.31 (s, 1H), 7.97–7.95 (m, 2H), 7.95–7.92 (m, 1H), 7.89–7.83 (m, 3H), 7.82–7.77 (m, 2H), 7.57–7.52 (m, 4H), 7.43–7.38 (m, 2H), 3.67–3.57 (m, 2H), 2.92 (d, *J* = 13.8 Hz, 1H), 2.89 (d, *J* = 13.9 Hz, 1H), 2.53 (d, *J* = 13.9 Hz, 1H), 2.51 (d, *J* = 13.9 Hz, 1H), 2.13–2.05 (m, 1H), 2.03–1.92 (m, 3H), 1.89 (s, 3H), 1.89 (s, 3H), 1.87–1.72 (m, 5H), 1.70 (s, 3H), 1.67–1.64 (m, 1H), 1.65 (s, 3H), 1.64 (d, *J* = 6.8 Hz, 3H), 1.61–1.58 (m, 1H), 1.59 (d, *J* = 6.6 Hz, 3H), 1.48–1.46 (m, 1H), 1.47 (s, 3H), 1.44–1.38 (m, 2H), 1.41 (s, 3H), 1.32–1.26 (m, 1H), 1.20–1.15 (m, 1H), 1.14–0.96 (m, 5H), 0.92–0.82 (m, 1H). **^{13}C NMR** (101 MHz, CD_2Cl_2) δ 182.4, 138.7, 138.5, 133.8, 133.7, 133.1, 130.3, 130.3, 128.7, 128.5, 128.0, 127.9, 127.5, 127.5, 127.4, 127.4, 124.9, 124.8, 123.4, 123.3, 79.5, 79.3, 62.7, 62.7, 54.8, 54.7, 49.6, 49.4, 43.4, 42.8, 31.2, 31.1, 29.5, 29.3, 28.3, 28.2, 28.0, 27.8, 27.4, 26.6, 26.1, 26.0, 25.9, 25.9, 25.9, 25.8, 21.6, 20.9. (2C not resolved). **^{19}F NMR** (CD_2Cl_2, 376 MHz) δ -149.49 (^{1}J ^{19}F-^{10}B), -149.54 (^{1}J ^{19}F-^{11}B). **IR** (ATR, neat) 2931, 2856, 1661, 1452, 1052, 820, 752. **HRMS** (ESI/QTOF) m/z: $[M\text{-}BF_4]^+$ calculated for $C_{25}H_{34}N^+$ 348.2686; found 348.2680. **R*f*** = 0.43 (1:9 MeOH/DCM, UV, CAM).

(*R*)-[Me, Cy]$^{Me, 3,5\text{-}diFPh}$CAAC(H^+)(BF_4^-) (8d)

IUPAC: 1-((*R*)-1-cyclohexylethyl)-4-(3,5-difluorophenyl)-2,2,4-trimethyl-3,4-dihydro-2H-pyrrol-1-ium tetrafluoroborate

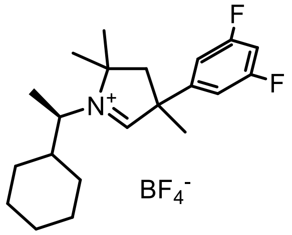

Following **GP4**: 2-(3,5-difluorophenyl)-2,4-dimethylpent-4-enal **6d** (422 mg, 1.88 mmol) and (*R*)-(–)-1-cyclohexylethylamine (249 mg, 1.92 mmol) afforded **8d** (438 mg, 1.04 mmol, 55 % over 3 steps) as a beige solid in a 1:1 mixture of diastereomers. **^{1}H NMR** (400 MHz, CD_2Cl_2) δ 9.25 (s, 1H), 9.22 (s, 1H), 6.96–6.89 (m, 4H), 6.87–6.81 (m, 2H), 3.66–3.58 (m, 2H), 2.72 (d, *J* = 14.0 Hz, 1H), 2.70 (d, *J* = 14.0 Hz, 1H), 2.50 (d, *J* = 14.0 Hz, 1H), 2.47 (d, *J* = 14.0 Hz, 1H), 2.12–2.02 (m, 1H), 1.97–1.91 (m, 2H), 1.90–1.82 (m, 2H), 1.80 (s, 6H), 1.76–1.70 (m, 3H), 1.69 (s, 3H), 1.63 (s, 3H), 1.62 (d, *J* = 6.8 Hz, 3H), 1.57 (d, *J* = 6.7 Hz, 3H), 1.49 (s, 3H), 1.46 (s, 3H), 1.42–1.36 (m, 3H), 1.34–1.10 (m, 6H), 1.08–0.89 (m, 5H). **^{13}C NMR** (101 MHz, CD_2Cl_2) δ 181.4, 181.1, 164.0 (dd, *J* = 250.7, 13.0 Hz), 164.0 (dd, *J* = 250.4, 13.0 Hz), 145.1 (t, *J* = 8.8 Hz), 145.1 (t, *J* = 8.9 Hz), 109.5 (m), 104.3 (t, *J* = 25.3 Hz), 79.5, 79.3, 63.0, 63.0, 54.7 (t, *J* = 2.0 Hz), 54.5 (t, *J* = 2.1 Hz), 49.5, 49.1, 43.2, 42.7, 31.2, 31.1, 29.5, 29.3, 28.1, 28.1, 28.0, 28.0, 27.1, 27.0, 26.1, 26.0, 25.9, 25.8, 21.7, 20.8. (4C not resolved). **^{19}F NMR** (CD_2Cl_2, 376 MHz) δ -107.03 (C-F), -107.08 (C-F), -150.07 (^{1}J ^{19}F-^{10}B), -150.13 (^{1}J ^{19}F-^{11}B). **IR** (ATR, neat) 2933, 2857, 1624, 1598, 1450, 1321, 1123, 1056, 854. **HRMS** (ESI/QTOF) m/z: $[M\text{-}BF_4]^+$ calculated for $C_{21}H_{30}F_2N^+$ 334.2341; found 334.2340. **R*f*** = 0.54 (1:9 MeOH/DCM, UV, faint stain CAM).

(R)-[Me, Cy]$^{Me, (4-OMe)Ph}$CAAC(H$^+$)(BF$_4^-$) (8e)

IUPAC: 1-((*R*)-1-cyclohexylethyl)-4-(4-methoxyphenyl)-2,2,4-trimethyl-3,4-dihydro-2H-pyrrol-1-ium tetrafluoroborate

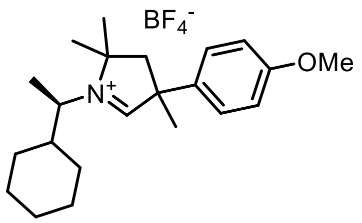

Following **GP4**: 2-(4-methoxyphenyl)-2,4-dimethylpent-4-enal **6e** (158 mg, 722 µmol) and (*R*)-(–)-1-cyclohexylethylamine (94.1 mg, 725 µmol) afforded **8e** (132 mg, 319 µmol, 44 % over 3 steps) as a beige solid in a 1:1 mixture of diastereomers. **^{1}H NMR** (400 MHz, CD_2Cl_2) δ 9.03 (s, 2H), 7.26–7.20 (m, 4H), 6.99–6.95 (m, 4H), 3.80 (s, 6H), 3.64–3.53 (m, 2H), 2.77 (d, J = 14.0 Hz, 1H), 2.73 (d, J = 13.9 Hz, 1H), 2.42 (d, J = 13.9 Hz, 1H), 2.40 (d, J = 13.9 Hz, 1H), 2.05–1.98 (m, 1H), 1.96–1.80 (m, 6H), 1.78 (s, 3H), 1.78 (s, 3H), 1.77–1.69 (m, 3H), 1.67 (s, 3H), 1.62 (s, 3H), 1.59 (d, J = 6.7 Hz, 3H), 1.54 (d, J = 6.7 Hz, 3H), 1.48 (s, 3H), 1.44 (s, 3H), 1.41–1.30 (m, 4H), 1.29–1.24 (m, 1H), 1.22–1.14 (m, 2H), 1.12–0.93 (m, 4H), 0.92–0.82 (m, 1H). **^{13}C NMR** (101 MHz, CD_2Cl_2) δ 182.1, 181.9, 160.0, 132.7, 132.6, 127.2, 127.2, 115.4, 115.4, 79.4, 79.1, 62.4, 55.8, 54.0, 49.9, 49.3, 43.2, 42.8, 31.0, 31.0, 29.4, 29.2, 28.4, 27.7, 27.7, 27.4, 26.6, 26.1, 26.0, 25.9, 25.9, 25.8, 21.6, 20.8. (6 C not resolved). **^{19}F NMR** (CD_2Cl_2, 376 MHz) δ -150.80 (^1J ^{19}F-^{10}B), -150.85 (^1J ^{19}F-^{11}B). **IR** (ATR, neat) 2932, 2856, 1660, 1609, 1514, 1454, 1256, 1188, 1052, 1029, 832. **HRMS** (ESI+APCI) m/z: [M-BF$_4$]$^+$ calculated for $C_{22}H_{34}NO^+$ 328.2635; found 328.2637. **R$_f$** = 0.53 (1:9 MeOH/DCM, UV, CAM).

(R)-[Me, Cy]$^{Me, 3,5-ditBuPh}$CAAC(H$^+$)(BF$_4^-$) (8f)

IUPAC: 1-((*R*)-1-cyclohexylethyl)-4-(3,5-di-tert-butylphenyl)-2,2,4-trimethyl-3,4-dihydro-2H-pyrrol-1-ium tetrafluoroborate

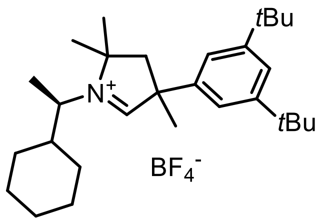

Following **GP4**: 2-(3,5-di-tert-butylphenyl)-2,4-dimethylpent-4-enal **6f** (304 mg, 1.01 mmol) and (*R*)-(–)-1-cyclohexylethylamine (133 mg, 1.02 mmol) afforded **8f** (287 mg, 577 µmol, 57 % over 3 steps) as a beige solid in a 1:1 mixture of diastereomers. **^{1}H NMR** (400 MHz, CD_2Cl_2) δ 9.07 (s, 1H), 9.05 (s, 1H), 7.43–7.42 (m, 2H), 7.10 (d, *J* = 1.7 Hz, 2H), 7.04 (d, *J* = 1.7 Hz, 2H), 3.69–3.57 (m, 2H), 2.77 (d, *J* = 13.9 Hz, 1H), 2.73 (d, *J* = 13.8 Hz, 1H), 2.47 (d, *J* = 14.0 Hz, 1H), 2.43 (d, *J* = 13.8 Hz, 1H), 2.08–2.01 (m, 1H), 1.99–1.90 (m, 3H), 1.88–1.83 (m, 2H), 1.83 (s, 3H), 1.82 (s, 3H), 1.80–1.76 (m, 1H), 1.75–1.66 (m, 3H), 1.69 (s, 3H), 1.63–1.57 (m, 1H), 1.63 (s, 3H), 1.61 (d, *J* = 6.7 Hz, 3H), 1.58 (d, *J* = 6.7 Hz, 3H), 1.48–1.44 (m, 1H), 1.46 (s, 3H), 1.45 (s, 3H), 1.42–1.34 (m, 3H), 1.32 (s, 36H), 1.25–1.18 (m, 2H), 1.16–1.11 (m, 1H), 1.10–0.98 (m, 3H), 0.96–0.86 (m, 1H). **^{13}C NMR** (101 MHz, CD_2Cl_2) δ 182.6, 182.0, 152.9, 152.9, 140.8, 140.4, 123.0, 120.1, 119.9, 79.5, 79.2, 62.5, 62.4, 55.1, 54.7, 50.6, 50.0, 43.1, 42.8, 35.5, 35.5, 31.5, 31.5, 31.0, 30.8, 29.5, 29.3, 28.3, 27.5, 27.5, 27.1, 26.8, 26.2, 26.1, 26.0, 26.0, 25.9, 25.8, 21.9, 20.9. (2C not resolved). **^{19}F NMR** (CD_2Cl_2, 376 MHz) δ -149.56 (^1J ^{19}F-^{10}B), -149.61 (^1J ^{19}F-^{11}B).). **IR** (ATR, neat) 2933, 2860, 1660, 1597, 1452, 1395, 1249, 1055, 712. **HRMS** (Nanochip-based ESI/LTQ-Orbitrap) m/z: [M-BF$_4$]$^+$ calculated for $C_{29}H_{48}N^+$ 410.3781; found 410.3779. **R$_f$** = 0.55 (1:9 MeOH/DCM, UV, CAM)–dia1. **R$_f$** = 0.50 (1:9 MeOH/DCM, UV, CAM)–dia2.

***(R)*-[Me, Cy]$^{iPr, Ph}$CAAC(H$^+$)(BF$_4^-$) (8g)**

IUPAC: 1-((*R*)-1-cyclohexylethyl)-4-isopropyl-2,2-dimethyl-4-phenyl-3,4-dihydro-2H-pyrrol-1-ium tetrafluoroborate

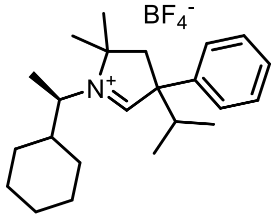

Following **GP4**: 2-isopropyl-4-methyl-2-phenylpent-4-enal **6g** (438 mg, 2.03 mmol) and (*R*)-(–)-1-cyclohexylethylamine (269 mg, 2.07 mmol) afforded **8g** (527 mg, 1.28 mmol, 63 % over 3 steps) as a beige solid in a 1:1 mixture of diastereomers. **^{1}H NMR** (400 MHz, CD_2Cl_2) δ 9.42 (s, 1H), 9.38 (s, 1H), 7.50–7.33 (m, 10H), 3.55 (dq, *J* = 9.3, 6.7 Hz, 1H), 3.40 (dq, *J* = 10.1, 6.6 Hz, 1H), 2.83 (d, *J* = 14.2 Hz, 1H), 2.81 (d, *J* = 14.2 Hz, 1H), 2.54 (hept, *J* = 6.7 Hz, 1H), 2.47 (hept, *J* = 6.8 Hz, 1H), 2.35 (d, *J* = 14.2 Hz, 1H), 2.35 (d, *J* = 14.2 Hz, 1H), 2.07–2.04 (m, 2H), 1.96–1.92 (m, 2H), 1.87–1.69 (m, 4H), 1.65 (s, 3H), 1.62 (s, 3H), 1.58 (d, *J* = 6.7 Hz, 3H), 1.54–1.46 (m, 2H), 1.43 (d, *J* = 6.7 Hz, 3H), 1.40–1.31 (m, 3H), 1.25 (s, 3H), 1.24 (s, 3H), 1.22–1.12 (m, 2H), 1.10–1.02 (m, 3H), 1.00 (d, *J* = 6.8 Hz, 3H), 0.99 (d, *J* = 6.8 Hz, 3H), 0.97–0.87 (m, 2H), 0.83 (d, *J* = 6.9 Hz, 3H), 0.79 (d, *J* = 6.8 Hz, 3H), 0.75–0.56 (m, 2H). **^{13}C NMR** (101 MHz, CD_2Cl_2) δ 183.0, 182.8, 138.7, 138.5, 130.0, 130.0, 128.8, 128.8, 127.0, 78.6, 78.1, 63.0, 62.9, 62.7, 62.6, 43.1, 42.8, 40.8, 40.0, 37.8, 37.6, 31.6, 30.6, 29.9, 29.3, 27.7, 26.9, 26.7, 26.1, 26.0, 25.8, 25.8, 25.7, 25.4, 21.3, 21.2, 18.9, 18.6, 17.5, 17.2. (2C not resolved). **^{19}F NMR** (CD_2Cl_2, 376 MHz) δ -150.70 (^{1}J ^{19}F-^{10}B), -150.76 (^{1}J ^{19}F-^{11}B). **IR** (ATR, neat) 3031, 2935, 2858, 1660, 1467, 1449, 1216, 1050, 1033, 747, 701. **HRMS** (ESI+APCI) m/z: [M-BF_4]$^+$ calculated for $C_{23}H_{36}N^+$ Exact Mass: 326.2842; found Exact Mass: 326.2844. **R$_f$** = 0.41 (1:9 MeOH/DCM, UV, CAM).

***(R)*-[Me, Cy]^{THN}CAAC(H$^+$)(BF$_4^-$) (8h)**

IUPAC: 1'-((*R*)-1-cyclohexylethyl)-5',5'-dimethyl-3,4,4',5'-tetrahydro-2H-spiro[naphthalene-1,3'-pyrrol]-1'-ium tetrafluoroborate

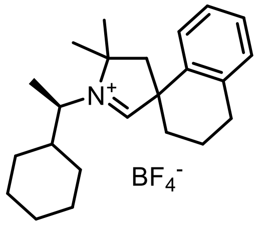

Following **GP4**: 1-(2-methylallyl)-1,2,3,4-tetrahydronaphthalene-1-carbaldehyde **6h** (215 mg, 1.00 mmol) and (*R*)-(–)-1-cyclohexylethylamine (137 mg, 1.03 mmol) afforded **8h** (289 mg, 703 µmol, 70 % over 3 steps) as an off-white solid in a 1:1 mixture of diastereomers. **^{1}H NMR** (400 MHz, CD_2Cl_2) δ 8.52 (s, 1H), 8.50 (s, 1H), 7.33–7.23 (m, 6H), 6.74–6.67 (m, 2H), 3.79–3.73 (m, 1H), 3.72–3.65 (m, 1H), 2.91–2.88 (m, 4H), 2.66 (d, *J* = 14.2 Hz, 1H), 2.65 (d, *J* = 14.1 Hz, 1H), 2.41 (dd, *J* = 14.2, 1.1 Hz, 1H), 2.40 (dd, *J* = 14.2, 1.1 Hz, 1H), 2.32–2.21 (m, 2H), 2.17–2.09 (m, 2H), 1.97–1.90 (m, 4H), 1.90–1.80 (m, 8H), 1.79–1.73 (m, 2H), 1.73 (s, 3H), 1.72–1.69 (m, 1H), 1.70 (s, 3H), 1.69 (s, 3H), 1.65 (s, 3H), 1.64 (d, *J* = 6.6 Hz, 3H), 1.58 (d, *J* = 6.8 Hz, 3H), 1.57–1.53 (m, 1H), 1.38–1.27 (m, 4H), 1.22–1.06 (m, 5H), 0.99 (qd, *J* = 12.4, 3.6 Hz, 1H). **^{13}C NMR** (101 MHz, CD_2Cl_2) δ 180.6, 180.5, 137.8, 137.3, 134.7, 134.6, 131.1, 131.1, 129.1, 129.0, 128.0, 127.9, 127.8, 127.6, 79.3, 62.5, 62.4, 55.2, 54.9, 52.0, 51.8, 43.5, 43.0, 33.4, 33.2, 31.4, 30.7, 29.6, 29.3, 29.1, 28.8, 28.8, 28.7, 28.2, 27.9, 26.1, 26.0, 26.0, 26.0, 25.9, 25.8, 22.0, 20.9, 19.2, 19.1. (1C not resolved). **^{19}F NMR** (CD_2Cl_2, 376 MHz) δ -151.38 (^{1}J ^{19}F-^{10}B), -151.43 (^{1}J ^{19}F-^{11}B). **IR** (ATR, neat) 2933, 2857, 1556, 1460, 1050, 1033, 745. **HRMS** (ESI/QTOF) m/z: [M-BF_4]$^+$ calculated for $C_{23}H_{34}N^+$ 324.2686; found 324.2693. **R$_f$** = 0.42 (1:9 MeOH/DCM, UV, CAM).

***(R)*-[Me, Cy]$^{Me, Cy}$CAAC(H$^+$)(BF$_4^-$) (8i)**

IUPAC: 4-cyclohexyl-1-((*R*)-1-cyclohexylethyl)-2,2,4-trimethyl-3,4-dihydro-2H-pyrrol-1-ium tetrafluoroborate

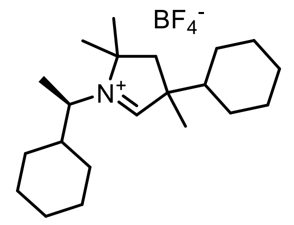

Following **GP4**: 2-cyclohexyl-2,4-dimethylpent-4-enal **6i** (238 mg, 1.22 mmol) and (*R*)-(–)-1-cyclohexylethylamine (160 mg, 1.23 mmol) afforded **8i** (267 mg, 683 µmol, 56 % over 3 steps) as an off-white solid in a 1:1 mixture of diastereomers. **^{1}H NMR** (400 MHz, CD_2Cl_2) δ 8.80 (s, 1H), 8.76 (s, 1H), 3.53 (dq, *J* = 9.0, 6.8 Hz, 1H), 3.45 (dq, *J* = 9.6, 6.7 Hz, 1H), 2.28 (d, *J* = 13.9 Hz, 1H), 2.23 (d, *J* = 13.9 Hz, 1H), 1.95–1.90 (m, 2H), 1.91 (d, *J* = 13.9 Hz, 1H), 1.89 (d, *J* = 13.9 Hz, 1H), 1.89–1.85 (m, 2H), 1.85–1.76 (m, 10H), 1.75–1.66 (m, 6H), 1.58 (s, 6H), 1.56–1.52 (m, 1H), 1.54 (s, 3H), 1.53 (s, 3H), 1.51 (d, *J* = 6.8 Hz, 3H), 1.49 (d, *J* = 6.7 Hz, 3H), 1.50–1.45 (m, 1H), 1.41 (s, 3H), 1.41 (s, 3H), 1.38–1.31 (m, 7H), 1.29–1.24 (m, 2H), 1.23–1.08 (m, 7H), 1.05–0.84 (m, 6H). **^{13}C NMR** (101 MHz, CD_2Cl_2) δ 185.5, 185.3, 78.8, 78.7, 62.2, 62.1, 55.2, 54.9, 45.0, 44.5, 44.0, 43.8, 43.4, 42.6, 31.4, 30.8, 30.0, 30.0, 29.7, 29.2, 28.8, 28.3, 28.0, 27.7, 27.5, 27.2, 26.5, 26.4, 26.4, 26.4, 26.4, 26.3, 26.1, 25.9, 25.9, 25.9, 25.8, 23.6, 22.9, 21.7, 21.0. (1C not resolved). **^{19}F NMR** (CD_2Cl_2, 376 MHz) δ -151.03 (^{1}J ^{19}F-^{10}B), -151.08 (^{1}J ^{19}F-^{11}B). **IR** (ATR, neat) 2928, 2855, 1662, 1451, 1382, 1050, 710. **HRMS** (ESI+APCI) m/z: [M-BF$_4$]$^+$ calculated for $C_{21}H_{38}N^+$ 304.2999; found 304.2996. **R$_f$** = 0.54 (1:9 MeOH/DCM, UV, CAM).

***(R)*-[Me, Cy]$^{Me, Ad}$CAAC(H$^+$)(BF$_4^-$) (8j)**

IUPAC: 4-(adamantan-1-yl)-1-((*R*)-1-cyclohexylethyl)-2,2,4-trimethyl-3,4-dihydro-2H-pyrrol-1-ium tetrafluoroborate

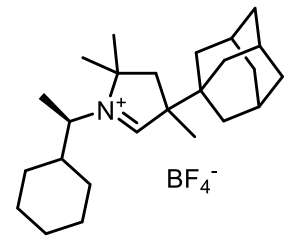

Following **GP4**: 2-(adamantan-1-yl)-2,4-dimethylpent-4-enal **6j** (228 mg, 924 µmol) and (*R*)-(–)-1-cyclohexylethylamine (120 mg, 926 µmol) afforded **8j** (98.0 mg, 221 µmol, 24 % over 3 steps) as a beige solid in a 1:1 mixture of diastereomers. **^{1}H NMR** (400 MHz, CD_2Cl_2) δ 8.84 (s, 1H), 8.78 (s, 1H), 3.55 (dq, *J* = 8.7, 6.8 Hz, 1H), 3.46 (dq, *J* = 9.9, 6.7 Hz, 1H), 2.43 (d, *J* = 14.0 Hz, 1H), 2.39 (d, *J* = 13.9 Hz, 1H), 2.09–2.05 (m, 6H), 2.03–1.87 (m, 5H), 1.85–1.81 (m, 2H), 1.83 (d, *J* = 13.9 Hz, 1H), 1.83 (d, *J* = 13.9 Hz, 1H), 1.80–1.76 (m, 7H), 1.75–1.72 (m, 5H), 1.71–1.67 (m, 7H), 1.66–1.62 (m, 3H), 1.60 (s, 3H), 1.60 (s, 3H), 1.56 (s, 3H), 1.55 (s, 3H), 1.52 (d, *J* = 6.8 Hz, 3H), 1.52 (d, *J* = 6.7 Hz, 3H), 1.48–1.43 (m, 6H), 1.41–1.37 (m, 2H), 1.36 (s, 3H), 1.35 (s, 3H), 1.31–1.22 (m, 3H), 1.19–1.11 (m, 2H), 1.10–1.05 (m, 1H), 1.01–0.94 (m, 2H), 0.94–0.84 (m, 1H). **^{13}C NMR** (101 MHz, CD_2Cl_2) δ 184.5, 184.4, 78.5, 78.4, 62.6, 62.4, 58.8, 58.3, 43.4, 43.2, 42.9, 42.5, 38.5, 38.5, 38.1, 37.7, 37.5, 37.3, 36.8, 36.7, 31.6, 30.8, 29.9, 29.2, 28.9, 28.8, 28.4, 28.0, 27.7, 26.1, 26.1, 25.9, 25.9, 25.9, 25.7, 21.9, 21.0, 20.1, 19.8. (1C not resolved). **^{19}F NMR** (CD_2Cl_2, 376 MHz) δ -150.91 (^{1}J ^{19}F-^{10}B), -150.96 (^{1}J ^{19}F-^{11}B). **IR** (ATR, neat) 2904, 2852, 1658, 1451, 1383, 1057. **HRMS** (ESI/QTOF) m/z: [M-BF$_4$]$^+$ calculated for $C_{25}H_{42}N^+$ 356.3312; found 356.3314. **R$_f$** = 0.56 (1:9 MeOH/DCM, UV, CAM).

(*R*)-[Me, Cy]$^{Me, (CH_2)_2SPh}$CAAC(H$^+$)(BF$_4^-$) (8k)

IUPAC: 1-((*R*)-1-cyclohexylethyl)-2,2,4-trimethyl-4-(2-(phenylthio)ethyl)-3,4-dihydro-2H-pyrrol-1-ium tetrafluoroborate

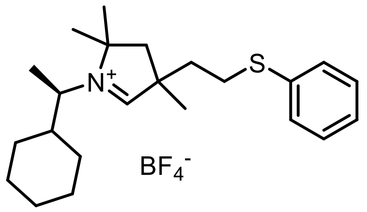

Following **GP4**: 2,4-dimethyl-2-(2-(phenylthio)ethyl)pent-4-enal **6k** (234 mg, 942 µmol) and (*R*)-(–)-1-cyclohexylethylamine (121 mg, 936 µmol) afforded **8k** (180 mg, 403 µmol, 43 % over 3 steps) as a brown gum in a 1.2:1 mixture of diastereomers. **^{1}H NMR** (400 MHz, CD_2Cl_2) δ 8.83 (s, 1H), 8.71 (s, 1.2H), 7.38–7.31 (m, 8.8H), 7.29–7.22 (m, 2.2H), 3.56–3.46 (m, 2.2H), 3.20–3.07 (m, 2.2H), 3.00–2.91 (m, 2.2H), 2.26 (d, *J* = 14.0 Hz, 1.2H), 2.22 (d, *J* = 13.9 Hz, 1H), 2.19–2.15 (m, 2.4H), 2.13–2.06 (m, 4.2H), 1.93–1.87 (m, 3.2H), 1.86–1.81 (m, 3.2H), 1.77–1.69 (m, 6.8H), 1.59 (s, 3H), 1.54 (s, 3.6H), 1.53–1.49 (m, 13.2H), 1.48 (s, 6.6H), 1.35–1.28 (m, 3.4H), 1.20–1.12 (m, 3.2H), 1.05–0.99 (m, 2.2H), 0.99–0.90 (m, 2.2H). **^{13}C NMR** (101 MHz, CD_2Cl_2) δ 182.9, 182.5, 135.1, 134.8, 130.0, 129.8, 129.7, 129.4, 127.2, 127.1, 78.9, 78.7, 62.3, 62.0, 51.0, 50.9, 46.7, 46.3, 43.0, 42.9, 38.7, 38.3, 31.4, 30.8, 29.5, 29.4, 29.2, 29.1, 28.7, 28.6, 27.9, 27.7, 26.0, 26.0, 26.0, 25.9, 25.8, 25.5, 24.5, 21.1, 20.7. (1C not resolved). **^{19}F NMR** (CD_2Cl_2, 376 MHz) δ -150.74 (^{1}J ^{19}F-^{10}B), -150.79 (^{1}J ^{19}F-^{11}B). **IR** (ATR, neat) 2929, 2855, 1659, 1452, 1381, 1051, 742, 693, 521. **HRMS** (ESI/QTOF) m/z: [M-BF$_4$]$^+$ calculated for $C_{23}H_{36}NS^+$ 358.2563; found 358.2558. **R*f*** = 0.59 (1:9 MeOH/DCM, UV, CAM, intense blue–dia1). **R*f*** = 0.58 (1:9 MeOH/DCM, UV, CAM, faint blue–dia2).

(*R*)-[Me, Cy]$^{CF3, Ph}$CAAC(H$^+$)(BF$_4^-$) (8l)

IUPAC: 1-((*R*)-1-cyclohexylethyl)-2,2-dimethyl-4-phenyl-4-(trifluoromethyl)-3,4-dihydro-2H-pyrrol-1-ium tetrafluoroborate

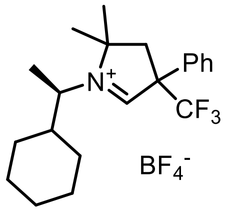

Following **GP4**: 4-methyl-2-phenyl-2-(trifluoromethyl)pent-4-enal **6l** (230 mg, 950 µmol) and (*R*)-(–)-1-cyclohexylethylamine (123 mg, 947 µmol) afforded **8l** (180 mg, 411 µmol, 43 % over 3 steps) as a beige solid in a 1.1:1 mixture of diastereomers. **^{1}H NMR** (400 MHz, CD_2Cl_2) δ 9.73 (s, 1H), 9.68 (s, 1.1H), 7.55–7.48 (m, 10.5H), 3.82 (dq, *J* = 9.3, 6.8 Hz, 1.1H), 3.71 (dq, *J* = 10.7, 6.8 Hz, 1H), δ 3.11 (d, *J* = 14.7 Hz, 1H), δ 3.07 (d, *J* = 14.7 Hz, 1.1H), 2.89 (d, *J* = 14.4 Hz, 1H), 2.86 (d, *J* = 14.2 Hz, 1.1H), 2.27–2.10 (m, 2.1H), 1.99–1.92 (m, 2.1H), 1.84–1.78 (m, 3.2H), 1.74 (s, 3H), 1.70 (s, 3.3H), 1.63 (d, *J* = 6.7 Hz, 3H), 1.60 (d, *J* = 6.7 Hz, 3.3H), 1.56–1.37 (m, 4.2H), 1.34 (s, 3.3H), 1.32 (s, 3H), 1.22–0.94 (m, 9.4H), 0.87–0.73 (m, 2.1H). **^{13}C NMR** (101 MHz, $CDCl_3$) δ 174.8, 174.6, 132.0, 131.8, 131.0, 130.9, 130.6, 130.5, 127.9, 127.7, 125.5 (q, *J* = 282.8 Hz), 124.2 (q, *J* = 282.8 Hz), 80.4, 80.2, 64.9, 64.6, 63.4 (q, *J* = 29.6 Hz), 43.1, 43.0, 42.7, 42.5, 31.0, 30.9, 29.8, 29.3, 27.6, 27.2, 26.6, 26.1, 26.0, 26.0, 25.8, 25.8, 25.7, 25.4, 21.6, 21.4. (1C not resolved). **^{19}F NMR** (CD_2Cl_2, 376 MHz) δ -70.58 (CF_3), -71.01 (CF_3), -150.91 (^{1}J ^{19}F-^{10}B), -150.96 (^{1}J ^{19}F-^{11}B). **IR** (ATR, neat) 2933, 2857, 1452, 1278, 1166, 1053, 710. **HRMS** (ESI/QTOF) m/z: [M-BF$_4$]$^+$ calculated for $C_{21}H_{29}F_3N^+$ 352.2247; found 352.2250. **R*f*** = 0.32 (1:9 MeOH/DCM, UV, faint stain CAM).

Diastereomerically pure carbene precursors

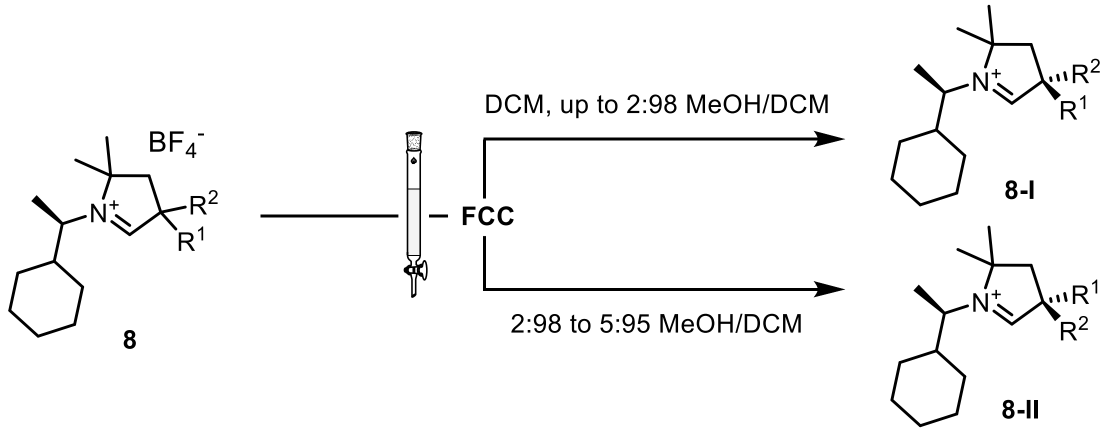

General Procedure (GP5)

The diastereomeric carbene precursor mixture (0.1-0.2 mmol) was separated by flash column chromatography (SiliaFlash 40A, conditioning: DCM, eluent: DCM, 0.5:99.5 *then* 1:99 *then* 2:98 MeOH/DCM) to afford the first eluting diastereomerically pure carbene precursor **8-I**. Then, the ratio of methanol was increased by 1 % increment (eluent: 2:98 to 5:95 MeOH/DCM) to afford the second eluting diastereomerically pure carbene precursor **8-II**.

(*R*,*S*)-[Me, Cy]$^{Me, Ph}$CAAC(H$^+$)(BF$_4^-$) (8a-I)

IUPAC: (*S*)-1-((*R*)-1-cyclohexylethyl)-2,2,4-trimethyl-4-phenyl-3,4-dihydro-2H-pyrrol-1-ium tetrafluoroborate

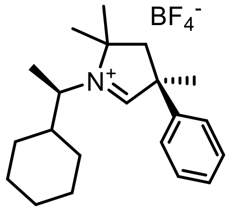

Following **GP5**: the diastereomeric carbene precursor mixture **8a** (100 mg, 260 µmol) afforded **8a-I** (42.1 mg, 109 µmol, 42 %) as a beige solid. **^{1}H NMR** (400 MHz, CD_2Cl_2) δ 9.12 (s, 1H), 7.48–7.43 (m, 2H), 7.39–7.35 (m, 1H), 7.31–7.28 (m, 2H), 3.63 (dq, *J* = 9.4, 6.7 Hz, 1H), 2.76 (d, *J* = 13.9 Hz, 1H), 2.45 (d, *J* = 13.9 Hz, 1H), 2.08–1.92 (m, 2H), 1.87–1.83 (m, 1H), 1.81 (s, 3H), 1.78–1.70 (m, 2H), 1.63 (s, 3H), 1.60–1.55 (m, 1H), 1.56 (d, *J* = 6.7 Hz, 3H), 1.47 (s, 3H), 1.44–1.33 (m, 2H), 1.22–1.14 (m, 1H), 1.12–1.04 (m, 1H), 1.03–0.95 (m, 1H). **^{13}C NMR** (101 MHz, CD_2Cl_2) δ 182.3, 141.4, 130.2, 130.2, 128.8, 125.8, 125.8, 79.5, 62.6, 54.5, 50.0, 42.8, 31.0, 29.4, 27.8, 27.7, 27.3, 26.1, 25.9, 25.8, 21.7. **^{19}F NMR** (CD_2Cl_2, 376 MHz) δ -150.64 (^{1}J ^{19}F-^{10}B), -150.70 (^{1}J ^{19}F-^{11}B). **IR** (ATR, neat) 2930, 2856, 1661, 1448, 1387, 1053, 765, 701. **HRMS** (Nanochip-based ESI/QTOF) m/z: [M-BF$_4$]$^+$ calculated for $C_{21}H_{32}N^+$ 298.2529; found 298.2521. **[α]$_D^{21}$** = -48.33 (c = 0.10, $CHCl_3$). **mp** 123.7-126.8 °C. **R$_f$** = 0.5 (1:9 MeOH/DCM, UV, CAM–intense blue spot).

(*R*,*R*)-[Me, Cy]$^{Me, Ph}$CAAC(H$^+$)(BF$_4^-$) (8a-II)

IUPAC: (*R*)-1-((*R*)-1-cyclohexylethyl)-2,2,4-trimethyl-4-phenyl-3,4-dihydro-2H-pyrrol-1-ium tetrafluoroborate

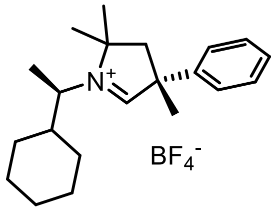

Following **GP5**: the diastereomeric carbene precursor mixture **8a** (100 mg, 0.26 mmol) afforded **8a-II** (33.9 mg, 87.9 µmol, 34 %) as a beige solid. **^{1}H NMR** (400 MHz, CD_2Cl_2) δ 9.27 (s, 1H), 7.47–7.43 (m, 2H), 7.38–7.31 (m, 3H), 3.59 (dq, *J* = 8.9, 6.8 Hz, 1H), 2.80 (d, *J* = 13.9 Hz, 1H), 2.47 (d, *J* = 13.9 Hz, 1H), 1.99–1.88 (m, 2H), 1.85–1.79 (m, 1H), 1.81 (s, 3H), 1.68 (s, 3H), 1.70–1.65 (m, 2H), 1.61 (d, *J* = 6.8 Hz, 3H), 1.43 (s, 3H), 1.41–1.28 (m, 2H), 1.27–1.19 (m, 1H), 1.17–1.09 (m, 1H), 1.08–0.98 (m, 1H), 0.88 (qd, *J* = 12.3, 3.3 Hz, 1H). **^{13}C NMR** (101 MHz, CD_2Cl_2) δ 182.3, 141.4, 130.1, 130.1, 128.8, 125.9, 125.9, 79.2, 62.6, 54.8, 49.3, 43.3, 31.1, 28.0, 26.7, 26.0, 25.9, 25.8, 20.8. **^{19}F NMR** (CD_2Cl_2, 376 MHz) δ -150.60 (^{1}J ^{19}F-^{10}B), -150.65 (^{1}J ^{19}F-^{11}B). **IR** (ATR, neat) 2931, 2856, 1661, 1449, 1381, 1051, 765, 702. **HRMS** (Nanochip-based ESI/QTOF) m/z: [M-BF$_4$]$^+$ calculated for $C_{21}H_{32}N^+$ 298.2529; found 298.2526. **[α]$_D^{21}$** = -73.33 (c = 0.10, $CHCl_3$). **mp** 157.5-161.0 °C. **R$_f$** = 0.47 (1:9 MeOH/DCM, UV, CAM–faint blue spot).

(*R*,*S*)-[Me, Cy]$^{Me, 1-Np}$CAAC(H^{+})(BF$_4^{-}$) (8b-I)

IUPAC: (*S*)-1-((*R*)-1-cyclohexylethyl)-2,2,4-trimethyl-4-(naphthalen-1-yl)-3,4-dihydro-2H-pyrrol-1-ium tetrafluoroborate

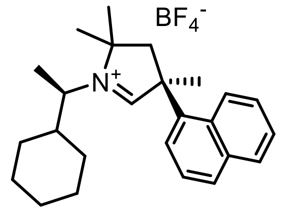

Following **GP5**: the diastereomeric carbene precursor mixture **8b** (52.1 mg, 120 µmol) afforded **8b-I** (19.8 mg, 45.4 µmol, 38 %) as a beige solid. **^{1}H NMR** (400 MHz, CD_2Cl_2) δ 9.50 (s, 1H), 7.97–7.91 (m, 2H), 7.87 (d, *J* = 8.3 Hz, 1H), 7.63–7.55 (m, 2H), 7.45 (dd, *J* = 8.3, 7.2 Hz, 1H), 7.23 (dd, *J* = 7.2, 1.1 Hz, 1H), 3.71 (dq, *J* = 9.2, 6.7 Hz, 1H), 3.06 (d, *J* = 13.6 Hz, 1H), 2.82 (d, *J* = 13.6 Hz, 1H), 2.20–2.11 (m, 1H), 2.06 (s, 3H), 2.02–1.97 (m, 1H), 1.90–1.74 (m, 3H), 1.71 (d, *J* = 6.7 Hz, 3H), 1.67 (s, 3H), 1.65–1.61 (m, 1H), 1.50–1.38 (m, 2H), 1.36 (s, 3H), 1.25–1.14 (m, 1H), 1.13–1.00 (m, 2H). **^{13}C NMR** (101 MHz, CD_2Cl_2) δ 183.6, 138.3, 135.8, 130.6, 130.0, 129.3, 126.9, 126.5, 125.7, 124.5, 123.2, 80.1, 62.8, 54.6, 50.3, 42.9, 31.2, 29.4, 28.3, 27.4, 27.2, 26.1, 25.9, 21.6. (1C not resolved). **^{19}F NMR** (CD_2Cl_2, 376 MHz) δ -150.80 (^{1}J ^{19}F-^{10}B), -150.86 (^{1}J ^{19}F-^{11}B). **IR** (ATR, neat) 2932, 2856, 1668, 1452, 1381, 1057, 805, 779. **HRMS** (Nanochip-based ESI/QTOF) m/z: $[M-BF_4]^+$ calculated for $C_{25}H_{34}N^+$ 348.2686; found 348.2681. **$[\alpha]_D^{21}$** = -163.33 (c = 0.10, $CHCl_3$). **mp** 173.8-176.4 °C. **R$_f$** = 0.39 (1:9 MeOH/DCM, UV, CAM).

(*R*,*R*)-[Me, Cy]$^{Me, 1-Np}$CAAC(H^{+})(BF$_4^{-}$) (8b-II)

IUPAC: (*R*)-1-((*R*)-1-cyclohexylethyl)-2,2,4-trimethyl-4-(naphthalen-1-yl)-3,4-dihydro-2H-pyrrol-1-ium tetrafluoroborate

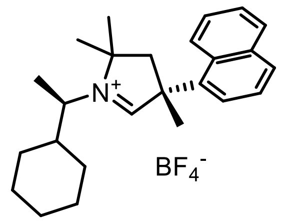

Following **GP5**: the diastereomeric carbene precursor mixture **8b** (52.1 mg, 120 µmol) afforded **8b-II** (20.9 mg, 48.0 µmol, 40 %) as a beige solid. **^{1}H NMR** (400 MHz, CD_2Cl_2) δ 9.56 (s, 1H), 7.97–7.93 (m, 2H), 7.87 (d, *J* = 8.3 Hz, 1H), 7.63–7.55 (m, 2H), 7.43 (t, *J* = 7.8 Hz, 1H), 7.21 (d, *J* = 7.2 Hz, 1H), 3.68 (dq, *J* = 9.4, 6.8 Hz, 1H), 2.99 (d, *J* = 13.6 Hz, 1H), 2.92 (d, *J* = 13.7 Hz, 1H), 2.18–2.08 (m, 1H), 2.05 (s, 3H), 2.05–2.00 (m, 1H), 1.90–1.85 (m, 1H), 1.79–1.73 (m, 2H), 1.72 (s, 3H), 1.69 (d, *J* = 6.7 Hz, 3H), 1.66–1.61 (m, 1H), 1.50–1.38 (m, 2H), 1.36 (s, 3H), 1.19 (ddd, *J* = 16.7, 8.5, 3.9 Hz, 1H), 1.09 (qd, *J* = 12.3, 3.7 Hz, 1H), 0.98 (qd, *J* = 12.4, 3.5 Hz, 1H). **^{13}C NMR** (101 MHz, CD_2Cl_2) δ 182.9, 138.6, 135.8, 130.5, 130.0, 129.4, 127.0, 126.5, 125.5, 124.6, 122.9, 79.8, 63.1, 55.0, 50.6, 43.5, 31.5, 29.5, 28.5, 27.4, 26.9, 26.1, 26.0, 25.8, 21.0. **^{19}F NMR** (CD_2Cl_2, 376 MHz) δ -150.51 (^{1}J ^{19}F-^{10}B), -150.57 (^{1}J ^{19}F-^{11}B). **IR** (ATR, neat) 2931, 2856, 1667, 1452, 1389, 1052, 804, 778. **HRMS** (Nanochip-based ESI/QTOF) m/z: $[M-BF_4]^+$ calculated for $C_{25}H_{34}N^+$ 348.2686; found 348.2696. **$[\alpha]_D^{21}$** = +55.00 (c = 0.10, $CHCl_3$). **mp** 176.7-179.5 °C. **R$_f$** = 0.33 (1:9 MeOH/DCM, UV, CAM).

(*R*,*S*)-[Me, Cy]$^{Me, 2\text{-}Np}$CAAC(H$^+$)(BF$_4^-$) (8c-I)

IUPAC: (*S*)-1-((*R*)-1-cyclohexylethyl)-2,2,4-trimethyl-4-(naphthalen-2-yl)-3,4-dihydro-2H-pyrrol-1-ium tetrafluoroborate

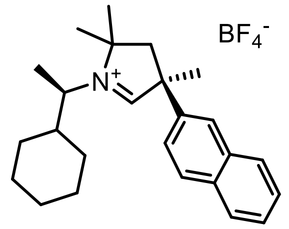

Following **GP5**: the diastereomeric carbene precursor mixture **8c** (55.8 mg, 128 µmol) afforded **8c-I** (23.4 mg, 53.8 µmol, 42 %) as a beige solid. **^{1}H NMR** (400 MHz, CD_2Cl_2) δ 9.32 (s, 1H), 7.95 (d, *J* = 8.7 Hz, 1H), 7.95–7.91 (m, 1H), 7.87–7.83 (m, 1H), 7.81 (d, *J* = 2.1 Hz, 1H), 7.56–7.51 (m, 2H), 7.41–7.38 (m, 1H), 3.64 (dq, *J* = 9.4, 6.7 Hz, 1H), 2.88 (d, *J* = 13.9 Hz, 1H), 2.51 (d, *J* = 13.9 Hz, 1H), 2.12–2.03 (m, 1H), 1.98–1.92 (m, 1H), 1.87 (s, 3H), 1.83–1.70 (m, 3H), 1.64 (s, 3H), 1.58 (d, *J* = 6.7 Hz, 3H), 1.46 (s, 3H), 1.46–1.34 (m, 2H), 1.23–1.12 (m, 2H), 1.09–0.99 (m, 2H). **^{13}C NMR** (101 MHz, CD_2Cl_2) δ 182.4, 138.6, 133.8, 133.0, 130.3, 128.7, 127.9, 127.4, 127.3, 124.8, 123.3, 79.5, 62.6, 54.7, 49.6, 42.8, 31.0, 29.4, 28.2, 27.7, 27.3, 26.1, 25.9, 25.8, 21.6. **^{19}F NMR** (CD_2Cl_2, 376 MHz) δ -150.34 (^{1}J ^{19}F-^{10}B), -150.39 (^{1}J ^{19}F-^{11}B). **IR** (ATR, neat) 2932, 2856, 1661, 1452, 1057, 822, 754. **HRMS** (ESI/QTOF) m/z: [M-BF$_4$]$^+$ calculated for $C_{25}H_{34}N^+$ 348.2686; found 348.2691. **$[\alpha]_D^{21}$** = -33.33 (c = 0.10, $CHCl_3$). **mp** 148.6-151.6 °C. **R$_f$** = 0.43 (1:9 MeOH/DCM, UV, CAM).

(*R*,*R*)-[Me, Cy]$^{Me, 2\text{-}Np}$CAAC(H$^+$)(BF$_4^-$) (8c-II)

IUPAC: (*R*)-1-((*R*)-1-cyclohexylethyl)-2,2,4-trimethyl-4-(naphthalen-2-yl)-3,4-dihydro-2H-pyrrol-1-ium tetrafluoroborate

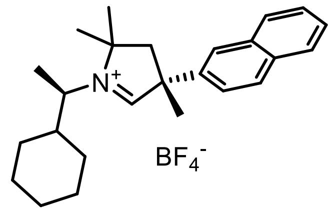

Following **GP5**: the diastereomeric carbene precursor mixture **8c** (55.8 mg, 128 µmol) afforded **8c-II** (16.7 mg, 38.4 µmol, 30 %) as a beige solid. **^{1}H NMR** (400 MHz, CD_2Cl_2) δ 9.35 (s, 1H), 7.96 (d, *J* = 8.7 Hz, 1H), 7.90–7.84 (m, 2H), 7.78 (d, *J* = 2.1 Hz, 1H), 7.57–7.51 (m, 2H), 7.42 (dd, *J* = 8.7, 2.1 Hz, 1H), 3.60 (dq, *J* = 9.2, 6.8 Hz, 1H), 2.92 (d, *J* = 13.9 Hz, 1H), 2.53 (d, *J* = 13.9 Hz, 1H), 2.06–1.91 (m, 2H), 1.88 (s, 3H), 1.86–1.80 (m, 1H), 1.70 (s, 3H), 1.69–1.65 (m, 2H), 1.64 (d, *J* = 6.8 Hz, 3H), 1.48–1.43 (m, 1H), 1.41 (s, 3H), 1.39–1.33 (m, 1H), 1.32–1.24 (m, 1H), 1.17–0.99 (m, 2H), 0.87 (qd, *J* = 12.4, 3.5 Hz, 1H). **^{13}C NMR** (101 MHz, CD_2Cl_2) δ 182.3, 138.7, 133.7, 133.0, 130.3, 128.5, 128.0, 127.5, 127.4, 124.8, 123.4, 79.3, 62.7, 54.8, 49.3, 43.4, 31.2, 29.3, 28.3, 28.0, 26.6, 26.0, 25.9, 25.8, 20.9. **^{19}F NMR** (CD_2Cl_2, 376 MHz) δ -150.36 (^{1}J ^{19}F-^{10}B), -150.41 (^{1}J ^{19}F-^{11}B). **IR** (ATR, neat) 2931, 2856, 1662, 1452, 1058, 823, 753. **HRMS** (ESI/QTOF) m/z: [M-BF$_4$]$^+$ calculated for $C_{25}H_{34}N^+$ 348.2686; found 348.2690. **$[\alpha]_D^{21}$** = -93.33 (c = 0.10, $CHCl_3$). **mp** 205.8-209.4 °C. **R$_f$** = 0.41 (1:9 MeOH/DCM, UV, CAM).

(*R*,*S*)-[Me, Cy]^{THN}CAAC(H$^+$)(BF_4^-) (8h-I)

IUPAC: (*S*)-1'-((*R*)-1-cyclohexylethyl)-5',5'-dimethyl-3,4,4',5'-tetrahydro-2H-spiro[naphthalene-1,3'-pyrrol]-1'-ium tetrafluoroborate

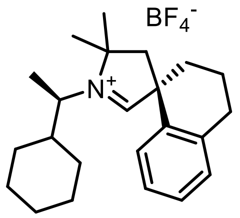

Following **GP5**: the diastereomeric carbene precursor mixture **8h** (50.9 mg, 124 µmol) afforded **8h-I** (22.1 mg, 53.7 µmol, 43 %) as a beige solid. **^{1}H NMR** (400 MHz, CD_2Cl_2) δ 8.49 (s, 1H), 7.23–7.24 (m, 3H), 6.69 (dd, *J* = 7.3, 1.6 Hz, 1H), 3.69 (dq, *J* = 9.5, 6.7 Hz, 1H), 2.90 (dd, *J* = 8.4, 4.3 Hz, 2H), 2.65 (d, *J* = 14.1 Hz, 1H), 2.41 (dd, *J* = 14.1, 1.1 Hz, 1H), 2.28–2.21 (m, 1H), 2.17–2.10 (m, 1H), 1.97–1.87 (m, 3H), 1.87–1.77 (m, 3H), 1.74 (s, 3H), 1.72–1.66 (m, 1H), 1.65 (s, 3H), 1.64 (d, *J* = 6.6 Hz, 3H), 1.59–1.53 (m, 1H), 1.41–1.28 (m, 2H), 1.22–1.06 (m, 2H), 1.04–0.94 (m, 1H). **^{13}C NMR** (101 MHz, CD_2Cl_2) δ 180.6, 137.3, 134.7, 131.1, 129.1, 127.9, 127.8, 79.3, 62.6, 54.9, 52.0, 43.0, 43.0, 30.7, 29.6, 28.8, 28.8, 28.3, 26.0, 25.9, 25.8, 22.0, 19.1. **^{19}F NMR** (CD_2Cl_2, 376 MHz) δ -151.49 (^{1}J ^{19}F-^{10}B), -151.54 (^{1}J ^{19}F-^{11}B). **IR** (ATR, neat) 2932, 2856, 1657, 1450, 1054, 763. **HRMS** (ESI/QTOF) m/z: [M-BF_4]$^+$ calculated for $C_{23}H_{34}N^+$ 324.2686; found 324.2688. **[α]$_D^{21}$** = -3.33 (c = 0.10, $CHCl_3$). **mp** 170.4-172.5 °C. **R$_f$** = 0.42 (1:9 MeOH/DCM, UV, CAM).

(*R*,*R*)-[Me, Cy]^{THN}CAAC(H$^+$)(BF_4^-) (8h-II)

IUPAC: (*R*)-1'-((*R*)-1-cyclohexylethyl)-5',5'-dimethyl-3,4,4',5'-tetrahydro-2H-spiro[naphthalene-1,3'-pyrrol]-1'-ium tetrafluoroborate

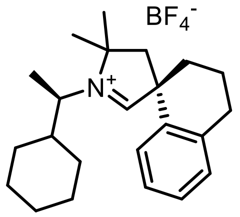

Following **GP5**: the diastereomeric carbene precursor mixture **8h** (50.9 mg, 124 µmol) afforded **8h-II** (13.7 mg, 33.3 µmol, 27 %) as a beige solid. **^{1}H NMR** (400 MHz, CD_2Cl_2) δ 8.53 (s, 1H), 7.32–7.24 (m, 3H), 6.74–6.71 (m, 1H), 3.79–3.72 (m, 1H), 2.92–2.88 (m, 2H), 2.66 (d, *J* = 14.2 Hz, 1H), 2.41 (d, *J* = 14.2 Hz, 1H), 2.33–2.25 (m, 1H), 2.17–2.09 (m, 1H), 2.02–1.89 (m, 2H), 1.88–1.80 (m, 4H), 1.79–1.73 (m, 2H), 1.71 (s, 3H), 1.69 (s, 3H), 1.59 (d, *J* = 6.8 Hz, 3H), 1.36–1.26 (m, 2H), 1.25–1.10 (m, 3H). **^{13}C NMR** (101 MHz, CD_2Cl_2) δ 180.6, 137.8, 134.6, 131.2, 129.1, 128.0, 127.7, 79.3, 62.4, 55.2, 51.8, 43.5, 33.2, 31.4, 29.3, 29.1, 28.8, 28.0, 26.1, 26.0, 26.0, 20.9, 19.2. **^{19}F NMR** (CD_2Cl_2, 376 MHz) δ -151.47 (^{1}J ^{19}F-^{10}B), -151.52 (^{1}J ^{19}F-^{11}B). **IR** (ATR, neat) 2931, 2856, 1656, 1450, 1049, 1032, 746. **HRMS** (ESI/QTOF) m/z: [M-BF_4]$^+$ calculated for $C_{23}H_{34}N^+$ 324.2686; found 324.2686. **[α]$_D^{21}$** = -160.00 (c = 0.10, $CHCl_3$). **mp** 202.3-205.3 °C. **R$_f$** = 0.42 (1:9 MeOH/DCM, UV, CAM).

(*R*,*S*)-[Me, Cy]$^{Me, (CH_2)_2SPh}$CAAC(H$^+$)(BF$_4^-$) (8k-I)

IUPAC: (*S*)-1-((*R*)-1-cyclohexylethyl)-2,2,4-trimethyl-4-(2-(phenylthio)ethyl)-3,4-dihydro-2H-pyrrol-1-ium tetrafluoroborate

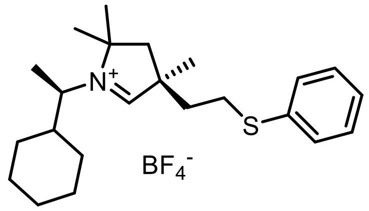

Following **GP5**: the diastereomeric carbene precursor mixture **8k** (42.3 mg, 94.9 µmol) afforded **8k-I** (13.9 mg, 31.3 µmol, 33 %) as a pale brown gum. **^{1}H NMR** (400 MHz, $CDCl_3$) δ 9.02 (s, 1H), 7.36–7.30 (m, 4H), 7.24–7.20 (m, 1H), 3.42 (dq, *J* = 9.5, 6.7 Hz, 1H), 3.14–3.07 (m, 1H), 3.02–2.95 (m, 1H), 2.31 (d, *J* = 13.8 Hz, 1H), 2.26–2.13 (m, 2H), 2.07 (d, *J* = 13.8 Hz, 1H), 2.04–1.98 (m, 1H), 1.95–1.89 (m, 1H), 1.83–1.77 (m, 1H), 1.75–1.68 (m, 2H), 1.55–1.50 (m, 13H), 1.46–1.35 (m, 2H), 1.14–1.05 (m, 1H), 1.02–0.92 (m, 1H), 0.91–0.81 (m, 1H). **^{13}C NMR** (101 MHz, $CDCl_3$) δ 183.4, 134.7, 129.6, 129.5, 126.9, 78.2, 62.0, 50.7, 46.4, 42.4, 37.7, 30.7, 29.5, 29.3, 28.6, 27.7, 25.8, 25.5, 25.4, 25.3, 20.8. **^{19}F NMR** ($CDCl_3$, 376 MHz) δ -150.15 (^{1}J ^{19}F-^{10}B), -150.21 (^{1}J ^{19}F-^{11}B). **IR** (ATR, neat) 2931, 2855, 1658, 1452, 1381, 1056, 744, 693, 521. **HRMS** (ESI/QTOF) m/z: [M-BF$_4$]$^+$ calculated for $C_{23}H_{36}NS^+$ 358.2563; found 358.2571. **[α]$_D^{21}$** = -25.33 (c = 0.10, $CHCl_3$). **R$_f$** = 0.59 (1:9 MeOH/DCM, UV, CAM, intense blue).

(*R*,*R*)-[Me, Cy]$^{Me, (CH_2)_2SPh}$CAAC(H$^+$)(BF$_4^-$) (8k-II)

IUPAC: (*R*)-1-((*R*)-1-cyclohexylethyl)-2,2,4-trimethyl-4-(2-(phenylthio)ethyl)-3,4-dihydro-2H-pyrrol-1-ium tetrafluoroborate

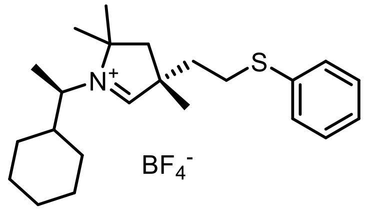

Following **GP5**: the diastereomeric carbene precursor mixture **8k** (42.3 mg, 94.9 µmol) afforded **8k-II** (9.38 mg, 21.0 µmol, 22 %) as a pale brown gum. **^{1}H NMR** (400 MHz, $CDCl_3$) δ 9.04 (s, 1H), 7.37–7.30 (m, 4H), 7.25–7.20 (m, 1H), 3.51–3.44 (m, 1H), 3.06 (ddd, *J* = 13.8, 7.7, 6.3 Hz, 1H), 2.95 (ddd, *J* = 13.4, 7.6, 7.6 Hz, 1H), 2.23 (d, *J* = 13.8 Hz, 1H), 2.17–2.05 (m, 2H), 2.11 (d, *J* = 14.0 Hz, 1H), 1.95–1.86 (m, 2H), 1.82–1.76 (m, 1H), 1.72–1.66 (m, 2H), 1.61–1.59 (m, 1H), 1.60 (s, 3H), 1.56 (d, *J* = 6.8 Hz, 3H), 1.52 (s, 3H), 1.49 (s, 3H), 1.35–1.20 (m, 2H), 1.15–1.07 (m, 1H), 1.05–0.94 (m, 1H), 0.94–0.84 (m, 1H). **^{13}C NMR** (101 MHz, $CDCl_3$) δ 183.7, 134.7, 130.1, 129.5, 127.1, 78.2, 62.3, 50.8, 46.9, 42.5, 38.3, 31.3, 29.6, 29.2, 28.7, 27.8, 25.8, 25.6, 25.6, 24.5, 20.9. **^{19}F NMR** ($CDCl_3$, 376 MHz) δ -150.63 (^{1}J ^{19}F-^{10}B), -150.68 (^{1}J ^{19}F-^{11}B). **IR** (ATR, neat) 2931, 2855, 1656, 1452, 1387, 1053, 743, 693, 521. **HRMS** (ESI/QTOF) m/z: [M-BF$_4$]$^+$ calculated for $C_{23}H_{36}NS^+$ 358.2563; found 358.2558. **[α]$_D^{21}$** = -58.67 (c = 0.10, $CHCl_3$). **R$_f$** = 0.58 (1:9 MeOH/DCM, UV, CAM, intense blue).

Transition metal complexes

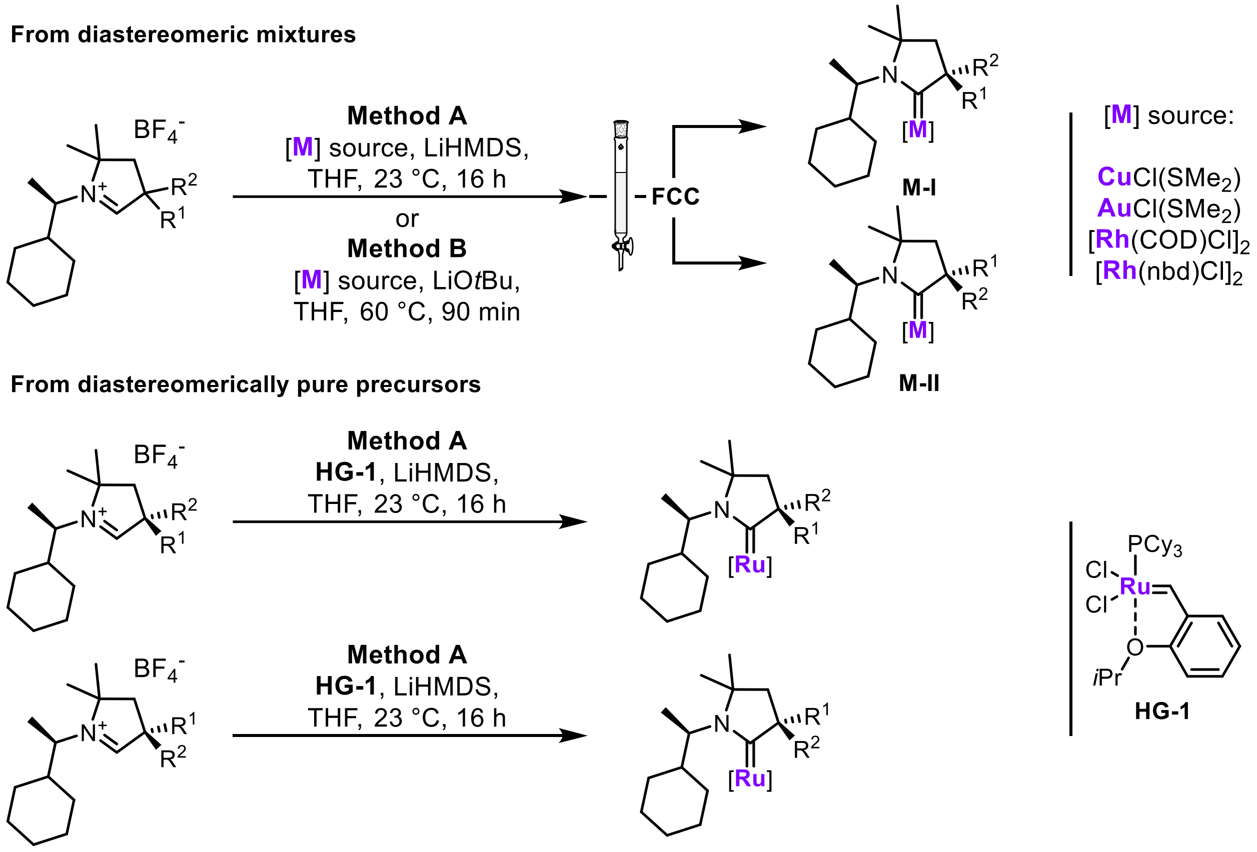

Method A: LiHMDS complexation

In a nitrogen-filled glovebox, THF (0.07 M) was added to a mixture of tetrafluoroborate CAAC precursor (1.0 equiv. (Rh) or 1.2 equiv. (Ru)), LiHMDS (1.05 equiv.) and the metal source (0.5 equiv. (Rh) or 1.0 equiv. (Ru)). The mixture was stirred for 16 h at 23 °C. The volatiles were removed by freeze-dry technique (Schlenk line). The residue was purified by column chromatography (**SiliaFlash 40A**) to afford the corresponding complex.

General purification conditions:

Ruthenium: SiliaFlash 40A, conditioning: 2:8 DCM/pentane, eluent: 2:8 then 4:6 then 8:2 DCM/pentane.

Rhodium: SiliaFlash 40A, conditioning: 5:95 EtOAc/pentane, eluent: 1:9 to 2:8 EtOAc/pentane.

Method B: LiO*t*Bu complexation

In a nitrogen-filled glovebox, THF (0.07 M) was added to a mixture of tetrafluoroborate CAAC precursor (1.0 equiv.), LiO*t*Bu (1.05 equiv.) and the metal source (1.2 equiv.) in a microwave tube. The vessel was sealed, the mixture was taken outside the glovebox and stirred for 90 min at 60 °C. The volatiles were removed by freeze-dry technique (Schlenk line). The residue was purified by column chromatography (**SiliaFlash 40A**) to afford the corresponding complex.

Copper complexes

(*R*,*S*)-[Me, Cy]$^{Me, Ph}$CAAC-CuCl (Cu1)

IUPAC: ((*S*)-1-((*R*)-1-cyclohexylethyl)-3,5,5-trimethyl-3-phenylpyrrolidin-2-ylidene)copper(I) chloride

Purification conditions: SiliaFlash 40A, conditioning: 5:95 EtOAc/pentane, eluent: 20:80 to 25:75 EtOAc/pentane.

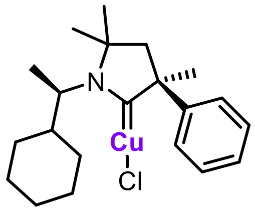

Following Method B using CAAC precursor **8a** (32.6 mg, 84.5 μmol) and CuCl(SMe$_2$) (16.4 mg, 102 μmol) afforded the corresponding copper complex (*R*,*S*)-(**Cu1**) (9.88 mg, 24.9 μmol, 29 %) as a white solid after purification by column chromatography. Suitable crystal for X-ray analysis was obtained by slow evaporation of a 3:1 hexane/DCM mixture in a nitrogen-filled glovebox. **^{1}H NMR** (400 MHz, CDCl$_3$) δ 7.36–7.31 (m, 2H), 7.27–7.21 (m, 3H), 3.28 (dq, *J* = 10.2, 6.5 Hz, 1H), 2.78–2.69 (m, 1H), 2.25 (d, *J* = 13.5 Hz, 1H), 2.11–2.08 (m, 1H), 2.06 (d, *J* = 13.5 Hz, 1H), 1.83–1.78 (m, 1H), 1.76–1.70 (m, 3H), 1.70 (d, *J* = 6.6 Hz, 3H), 1.70 (s, 3H), 1.42 (s, 3H), 1.45–1.37 (m, 1H), 1.34 (s, 3H), 1.36–1.31 (m, 1H), 1.17–1.07 (m, 1H), 1.04–0.94 (m, 1H), 0.88–0.77 (m, 1H). **^{13}C NMR** (101 MHz, CDCl$_3$) δ 237.3, 146.3, 129.1, 127.1, 126.3, 78.5, 60.1, 59.8, 51.8, 45.1, 30.9, 30.7, 28.8, 28.2, 27.9, 26.2, 26.0, 26.0, 23.9. **IR** (ATR, neat) 2970, 2927, 2852, 1550, 1494, 1449, 1376, 1175, 763, 700. **HRMS** (ESI/QTOF) m/z: [M-Cl+CH$_3$CN]$^+$ calculated for $C_{23}H_{34}CuN_2^+$ 401.2012; found 401.2007. **$[\alpha]_D^{21}$** = -123.33 (c = 0.10, CHCl$_3$). **mp** 200.5–203.2 °C (*decomp.*). **R$_f$** = 0.68 (1:1 EtOAc/pentane, UV, slight stain in CAM).

(*R*,*R*)-[Me, Cy]$^{Me, Ph}$CAAC-CuCl (Cu1)

IUPAC: ((*R*)-1-((*R*)-1-cyclohexylethyl)-3,5,5-trimethyl-3-phenylpyrrolidin-2-ylidene)copper(I) chloride

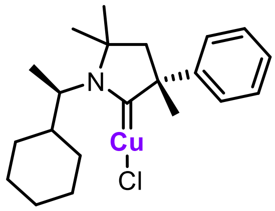

Following Method B using CAAC precursor **8a** (32.6 mg, 84.5 μmol) and CuCl(SMe$_2$) (16.4 mg, 102 μmol) afforded the corresponding copper complex (*R*,*R*)-(**Cu1**) (10.0 mg, 25.2 μmol, 30 %) as a white solid after purification by column chromatography. Suitable crystal for X-ray analysis was obtained by slow evaporation of a 3:1 hexane/DCM mixture in a nitrogen-filled glovebox. **^{1}H NMR** (400 MHz, CDCl$_3$) δ 7.36–7.31 (m, 4H), 7.25–7.21 (m, 1H), 3.29 (dq, *J* = 9.9, 6.5 Hz, 1H), 2.71–2.63 (m, 1H), 2.26 (d, *J* = 13.5 Hz, 1H), 2.10 (d, *J* = 13.5 Hz, 1H), 2.11–2.07 (m, 1H), 1.82–1.70 (m, 4H), 1.68–1.66 (m, 6H), 1.47 (s, 3H), 1.43–1.36 (m, 1H), 1.33 (s, 3H), 1.31–1.27 (m, 1H), 1.17–1.06 (m, 1H), 1.04–0.94 (m, 1H), 0.88–0.78 (m, 1H). **^{13}C NMR** (101 MHz, CDCl$_3$) δ 236.8, 146.1, 129.0, 127.2, 126.4, 78.3, 60.6, 60.0, 51.5, 45.6, 31.3, 30.6, 29.3, 27.9, 27.7, 26.2, 26.0, 25.9, 23.6. **IR** (ATR, neat) 2928, 2852, 1609, 1548, 1511, 1450, 1377, 1252, 1182, 1031, 829. **HRMS** (ESI/QTOF) m/z: [M-Cl+CH$_3$CN]$^+$ calculated for $C_{23}H_{34}CuN_2^+$ 401.2012; found 401.2007. **$[\alpha]_D^{21}$** = -21.67 (c = 0.10, CHCl$_3$). **mp** 204.1–206.7 °C (*decomp.*). **R$_f$** = 0.57 (1:1 EtOAc/pentane, UV, slight stain in CAM).

(*R*,*S*)-[Me, Cy]$^{Me,\ 1\text{-}Np}$CAAC-CuCl (Cu2)

IUPAC: ((*S*)-1-((*R*)-1-cyclohexylethyl)-3,5,5-trimethyl-3-(naphthalen-1-yl)pyrrolidin-2-ylidene)copper(I) chloride

Purification conditions: SiliaFlash 40A, conditioning: pentane, eluent: 1:9 to 2:8 EtOAc/pentane.

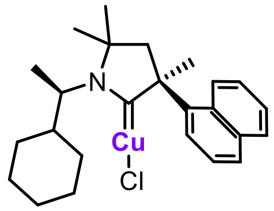

Following Method B using CAAC precursor **8b** (33.7 mg, 77.4 µmol) and $CuCl(SMe_2)$ (15.0 mg, 92.9 µmol) afforded the corresponding copper complex (*R*,*S*)-(**Cu2**) (10.0 mg, 22.4 µmol, 29 %) as a white solid after purification by column chromatography. **^{1}H NMR** (400 MHz, $CDCl_3$) δ 7.91–7.87 (m, 1H), 7.81–7.75 (m, 2H), 7.68 (dd, *J* = 7.3, 1.2 Hz, 1H), 7.49–7.43 (m, 2H), 7.39 (dd, *J* = 8.2, 7.3 Hz, 1H), 3.38 (dq, *J* = 10.2, 6.5 Hz, 1H), 2.87–2.78 (m, 1H), 2.65 (d, *J* = 13.2 Hz, 1H), 2.24 (d, *J* = 13.2 Hz, 1H), 2.15–2.10 (m, 1H), 1.94 (s, 3H), 1.82 (d, *J* = 6.5 Hz, 3H), 1.85–1.71 (m, 4H), 1.48 (s, 3H), 1.47–1.34 (m, 2H), 1.31 (s, 3H), 1.20–1.10 (m, 1H), 1.08–0.98 (m, 1H), 0.93–0.82 (m, 1H). **^{13}C NMR** (101 MHz, $CDCl_3$) δ 239.6, 141.8, 135.3, 130.6, 130.1, 128.7, 125.4, 125.4, 124.9, 124.2, 78.5, 59.9, 50.6, 45.2, 30.9, 30.8, 28.9, 28.2, 28.0, 26.2, 26.0, 26.0, 23.2. (2C not resolved). **IR** (ATR, neat) 2974, 2928, 2853, 1542, 1450, 1377, 916, 802, 778, 731. **HRMS** (nanochip-ESI/LTQ-Orbitrap) m/z: $[M\text{-}Cl+CH_3CN]^+$ calculated for $C_{27}H_{36}CuN_2^+$ 451.2169; found 451.2164. **$[\alpha]_D^{21}$** = -123.81 (c = 0.14, $CHCl_3$). **mp** 202.1–210.0 °C (*decomp.*). **R_f** = 0.67 (1:1 EtOAc/pentane, UV, CAM).

(*R*,*R*)-[Me, Cy]$^{Me,\ 1\text{-}Np}$CAAC-CuCl (Cu2)

IUPAC: ((*S*)-1-((*R*)-1-cyclohexylethyl)-3,5,5-trimethyl-3-(naphthalen-1-yl)pyrrolidin-2-ylidene)copper(I) chloride

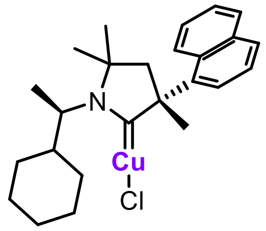

Following Method B using CAAC precursor **8b** (33.7 mg, 77.4 µmol) and $CuCl(SMe_2)$ (15.0 mg, 92.9 µmol) afforded the corresponding copper complex (*R*,*R*)-(**Cu2**) (10.5 mg, 23.5 µmol, 30 %) as a white solid after purification by column chromatography. **^{1}H NMR** (400 MHz, $CDCl_3$) δ 7.90–7.86 (m, 2H), 7.80–7.75 (m, 2H), 7.49–7.41 (m, 2H), 7.38 (dd, *J* = 8.2, 7.3 Hz, 1H), 3.41 (dq, *J* = 9.8, 6.6 Hz, 1H), 2.90–2.81 (m, 1H), 2.60 (d, *J* = 13.3 Hz, 1H), 2.38 (d, *J* = 13.3 Hz, 1H), 2.18–2.13 (m, 1H), 1.98–1.93 (m, 1H), 1.91 (s, 3H), 1.87–1.75 (m, 3H), 1.73 (d, *J* = 6.6 Hz, 3H), 1.53 (s, 3H), 1.48–1.40 (m, 2H), 1.29 (s, 3H), 1.22–1.12 (m, 1H), 1.09–0.999 (m, 1H), 1.00–0.90 (m, 1H). **^{13}C NMR** (101 MHz, $CDCl_3$) δ 238.4, 142.2, 135.3, 130.6, 130.1, 128.6, 125.4, 125.4, 125.3, 125.0, 124.1, 78.5, 60.3, 51.0, 45.5, 32.0, 30.4, 29.2, 28.8, 27.8, 26.2, 26.1, 26.0, 23.3. (1C not resolved). **IR** (ATR, neat) 2973, 2925, 2853, 1536, 1449, 1377, 916, 802, 778, 731. **HRMS** (nanochip-ESI/LTQ-Orbitrap) m/z: $[M\text{-}Cl+CH_3CN]^+$ calculated for $C_{27}H_{36}CuN_2^+$ 451.2169; found 451.2165. **$[\alpha]_D^{21}$** = +36.36 (c = 0.11, $CHCl_3$). **mp** 139.9–143.9 °C (*decomp.*). **R_f** = 0.53 (1:1 EtOAc/pentane, UV, CAM).

(*R*,*S*)-[Me, Cy]$^{Me, 2\text{-}Np}$CAAC-CuCl (Cu3)

IUPAC: ((*S*)-1-((*R*)-1-cyclohexylethyl)-3,5,5-trimethyl-3-(naphthalen-2-yl)pyrrolidin-2-ylidene)copper(I) chloride

Purification conditions: SiliaFlash 40A, conditioning: pentane, eluent: 1:9 to 2:8 EtOAc/pentane.

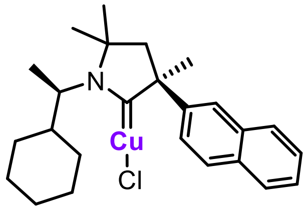

Following Method B using CAAC precursor **8c** (33.5 mg, 76.8 µmol) and CuCl(SMe$_2$) (14.9 mg, 92.2 µmol) afforded the corresponding copper complex (*R,S*)-(**Cu3**) (9.59 mg, 21.6 µmol, 28 %) as a white solid after purification by column chromatography. **^{1}H NMR** (400 MHz, CDCl$_3$) δ 7.83–7.78 (m, 4H), 7.50–7.44 (m, 2H), 7.30 (dd, *J* = 8.6, 1.9 Hz, 1H), 3.32 (dq, *J* = 10.4, 6.5 Hz, 1H), 2.82–2.72 (m, 1H), 2.34 (d, *J* = 13.6 Hz, 1H), 2.12 (d, *J* = 13.5 Hz, 1H), 2.14–2.08 (m, 1H), 1.81 (s, 3H), 1.83–1.70 (m, 4H), 1.75 (d, *J* = 6.5 Hz, 3H), 1.45 (s, 3H), 1.49–1.29 (m, 2H), 1.37 (s, 3H), 1.20–1.08 (m, 1H), 1.06–0.96 (m, 1H), 0.89–0.79 (m, 1H). **^{13}C NMR** (101 MHz, CDCl$_3$) δ 237.3, 143.4, 133.4, 132.4, 129.0, 128.3, 127.6, 126.5, 126.2, 124.9, 124.6, 78.5, 60.3, 59.9, 51.5, 45.2, 30.9, 30.7, 28.9, 28.2, 27.9, 26.2, 26.0, 26.0, 23.9. **IR** (ATR, neat) 2971, 2928, 2852, 1599, 1450, 1377, 916, 816, 749, 731. **HRMS** (ESI/QTOF) m/z: [M-Cl+CH$_3$CN]$^+$ calculated for $C_{27}H_{36}CuN_2^+$ 451.2169; found 451.2183. **[α]$_D^{21}$** = -111.67 (c = 0.10, CHCl$_3$). **mp** 206.5–210.8 °C (*decomp.*). **R$_f$** = 0.68 (1:1 EtOAc/pentane, UV, CAM).

(*R*,*R*)-[Me, Cy]$^{Me, 2\text{-}Np}$CAAC-CuCl (Cu3)

IUPAC: ((*R*)-1-((*R*)-1-cyclohexylethyl)-3,5,5-trimethyl-3-(naphthalen-2-yl)pyrrolidin-2-ylidene)copper(I) chloride

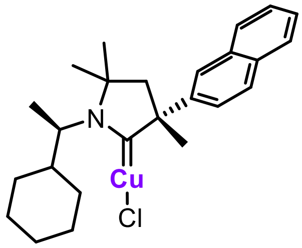

Following Method B using CAAC precursor **8c** (33.5 mg, 76.8 µmol) and CuCl(SMe$_2$) (14.9 mg, 92.2 µmol) afforded the corresponding copper complex (*R,R*)-(**Cu3**) (8.70 mg, 19.4 µmol, 25 %) as a white solid after purification by column chromatography. **^{1}H NMR** (400 MHz, CDCl$_3$) δ 7.84–7.76 (m, 4H), 7.50–7.44 (m, 2H), 7.37 (dd, *J* = 8.7, 1.9 Hz, 1H), 3.33 (dq, *J* = 10.0, 6.6 Hz, 1H), 2.79–2.69 (m, 1H), 2.37 (d, *J* = 13.5 Hz, 1H), 2.15 (d, *J* = 13.5 Hz, 1H), 2.13–2.08 (m, 1H), 1.86–1.79 (m, 2H), 1.77 (s, 3H), 1.76–1.71 (m, 1H), 1.70 (d, *J* = 6.6 Hz, 3H), 1.50 (s, 3H), 1.48–1.30 (m, 3H), 1.33 (s, 3H), 1.20–1.08 (m, 1H), 1.06–0.96 (m, 1H), 0.86 (qd, *J* = 12.5, 3.3 Hz, 1H). **^{13}C NMR** (101 MHz, CDCl$_3$) δ 237.1, 143.4, 133.4, 132.4, 129.0, 128.3, 127.6, 126.6, 126.3, 124.9, 124.7, 78.4, 60.6, 60.1, 51.4, 45.8, 31.5, 30.6, 29.4, 27.9, 27.7, 26.2, 26.0, 25.9, 23.6. **IR** (ATR, neat) 2971, 2927, 2853, 1599, 1450, 1377, 917, 816, 749, 732. **HRMS** (ESI/QTOF) m/z: [M-Cl+CH$_3$CN]$^+$ calculated for $C_{27}H_{36}CuN_2^+$ 451.2169; found 451.2175. **[α]$_D^{21}$** = -41.67 (c = 0.10, CHCl$_3$). **mp** 170.8–174.3 °C (*decomp.*). **R$_f$** = 0.53 (1:1 EtOAc/pentane, UV, CAM).

(*R*,*S*)-[Me, Cy]$^{Me,\ 3,5\text{-}diFPh}$CAAC-CuCl (Cu4)

IUPAC: ((*S*)-1-((*R*)-1-cyclohexylethyl)-3-(3,5-difluorophenyl)-3,5,5-trimethylpyrrolidin-2-ylidene)copper(I) chloride

Purification conditions: SiliaFlash 40A, conditioning: pentane, eluent: 10:90 then 20:80, then 25:75, then 30:70 then 1:1 EtOAc/pentane.

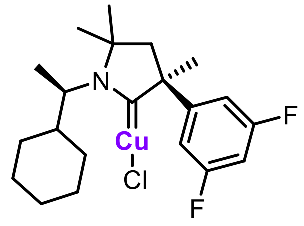

Following Method B using CAAC precursor **8d** (33.0 mg, 78.3 μmol) and CuCl(SMe$_2$) (15.15 mg, 94.0 μmol) afforded the corresponding copper complex (*R*,*S*)-(**Cu4**) (10.4 mg, 24.0 μmol, 31 %) as a white solid after purification by column chromatography. **^{1}H NMR** (400 MHz, CDCl$_3$) δ 6.80–6.66 (m, 3H), 3.30 (dq, *J* = 10.2, 6.5 Hz, 1H), 2.72 (dtd, *J* = 13.9, 11.0, 3.2 Hz, 1H), 2.16 (d, *J* = 13.6 Hz, 1H), 2.08 (d, *J* = 13.6 Hz, 1H), 2.11–2.06 (m, 1H), 1.83–1.77 (m, 1H), 1.75–1.68 (m, 3H), 1.71 (d, *J* = 6.5 Hz, 3H), 1.67 (s, 3H), 1.43 (s, 3H), 1.42–1.39 (m, 1H), 1.38 (s, 3H), 1.31 (ddd, *J* = 14.1, 10.2, 3.9 Hz, 1H), 1.12 (qt, *J* = 12.5, 3.3 Hz, 1H), 1.05–0.95 (m, 1H), 0.82 (qd, *J* = 12.8, 3.8 Hz, 1H). **^{13}C NMR** (101 MHz, CDCl$_3$) δ 235.4, 163.3 (dd, *J* = 249.4, 13.0 Hz), 150.1 (t, *J* = 8.2 Hz), 109.5 (dd, *J* = 26.1, 7.6 Hz), 102.9 (t, *J* = 25.4 Hz), 78.7, 60.1, 60.0, 51.7, 45.3, 30.9, 30.7, 29.0, 28.1, 27.2, 26.2, 26.0, 25.9, 23.9. **^{19}F NMR** (CDCl$_3$, 376 MHz) δ -108.04 (t, *J* = 8.7 Hz). **IR** (ATR, neat) 2972, 2929, 2853, 1622, 1595, 1450, 1432, 1323, 1119, 986, 853, 695. **HRMS** (ESI/QTOF) m/z: [M-Cl+CH$_3$CN]$^+$ calculated for $C_{23}H_{32}CuF_2N_2^+$ 437.1824; found 437.1825. **[α]$_D^{21}$** = -100.00 (c = 0.10, CHCl$_3$). **mp** 241.8–244.5 °C (*decomp.*). **R$_f$** = 0.65 (1:1 EtOAc/pentane, UV, CAM).

(*R*,*R*)-[Me, Cy]$^{Me,\ 3,5\text{-}diFPh}$CAAC-CuCl (Cu4)

IUPAC: ((*R*)-1-((*R*)-1-cyclohexylethyl)-3-(3,5-difluorophenyl)-3,5,5-trimethylpyrrolidin-2-ylidene)copper(I) chloride

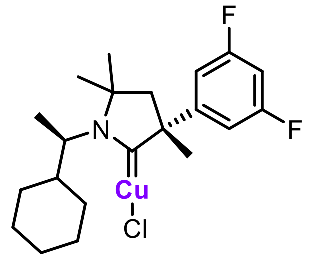

Following Method B using CAAC precursor **8d** (33.0 mg, 78.3 μmol) and CuCl(SMe$_2$) (15.15 mg, 94.0 μmol) afforded the corresponding copper complex (*R*,*R*)-(**Cu4**) (11.17 mg, 25.8 μmol, 33 %) as a white solid after purification by column chromatography **^{1}H NMR** (400 MHz, CDCl$_3$) δ 6.81–6.76 (m, 2H), 6.70 (tt, *J* = 8.7, 2.3 Hz, 1H), 3.32 (dq, *J* = 10.0, 6.6 Hz, 1H), 2.65 (dtd, *J* = 14.5, 11.3, 3.2 Hz, 1H), 2.16 (d, *J* = 13.7 Hz, 1H), 2.12 (d, *J* = 13.8 Hz, 1H), 2.12–2.07 (m, 1H), 1.83–1.70 (m, 4H), 1.67 (d, *J* = 6.6 Hz, 3H), 1.64 (s, 3H), 1.48 (s, 3H), 1.45–1.38 (m, 1H), 1.37 (s, 3H), 1.35–1.29 (m, 1H), 1.13 (qt, *J* = 13.0, 4.0 Hz, 1H), 1.00 (qd, *J* = 11.8, 2.9 Hz, 1H), 0.93–0.83 (m, 1H). **^{13}C NMR** (101 MHz, CDCl$_3$) δ 235.1, 163.3 (dd, *J* = 249.3, 13.0 Hz), 150.0 (t, *J* = 8.4 Hz), 109.7 (dd, *J* = 26.1, 7.2 Hz), 102.9 (t, *J* = 25.4 Hz), 78.6, 60.4, 60.2, 51.5, 45.6, 31.5, 30.5, 29.2, 28.1, 27.0, 26.1, 25.9, 25.8, 23.6. **^{19}F NMR** (CDCl$_3$, 376 MHz) δ -108.10 (t, *J* = 8.6 Hz). **IR** (ATR, neat) 2973, 2929, 2853, 1623, 1595, 1450, 1433, 1324, 1119, 986, 853, 694. **HRMS** (ESI/QTOF) m/z: [M-Cl+CH$_3$CN]$^+$ calculated for $C_{23}H_{32}CuF_2N_2^+$ 437.1824; found 437.1832. **[α]$_D^{21}$** = -23.33 (c = 0.14, CHCl$_3$). **mp** 155.0–158.6 °C (*decomp.*). **R$_f$** = 0.42 (1:1 EtOAc/pentane, UV, CAM).

(*R*,*S*)-[Me, Cy]$^{Me, (4-OMe)Ph}$CAAC-CuCl (Cu5)

IUPAC: ((*S*)-1-((*R*)-1-cyclohexylethyl)-3-(4-methoxyphenyl)-3,5,5-trimethylpyrrolidin-2-ylidene)copper(I) chloride

Purification conditions: SiliaFlash 40A, conditioning: 5:95 EtOAc/pentane, eluent: 20:80 to 25:75 EtOAc/pentane.

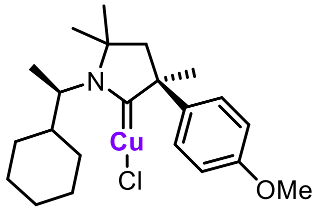

Following Method B using CAAC precursor **8e** (40.4 mg, 97.3 μmol) and CuCl(SMe$_2$) (18.8 mg, 117 μmol) afforded the corresponding copper complex (*R*,*S*)-(**Cu5**) (14.8 mg, 34.7 μmol, 36 %) as a white solid after purification by column chromatography. **^{1}H NMR** (400 MHz, CDCl$_3$) δ 7.20–7.16 (m, 2H), 6.88–6.84 (m, 2H), 3.79 (s, 3H), 3.26 (dq, *J* = 10.2, 6.5 Hz, 1H), 2.77–2.67 (m, 1H), 2.22 (d, *J* = 13.5 Hz, 1H), 2.10–2.05 (m, 1H), 2.03 (d, *J* = 13.5 Hz, 1H), 1.83–1.77 (m, 1H), 1.75–1.70 (m, 3H), 1.68 (d, *J* = 6.5 Hz, 3H), 1.67 (s, 3H), 1.41 (s, 3H), 1.44–1.37 (m, 1H), 1.34 (s, 3H), 1.32–1.26 (m, 1H), 1.18–1.06 (m, 1H), 1.03–0.93 (m, 1H), 0.81 (qd, *J* = 12.3, 3.3 Hz, 1H). **^{13}C NMR** (101 MHz, CDCl$_3$) δ 237.3, 158.5, 138.2, 127.4, 114.4, 78.4, 59.7, 59.6, 55.4, 51.7, 45.1, 30.9, 30.7, 28.8, 28.2, 28.1, 26.2, 26.0, 26.0, 23.9. **IR** (ATR, neat) 2928, 2852, 1609, 1549, 1511, 1451, 1376, 1252, 1182, 1031, 829. **HRMS** (APCI/QTOF) m/z: [M-Cl+CH$_3$CN]$^+$ calculated for $C_{24}H_{36}CuN_2O^+$ 431.2118; found 431.2111.**[α]$_D^{21}$** = -145.0 (c = 0.10, CHCl$_3$). **mp** 192.6–196.1 °C (*decomp.*). **R*$_f$*** = 0.58 (1:1 EtOAc/pentane, UV, slight stain in CAM, KMnO$_4$).

(*R*,*R*)-[Me, Cy]$^{iPr, (4-OMe)Ph}$CAAC-CuCl (Cu5)

IUPAC: ((*R*)-1-((*R*)-1-cyclohexylethyl)-3-(4-methoxyphenyl)-3,5,5-trimethylpyrrolidin-2-ylidene)copper(I) chloride

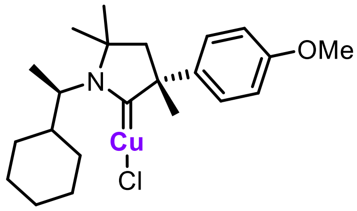

Following Method B using CAAC precursor **8e** (40.4 mg, 97.3 μmol) and CuCl(SMe$_2$) (18.8 mg, 117 μmol) afforded the corresponding copper complex (*R*,*R*)-(**Cu5**) (10.1 mg, 23.5 μmol, 24 %) as a white solid after purification by column chromatography. **^{1}H NMR** (400 MHz, CDCl$_3$) δ 7.26–7.22 (m, 2H), 6.89–6.85 (m, 2H), 3.80 (s, 3H), 3.27 (dq, *J* = 9.9, 6.6 Hz, 1H), 2.69–2.60 (m, 1H), 2.24 (d, *J* = 13.5 Hz, 1H), 2.12–2.05 (m, 1H), 2.08 (d, *J* = 13.6 Hz, 1H), 1.82–1.77 (m, 1H), 1.74–1.68 (m, 3H), 1.66 (d, *J* = 6.6 Hz, 3H), 1.63 (s, 3H), 1.46 (s, 3H), 1.42–1.37 (m, 1H), 1.33 (s, 3H), 1.36–1.29 (m, 1H), 1.17–1.06 (m, 1H), 1.03–0.93 (m, 1H), 0.87–0.77 (m, 1H). **^{13}C NMR** (101 MHz, CDCl$_3$) δ 236.9, 158.5, 138.0, 127.5, 114.4, 78.3, 60.0, 59.8, 55.4, 51.4, 45.5, 31.3, 30.7, 29.4, 27.9, 26.2, 26.0, 25.9, 23.6. **IR** (ATR, neat) 2928, 2852, 1609, 1548, 1511, 1450, 1377, 1252, 1182, 1031, 829. (1C not resolved). **HRMS** (APCI/QTOF) m/z: [M-Cl+CH$_3$CN]$^+$ calculated for $C_{24}H_{36}CuN_2O^+$ 431.2118; found 431.2111. **[α]$_D^{21}$** = -25.49 (c = 0.17, CHCl$_3$). **mp** 185.9–189.7 °C (*decomp.*). **R*$_f$*** = 0.47 (1:1 EtOAc/pentane, UV, slight stain in CAM, KMnO$_4$).

(*R*,*S*)-[Me, Cy]$^{Me, 3,5\text{-di}t\text{BuPh}}$CAAC-CuCl (Cu6)

IUPAC: ((*S*)-1-((*R*)-1-cyclohexylethyl)-3-(3,5-di-tert-butylphenyl)-3,5,5-trimethylpyrrolidin-2-ylidene)copper(I) chloride

Purification conditions: SiliaFlash 40A, conditioning: 5:95 EtOAc/pentane, eluent: 1:9 then 2:8 then 3:7 EtOAc/pentane.

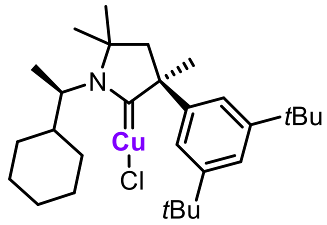

Following Method B using CAAC precursor **8f** (26.7 mg, 53.7 μmol) and CuCl(SMe_2) (18.8 mg, 117 μmol) afforded the corresponding copper complex (*R*,*S*)-(**Cu6**) (5.3 mg, 10.4 μmol, 19 %) as a white solid after purification by column chromatography. **^{1}H NMR** (400 MHz, $CDCl_3$) δ 7.28 (t, *J* = 1.8 Hz, 1H), 7.10 (d, *J* = 1.8 Hz, 2H), 3.28 (dq, *J* = 10.1, 6.5 Hz, 1H), 2.83–2.73 (m, 1H), 2.27 (d, *J* = 13.4 Hz, 1H), 2.11–2.07 (m, 1H), 2.03 (d, *J* = 13.4 Hz, 1H), 1.82–1.77 (m, 2H), 1.72 (d, *J* = 6.5 Hz, 3H), 1.71 (s, 3H), 1.73–1.70 (m, 1H), 1.48–1.43 (m, 1H), 1.41 (s, 3H), 1.40–1.35 (m, 2H), 1.33 (s, 3H), 1.31 (s, 18H), 1.18–1.07 (m, 1H), 1.04–0.94 (m, 1H), 0.86–0.76 (m, 1H). **^{13}C NMR** (101 MHz, $CDCl_3$) δ 237.9, 151.1, 145.5, 121.0, 120.5, 78.4, 60.4, 59.7, 51.7, 44.9, 35.2, 31.7, 30.7, 28.6, 28.4, 28.2, 26.3, 26.0, 26.0, 23.9. **IR** (ATR, neat) 2962, 2927, 2854, 1596, 1549, 1450, 1363, 1248, 872, 714. **HRMS** (ESI/QTOF) m/z: [M-Cl+CH_3CN]$^+$ calculated for $C_{31}H_{50}CuN_2^+$ 513.3264; found 513.3263. **[α]$_D$^{21}** = -73.33 (c = 0.10, $CHCl_3$). **mp** 153.7–157.8 °C (*decomp.*). **R*$_f$*** = 0.56 (2:8 EtOAc/pentane, UV, CAM).

(*R*,*R*)-[Me, Cy]$^{Me, 3,5\text{-di}t\text{BuPh}}$CAAC-CuCl (Cu6)

IUPAC: ((*R*)-1-((*R*)-1-cyclohexylethyl)-3-(3,5-di-tert-butylphenyl)-3,5,5-trimethylpyrrolidin-2-ylidene)copper(I) chloride

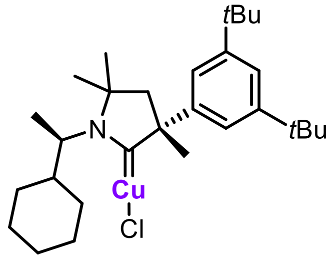

Following Method B using CAAC precursor **8f** (26.7 mg, 53.7 μmol) and CuCl(SMe_2) (18.8 mg, 117 μmol) afforded the corresponding copper complex (*R*,*R*)-(**Cu6**) (5.90 mg, 11.6 μmol, 22 %) as a white solid after purification by column chromatography. **^{1}H NMR** (400 MHz, $CDCl_3$) δ 7.30 (t, *J* = 1.8 Hz, 1H), 7.16 (d, *J* = 1.7 Hz, 2H), 3.29 (dq, *J* = 10.1, 6.5 Hz, 1H), 2.77–2.67 (m, 1H), 2.26 (d, *J* = 13.6 Hz, 1H), 2.11 (d, *J* = 13.5 Hz, 1H), 2.11–2.07 (m, 1H), 1.86–1.78 (m, 2H), 1.72–1.69(m, 2H), 1.69–1.66 (m, 6H), 1.47 (s, 3H), 1.44–1.39 (m, 1H), 1.35 (s, 3H), 1.32 (s, 18H), 1.32–1.28 (m, 1H), 1.17–1.07 (m, 1H), 1.03–0.93 (m, 1H), 0.89–0.79 (m, 1H). **^{13}C NMR** (101 MHz, $CDCl_3$) δ 236.9, 151.2, 145.4, 121.1, 120.6, 78.4, 61.0, 59.8, 51.8, 45.6, 35.2, 31.6, 31.2, 30.7, 28.0, 27.6, 26.2, 26.1, 26.0, 23.6. **IR** (ATR, neat) 2962, 2927, 2855, 1596, 1548, 1450, 1363, 1248, 872, 714.**HRMS** (ESI/QTOF) m/z: [M-Cl+CH_3CN]$^+$ calculated for $C_{31}H_{50}CuN_2^+$ 513.3264; found 513.3263. **[α]$_D$^{21}** = -9.72 (c = 0.12, $CHCl_3$). **mp** 181.2–186.9 °C (*decomp.*). **R*$_f$*** = 0.27 (2:8 EtOAc/pentane, UV, CAM).

($\boldsymbol{R,R}$)-[Me, Cy]$^{iPr, Ph}$CAAC-CuCl (Cu7)

IUPAC: ((*R*)-1-((*R*)-1-cyclohexylethyl)-3-isopropyl-5,5-dimethyl-3-phenylpyrrolidin-2-ylidene)copper(I) chloride

Purification conditions: SiliaFlash 40A, conditioning: 5:95 EtOAc/pentane, eluent: 1:9 then 2:8 EtOAc/pentane.

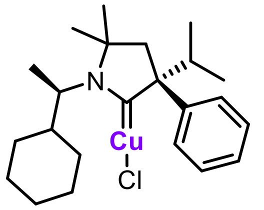

Following Method B using CAAC precursor **8g** (43.2 mg, 102 μmol) and CuCl(SMe$_2$) (19.7 mg, 122 μmol) afforded the corresponding copper complex (*R,R*)-(**Cu7**) (11.7 mg, 27.5 μmol, 27 %) as a white solid after purification by column chromatography. **^{1}H NMR** (400 MHz, CDCl$_3$) δ 7.70 (d, *J* = 7.9 Hz, 2H), 7.34 (t, *J* = 7.6 Hz, 2H), 7.22 (t, *J* = 7.4 Hz, 1H), 3.25 (dq, *J* = 10.0, 6.6 Hz, 1H), 2.78–2.69 (m, 1H), 2.69–2.60 (m, 1H), 2.44 (d, *J* = 13.8 Hz, 1H), 2.09–2.02 (m, 1H), 1.99 (d, *J* = 13.8 Hz, 1H), 1.83–1.77 (m, 1H), 1.76–1.68 (m, 3H), 1.50 (d, *J* = 6.6 Hz, 3H), 1.43 (s, 3H), 1.43–1.27 (m, 2H), 1.17–1.10 (m, 1H), 1.12 (s, 3H), 1.07 (d, *J* = 6.8 Hz, 3H), 1.01–0.91 (m, 1H), 0.89–0.83 (m, 1H), 0.78 (d, *J* = 6.8 Hz, 3H). **^{13}C NMR** (101 MHz, CDCl$_3$) δ 239.4, 142.8, 128.7, 128.0, 126.9, 76.9, 68.4, 59.9, 45.2, 40.3, 38.2, 31.4, 30.5, 28.0, 27.3, 26.2, 26.0, 25.9, 23.8, 19.8, 18.4. **IR** (ATR, neat) 2927, 2853, 1546, 1466, 1447, 1376, 1127, 917, 763, 731, 701. **HRMS** (ESI/QTOF) m/z: [M-Cl+CH$_3$CN]$^+$ calculated for $C_{25}H_{38}CuN_2^+$ 429.2325; found 429.2319. **[α]$_D^{21}$** = -90.00 (c = 0.10, CHCl$_3$). **mp** 242.6–246.4 °C (*decomp.*). **R$_f$** = 0.22 (2:8 EtOAc/pentane, UV, CAM, KMnO$_4$).

($\boldsymbol{R,S}$)-[Me, Cy]$^{iPr, Ph}$CAAC-CuCl (Cu7)

IUPAC: ((*S*)-1-((*R*)-1-cyclohexylethyl)-3-isopropyl-5,5-dimethyl-3-phenylpyrrolidin-2-ylidene)copper(I) chloride

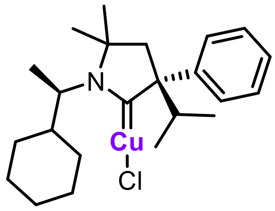

Following Method B using CAAC precursor **8g** (43.2 mg, 102 μmol) and CuCl(SMe$_2$) (19.7 mg, 122 μmol) afforded the corresponding copper complex (*R,S*)-(**Cu7**) (10.1 mg, 23.8 μmol, 23 %) as a white solid after purification by column chromatography. **^{1}H NMR** (400 MHz, CDCl$_3$) δ 7.73 (d, *J* = 7.9 Hz, 2H), 7.33 (t, *J* = 7.6 Hz, 2H), 7.21 (t, *J* = 7.4 Hz, 1H), 3.15–3.08 (m, 1H), 2.82–2.73 (m, 1H), 2.71–2.61 (m, 1H), 2.49 (d, *J* = 14.0 Hz, 1H), 2.04–1.97 (m, 1H), 1.99 (d, *J* = 13.9 Hz, 1H), 1.76–1.69 (m, 1H), 1.63 (d, *J* = 6.5 Hz, 3H), 1.61–1.58 (m, 1H), 1.47 (s, 3H), 1.51–1.43 (m, 1H), 1.38–1.29 (m, 1H), 1.14 (s, 3H), 1.16–1.08 (m, 2H), 1.02 (d, *J* = 6.8 Hz, 3H), 1.06–0.96 (m, 1H), 0.92–0.82 (m, 1H), 0.73 (d, *J* = 6.8 Hz, 3H), 0.49–0.39 (m, 1H). **^{13}C NMR** (101 MHz, CDCl$_3$) δ 238.8, 142.1, 128.6, 128.0, 127.0, 76.9, 68.4, 60.0, 45.9, 39.2, 38.1, 30.8, 30.5, 29.1, 26.1, 26.0, 26.0, 25.6, 23.2, 19.2, 18.0. **IR** (ATR, neat) 2930, 2853, 1551, 1467, 1449, 1376, 1126, 917, 763, 733, 701. **HRMS** (ESI/QTOF) m/z: [M-Cl+CH$_3$CN]$^+$ calculated for $C_{25}H_{38}CuN_2^+$ 429.2325; found 429.2326. **[α]$_D^{21}$** = -38.33 (c = 0.17, CHCl$_3$). **mp** 158.6–160.9 °C (*decomp.*). **R$_f$** = 0.16 (2:8 EtOAc/pentane, UV, CAM, KMnO$_4$).

(*R*,*S*)-[Me, Cy]^{THN}CAAC-CuCl (Cu8)

IUPAC: ((*S*)-1'-((*R*)-1-cyclohexylethyl)-5',5'-dimethyl-3,4-dihydro-2H-spiro[naphthalene-1,3'-pyrrolidin]-2'-ylidene)copper(I) chloride

Purification conditions: SiliaFlash 40A, conditioning: 5:95 EtOAc/pentane, eluent: 1:9 then 2:8 EtOAc/pentane.

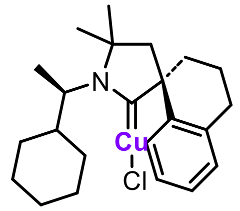

Following Method B using CAAC precursor **8h** (43.3 mg, 105 μmol) and CuCl(SMe$_2$) (20.3 mg, 126 μmol) afforded the corresponding copper complex (*R,S*)-(**Cu8**) (9.58 mg, 22.6 μmol, 22 %) as a white solid after purification by column chromatography. **^{1}H NMR** (400 MHz, CDCl$_3$) δ 7.16–7.09 (m, 3H), 6.58–6.56 (m, 1H), 3.26 (dq, *J* = 10.2, 6.5 Hz, 1H), 2.95–2.70 (m, 3H), 2.33 (td, *J* = 12.8, 3.0 Hz, 1H), 2.25 (d, *J* = 13.7 Hz, 1H), 2.13–2.06 (m, 2H), 2.02 (d, *J* = 13.7 Hz, 1H), 1.87–1.78 (m, 2H), 1.74 (d, *J* = 6.5 Hz, 3H), 1.72–1.67 (m, 2H), 1.63–1.57 (m, 1H), 1.49 (s, 3H), 1.48–1.43 (m, 1H), 1.42 (s, 3H), 1.40–1.27 (m, 2H), 1.17–1.06 (m, 1H), 1.03–0.93 (m, 1H), 0.83–0.73 (m, 1H). **^{13}C NMR** (101 MHz, CDCl$_3$) δ 236.1, 140.3, 136.0, 129.9, 128.7, 127.1, 126.8, 77.9, 61.4, 59.9, 52.3, 45.0, 34.8, 31.0, 30.6, 29.8, 29.2, 28.7, 26.2, 26.0, 26.0, 23.8, 19.5. **HRMS** (ESI/QTOF) m/z: $[M-Cl+CH_3CN]^+$ calculated for $C_{25}H_{36}CuN_2^+$ 427.2169; found 427.2174. **$[\alpha]_D^{21}$** = -121.67 (c = 0.10, CHCl$_3$). **mp** >230 °C (*decomp.*). **R_f** = 0.70 (1:1 EtOAc/pentane, UV, CAM).

(*R*,*R*)-[Me, Cy]^{THN}CAAC-CuCl (Cu8)

IUPAC: ((*R*)-1'-((*R*)-1-cyclohexylethyl)-5',5'-dimethyl-3,4-dihydro-2H-spiro[naphthalene-1,3'-pyrrolidin]-2'-ylidene)copper(I) chloride

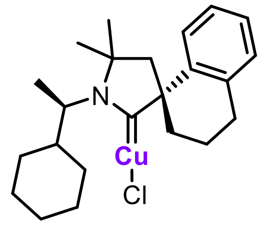

Following Method B using CAAC precursor **8h** (43.3 mg, 105 μmol) and CuCl(SMe$_2$) (20.3 mg, 126 μmol) afforded the corresponding copper complex (*R,R*)-(**Cu8**) (9.69 mg, 22.6 μmol, 22 %) as a white solid after purification by column chromatography. **^{1}H NMR** (400 MHz, CDCl$_3$) δ 7.16–7.09 (m, 3H), 6.63–6.61 (m, 1H), 3.33 (dq, *J* = 10.0, 6.6 Hz, 1H), 2.91 (ddd, *J* = 16.6, 10.8, 5.6 Hz, 1H), 2.84–2.78 (m, 1H), 2.71–2.63 (m, 1H), 2.29 (d, *J* = 13.7 Hz, 1H), 2.30–2.24 (m, 1H), 2.17–2.09 (m, 2H), 2.00 (d, *J* = 13.6 Hz, 1H), 1.95–1.89 (m, 1H), 1.87–1.77 (m, 3H), 1.75–1.71 (m, 1H), 1.66 (d, *J* = 6.6 Hz, 3H), 1.61–1.56 (m, 1H), 1.46 (s, 3H), 1.46 (s, 3H), 1.42–1.34 (m, 2H), 1.21–1.10 (m, 1H), 1.06–0.91 (m, 2H). **^{13}C NMR** (101 MHz, CDCl$_3$) δ 236.3, 140.2, 136.4, 129.9, 128.9, 127.1, 126.9, 77.8, 61.8, 59.9, 52.3, 45.2, 34.5, 31.5, 30.6, 29.9, 29.4, 28.8, 26.2, 26.0, 26.0, 23.9, 19.7. **HRMS** (ESI/QTOF) m/z: $[M-Cl+CH_3CN]^+$ calculated for $C_{25}H_{36}CuN_2^+$ 427.2169; found 427.2169. **$[\alpha]_D^{21}$** = -53.33 (c = 0.10, CHCl$_3$). **mp** 267.7–269.3 °C (*decomp.*). **R_f** = 0.62 (1:1 EtOAc/pentane, UV, CAM).

(*R*,*S*)-[Me, Cy]$^{Me, Cy}$CAAC-CuCl (Cu9)

IUPAC: ((*S*)-3-cyclohexyl-1-((*R*)-1-cyclohexylethyl)-3,5,5-trimethylpyrrolidin-2-ylidene)copper(I) chloride

Purification conditions: SiliaFlash 40A, conditioning: 5:95 EtOAc/pentane, eluent: 1:9 then 2:8

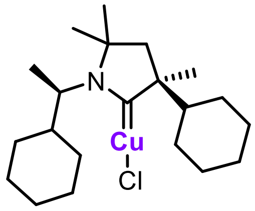

Following Method B using CAAC precursor **8i** (30.6 mg, 78.2 µmol) and CuCl(SMe$_2$) (15.1 mg, 93.8 µmol) afforded the corresponding copper complex (*R*,*S*)-(**Cu9**) (12.7 mg, 31.5 µmol, 40 %) as a white solid after purification by column chromatography. **^{1}H NMR** (400 MHz, CDCl$_3$) δ 3.10 (dq, *J* = 10.4, 6.5 Hz, 1H), 2.69–2.61 (m, 1H), 2.06–1.98 (m, 2H), 1.91 (d, *J* = 13.5 Hz, 1H), 1.87–1.84 (m, 1H), 1.80–1.74 (m, 2H), 1.72–1.65 (m, 5H), 1.54 (d, *J* = 6.5 Hz, 3H), 1.48 (d, *J* = 13.7 Hz, 1H), 1.46–1.41 (m, 2H), 1.37 (s, 3H), 1.35–1.32 (m, 1H), 1.33 (s, 3H), 1.29–1.24 (m, 2H), 1.22 (s, 3H), 1.14–1.04 (m, 2H), 0.99–0.88 (m, 2H), 0.85–0.68 (m, 2H). **^{13}C NMR** (101 MHz, CDCl$_3$) δ 240.0, 77.9, 60.8, 59.5, 46.7, 45.2, 42.8, 31.0, 30.5, 30.3, 29.1, 28.5, 27.2, 26.8, 26.5, 26.4, 26.2, 26.2, 26.0, 26.0, 23.5. **IR** (ATR, neat) 2972, 2923, 2851, 1548, 1449, 1376, 1170, 753. **HRMS** (nanochip-ESI/LTQ-Orbitrap) m/z: [M-Cl+CH$_3$CN]$^+$ calculated for $C_{23}H_{40}CuN_2^+$ 407.2482; found 407.2472.**[α]$_D^{21}$** = -76.67 (c = 0.10, CHCl$_3$). **mp** >201 °C (*decomp.*). **R$_f$** = 0.83 (1:1 EtOAc/pentane, UV, slight stain in CAM).

(*R*,*R*)-[Me, Cy]$^{Me, Cy}$CAAC-CuCl (Cu9)

IUPAC: ((*R*)-3-cyclohexyl-1-((*R*)-1-cyclohexylethyl)-3,5,5-trimethylpyrrolidin-2-ylidene)copper(I) chloride

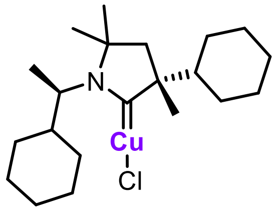

Following Method B using CAAC precursor **8i** (30.6 mg, 78.2 µmol) and CuCl(SMe$_2$) (15.1 mg, 93.8 µmol) afforded the corresponding copper complex (*R*,*R*)-(**Cu9**) (10.1 mg, 25.1 µmol, 32 %) as a white solid after purification by column chromatography. **^{1}H NMR** (400 MHz, CDCl$_3$) δ 3.17 (dq, *J* = 10.1, 6.6 Hz, 1H), 2.58–2.48 (m, 1H), 2.06–1.94 (m, 2H), 1.90–1.85 (m, 1H), 1.87 (d, *J* = 13.4 Hz, 1H), 1.80–1.74 (m, 2H), 1.71–1.65 (m, 4H), 1.58–1.55 (m, 1H), 1.57 (d, *J* = 6.6 Hz, 3H), 1.51 (d, *J* = 13.5 Hz, 1H), 1.50–1.45 (m, 1H), 1.42–1.38 (m, 1H), 1.37 (s, 3H), 1.34 (s, 3H), 1.32–1.22 (m, 3H), 1.19 (s, 3H), 1.16–1.07 (m, 2H), 1.02–0.89 (m, 2H), 0.86–0.73 (m, 2H). **^{13}C NMR** (101 MHz, CDCl$_3$) δ 239.8, 77.8, 61.1, 59.4, 46.7, 45.2, 42.7, 31.3, 30.5, 30.4, 29.5, 28.3, 27.4, 26.7, 26.5, 26.4, 26.2, 26.0, 25.9, 25.8, 23.7. **IR** (ATR, neat) 2972, 2923, 2852, 1548, 1449, 1376, 1169, 752. **HRMS** (nanochip-ESI/LTQ-Orbitrap) m/z: [M-Cl+CH$_3$CN]$^+$ calculated for $C_{23}H_{40}CuN_2^+$ 407.2482; found 407.2470. **[α]$_D^{21}$** = -58.33 (c = 0.10, CHCl$_3$). **mp** >181 °C (*decomp.*). **R$_f$** = 0.74 (1:1 EtOAc/pentane, UV, slight stain in CAM).

(*R*,*S*)-[Me, Cy]$^{Me, Ad}$CAAC-CuCl (Cu10)

IUPAC: ((*S*)-3-(adamantan-1-yl)-1-((*R*)-1-cyclohexylethyl)-3,5,5-trimethylpyrrolidin-2-ylidene)copper(I) chloride

Purification conditions: SiliaFlash 40A, conditioning: 5:95 EtOAc/pentane, eluent: 1:9 then 2:8

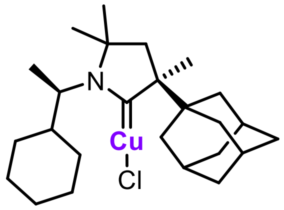

Following Method B using CAAC precursor **8j** (31.7 mg, 71.5 µmol) and CuCl(SMe$_2$) (13.8 mg, 85.9 µmol) afforded the corresponding copper complex (*R*,*S*)-(**Cu10**) (9.58 mg, 21.0 µmol, 29 %) as a white solid after purification by column chromatography. ^{1}**H NMR** (400 MHz, CDCl$_3$) δ 3.19–3.11 (m, 1H), 2.83–2.75 (m, 1H), 2.11 (d, *J* = 13.5 Hz, 1H), 2.13–2.08 (m, 2H), 2.07–2.04 (m, 4H), 1.81–1.78 (m, 1H), 1.74–1.70 (m, 7H), 1.68–1.62 (m, 5H), 1.60 (d, *J* = 6.5 Hz, 3H), 1.51–1.45 (m, 2H), 1.43 (s, 3H), 1.44–1.39 (m, 1H), 1.37 (s, 3H), 1.35–1.28 (m, 2H), 1.14 (s, 3H), 1.01–0.90 (m, 1H), 0.79–0.68 (m, 1H). ^{13}**C NMR** (101 MHz, CDCl$_3$) δ 239.4, 76.4, 64.1, 59.8, 45.6, 43.3, 38.6, 38.2, 37.1, 31.1, 30.5, 29.8, 28.7, 28.3, 26.3, 26.0, 26.0, 23.5, 21.1. **IR** (ATR, neat) 2970, 2904, 2849, 1667, 1544, 1449, 1375, 1344, 1309, 500. **HRMS** (ESI/QTOF) m/z: [M-Cl+CH$_3$CN]$^+$ calculated for $C_{27}H_{44}CuN_2^+$ 459.2795; found 459.2801. **[α]$_D^{21}$** = -28.33 (c = 0.10, CHCl$_3$). **mp** 205.1–209.5 °C (*decomp.*). **R$_f$** = 0.31 (2:8 EtOAc/pentane, UV, CAM).

(*R*,*R*)-[Me, Cy]$^{Me, Ad}$CAAC-CuCl (Cu10)

IUPAC: ((*R*)-3-(adamantan-1-yl)-1-((*R*)-1-cyclohexylethyl)-3,5,5-trimethylpyrrolidin-2-ylidene)copper(I) chloride

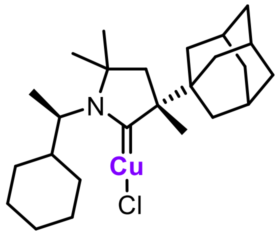

Following Method B using CAAC precursor **8j** (31.7 mg, 71.5 µmol) and CuCl(SMe$_2$) (13.8 mg, 85.9 µmol) afforded the corresponding copper complex (*R*,*R*)-(**Cu10**) (9.38 mg, 20.6 µmol, 29 %) as a white solid after purification by column chromatography. ^{1}**H NMR** (400 MHz, CDCl$_3$) δ 3.20 (dq, *J* = 10.0, 6.6 Hz, 1H), 2.65 (q, *J* = 11.2 Hz, 1H), 2.10–2.02 (m, 7H), 1.79–1.76 (m, 1H), 1.71–1.64 (m, 9H), 1.60 (d, *J* = 6.6 Hz, 3H), 1.62–1.58 (m, 3H), δ 1.48 (d, *J* = 13.4 Hz, 1H), 1.45–1.40 (m, 1H), 1.38 (s, 3H), 1.37 (s, 3H), 1.34–1.23 (m, 2H), 1.16–1.11 (m, 1H), 1.08 (s, 3H), 0.94 (ddd, *J* = 12.6, 12.6, 3.6 Hz, 1H), 0.78 (ddd, *J* = 12.8, 12.1, 3.7 Hz, 1H). ^{13}**C NMR** (101 MHz, CDCl$_3$) δ 239.1, 76.4, 64.4, 59.8, 45.1, 43.1, 38.8, 38.1, 37.1, 31.3, 30.6, 29.5, 28.8, 28.7, 26.2, 26.0, 25.9, 24.1, 20.7. **IR** (ATR, neat) 2970, 2904, 2849, 1667, 1544, 1449, 1375, 1344, 1309, 500. **HRMS** (ESI/QTOF) m/z: [M-Cl+CH$_3$CN]$^+$ calculated for $C_{27}H_{44}CuN_2^+$ 459.2795; found 459.2801. **[α]$_D^{21}$** = -66.67 (c = 0.10, CHCl$_3$). **mp** 216.5–219.2 °C (*decomp.*). **R$_f$** = 0.22 (2:8 EtOAc/pentane, UV, CAM).

(*R*,*S*)-[Me, Cy]$^{Me, (CH_2)_2SPh}$CAAC-CuCl (Cu11)

IUPAC: ((*S*)-1-((*R*)-1-cyclohexylethyl)-3,5,5-trimethyl-3-(2-(phenylthio)ethyl)pyrrolidin-2-ylidene)copper(I) chloride

Purification conditions: SiliaFlash 40A, conditioning: 5:95 EtOAc/pentane, eluent: 10:90 then 20:80 then 25:75 then 30:70 EtOAc/pentane.

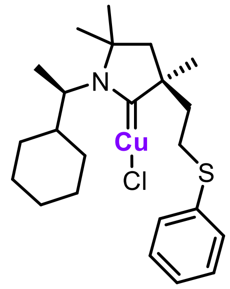

Following Method B using CAAC precursor **8k** (39.6 mg, 88.8 μmol) and CuCl(SMe$_2$) (17.4 mg, 108 μmol) afforded the corresponding copper complex (*R,S*)-(**Cu11**) (10.0 mg, 21.9 μmol, 25 %) as a white solid after purification by column chromatography. **^{1}H NMR** (800 MHz, CD$_2$Cl$_2$) δ 7.36–7.35 (m, 2H), 7.33–7.30 (m, 2H), 7.18 (tt, *J* = 7.3, 1.3 Hz, 1H), 3.18 (dq, *J* = 10.2, 6.5 Hz, 1H), 3.04 (ddd, *J* = 12.8, 10.7, 5.5 Hz, 1H), 2.90 (ddd, *J* = 12.8, 10.8, 5.8 Hz, 1H), 2.59 (qt, *J* = 11.5, 3.3 Hz, 1H), 2.07–2.04 (m, 1H), 2.04–1.97 (m, 2H), 1.88 (d, *J* = 13.5 Hz, 1H), 1.81–1.79 (m, 1H), 1.73 (d, *J* = 13.5 Hz, 1H), 1.72–1.68 (m, 2H), 1.65–1.63 (m, 1H), 1.55 (d, *J* = 6.6 Hz, 3H), 1.39–1.36 (m, 1H), 1.35 (s, 3H), 1.34 (s, 3H), 1.29 (s, 3H), 1.26–1.23 (m, 1H), 1.13 (qt, *J* = 13.0, 3.9 Hz, 1H), 0.98 (tdd, *J* = 12.8, 11.3, 3.7 Hz, 1H), 0.78 (qd, *J* = 12.5, 3.4 Hz, 1H). **^{13}C NMR** (101 MHz, CDCl$_3$) δ 238.0, 136.4, 129.4, 129.2, 126.3, 78.6, 59.7, 57.6, 46.5, 45.8, 40.8, 31.0, 30.7, 29.6, 29.5, 28.4, 27.0, 26.5, 26.4, 26.4, 23.7. **IR** (ATR, neat) 2926, 2852, 1583, 1561, 1450, 1377, 740, 692. **HRMS** (ESI/QTOF) m/z: [M-Cl+CH$_3$CN]$^+$ calculated for $C_{25}H_{38}CuN_2S^+$ 461.2046; found 461.2058. **[α]$_D$^{21}** = -56.35 (c = 0.63, CH$_2$Cl$_2$). **mp** 122.8–125.3 °C (*decomp.*). **R*$_f$*** = 0.76 (1:1 EtOAc/pentane, UV, CAM).

(*R*,*R*)-[Me, Cy]$^{Me, (CH_2)_2SPh}$CAAC-CuCl (Cu11)

IUPAC: ((*R*)-1-((*R*)-1-cyclohexylethyl)-3,5,5-trimethyl-3-(2-(phenylthio)ethyl)pyrrolidin-2-ylidene)copper(I) chloride

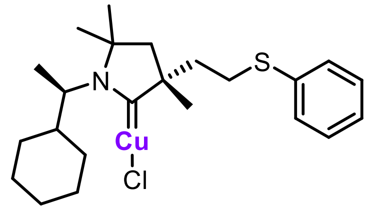

Following Method B using CAAC precursor **8k** (39.6 mg, 88.8 μmol) and CuCl(SMe$_2$) (17.4 mg, 108 μmol) afforded the corresponding copper complex (*R,R*)-(**Cu11**) (10.1 mg, 22.1 μmol, 25 %) as a white solid after purification by column chromatography. **^{1}H NMR** (800 MHz, CD$_2$Cl$_2$) δ 7.35–7.17 (m, 5H), 3.22 (dq, *J* = 10.1, 6.6 Hz, 1H), 3.06 (ddd, *J* = 12.6, 11.0, 5.3 Hz, 1H), 2.90 (ddd, *J* = 12.5, 11.0, 5.4 Hz, 1H), 2.56–2.51 (m, 1H), 2.07–2.04 (m, 1H), 2.04–1.96 (m, 2H), 1.86 (d, *J* = 13.5 Hz, 1H), 1.82–1.78 (m, 1H), 1.75 (d, *J* = 13.4 Hz, 1H), 1.71–1.67 (m, 2H), 1.62–1.59 (m, 1H), 1.58 (d, *J* = 6.6 Hz, 3H), 1.38 (s, 3H), 1.37–1.33 (m, 1H), 1.31 (s, 3H), 1.27 (s, 3H), 1.26–1.20 (m, 1H), 1.16–1.10 (m, 1H), 1.01–0.96 (m, 1H), 0.82–0.77 (m, 1H). **^{13}C NMR** (201 MHz, CD$_2$Cl$_2$) δ 238.0, 136.5, 129.4, 129.4, 129.2, 129.2, 126.4, 78.6, 59.6, 57.6, 46.6, 45.8, 40.9, 31.2, 30.7, 29.7, 29.7, 28.4, 26.5, 26.4, 26.4, 26.4, 23.8. **IR** (ATR, neat) 2926, 2852, 1579, 1560, 1449, 1377, 740, 692. **HRMS** (ESI/QTOF) m/z: [M-Cl+CH$_3$CN]$^+$ calculated for $C_{25}H_{38}CuN_2S^+$ 461.2046; found 461.2059. **[α]$_D$^{21}** = -28.29 (c = 0.76, CH$_2$Cl$_2$). **mp** 89.4–91.9 °C (*decomp.*). **R*$_f$*** = 0.64 (1:1 EtOAc/pentane, UV, CAM).

Gold complexes

(*R*,*S*)-[Me, Cy]$^{Me, Ph}$CAAC-AuCl (Au1)

IUPAC: ((*S*)-1-((*R*)-1-cyclohexylethyl)-3,5,5-trimethyl-3-phenylpyrrolidin-2-ylidene)gold(I) chloride

Purification conditions: SiliaFlash 40A, conditioning: 5:95 EtOAc/pentane, eluent: 5:95 then 10:90 then 25:75 EtOAc/pentane.

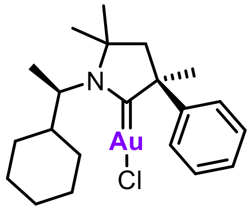

Following Method B using CAAC precursor **8a** (29.2 mg, 75.7 µmol) and $AuCl(SMe_2)$ (26.8 mg, 90.9 µmol) afforded the corresponding gold complex (*R*,*S*)-(**Au1**) (12.4 mg, 23.3 µmol, 31 %) as a white solid after purification by column chromatography. **^{1}H NMR** (400 MHz, $CDCl_3$) δ 7.35–7.30 (m, 2H), 7.28–7.21 (m, 3H), 3.32 (dq, *J* = 10.1, 6.5 Hz, 1H), 3.21–3.11 (m, 1H), 2.31 (d, *J* = 13.5 Hz, 1H), 2.12 (d, *J* = 13.5 Hz, 1H), 2.08–2.03 (m, 1H), 1.82 (d, *J* = 6.5 Hz, 3H), 1.78 (s, 3H), 1.75–1.69 (m, 3H), 1.45 (s, 3H), 1.51–1.40 (m, 1H), 1.35 (s, 3H), 1.38–1.19 (m, 2H), 1.19–1.06 (m, 1H), 1.03–0.93 (m, 1H), 0.83–0.74 (m, 1H). **^{13}C NMR** (101 MHz, $CDCl_3$) δ 225.8, 146.0, 129.0, 129.0, 127.2, 126.4, 126.4, 78.2, 60.8, 60.6, 51.9, 43.9, 31.4, 30.6, 28.5, 28.4, 28.4, 26.2, 26.0, 26.0, 22.4. **IR** (ATR, neat) 2974, 2930, 2853, 1559, 1496, 1449, 1377, 915, 731, 700. **HRMS** (ESI+APCI) m/z: $[M\text{-}Cl+CH_3CN]^+$ calculated for $C_{23}H_{34}AuN_2^+$ 535.2382; found 535.2379. **$[\alpha]_D^{21}$** = -91.03 (c = 0.13, $CHCl_3$). **mp** 222.1-224.6 °C (*decomp.*). **R*f*** = 0.68 (1:1 EtOAc/pentane, UV, slight stain in CAM).

(*R*,*R*)-[Me, Cy]$^{Me, Ph}$CAAC-AuCl (Au1)

IUPAC: ((*R*)-1-((*R*)-1-cyclohexylethyl)-3,5,5-trimethyl-3-phenylpyrrolidin-2-ylidene)gold(I) chloride

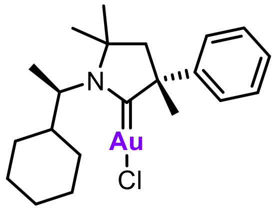

Following Method B using CAAC precursor **8a** (29.2 mg, 75.7 µmol) and $AuCl(SMe_2)$ (26.8 mg, 90.9 µmol) afforded the corresponding gold complex (*R*,*R*)-(**Au1**) (11.9 mg, 22.4 µmol, 30 %) as a white solid after purification by column chromatography. **^{1}H NMR** (400 MHz, CD_2Cl_2) δ 7.36–7.30 (m, 4H), 7.28–7.22 (m, 1H), 3.36–3.29 (m, 1H), 3.14–3.06 (m, 1H), 2.32 (d, *J* = 13.6 Hz, 1H), 2.18 (d, *J* = 13.7 Hz, 1H), 2.08–2.03 (m, 1H), 1.83–1.79 (m, 1H), 1.77 (d, *J* = 6.6 Hz, 3H), 1.75 (s, 3H), 1.74–1.67 (m, 2H), 1.49 (s, 3H), 1.42–1.36 (m, 1H), 1.37 (s, 3H), 1.35–1.25 (m, 2H), 1.17–1.06 (m, 1H), 1.01–0.91 (m, 1H), 0.87–0.77 (m, 1H). **^{13}C NMR** (101 MHz, $CDCl_3$) δ 225.3, 145.6, 129.0, 129.0, 127.3, 126.6, 126.6, 78.0, 61.3, 60.9, 51.7, 44.1, 31.2, 31.1, 29.2, 28.0, 27.9, 26.2, 25.9, 25.9, 22.1. **IR** (ATR, neat) 2974, 2927, 2853, 1557, 1495, 1448, 1377, 914, 730, 699. **HRMS** (ESI+APCI) m/z: $[M\text{-}Cl+CH_3CN]^+$ calculated for $C_{23}H_{34}AuN_2^+$ 535.2382; found 535.2379. **$[\alpha]_D^{21}$** = -27.78 (c = 0.12, $CHCl_3$). **mp** 248.6-250.5 °C (*decomp.*). **R*f*** = 0.57 (1:1 EtOAc/pentane, UV, slight stain in CAM).

Rhodium complexes

(*R*,*S*)-[Me,Cy]$^{Me, Ph}$CAC-Rh(COD)Cl (Rh1)

IUPAC: ((*S*)-1-((*R*)-1-cyclohexylethyl)-3,5,5-trimethyl-3-phenylpyrrolidin-2-ylidene)-(1,5-cyclooctadiene)rhodium(I) chloride

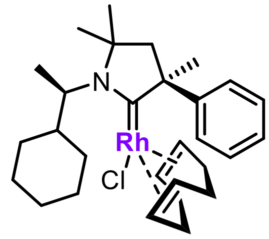

Following Method A using CAAC precursor **8a** (26.6 mg, 68.9 µmol) and $[Rh(COD)Cl]_2$ (15.4 mg, 31.2 µmol) afforded the corresponding rhodium complex (*R*,*S*)-(**Rh1**) (11.7 mg, 21.4 µmol, 31 %) as a yellow solid after purification by column chromatography. **^{1}H NMR** (400 MHz, CD_2Cl_2) δ 7.30–7.24 (m, 2H), 7.21–7.17 (m, 1H), 7.07–7.03 (m, 2H), 5.04–5.00 (m, 1H), 4.85 (br s, 1H), 4.76–4.71 (m, 1H), 3.36–3.31 (m, 1H), 3.12 (br s, 1H), 2.82–2.77 (m, 1H), 2.37–2.21 (m, 4H), 2.16 (s, 3H), 2.12 (d, *J* = 12.8 Hz, 1H), 2.09–2.05 (m, 2H), 1.97 (d, *J* = 7.0 Hz, 1H), 1.94–1.86 (m, 2H), 1.79–1.73 (m, 3H), 1.71–1.68 (m, 1H), 1.70 (d, *J* = 12.8 Hz, 3H), 1.67–1.64 (m, 1H), 1.52–1.45 (m, 1H), 1.44 (s, 3H), 1.41–1.28 (m, 2H), 1.20 (s, 3H), 1.18–1.07 (m, 1H), 1.07–0.98 (m, 1H). **^{13}C NMR** (101 MHz, CD_2Cl_2) δ 262.5, 148.2, 128.6, 126.8, 126.6, 98.4 (d, *J* = 6.1 Hz), 76.4, 71.6 (d, *J* = 15.7 Hz), 64.9 (d, *J* = 14.1 Hz), 62.1, 43.4, 34.4, 33.5, 32.1, 31.7, 31.4, 30.1, 29.6, 28.4, 27.5, 27.2, 27.0, 26.7, 26.4, 21.3. (2C not resolved). **IR** (ATR, neat) 2924, 2876, 2851, 2831, 1492, 1449, 1374, 1175, 956, 762, 704. **HRMS** (ESI/QTOF) m/z: $[M-Cl]^+$ calculated for $C_{29}H_{43}NRh^+$ 508.2445; found 508.2461. **$[\alpha]_D^{21}$** = -98.33 (c = 0.1, CH_2Cl_2). **mp** 208.5-211.0 °C (*decomp.*). **R_f** = 0.78 (3:7 EtOAc/pentane, UV, CAM).

(*R*,*R*)-[Me,Cy]$^{Me, Ph}$CAAC-Rh(COD)Cl (dia2) (Rh1)

IUPAC: ((*R*)-1-((*R*)-1-cyclohexylethyl)-3,5,5-trimethyl-3-phenylpyrrolidin-2-ylidene)(1,5-cyclooctadiene)rhodium(I) chloride

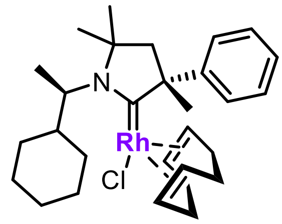

Following Method A using CAAC precursor **8a** (26.6 mg, 68.9 µmol) and $[Rh(COD)Cl]_2$ (15.4 mg, 31.2 µmol) afforded the corresponding rhodium complex (*R*,*R*)-(**Rh1**) (10.5 mg, 19.2 µmol, 28 %) as a yellow solid after purification by column chromatography. **^{1}H NMR** (400 MHz, CD_2Cl_2) δ 7.30–7.25 (m, 2H), 7.21–7.17 (m, 1H), 7.08–7.05 (m, 2H), 5.20–5.15 (m, 1H), 4.69–4.63 (m, 1H), 3.89–3.82 (m, 1H), 3.39–3.31 (m, 1H), 3.14–3.08 (m, 1H), 2.94–2.90 (m, 1H), 2.51–2.41 (m, 1H), 2.26–2.19 (m, 1H), 2.18 (s, 3H), 2.18 (d, *J* = 13.0 Hz, 1H), 2.05–1.97 (m, 3H), 1.99 (d, *J* = 13.0 Hz, 1H), 1.93 (d, *J* = 7.0 Hz, 4H), 1.95–1.92 (m, 1H), 1.82–1.72 (m, 3H), 1.69–1.57 (m, 2H), 1.51–1.42 (m, 1H), 1.39 (s, 3H), 1.34–1.28 (m, 2H), 1.25–1.19 (m, 1H), 1.15–1.09 (m, 1H), 1.13 (s, 3H), 1.09–1.01 (m, 1H). **^{13}C NMR** (101 MHz, CD_2Cl_2) δ 260.6 (d, *J* = 44.1 Hz), 148.5, 128.5, 126.8, 126.7, 99.1 (d, *J* = 6.9 Hz), 98.0 (d, *J* = 5.8 Hz), 77.2, 77.2, 73.2 (d, *J* = 15.6 Hz), 64.0 (d, *J* = 14.8 Hz), 62.7, 42.9, 35.4, 33.4, 31.1, 30.9, 30.8, 30.1, 29.7, 27.3, 26.8, 26.7, 26.5, 26.3, 25.1. **IR** (ATR, neat) 2920, 2876, 2850, 2830, 1492, 1449, 1375, 1175, 956, 763, 705. **HRMS** (ESI/QTOF) m/z: $[M-Cl]^+$ calculated for $C_{29}H_{43}NRh^+$ 508.2445; found 508.2446. **$[\alpha]_D^{21}$** = +58.33 (c = 0.1, CH_2Cl_2). **mp** 187.0-189.3 °C (*decomp.*). **R_f** = 0.61 (3:7 EtOAc/pentane, UV, CAM).

(*R*,*S*)-[Me,Cy]$^{Me, Ph}$CAAC-Rh(nbd)Cl (Rh2)

IUPAC: (bicyclo[2.2.1]hepta-2,5-diene)-((*S*)-1-((*R*)-1-cyclohexylethyl)-3,5,5-trimethyl-3-phenylpyrrolidin-2-ylidene)rhodium(I) chloride

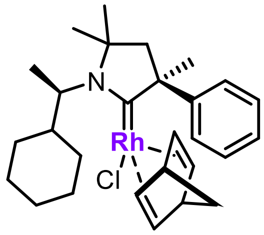

Following Method A using CAAC precursor **8a** (23.6 mg, 61.3 µmol) and $[Rh(nbd)Cl]_2$ (15.0 mg, 32.4 µmol) afforded the corresponding rhodium complex (*R*,*S*)-(**Rh2**) (13.6 mg, 25.7 µmol, 42 %) as a yellow solid after purification by column chromatography. **^{1}H NMR** (400 MHz, CD_2Cl_2) δ 7.30–7.25 (m, 2H), 7.20–7.16 (m, 1H), 6.99–6.95 (m, 2H), 4.61–4.56 (m, 2H), 4.27 (br s, 1H), 3.63–3.60 (m, 1H), 3.37–3.30 (m, 2H), 3.27–3.24 (m, 1H), 2.38–2.35 (m, 1H), 2.32–2.26 (m, 1H), 2.13–2.08 (m, 1H), 2.11 (s, 3H), 2.04 (d, *J* = 13.0 Hz, 1H), 1.96–1.90 (m, 1H), 1.93 (d, *J* = 12.9 Hz, 1H), 1.74–1.69 (m, 1H), 1.72 (d, *J* = 6.9 Hz, 3H), 1.65–1.60 (m, 1H), 1.50–1.42 (m, 1H), 1.40–1.32 (m, 1H), 1.36 (s, 3H), 1.25–1.20 (m, 1H), 1.19 (s, 3H), 1.16–1.09 (m, 3H), 0.99–0.89 (m, 1H). **^{13}C NMR** (101 MHz, CD_2Cl_2) δ 265.0 (d, *J* = 50.4 Hz), 148.9, 128.5, 128.5, 126.8, 126.8, 126.4, 79.2 (d, *J* = 5.6 Hz), 78.9 (d, *J* = 5.2 Hz), 76.6 (d, *J* = 2.5 Hz), 64.5, 63.2 (d, *J* = 5.3 Hz), 61.8, 54.7, 53.6 (d, *J* = 14.2 Hz), 50.6 (d, *J* = 2.7 Hz), 49.7 (d, *J* = 2.5 Hz), 45.8 (d, *J* = 12.8 Hz), 43.7, 33.1, 31.2, 30.9, 28.8, 27.6, 27.2, 26.7, 26.1, 21.8. **IR** (ATR, neat) 2922, 2849, 1493, 1448, 1374, 1175, 735, 702. **HRMS** (ESI/QTOF) m/z: $[M-Cl]^+$ calculated for $C_{28}H_{39}NRh^+$ 492.2132; found 492.2149. **$[\alpha]_D^{21}$** = -55.00 (c = 0.1, CH_2Cl_2). **mp** 187.0-189.5 °C (*decomp.*). **R*f*** = 0.68 (3:7 EtOAc/pentane, UV, CAM).

(*R*,*R*)-[Me,Cy]$^{Me, Ph}$CAAC-Rh(nbd)Cl (Rh2)

IUPAC: (bicyclo[2.2.1]hepta-2,5-diene)-((*R*)-1-((*R*)-1-cyclohexylethyl)-3,5,5-trimethyl-3-phenylpyrrolidin-2-ylidene)rhodium(I) chloride

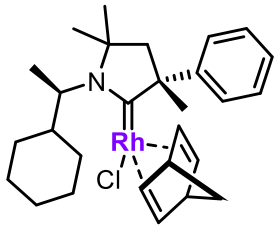

Following Method A using CAAC precursor **8a** (23.6 mg, 61.3 µmol) and $[Rh(nbd)Cl]_2$ (15.0 mg, 32.4 µmol) afforded the corresponding rhodium complex (*R*,*R*)-(**Rh2**) (12.1 mg, 22.9 µmol, 37 %) as a yellow solid after purification by column chromatography. **^{1}H NMR** (400 MHz, CD_2Cl_2) δ 7.33–7.28 (m, 2H), 7.21–7.17 (m, 1H), 7.07–7.03 (m, 2H), 4.63–4.60 (m, 1H), 4.53–4.50 (m, 1H), 4.15–4.05 (m, 1H), 3.62–3.53 (m, 2H), 3.30–3.28 (m, 1H), 3.13–3.10 (m, 1H), 2.33–2.27 (m, 1H), 2.10–2.07 (m, 1H), 2.05 (s, 3H), 2.04 (d, *J* = 13.3 Hz, 1H), 1.99–1.92 (m, 1H), 1.95 (d, *J* = 13.3 Hz, 1H), 1.90–1.81 (m, 2H), 1.76 (d, *J* = 6.9 Hz, 3H), 1.66–1.58 (m, 1H), 1.54–1.46 (m, 1H), 1.36 (s, 3H), 1.39–1.30 (m, 1H), 1.30–1.22 (m, 1H), 1.23 (s, 3H), 1.17–1.05 (m, 4H). **^{13}C NMR** (101 MHz, CD_2Cl_2) δ 261.9 (d, *J* = 50.8 Hz), 148.9, 128.6, 128.6, 126.9, 126.9, 126.5, 79.3 (d, *J* = 5.7 Hz), 77.8 (d, *J* = 5.1 Hz), 77.5 (d, *J* = 2.7 Hz), 63.1 (d, *J* = 5.3 Hz), 62.4, 61.7, 54.9 (d, *J* = 14.0 Hz), 54.1, 50.9 (d, *J* = 2.9 Hz), 49.6 (d, *J* = 2.5 Hz), 44.9 (d, *J* = 12.5 Hz), 42.8, 33.1, 30.9, 30.6, 28.9, 28.0, 27.3, 26.8, 26.3, 24.4. (1C not resolved). **HRMS** (ESI/QTOF) m/z: $[M-Cl]^+$ calculated for $C_{28}H_{39}NRh^+$ 492.2132; found 492.2149. **$[\alpha]_D^{21}$** = -121.67 (c = 0.1, CH_2Cl_2). **mp** 174.4-177.0 °C (*decomp.*). **R*f*** = 0.36 (3:7 EtOAc/pentane, UV, CAM).

Ruthenium complexes

(*R*,*S*)-[Me,Cy]$^{Me, Ph}$CAAC-Ru (Ru1)

IUPAC: ((*S*)-1-((*R*)-1-cyclohexylethyl)-3,5,5-trimethyl-3-phenylpyrrolidin-2-ylidene)(2-isopropoxybenzylidene)ruthenium(II) chloride

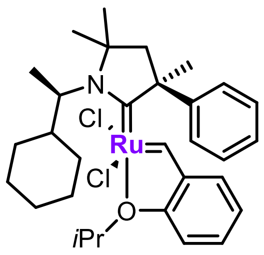

Following Method A using CAAC precursor **8a-I** (28.7 mg, 74.6 µmol, 1.2 equiv.) and Hoveyda-Grubbs 1st generation catalyst (33.8 mg, 56.3 µmol, 1.0 equiv.) afforded the corresponding ruthenium complex (*R*,*S*)-(**Ru1**) (27.9 mg, 45.2 µmol, 80 %) as a green solid after purification by column chromatography. **^{1}H NMR** (400 MHz, CD_2Cl_2) δ 17.59 (s, 1H), 7.61 (ddd, *J* = 8.8, 7.4, 1.7 Hz, 1H), 7.51–7.48 (m, 2H), 7.32 (t, *J* = 7.7 Hz, 2H), 7.25–7.20 (m, 1H), 7.17 (dd, *J* = 7.6, 1.7 Hz, 1H), 7.02 (d, *J* = 8.4 Hz, 1H), 6.94 (td, *J* = 7.5, 0.8 Hz, 1H), 5.16 (hept, *J* = 6.2 Hz, 1H), 4.93–4.85 (m, 1H), 2.29–2.21 (m, 1H), 2.25 (d, *J* = 12.8 Hz, 1H), 2.16 (d, *J* = 12.8 Hz, 1H), 2.15–2.11 (m, 1H), 2.03–1.97 (m, 1H), 1.92–1.85 (m, 2H), 1.89 (d, *J* = 7.0 Hz, 3H), 1.83 (s, 3H), 1.82–1.78 (m, 1H), 1.75–1.71 (m, 1H), 1.73 (d, *J* = 6.1 Hz, 3H), 1.72 (d, *J* = 6.1 Hz, 3H), 1.58 (s, 3H), 1.48 (s, 3H), 1.45–1.36 (m, 1H), 1.34–1.26 (m, 3H). **^{13}C NMR** (101 MHz, CD_2Cl_2) δ 295.2, 263.8, 153.5, 150.4, 143.9, 131.0, 128.7, 126.8, 126.7, 123.2, 122.5, 113.6, 75.0, 73.6, 68.3, 62.8, 60.1, 44.6, 33.0, 32.4, 31.2, 28.9, 26.8, 26.6, 26.4, 26.3, 22.4, 22.3, 21.4. **IR** (ATR, neat) 2979, 2926, 2850, 1588, 1474, 1446, 1422, 1382, 1334, 1221, 1141, 938, 747, 701. **HRMS** (Nanochip-ESI/LTQ-Orbitrap) m/z: $[M-Cl+CH_3CN]^+$ calculated for $C_{33}H_{46}ClN_2ORu^+$ 623.2337; found 623.2359. **[α]$_D^{21}$** = -298.50 (c = 0.1, $CHCl_3$). **mp** 217.5–220.3 °C. **R$_f$** = 0.42 (1:1 DCM/pentane, UV, CAM).

(*R*,*R*)-[Me,Cy]$^{Me, Ph}$CAAC-Ru (Ru1)

IUPAC: ((*R*)-1-((*R*)-1-cyclohexylethyl)-3,5,5-trimethyl-3-phenylpyrrolidin-2-ylidene)(2-isopropoxybenzylidene)ruthenium(II) chloride

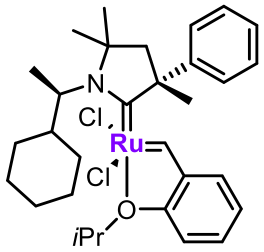

Following Method A using CAAC precursor **8a-II** (11.4 mg, 29.6 µmol, 1.2 equiv.) and Hoveyda-Grubbs 1st generation catalyst (14.4 mg, 24.0 µmol, 1.0 equiv.) afforded the corresponding ruthenium complex (*R*,*R*)-(**Ru1**) (11.0 mg, 17.9 µmol, 74 %) as a green solid after purification by column chromatography. **^{1}H NMR** (400 MHz, CD_2Cl_2) δ 17.44 (s, 1H), 7.60 (ddd, *J* = 8.8, 7.4, 1.7 Hz, 1H), 7.56–7.53 (m, 2H), 7.32–7.27 (m, 2H), 7.24–7.20 (m, 1H), 7.07 (dd, *J* = 7.6, 1.7 Hz, 1H), 7.01 (d, *J* = 8.4 Hz, 1H), 6.93 (td, *J* = 7.4, 0.8 Hz, 1H), 5.17 (hept, *J* = 6.1 Hz, 1H), 5.08–5.01 (m, 1H), 2.25 (s, 2H), 2.23–2.08 (m, 3H), 1.93–1.81 (m, 2H), 1.84 (d, *J* = 6.9 Hz, 3H), 1.83 (s, 3H), 1.77–1.70 (m, 2H), 1.75 (d, *J* = 6.1 Hz, 3H), 1.71 (d, *J* = 6.1 Hz, 3H), 1.60 (s, 3H), 1.48 (s, 3H), 1.42–1.28 (m, 2H), 1.26–1.14 (m, 2H). **^{13}C NMR** (101 MHz, CD_2Cl_2) δ 295.0, 264.5, 153.3, 149.9, 143.8, 130.9, 128.4, 127.4, 126.7, 123.0, 122.5, 113.5, 75.0, 73.9, 71.4, 62.7, 60.5, 43.6, 33.7, 32.0, 31.9, 29.6, 28.0, 27.1, 26.7, 26.7, 23.3, 22.4, 22.1. **IR** (ATR, neat) 2979, 2925, 2850, 1588, 1474, 1449, 1421, 1378, 1332, 1221, 1139, 938, 747, 703. **HRMS** (Nanochip-ESI/LTQ-Orbitrap) m/z: $[M-Cl+CH_3CN]^+$ calculated for $C_{33}H_{46}ClN_2ORu^+$ 623.2337; found 623.2322. **[α]$_D^{21}$** = +75.17 (c = 0.1, $CHCl_3$). **mp** 191.1–193.6 °C. **R$_f$** = 0.39 (1:1 DCM/pentane, UV, CAM).

(*R*,*S*)-[Me,Cy]$^{Me, 1-Np}$CAAC-Ru (Ru2)

IUPAC: ((*S*)-1-((*R*)-1-cyclohexylethyl)-3,5,5-trimethyl-3-(naphthalen-1-yl)pyrrolidin-2-ylidene)(2-isopropoxybenzylidene)ruthenium(II) chloride

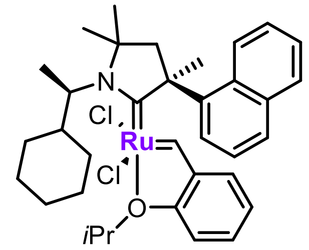

Following Method A using CAAC precursor **8b-I** (13.6 mg, 31.1 µmol, 1.2 equiv.) and Hoveyda-Grubbs 1st generation catalyst (15.5 mg, 25.8 µmol, 1.0 equiv.) afforded the corresponding ruthenium complex (*R*,*S*)-(**Ru2**) (9.90 mg, 14.8 µmol, 57 %) as a green solid after purification by column chromatography. NMR analysis revealed the existence of two rotamers in a 1:0.7 ratio in CD_2Cl_2 at 23 °C. **^{1}H NMR** (400 MHz, CD_2Cl_2) δ 18.06 (s, 1H), 17.71 (s, 0.7H), 8.40 (dd, *J* = 8.4, 1.4 Hz, 1H), 8.10 (dd, *J* = 7.5, 1.2 Hz, 1H), 8.05 (dd, *J* = 7.5, 1.2 Hz, 0.7H), 7.94 (dd, *J* = 7.8, 1.8 Hz, 1H), 7.88 (d, *J* = 8.2 Hz, 0.7H), 7.80 (dd, *J* = 8.2, 1.5 Hz, 0.7H), 7.77–7.73 (m, 1.7H), 7.66–7.61 (m, 1.7H), 7.59–7.50 (m, 2.7H), 7.35 (ddd, *J* = 8.0, 6.8, 1.2 Hz, 0.7H), 7.30–7.24 (m, 1.7H), 7.18 (t, *J* = 7.8 Hz, 1H), 7.08 (d, *J* = 8.4 Hz, 1H), 6.95–6.91 (m, 1.7H), 6.88–6.82 (m, 1.4H), 5.28–5.19 (m, 2H), 5.06 (hept, *J* = 6.1 Hz, 0.7H), 4.58 (br s, 0.7H), 2.84 (d, *J* = 12.6 Hz, 1H), 2.78–2.67 (m, 0.7H), 2.69 (d, *J* = 13.3 Hz, 0.7H), 2.53–2.48 (m, 0.7H), 2.51 (d, *J* = 12.5 Hz, 1H), 2.29–2.24 (m, 1.7H), 2.20 (s, 3H), 2.16–2.11 (m, 1H), 2.12 (d, *J* = 5.4 Hz, 0.7H), 2.01 (d, *J* = 7.0 Hz, 2.1H), 1.95–1.89 (m, 1.7H), 1.93 (d, *J* = 7.1 Hz, 3H), 1.89–1.87 (m, 1H), 1.86 (s, 2.1H), 1.84–1.81 (m, 1H), 1.80 (d, *J* = 6.1 Hz, 3H), 1.79 (d, *J* = 6.1 Hz, 3H), 1.77–1.72 (m, 3.4H), 1.70 (d, *J* = 6.2 Hz, 2.1H), 1.69 (s, 2.1H), 1.64 (s, 2.1H), 1.61 (d, *J* = 6.1 Hz, 2.1H), 1.58 (s, 3H), 1.55–1.49 (m, 0.7H), 1.47–1.28 (m, 5.1H), 1.27–1.23 (m, 1.7H), 1.20 (s, 3H). **^{13}C NMR** (101 MHz, CD_2Cl_2) δ 295.2, 264.7, 154.0, 152.8, 145.6, 145.2, 144.1, 143.8, 135.7, 135.4, 131.2, 131.1, 130.8, 130.6, 130.2, 129.0, 128.8, 128.5, 128.1, 127.1, 126.5, 125.8, 125.7, 125.5, 125.4, 125.3, 125.3, 124.3, 123.7, 123.3, 122.6, 122.2, 113.7, 113.5, 75.3, 74.6, 74.3, 73.8, 68.1, 67.5, 64.5, 61.8, 57.7, 56.8, 44.9, 43.9, 33.8, 32.5, 32.3, 31.3, 30.8, 30.1, 29.9, 29.4, 28.1, 26.9, 26.6, 26.5, 26.4, 26.0, 22.7, 22.4, 22.3, 21.4. (6C not resolved). **IR** (ATR, neat) 3048, 2978, 2925, 2851, 1588, 1450, 1383, 1225, 1116, 938, 804, 736. **HRMS** (Nanochip-ESI/LTQ-Orbitrap) m/z: $[M-Cl]^+$ calculated for $C_{35}H_{45}ClNORu^+$ 632.2228; found 632.2206. **$[\alpha]_D^{21}$** = -279.83 (c = 0.1, $CHCl_3$). **mp** 175.4-179.3 °C. **R_f** = 0.38 (1:1 DCM/pentane, UV, CAM).

(*R*,*R*)-[Me,Cy]$^{Me, 1-Np}$CAAC-Ru (Ru2)

IUPAC: ((*R*)-1-((*R*)-1-cyclohexylethyl)-3,5,5-trimethyl-3-(naphthalen-1-yl)pyrrolidin-2-ylidene)(2-isopropoxybenzylidene)ruthenium(II) chloride

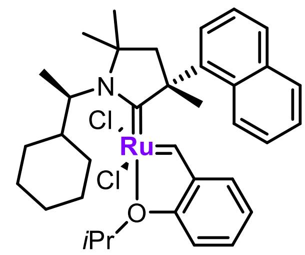

Following Method A using CAAC precursor **8b-II** (13.0 mg, 29.8 µmol, 1.2 equiv.) and Hoveyda-Grubbs 1st generation catalyst (14.8 mg, 24.6 µmol, 1.0 equiv.) afforded the corresponding ruthenium complex (*R*,*R*)-(**Ru2**) (12.8 mg, 19.2 µmol, 78 %) as a green solid after purification by column chromatography. NMR analysis revealed the existence of two rotamers in a 1:1 ratio in CD_2Cl_2 at 23 °C. **^{1}H NMR** (400 MHz, CD_2Cl_2) δ 17.99 (s, 1H), 17.52 (s, 1H), 8.38–8.35 (m, 1H), 8.21 (dd, *J* = 7.5, 1.2 Hz, 1H), 8.08 (dd, *J* = 7.5, 1.2 Hz, 1H), 7.95 (dd, *J* = 7.8, 1.8 Hz, 1H), 7.89–7.86 (m, 1H), 7.81–7.78 (m, 1H), 7.76 (d, *J* = 8.0, 1H), 7.72–7.68 (m, 1H), 7.66–7.61 (m, 2H), 7.59–7.49 (m, 3H), 7.38–7.32 (m, 2H), 7.18–7.10 (m, 2H), 7.08 (d, *J* = 8.4 Hz, 1H), 6.95–6.91 (m, 3H), 6.87–6.83 (m, 1H), 5.29–5.21 (m, 2H), 5.20–5.13 (m, 1H), 5.09 (hept, *J* = 6.1 Hz, 1H), 2.92 (d, *J* = 12.3 Hz, 1H), 2.86 (d, *J* = 13.2 Hz, 1H), 2.49–2.43 (m, 1H), 2.47 (d, *J* = 12.3 Hz, 1H), 2.37–2.27 (m, 1H), 2.25–2.18 (m, 2H), 2.17 (s, 3H), 2.16–2.12 (m, 1H), 2.15 (d, *J* = 13.0 Hz, 1H), 2.10–2.06 (m, 1H), 1.96–1.91 (m, 1H), 1.93 (d, *J* = 6.9 Hz, 3H), 1.93 (s, 3H), 1.89–1.85 (m, 2H), 1.86 (d, *J* = 6.6 Hz, 3H), 1.84–1.82 (m, 1H), 1.83 (d, *J* = 6.1 Hz, 3H), 1.80–1.78 (m, 1H), 1.79 (d, *J* = 6.0 Hz, 3H), 1.76 (s, 3H), 1.76–1.73 (m, 2H), 1.72 (s, 3H), 1.70 (d, *J* = 6.1 Hz, 3H), 1.61 (d, *J* = 6.1 Hz, 3H), 1.58 (s, 3H), 1.58–1.54 (m, 1H), 1.49–1.28 (m, 7H), 1.25–1.22 (m, 1H), 1.20–1.19 (m, 3H). **^{13}C NMR** (101 MHz, CD_2Cl_2) δ 294.1, 268.2, 263.5, 153.9, 152.8, 144.7, 143.9, 143.7, 143.6, 135.8, 135.4, 131.0, 130.9, 130.7, 130.7, 130.3, 128.9, 128.9, 128.7, 128.4, 128.4, 126.5, 125.7, 125.7, 125.5, 125.3, 125.2, 124.8, 124.5, 123.3, 123.2, 122.6, 122.3, 113.7, 113.4, 75.3, 74.9, 74.1, 73.6, 72.3, 71.8, 63.5, 62.4, 58.9, 57.1, 45.1, 42.8, 35.0, 32.7, 32.2, 32.2, 31.6, 31.5, 31.4, 30.6, 28.9, 27.7, 27.2, 27.1, 26.8, 26.7, 26.6, 22.9, 22.4, 22.3, 22.1, 22.0, 21.9. (2C not resolved). **IR** (ATR, neat) 3048, 2978, 2925, 2851, 1589, 1449, 1382, 1222, 1116, 938, 804, 746. **HRMS** (Nanochip-ESI/LTQ-Orbitrap) m/z: $[M-Cl]^+$ calculated for $C_{35}H_{45}ClNORu^+$ 632.2228; found 632.2222. **$[\alpha]_D^{21}$** = -202.17 (c = 0.1, $CHCl_3$). **mp** 206.0-211.6 °C. **R_f** = 0.36 (1:1 DCM/pentane, UV, CAM).

(*R*,*S*)-[Me,Cy]$^{Me, 2-Np}$CAAC-Ru (Ru3)

IUPAC: ((*S*)-1-((*R*)-1-cyclohexylethyl)-3,5,5-trimethyl-3-(naphthalen-2-yl)pyrrolidin-2-ylidene)(2-isopropoxybenzylidene)ruthenium(II) chloride

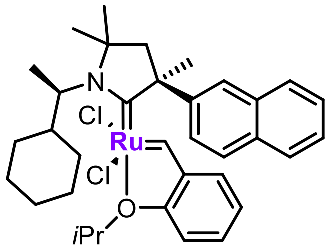

Following Method A using CAAC precursor **8c-I** (18.2 mg, 41.8 µmol, 1.2 equiv.) and Hoveyda-Grubbs 1st generation catalyst (20.8 mg, 34.6 µmol, 1.0 equiv.) afforded the corresponding ruthenium complex (*R*,*S*)-(**Ru3**) (13.5 mg, 20.2 µmol, 58 %) as a green solid after purification by column chromatography. **^{1}H NMR** (400 MHz, CD_2Cl_2) δ 17.69 (s, 1H), 8.14–8.12 (m, 1H), 7.84–7.78 (m, 3H), 7.59–7.53 (m, 2H), 7.47–7.41 (m, 2H), 7.04–6.98 (m, 2H), 6.87–6.82 (m, 1H), 5.20–5.13 (m, 1H), 5.00–4.92 (m, 1H), 2.33 (d, *J* = 12.9 Hz, 1H), 2.31–2.26 (m, 1H), 2.20 (d, *J* = 12.9 Hz, 1H), 2.20–2.14 (m, 1H), 2.10–1.98 (m, 2H), 1.94 (s, 3H), 1.93 (d, *J* = 6.8 Hz, 3H), 1.93–1.91 (m, 1H), 1.89–1.81 (m, 2H), 1.76–1.70 (m, 1H), 1.74 (d, *J* = 6.1 Hz, 3H), 1.72 (d, *J* = 5.9 Hz, 3H), 1.61 (s, 3H), 1.47 (s, 3H), 1.43–1.32 (m, 3H). **^{13}C NMR** (101 MHz, CD_2Cl_2) δ 295.4, 264.0, 153.5, 147.8, 143.9, 133.8, 132.5, 131.0, 128.6, 128.4, 127.8, 126.3, 126.0, 124.9, 123.2, 122.4, 113.6, 75.0, 73.6, 68.4, 62.8, 59.8, 44.6, 33.0, 32.4, 31.2, 28.7, 26.8, 26.6, 26.5, 26.4, 22.4, 22.2, 21.4. (1 C not resolved). **IR** (ATR, neat) 2978, 2829, 2851, 1589, 1474, 1451, 1427, 1384, 1230, 1141, 939, 816, 747. **HRMS** (Nanochip-based ESI/LTQ-Orbitrap) m/z: $[M-Cl]^+$ calculated for $C_{35}H_{45}ClNORu^+$ 632.2228; found 632.2224. **$[\alpha]_D^{21}$** = -267.33 (c = 0.1, $CHCl_3$). **mp** 206.7-209.6 °C. **R_f** = 0.42 (1:1 DCM/pentane, UV, CAM).

(*R*,*R*)-[Me,Cy]$^{Me, 2-Np}$CAAC-Ru (Ru3)

IUPAC: ((*R*)-1-((*R*)-1-cyclohexylethyl)-3,5,5-trimethyl-3-(naphthalen-2-yl)pyrrolidin-2-ylidene)(2-isopropoxybenzylidene)ruthenium(II) chloride

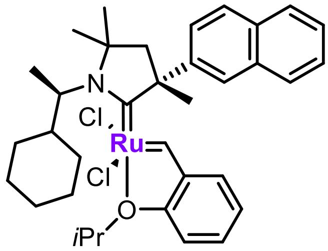

Following Method A using CAAC precursor **8c-II** (25.2 mg, 57.9 µmol, 1.2 equiv.) and Hoveyda-Grubbs 1st generation catalyst (28.8 mg, 48.0 µmol, 1.0 equiv.) afforded the corresponding ruthenium complex (*R*,*R*)-(**Ru3**) (20.5 mg, 30.6 µmol, 64 %) as a green solid after purification by column chromatography. **^{1}H NMR** (400 MHz, CD_2Cl_2) δ 17.56 (s, 1H), 8.19 (d, *J* = 2.0 Hz, 1H), 7.80–7.75 (m, 3H), 7.60–7.53 (m, 2H), 7.46–7.42 (m, 2H), 6.99 (d, *J* = 8.4 Hz, 1H), 6.86–6.78 (m, 2H), 5.22–5.13 (m, 1H), 5.13–5.06 (m, 1H), 2.34 (d, *J* = 12.9 Hz, 1H), 2.29 (d, *J* = 12.8 Hz, 1H), 2.25–2.09 (m, 3H), 1.94 (s, 3H), 1.93–1.91 (m, 1H), 1.89 (d, *J* = 6.7 Hz, 3H), 1.86–1.80 (m, 2H), 1.78–1.75 (m, 1H), 1.77 (d, *J* = 6.1 Hz, 3H), 1.74–1.70 (m, 1H), 1.72 (d, *J* = 6.1 Hz, 3H), 1.62 (s, 3H), 1.47 (s, 3H), 1.43–1.31 (m, 2H), 1.26–1.16 (m, 1H). **^{13}C NMR** (101 MHz, CD_2Cl_2) δ 295.3, 264.5, 153.3, 147.3, 143.8, 133.5, 132.5, 130.9, 128.5, 128.1, 127.8, 126.8, 126.3, 126.0, 125.2, 123.0, 122.5, 113.5, 75.1, 73.9, 71.5, 62.8, 60.2, 43.7, 33.6, 32.0, 31.9, 29.6, 28.2, 27.1, 26.7, 26.7, 23.3, 22.4, 22.1. **IR** (ATR, neat) 2979, 2927, 2851, 1589, 1471, 1450, 1426, 1382, 1235, 1158, 939, 816, 747. **HRMS** (Nanochip-based ESI/LTQ-Orbitrap) m/z: $[M-Cl]^+$ calculated for $C_{35}H_{45}ClNORu^+$ 632.2228; found 632.2226. **$[\alpha]_D^{21}$** = +72.83 (c = 0.1, $CHCl_3$). **mp** 137.8-142.0 °C. **R_f** = 0.42 (1:1 DCM/pentane, UV, CAM).

(R,S)-[Me,Cy]^{THN}CAAC-Ru (Ru4)

IUPAC: ((*S*)-1'-((*R*)-1-cyclohexylethyl)-5',5'-dimethyl-3,4-dihydro-2H-spiro[naphthalene-1,3'-pyrrolidin]-2'-ylidene)(2-isopropoxybenzylidene)ruthenium(II) chloride

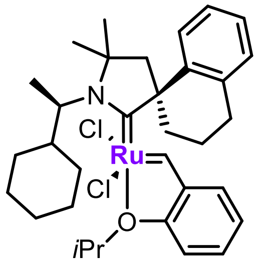

Following Method A using CAAC precursor **8h-I** (19.5 mg, 47.4 µmol, 1.2 equiv.) and Hoveyda-Grubbs 1st generation catalyst (23.4 mg, 39.0 µmol, 1.0 equiv.) afforded the corresponding ruthenium complex (*R*,*S*)-(**Ru4**) (18.7 mg, 29.0 µmol, 74 %) as a green solid after purification by column chromatography. **^{1}H NMR** (400 MHz, CD_2Cl_2) δ 17.49 (s, 1H), 7.61 (ddd, *J* = 8.7, 7.3, 1.7 Hz, 1H), 7.21 (d, *J* = 7.6 Hz, 1H), 7.16–7.10 (m, 2H), 7.02–6.98 (m, 2H), 6.95 (td, *J* = 7.5, 0.9 Hz, 1H), 6.85 (dd, *J* = 7.9, 1.3 Hz, 1H), 5.14 (hept, *J* = 5.9 Hz, 1H), 4.88–4.81 (m, 1H), 3.19–3.02 (m, 2H), 2.46 (d, *J* = 13.1 Hz, 1H), 2.42–2.25 (m, 2H), 2.17–2.11 (m, 1H), 2.14 (d, *J* = 13.1 Hz, 1H), 2.09–2.01 (m, 2H), 1.99–1.92 (m, 2H), 1.91–1.89 (m, 1H), 1.89 (d, *J* = 6.9 Hz, 3H), 1.87–1.82 (m, 2H), 1.76–1.72 (m, 1H), 1.71 (d, *J* = 6.1 Hz, 3H), 1.68 (d, *J* = 6.1 Hz, 3H), 1.60 (s, 6H), 1.49–1.34 (m, 2H), 1.32–1.23 (m, 2H). **^{13}C NMR** (101 MHz, CD_2Cl_2) δ 295.6, 264.3, 153.2, 144.1, 144.1, 135.7, 130.9, 130.5, 128.9, 126.8, 126.8, 123.1, 122.5, 113.6, 74.9, 73.7, 68.4, 64.2, 58.1, 44.8, 34.2, 34.0, 32.6, 31.3, 30.2, 29.5, 26.8, 26.6, 26.3, 22.4, 22.2, 21.6, 20.7. **IR** (ATR, neat) 2980, 2930, 2853, 1589, 1475, 1450, 1423, 1230, 1115, 940, 750. **HRMS** (Nanochip-ESI/LTQ-Orbitrap) m/z: $[M-Cl+CH_3CN]^+$ calculated for $C_{35}H_{48}ClN_2ORu^+$ 649.2493; found 649.2492. **$[\alpha]_D^{21}$** = -228.00 (c = 0.1, $CHCl_3$). **mp** 232.0-234.2 °C. **R_f** = 0.22 (1:1 DCM/pentane, UV, CAM).

(R,R)-[Me,Cy]^{THN}CAAC-Ru (Ru4)

IUPAC: ((*R*)-1'-((*R*)-1-cyclohexylethyl)-5',5'-dimethyl-3,4-dihydro-2H-spiro[naphthalene-1,3'-pyrrolidin]-2'-ylidene)(2-isopropoxybenzylidene)ruthenium(II) chloride

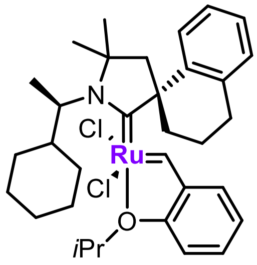

Following Method A using CAAC precursor **8h-II** (12.7 mg, 30.9 µmol, 1.2 equiv.) and Hoveyda-Grubbs 1st generation catalyst (15.6 mg, 26.0 µmol, 1.0 equiv.) afforded the corresponding ruthenium complex (*R*,*R*)-(**Ru4**) (13.5 mg, 21.0 µmol, 81 %) as a green solid after purification by column chromatography. **^{1}H NMR** (400 MHz, CD_2Cl_2) δ 17.28 (s, 1H), 7.59 (ddd, *J* = 8.7, 7.3, 1.7 Hz, 1H), 7.22–7.20 (m, 1H), 7.15–7.09 (m, 1H), 7.04 (dd, *J* = 7.6, 1.7 Hz, 1H), 6.99 (d, *J* = 8.4 Hz, 1H), 6.96–6.91 (m, 3H), 5.13 (h, *J* = 6.1 Hz, 1H), 5.06–4.99 (m, 1H), 3.17–2.99 (m, 2H), 2.52 (d, *J* = 13.2 Hz, 1H), 2.49–2.42 (m, 1H), 2.28–2.21 (m, 2H), 2.18–2.13 (m, 1H), 2.17 (dd, *J* = 13.2, 1.4 Hz, 1H), 2.08–1.89 (m, 3H), 1.86 (d, *J* = 6.9 Hz, 3H), 1.88–1.80 (m, 2H), 1.75–1.70 (m, 1H), 1.72 (d, *J* = 6.1 Hz, 3H), 1.68 (d, *J* = 6.1 Hz, 3H), 1.64 (s, 3H), 1.61 (s, 3H), 1.60–1.56 (m, 1H), 1.43–1.26 (m, 3H), 1.25–1.15 (m, 1H). **^{13}C NMR** (101 MHz, CD_2Cl_2) δ 295.3, 265.1, 153.1, 144.3, 144.0, 135.8, 131.0, 130.8, 128.8, 126.8, 126.5, 123.0, 122.5, 113.5, 74.9, 73.8, 71.1, 63.9, 58.9, 43.1, 34.7, 34.3, 32.4, 31.5, 31.3, 30.1, 27.1, 26.6, 26.6, 24.0, 22.4, 22.1, 20.2. **IR** (ATR, neat) 2982, 2926, 2852, 1589, 1475, 1448, 1421, 1222, 1115, 939, 748. **HRMS** (Nanochip-ESI/LTQ-Orbitrap) m/z: $[M-Cl+CH_3CN]^+$ calculated for $C_{35}H_{48}ClN_2ORu^+$ 649.2493; found 649.2471. **$[\alpha]_D^{21}$** = -27.00 (c = 0.1, $CHCl_3$). **mp** 199.7-201.7 °C. **R_f** = 0.22 (1:1 DCM/pentane, UV, CAM).

Asymmetric Conjugate Borylation (ACB) reaction

(*E*)-Ethyl-5-phenylpent-2-enoate (9)

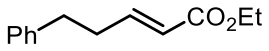

(*E*)-Ethyl-5-phenylpent-2-enoate was prepared following an adapted literature procedures and the characterization data matched those previously reported.[8,9] To a stirred solution of ethyl (triphenylphosphoranylidene)acetate (4.90 g, 13.8 mmol, 1.2 equiv.) in toluene (37.5 mL, 0.3 M) at 23 °C was added 3-phenylpropionaldehyde (1.50 g, 11.2 mmol, 1.0 equiv.) and the reaction mixture was refluxed for 2 h. The reaction was quenched with saturated aqueous NH_4Cl (10 mL) and diluted with EtOAc (50 mL). The organic phase was collected and the aqueous phase was extracted twice with EtOAc (2 × 30 mL). The combined organic layers were washed with brine (30 mL), dried over $MgSO_4$, filtered and concentrated in vacuo. The residue was purified by column chromatography (conditioning: pentane, eluent: EtOAc/pentane 1:99 to 5:95) to afford (*E*)-ethyl-5-phenylpent-2-enoate **9** (1.69 g, 8.27 mmol, 74 %) and (*Z*)-ethyl-5-phenylpent-2-enoate (114 mg, 0.56 mmol, 5 %) as colorless oils.

^{1}H NMR (400 MHz, $CDCl_3$) δ 7.32–7.28 (m, 2H), 7.23–7.17 (m, 3H), 7.01 (dt, *J* = 15.6, 6.8 Hz, 1H), 5.85 (dt, *J* = 15.6, 1.6 Hz, 1H), 4.19 (q, *J* = 7.1 Hz, 2H), 2.80–2.76 (m, 2H), 2.56–2.50 (m, 2H), 1.29 (t, *J* = 7.1 Hz, 3H). **R_f** = 0.62 (1:9 EtOAc/pentane, UV, $KMnO_4$ - (*E*)-isomer). **^{1}H NMR** (400 MHz, $CDCl_3$) δ 7.31–7.27 (m, 2H), 7.23–7.17 (m, 3H), 6.23 (dt, *J* = 11.5, 7.5 Hz, 1H), 5.78 (dt, *J* = 11.5, 1.7 Hz, 1H), 4.17 (q, *J* = 7.2 Hz, 2H), 2.99 (qd, *J* = 7.6, 1.6 Hz, 2H), 2.77 (t, *J* = 7.7 Hz, 2H), 1.28 (t, *J* = 7.1 Hz, 3H). **R_f** = 0.73 (1:9 EtOAc/pentane, UV, $KMnO_4$ - (*Z*)-isomer).

Ethyl 5-phenyl-3-(4,4,5,5-tetramethyl-1,3,2-dioxaborolan-2-yl)pentanoate (10)

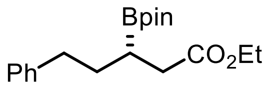

Representative procedure adapted from reported conditions.[10] In a nitrogen-filled glovebox, a vial was charged with **(*R*,*S*)-Cu1** (1.65 mg, 4.2 µmol, 4 mol%) and NaO*t*Bu (2.0 mg, 21 µmol, 20 mol%). Dry and degassed THF (0.15 mL) was added and the mixture was stirred at 23 °C for 10 min. A solution of bis(pinacolato)diboron (31.0 mg, 0.12 mmol, 1.1 equiv.) in dry and degassed THF (0.15 mL) was then added. After stirring at 23 °C for 10 min, a solution of methanol (16 µL, 2.0 equiv.) and (*E*)-ethyl-5-phenylpent-2-enoate (21.3 mg, 0.10 mmol, 1.0 equiv.) in THF (0.20 mL) was slowly added at the indicated temperature (23 °C or -20 °C). The resulting medium was stirred for 16 h at the indicated temperature (23 °C or -20 °C). At the end of the reaction, the mixture was quenched with water (0.4 mL) and extracted Et_2O (3 × 2 mL). The combined organic layers were dried over anhydrous $MgSO_4$, filtered and concentrated under vacuum. The residue was purified by column chromatography (conditioning: pentane, eluent: pentane to 5:95 EtOAc/pentane) to afford product **10** (29.8 mg, 86 %) as a colorless oil. The characterization data matched those previously reported in the literature.[11]

^{1}H NMR (400 MHz, $CDCl_3$) δ 7.28–7.24 (m, 2H), 7.18–7.14 (m, 3H), 4.11 (qd, *J* = 7.1, 1.2 Hz, 2H), 2.69–2.58 (m, 2H), 2.51–2.40 (m, 2H), 1.84–1.76 (m, 1H), 1.68–1.61 (m, 1H), 1.40 (tt, *J* = 8.3, 6.5 Hz, 1H), 1.26–1.22 (m, 15H).

Enantiomeric excess determination.

Ethyl 3-hydroxy-5-phenylpentanoate (S-V)

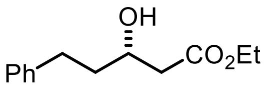

Ethyl 5-phenyl-3-(4,4,5,5-tetramethyl-1,3,2-dioxaborolan-2-yl)pentanoate **10** (44.4 mg, 0.13 mmol) was oxidized with $NaBO_3.4H_2O$ (68.8 mg, 0.68 mmol, 5.0 equiv.) in 1:1 THF/H_2O (660 µL) following a reported procedure[10] to afford ethyl 3-hydroxy-5-phenylpentanoate **S-V** (26.7 mg, 90 %) as a colorless oil and was analyzed as such by HPLC using a chiral stationary phase. The characterization data matched those previously reported in the literature.[12]

^{1}H NMR (400 MHz, $CDCl_3$) δ 7.31–7.26 (m, 2H), 7.22–7.17 (m, 3H), 4.17 (q, *J* = 7.2 Hz, 2H), 4.02 (tp, *J* = 8.3, 4.2 Hz, 1H), 3.07 (dd, *J* = 4.0, 0.9 Hz, 1H), 2.83 (ddd, *J* = 14.7, 9.7, 5.5 Hz, 1H), 2.71 (ddd, *J* = 13.8, 9.5, 6.9 Hz, 1H), 2.55–2.39 (m, 2H), 1.85 (dddd, *J* = 13.8, 9.4, 8.5, 5.5 Hz, 1H), 1.74 (dddd, *J* = 4.0, 6.9, 9.5, 14.0 Hz, 1H), 1.27 (t, *J* = 7.2 Hz, 3H).

$[\alpha]_D^{21}$ = -0.67 (c = 0.25, $CHCl_3$, 95:5 er). Litt: **$[\alpha]_D^{21}$** = -0.64 (c = 1.0, $CHCl_3$, 93:7 er) for (*S*)-alcohol[13].

Chiral HPLC Chiralpak IB-3 column (2:98 *i*PrOH/hexane, 35 °C, 1 mL/min, detection at 210 nm): **t_{R1}** = 8.5 min, **t_{R2}** = 9.5 min.

The authentic racemic alcohol was prepared following a reported procedure and the characterization data matched those previously reported.[12]

Chiral HPLC Chiralpak IB-3 column (2:98 *i*PrOH/hexane, 35 °C, 1 mL/min, detection at 210 nm): $\mathbf{t_{R1}}$ = 8.5 min, $\mathbf{t_{R2}}$ = 9.5 min (ACB Reaction with (*R*,*S*)-**Cu1** at 23 °C).

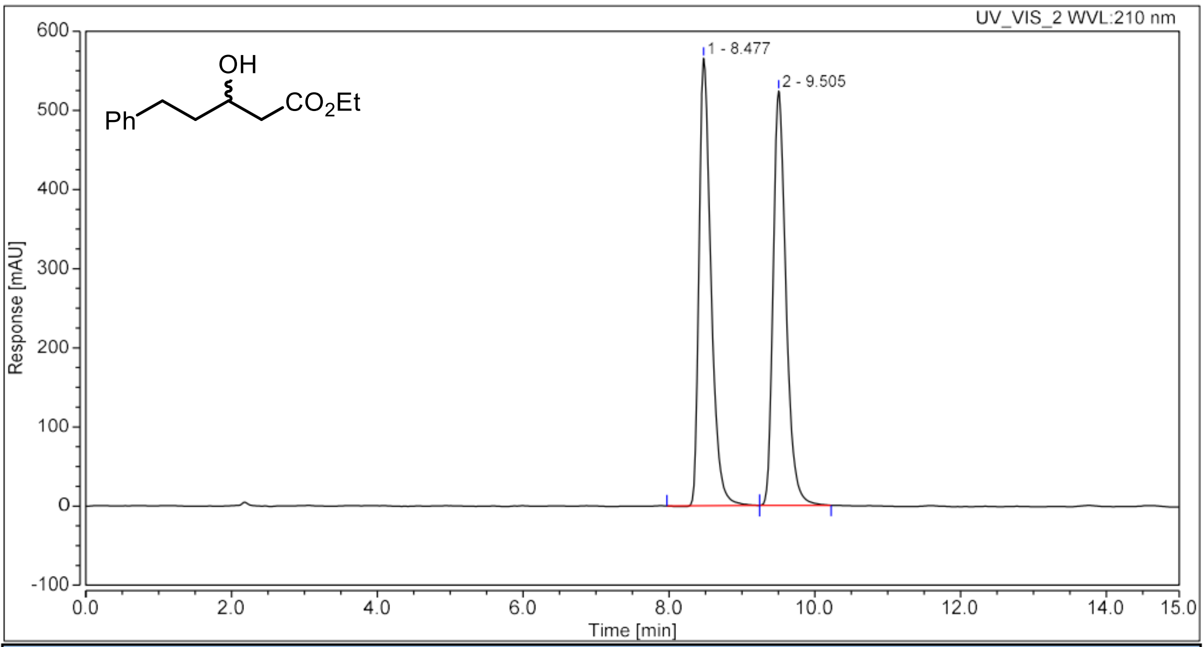

Integration Results

No.	Peak Name	Retention Time min	Area mAU*min	Height mAU	Relative Area %	Relative Height %	Amount n.a.
1		8.477	107.353	565.331	49.99	51.93	n.a.
2		9.505	107.381	523.378	50.01	48.07	n.a.
**Total:**			**214.733**	**1088.708**	**100.00**	**100.00**	

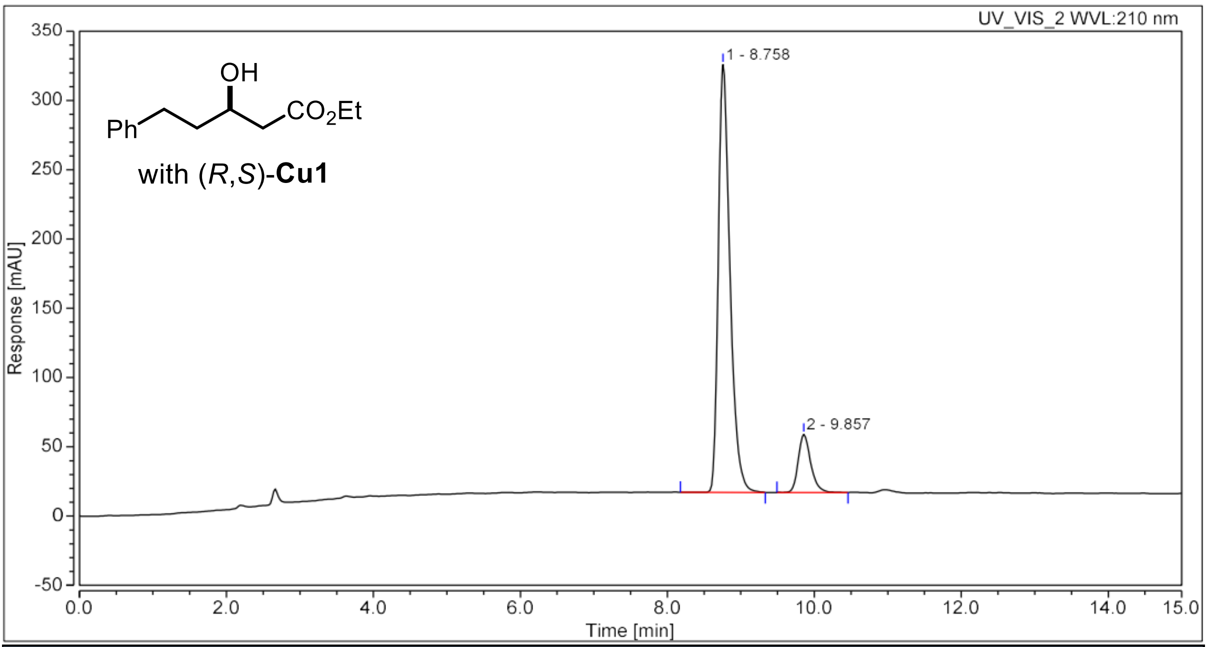

Integration Results

No.	Peak Name	Retention Time min	Area mAU*min	Height mAU	Relative Area %	Relative Height %	Amount n.a.
1		8.758	56.983	308.770	87.31	88.05	n.a.
2		9.857	8.279	41.925	12.69	11.95	n.a.
**Total:**			**65.262**	**350.696**	**100.00**	**100.00**	

Chiral HPLC Chiralpak IB-3 column (2:98 *i*PrOH/hexane, 35 °C, 1 mL/min, detection at 210 nm): $\mathbf{t_{R1}}$ = 8.5 min, $\mathbf{t_{R2}}$ = 9.5 min (ACB Reaction with (*R*,*R*)-**Cu1** at 23 °C).

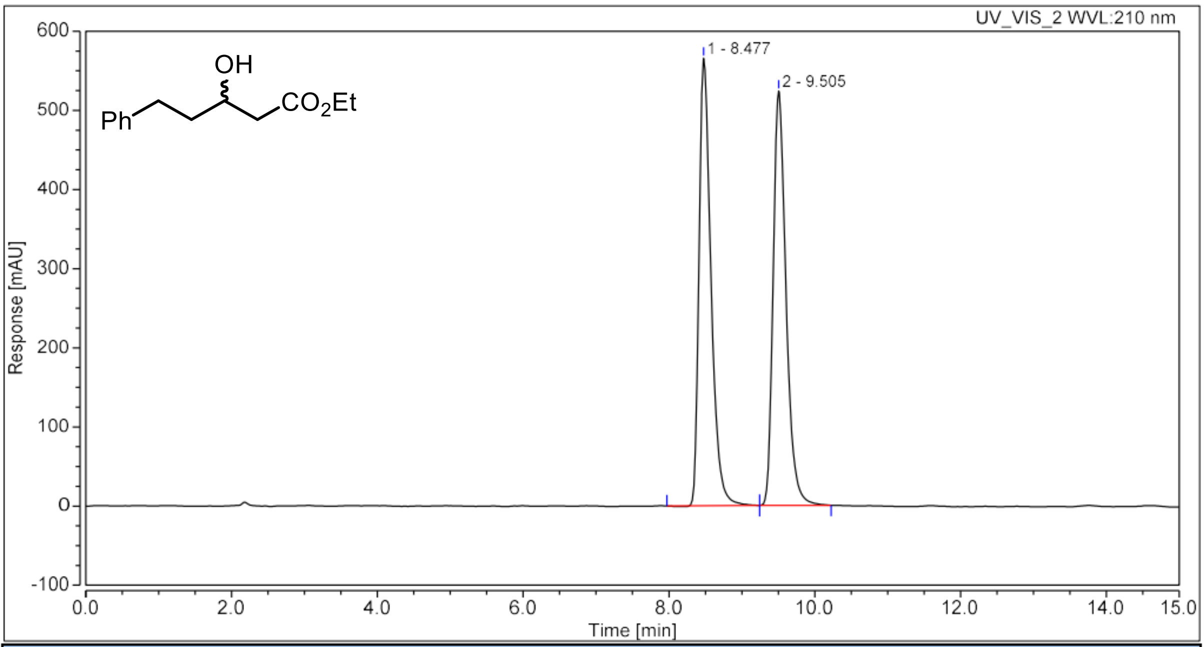

Integration Results							
No.	Peak Name	Retention Time min	Area mAU*min	Height mAU	Relative Area %	Relative Height %	Amount n.a.
1		8.477	107.353	565.331	49.99	51.93	n.a.
2		9.505	107.381	523.378	50.01	48.07	n.a.
**Total:**			**214.733**	**1088.708**	**100.00**	**100.00**	

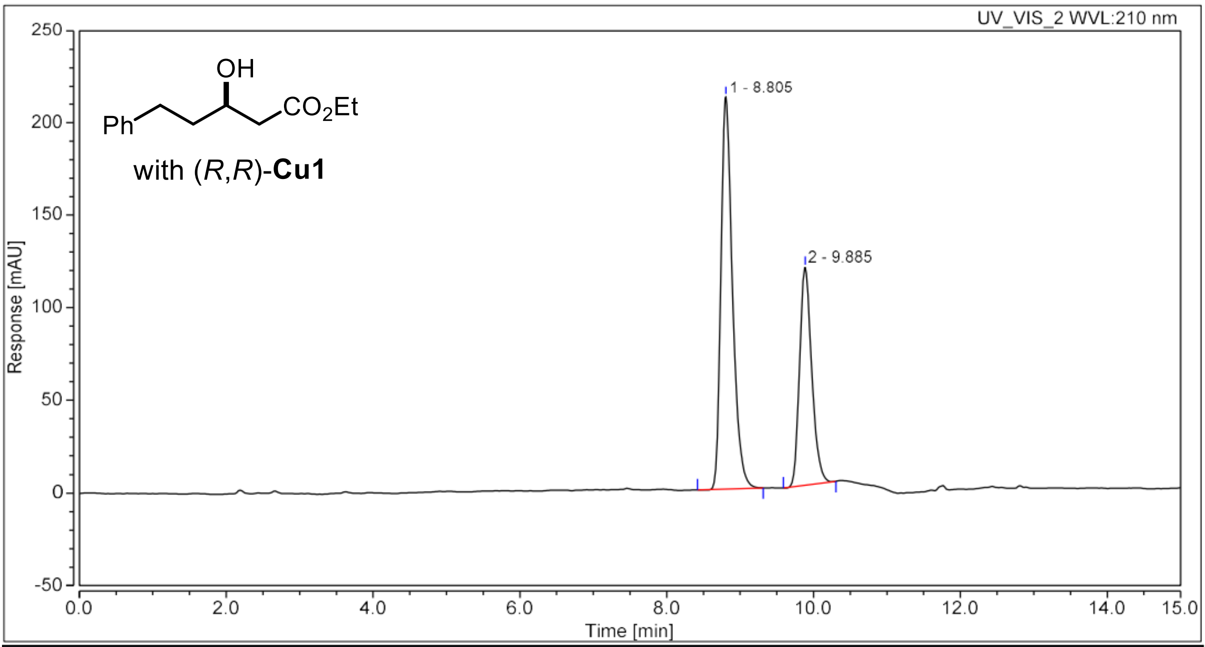

Integration Results							
No.	Peak Name	Retention Time min	Area mAU*min	Height mAU	Relative Area %	Relative Height %	Amount n.a.
1		8.805	38.522	211.679	63.13	64.32	n.a.
2		9.885	22.497	117.446	36.87	35.68	n.a.
**Total:**			**61.019**	**329.125**	**100.00**	**100.00**	

Chiral HPLC Chiralpak IB-3 column (2:98 *i*PrOH/hexane, 35 °C, 1 mL/min, detection at 210 nm): $\mathbf{t_{R1}}$ = 8.5 min, $\mathbf{t_{R2}}$ = 9.5 min (ACB Reaction with (*R*,*S*)-**Cu2** at 23 °C).

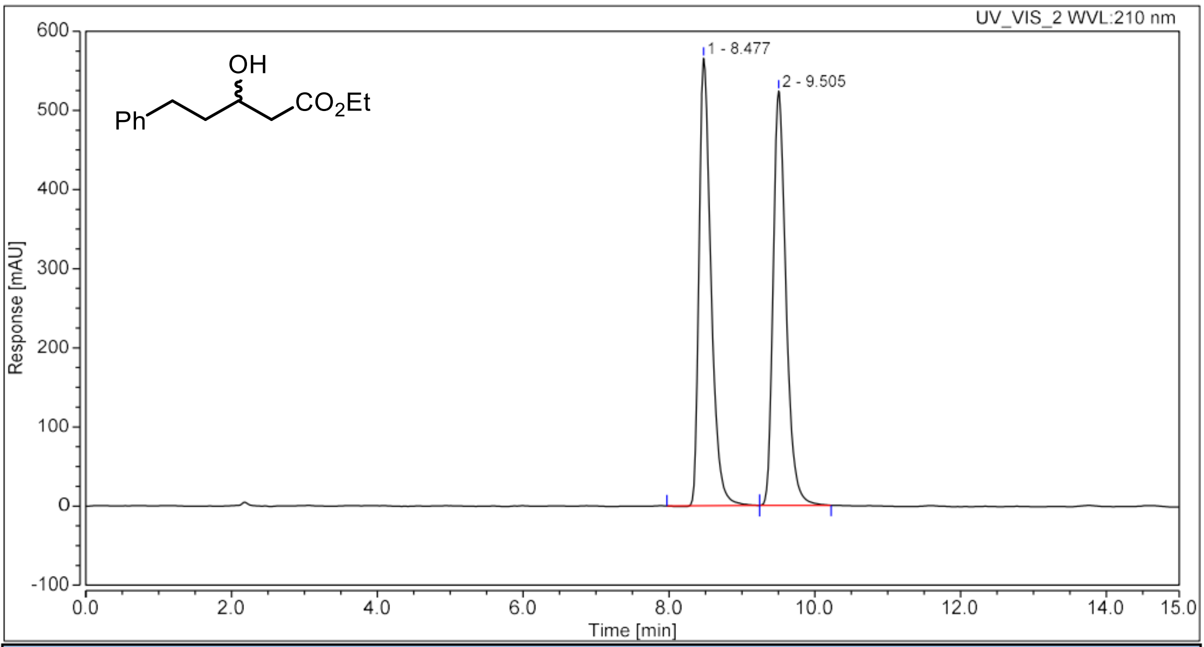

Integration Results

No.	Peak Name	Retention Time min	Area mAU*min	Height mAU	Relative Area %	Relative Height %	Amount n.a.
1		8.477	107.353	565.331	49.99	51.93	n.a.
2		9.505	107.381	523.378	50.01	48.07	n.a.
**Total:**			**214.733**	**1088.708**	**100.00**	**100.00**	

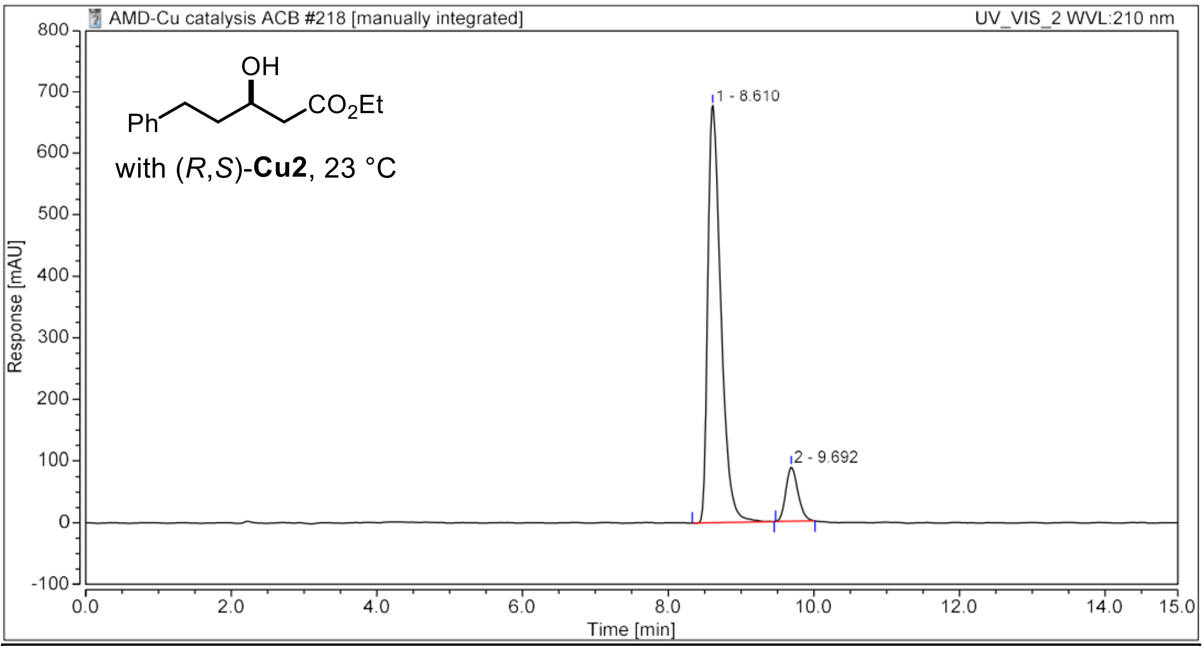

Integration Results

No.	Peak Name	Retention Time min	Area mAU*min	Height mAU	Relative Area %	Relative Height %	Amount n.a.
1		8.610	140.751	677.151	89.24	88.52	n.a.
2		9.692	16.977	87.791	10.76	11.48	n.a.
**Total:**			**157.728**	**764.942**	**100.00**	**100.00**	

Chiral HPLC Chiralpak IB-3 column (2:98 *i*PrOH/hexane, 35 °C, 1 mL/min, detection at 210 nm): $\mathbf{t_{R1}}$ = 8.5 min, $\mathbf{t_{R2}}$ = 9.5 min (ACB Reaction with (*R*,*S*)-**Cu2** at -20 °C).

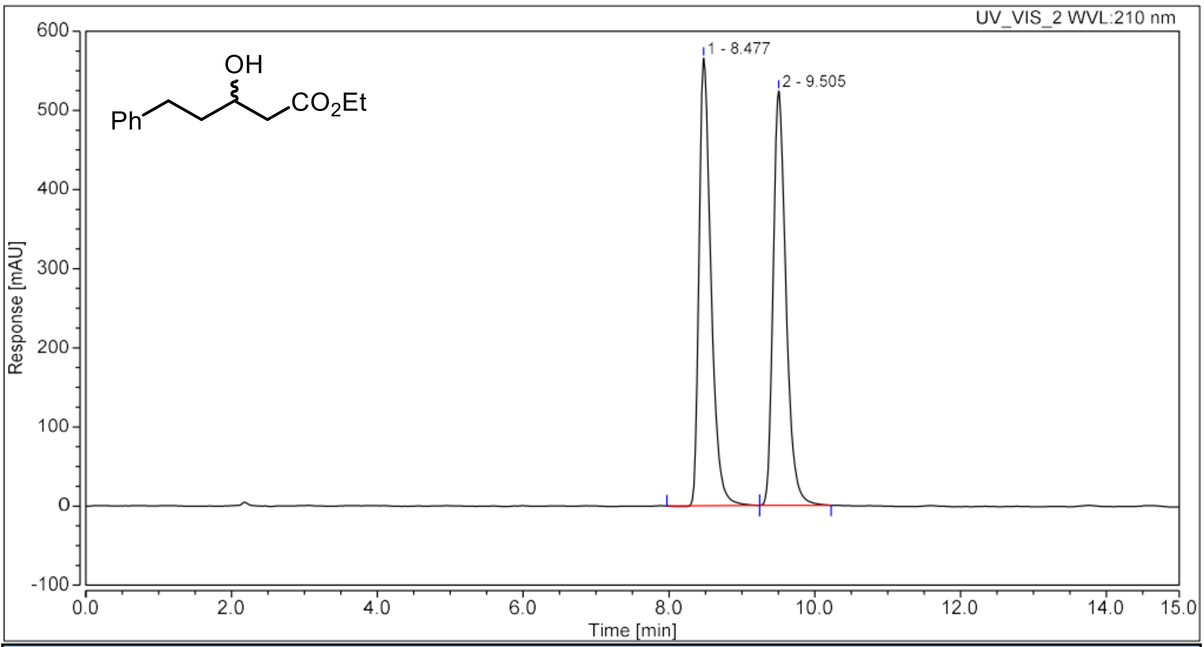

Integration Results

No.	Peak Name	Retention Time min	Area mAU*min	Height mAU	Relative Area %	Relative Height %	Amount n.a.
1		8.477	107.353	565.331	49.99	51.93	n.a.
2		9.505	107.381	523.378	50.01	48.07	n.a.
**Total:**			**214.733**	**1088.708**	**100.00**	**100.00**	

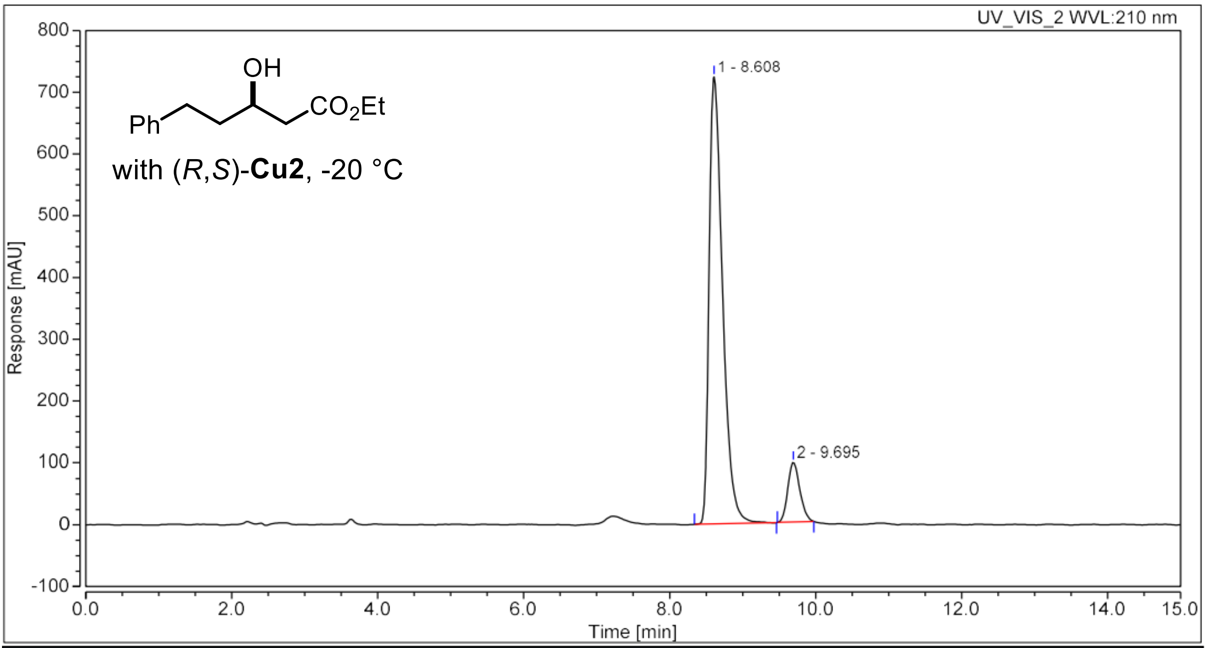

Integration Results

No.	Peak Name	Retention Time min	Area mAU*min	Height mAU	Relative Area %	Relative Height %	Amount n.a.
1		8.608	154.112	723.450	89.31	88.29	n.a.
2		9.695	18.442	95.937	10.69	11.71	n.a.
**Total:**			**172.554**	**819.387**	**100.00**	**100.00**	

Chiral HPLC Chiralpak IB-3 column (2:98 *i*PrOH/hexane, 35 °C, 1 mL/min, detection at 210 nm): $\mathbf{t_{R1}}$ = 8.5 min, $\mathbf{t_{R2}}$ = 9.5 min (ACB Reaction with (*R*,*R*)-**Cu2** at 23 °C).

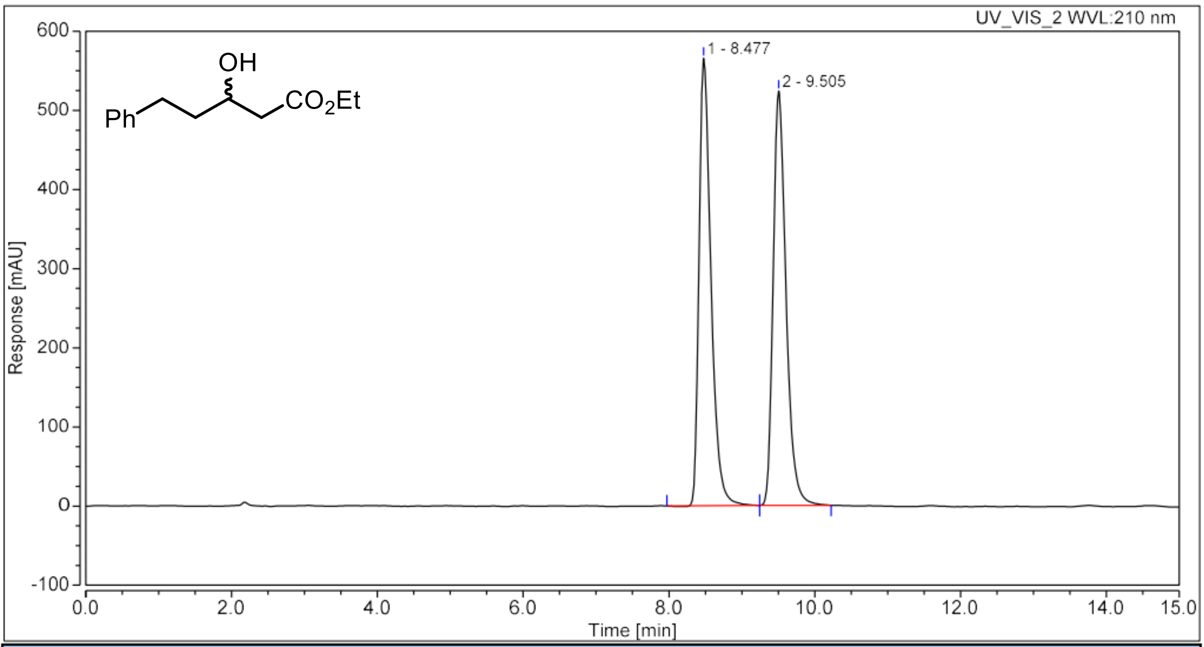

Integration Results							
No.	Peak Name	Retention Time min	Area mAU*min	Height mAU	Relative Area %	Relative Height %	Amount n.a.
1		8.477	107.353	565.331	49.99	51.93	n.a.
2		9.505	107.381	523.378	50.01	48.07	n.a.
**Total:**			**214.733**	**1088.708**	**100.00**	**100.00**	

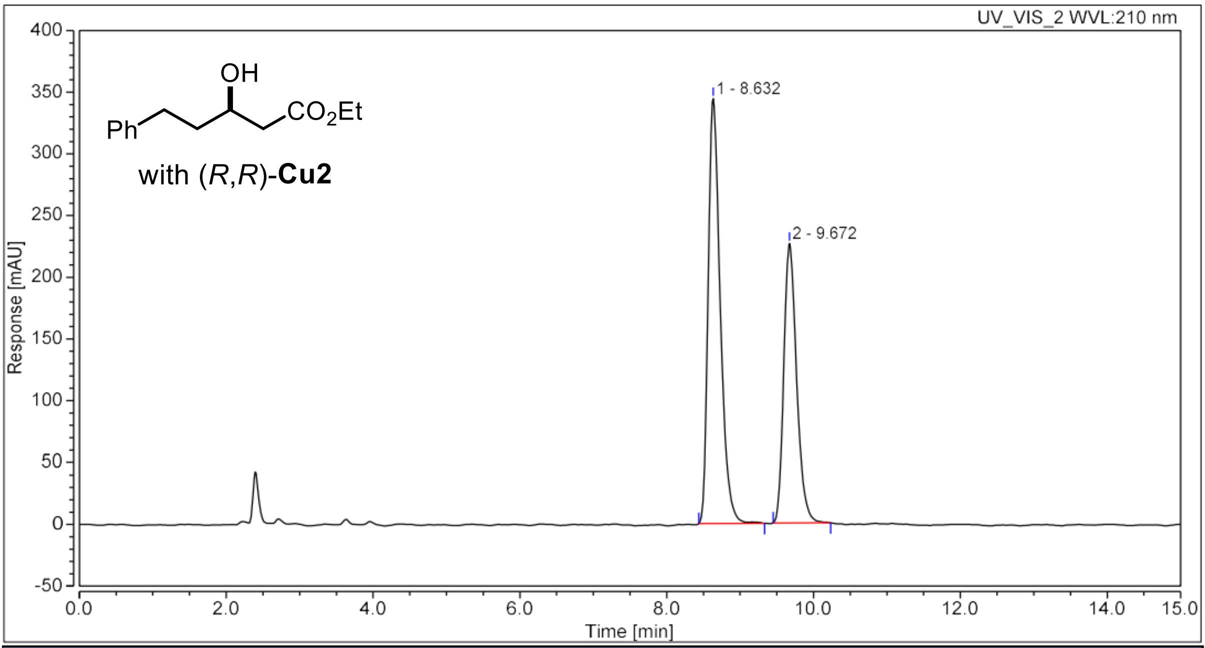

Integration Results							
No.	Peak Name	Retention Time min	Area mAU*min	Height mAU	Relative Area %	Relative Height %	Amount n.a.
1		8.632	65.531	344.021	58.88	60.32	n.a.
2		9.672	45.767	226.306	41.12	39.68	n.a.
**Total:**			**111.297**	**570.327**	**100.00**	**100.00**	

Chiral HPLC Chiralpak IB-3 column (2:98 *i*PrOH/hexane, 35 °C, 1 mL/min, detection at 210 nm): $\mathbf{t_{R1}}$ = 8.5 min, $\mathbf{t_{R2}}$ = 9.5 min (ACB Reaction with (*R*,*S*)-**Cu3** at 23 °C).

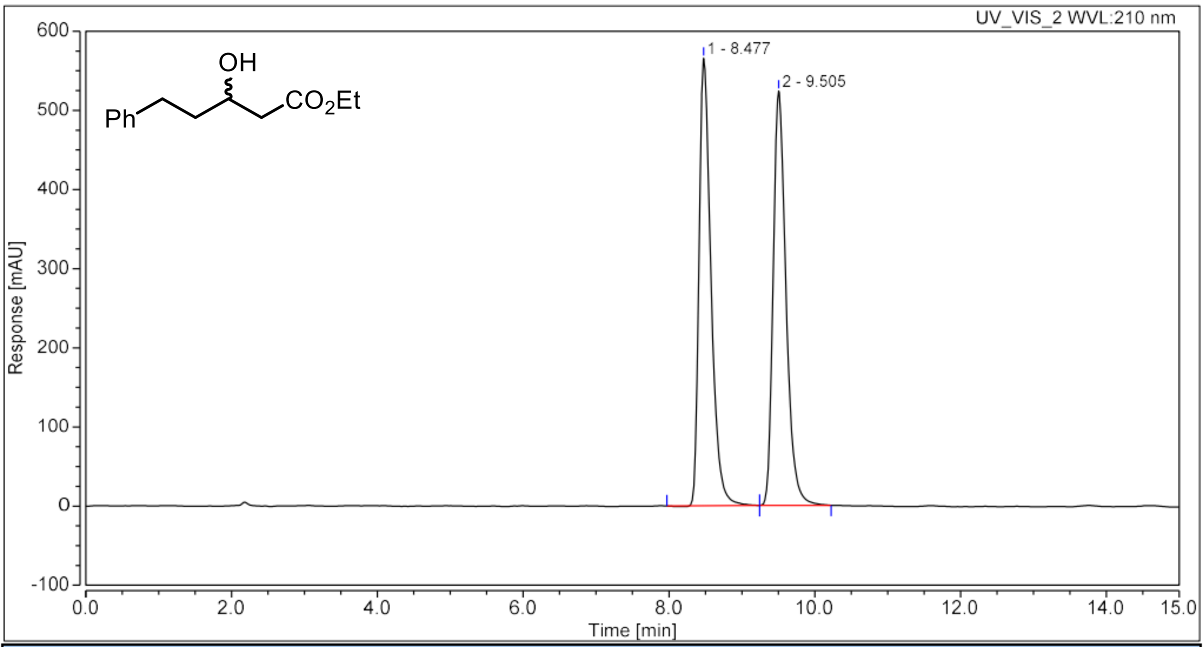

Integration Results							
No.	Peak Name	Retention Time min	Area mAU*min	Height mAU	Relative Area %	Relative Height %	Amount n.a.
1		8.477	107.353	565.331	49.99	51.93	n.a.
2		9.505	107.381	523.378	50.01	48.07	n.a.
Total:			214.733	1088.708	100.00	100.00	

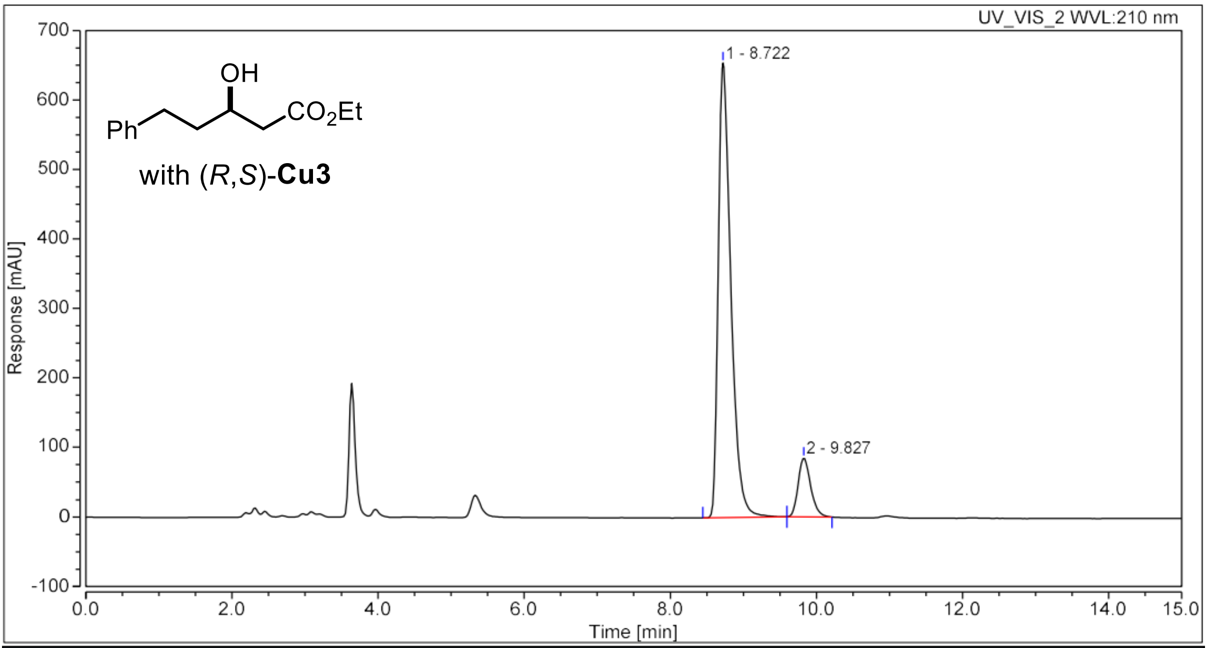

Integration Results							
No.	Peak Name	Retention Time min	Area mAU*min	Height mAU	Relative Area %	Relative Height %	Amount n.a.
1		8.722	130.513	654.274	88.62	88.63	n.a.
2		9.827	16.755	83.952	11.38	11.37	n.a.
Total:			147.268	738.225	100.00	100.00	

Chiral HPLC Chiralpak IB-3 column (2:98 *i*PrOH/hexane, 35 °C, 1 mL/min, detection at 210 nm): $\mathbf{t_{R1}}$ = 8.5 min, $\mathbf{t_{R2}}$ = 9.5 min (ACB Reaction with (*R*,*R*)-**Cu3** at 23 °C).

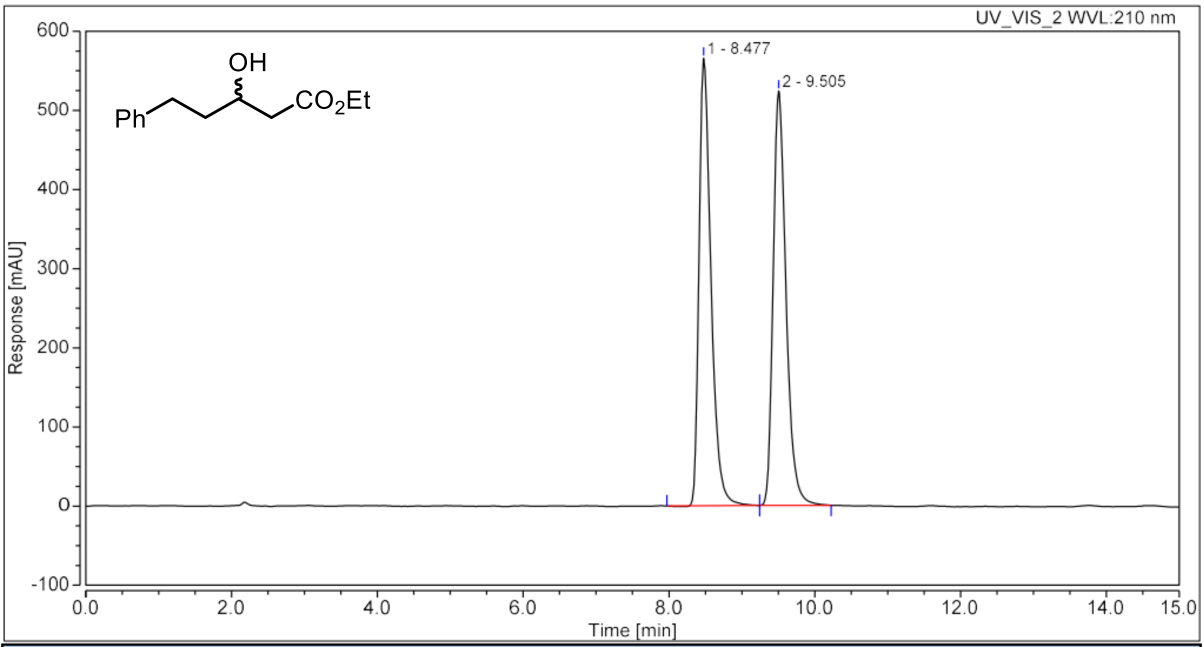

Integration Results

No.	Peak Name	Retention Time min	Area mAU*min	Height mAU	Relative Area %	Relative Height %	Amount n.a.
1		8.477	107.353	565.331	49.99	51.93	n.a.
2		9.505	107.381	523.378	50.01	48.07	n.a.
**Total:**			**214.733**	**1088.708**	**100.00**	**100.00**	

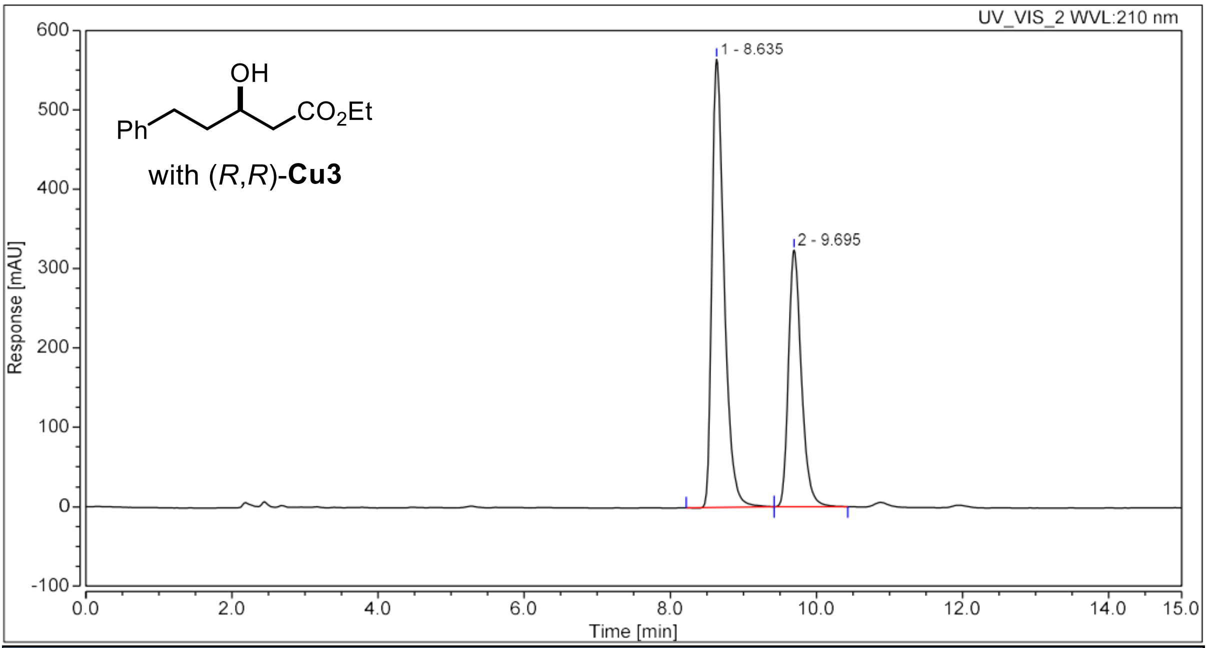

Integration Results

No.	Peak Name	Retention Time min	Area mAU*min	Height mAU	Relative Area %	Relative Height %	Amount n.a.
1		8.635	109.618	564.685	62.38	63.61	n.a.
2		9.695	66.117	323.012	37.62	36.39	n.a.
**Total:**			**175.735**	**887.697**	**100.00**	**100.00**	

Chiral HPLC Chiralpak IB-3 column (2:98 *i*PrOH/hexane, 35 °C, 1 mL/min, detection at 210 nm): $\mathbf{t_{R1}}$ = 8.5 min, $\mathbf{t_{R2}}$ = 9.5 min (ACB Reaction with (*R*,*S*)-**Cu4** at 23 °C).

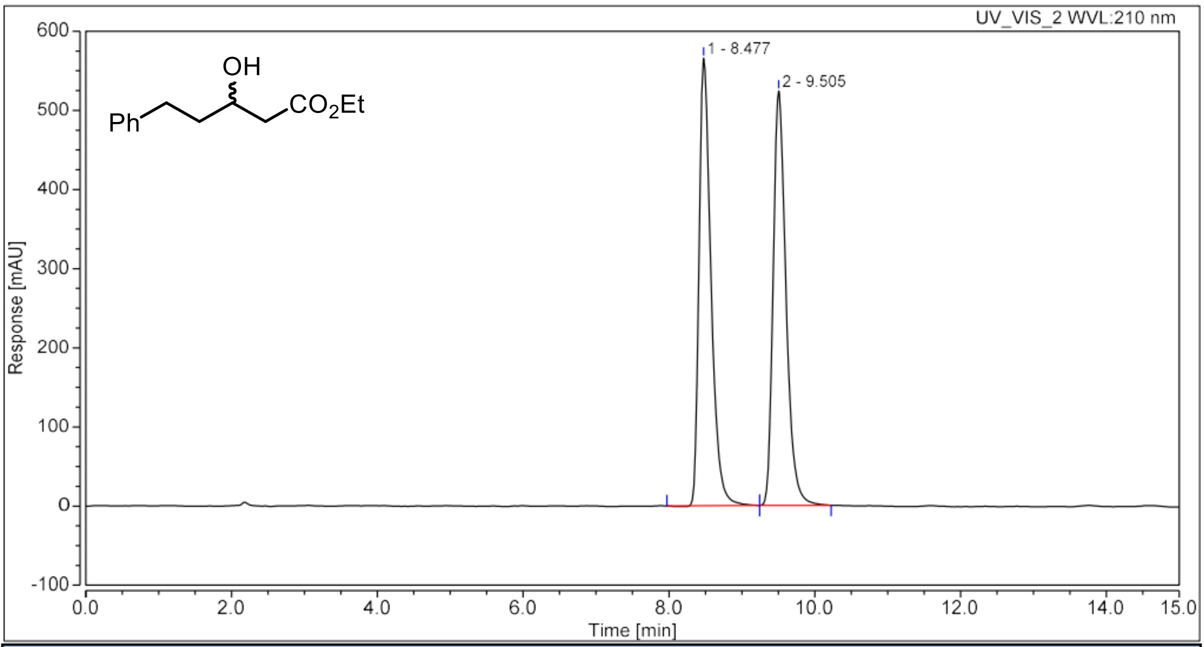

Integration Results

No.	Peak Name	Retention Time min	Area mAU*min	Height mAU	Relative Area %	Relative Height %	Amount n.a.
1		8.477	107.353	565.331	49.99	51.93	n.a.
2		9.505	107.381	523.378	50.01	48.07	n.a.
**Total:**			**214.733**	**1088.708**	**100.00**	**100.00**	

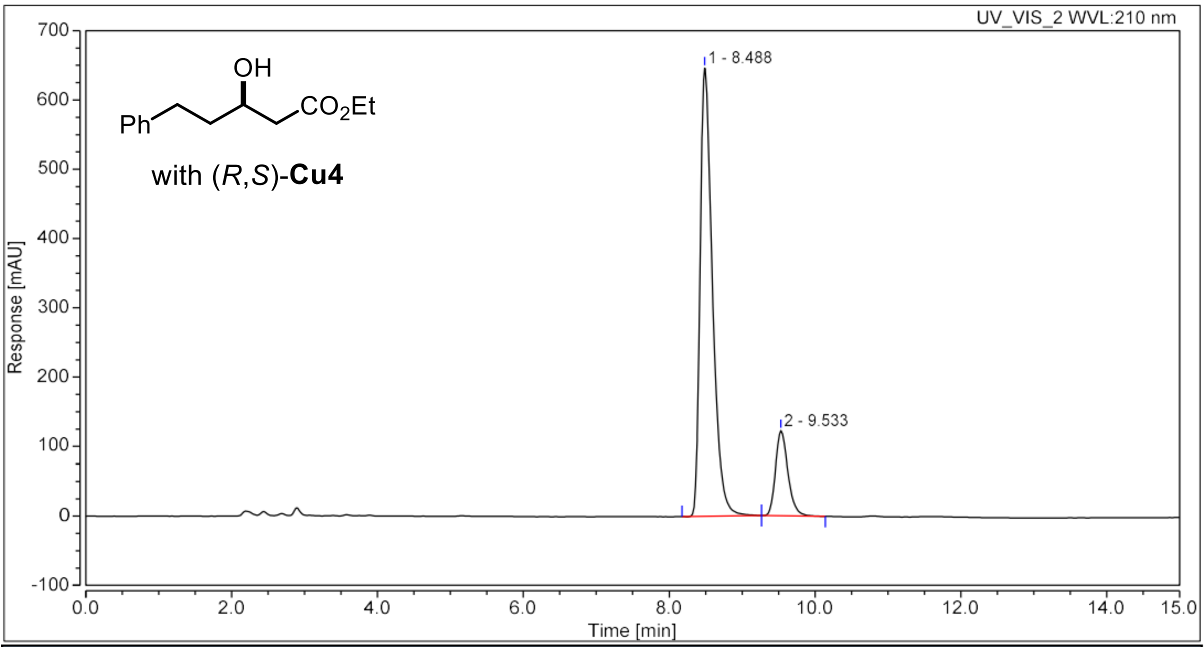

Integration Results

No.	Peak Name	Retention Time min	Area mAU*min	Height mAU	Relative Area %	Relative Height %	Amount n.a.
1		8.488	126.934	646.523	84.00	84.11	n.a.
2		9.533	24.186	122.167	16.00	15.89	n.a.
**Total:**			**151.120**	**768.690**	**100.00**	**100.00**	

Chiral HPLC Chiralpak IB-3 column (2:98 *i*PrOH/hexane, 35 °C, 1 mL/min, detection at 210 nm): $\mathbf{t_{R1}}$ = 8.5 min, $\mathbf{t_{R2}}$ = 9.5 min (ACB Reaction with (*R*,*R*)-**Cu4** at 23 °C).

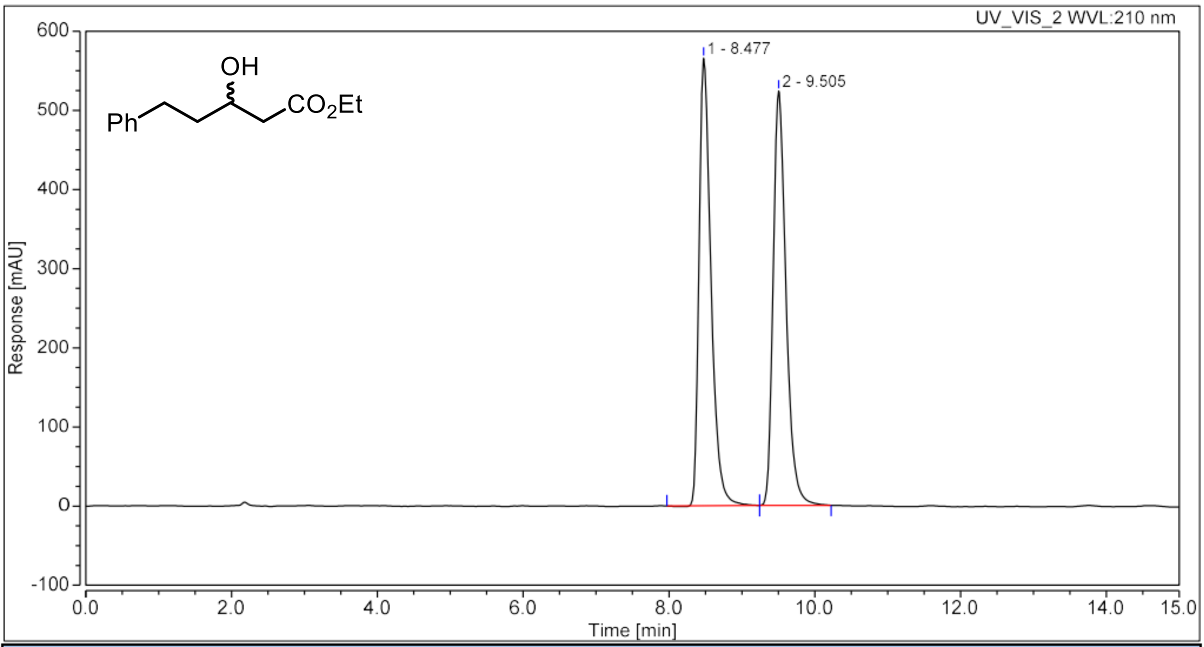

Integration Results							
No.	Peak Name	Retention Time min	Area mAU*min	Height mAU	Relative Area %	Relative Height %	Amount n.a.
1		8.477	107.353	565.331	49.99	51.93	n.a.
2		9.505	107.381	523.378	50.01	48.07	n.a.
**Total:**			**214.733**	**1088.708**	**100.00**	**100.00**	

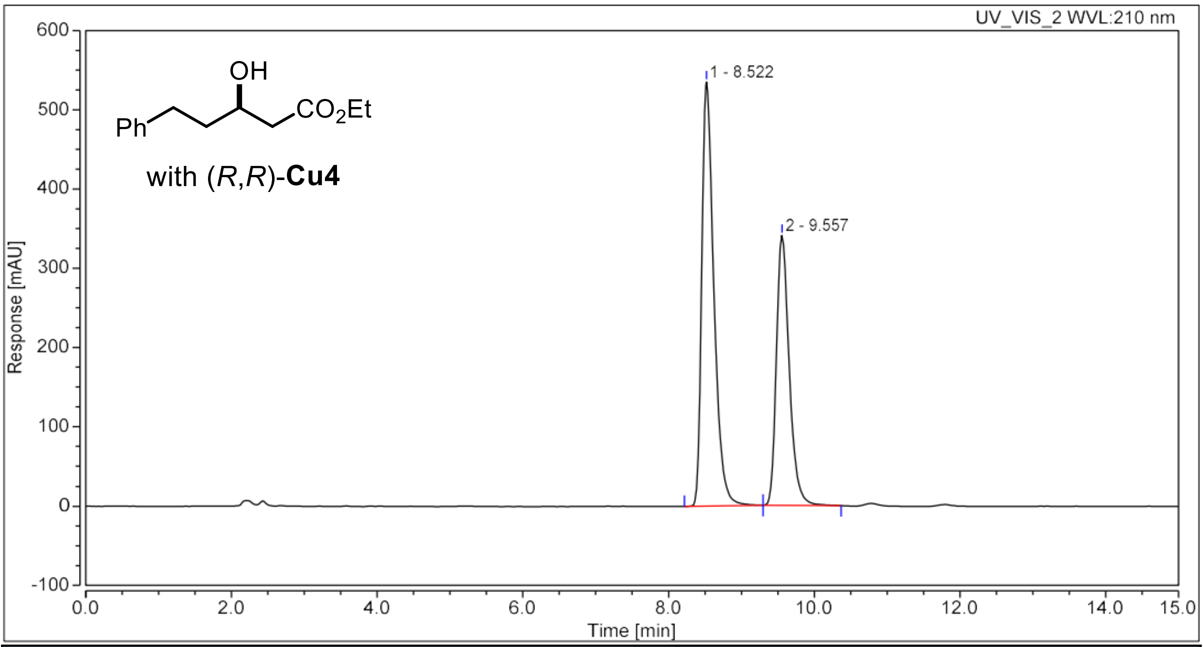

Integration Results							
No.	Peak Name	Retention Time min	Area mAU*min	Height mAU	Relative Area %	Relative Height %	Amount n.a.
1		8.522	102.686	534.793	59.62	61.13	n.a.
2		9.557	69.558	340.119	40.38	38.87	n.a.
**Total:**			**172.245**	**874.911**	**100.00**	**100.00**	

Chiral HPLC Chiralpak IB-3 column (2:98 *i*PrOH/hexane, 35 °C, 1 mL/min, detection at 210 nm): $\mathbf{t_{R1}}$ = 8.5 min, $\mathbf{t_{R2}}$ = 9.5 min (ACB Reaction with (*R*,*S*)-**Cu5** at 23 °C).

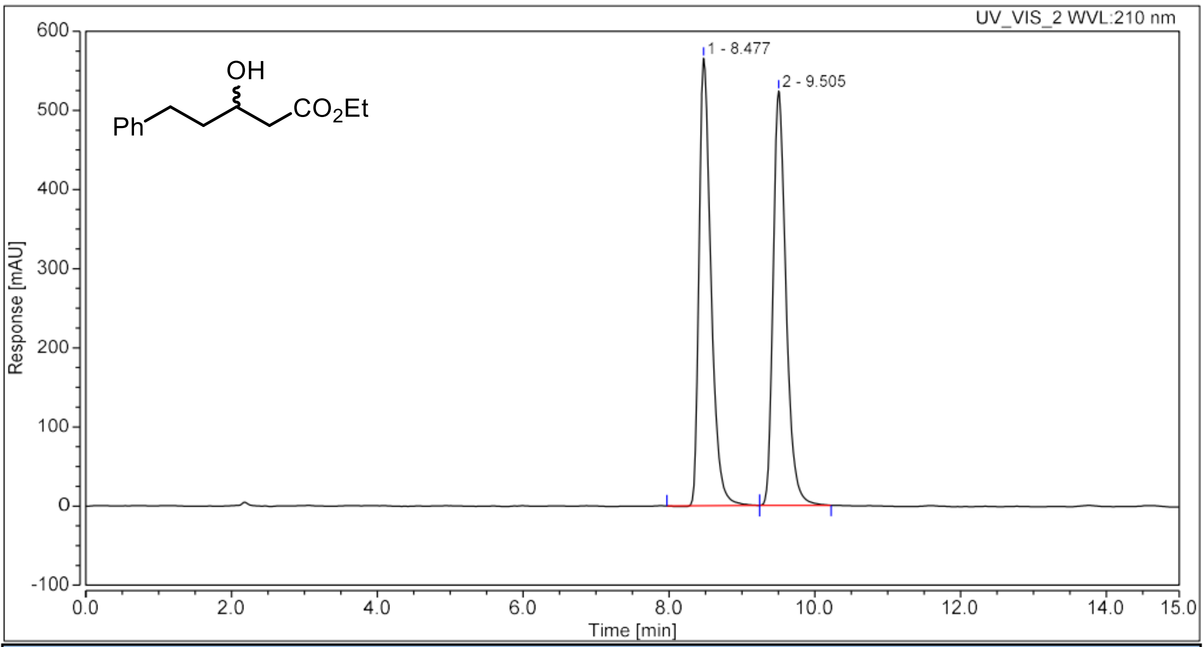

Integration Results							
No.	Peak Name	Retention Time min	Area mAU*min	Height mAU	Relative Area %	Relative Height %	Amount n.a.
1		8.477	107.353	565.331	49.99	51.93	n.a.
2		9.505	107.381	523.378	50.01	48.07	n.a.
**Total:**			**214.733**	**1088.708**	**100.00**	**100.00**	

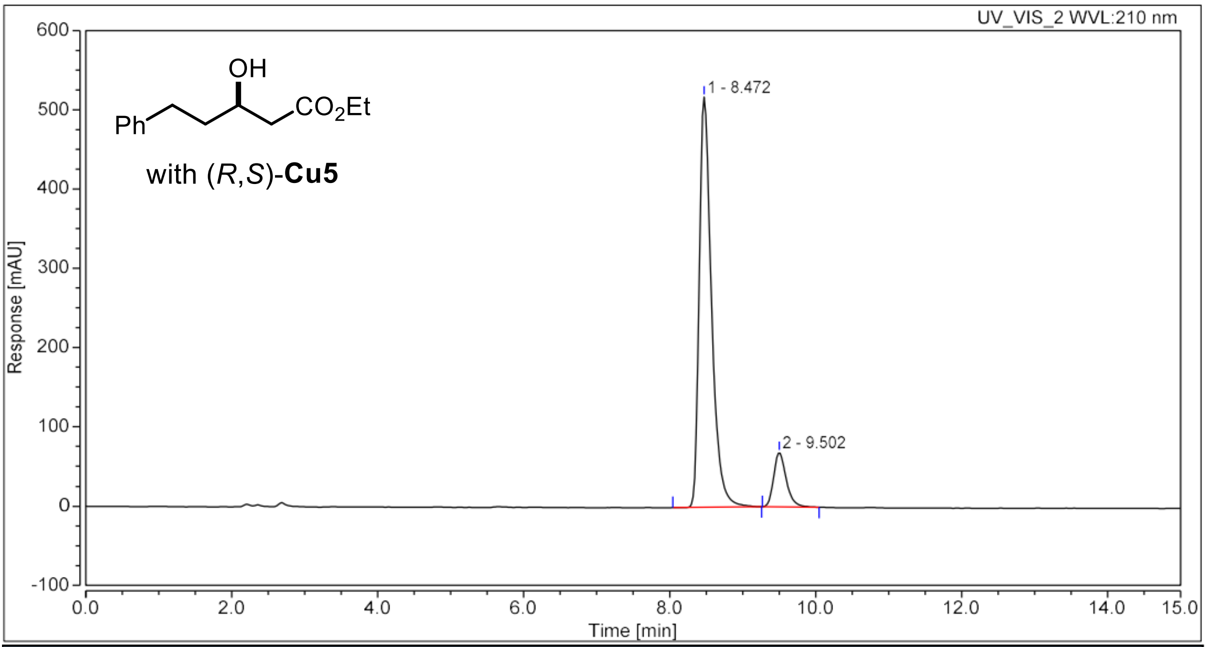

Integration Results							
No.	Peak Name	Retention Time min	Area mAU*min	Height mAU	Relative Area %	Relative Height %	Amount n.a.
1		8.472	100.598	517.269	88.12	88.41	n.a.
2		9.502	13.559	67.806	11.88	11.59	n.a.
**Total:**			**114.157**	**585.075**	**100.00**	**100.00**	

Chiral HPLC Chiralpak IB-3 column (2:98 *i*PrOH/hexane, 35 °C, 1 mL/min, detection at 210 nm): $\mathbf{t_{R1}}$ = 8.5 min, $\mathbf{t_{R2}}$ = 9.5 min (ACB Reaction with (*R*,*R*)-**Cu5** at 23 °C).

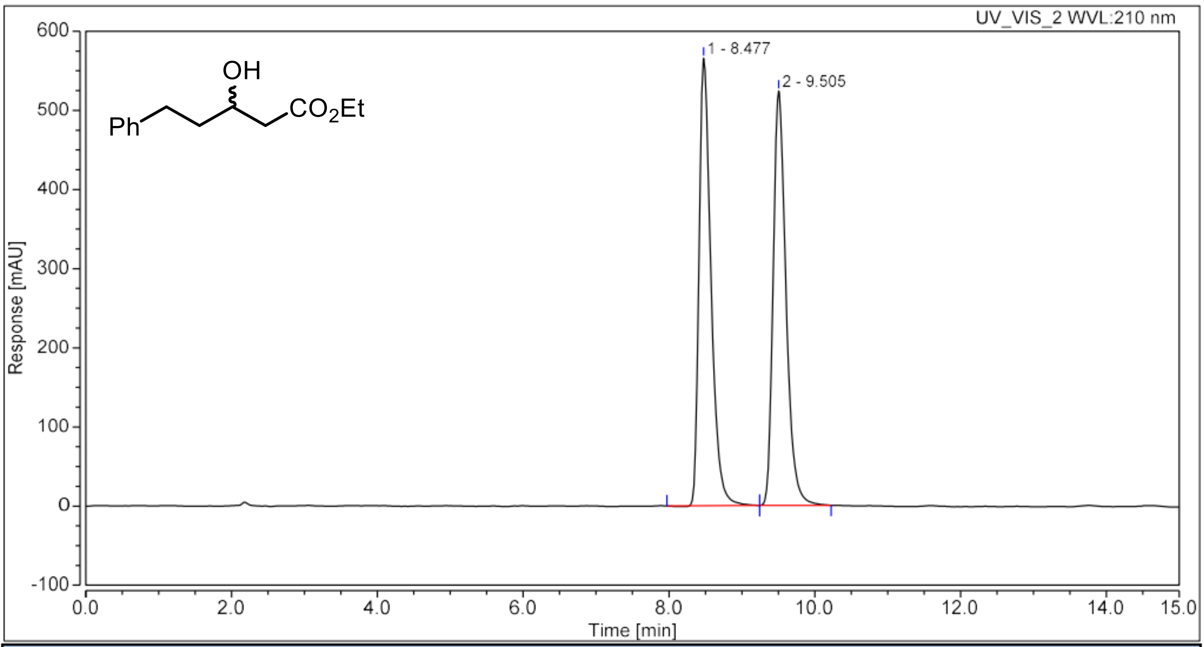

Integration Results

No.	Peak Name	Retention Time min	Area mAU*min	Height mAU	Relative Area %	Relative Height %	Amount n.a.
1		8.477	107.353	565.331	49.99	51.93	n.a.
2		9.505	107.381	523.378	50.01	48.07	n.a.
**Total:**			**214.733**	**1088.708**	**100.00**	**100.00**	

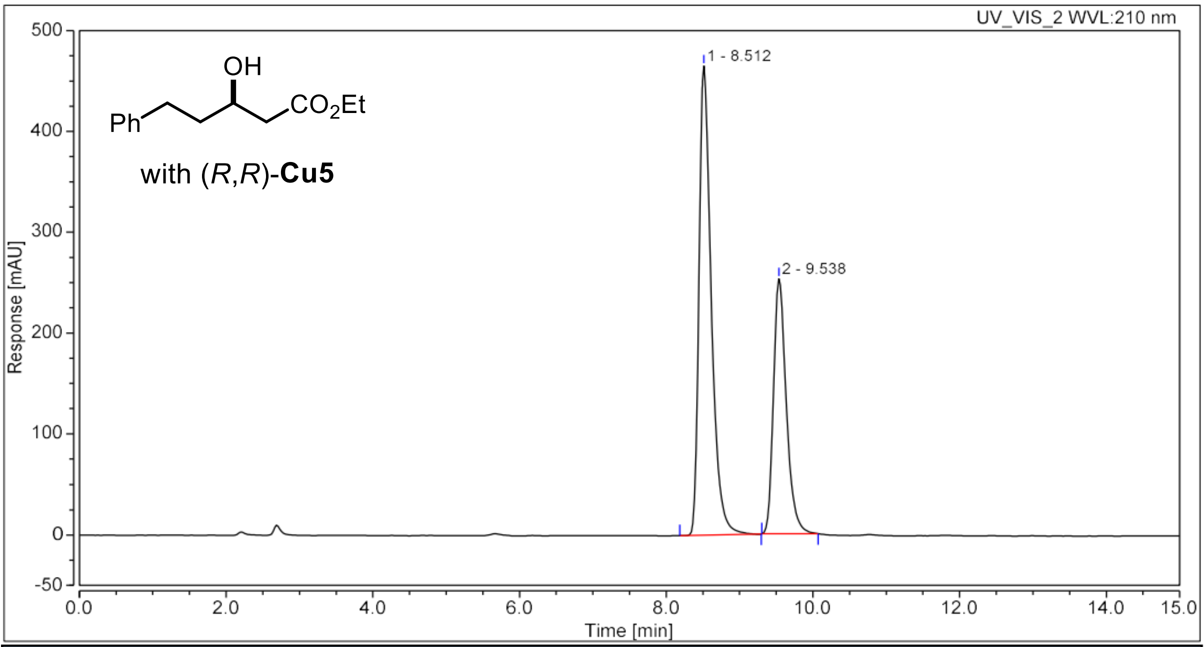

Integration Results

No.	Peak Name	Retention Time min	Area mAU*min	Height mAU	Relative Area %	Relative Height %	Amount n.a.
1		8.512	89.788	465.087	63.67	64.82	n.a.
2		9.538	51.224	252.444	36.33	35.18	n.a.
**Total:**			**141.012**	**717.531**	**100.00**	**100.00**	

Chiral HPLC Chiralpak IB-3 column (2:98 *i*PrOH/hexane, 35 °C, 1 mL/min, detection at 210 nm): $\mathbf{t_{R1}}$ = 8.5 min, $\mathbf{t_{R2}}$ = 9.5 min (ACB Reaction with (*R*,*S*)-**Cu6** at 23 °C).

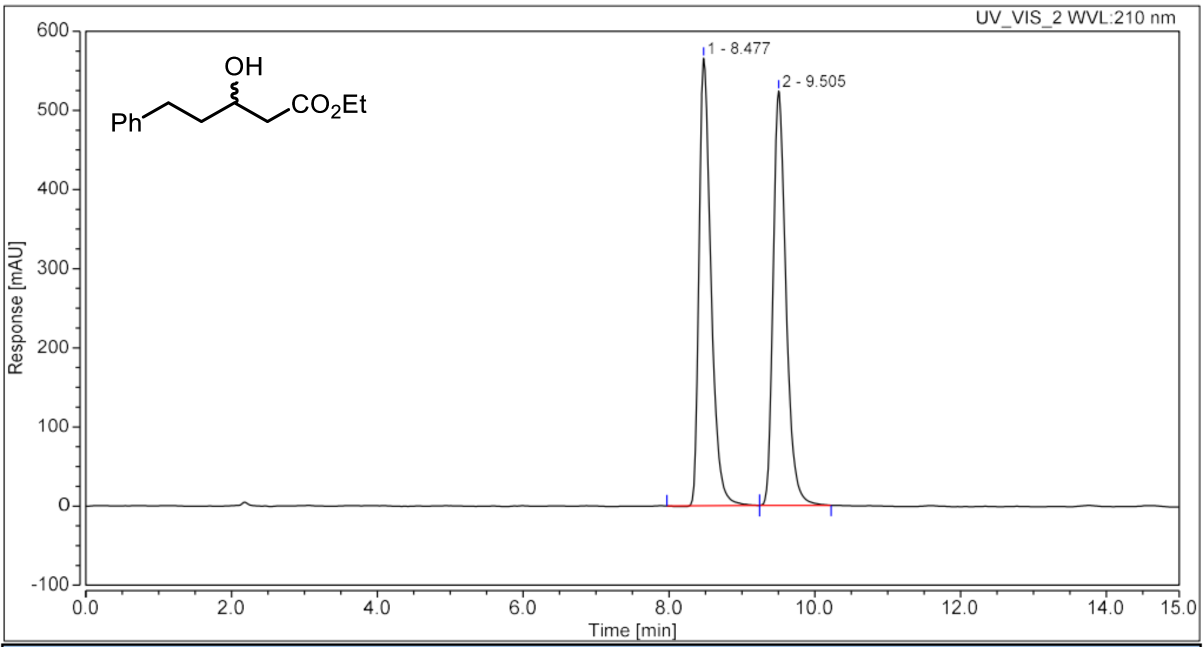

Integration Results

No.	Peak Name	Retention Time<br>min	Area<br>mAU*min	Height<br>mAU	Relative Area<br>%	Relative Height<br>%	Amount<br>n.a.
1		8.477	107.353	565.331	49.99	51.93	n.a.
2		9.505	107.381	523.378	50.01	48.07	n.a.
**Total:**			**214.733**	**1088.708**	**100.00**	**100.00**	

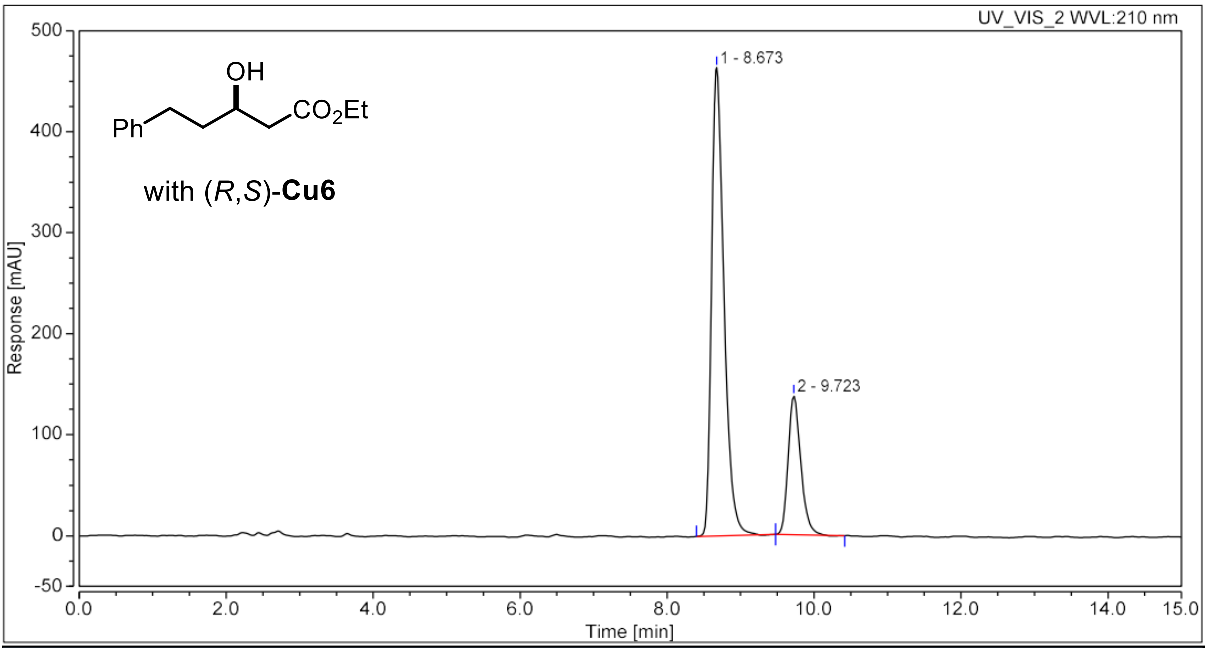

Integration Results

No.	Peak Name	Retention Time<br>min	Area<br>mAU*min	Height<br>mAU	Relative Area<br>%	Relative Height<br>%	Amount<br>n.a.
1		8.673	90.574	463.467	76.83	77.18	n.a.
2		9.723	27.322	137.004	23.17	22.82	n.a.
**Total:**			**117.896**	**600.471**	**100.00**	**100.00**	

Chiral HPLC Chiralpak IB-3 column (2:98 *i*PrOH/hexane, 35 °C, 1 mL/min, detection at 210 nm): $\mathbf{t_{R1}}$ = 8.5 min, $\mathbf{t_{R2}}$ = 9.5 min (ACB Reaction with (*R*,*R*)-**Cu6** at 23 °C).

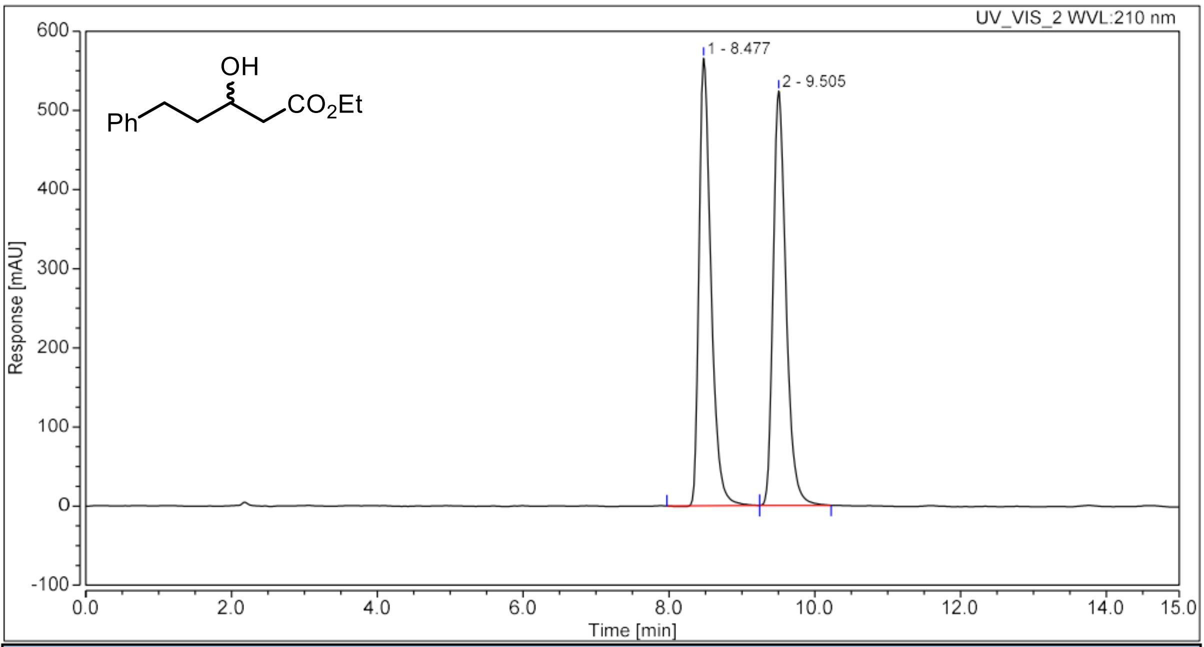

Integration Results

No.	Peak Name	Retention Time min	Area mAU*min	Height mAU	Relative Area %	Relative Height %	Amount n.a.
1		8.477	107.353	565.331	49.99	51.93	n.a.
2		9.505	107.381	523.378	50.01	48.07	n.a.
**Total:**			**214.733**	**1088.708**	**100.00**	**100.00**	

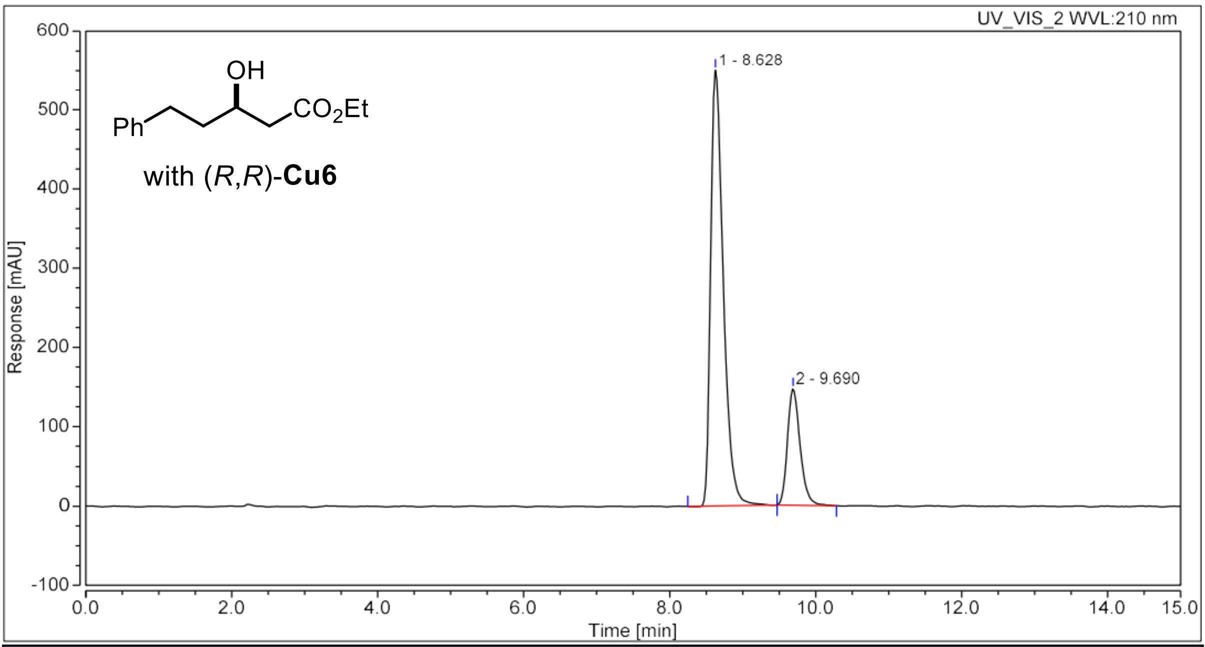

Integration Results

No.	Peak Name	Retention Time min	Area mAU*min	Height mAU	Relative Area %	Relative Height %	Amount n.a.
1		8.628	110.354	550.659	78.93	79.00	n.a.
2		9.690	29.451	146.418	21.07	21.00	n.a.
**Total:**			**139.806**	**697.076**	**100.00**	**100.00**	

Chiral HPLC Chiralpak IB-3 column (2:98 *i*PrOH/hexane, 35 °C, 1 mL/min, detection at 210 nm): $\mathbf{t_{R1}}$ = 8.5 min, $\mathbf{t_{R2}}$ = 9.5 min (ACB Reaction with (*R*,*R*)-**Cu7** at 23 °C).

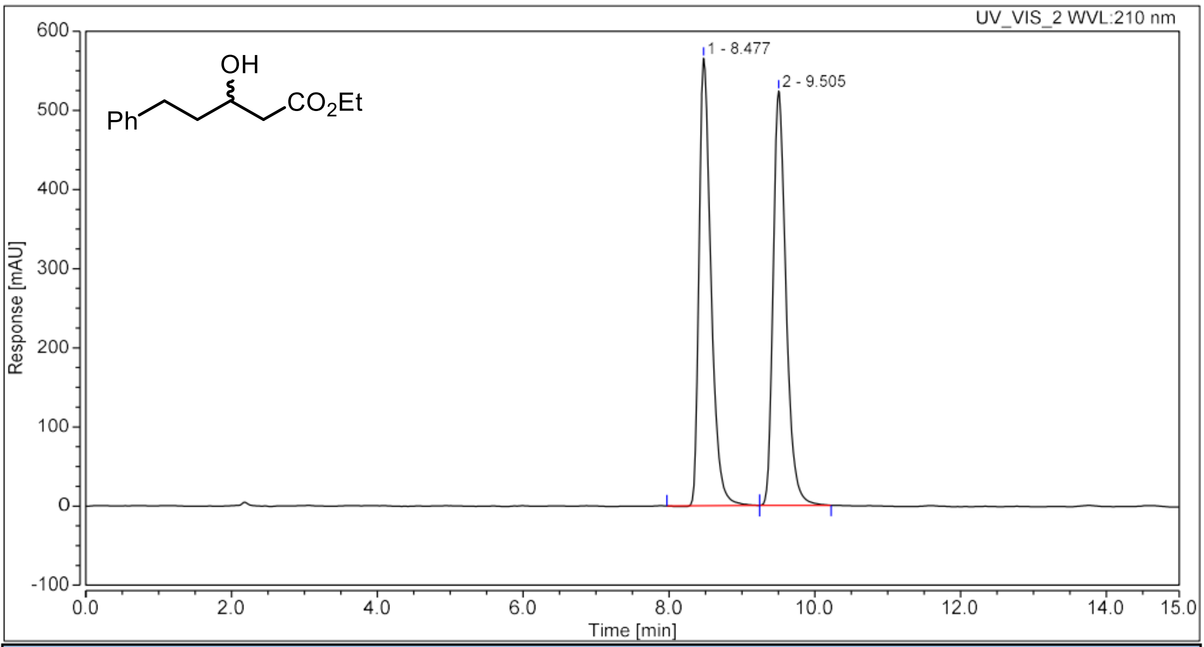

Integration Results							
No.	Peak Name	Retention Time min	Area mAU*min	Height mAU	Relative Area %	Relative Height %	Amount n.a.
1		8.477	107.353	565.331	49.99	51.93	n.a.
2		9.505	107.381	523.378	50.01	48.07	n.a.
**Total:**			**214.733**	**1088.708**	**100.00**	**100.00**	

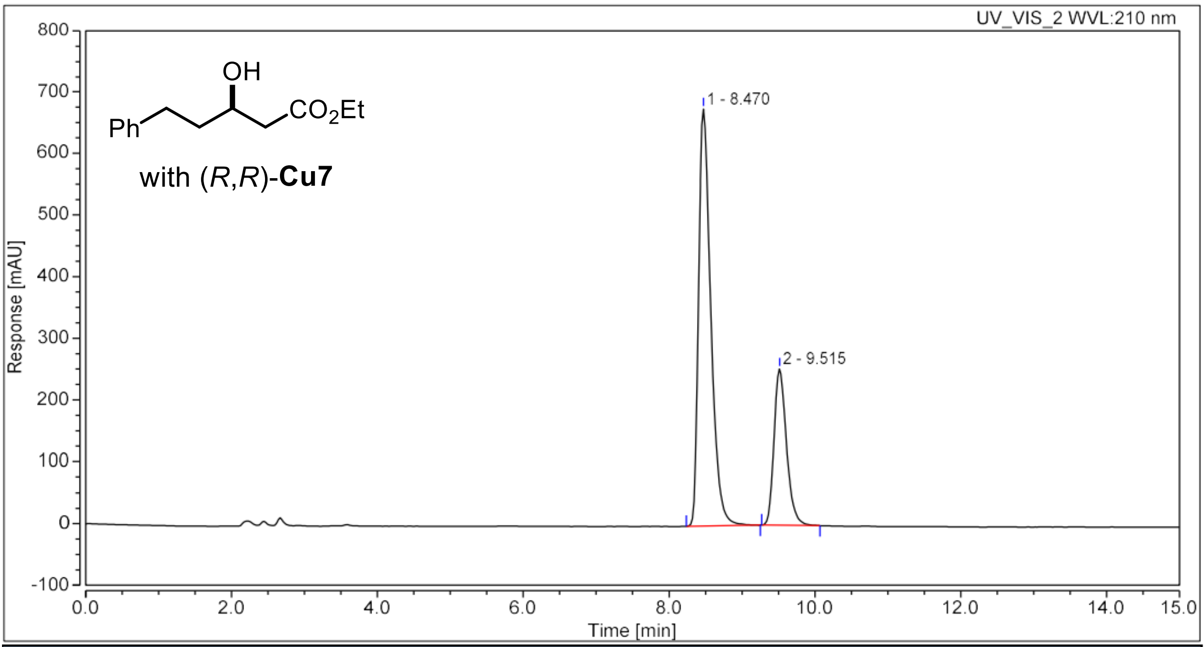

Integration Results							
No.	Peak Name	Retention Time min	Area mAU*min	Height mAU	Relative Area %	Relative Height %	Amount n.a.
1		8.470	130.662	676.133	72.24	72.78	n.a.
2		9.515	50.200	252.905	27.76	27.22	n.a.
**Total:**			**180.862**	**929.038**	**100.00**	**100.00**	

Chiral HPLC Chiralpak IB-3 column (2:98 *i*PrOH/hexane, 35 °C, 1 mL/min, detection at 210 nm): $\mathbf{t_{R1}}$ = 8.5 min, $\mathbf{t_{R2}}$ = 9.5 min (ACB Reaction with (*R*,*S*)-**Cu7** at 23 °C).

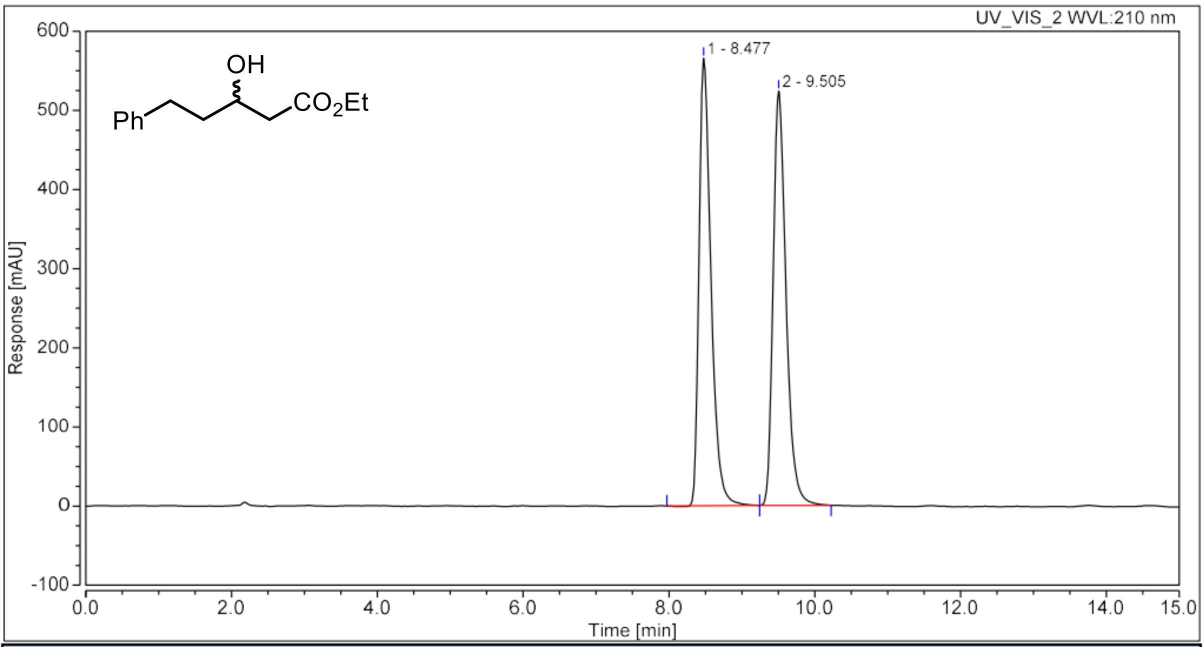

Integration Results

No.	Peak Name	Retention Time min	Area mAU*min	Height mAU	Relative Area %	Relative Height %	Amount n.a.
1		8.477	107.353	565.331	49.99	51.93	n.a.
2		9.505	107.381	523.378	50.01	48.07	n.a.
**Total:**			**214.733**	**1088.708**	**100.00**	**100.00**	

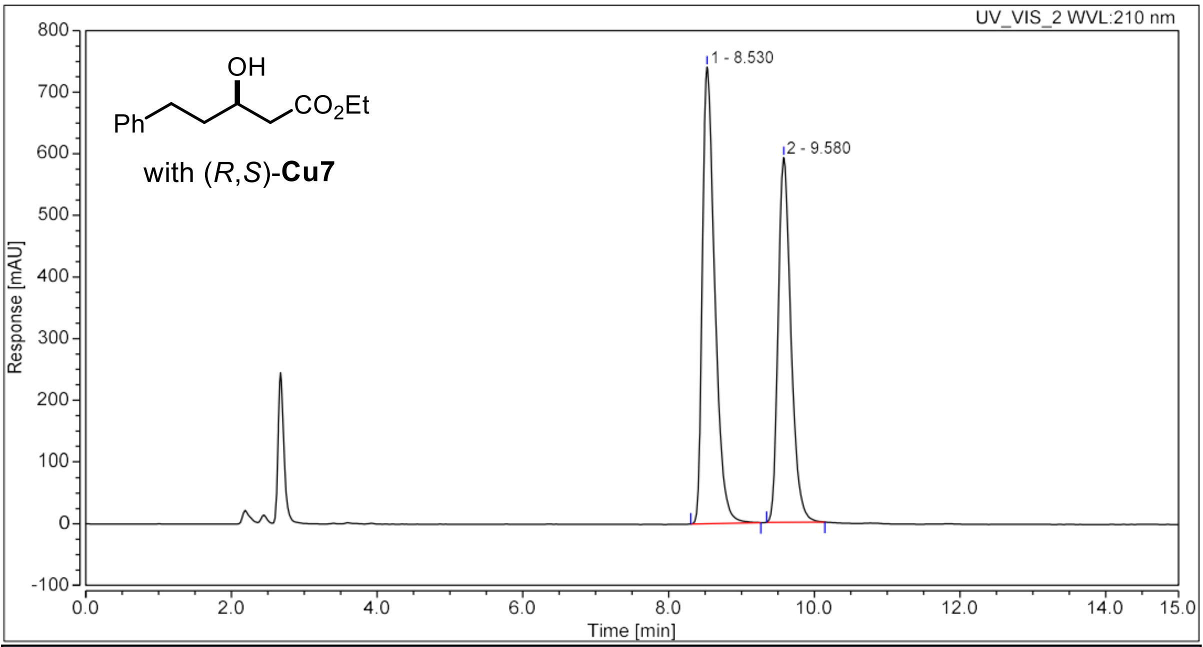

Integration Results

No.	Peak Name	Retention Time min	Area mAU*min	Height mAU	Relative Area %	Relative Height %	Amount n.a.
1		8.530	145.601	740.335	54.76	55.61	n.a.
2		9.580	120.297	590.995	45.24	44.39	n.a.
**Total:**			**265.898**	**1331.330**	**100.00**	**100.00**	

Chiral HPLC Chiralpak IB-3 column (2:98 *i*PrOH/hexane, 35 °C, 1 mL/min, detection at 210 nm): $\mathbf{t_{R1}}$ = 8.5 min, $\mathbf{t_{R2}}$ = 9.5 min (ACB Reaction with (*R*,*S*)-**Cu8** at 23 °C).

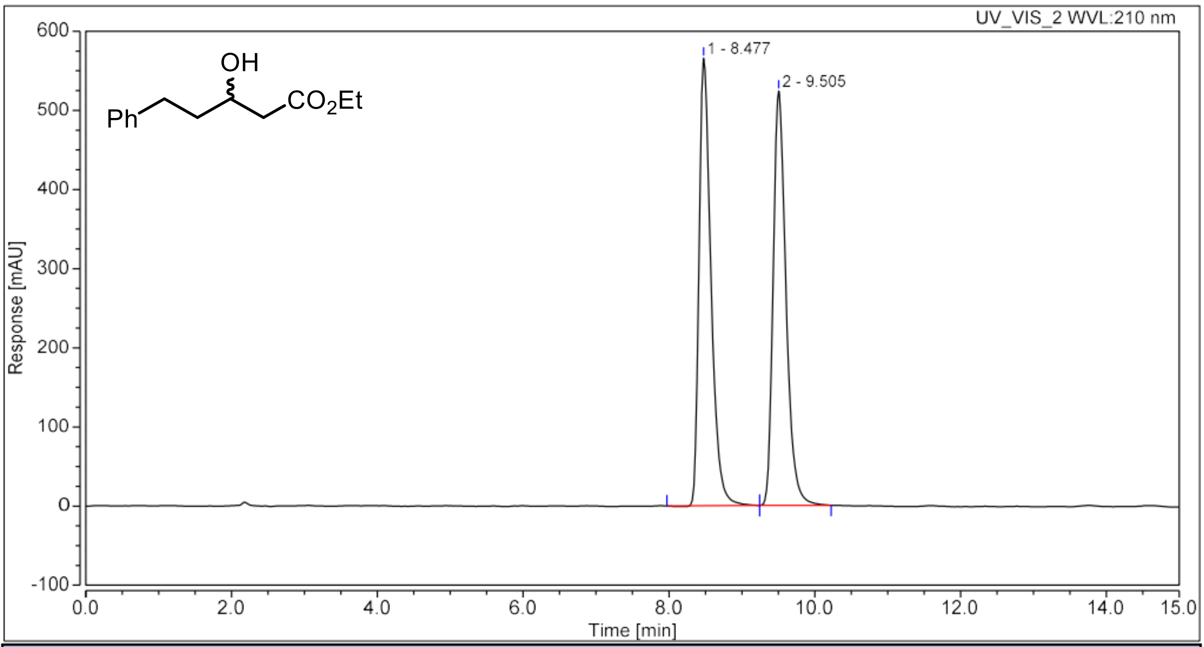

Integration Results							
No.	Peak Name	Retention Time min	Area mAU*min	Height mAU	Relative Area %	Relative Height %	Amount n.a.
1		8.477	107.353	565.331	49.99	51.93	n.a.
2		9.505	107.381	523.378	50.01	48.07	n.a.
**Total:**			**214.733**	**1088.708**	**100.00**	**100.00**	

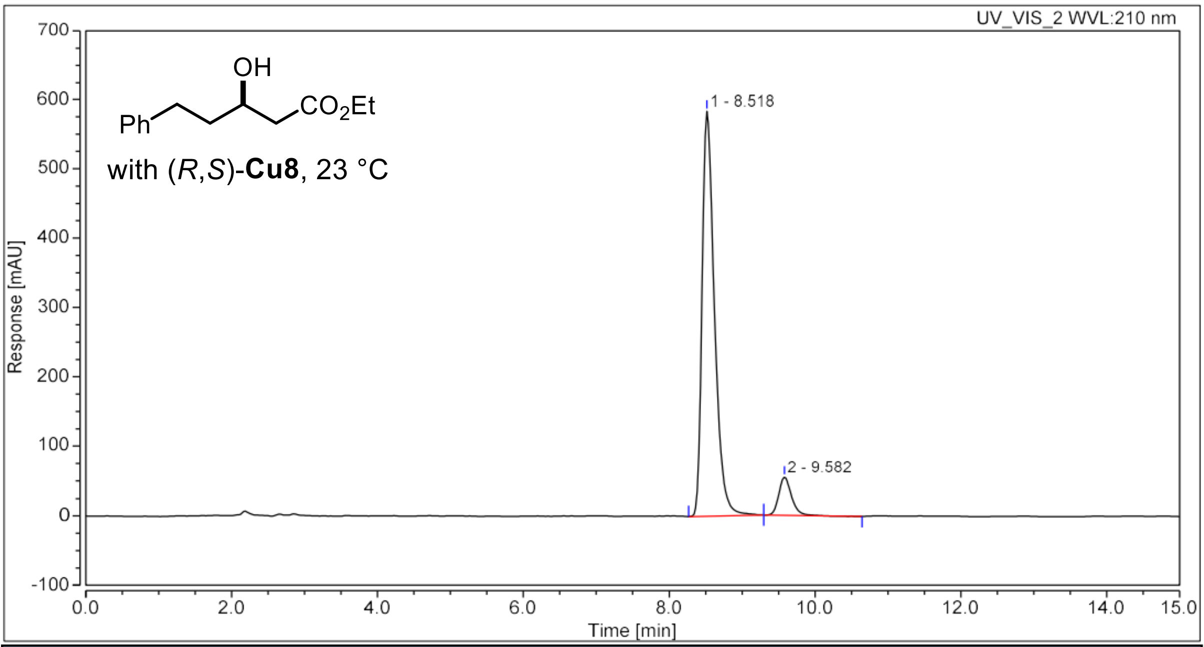

Integration Results							
No.	Peak Name	Retention Time min	Area mAU*min	Height mAU	Relative Area %	Relative Height %	Amount n.a.
1		8.518	114.491	583.290	91.23	91.49	n.a.
2		9.582	11.008	54.279	8.77	8.51	n.a.
**Total:**			**125.500**	**637.569**	**100.00**	**100.00**	

Chiral HPLC Chiralpak IB-3 column (2:98 *i*PrOH/hexane, 35 °C, 1 mL/min, detection at 210 nm): $\mathbf{t_{R1}}$ = 8.5 min, $\mathbf{t_{R2}}$ = 9.5 min (ACB Reaction with (*R*,*S*)-**Cu8** at -20 °C).

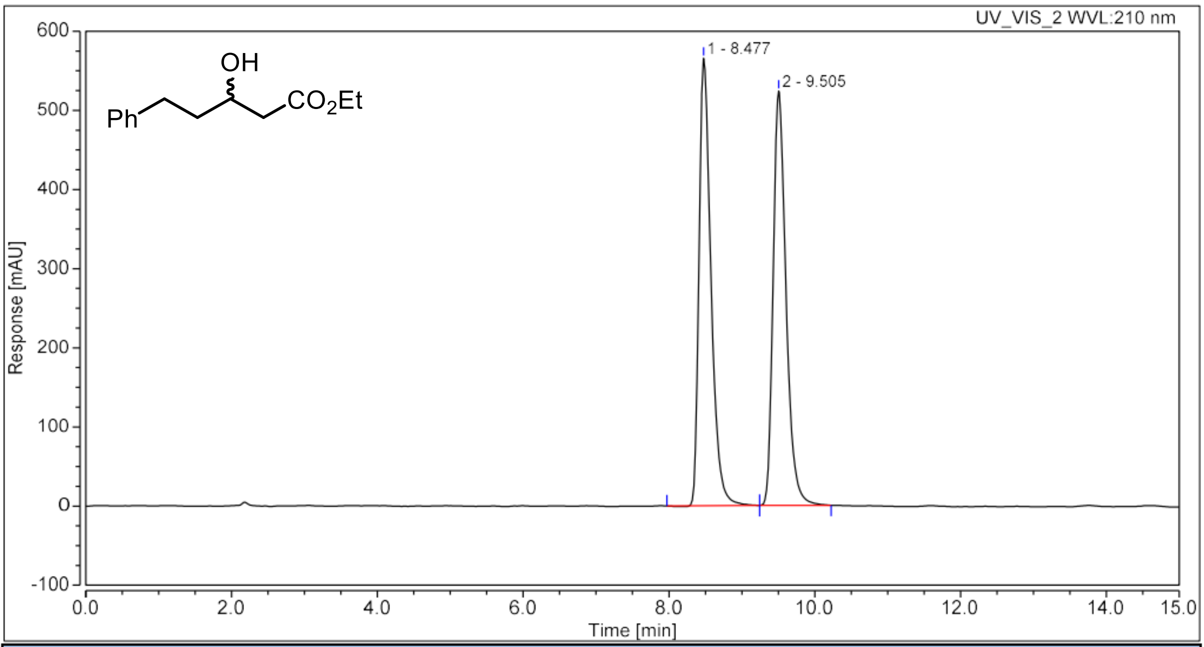

Integration Results

No.	Peak Name	Retention Time min	Area mAU*min	Height mAU	Relative Area %	Relative Height %	Amount n.a.
1		8.477	107.353	565.331	49.99	51.93	n.a.
2		9.505	107.381	523.378	50.01	48.07	n.a.
**Total:**			**214.733**	**1088.708**	**100.00**	**100.00**	

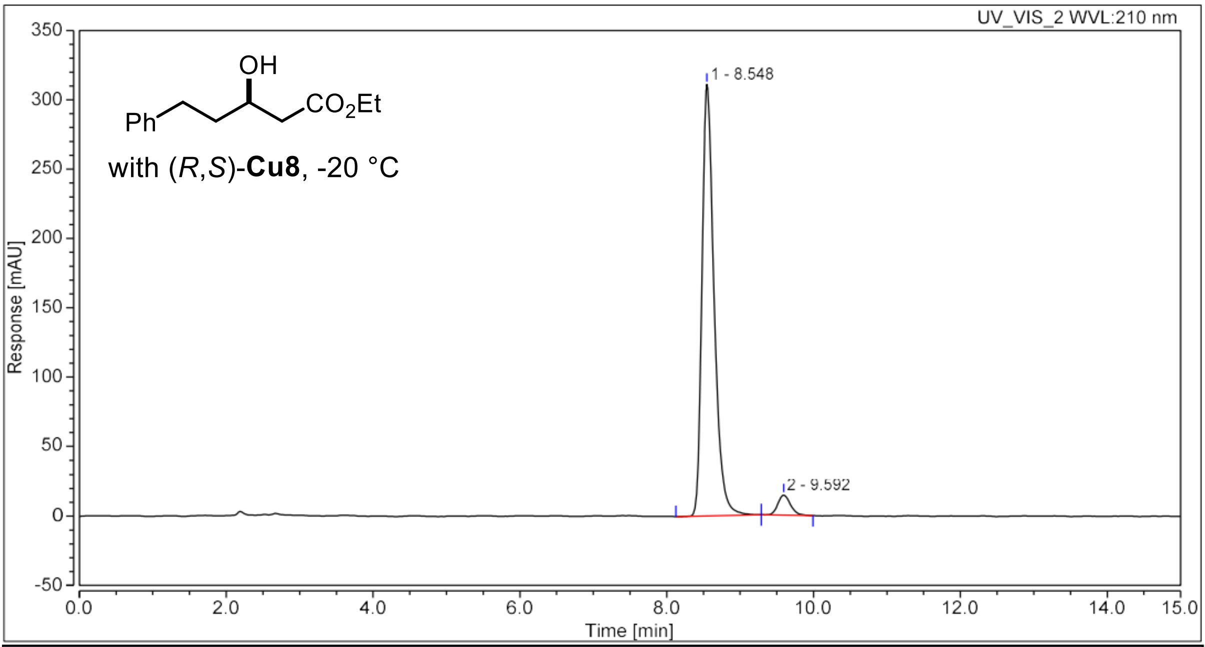

Integration Results

No.	Peak Name	Retention Time min	Area mAU*min	Height mAU	Relative Area %	Relative Height %	Amount n.a.
1		8.548	59.240	310.976	95.33	95.53	n.a.
2		9.592	2.904	14.568	4.67	4.47	n.a.
**Total:**			**62.144**	**325.544**	**100.00**	**100.00**	

Chiral HPLC Chiralpak IB-3 column (2:98 *i*PrOH/hexane, 35 °C, 1 mL/min, detection at 210 nm): $\mathbf{t_{R1}}$ = 8.5 min, $\mathbf{t_{R2}}$ = 9.5 min (ACB Reaction with (*R*,*R*)-**Cu8** at 23 °C).

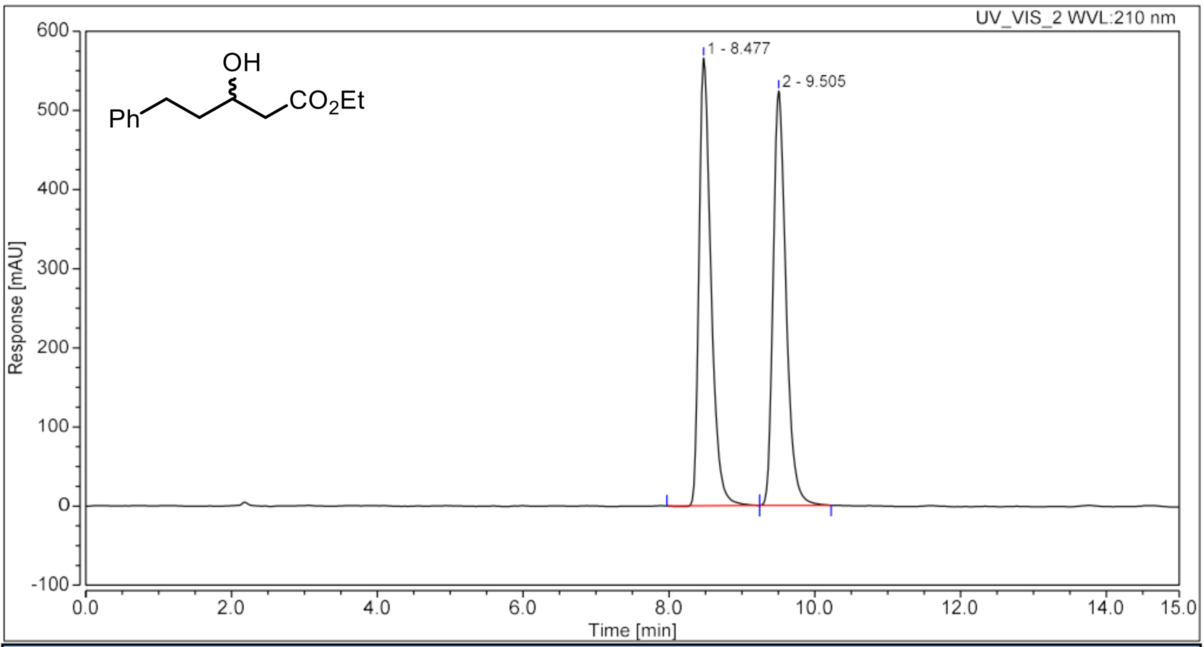

Integration Results

No.	Peak Name	Retention Time min	Area mAU*min	Height mAU	Relative Area %	Relative Height %	Amount n.a.
1		8.477	107.353	565.331	49.99	51.93	n.a.
2		9.505	107.381	523.378	50.01	48.07	n.a.
**Total:**			**214.733**	**1088.708**	**100.00**	**100.00**	

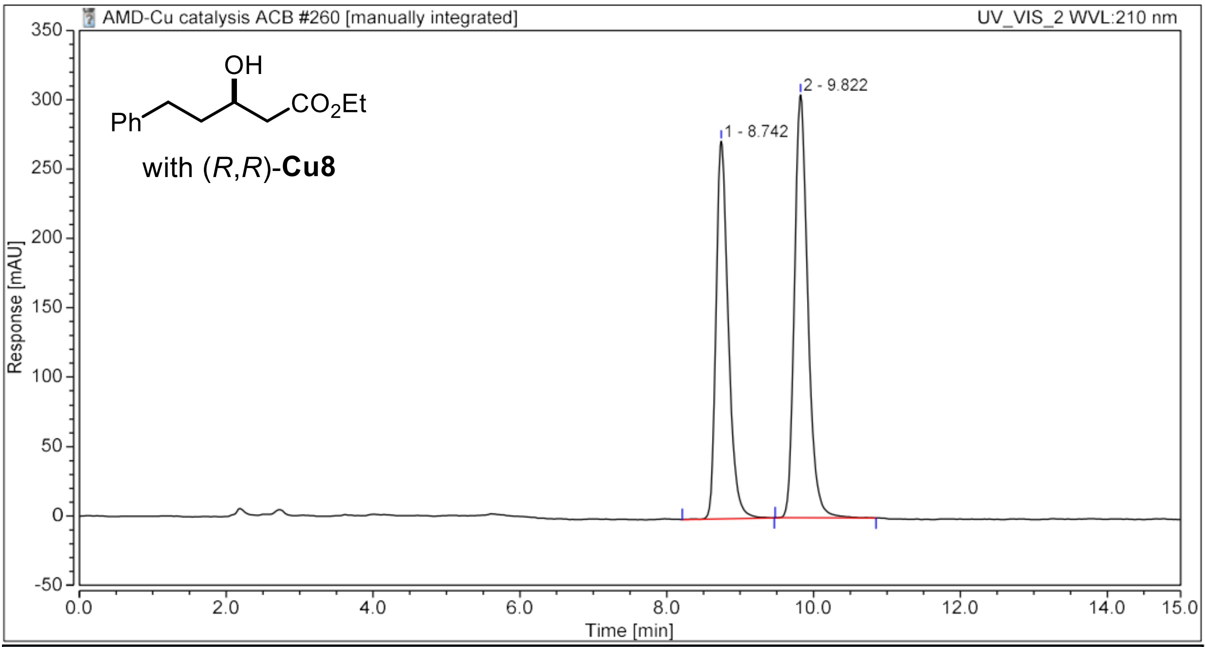

Integration Results

No.	Peak Name	Retention Time min	Area mAU*min	Height mAU	Relative Area %	Relative Height %	Amount n.a.
1		8.742	52.584	271.999	44.95	47.16	n.a.
2		9.822	64.390	304.742	55.05	52.84	n.a.
**Total:**			**116.974**	**576.741**	**100.00**	**100.00**	

Chiral HPLC Chiralpak IB-3 column (2:98 *i*PrOH/hexane, 35 °C, 1 mL/min, detection at 210 nm): $\mathbf{t_{R1}}$ = 8.5 min, $\mathbf{t_{R2}}$ = 9.5 min (ACB Reaction with (*R*,*S*)-**Cu9** at 23 °C).

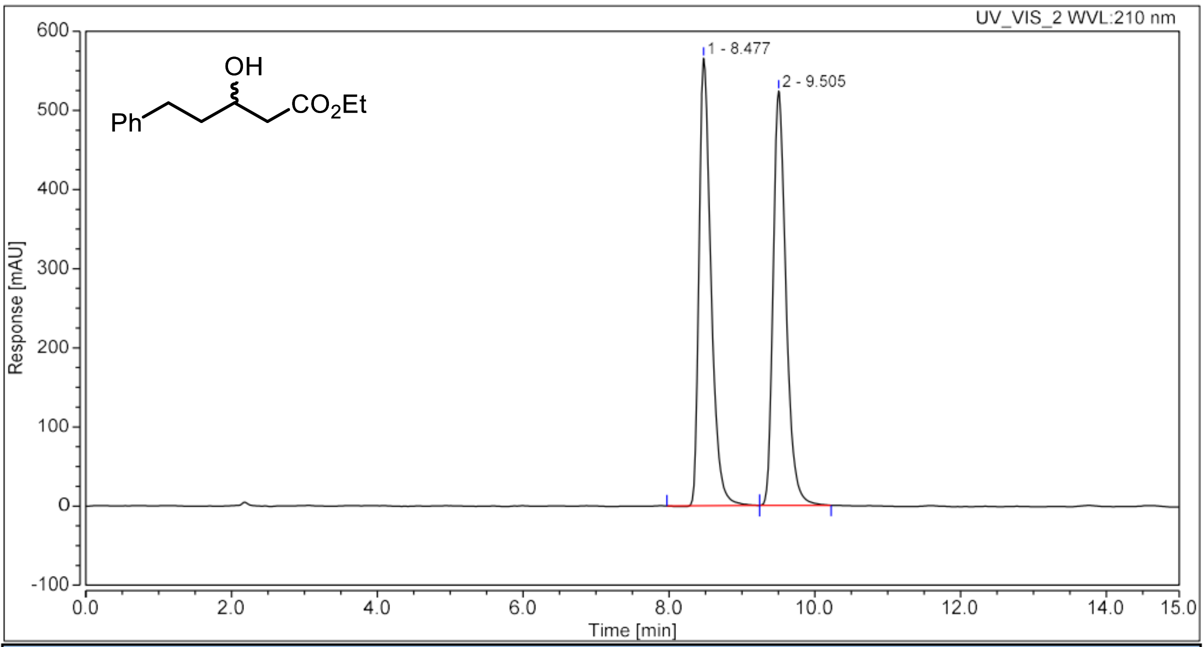

Integration Results

No.	Peak Name	Retention Time min	Area mAU*min	Height mAU	Relative Area %	Relative Height %	Amount n.a.
1		8.477	107.353	565.331	49.99	51.93	n.a.
2		9.505	107.381	523.378	50.01	48.07	n.a.
**Total:**			**214.733**	**1088.708**	**100.00**	**100.00**	

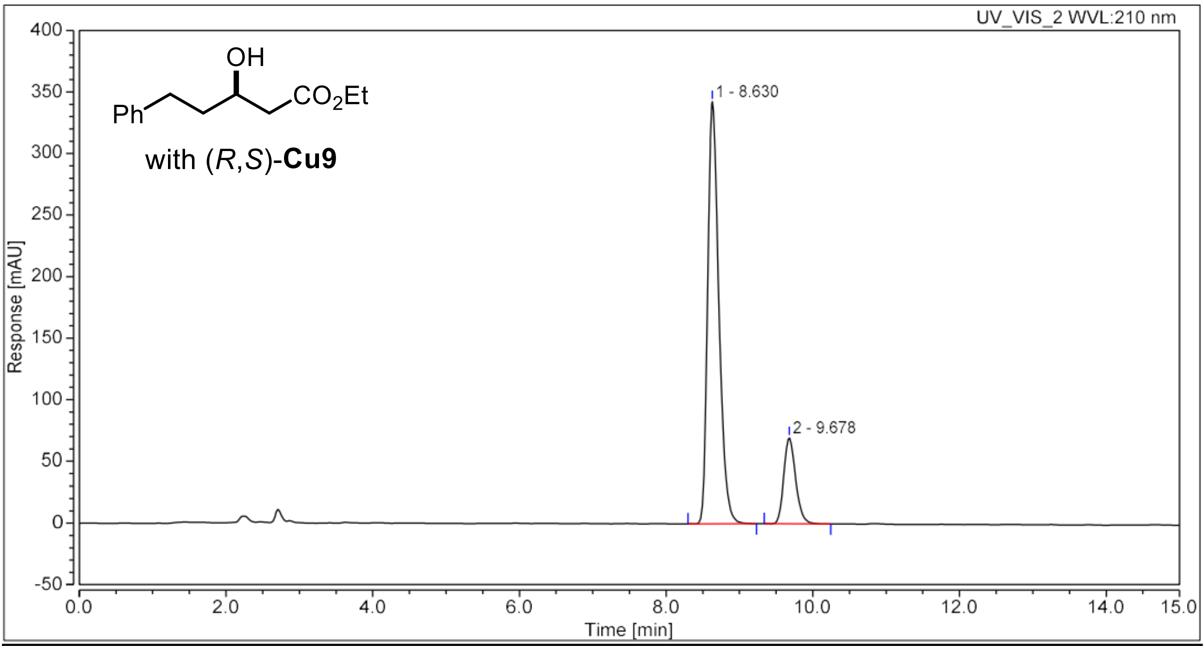

Integration Results

No.	Peak Name	Retention Time min	Area mAU*min	Height mAU	Relative Area %	Relative Height %	Amount n.a.
1		8.630	60.455	342.758	82.27	83.12	n.a.
2		9.678	13.031	69.592	17.73	16.88	n.a.
**Total:**			**73.486**	**412.350**	**100.00**	**100.00**	

Chiral HPLC Chiralpak IB-3 column (2:98 *i*PrOH/hexane, 35 °C, 1 mL/min, detection at 210 nm): $\mathbf{t_{R1}}$ = 8.5 min, $\mathbf{t_{R2}}$ = 9.5 min (ACB Reaction with (*R*,*R*)-**Cu9** at 23 °C).

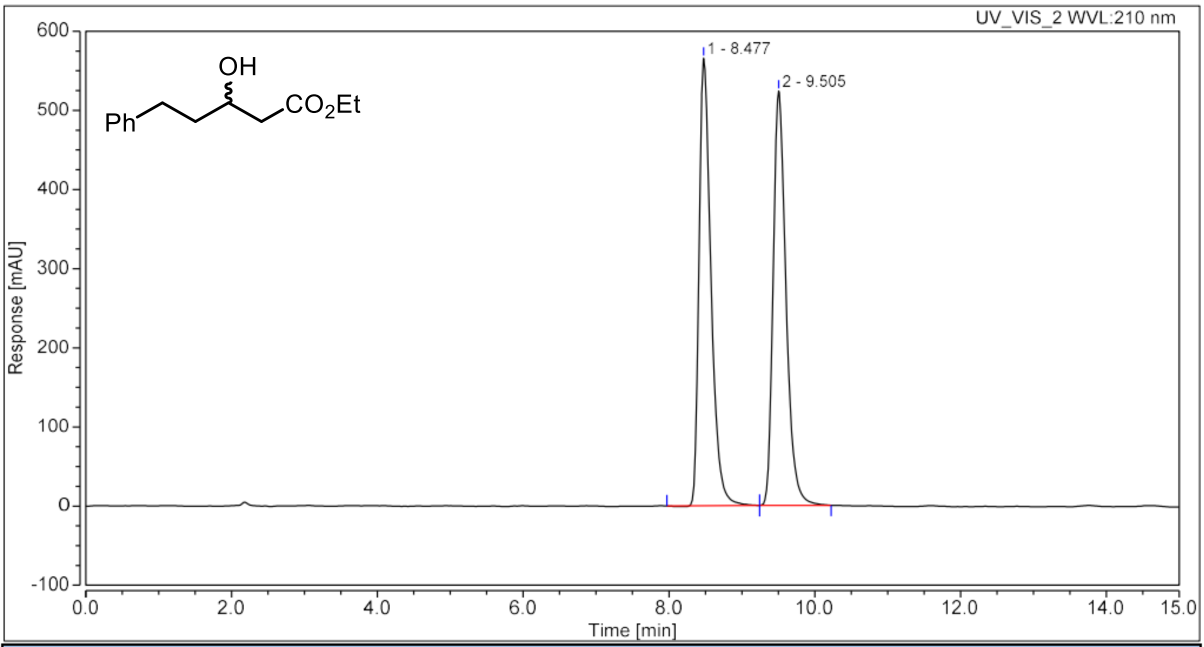

Integration Results							
No.	Peak Name	Retention Time min	Area mAU*min	Height mAU	Relative Area %	Relative Height %	Amount n.a.
1		8.477	107.353	565.331	49.99	51.93	n.a.
2		9.505	107.381	523.378	50.01	48.07	n.a.
**Total:**			**214.733**	**1088.708**	**100.00**	**100.00**	

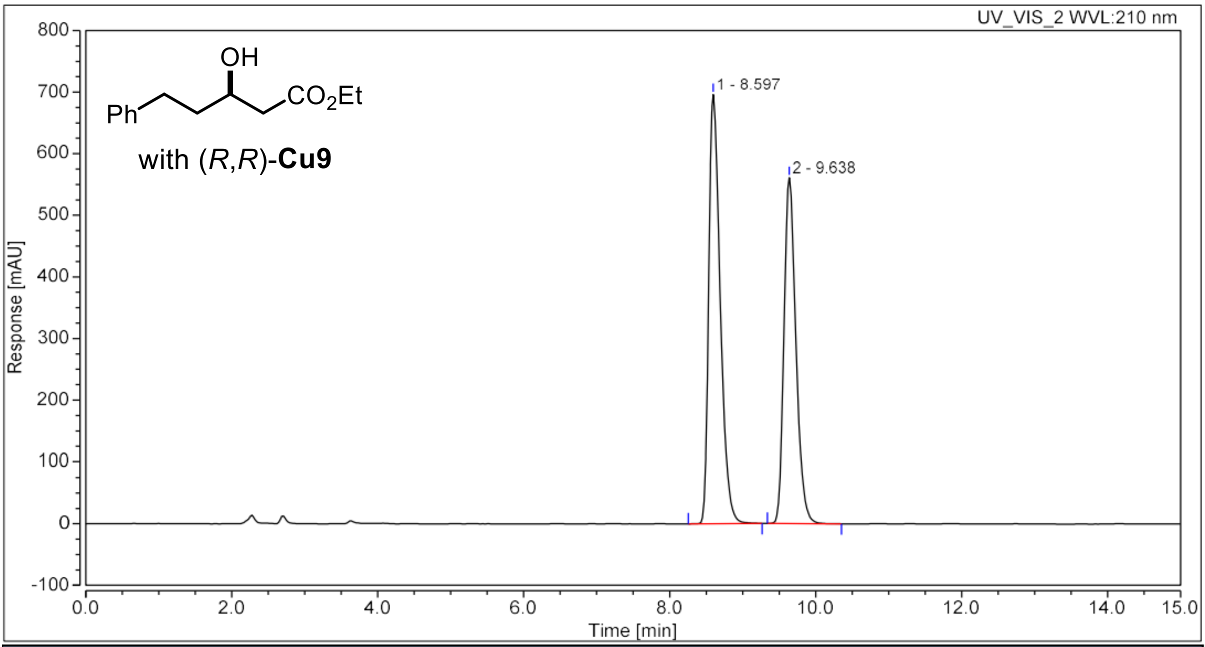

Integration Results							
No.	Peak Name	Retention Time min	Area mAU*min	Height mAU	Relative Area %	Relative Height %	Amount n.a.
1		8.597	126.741	696.004	54.38	55.37	n.a.
2		9.638	106.325	560.987	45.62	44.63	n.a.
**Total:**			**233.066**	**1256.991**	**100.00**	**100.00**	

Chiral HPLC Chiralpak IB-3 column (2:98 *i*PrOH/hexane, 35 °C, 1 mL/min, detection at 210 nm): $\mathbf{t_{R1}}$ = 8.5 min, $\mathbf{t_{R2}}$ = 9.5 min (ACB Reaction with (*R*,*S*)-**Cu10** at 23 °C).

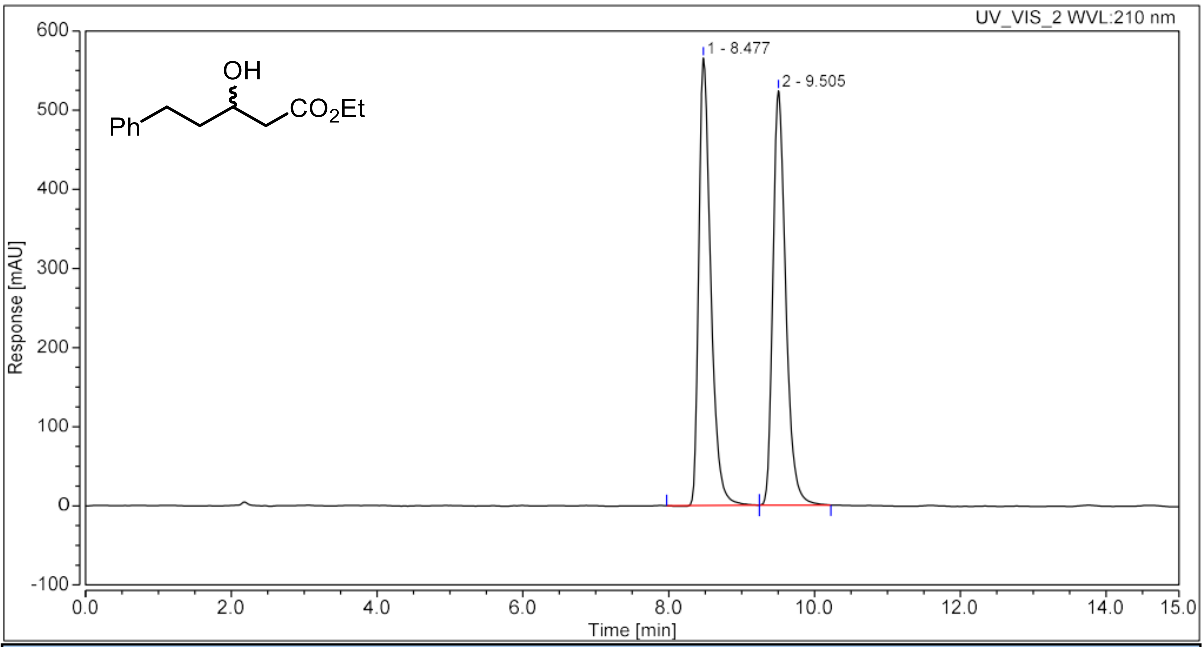

Integration Results

No.	Peak Name	Retention Time min	Area mAU*min	Height mAU	Relative Area %	Relative Height %	Amount n.a.
1		8.477	107.353	565.331	49.99	51.93	n.a.
2		9.505	107.381	523.378	50.01	48.07	n.a.
**Total:**			**214.733**	**1088.708**	**100.00**	**100.00**	

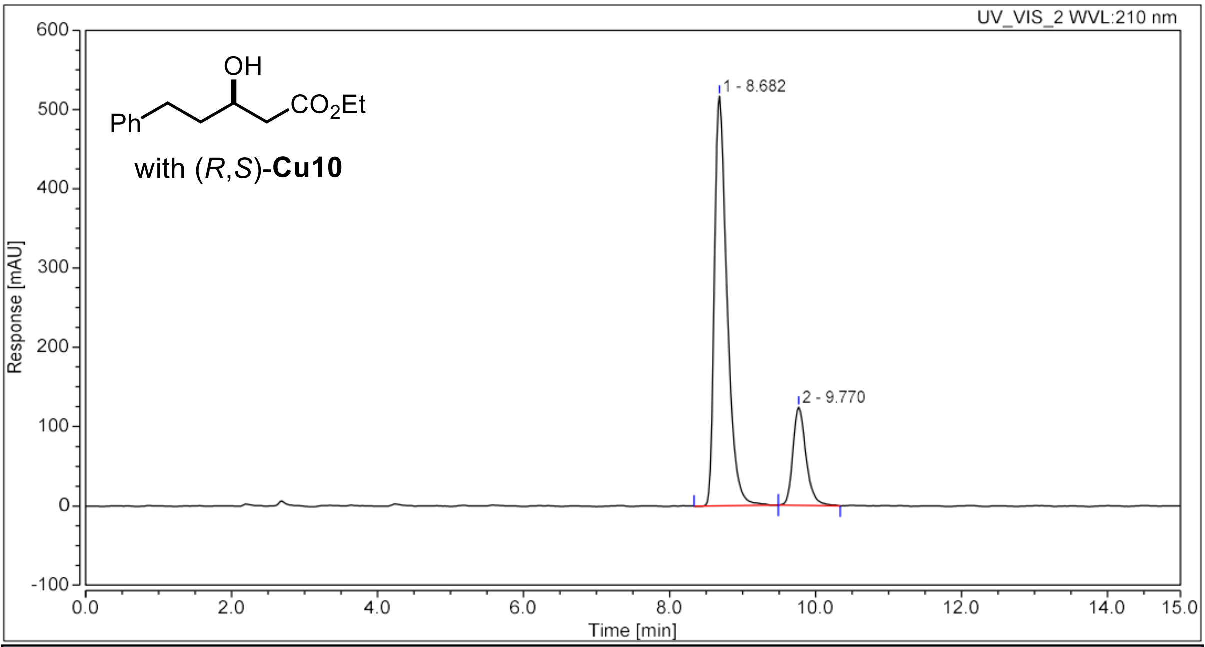

Integration Results

No.	Peak Name	Retention Time min	Area mAU*min	Height mAU	Relative Area %	Relative Height %	Amount n.a.
1		8.682	103.327	516.498	80.08	80.75	n.a.
2		9.770	25.698	123.148	19.92	19.25	n.a.
**Total:**			**129.025**	**639.646**	**100.00**	**100.00**	

Chiral HPLC Chiralpak IB-3 column (2:98 *i*PrOH/hexane, 35 °C, 1 mL/min, detection at 210 nm): $\mathbf{t_{R1}}$ = 8.5 min, $\mathbf{t_{R2}}$ = 9.5 min (ACB Reaction with (*R*,*R*)-**Cu10** at 23 °C).

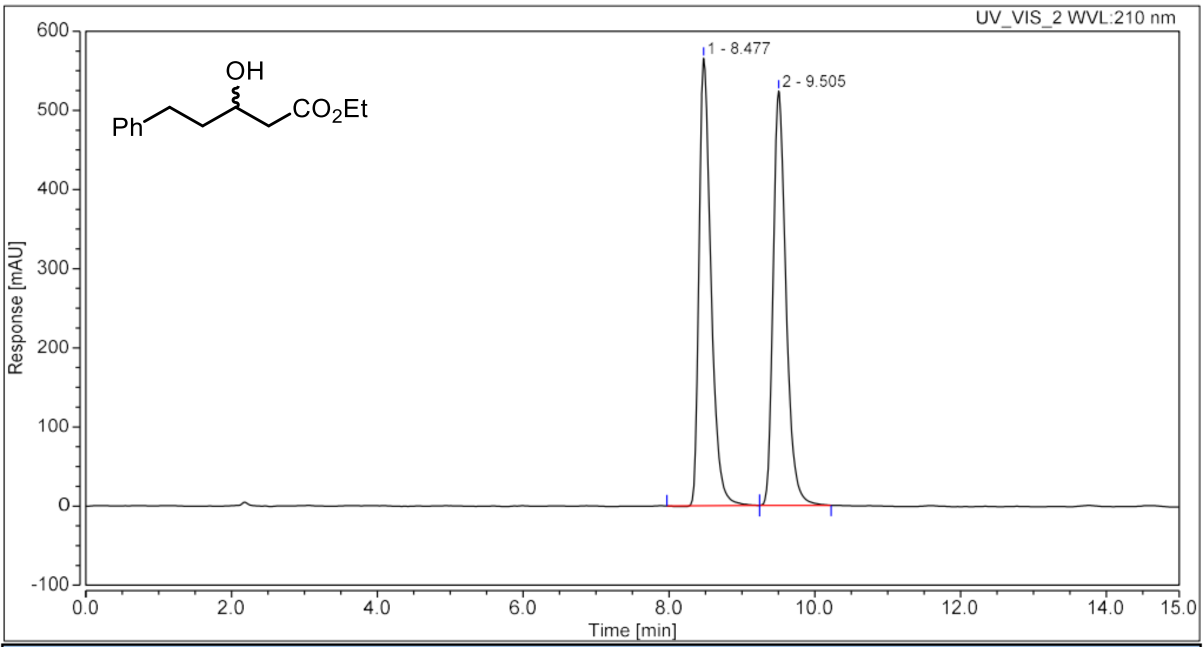

Integration Results

No.	Peak Name	Retention Time min	Area mAU*min	Height mAU	Relative Area %	Relative Height %	Amount n.a.
1		8.477	107.353	565.331	49.99	51.93	n.a.
2		9.505	107.381	523.378	50.01	48.07	n.a.
**Total:**			**214.733**	**1088.708**	**100.00**	**100.00**	

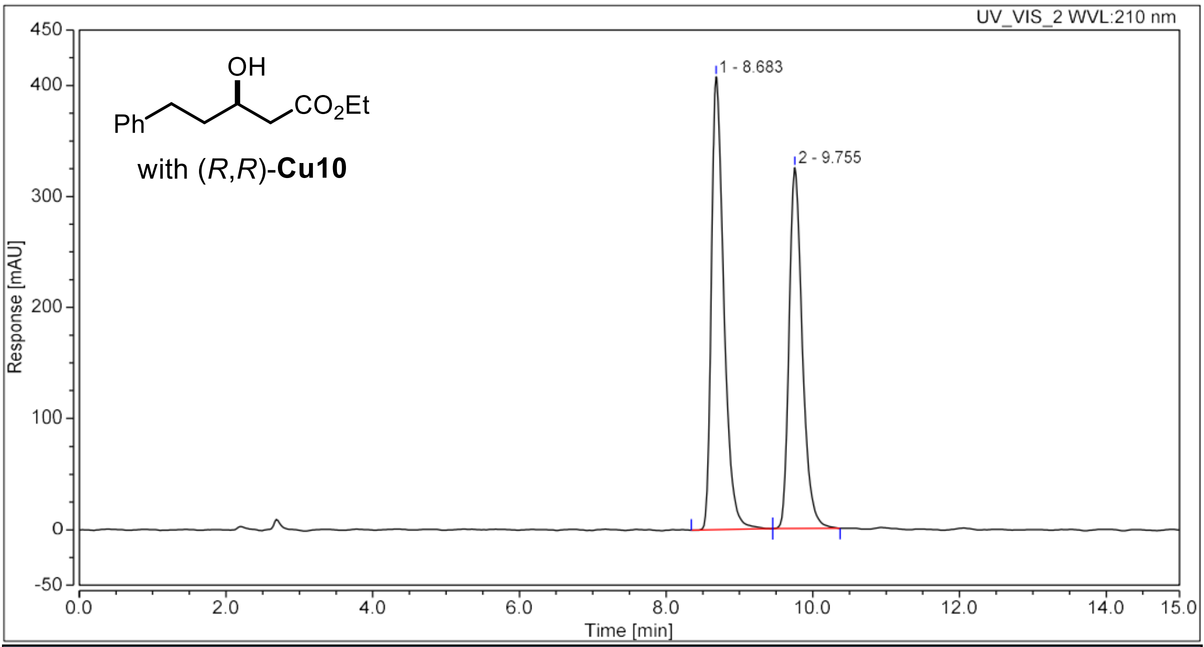

Integration Results

No.	Peak Name	Retention Time min	Area mAU*min	Height mAU	Relative Area %	Relative Height %	Amount n.a.
1		8.683	80.112	407.809	54.13	55.66	n.a.
2		9.755	67.893	324.826	45.87	44.34	n.a.
**Total:**			**148.005**	**732.635**	**100.00**	**100.00**	

Chiral HPLC Chiralpak IB-3 column (2:98 *i*PrOH/hexane, 35 °C, 1 mL/min, detection at 210 nm): $\mathbf{t_{R1}}$ = 8.5 min, $\mathbf{t_{R2}}$ = 9.5 min (ACB Reaction with (*R*,*S*)-**Cu11** at 23 °C).

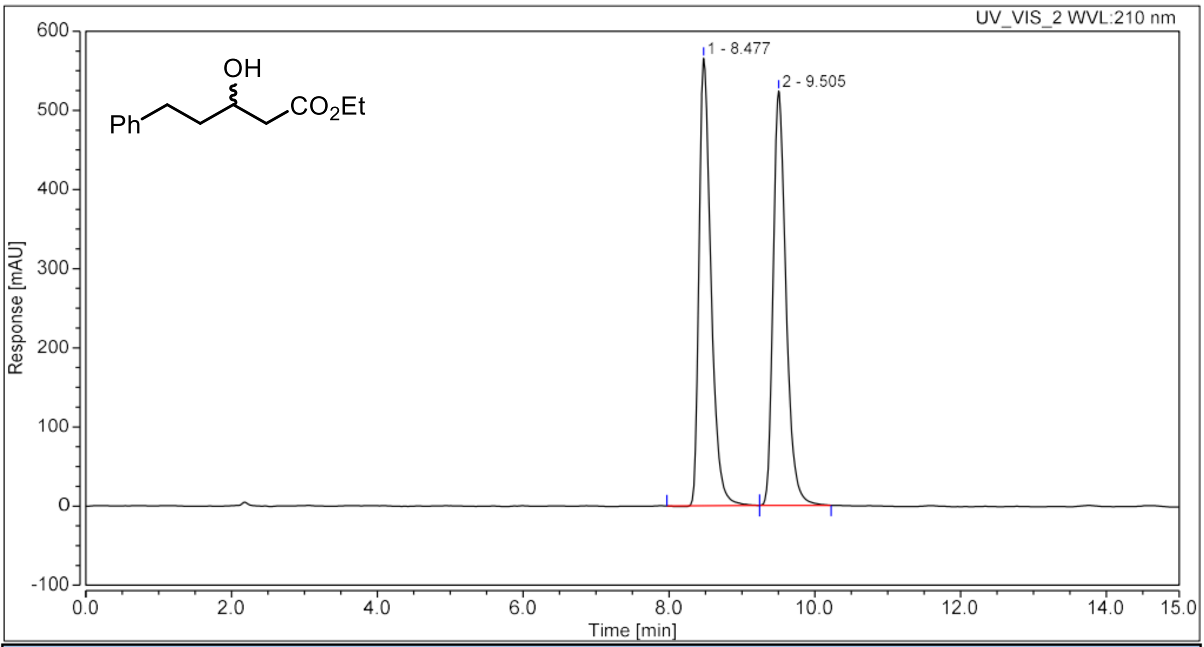

Integration Results

No.	Peak Name	Retention Time min	Area mAU*min	Height mAU	Relative Area %	Relative Height %	Amount n.a.
1		8.477	107.353	565.331	49.99	51.93	n.a.
2		9.505	107.381	523.378	50.01	48.07	n.a.
**Total:**			**214.733**	**1088.708**	**100.00**	**100.00**	

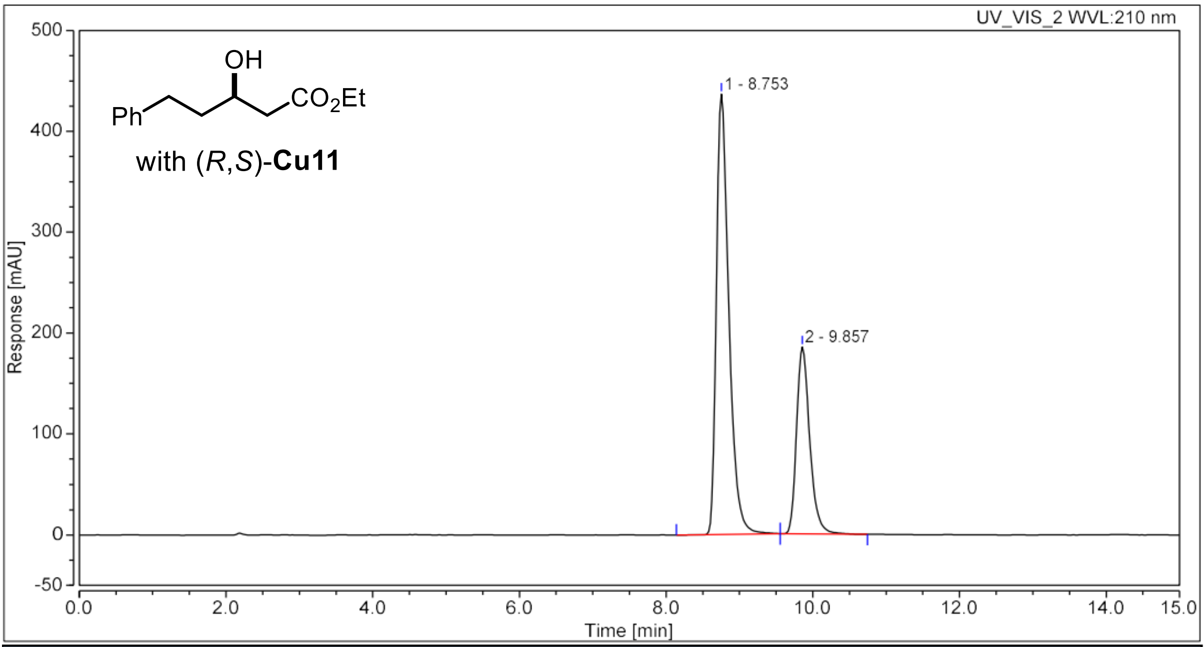

Integration Results

No.	Peak Name	Retention Time min	Area mAU*min	Height mAU	Relative Area %	Relative Height %	Amount n.a.
1		8.753	86.094	436.543	69.09	70.23	n.a.
2		9.857	38.523	185.031	30.91	29.77	n.a.
**Total:**			**124.617**	**621.574**	**100.00**	**100.00**	

Chiral HPLC Chiralpak IB-3 column (2:98 *i*PrOH/hexane, 35 °C, 1 mL/min, detection at 210 nm): $\mathbf{t_{R1}}$ = 8.5 min, $\mathbf{t_{R2}}$ = 9.5 min (ACB Reaction with (*R*,*R*)-**Cu11** at 23 °C).

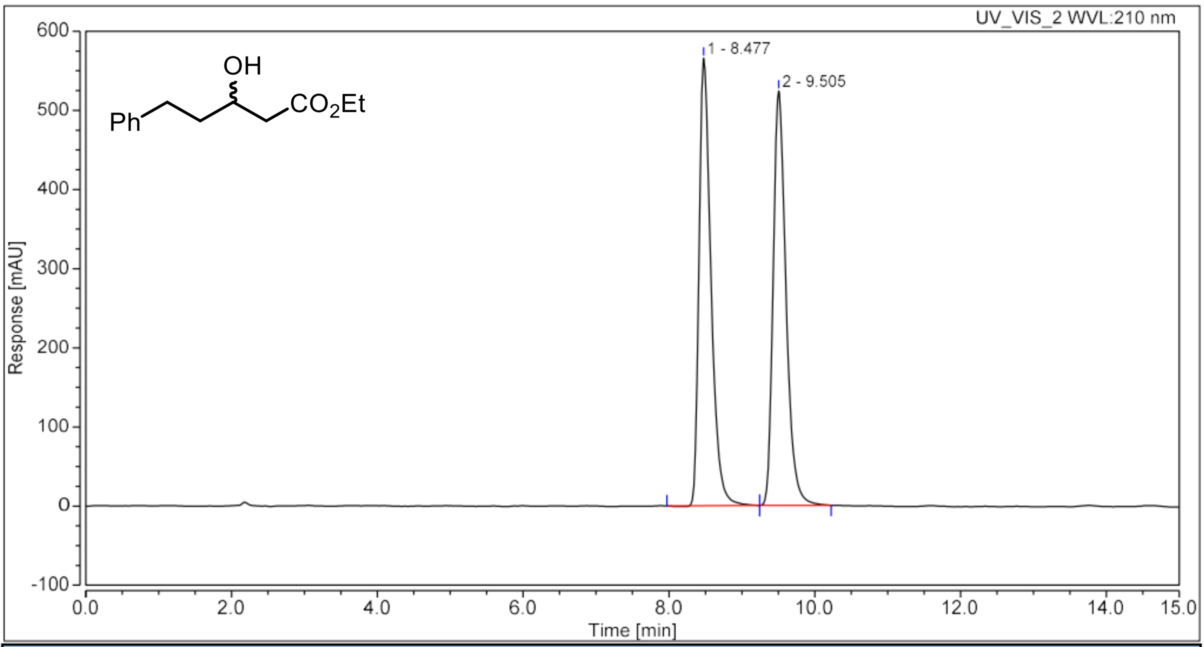

Integration Results

No.	Peak Name	Retention Time min	Area mAU*min	Height mAU	Relative Area %	Relative Height %	Amount n.a.
1		8.477	107.353	565.331	49.99	51.93	n.a.
2		9.505	107.381	523.378	50.01	48.07	n.a.
**Total:**			**214.733**	**1088.708**	**100.00**	**100.00**	

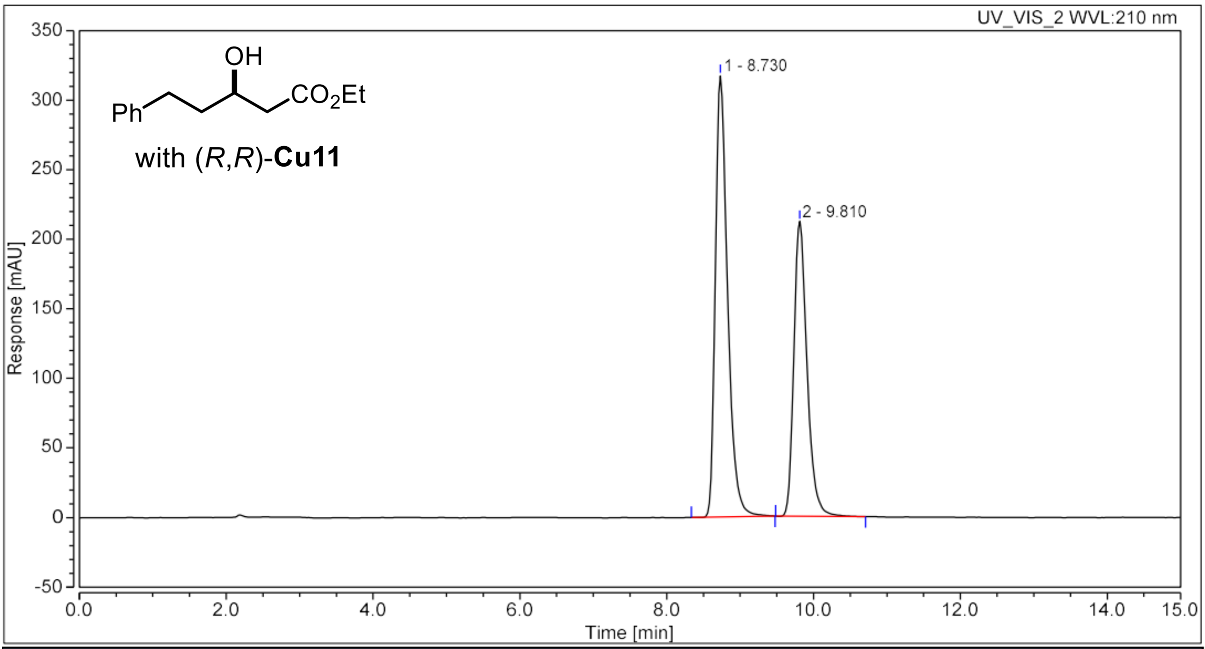

Integration Results

No.	Peak Name	Retention Time min	Area mAU*min	Height mAU	Relative Area %	Relative Height %	Amount n.a.
1		8.730	61.228	317.106	58.15	59.96	n.a.
2		9.810	44.064	211.745	41.85	40.04	n.a.
**Total:**			**105.292**	**528.851**	**100.00**	**100.00**	

Asymmetric Ring Closing Metathesis (ARCM)

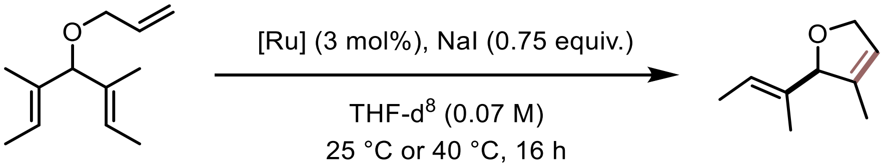

11 **12**

(2E,5E)-4-(allyloxy)-3,5-dimethylhepta-2,5-diene (11)

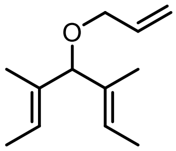

(2E,5E)-4-(allyloxy)-3,5-dimethylhepta-2,5-diene was prepared following literature procedures and the characterization data matched those previously reported.[14]

^{1}H NMR (400 MHz, $CDCl_3$) δ 5.92 (ddt, *J* = 17.2, 10.6, 5.4 Hz, 1H), δ 5.55 (qq, *J* = 6.8, 1.4 Hz, 2H), 5.26 (dq, *J* = 17.2, 1.8 Hz, 1H), 5.13 (dq, *J* = 10.4, 1.5 Hz, 1H), 3.94 (br s, 1H), 3.85 (dt, *J* = 5.4, 1.5 Hz, 2H), 1.64 (dq, *J* = 6.8, 1.1 Hz, 6H), 1.47 (t, *J* = 1.3 Hz, 6H).

(E)-2-(but-2-en-2-yl)-3-methyl-2,5-dihydrofuran (12)

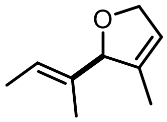

In a nitrogen-filled glovebox, the ruthenium complex (1.00 mg, 1.55 µmol, 3 mol%) was added as a stock solution (4.84 µmol/mL) in dry and degassed THF-d_8 (320 µL) to NaI (5.67 mg, 37.8 µmol, 0.75 equiv., 25 equiv. relative to ruthenium). The mixture was left to stir for 30 min in a microwave tube closed with a rubber septum. The triene (11.4 mg, 50.3 µmol, 1.0 equiv.) was transferred to the mixture with two portions of dry and degassed THF-d_8 (2 × 200 µL, total 720 µL, 0.07 M). The microwave tube was sealed and the mixture was stirred at the indicated temperature (23 °C or 40 °C) for 16 h.

Conversion and yield were assessed by NMR in THF-d_8 with 1,3,5-trimethoxybenzene as internal standard.

Enantiomeric excess was determined by crude injection on chiral GC after filtration of the THF solution over a small pad of neutral alumina.

^{1}H NMR (400 MHz, THF-d_8) δ 5.44 (qd, *J* = 6.8, 1.7 Hz, 1H), 4.81–4.78 (m, 1H), 4.58–4.51 (m, 1H), 4.49–4.43 (m, 1H), 1.61 (dq, *J* = 6.6, 1.1 Hz, 3H), 1.54 (dq, *J* = 2.1, 1.1 Hz, 3H), 1.44 (q, *J* = 1.2 Hz, 3H).

Authentic racemic material was prepared following the same procedure with Hoveyda-Grubbs 1st generation catalyst (10 mol%). The characterization data matched those previously reported in the literature.[14]

Enantiomeric excess determination. Enantiomeric purity was determined by chiral GC analysis in comparison with authentic racemic material (Astec® CHIRALDEX™ G-TA column: 30 m × 0.25 mm × 0.12 µm), Hydrogen as carrier gas (40 cm/sec), 100:1 split ratio, temperature program (Temperature - Hold Time): 60 °C - 60 min;

Authentic racemate: $\mathbf{t_{R1}}$= 35.9 min, $\mathbf{t_{R2}}$= 39.5 min.

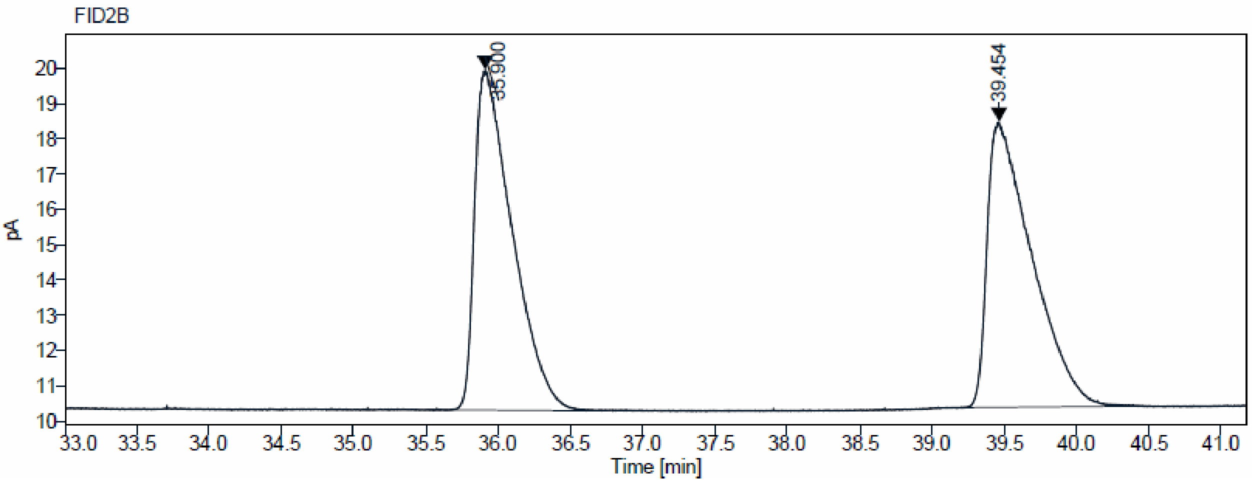

Signal: FID2B

RT [min]	Type	Width [min]	Area	Height	Area%	Name
35.900	MM m	1.1495	174.0429	9.6245	50.0224	
39.454	MM m	1.4398	173.8866	8.0747	49.9776	
		**Sum**	**347.9295**			

With (*R*)-**Ru0** (23 °C):

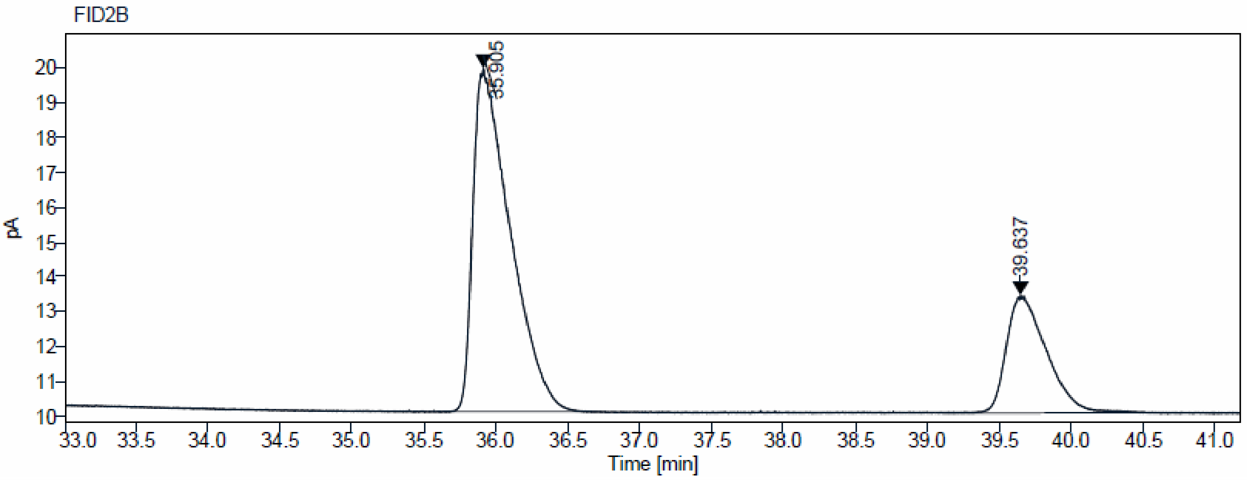

Signal: FID2B

RT [min]	Type	Width [min]	Area	Height	Area%	Name
35.905	MM m	1.1263	175.9324	9.7662	73.0679	
39.637	MM m	1.4630	64.8470	3.3390	26.9321	
		**Sum**	**240.7794**			

Authentic racemate: $\mathbf{t_{R1}}$= 38.0 min, $\mathbf{t_{R2}}$= 41.7 min.

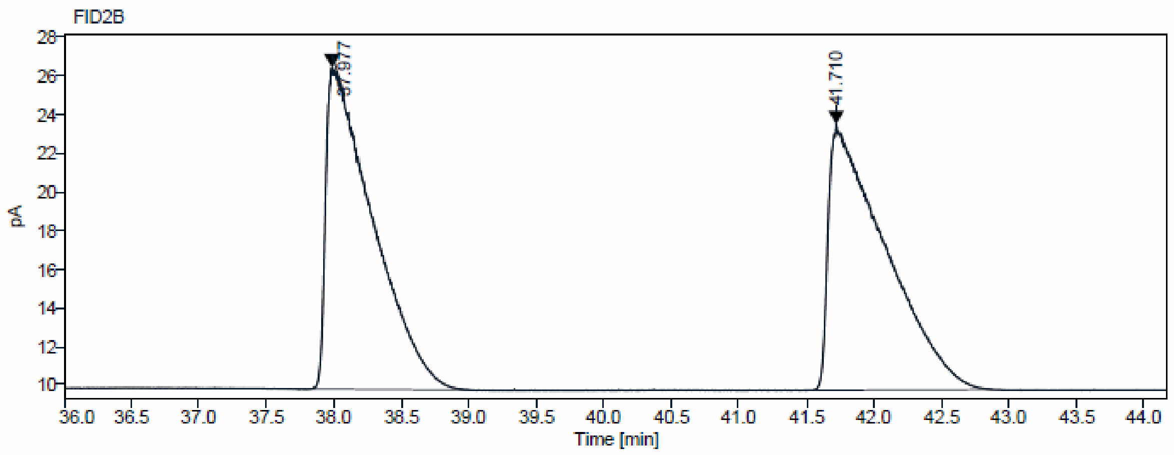

Signal: FID2B

RT [min]	Type	Width [min]	Area	Height	Area%	Name
37.977	MM m	1.3036	400.3474	16.6272	49.9107	
41.710	MM m	1.4649	401.7794	13.7527	50.0893	
		**Sum**	**802.1267**			

With (*R*,*S*)-**Ru1** (23 °C):

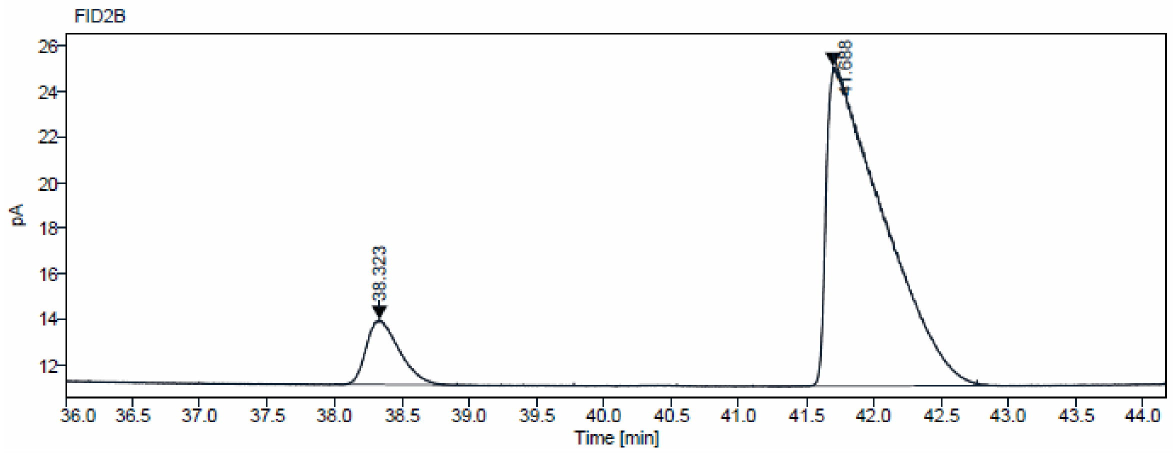

Signal: FID2B

RT [min]	Type	Width [min]	Area	Height	Area%	Name
38.323	MM m	1.0974	48.1615	2.8616	10.5477	
41.688	MM m	1.9969	408.4441	14.0613	89.4523	
		**Sum**	**456.6056**			

Authentic racemate: $\mathbf{t_{R1}}$= 38.0 min, $\mathbf{t_{R2}}$= 41.7 min.

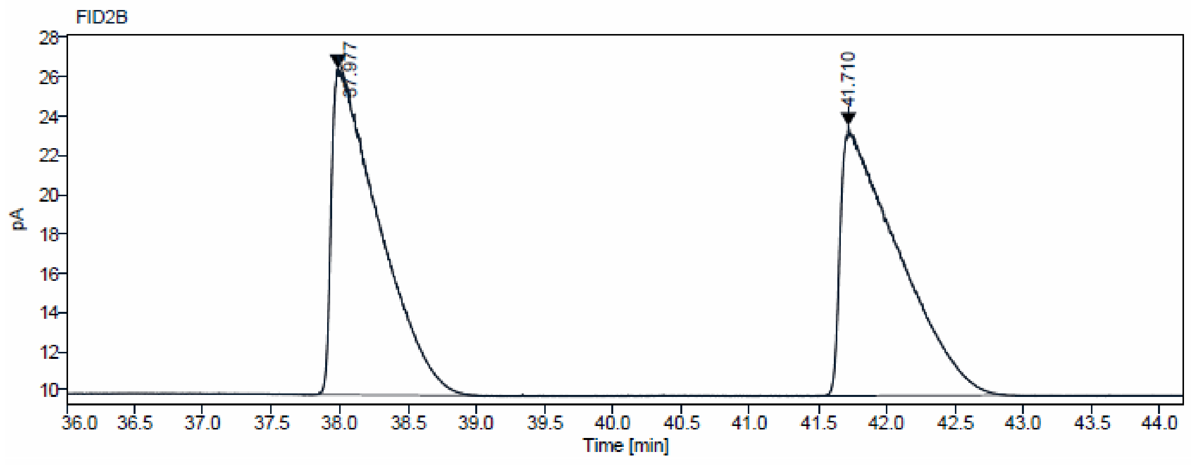

Signal: FID2B

RT [min]	Type	Width [min]	Area	Height	Area%	Name
37.977	MM m	1.3036	400.3474	16.6272	49.9107	
41.710	MM m	1.4649	401.7794	13.7527	50.0893	
		**Sum**	**802.1267**			

With (*R*,*S*)-**Ru1** (40 °C):

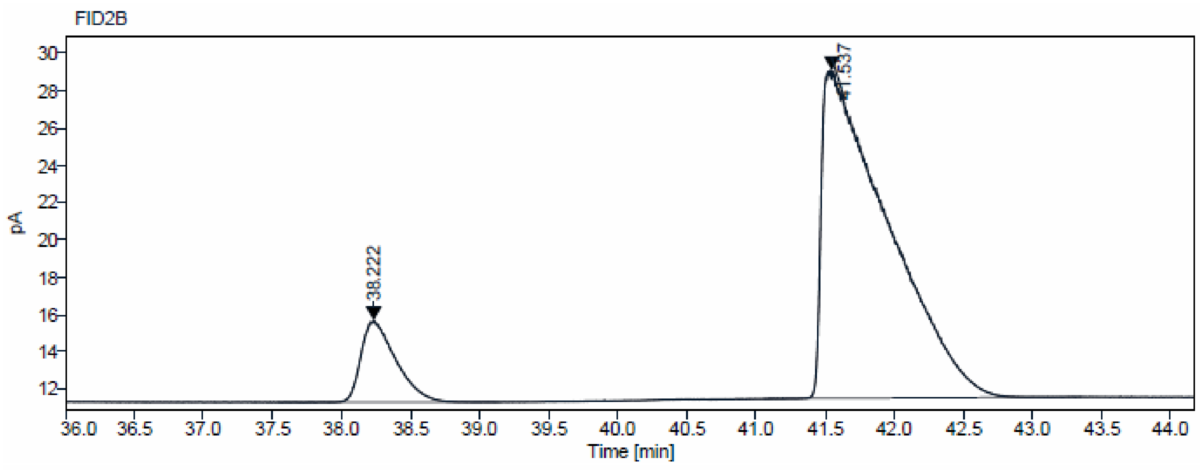

Signal: FID2B

RT [min]	Type	Width [min]	Area	Height	Area%	Name
38.222	MM m	1.6005	75.8736	4.3562	11.5024	
41.537	MM m	2.3042	583.7598	17.5606	88.4976	
		**Sum**	**659.6334**			

Authentic racemate: $\mathbf{t_{R1}}$= 38.0 min, $\mathbf{t_{R2}}$= 41.7 min.

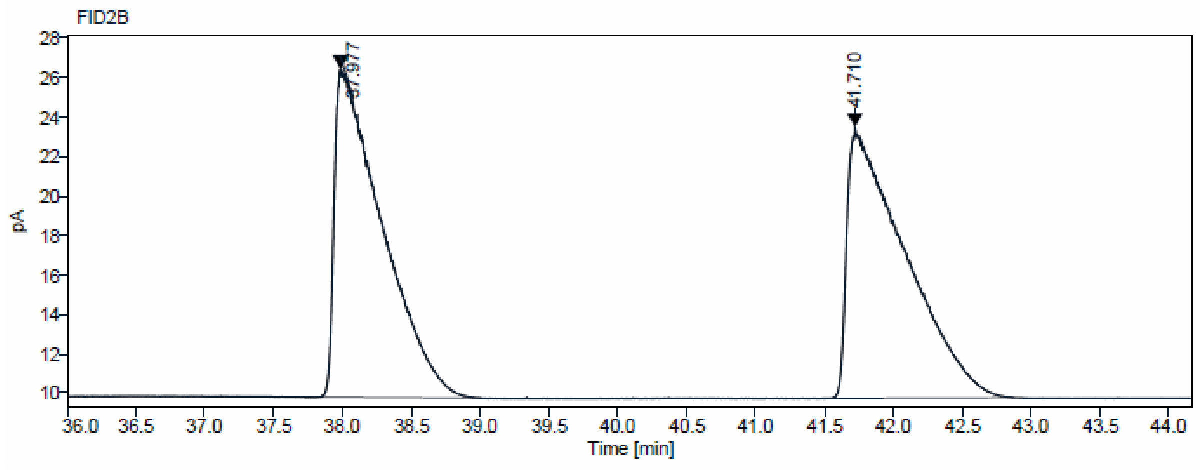

Signal: FID2B

RT [min]	Type	Width [min]	Area	Height	Area%	Name
37.977	MM m	1.3036	400.3474	16.6272	49.9107	
41.710	MM m	1.4649	401.7794	13.7527	50.0893	
		**Sum**	**802.1267**			

With (*R*,*R*)-**Ru1**:

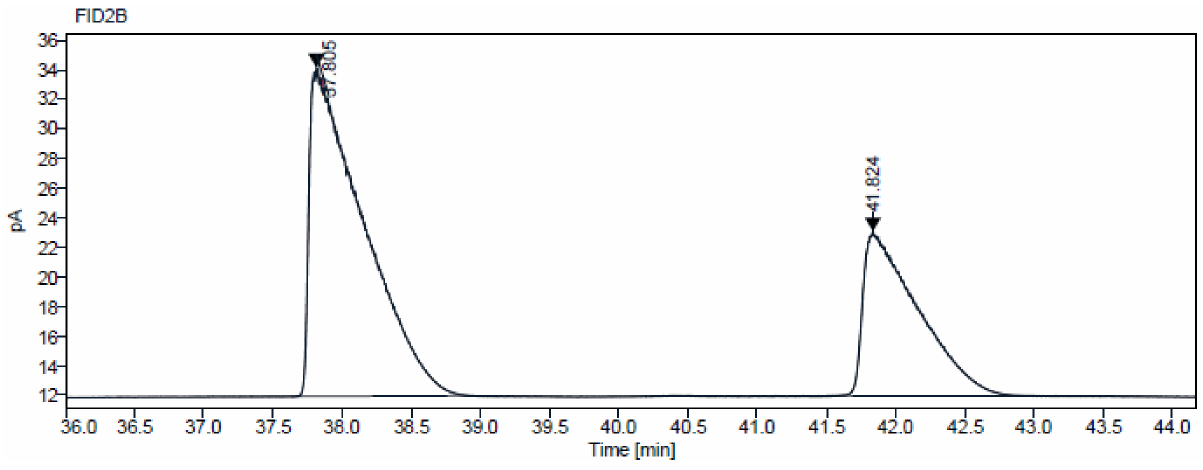

Signal: FID2B

RT [min]	Type	Width [min]	Area	Height	Area%	Name
37.805	MM m	1.2930	597.4756	22.1367	66.2244	
41.824	MM m	1.8685	304.7235	11.0935	33.7756	
		**Sum**	**902.1991**			

Authentic racemate: $\mathbf{t_{R1}}$= 38.0 min, $\mathbf{t_{R2}}$= 41.7 min.

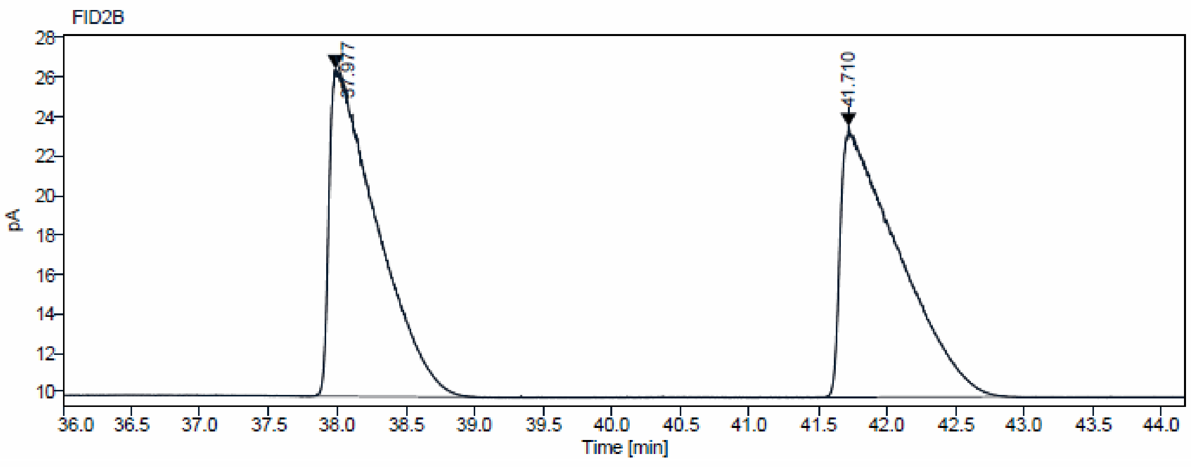

Signal: FID2B

RT [min]	Type	Width [min]	Area	Height	Area%	Name
37.977	MM m	1.3036	400.3474	16.6272	49.9107	
41.710	MM m	1.4649	401.7794	13.7527	50.0893	
		**Sum**	**802.1267**			

With (*R*,*S*)-**Ru2** (23 °C):

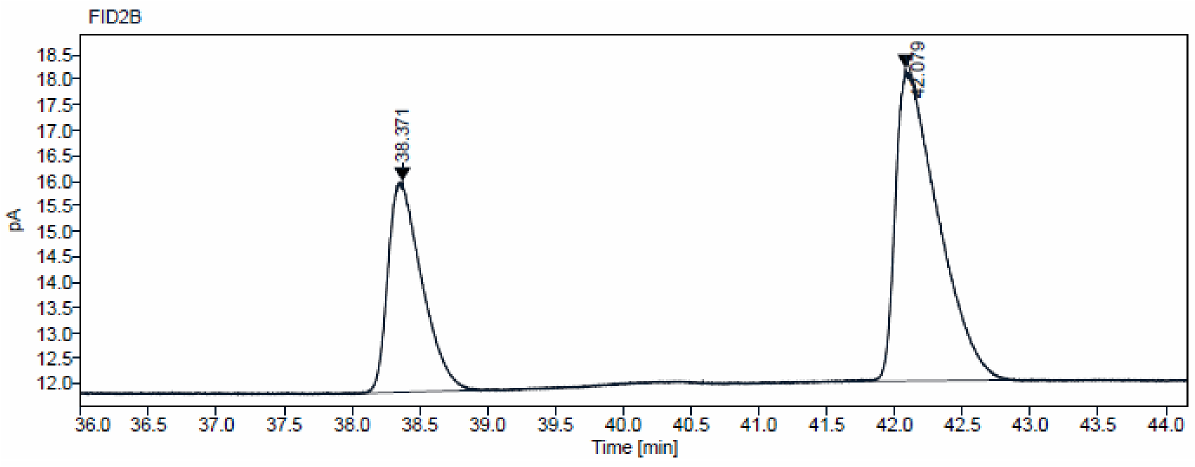

Signal: FID2B

RT [min]	Type	Width [min]	Area	Height	Area%	Name
38.371	MM m	1.1005	72.0772	4.1550	35.0675	
42.079	MM m	1.5461	133.4614	6.1751	64.9325	
		**Sum**	**205.5386**			

Authentic racemate: $\mathbf{t_{R1}}$= 38.0 min, $\mathbf{t_{R2}}$= 41.7 min.

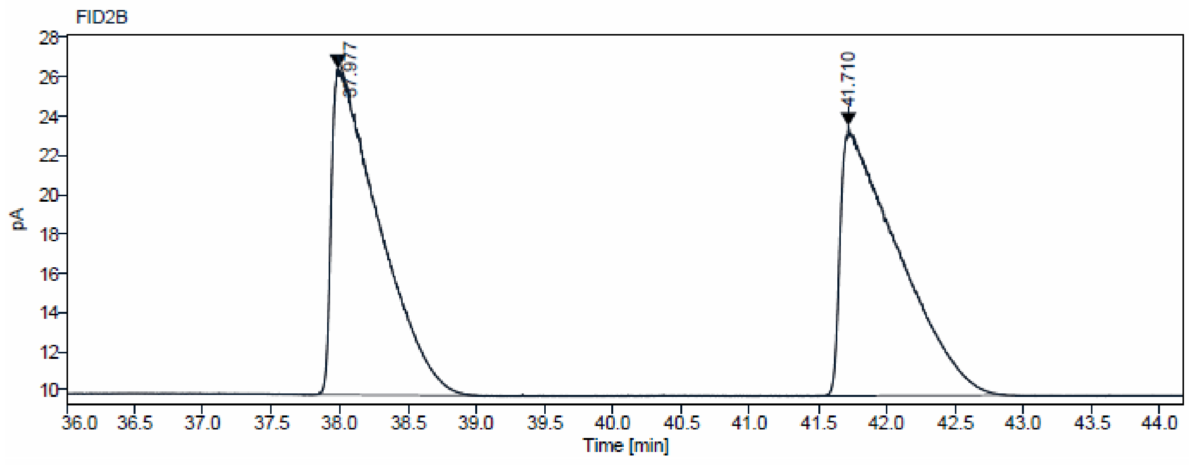

Signal: FID2B

RT [min]	Type	Width [min]	Area	Height	Area%	Name
37.977	MM m	1.3036	400.3474	16.6272	49.9107	
41.710	MM m	1.4649	401.7794	13.7527	50.0893	
		**Sum**	**802.1267**			

With (*R*,*S*)-**Ru2** (40 °C):

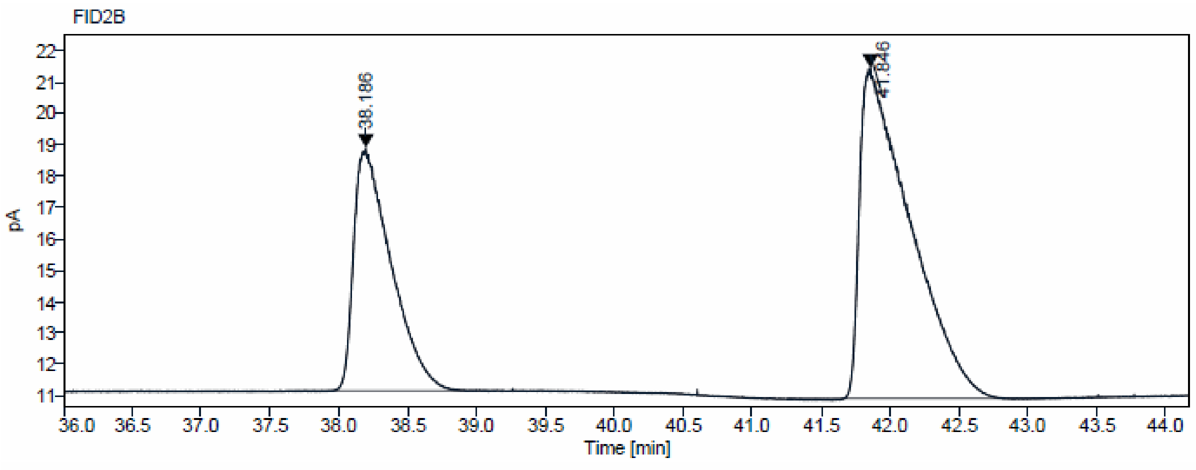

Signal: FID2B

RT [min]	Type	Width [min]	Area	Height	Area%	Name
38.186	MM m	1.4717	145.2973	7.7251	35.2910	
41.846	MM m	1.9302	266.4149	10.5185	64.7090	
		**Sum**	**411.7121**			

Authentic racemate: $\mathbf{t_{R1}}$= 38.0 min, $\mathbf{t_{R2}}$= 41.7 min.

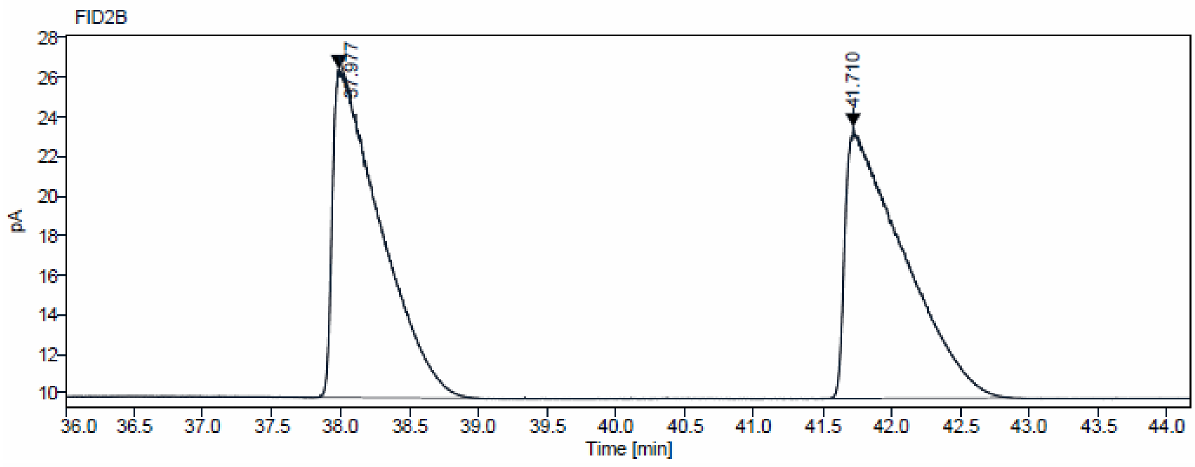

Signal: FID2B

RT [min]	Type	Width [min]	Area	Height	Area%	Name
37.977	MM m	1.3036	400.3474	16.6272	49.9107	
41.710	MM m	1.4649	401.7794	13.7527	50.0893	
		**Sum**	**802.1267**			

With (*R*,*R*)-**Ru2**:

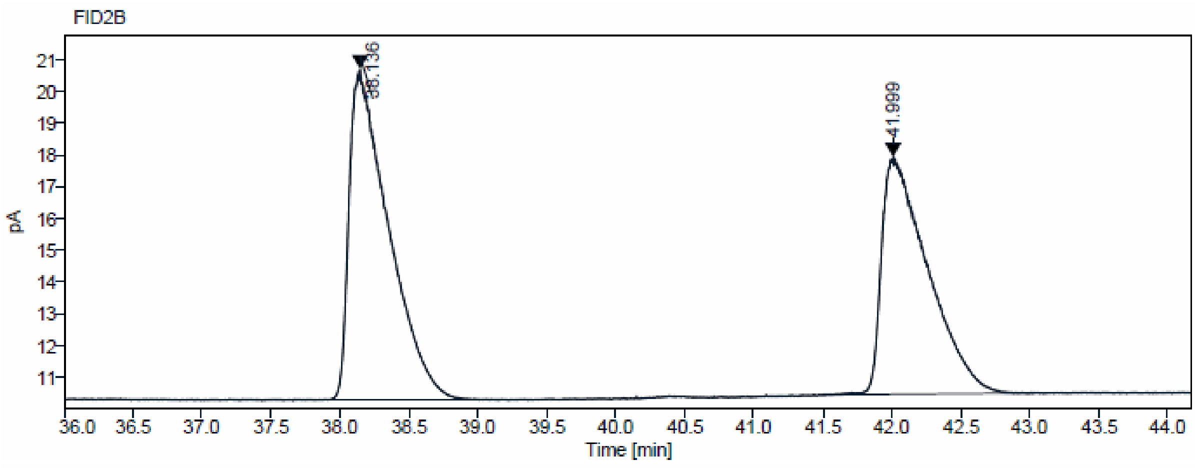

Signal: FID2B

RT [min]	Type	Width [min]	Area	Height	Area%	Name
38.136	MM m	1.7477	205.3255	10.4020	54.2341	
41.999	MM m	1.5077	173.2654	7.4653	45.7659	
		**Sum**	**378.5909**			

Authentic racemate: $\mathbf{t_{R1}}$= 38.0 min, $\mathbf{t_{R2}}$= 41.7 min.

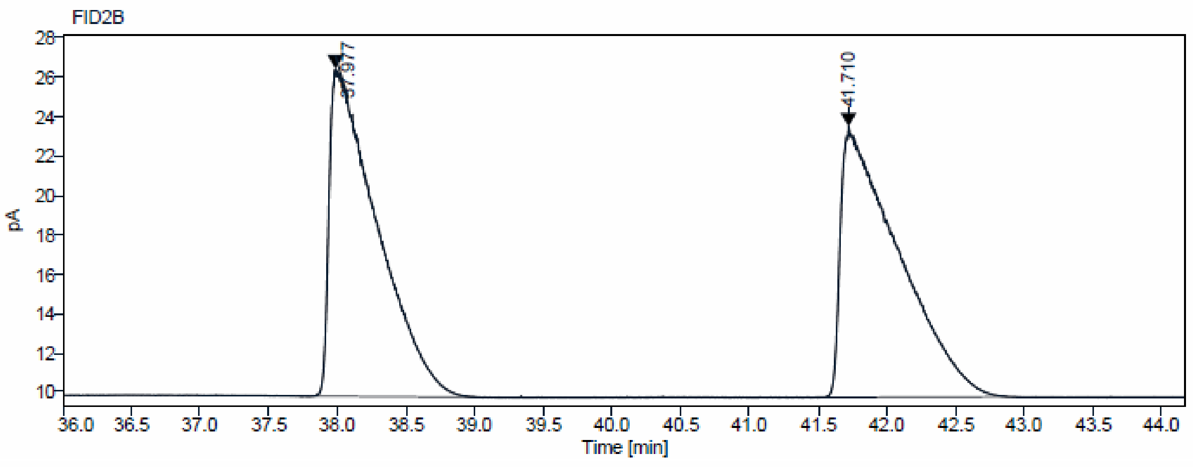

Signal: FID2B

RT [min]	Type	Width [min]	Area	Height	Area%	Name
37.977	MM m	1.3036	400.3474	16.6272	49.9107	
41.710	MM m	1.4649	401.7794	13.7527	50.0893	
		**Sum**	**802.1267**			

With (*R*,*S*)-**Ru3** (23 °C):

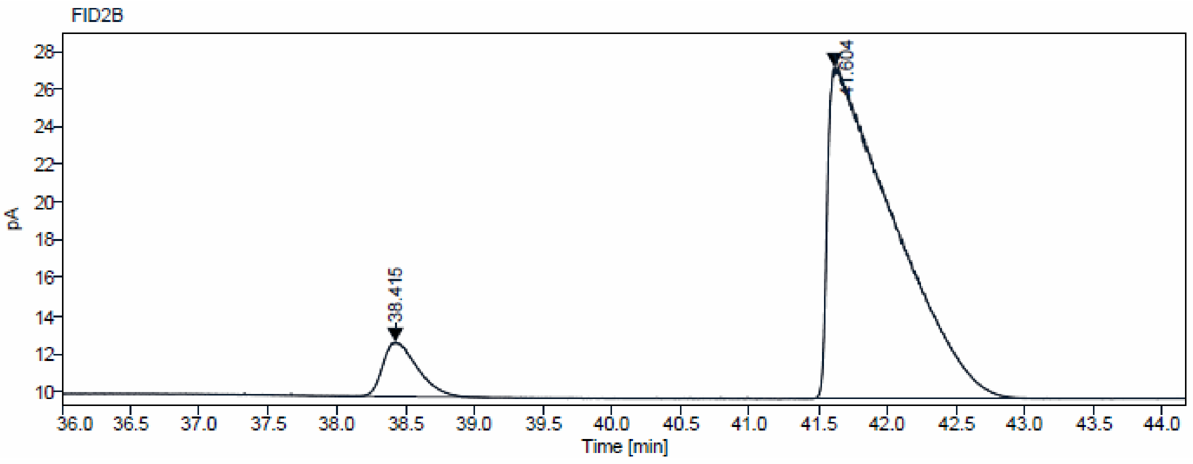

Signal: FID2B

RT [min]	Type	Width [min]	Area	Height	Area%	Name
38.415	MM m	1.1009	50.3388	2.8954	7.9431	
41.604	MM m	2.1539	583.4043	17.6011	92.0569	
		**Sum**	**633.7431**			

Authentic racemate: $\mathbf{t_{R1}}$= 38.0 min, $\mathbf{t_{R2}}$= 41.7 min.

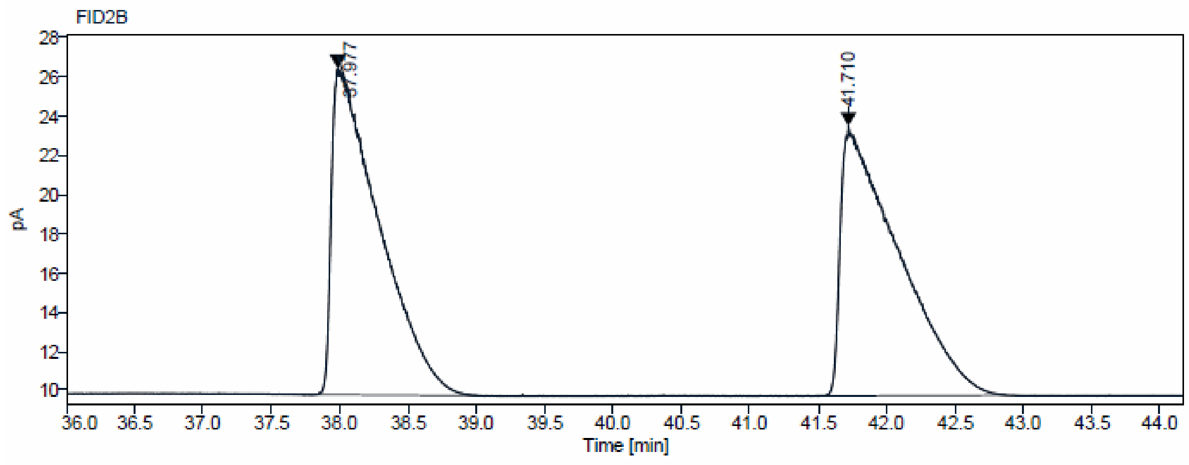

Signal: FID2B

RT [min]	Type	Width [min]	Area	Height	Area%	Name
37.977	MM m	1.3036	400.3474	16.6272	49.9107	
41.710	MM m	1.4649	401.7794	13.7527	50.0893	
		**Sum**	**802.1267**			

With (*R*,*S*)-**Ru3** (40 °C):

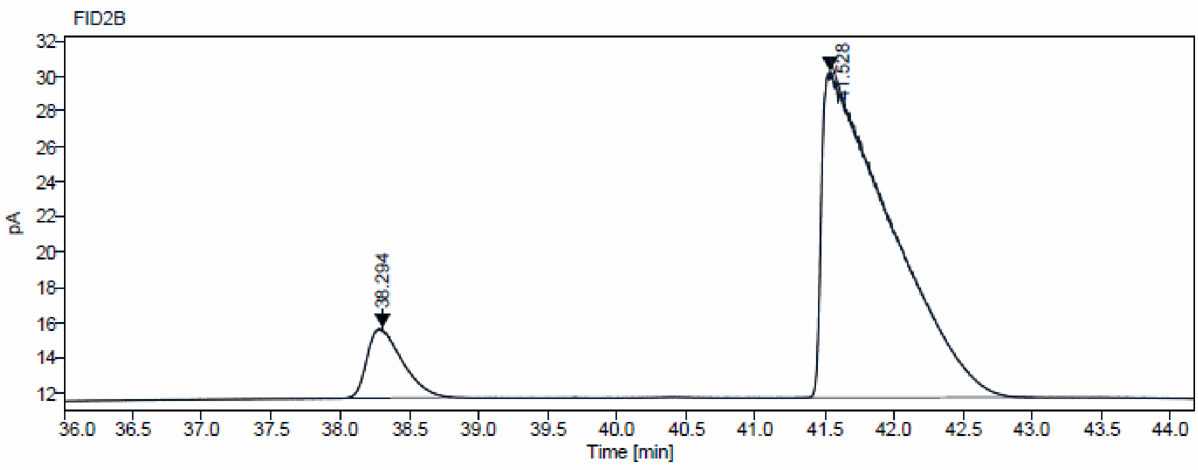

Signal: FID2B

RT [min]	Type	Width [min]	Area	Height	Area%	Name
38.294	MM m	0.7825	69.4864	3.9703	9.8787	
41.528	MM m	1.7280	633.9085	18.5680	90.1213	
		**Sum**	**703.3949**			

Authentic racemate: $\mathbf{t_{R1}}$= 38.0 min, $\mathbf{t_{R2}}$= 41.7 min.

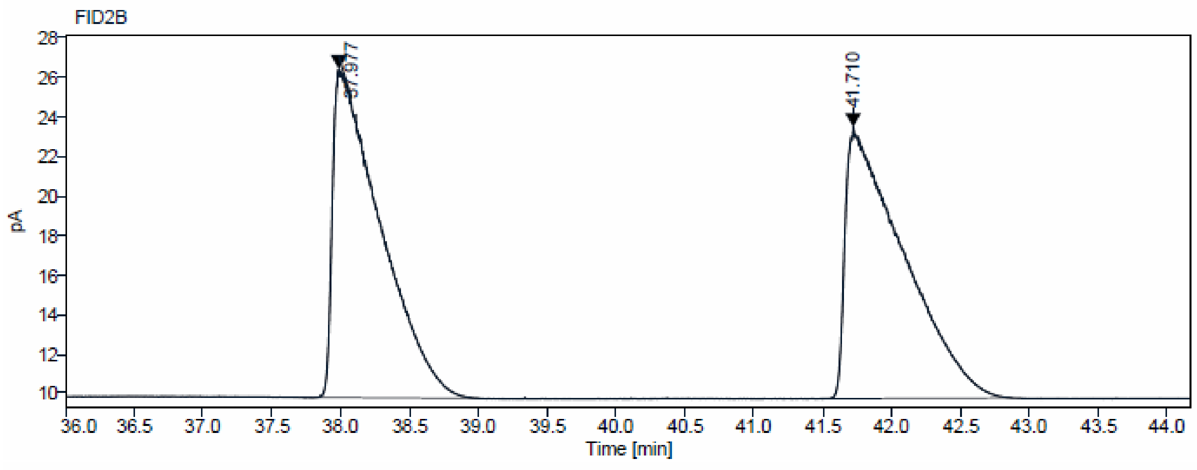

Signal: FID2B

RT [min]	Type	Width [min]	Area	Height	Area%	Name
37.977	MM m	1.3036	400.3474	16.6272	49.9107	
41.710	MM m	1.4649	401.7794	13.7527	50.0893	
		**Sum**	**802.1267**			

With (*R*,*R*)-**Ru3**:

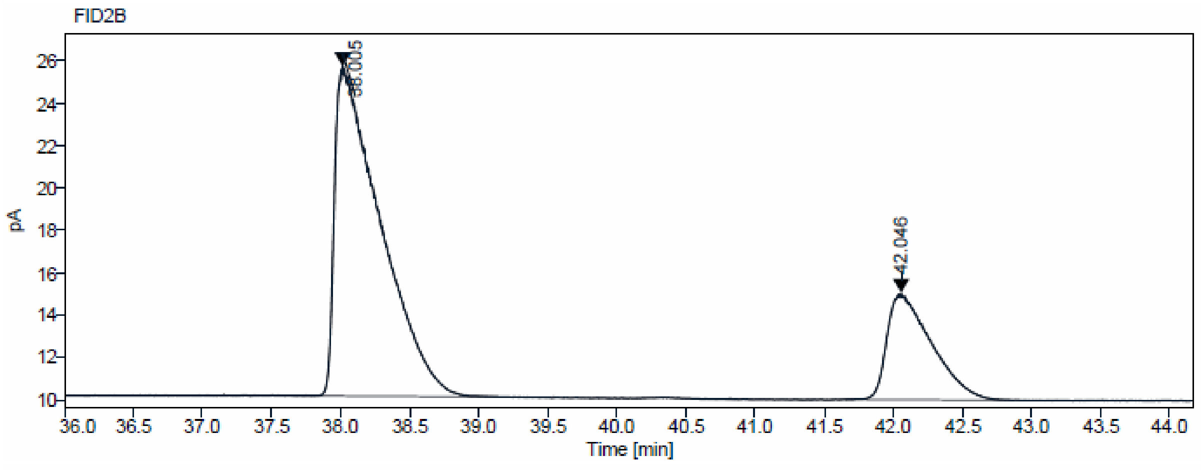

Signal: FID2B

RT [min]	Type	Width [min]	Area	Height	Area%	Name
38.005	MM m	1.5680	358.1177	15.5585	76.1573	
42.046	MM m	1.2052	112.1167	5.0365	23.8427	
		**Sum**	**470.2344**			

Authentic racemate: $\mathbf{t_{R1}}$= 38.0 min, $\mathbf{t_{R2}}$= 41.7 min.

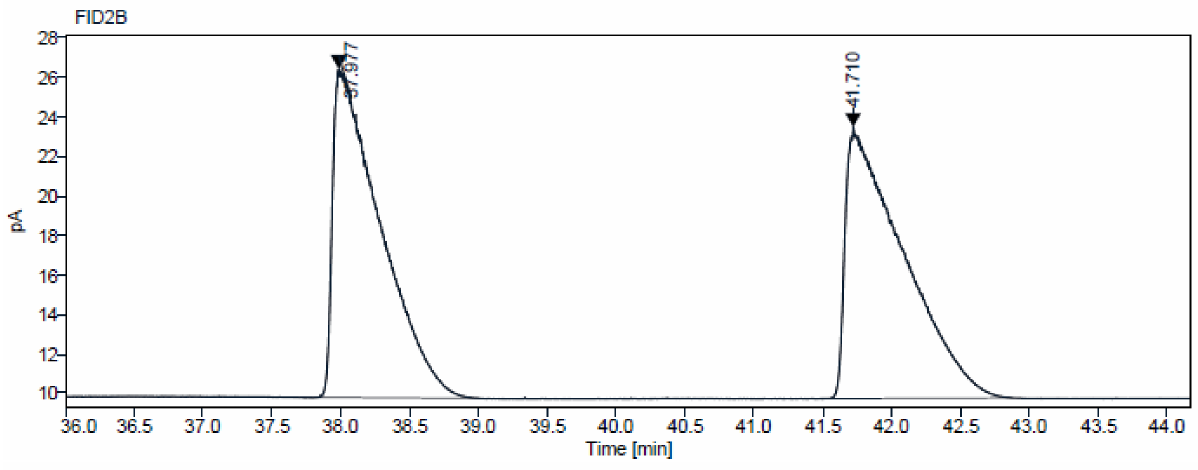

Signal: FID2B

RT [min]	Type	Width [min]	Area	Height	Area%	Name
37.977	MM m	1.3036	400.3474	16.6272	49.9107	
41.710	MM m	1.4649	401.7794	13.7527	50.0893	
		**Sum**	**802.1267**			

With (*R*,*R*)-**Ru4**:

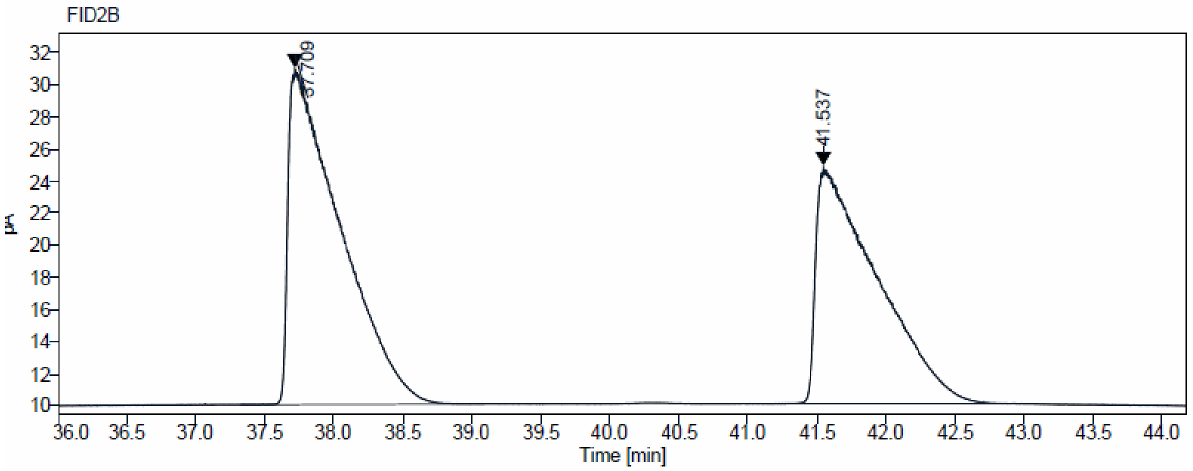

Signal: FID2B

RT [min]	Type	Width [min]	Area	Height	Area%	Name
37.709	MM m	1.7611	546.7404	20.9654	54.7514	
41.537	MM m	1.9494	451.8474	14.8031	45.2486	
		**Sum**	**998.5879**			

Crystallographic data. Crystallographic data for the structures reported in this paper have been deposited at the Cambridge Crystallographic Data Center (CCDC) as Supplementary Publication No. 2193067 ((*R*,*S*)-**Cu1**), 2193068 ((*R*,*R*)-**Cu1**), 2366137 ((*R*,*R*)-**Cu7**), 2366138 ((*R*,*R*)-**Cu11**), 2366139 ((*R*,*S*)-**Ru3**) and 2355628 ((*R*)-**Ru0**). Copies of the data can be obtained free of charge on application to the CCDC via https://www.ccdc.cam.ac.uk/data_request/cif.

The suitable crystals were selected and mounted on a SuperNova, Dual, Cu at home/near, Atlas diffractometer. The crystals were kept at a steady T = 140.00(10) K during data collection. The structure was solved with the **ShelXT**[15] solution program using dual methods and by using **Olex2** 1.5[16] as the graphical interface. The model was refined with **ShelXL** 2018/3[17] using full matrix least squares minimization on $\boldsymbol{F}^2$. Data were measured using ω scans with Cu K_α radiation. The diffraction pattern was indexed and the total number of runs and images was based on the strategy calculation from the program CrysAlis^{Pro} 1.171.41.123a (*Rigaku OD*, **2022**). All non-hydrogen atoms were refined anisotropically. Hydrogen atom positions were calculated geometrically and refined using the riding model.

Suitable crystals for X-Ray analysis of (*R*,*S*)-**Cu1**, (*R*,*R*)-**Cu1**, (*R*,*R*)-**Cu7** and (*R*,*R*)-**Cu11** were obtained by slow evaporation of a 3:1 hexane/DCM mixture in a nitrogen-filled glovebox.

(*R*,*S*)-**Cu1**, (*R*,*R*)-**Cu1**, (*R*,*R*)-**Cu7**, (*R*,*R*)-**Cu11** and (*R*,*S*)-**Ru3** contain a single molecule in their respective asymmetric unit.

(*R*)-**Ru0** contain two independent molecules in the asymmetric unit. A solvent mask was calculated and 423 electrons were found in a volume of 1247 Å^3 in one void per unit cell. This is consistent with the presence of two solvent molecules of hexane per asymmetric unit which accounts for 384 electrons per unit cell.

(*R*,*R*)-**Cu7** and (*R*)-**Ru0** were refined as a 2-component inversion twin.

Table S1. Crystal data for (*R*,*S*)-**Cu1** and (*R*,*R*)-**Cu1**.

**Compound**	(*R*,*S*)-**Cu1**	(*R*,*R*)-**Cu1**
Formula	$C_{21}H_{31}ClCuN$	$C_{21}H_{31}ClCuN$
$D_{calc.}$/ g cm^{-3}	1.306	1.301
μ/mm^{-1}	2.735	2.725
Formula Weight	396.46	396.46
Color	colorless	colorless
Shape	plate-shaped	needle-shaped
Size/mm^3	0.52×0.32×0.08	0.66×0.06×0.04
T/K	140.00(10)	140.00(10)
Crystal System	orthorhombic	monoclinic
Flack Parameter	-0.007(7)	-0.028(16)
Space Group	$P2_12_12_1$	$P2_1$
a/Å	8.14215(12)	9.6227(3)
b/Å	11.39563(20)	8.2304(3)
c/Å	21.7386(3)	12.9158(3)
$\alpha/^\circ$	90	90
$\beta/^\circ$	90	98.352(2)
$\gamma/^\circ$	90	90
V/Å^3	2017.01(6)	1012.06(5)
Z	4	2
Z'	1	1
Wavelength/Å	1.54184	1.54184
Radiation type	Cu K_α	Cu K_α
$\Theta_{min}/^\circ$	4.381	3.459
$\Theta_{max}/^\circ$	76.540	76.457
Measured Refl's.	10571	11385
Indep't Refl's	4192	3906
Refl's I≥2 σ(I)	4100	3671
R_{int}	0.0169	0.0330
Parameters	222	221
Restraints	0	1
Largest Peak	0.227	0.298
Deepest Hole	-0.237	-0.294
GooF	1.034	1.046
wR_2 (all data)	0.0544	0.0777
wR_2	0.0538	0.0756
R_1 (all data)	0.0211	0.0343
R_1	0.0202	0.0310
CCDC number	**2193067**	**2193068**

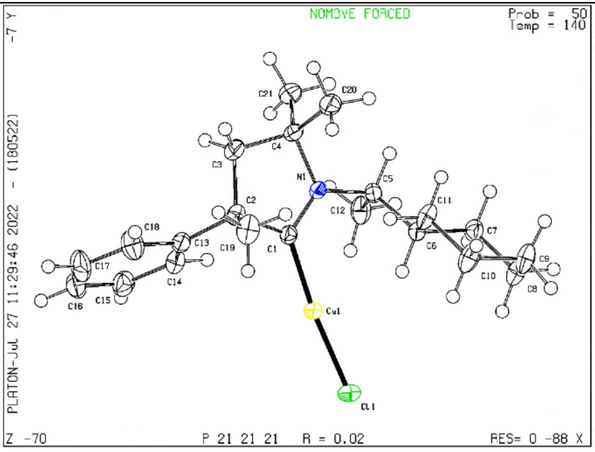

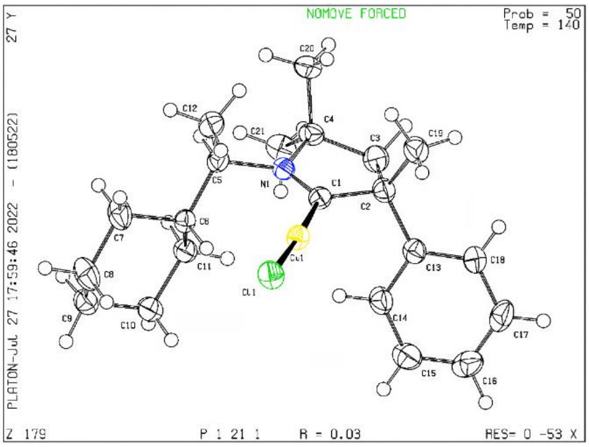

Table S2. Crystal data for (*R*,*R*)-**Cu7** and (*R*,*R*)-**Cu11**.

**Compound**	(*R*,*R*)-**Cu7**	(*R*,*R*)-**Cu11**
Formula	$C_{23}H_{35}ClCuN$	$C_{23}H_{35}ClCuNS$
$D_{calc.}$/ g cm^{-3}	1.244	1.303
μ/mm^{-1}	2.467	3.255
Formula Weight	424.51	456.57
Color	clear pale colorless	clear pale colorless
Shape	irregular-shaped	plate
Size/mm^3	0.37×0.25×0.21	0.40×0.12×0.09
T/K	200.00(10)	140.0(3)
Crystal System	orthorhombic	monoclinic
Flack Parameter	0.12(7)	0.01(3)
Hooft Parameter	-0.04(2)	0.01(3)
Space Group	$P2_12_12_1$	$P2_1$
a/Å	10.5297(5)	8.5182(2)
b/Å	13.2008(7)	15.3074(5)
c/Å	16.3003(7)	9.1646(3)
α/°	90	90
β/°	90	103.104(3)
γ/°	90	90
V/Å^3	2265.75(19)	1163.87(6)
Z	4	2
Z'	1	1
Wavelength/Å	1.54184	1.54184
Radiation type	Cu K$_\alpha$	Cu K$_\alpha$
Θ_{min}/°	4.310	4.955
Θ_{max}/°	71.975	73.322
Measured Refl's.	9478	10462
Indep't Refl's	4349	4323
Refl's I≥2 σ(I)	3876	4127
R_{int}	0.0688	0.0465
Parameters	307	248
Restraints	250	1
Largest Peak	0.840	1.344
Deepest Hole	-0.394	-0.555
GooF	1.061	1.041
wR_2 (all data)	0.2470	0.1234
wR_2	0.2395	0.1217
R_1 (all data)	0.0905	0.0493
R_1	0.0855	0.0472
CCDC number	**2366137**	**2366138**

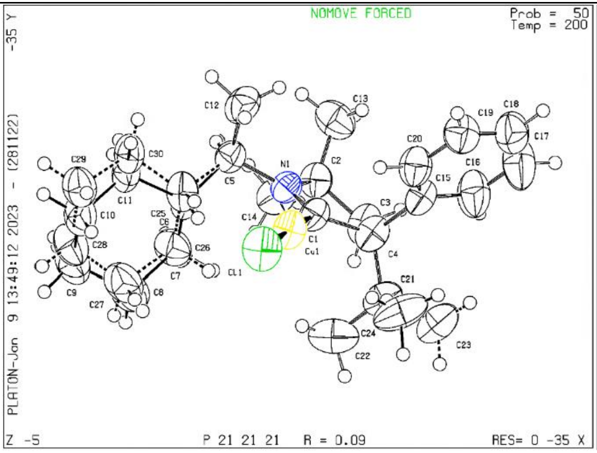

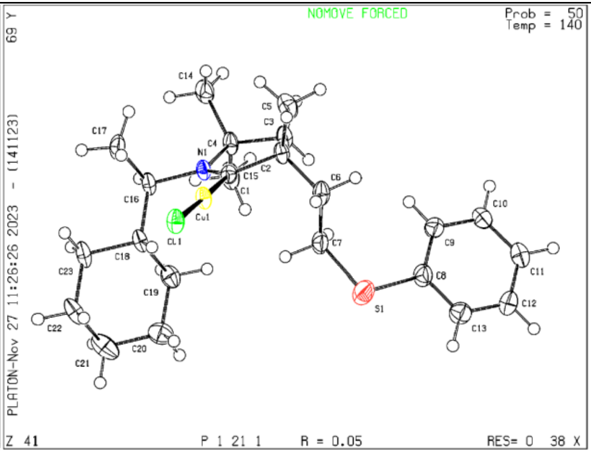

Table S3. Crystal data for (*R*)-**Ru0** and (*R*,*S*)-**Ru3**.

**Compound**	(*R*,S)-**Ru3**	(*R*)-**Ru0**
Formula	$C_{35}H_{45}NOCl_2Ru$	$C_{48}H_{69}Cl_2NORu$
$D_{calc.}$/ g cm^{-3}	1.346	1.310
μ/mm^{-1}	5.538	4.357
Formula Weight	667.69	848.01
Color	clear intense green	clear intense green
Shape	irregular	needle-shaped
Size/mm^3	0.17×0.12×0.06	0.29×0.04×0.02
T/K	139.99(10)	140.00(10)
Crystal System	orthorhombic	monoclinic
Flack Parameter	-0.037(7)	0.248(18)
Hooft Parameter	-0.037(7)	-
Space Group	$P2_12_12_1$	$P2_1$
a/Å	11.5043(3)	18.5489(9)
b/Å	16.0340(4)	8.9270(4)
c/Å	17.8672(5)	27.5267(11)
α/°	90	90
β/°	90	109.441(5)
γ/°	90	90
V/Å^3	3295.78(15)	4298.1(3)
Z	4	4
Z'	1	2
Wavelength/Å	1.54184	1.54184
Radiation type	Cu K_α	Cu K_α
Θ_{min}/°	3.704	2.526
Θ_{max}/°	75.776	75.351
Measured Refl's.	38532	46618
Indep't Refl's	6742	16645
Refl's I≥2 σ(I)	5827	13986
R_{int}	0.0565	0.0516
Parameters	368	678
Restraints	0	1202
Largest Peak	1.458	1.188
Deepest Hole	-1.661	-1.856
GooF	1.065	1.088
wR_2 (all data)	0.1448	0.2261
wR_2	0.1370	0.2177
R_1 (all data)	0.0659	0.0966
R_1	0.0550	0.0852
CCDC number	**2366139**	**2355628**

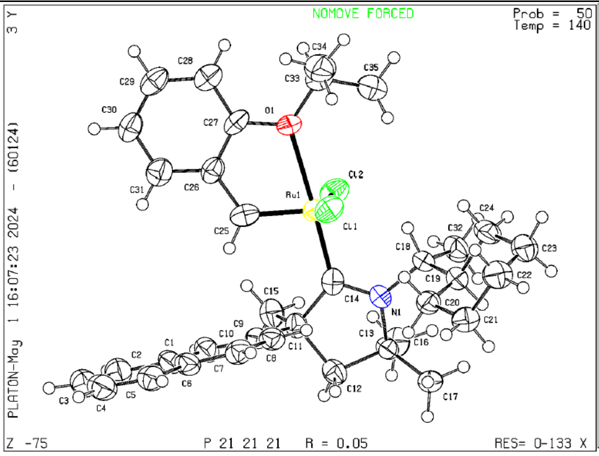

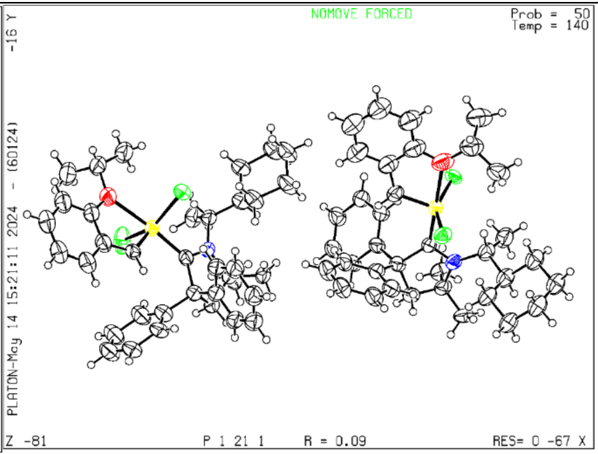

References

1 E. A. Doud, M. S. Inkpen, G. Lovat, E. Montes, D. W. Paley, M. L. Steigerwald, H. Vázquez, L. Venkataraman and X. Roy, *J. Am. Chem. Soc.*, 2018, **140**, 8944–8949.

2 F. Sánchez-Cantalejo, J. D. Priest and P. W. Davies, *Chem. - A Eur. J.*, 2018, **24**, 17215–17219.

3 D. R. Baghurst, D. Michael, P. Mingos and M. J. Watson, *J. Organomet. Chem.*, 1989, **368**, C43–C45.

4 G. R. Fulmer, A. J. M. Miller, N. H. Sherden, H. E. Gottlieb, A. Nudelman, B. M. Stoltz, J. E. Bercaw and K. I. Goldberg, *Organometallics*, 2010, **29**, 2176–2179.

5 Y. Schramm, F. Barrios-Landeros and A. Pfaltz, *Chem. Sci.*, 2013, **4**, 2760.

6 P. V. Ramachandran, A. A. Alawaed and H. J. Hamann, *J. Org. Chem.*, 2022, **87**, 13259–13269.

7 J.-L. Tu, A.-M. Hu, L. Guo and W. Xia, *J. Am. Chem. Soc.*, 2023, **145**, 7600–7611.

8 T. Borg, J. Danielsson, M. Mohiti, P. Restorp and P. Somfai, *Adv. Synth. Catal.*, 2011, **353**, 2022–2036.

9 M. Yoshida, H. Otaka and T. Doi, *Eur. J. Org. Chem.*, 2014, **2014**, 6010–6016.

10 D. Pichon, M. Soleilhavoup, J. Morvan, G. P. Junor, T. Vives, C. Crévisy, V. Lavallo, J. M. Campagne, M. Mauduit, R. Jazzar and G. Bertrand, *Chem. Sci.*, 2019, **10**, 7807–7811.

11 H. Y. Jung, X. Feng, H. Kim and J. Yun, in *Tetrahedron*, 2012, vol. 68, pp. 3444–3449.

12 I. Shiina, Y. Umezaki, N. Kuroda, T. Iizumi, S. Nagai and T. Katoh, *J. Org. Chem.*, 2012, **77**, 4885–4901.

13 K. Toribatake, L. Zhou, A. Tsuruta and H. Nishiyama, *Tetrahedron*, 2013, **69**, 3551–3560.

14 T. W. Funk, J. M. Berlin and R. H. Grubbs, *J. Am. Chem. Soc.*, 2006, **128**, 1840–1846.

15 G. M. Sheldrick, *Acta Crystallogr. Sect. A Found. Adv.*, 2015, **71**, 3–8.

16 O. V. Dolomanov, L. J. Bourhis, R. J. Gildea, J. A. K. Howard and H. Puschmann, *J. Appl. Crystallogr.*, 2009, **42**, 339–341.

17 G. M. Sheldrick, *Acta Crystallogr. Sect. C Struct. Chem.*, 2015, **71**, 3–8.

NMR Spectra

^{1}H NMR ($CDCl_3$, 400 MHz)

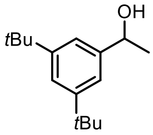

Crude S-IIa

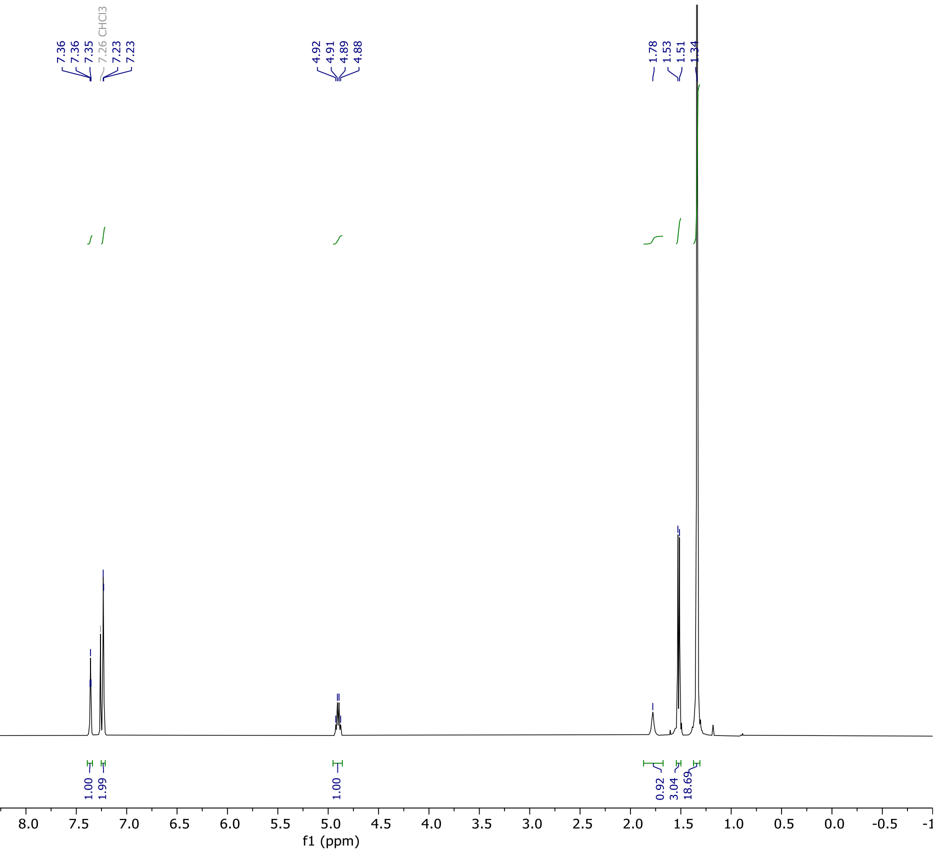

^{1}H NMR ($CDCl_3$, 400 MHz)

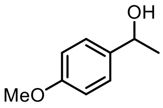

Crude S-IIb

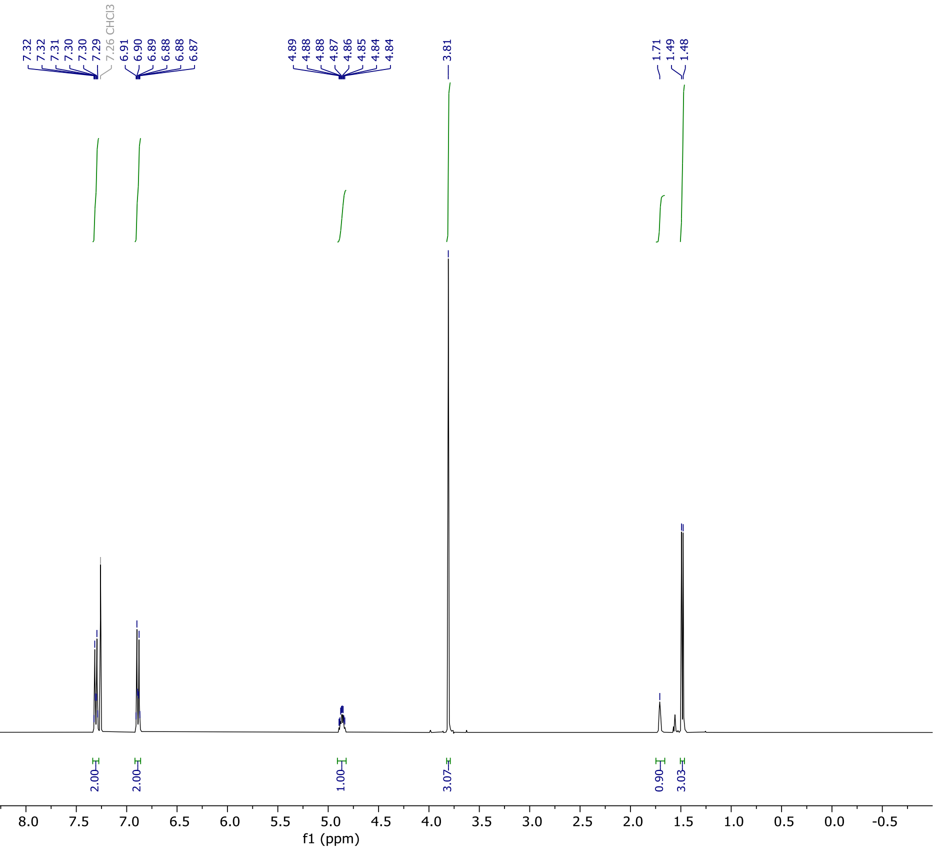

^{1}H NMR ($CDCl_3$, 400 MHz)

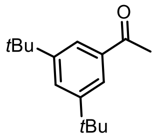

Crude S-IIIa

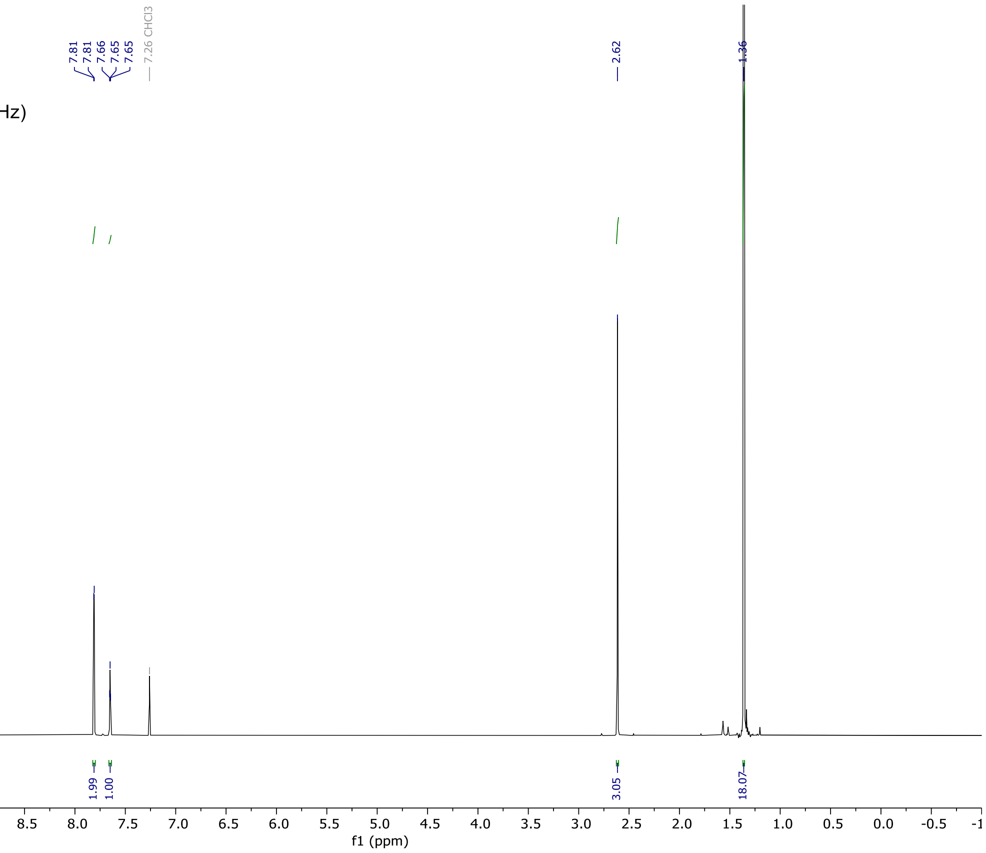

^{1}H NMR ($CDCl_3$, 400 MHz)

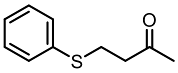

Crude S-IV

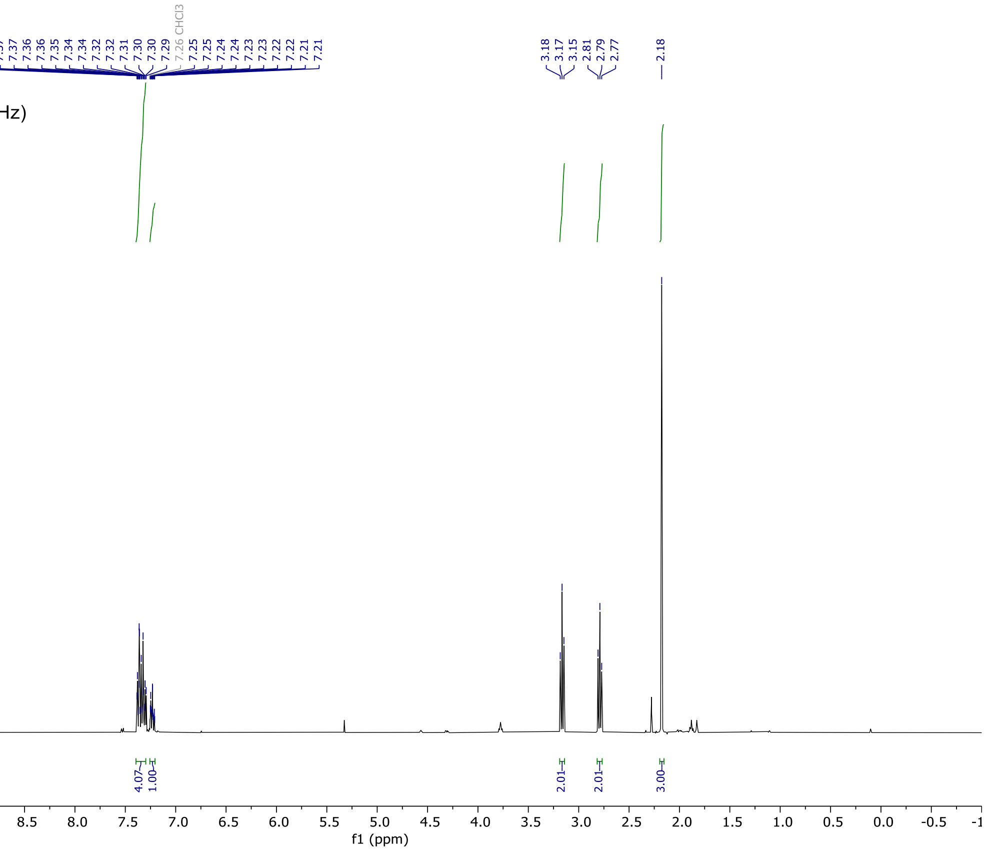

^{1}H NMR ($CDCl_3$, 400 MHz)

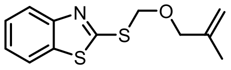

2

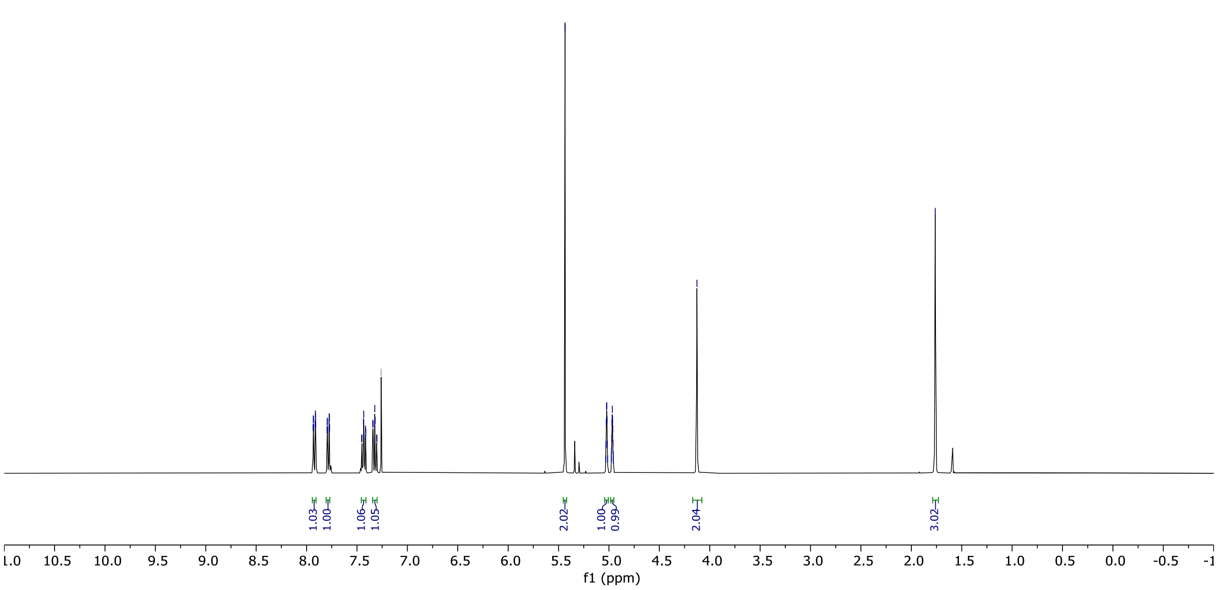

^{13}C NMR ($CDCl_3$, 101 MHz)

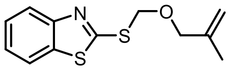

2

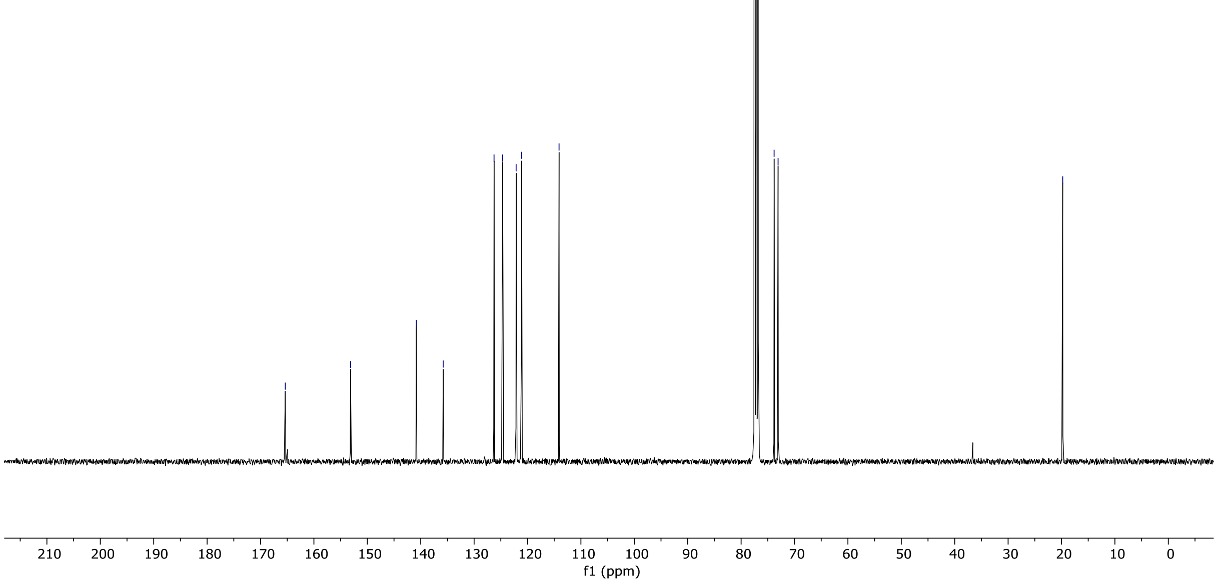

^{1}H NMR ($CDCl_3$, 400 MHz)

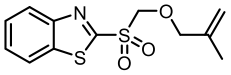

3

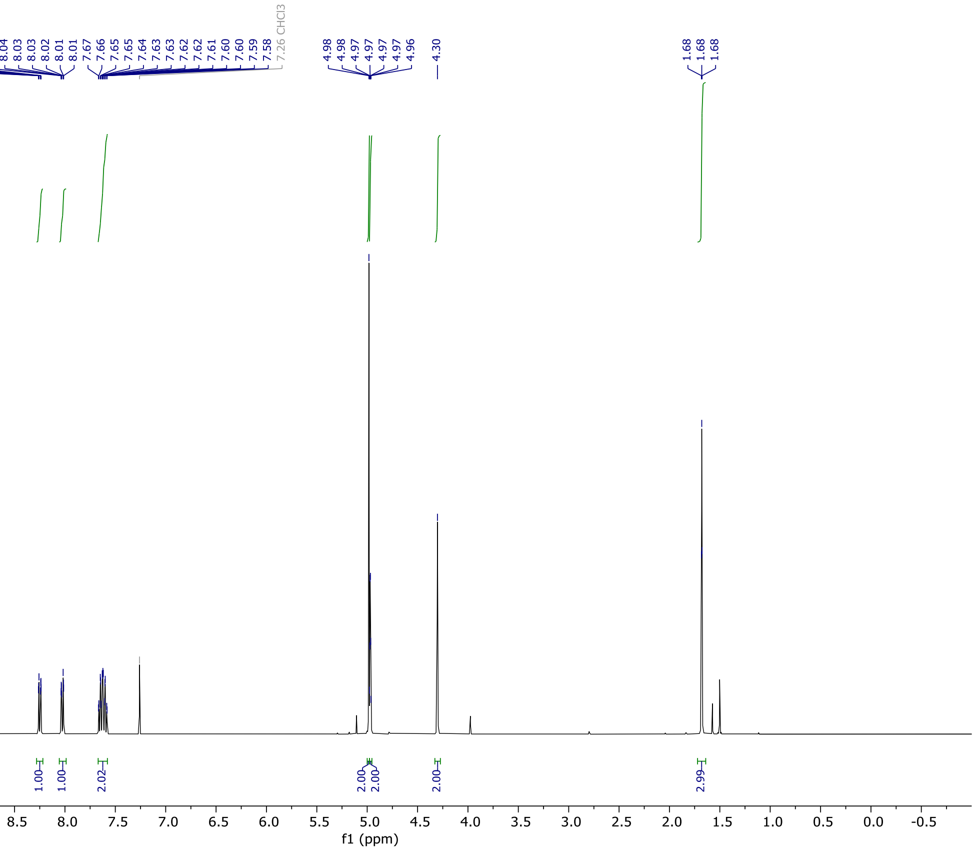

^{13}C NMR ($CDCl_3$, 101 MHz)

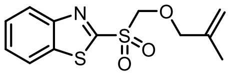

3

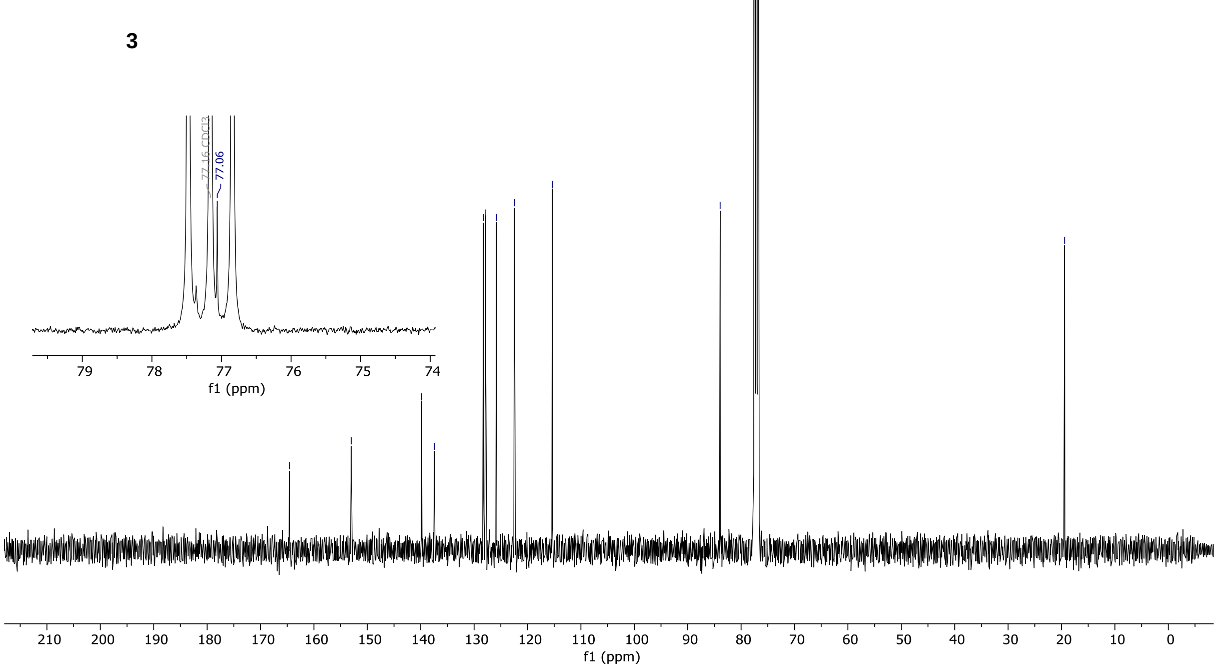

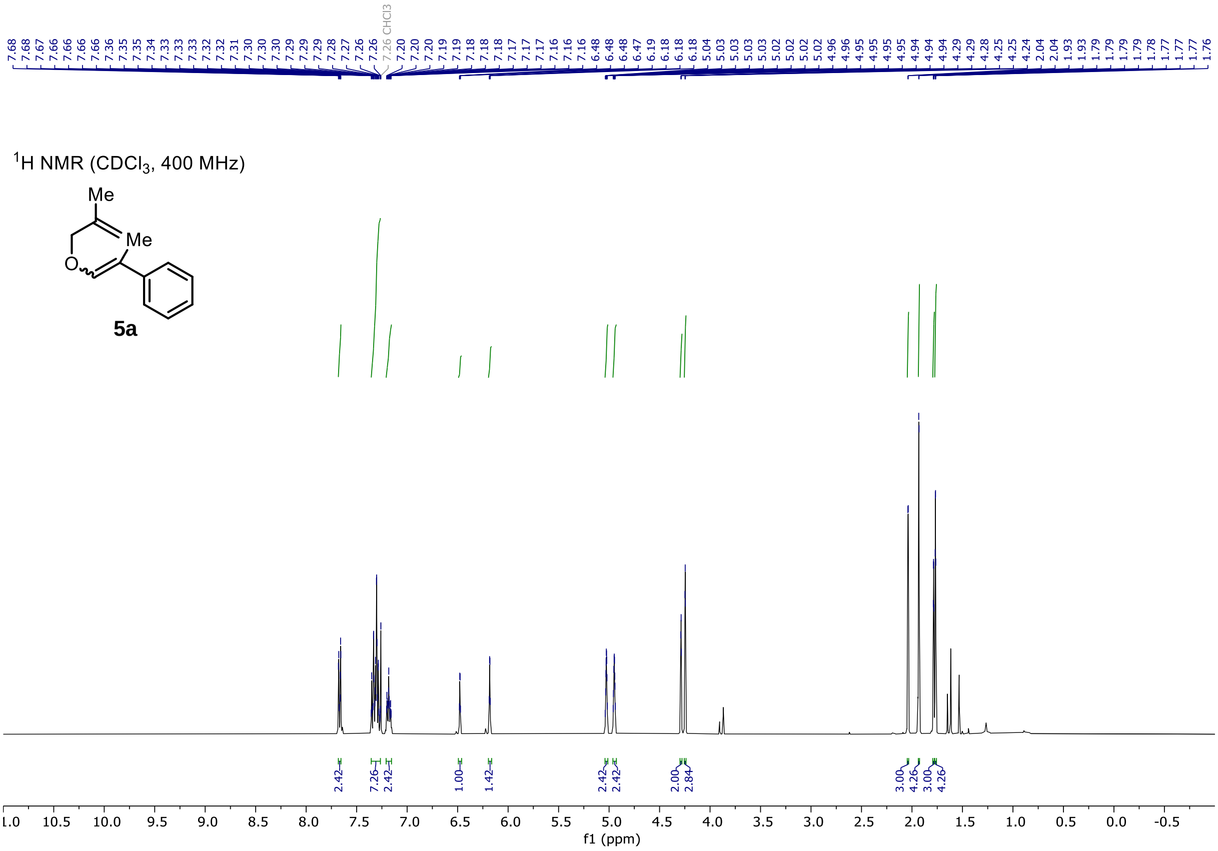
7.68 7.68 7.67 7.66 7.66 7.66 7.36 7.35 7.35 7.34 7.33 7.33 7.33 7.32 7.32 7.31 7.30 7.30 7.29 7.29 7.29 7.28 7.27 7.26 7.26 7.26 CHCl3 7.20 7.20 7.20 7.19 7.19 7.18 7.18 7.17 7.17 7.17 7.16 7.16 7.16 6.48 6.48 6.48 6.47 6.19 6.18 6.18 6.18 5.04 5.03 5.03 5.03 5.02 5.02 5.02 4.96 4.95 4.95 4.95 4.95 4.94 4.94 4.94 4.29 4.29 4.28 4.25 4.25 4.24 2.04 2.04 1.93 1.93 1.79 1.79 1.79 1.78 1.77 1.77 1.77 1.76
1H NMR (CDCl3, 400 MHz)
Me
Me
O
5a
2.42 7.26 2.42 1.00 1.42 2.42 2.42 2.00 2.84 3.00 4.26 3.00 4.26
f1 (ppm)

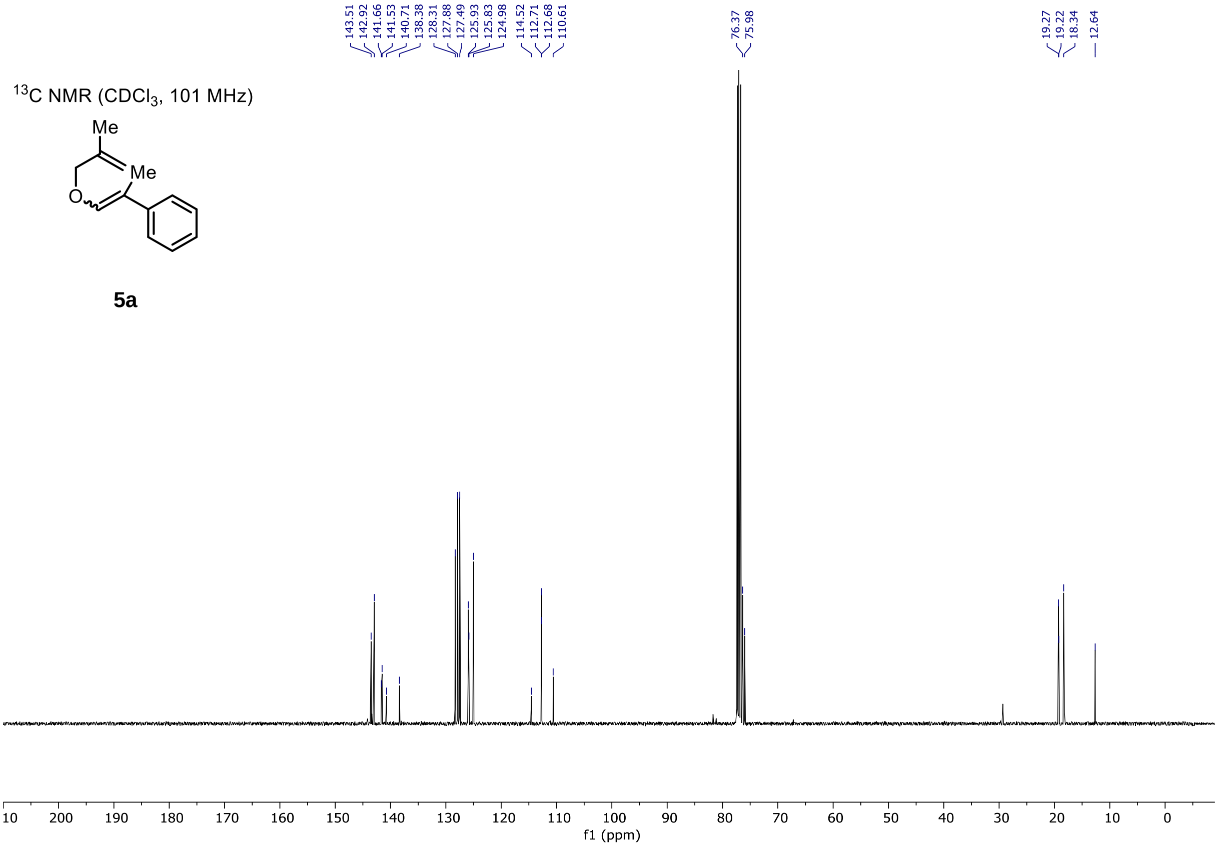
143.51 142.92 141.66 141.53 140.71 138.38 128.31 127.88 127.49 125.93 125.83 124.98 114.52 112.71 112.68 110.61
76.37 75.98
19.27 19.22 18.34 12.64
13C NMR (CDCl3, 101 MHz)
Me
Me
O
5a
f1 (ppm)

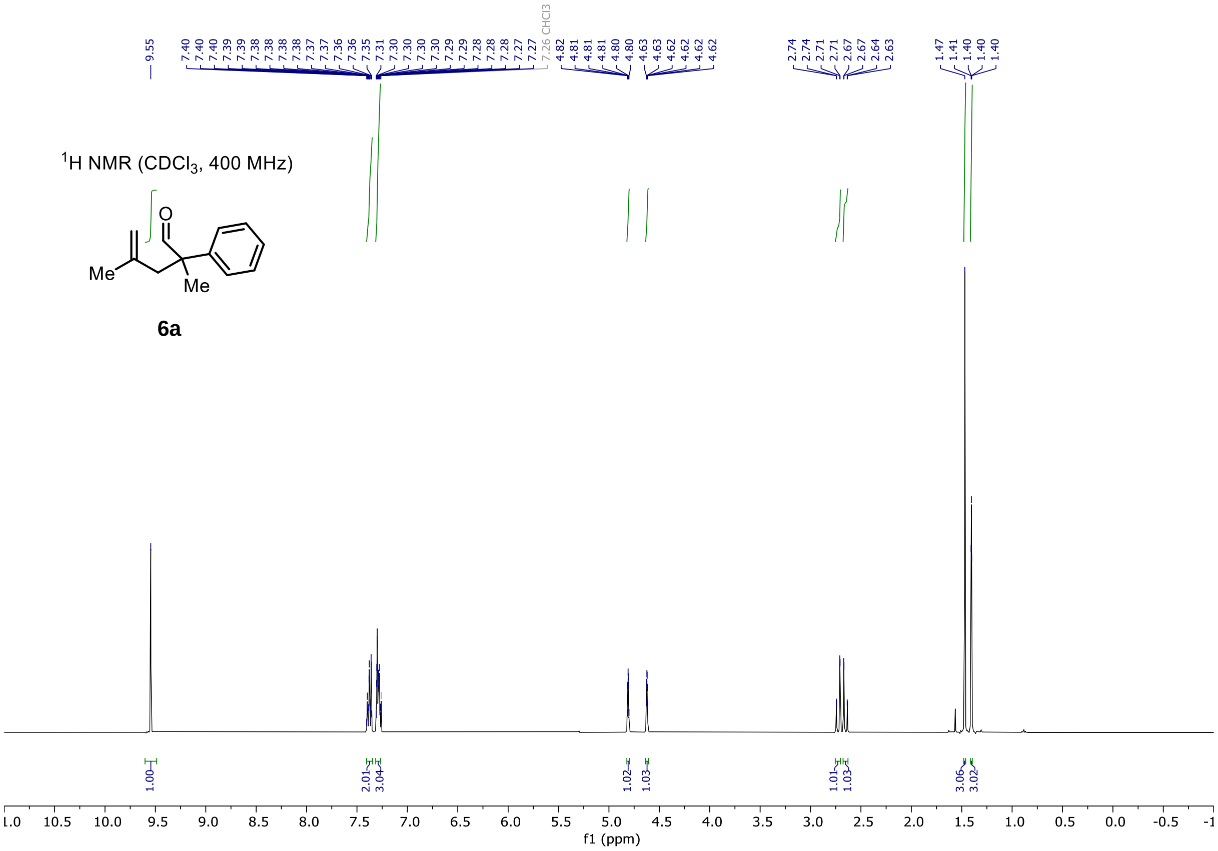
^{1}H NMR (CDCl$_3$, 400 MHz)
O
Me
Me
6a
f1 (ppm)

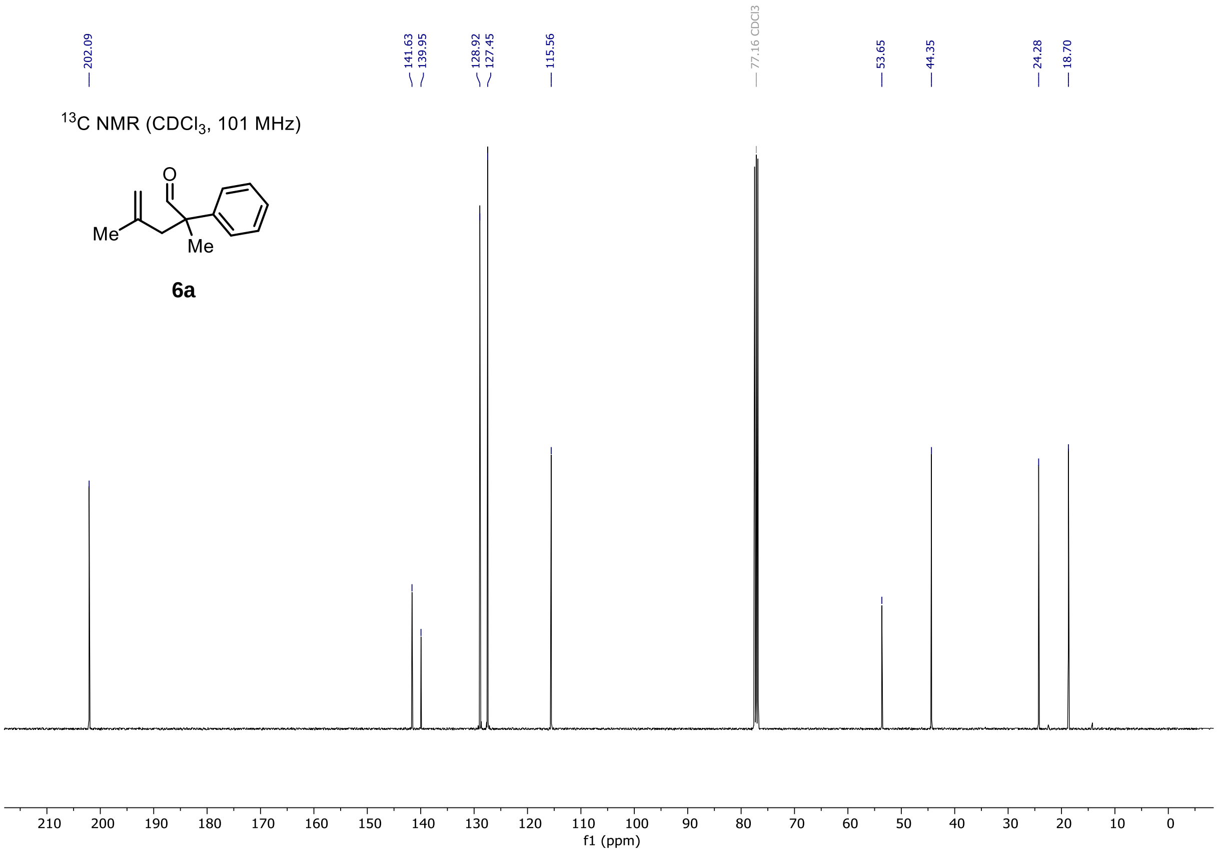
^{13}C NMR (CDCl$_3$, 101 MHz)
O
Me
Me
6a
f1 (ppm)

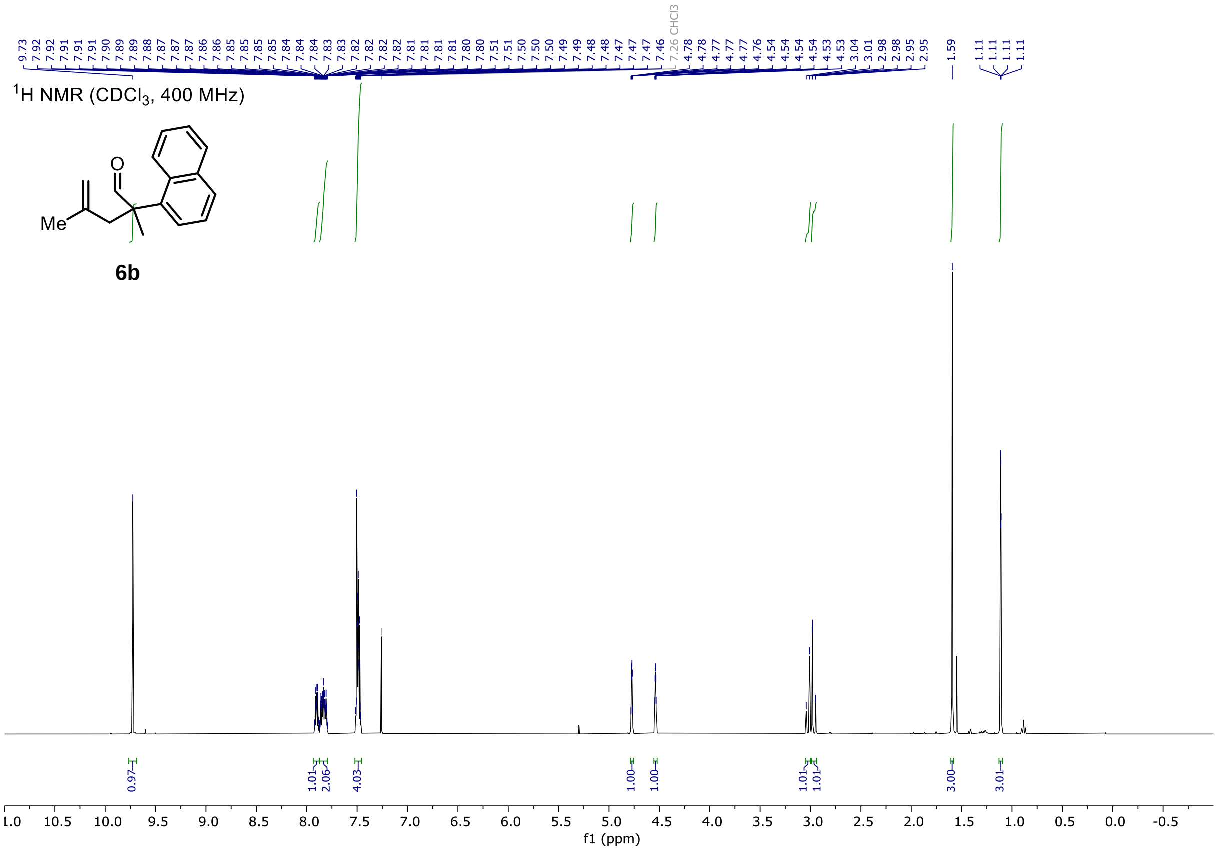
1H NMR (CDCl3, 400 MHz)
O
Me
6b
f1 (ppm)

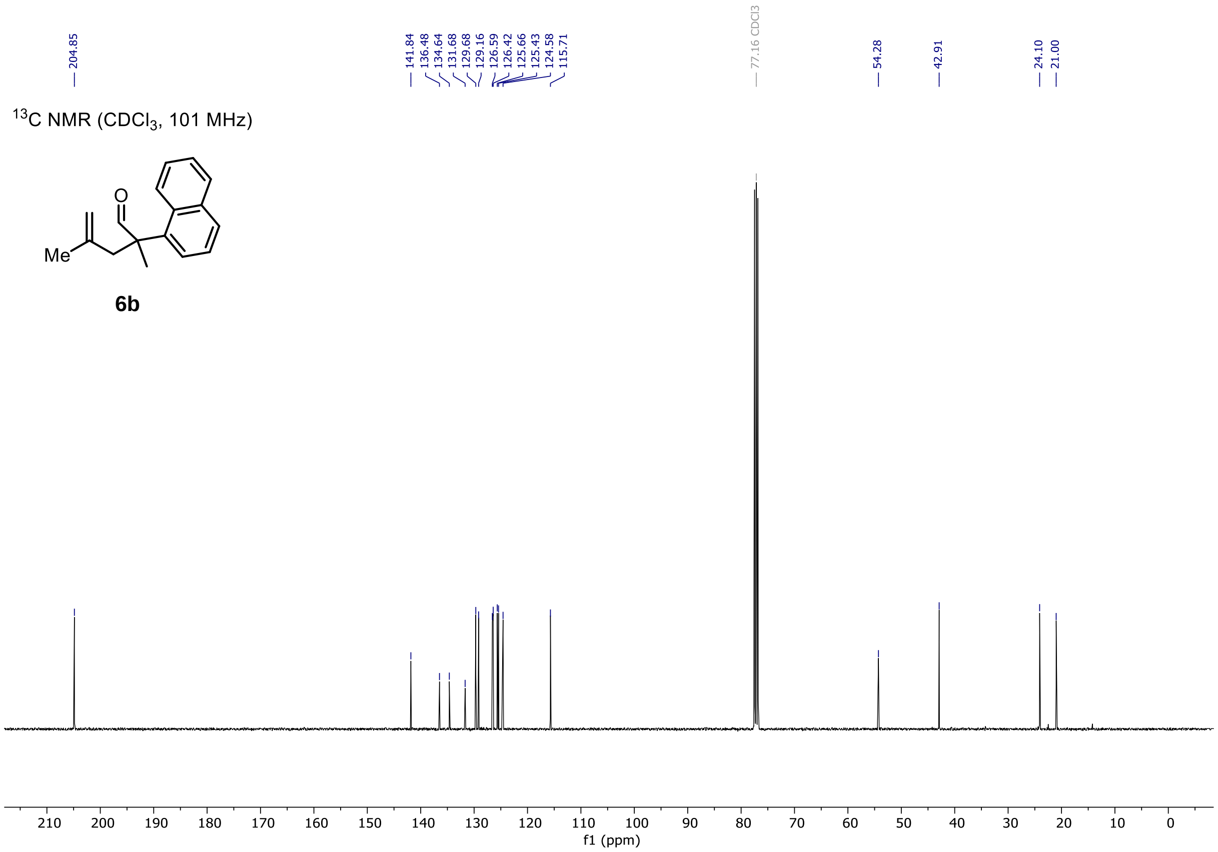
13C NMR (CDCl3, 101 MHz)
O
Me
6b
f1 (ppm)

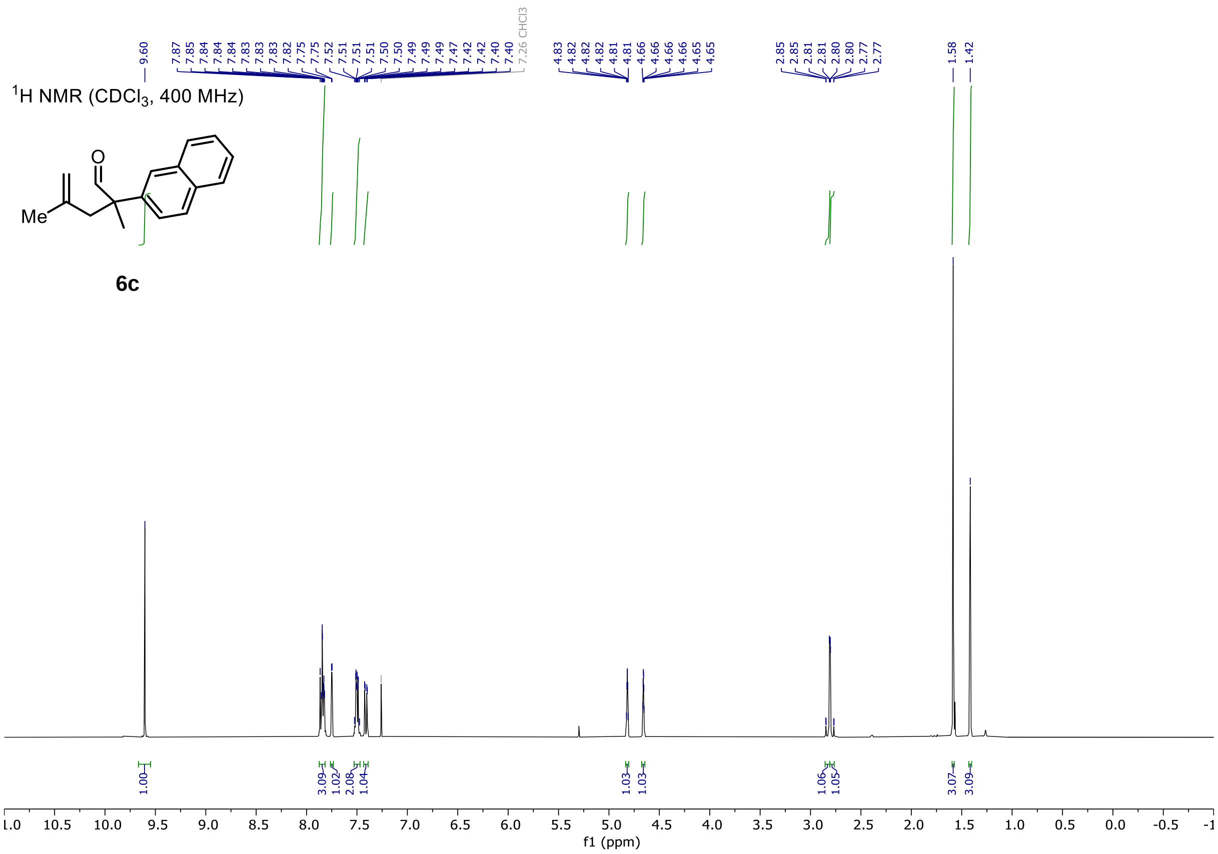
^{1}H NMR (CDCl$_3$, 400 MHz)
6c
f1 (ppm)

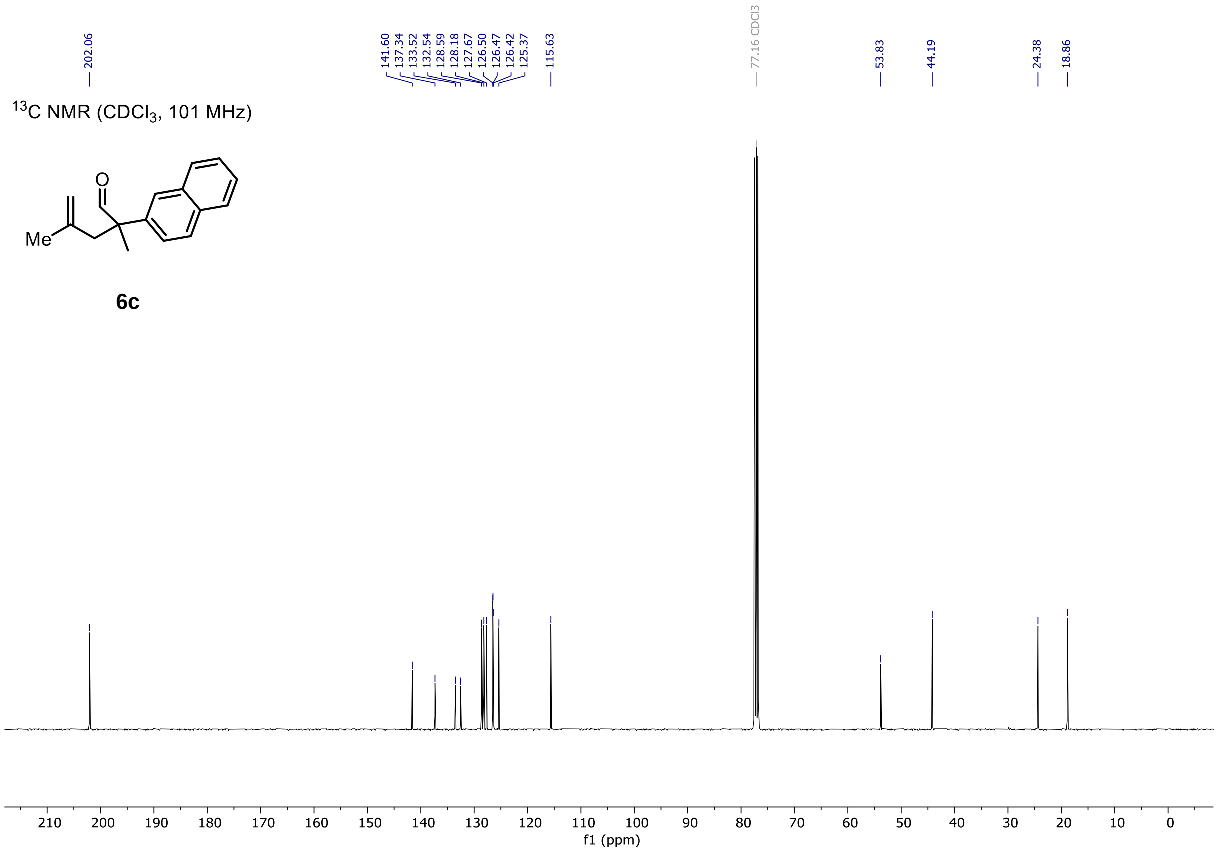
^{13}C NMR (CDCl$_3$, 101 MHz)
6c
f1 (ppm)

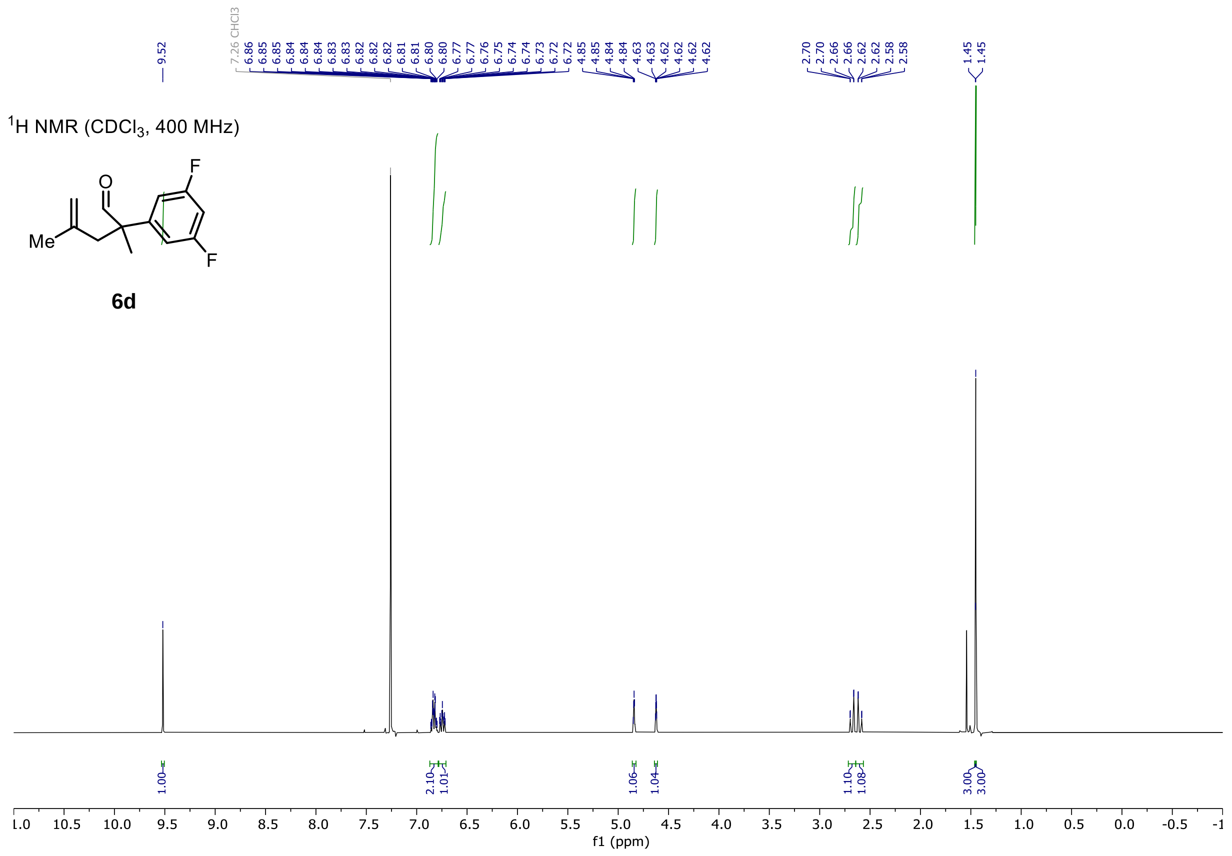

^{1}H NMR ($CDCl_3$, 400 MHz)
6d

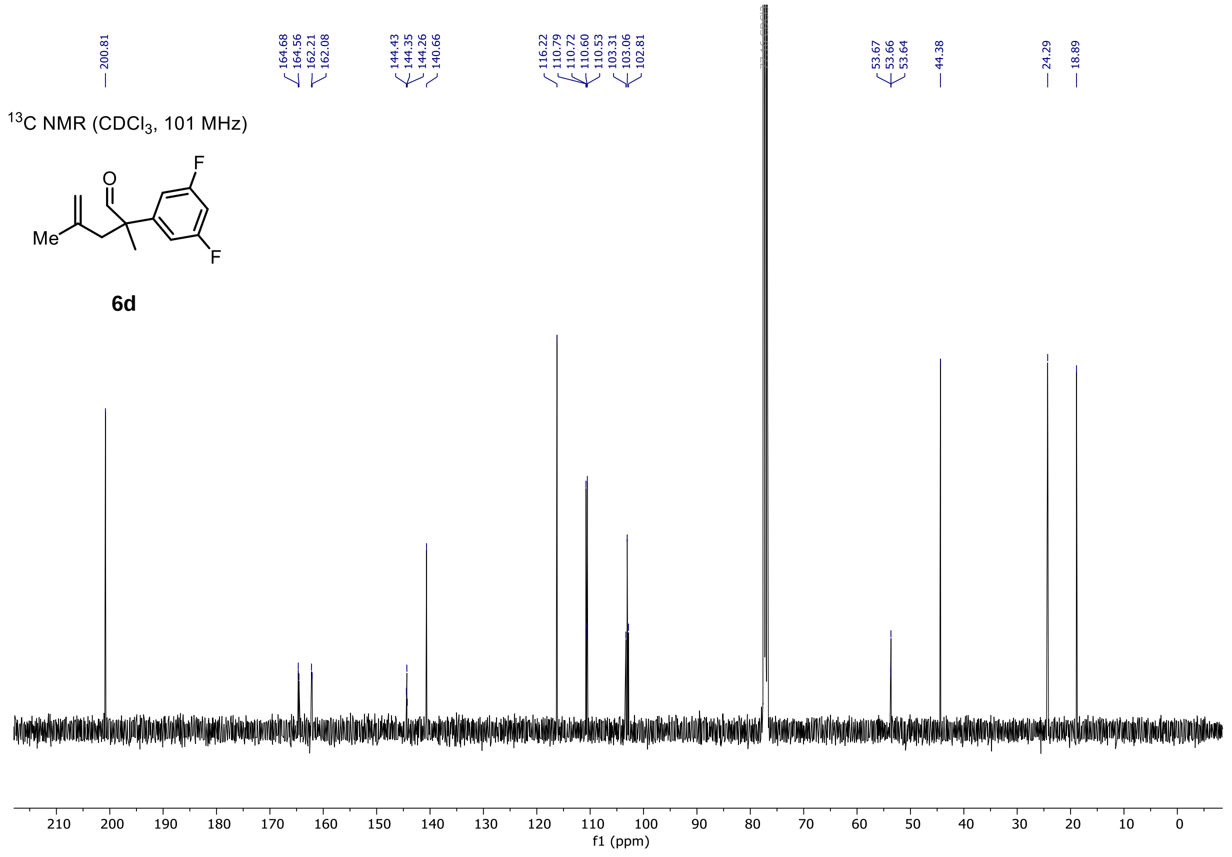

^{13}C NMR ($CDCl_3$, 101 MHz)
6d

^{19}F NMR ($CDCl_3$, 376 MHz)

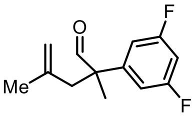

6d

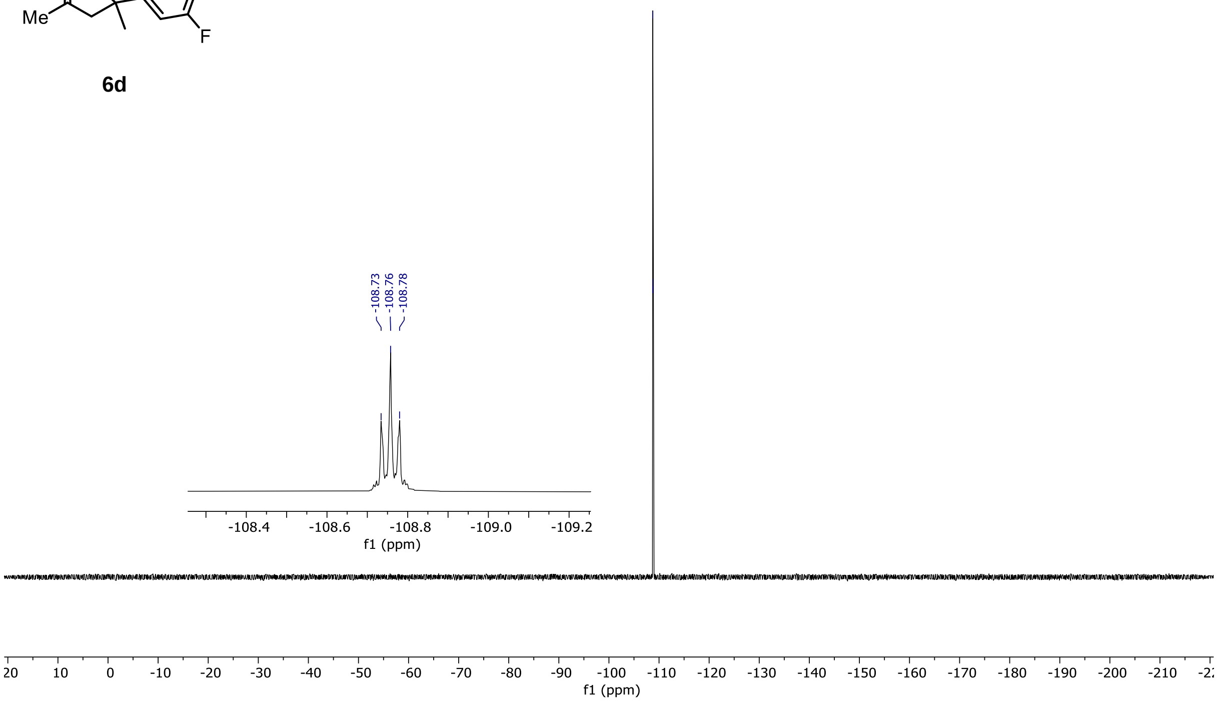

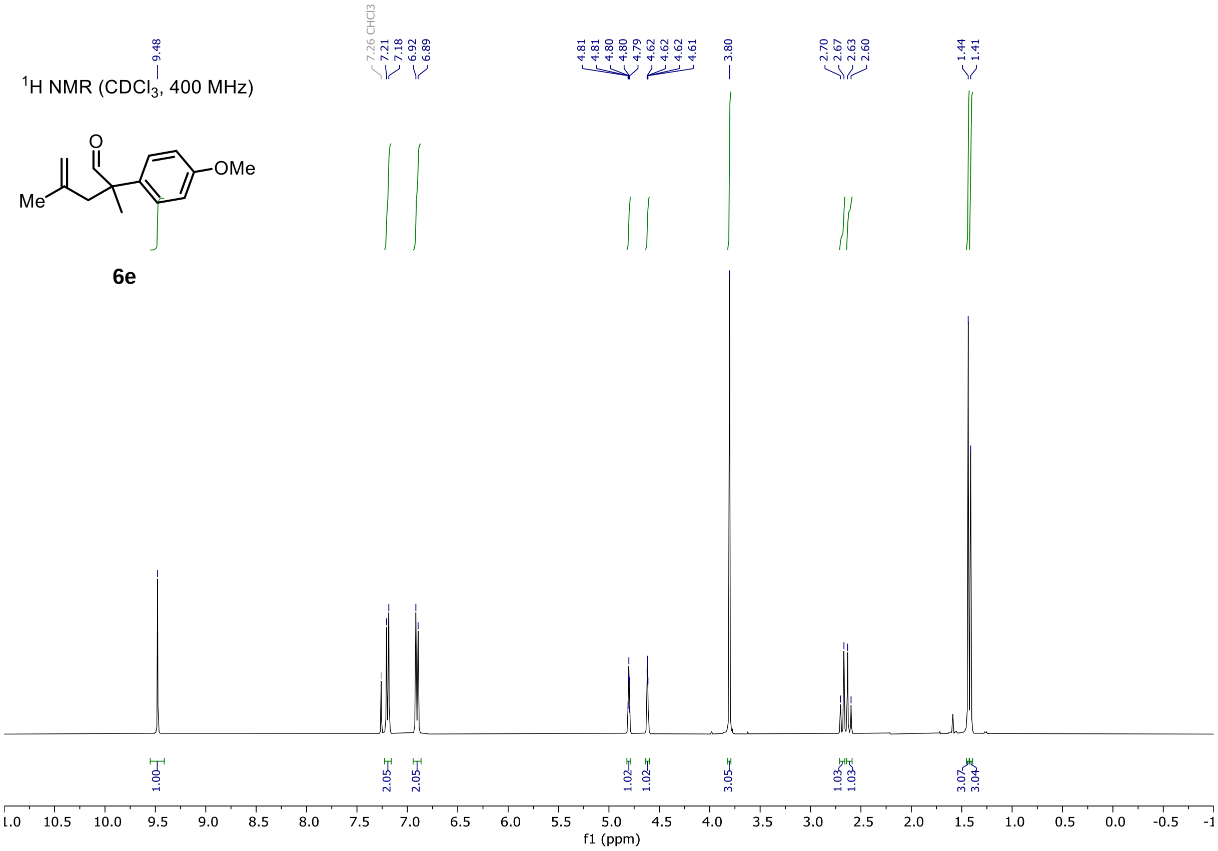
^{1}H NMR ($CDCl_3$, 400 MHz)
OMe
Me
6e
f1 (ppm)

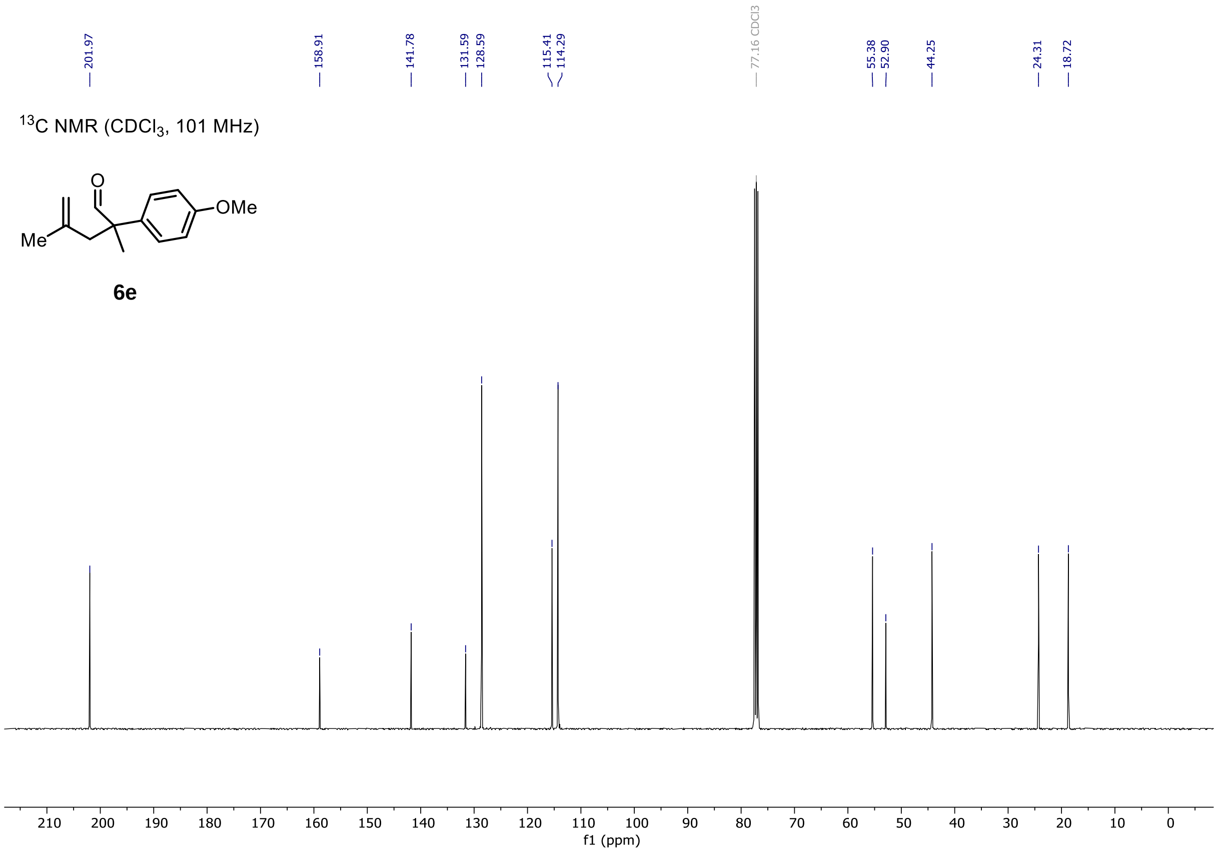
^{13}C NMR ($CDCl_3$, 101 MHz)
OMe
Me
6e
f1 (ppm)

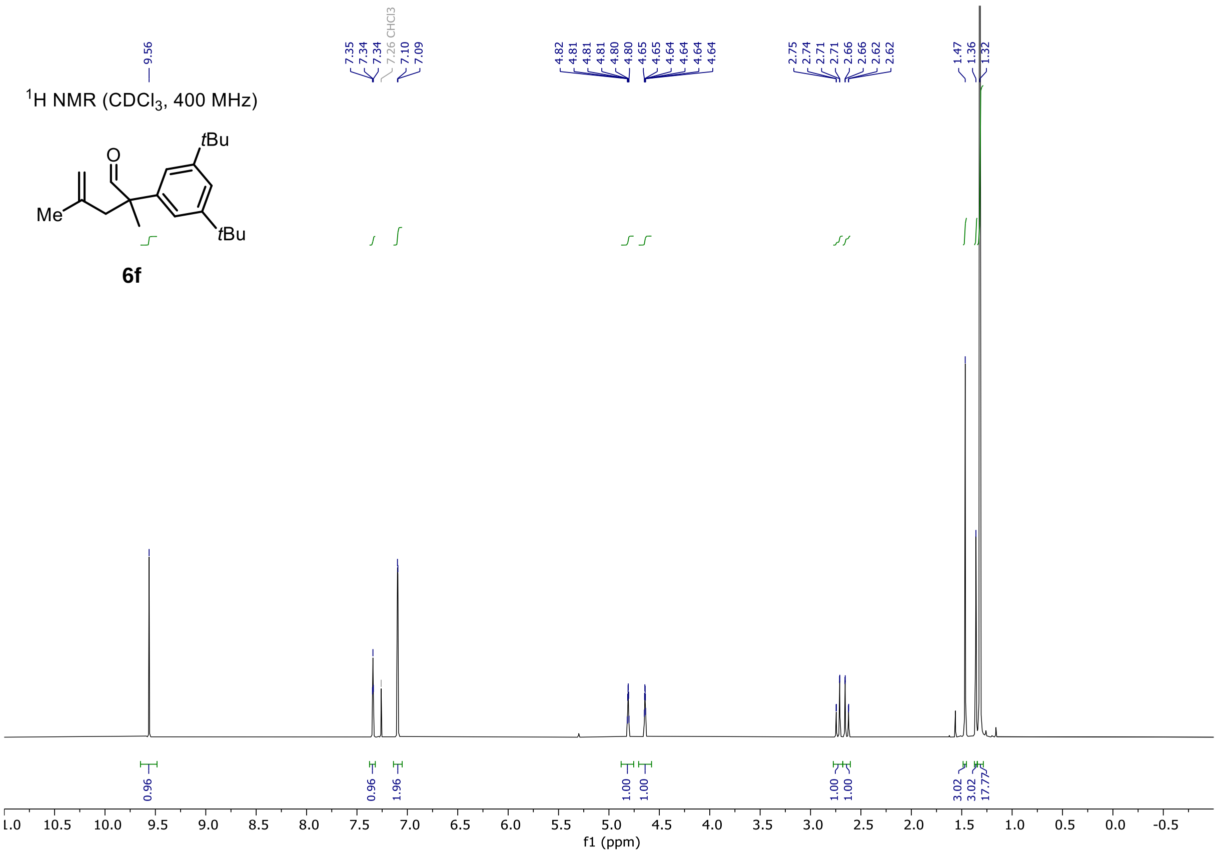

9.56
7.35
7.34
7.34
7.26 CHCl3
7.10
7.09
4.82
4.81
4.81
4.81
4.80
4.80
4.65
4.65
4.64
4.64
4.64
4.64
2.75
2.74
2.71
2.71
2.66
2.66
2.62
2.62
1.47
1.36
1.32
^{1}H NMR ($CDCl_3$, 400 MHz)
O
tBu
Me
tBu
6f
0.96
0.96
1.96
1.00
1.00
1.00
1.00
3.02
3.02
17.77
f1 (ppm)

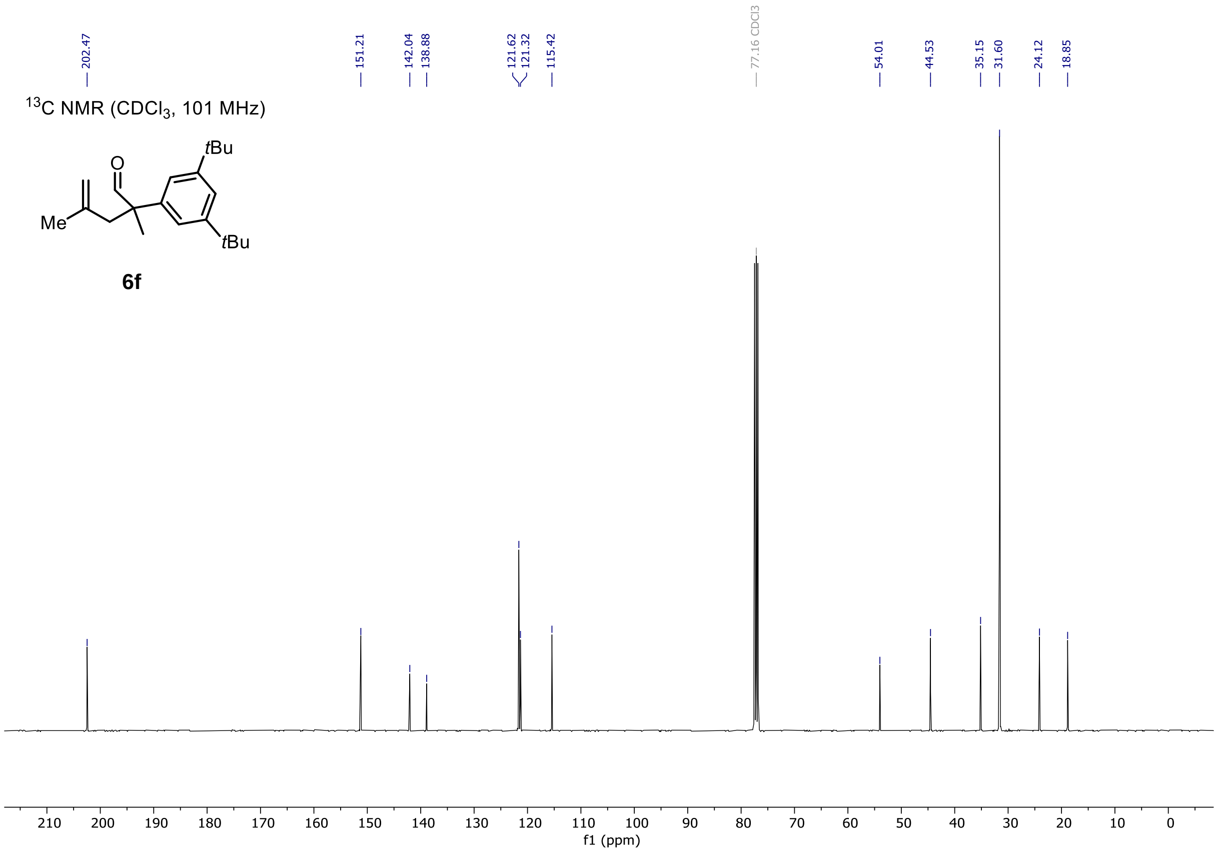

202.47
151.21
142.04
138.88
121.62
121.32
115.42
77.16 CDCl3
54.01
44.53
35.15
31.60
24.12
18.85
^{13}C NMR ($CDCl_3$, 101 MHz)
O
tBu
Me
tBu
6f
f1 (ppm)

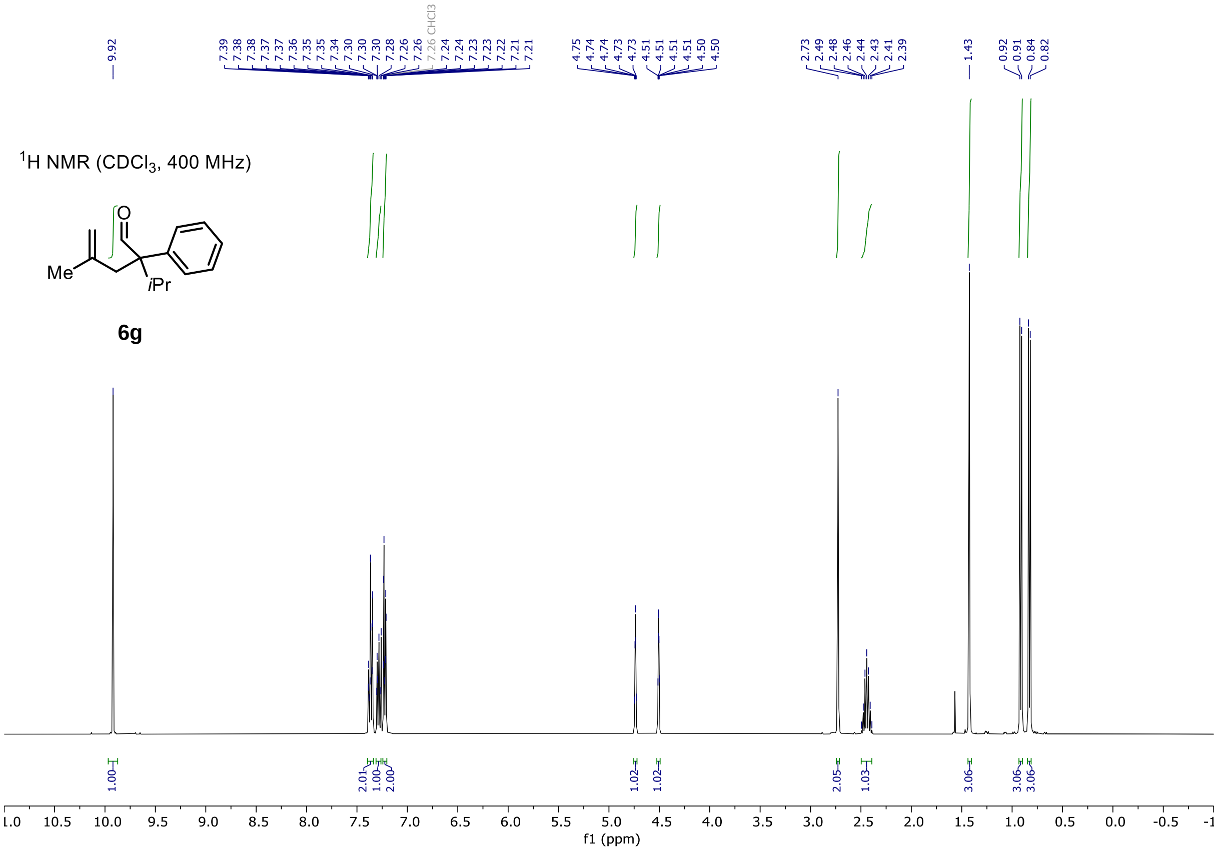
9.92
7.39 7.38 7.38 7.37 7.37 7.36 7.35 7.35 7.34 7.30 7.30 7.28 7.26 7.26 7.26 CHCl3 7.24 7.24 7.24 7.23 7.23 7.22 7.21 7.21
4.75 4.74 4.74 4.73 4.73 4.51 4.51 4.51 4.51 4.50 4.50
2.73 2.49 2.48 2.46 2.44 2.43 2.41 2.39
1.43
0.92 0.91 0.84 0.82
1H NMR (CDCl3, 400 MHz)
O
Me
iPr
6g
1.00
2.01 1.00 2.00
1.02 1.02
2.05 1.03
3.06
3.06 3.06
f1 (ppm)

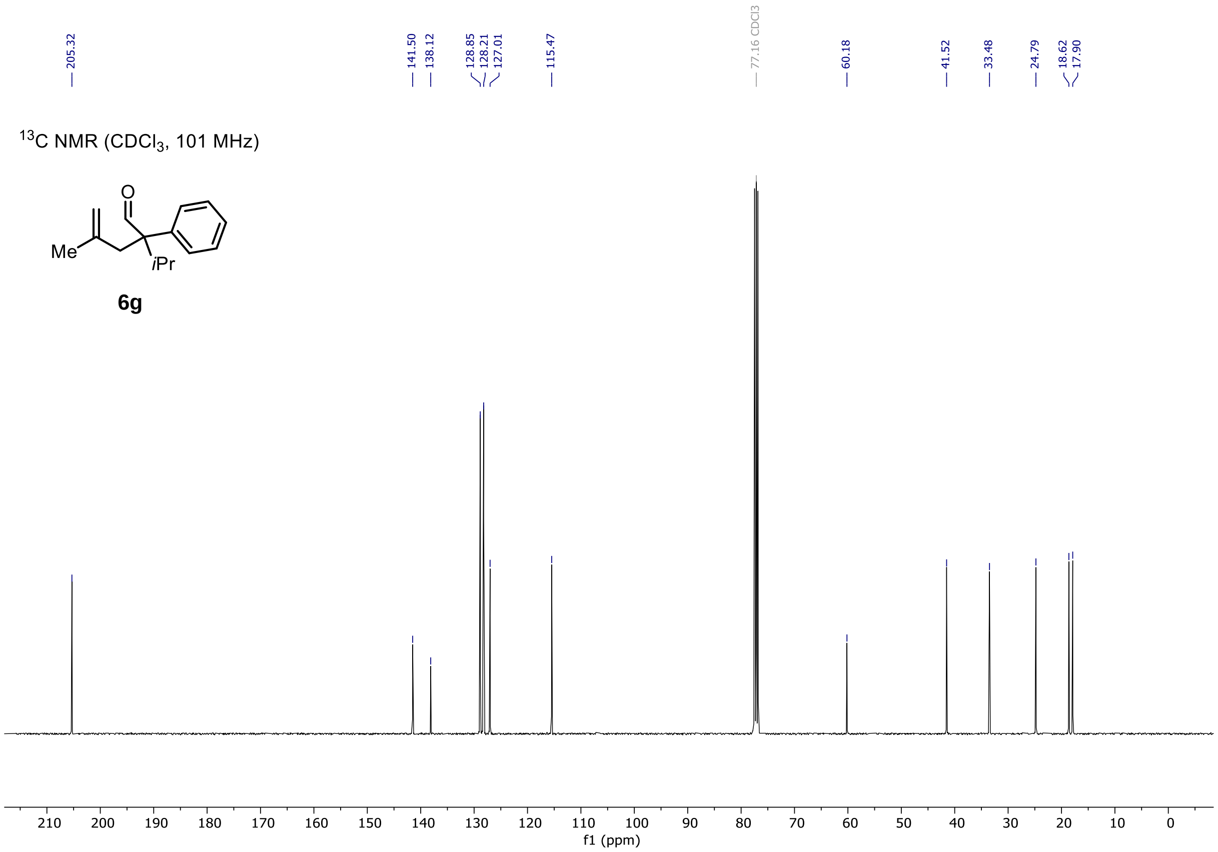
205.32
141.50 138.12
128.85 128.21 127.01
115.47
77.16 CDCl3
60.18
41.52
33.48
24.79
18.62 17.90
13C NMR (CDCl3, 101 MHz)
O
Me
iPr
6g
f1 (ppm)

^{1}H NMR ($CDCl_3$, 400 MHz)

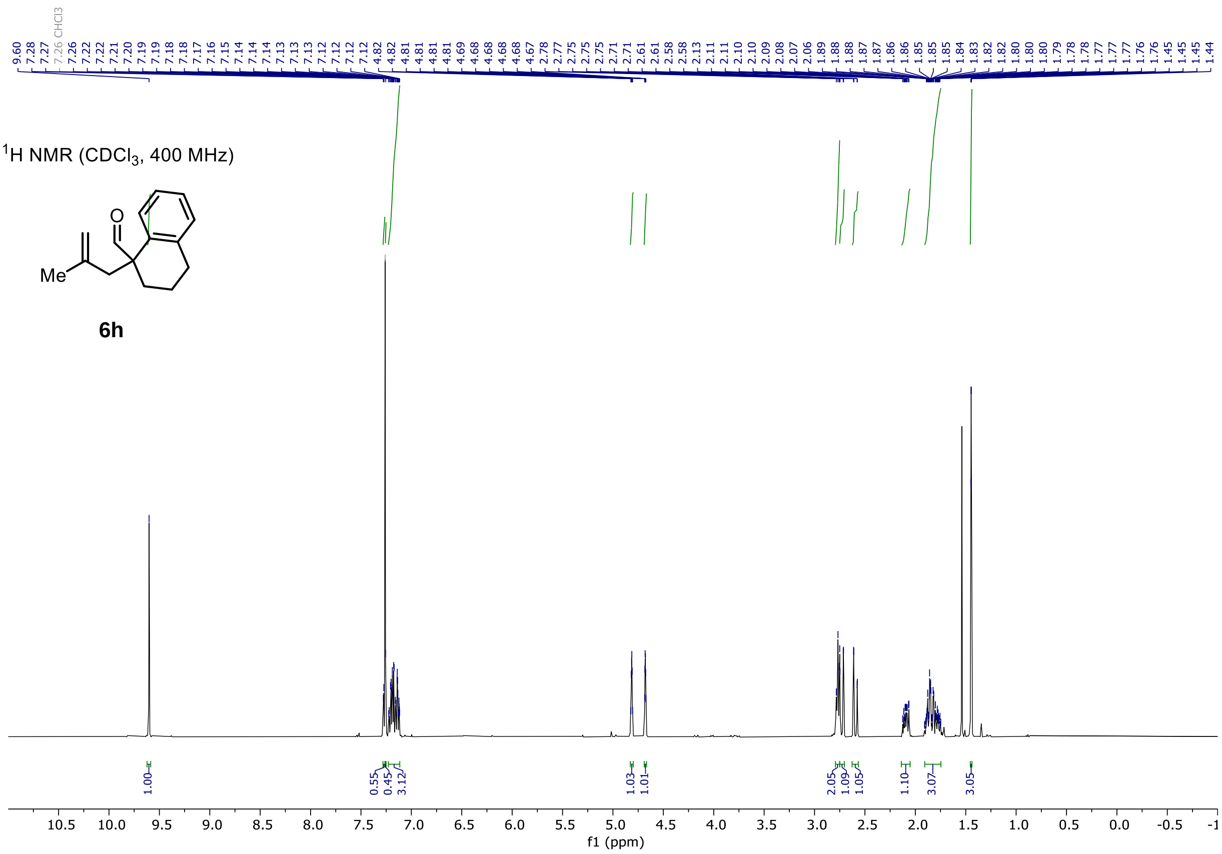

6h

^{13}C NMR ($CDCl_3$, 101 MHz)

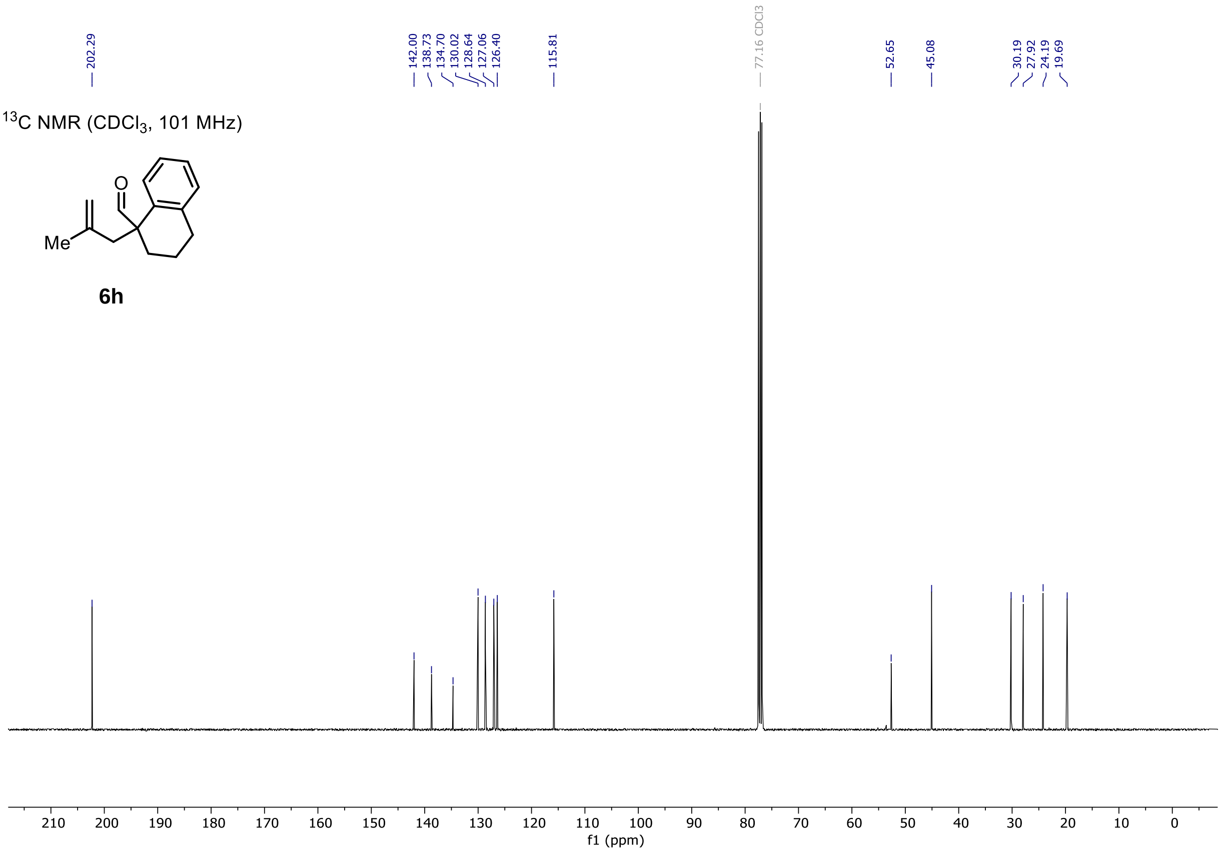

6h

^{1}H NMR ($CDCl_3$, 400 MHz)

6i

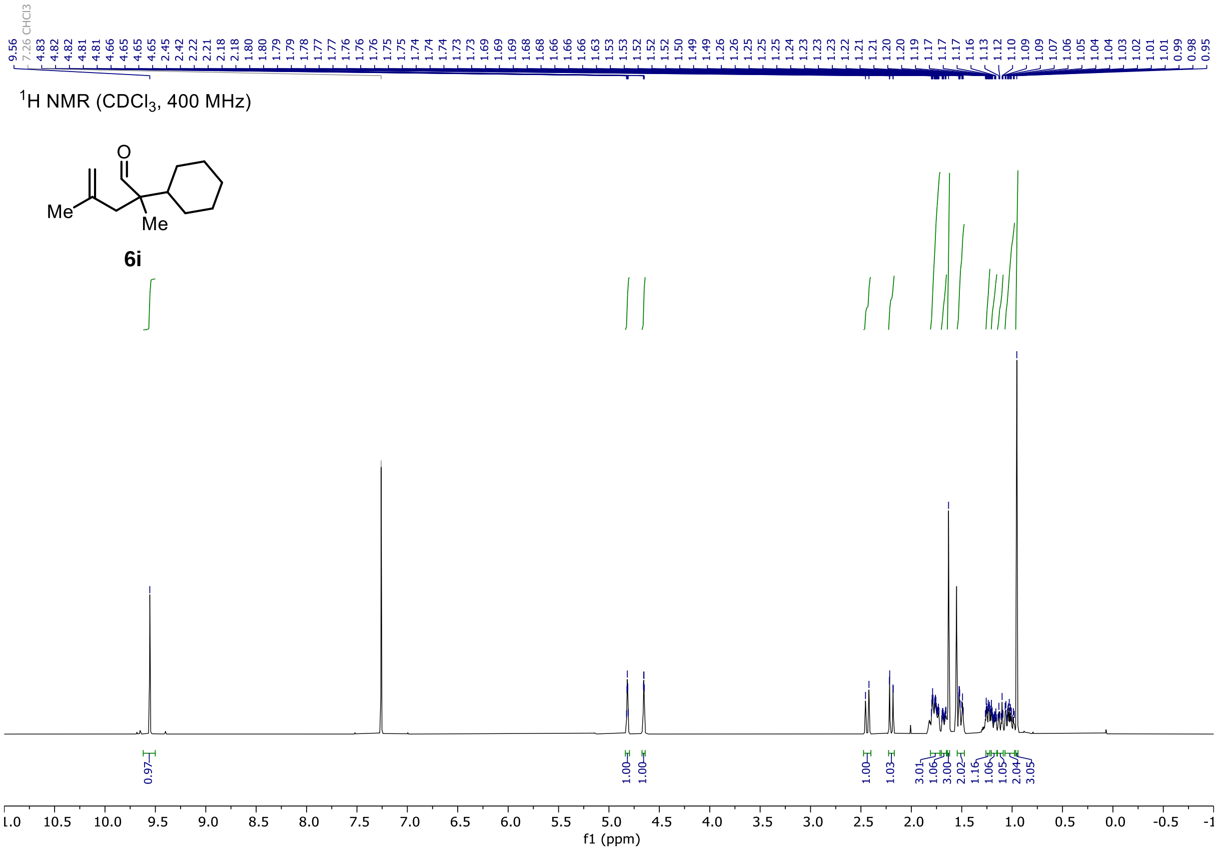

^{13}C NMR ($CDCl_3$, 101 MHz)

6i

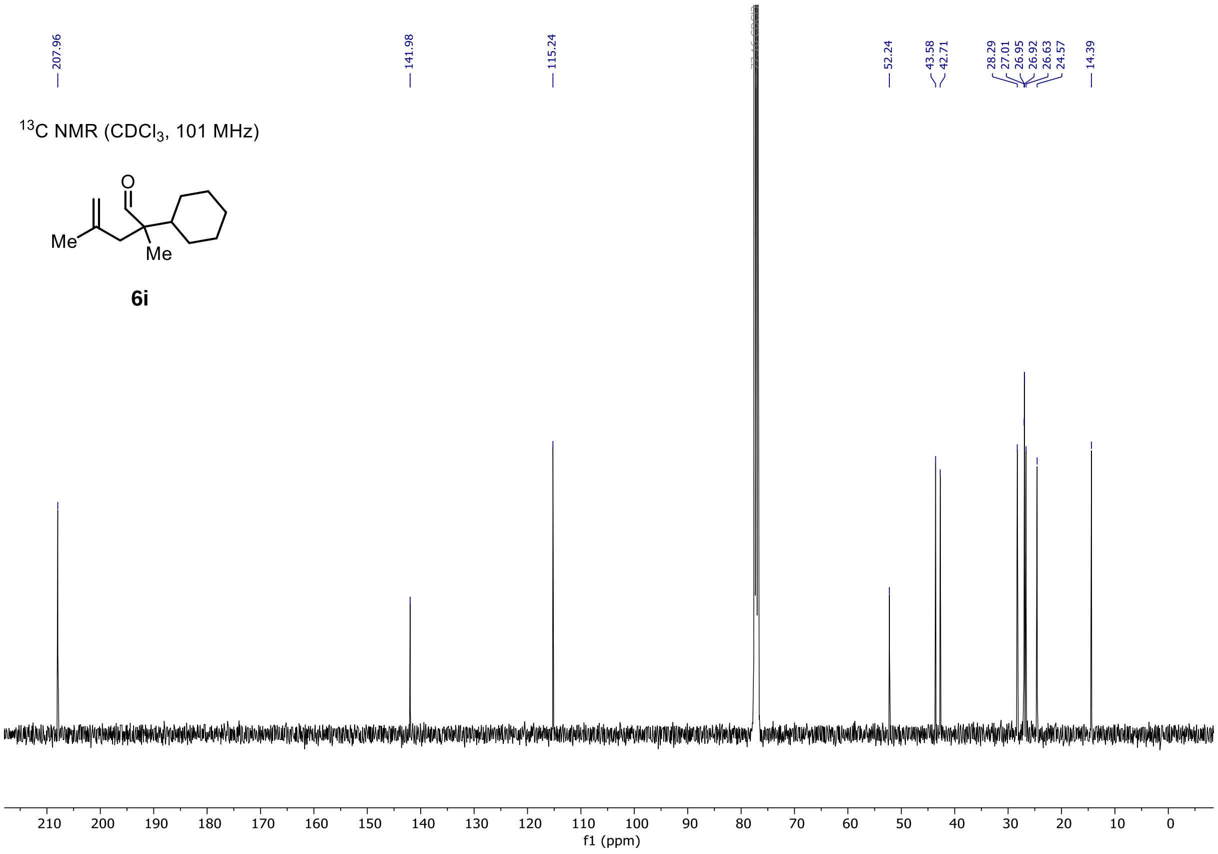

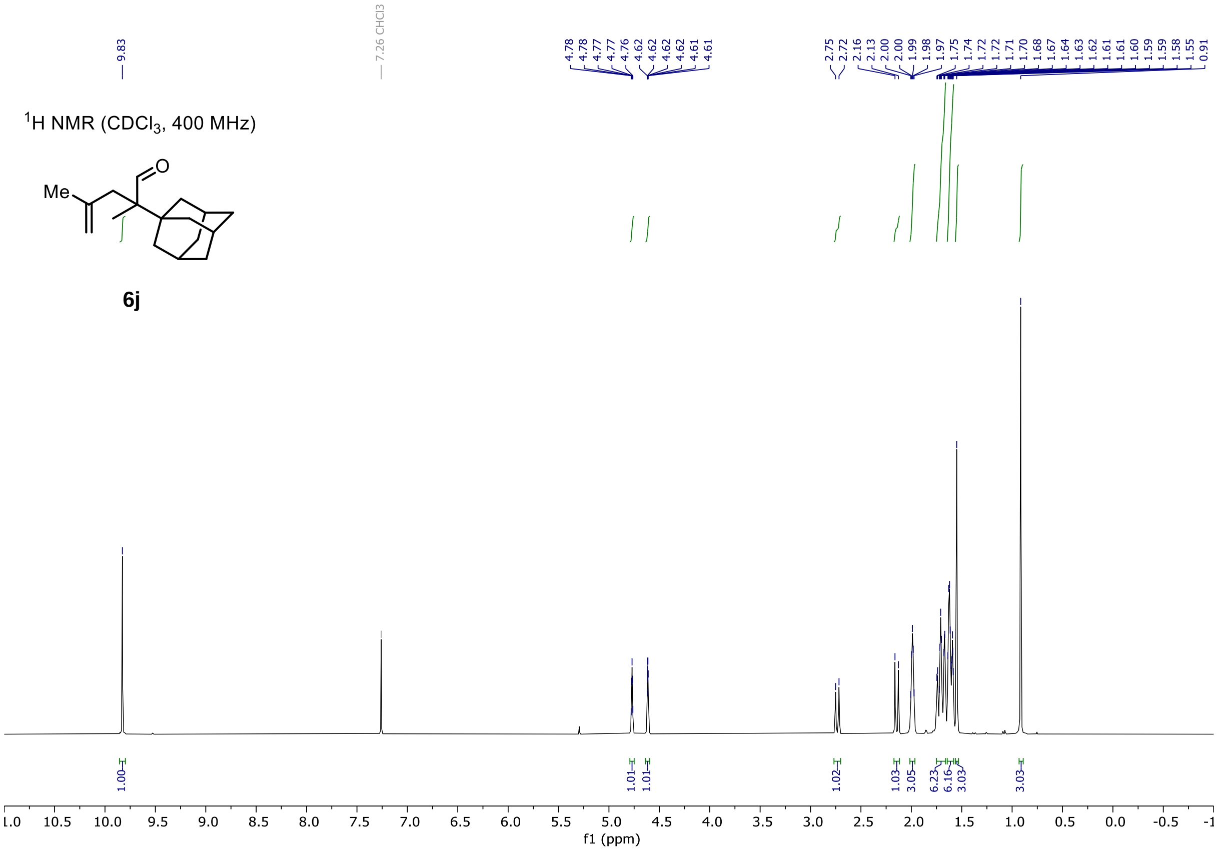

9.83
7.26 CHCl3
4.78 4.78 4.77 4.77 4.76 4.62 4.62 4.62 4.62 4.61 4.61
2.75 2.72 2.16 2.13 2.00 2.00 1.99 1.98 1.97 1.75 1.74 1.72 1.72 1.71 1.70 1.68 1.67 1.64 1.63 1.62 1.61 1.61 1.60 1.59 1.59 1.58 1.55 0.91
^{1}H NMR (CDCl$_3$, 400 MHz)
Me
O
6j
1.00
1.01
1.01
1.02
1.03
3.05
6.23
6.16
3.03
3.03
f1 (ppm)

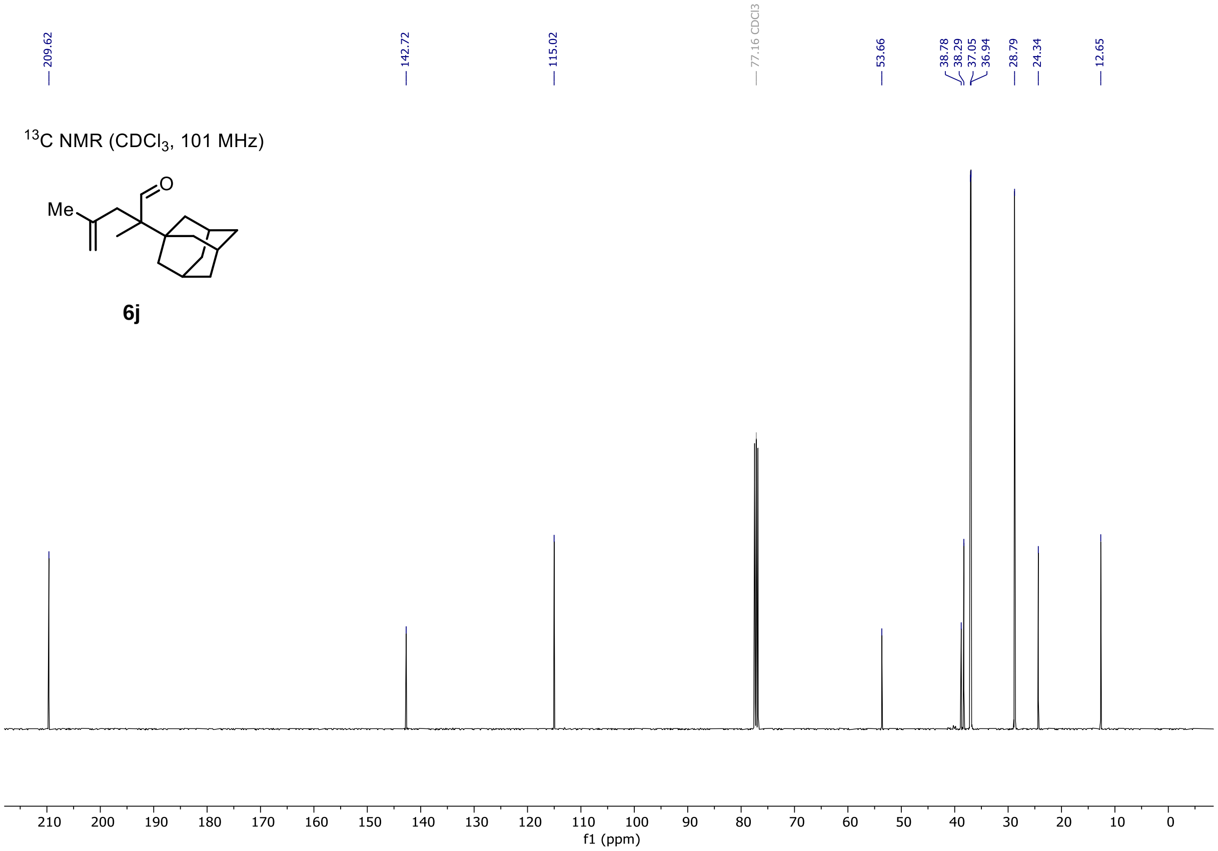

209.62
142.72
115.02
77.16 CDCl3
53.66
38.78 38.29 37.05 36.94
28.79
24.34
12.65
^{13}C NMR (CDCl$_3$, 101 MHz)
Me
O
6j
f1 (ppm)

^{1}H NMR ($CDCl_3$, 400 MHz)

6k

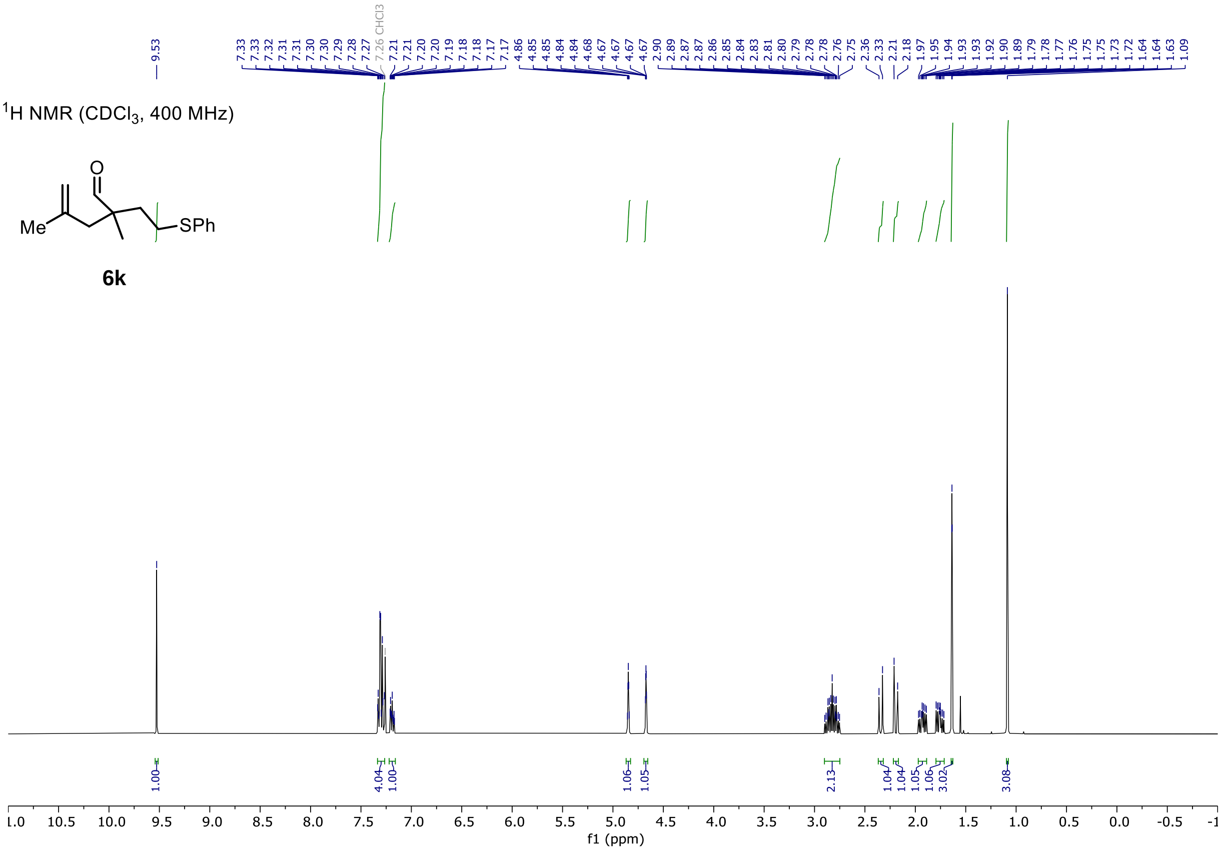

^{13}C NMR ($CDCl_3$, 101 MHz)

6k

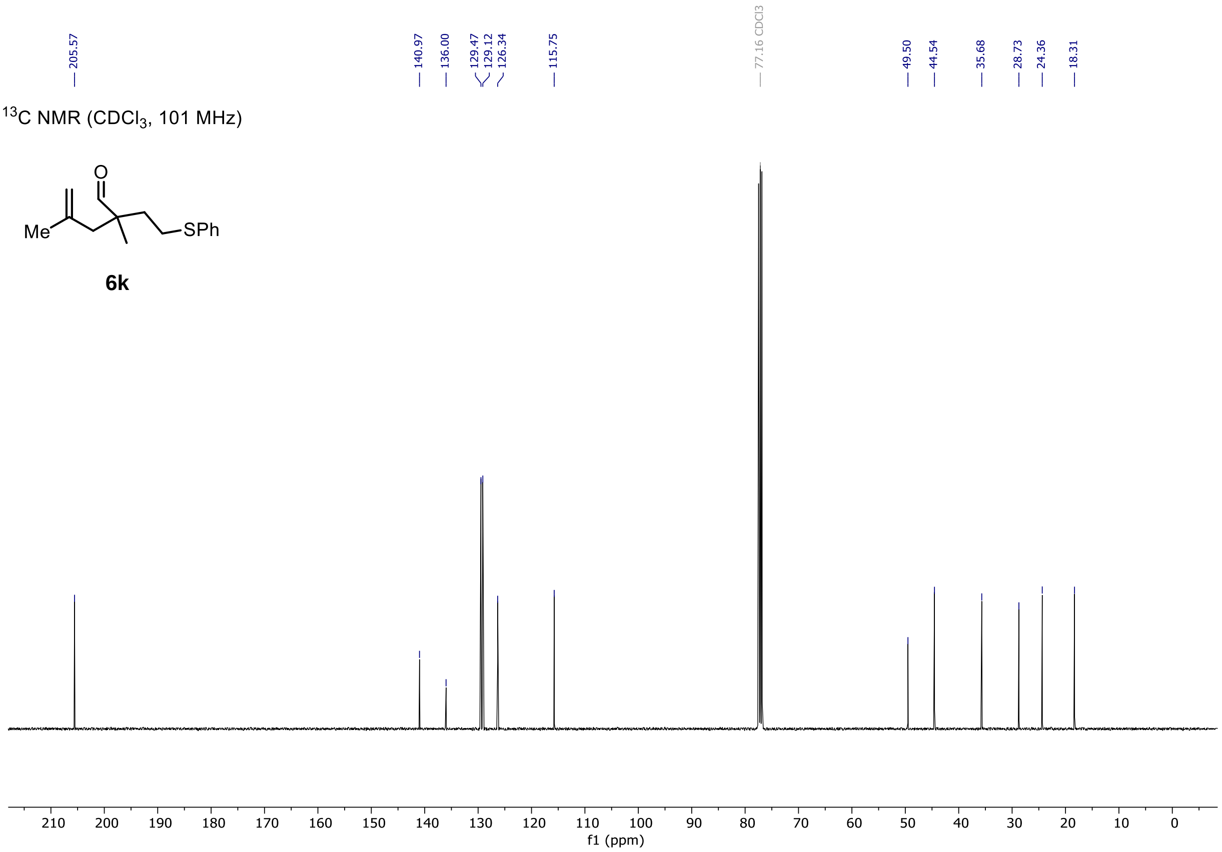

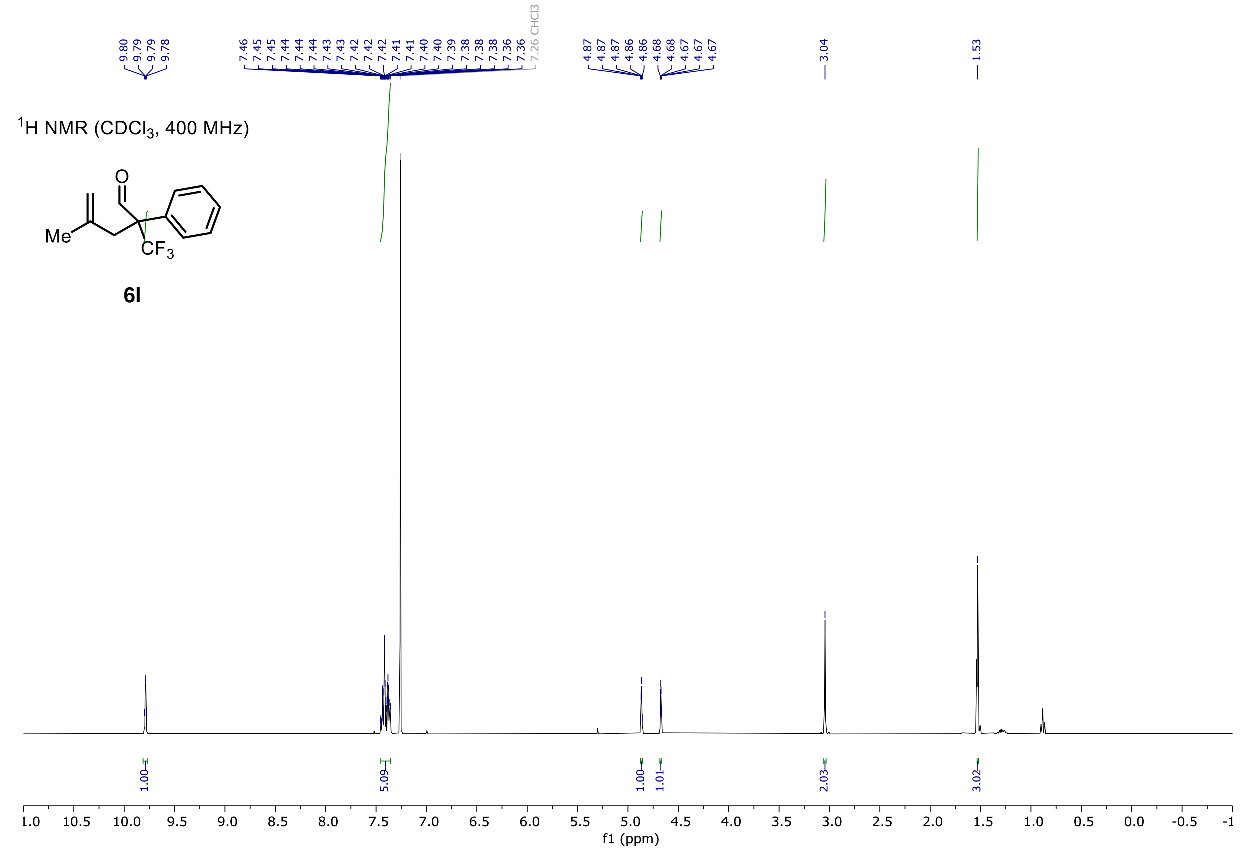
1H NMR (CDCl3, 400 MHz)
Me
O
CF3
6l
9.80 9.79 9.79 9.78
7.46 7.45 7.45 7.44 7.44 7.44 7.43 7.43 7.42 7.42 7.42 7.41 7.41 7.40 7.40 7.39 7.38 7.38 7.38 7.36 7.36
7.26 CHCl3
4.87 4.87 4.87 4.86 4.68 4.68 4.67 4.67 4.67
3.04
1.53
1.00
5.09
1.00
1.01
2.03
3.02
f1 (ppm)

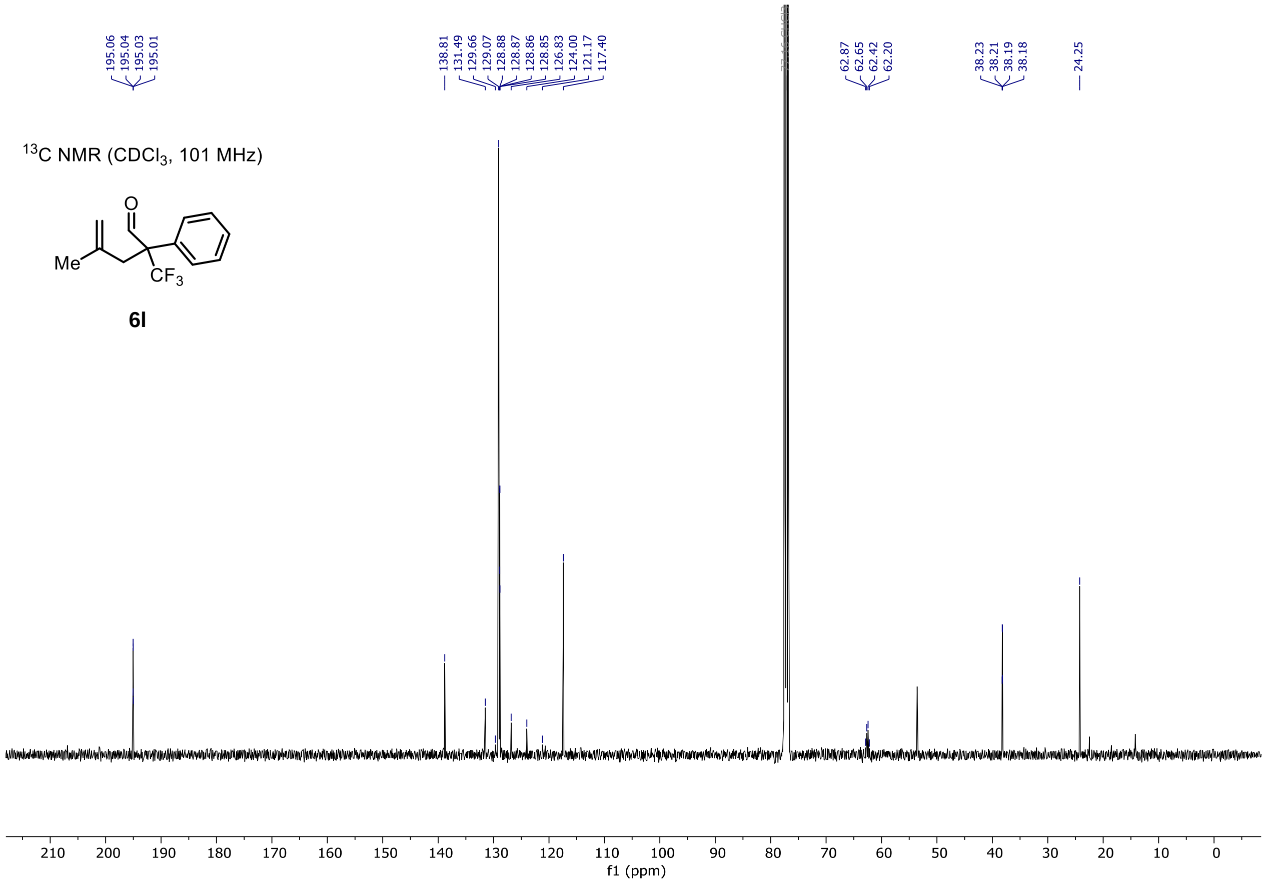
13C NMR (CDCl3, 101 MHz)
Me
O
CF3
6l
195.06 195.04 195.03 195.01
138.81 131.49 129.66 129.07 128.88 128.87 128.86 128.85 126.83 124.00 121.17 117.40
62.87 62.65 62.42 62.20
38.23 38.21 38.19 38.18
24.25
f1 (ppm)

^{19}F NMR ($CDCl_3$, 376 MHz)

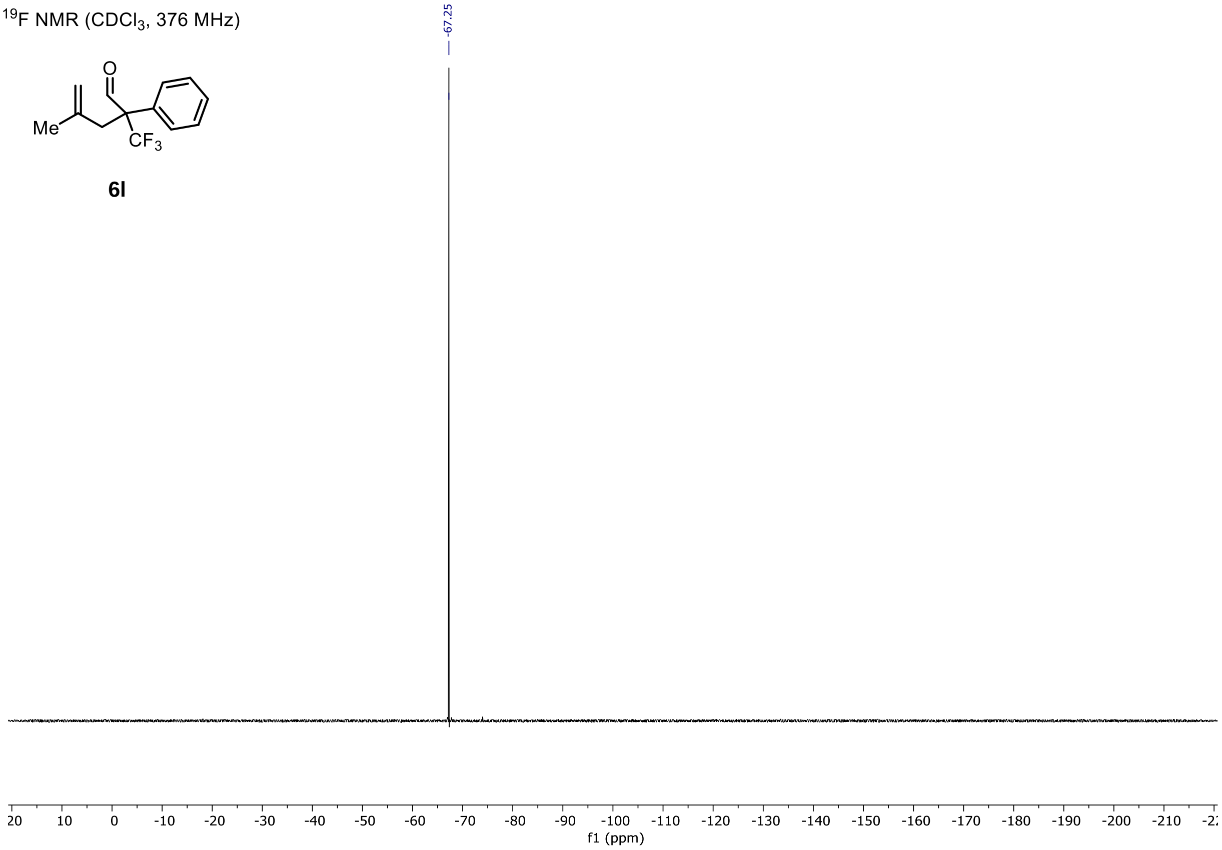

6l

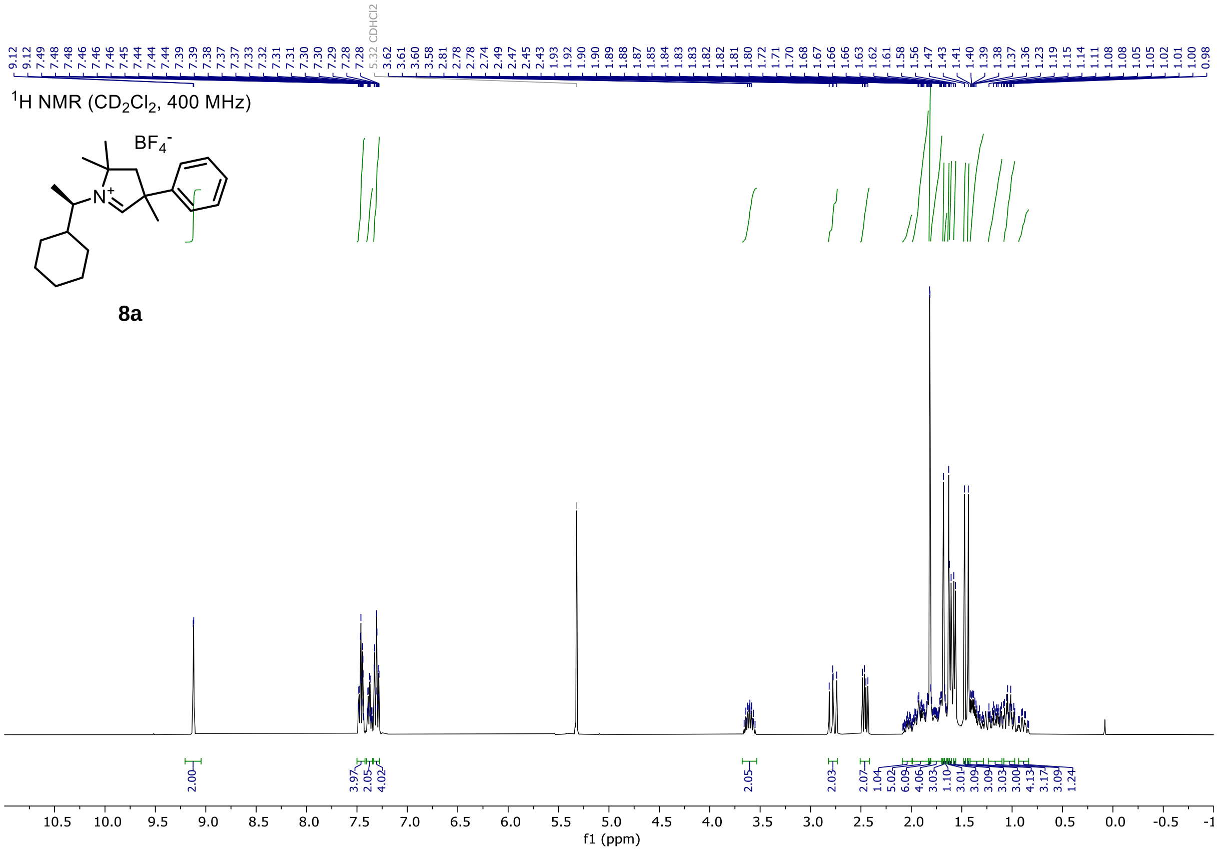

^{1}H NMR (CD_2Cl_2, 400 MHz)
BF4-
8a
f1 (ppm)

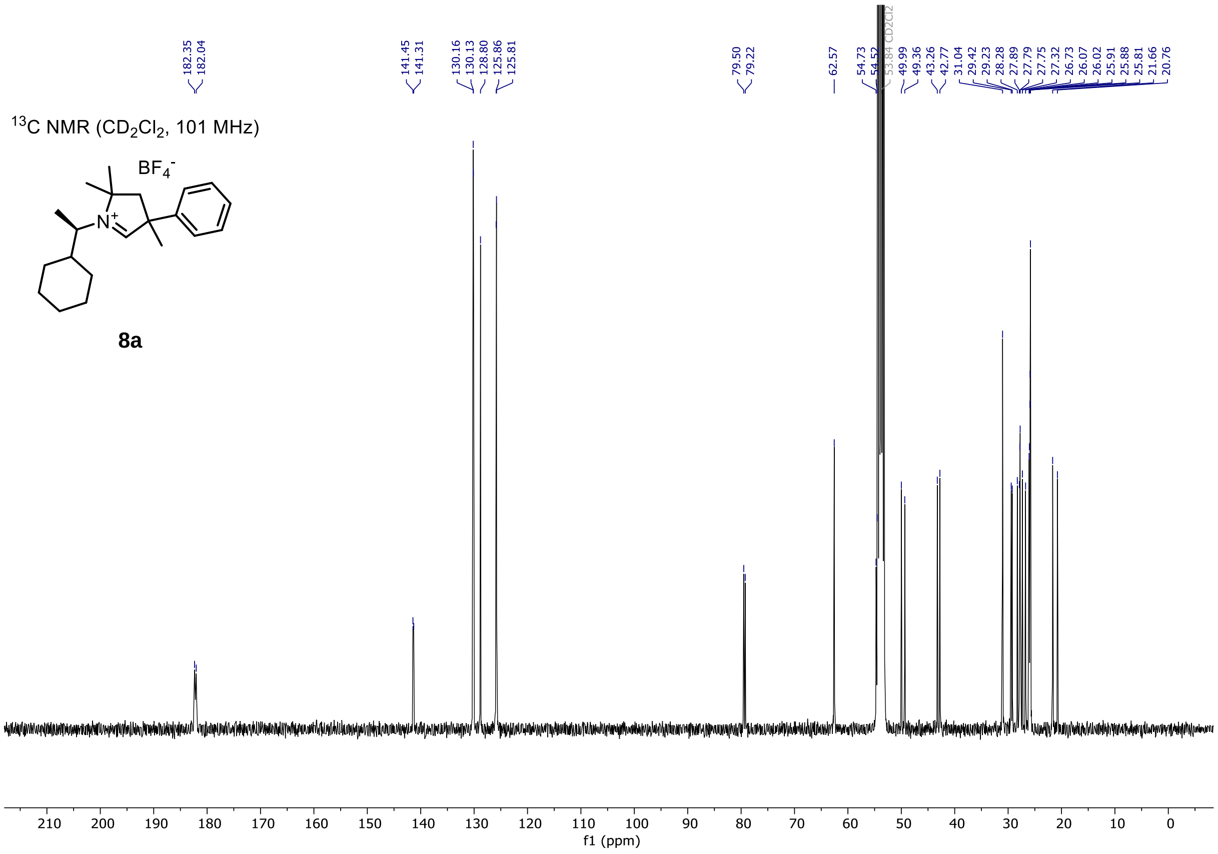

^{13}C NMR (CD_2Cl_2, 101 MHz)
BF4-
8a
f1 (ppm)

^{19}F NMR (CD_2Cl_2, 376 MHz)

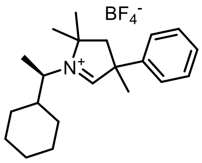

8a

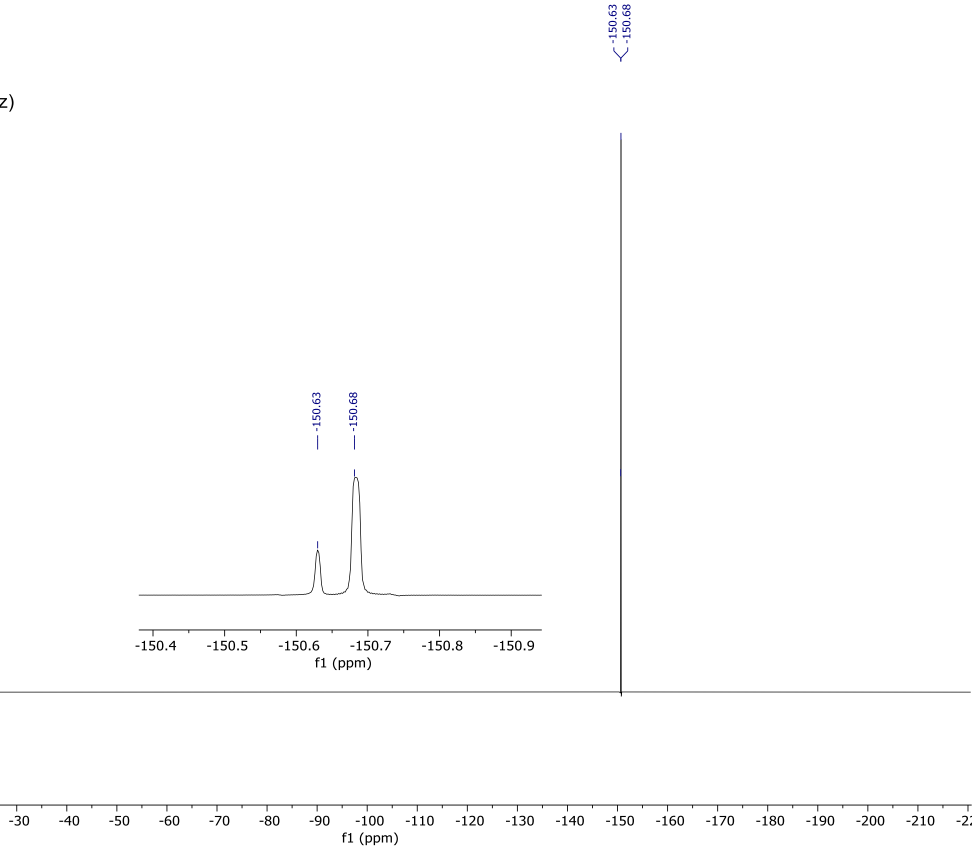

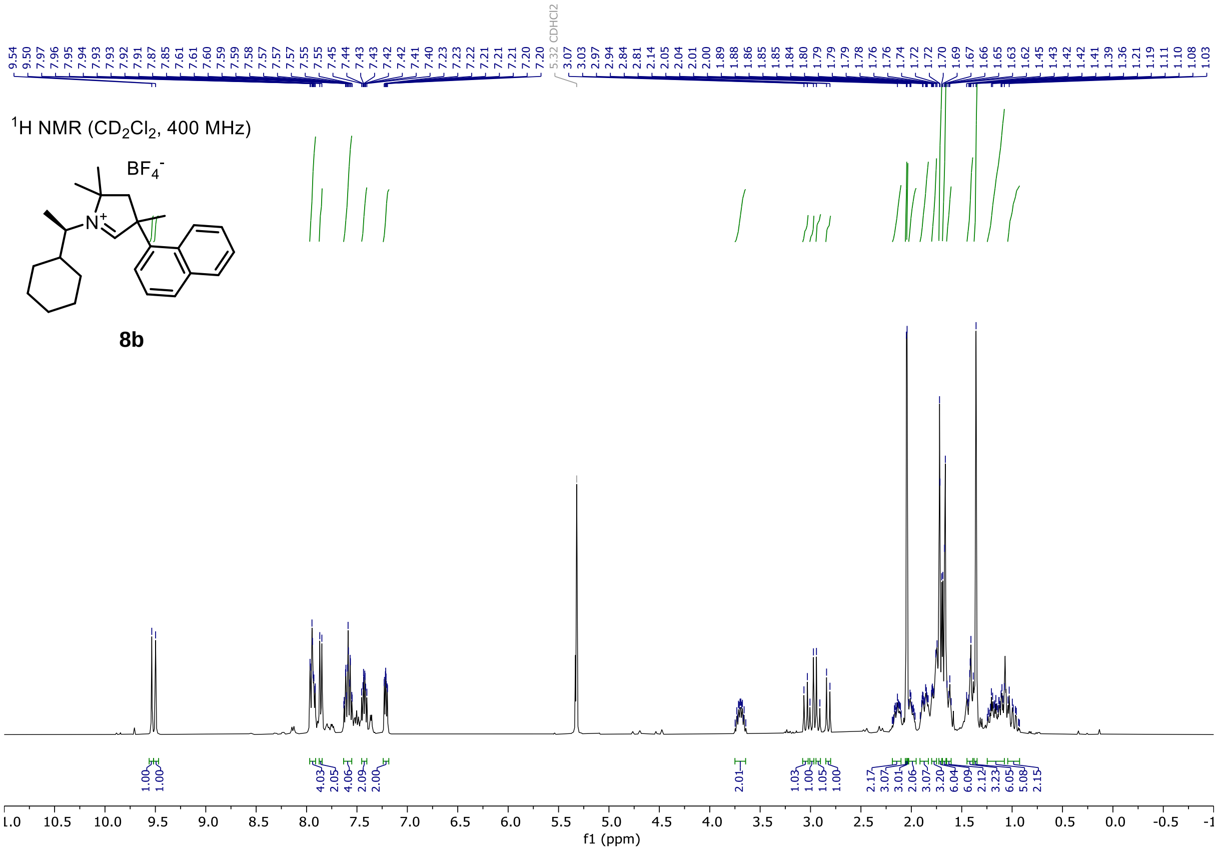
9.54 9.50 7.97 7.96 7.95 7.94 7.93 7.93 7.92 7.91 7.87 7.85 7.61 7.60 7.59 7.59 7.58 7.57 7.57 7.57 7.55 7.55 7.45 7.44 7.43 7.43 7.42 7.42 7.41 7.40 7.23 7.23 7.22 7.22 7.21 7.21 7.21 7.20
5.32 CDHCl2
3.07 3.03 2.97 2.94 2.84 2.81 2.14 2.05 2.04 2.01 2.00 1.89 1.88 1.86 1.85 1.85 1.84 1.80 1.79 1.79 1.79 1.78 1.76 1.76 1.74 1.72 1.72 1.70 1.69 1.67 1.66 1.65 1.63 1.62 1.45 1.43 1.43 1.42 1.41 1.39 1.36 1.21 1.19 1.11 1.10 1.08 1.03
1H NMR (CD2Cl2, 400 MHz)
BF4-
N+
8b
1.00 1.00 4.03 2.05 4.06 2.09 2.00 2.01 1.03 1.00 1.05 1.00 2.17 3.07 3.01 2.06 3.07 3.20 6.04 6.09 2.12 3.23 6.05 5.08 2.15
f1 (ppm)

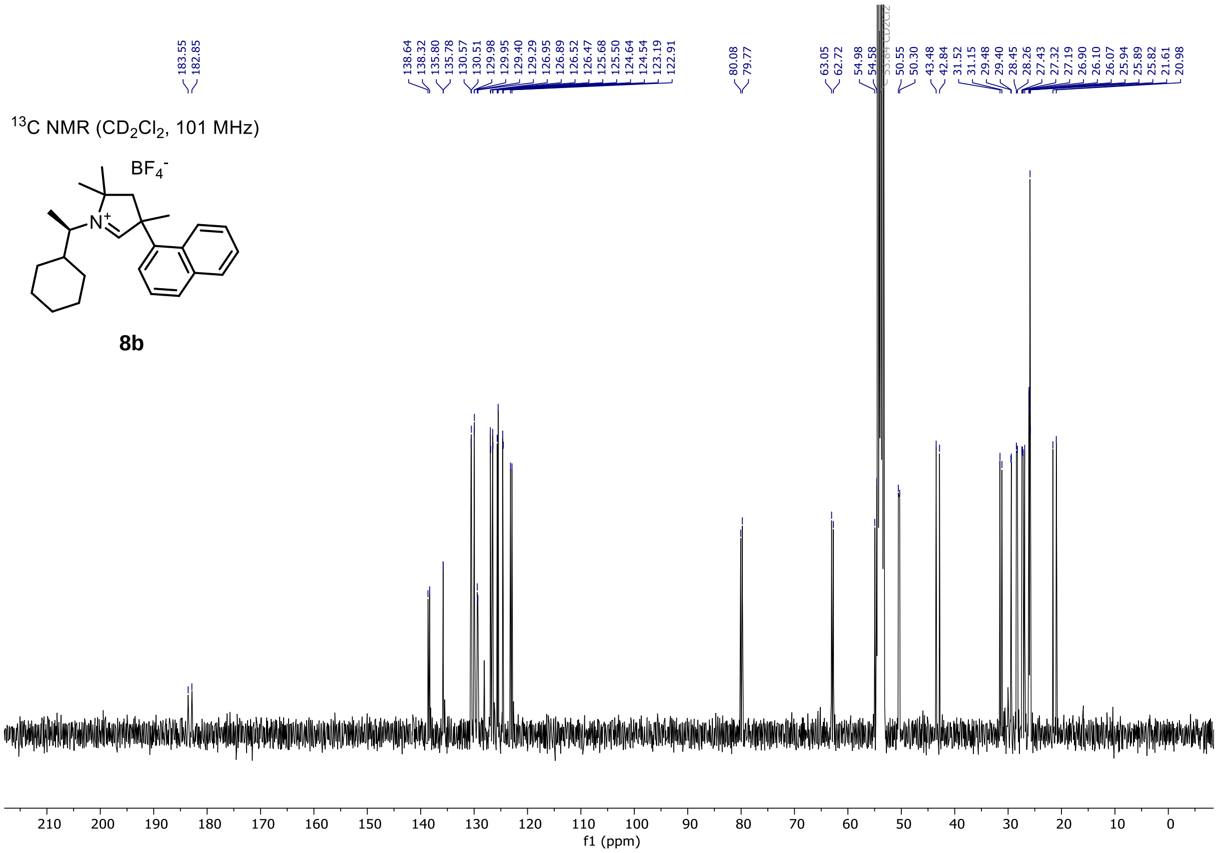
183.55 182.85 138.64 138.32 135.80 135.78 130.57 130.51 129.98 129.95 129.40 129.29 126.95 126.89 126.52 126.47 125.68 125.50 124.64 124.54 123.19 122.91 80.08 79.77 63.05 62.72 54.98 54.58 53.84 CD2Cl2 50.55 50.30 43.48 42.84 31.52 31.15 29.48 29.40 28.45 28.26 27.43 27.32 27.19 26.90 26.10 26.07 25.94 25.89 25.82 21.61 20.98
13C NMR (CD2Cl2, 101 MHz)
BF4-
N+
8b
f1 (ppm)

^{19}F NMR (CD_2Cl_2, 376 MHz)

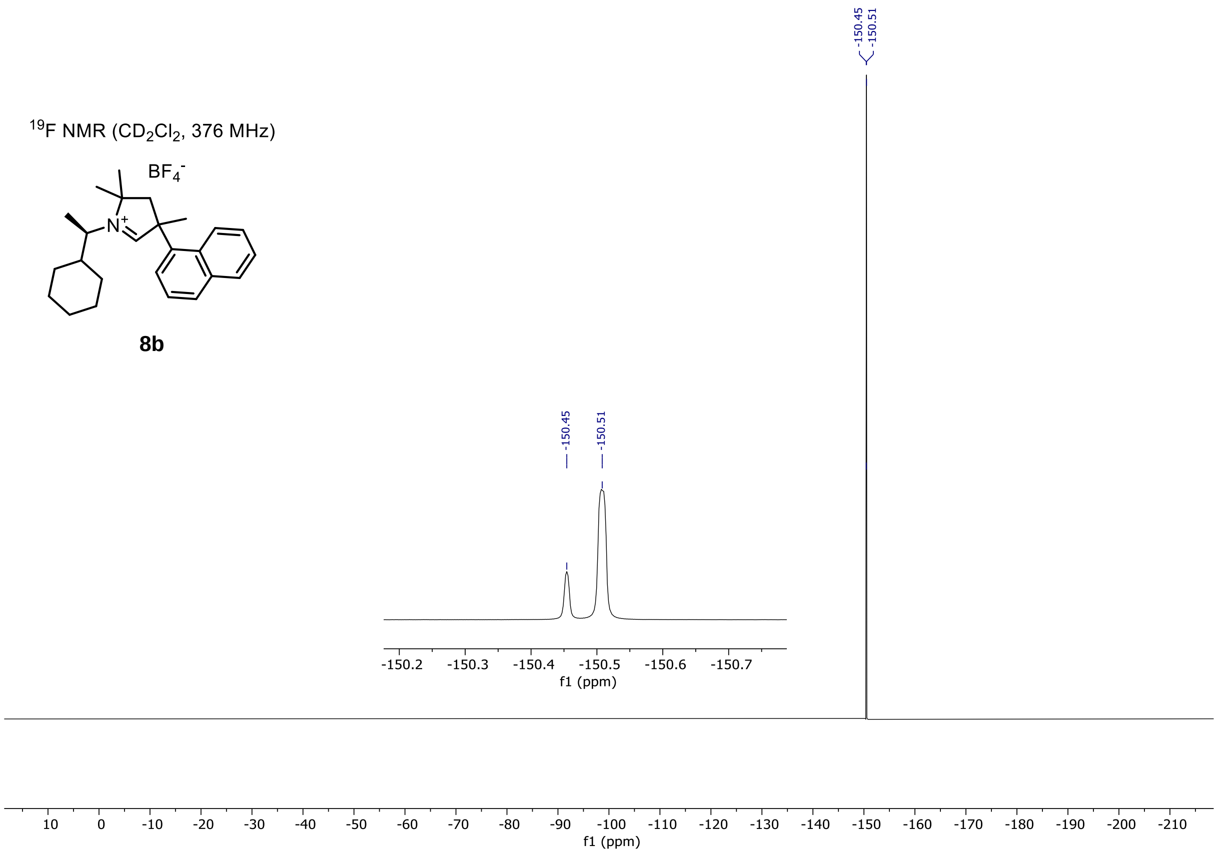

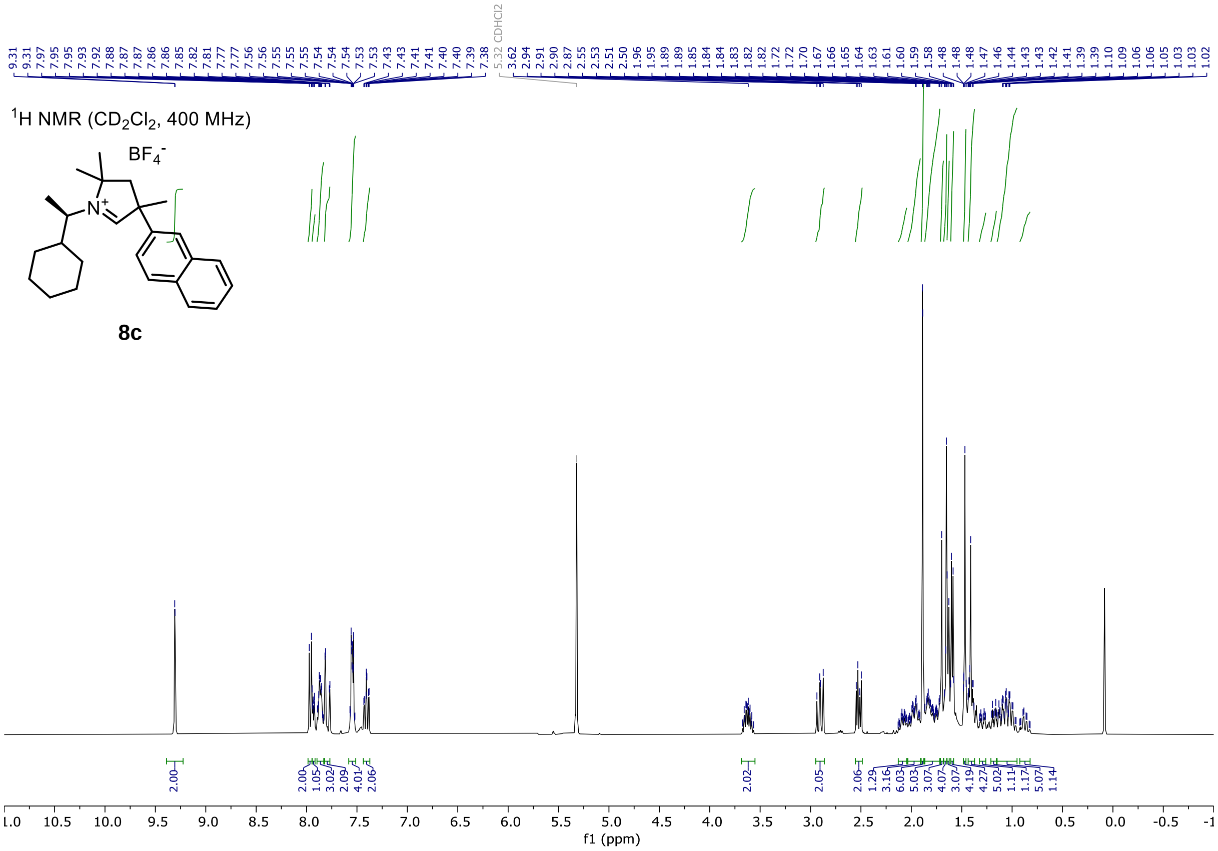
1H NMR (CD2Cl2, 400 MHz)
BF4-
8c
f1 (ppm)

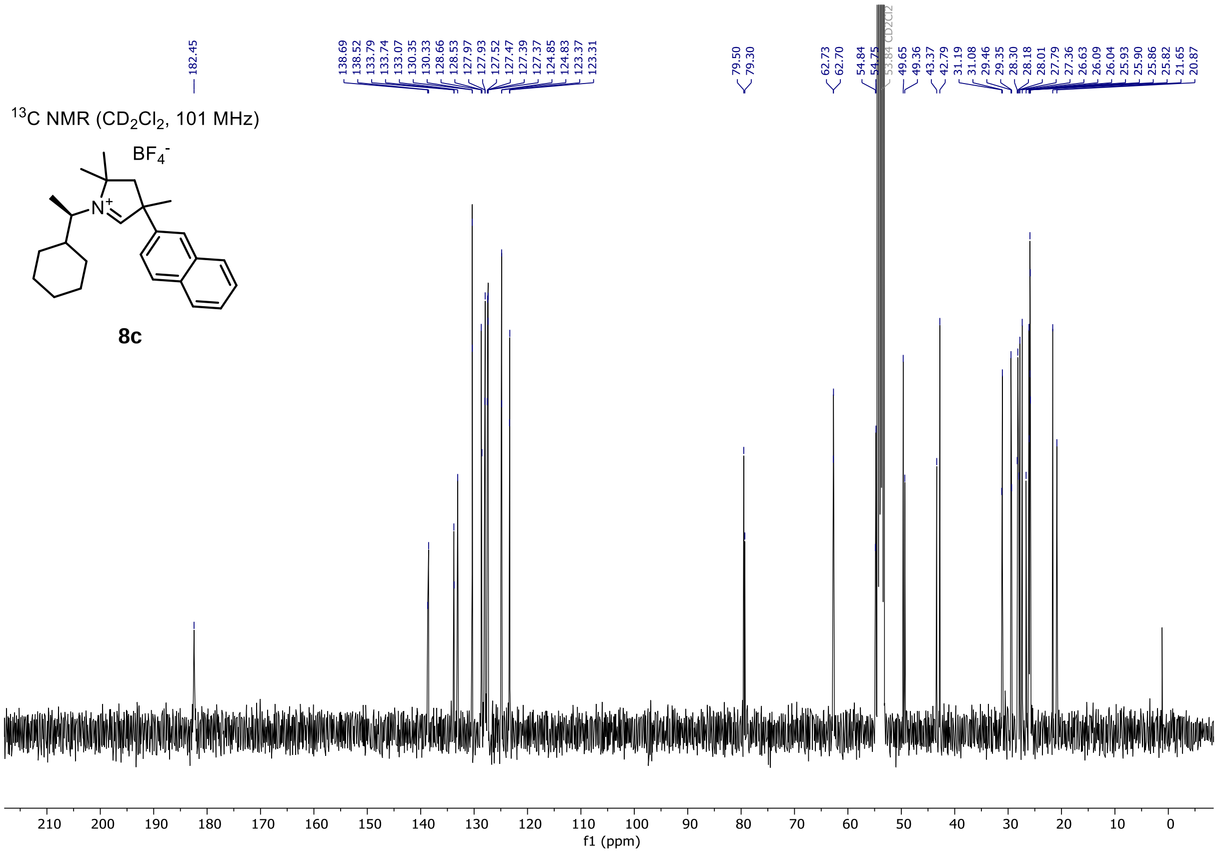
13C NMR (CD2Cl2, 101 MHz)
BF4-
8c
f1 (ppm)

^{19}F NMR (CD_2Cl_2, 376 MHz)

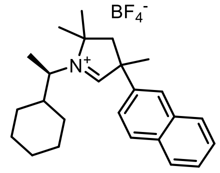

8c

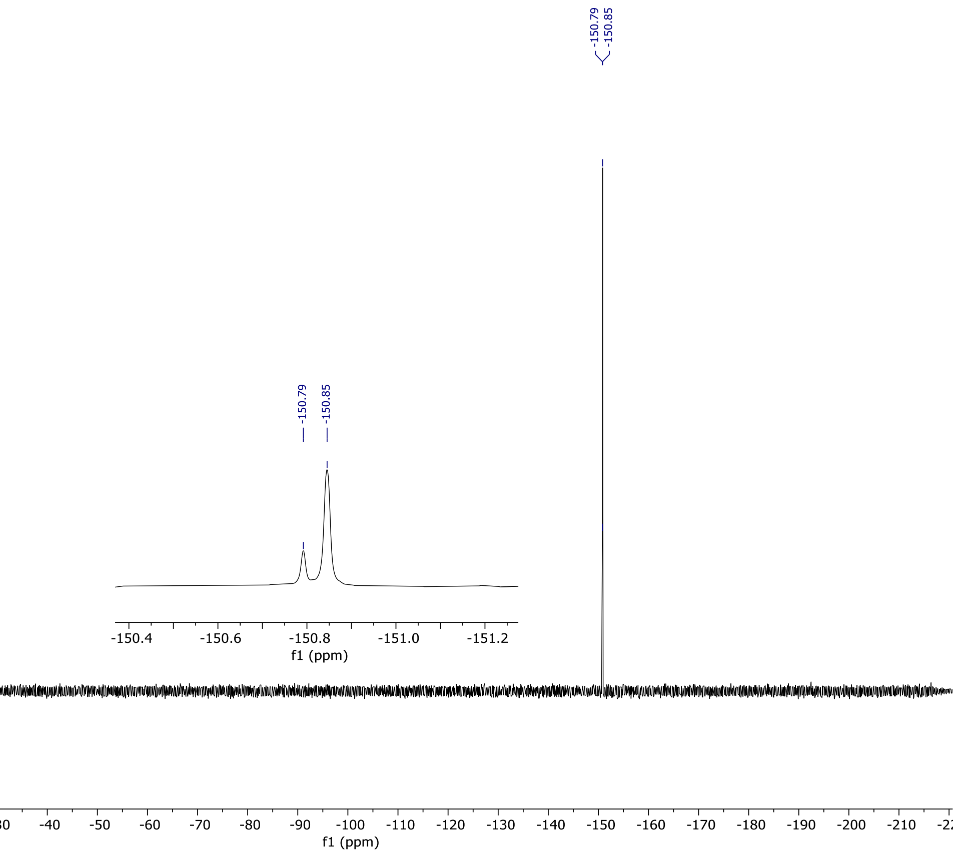

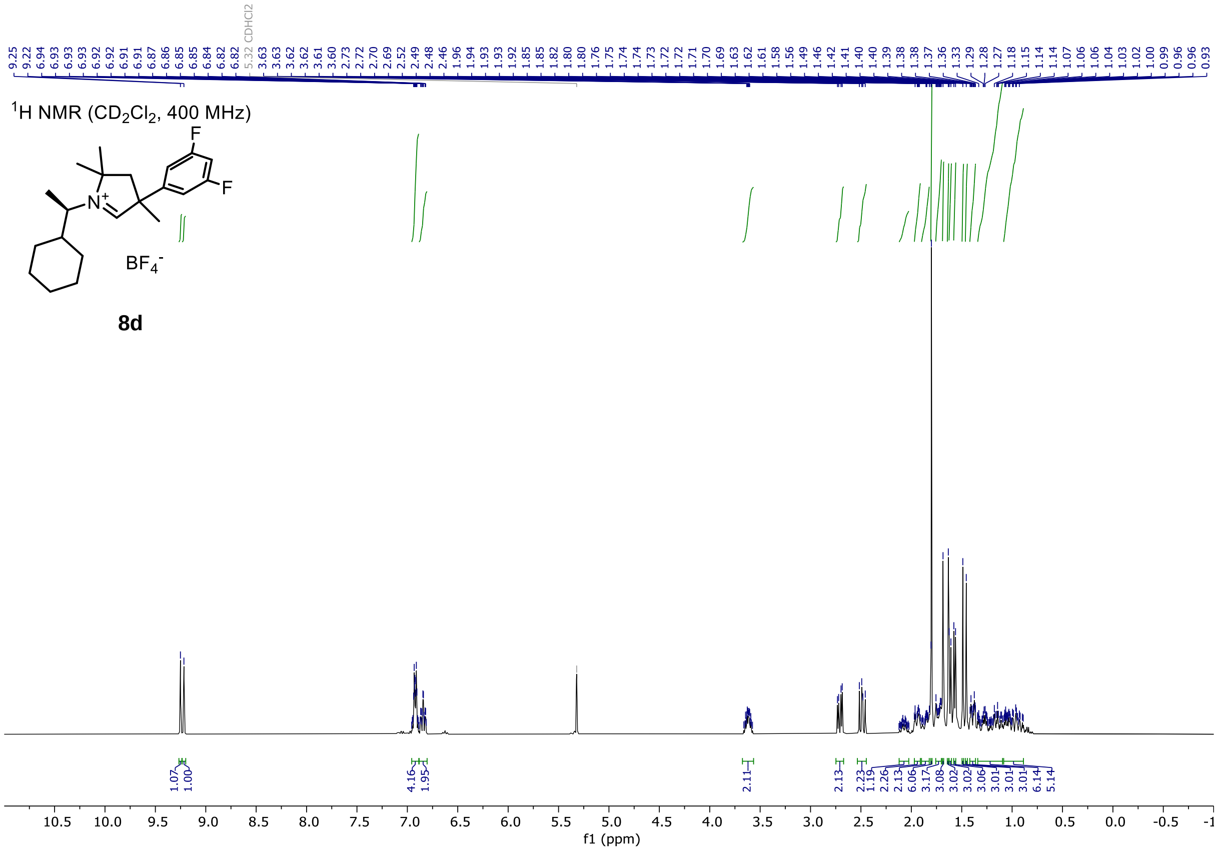

1H NMR (CD2Cl2, 400 MHz)
F
F
N+
BF4-
8d
f1 (ppm)

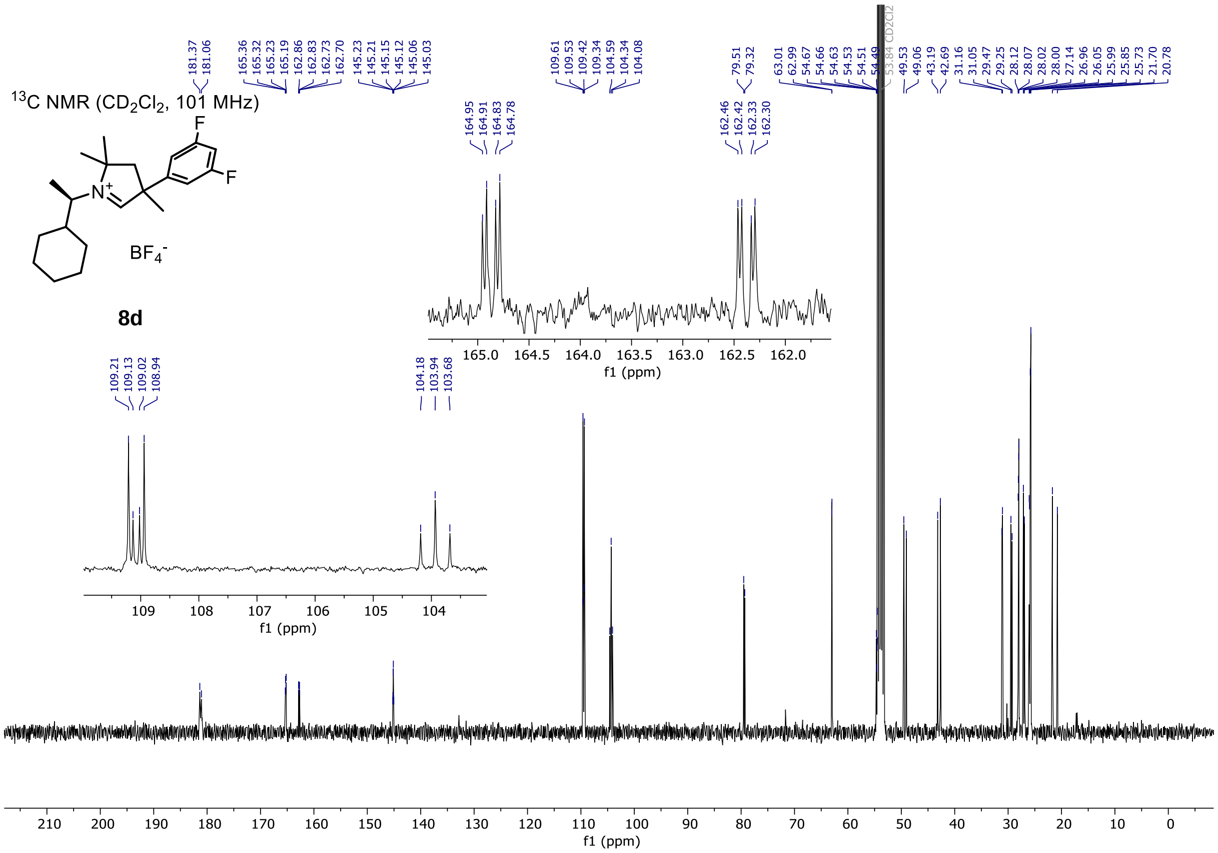

13C NMR (CD2Cl2, 101 MHz)
F
F
N+
BF4-
8d
f1 (ppm)

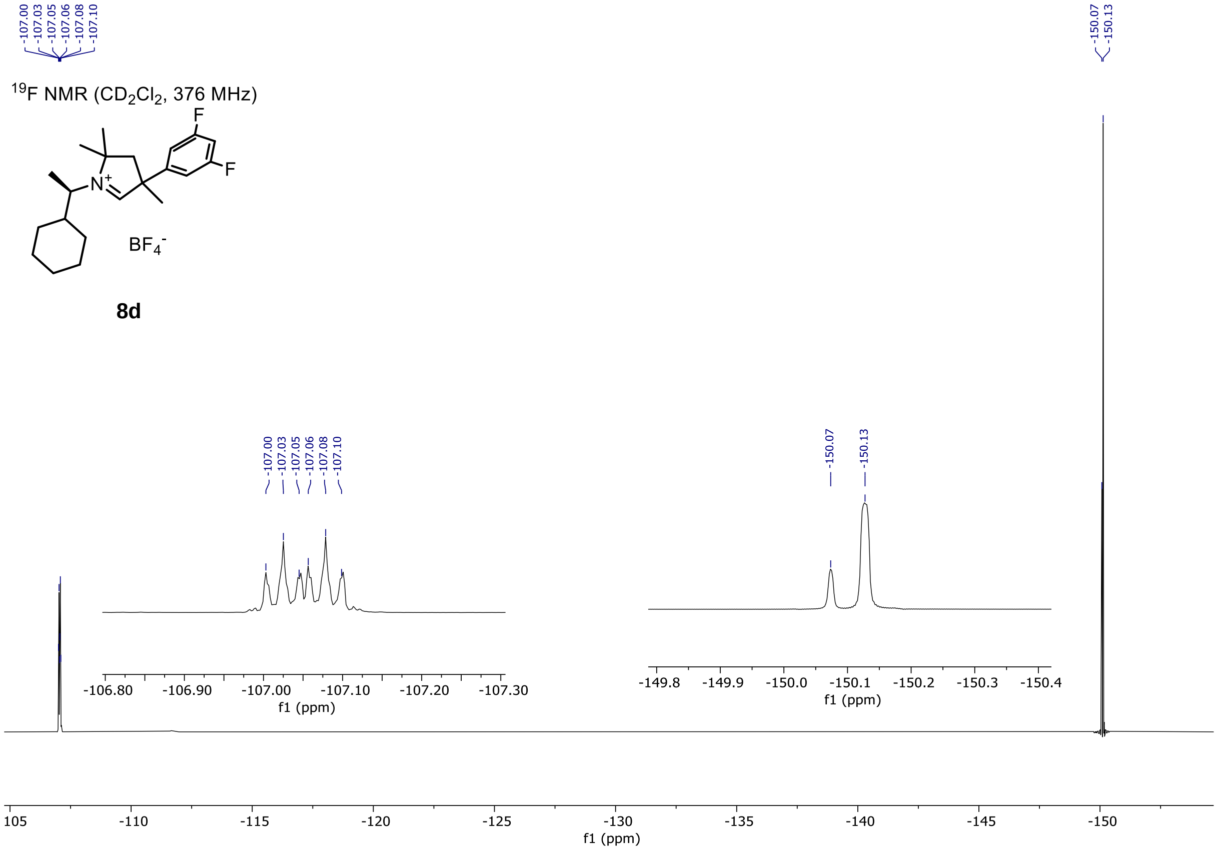

-107.00
-107.03
-107.05
-107.06
-107.08
-107.10
-150.07
-150.13
^{19}F NMR (CD_2Cl_2, 376 MHz)
F
F
N+
BF_4^-
8d
-106.80
-106.90
-107.00
-107.10
-107.20
-107.30
f1 (ppm)
-149.8
-149.9
-150.0
-150.1
-150.2
-150.3
-150.4
f1 (ppm)
105
-110
-115
-120
-125
-130
-135
-140
-145
-150
f1 (ppm)

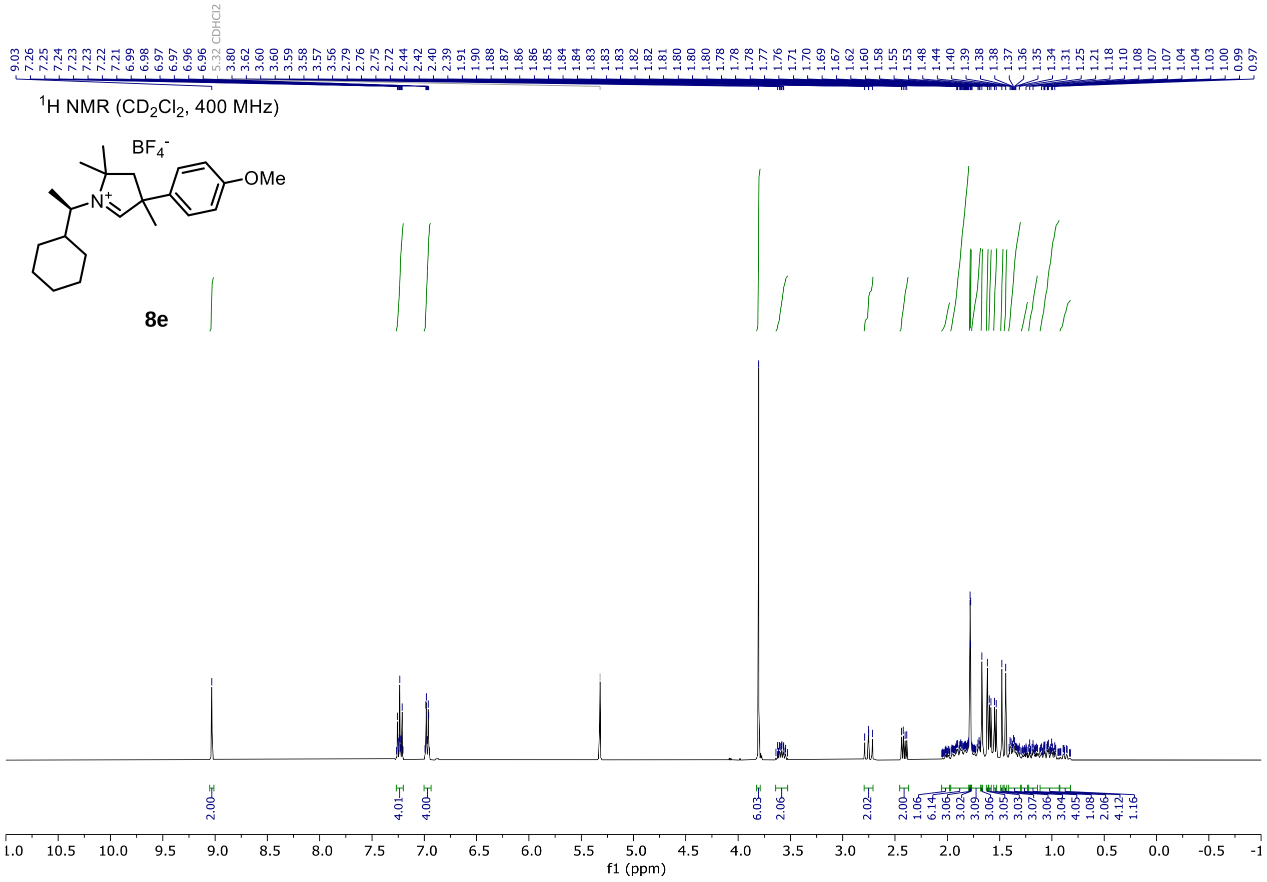

^{1}H NMR (CD_2Cl_2, 400 MHz)
BF_4^-
OMe
8e
f1 (ppm)

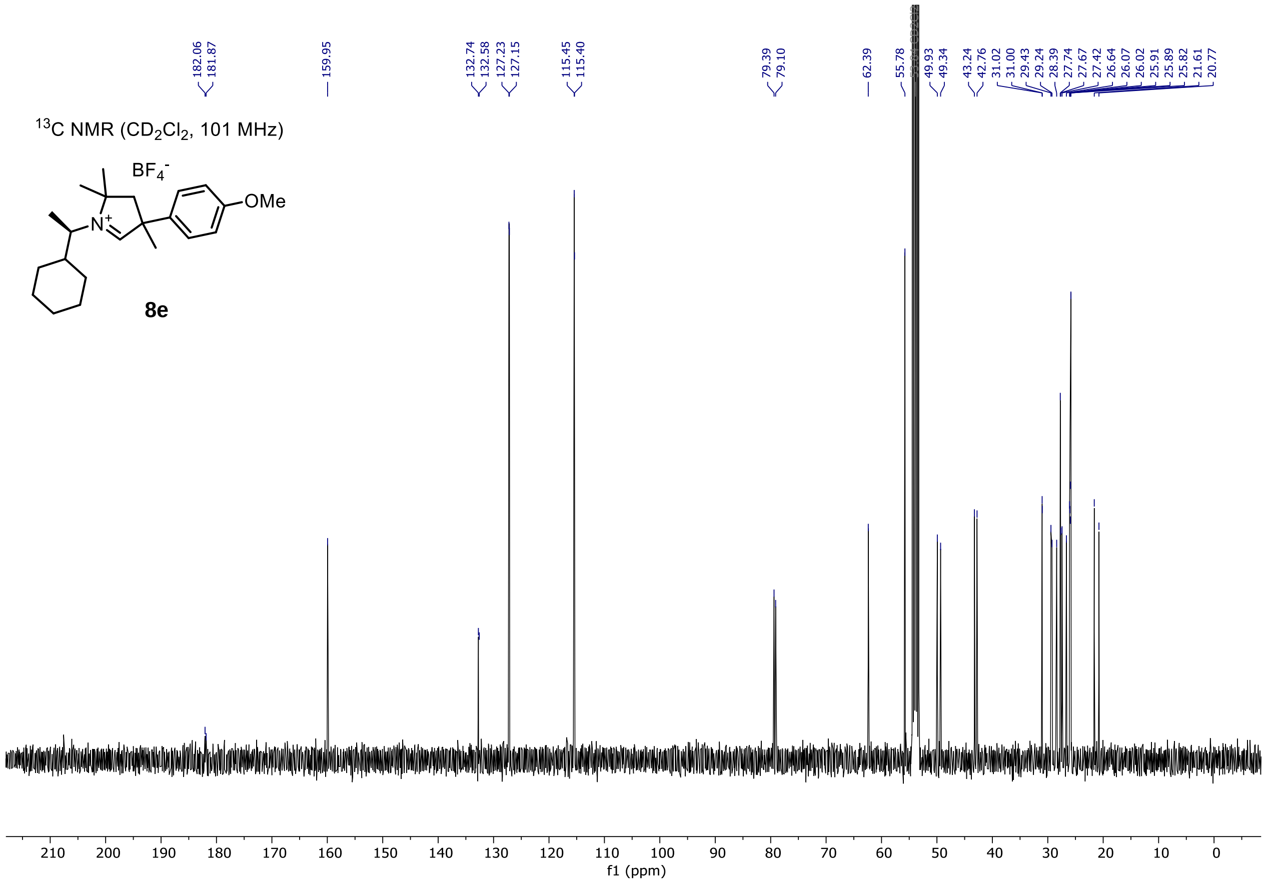

^{13}C NMR (CD_2Cl_2, 101 MHz)
BF_4^-
OMe
8e
182.06
181.87
159.95
132.74
132.58
127.23
127.15
115.45
115.40
79.39
79.10
62.39
55.78
49.93
49.34
43.24
42.76
31.02
31.00
29.43
29.24
28.39
27.74
27.67
27.42
26.64
26.07
26.02
25.91
25.89
25.82
21.61
20.77
f1 (ppm)

^{19}F NMR (CD_2Cl_2, 376 MHz)

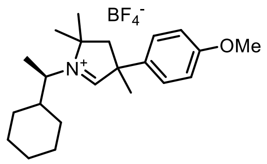

8e

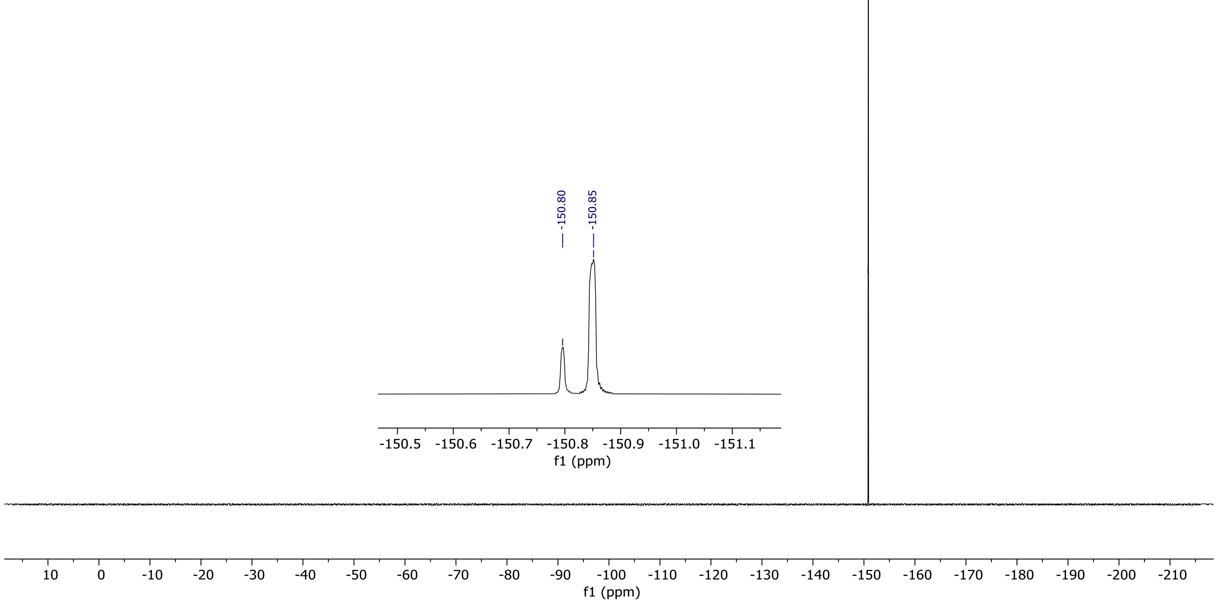

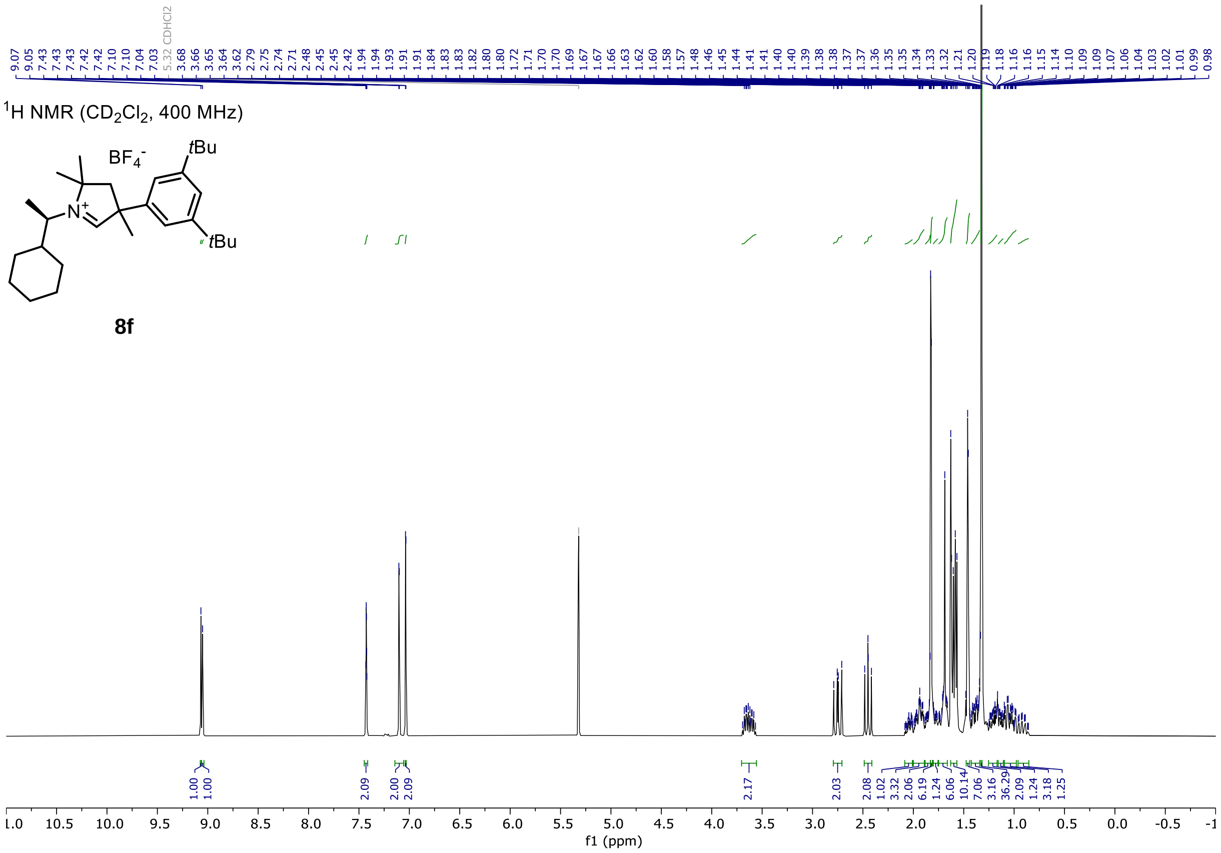
9.07 9.05 7.43 7.43 7.42 7.42 7.10 7.10 7.04 7.03 5.32 CDHCl2 3.68 3.66 3.65 3.64 3.62 2.79 2.75 2.74 2.71 2.48 2.45 2.45 2.42 1.94 1.94 1.93 1.91 1.91 1.84 1.83 1.83 1.82 1.80 1.72 1.71 1.70 1.69 1.67 1.67 1.66 1.63 1.62 1.60 1.58 1.57 1.48 1.46 1.45 1.44 1.41 1.41 1.40 1.39 1.38 1.38 1.37 1.37 1.36 1.35 1.35 1.34 1.33 1.32 1.21 1.20 1.19 1.18 1.16 1.16 1.15 1.14 1.10 1.09 1.07 1.06 1.04 1.03 1.02 1.01 0.99 0.98
1H NMR (CD2Cl2, 400 MHz)
BF4-
tBu
N+
tBu
1.00 1.00 2.09 2.00 2.09 2.17 2.03 2.08 1.02 3.32 2.06 6.19 1.24 6.06 10.14 7.06 3.16 36.29 2.09 1.24 3.18 1.25
f1 (ppm)

8f

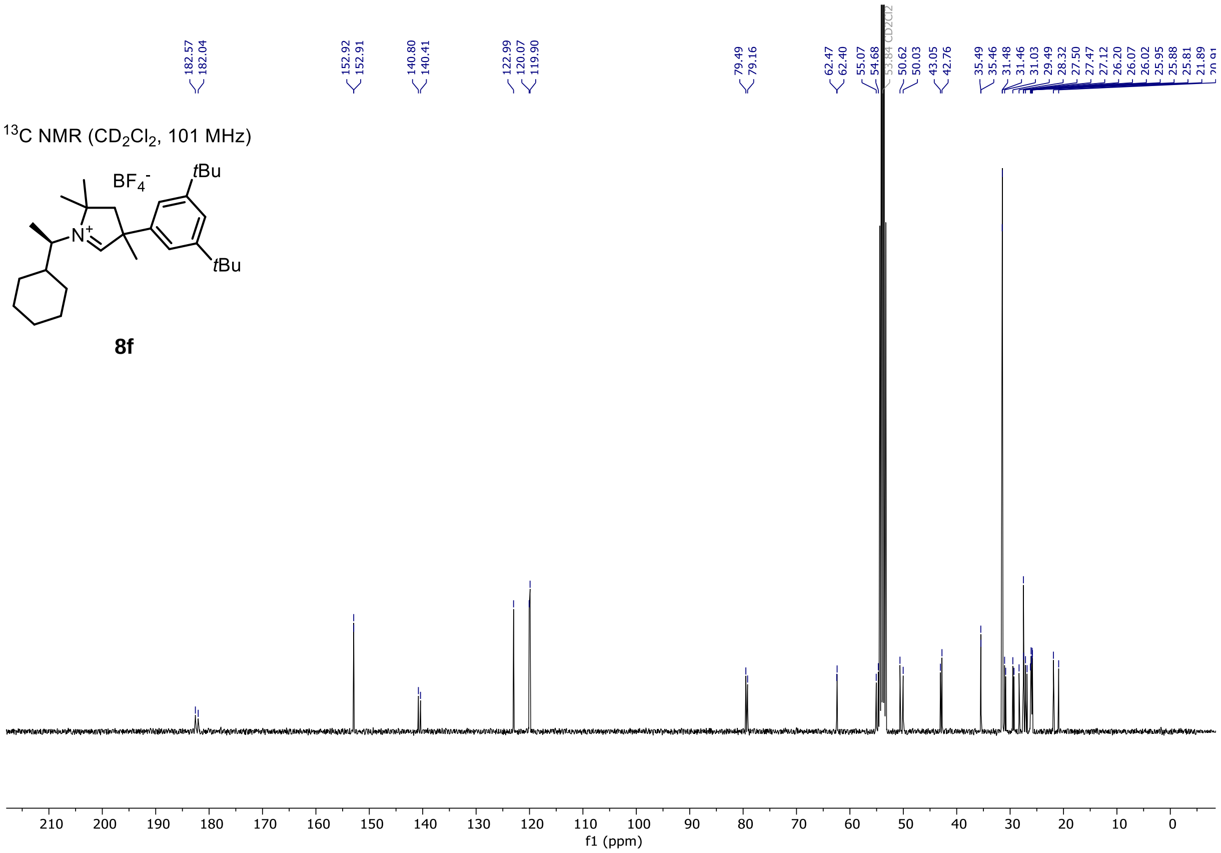
182.57 182.04 152.92 152.91 140.80 140.41 122.99 120.07 119.90 79.49 79.16 62.47 62.40 55.07 54.68 53.84 CD2Cl2 50.62 50.03 43.05 42.76 35.49 35.46 31.48 31.46 31.03 29.49 28.32 27.50 27.47 27.12 26.20 26.07 26.02 25.95 25.88 25.81 21.89
13C NMR (CD2Cl2, 101 MHz)
BF4-
tBu
N+
tBu
f1 (ppm)

8f

^{19}F NMR (CD_2Cl_2, 376 MHz)

8f

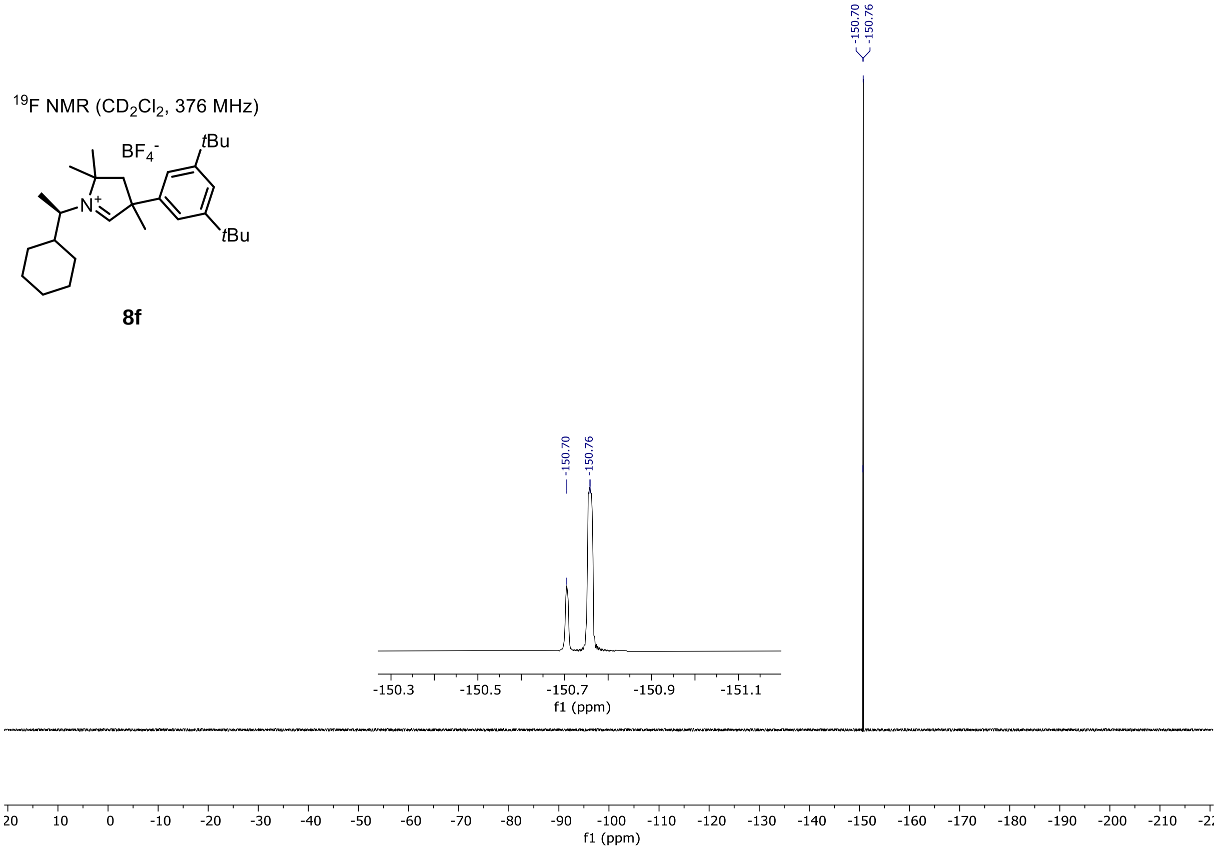

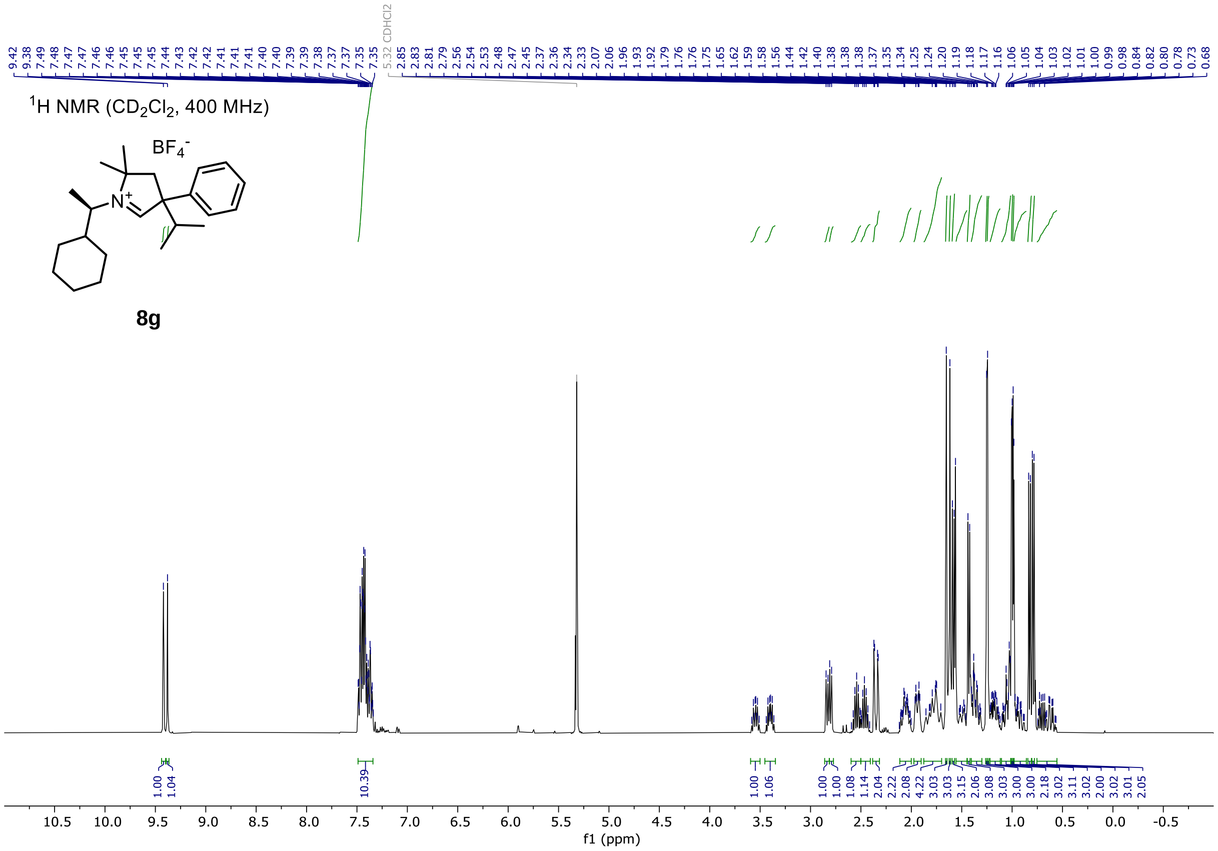

^{1}H NMR (CD_2Cl_2, 400 MHz)
BF_4^-
8g
f1 (ppm)

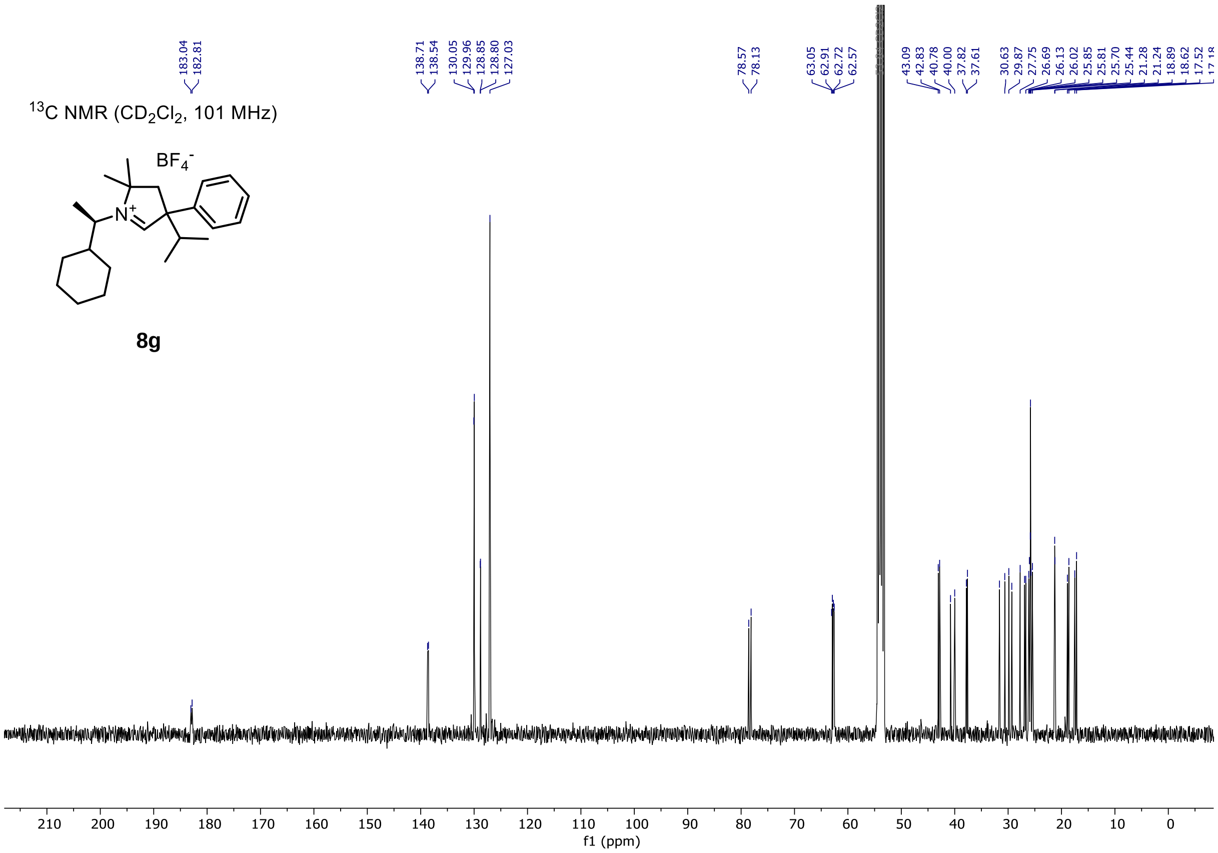

^{13}C NMR (CD_2Cl_2, 101 MHz)
BF_4^-
8g
f1 (ppm)

^{19}F NMR (CD_2Cl_2, 376 MHz)

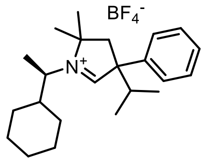

8g

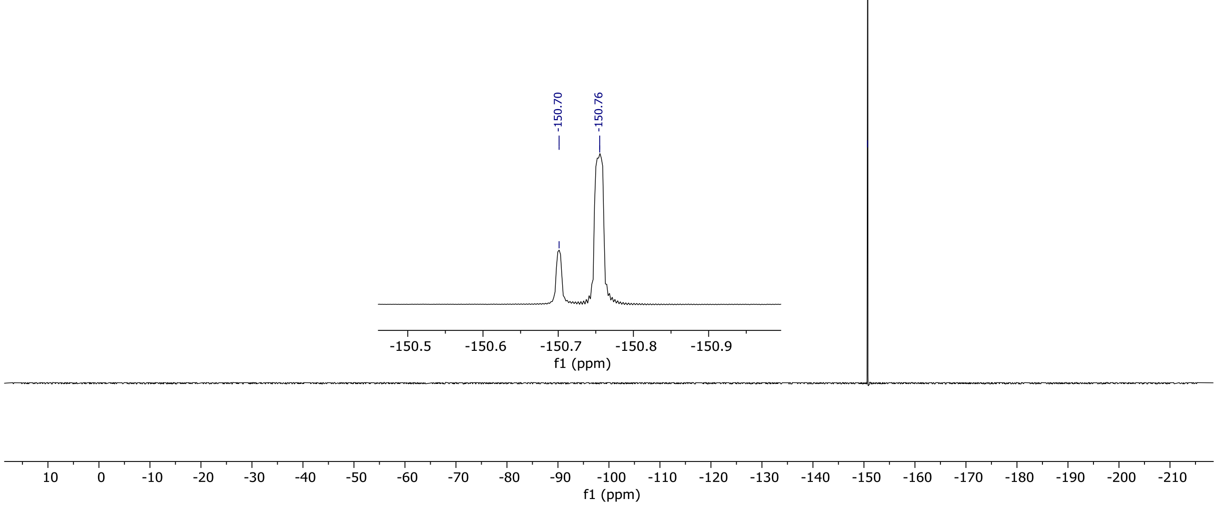

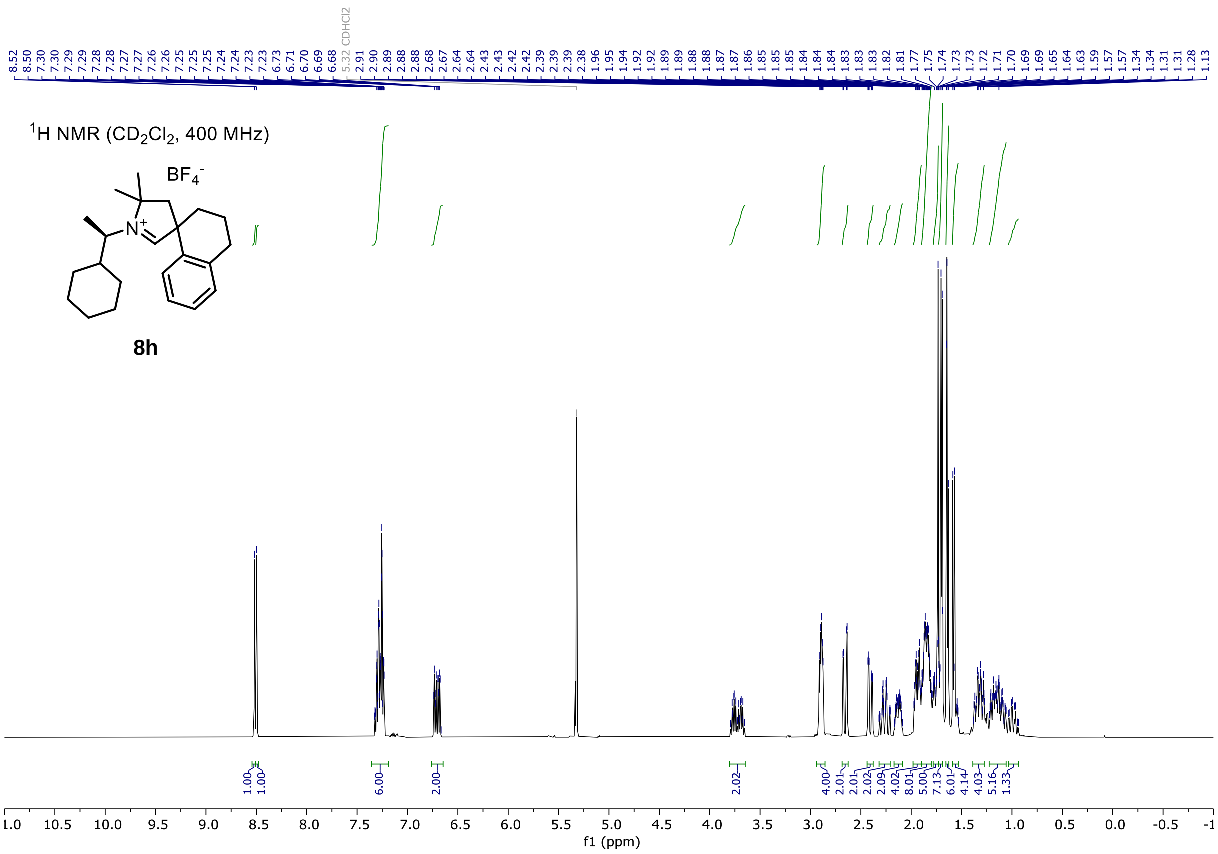

1H NMR (CD2Cl2, 400 MHz)
BF4-
N+
8h
f1 (ppm)

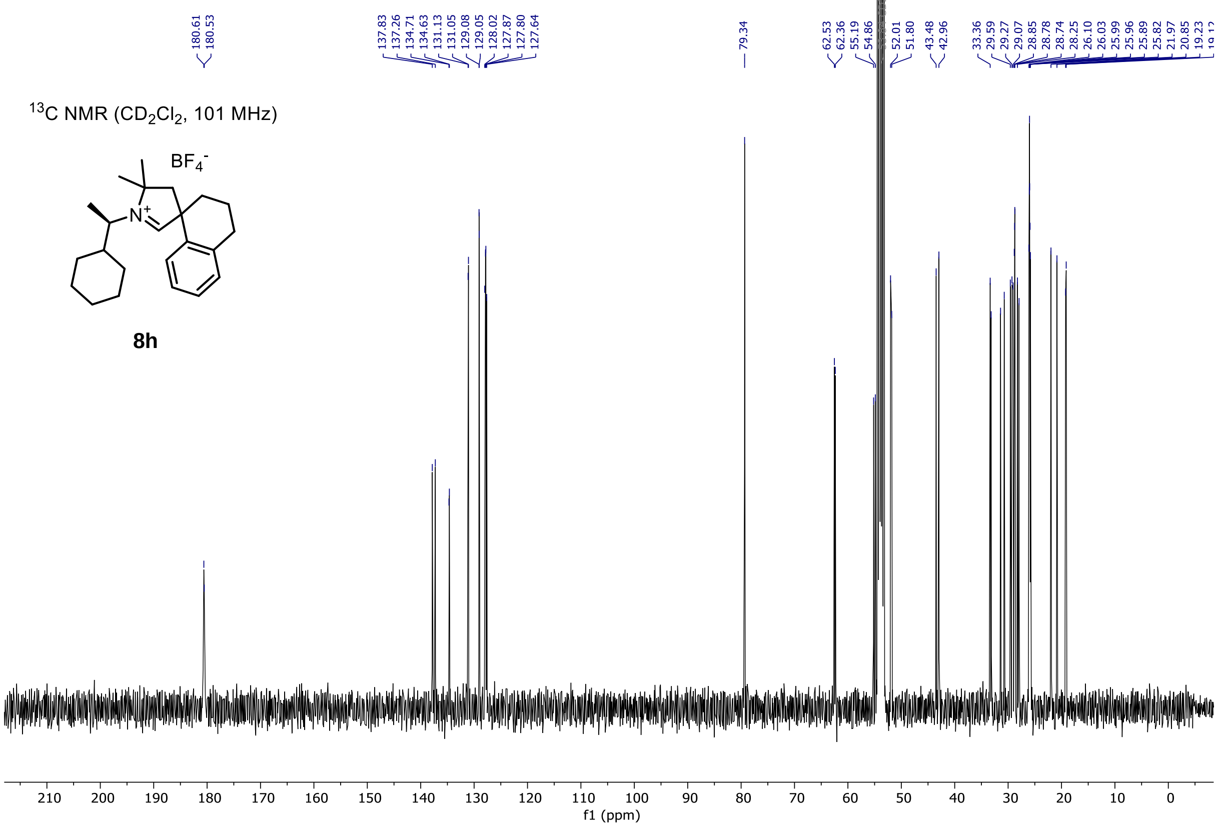

13C NMR (CD2Cl2, 101 MHz)
BF4-
N+
8h
f1 (ppm)

^{19}F NMR (CD_2Cl_2, 376 MHz)

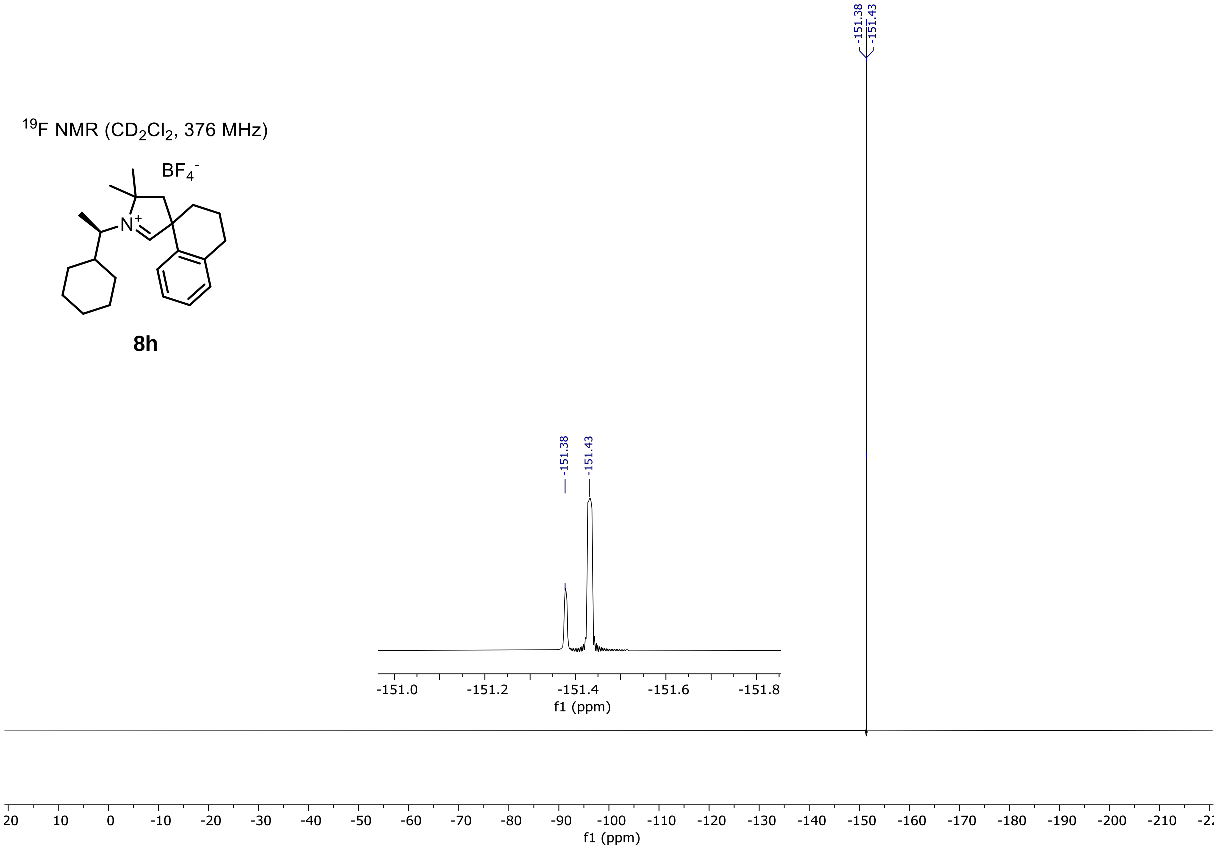

8h

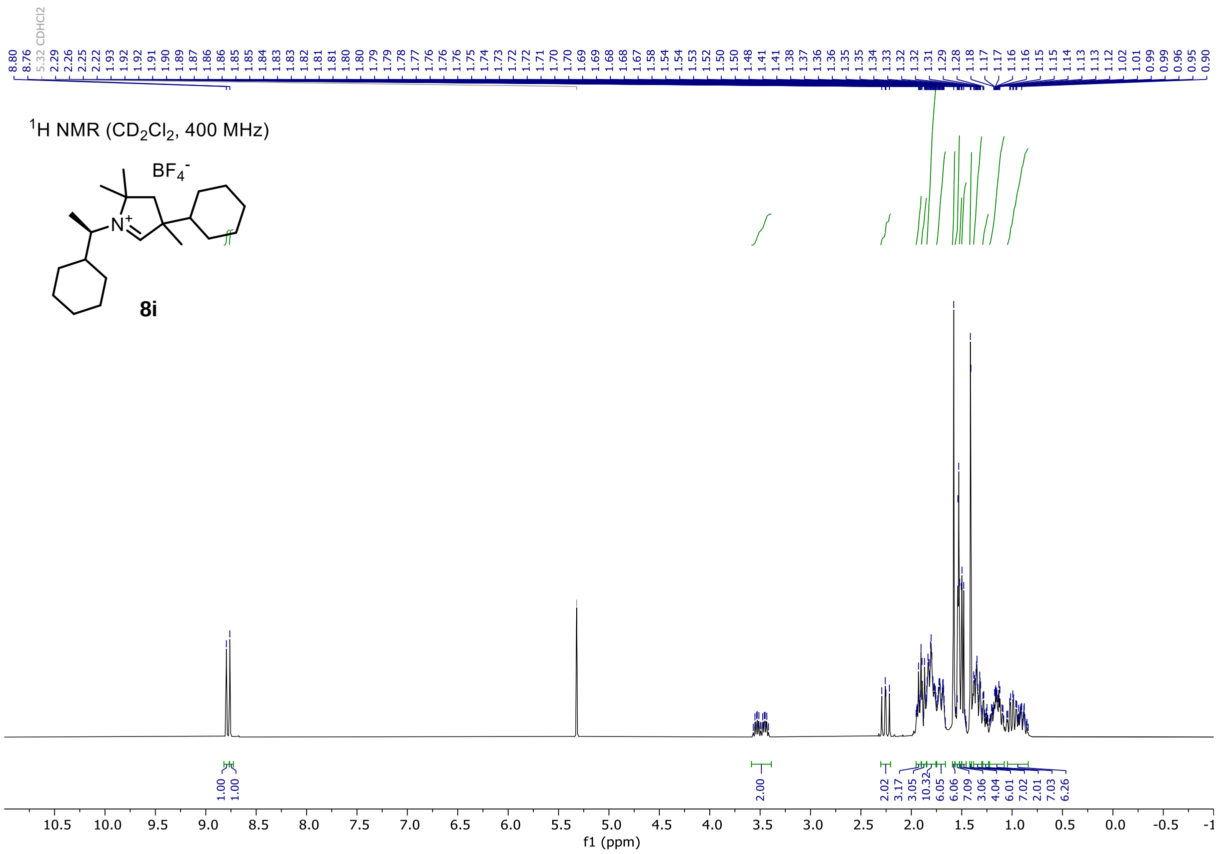

1H NMR (CD2Cl2, 400 MHz)
BF4-
N+
8i
f1 (ppm)

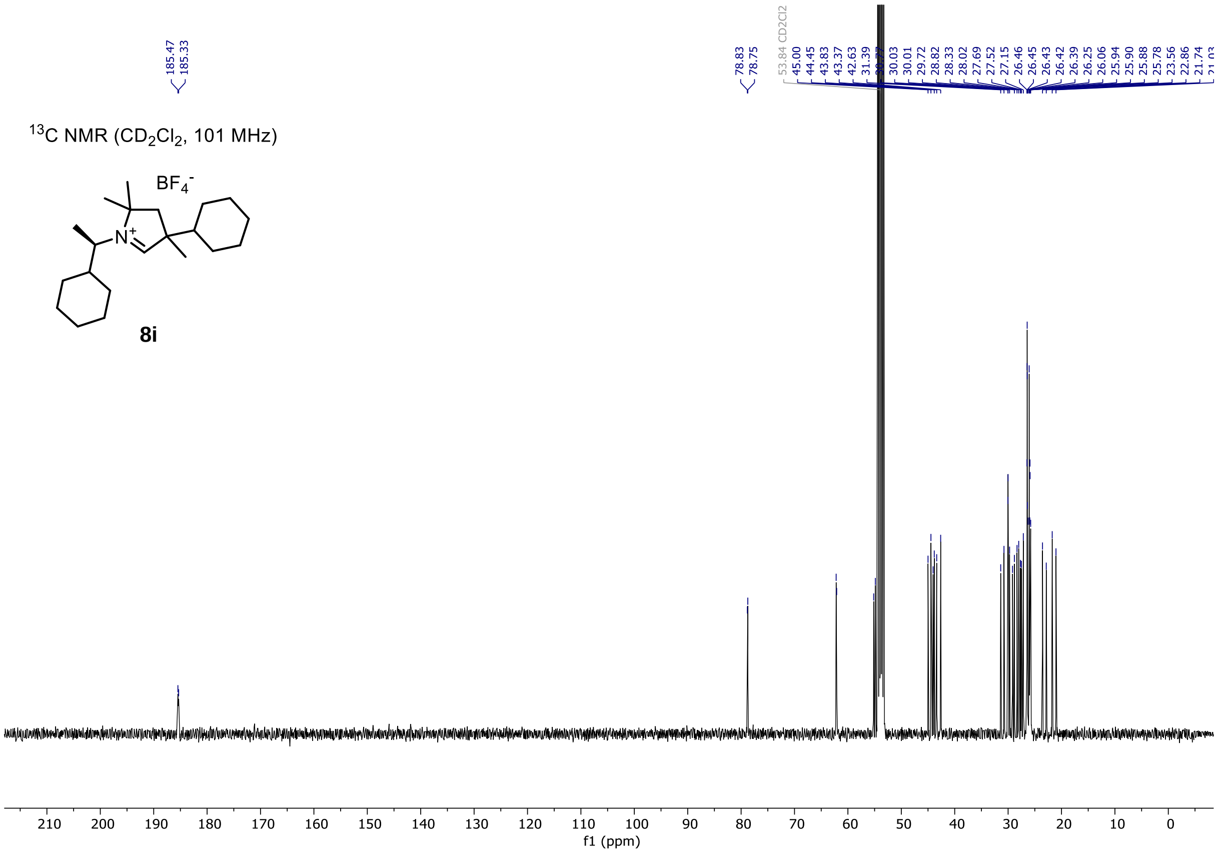

13C NMR (CD2Cl2, 101 MHz)
BF4-
N+
8i
f1 (ppm)

^{19}F NMR (CD_2Cl_2, 376 MHz)

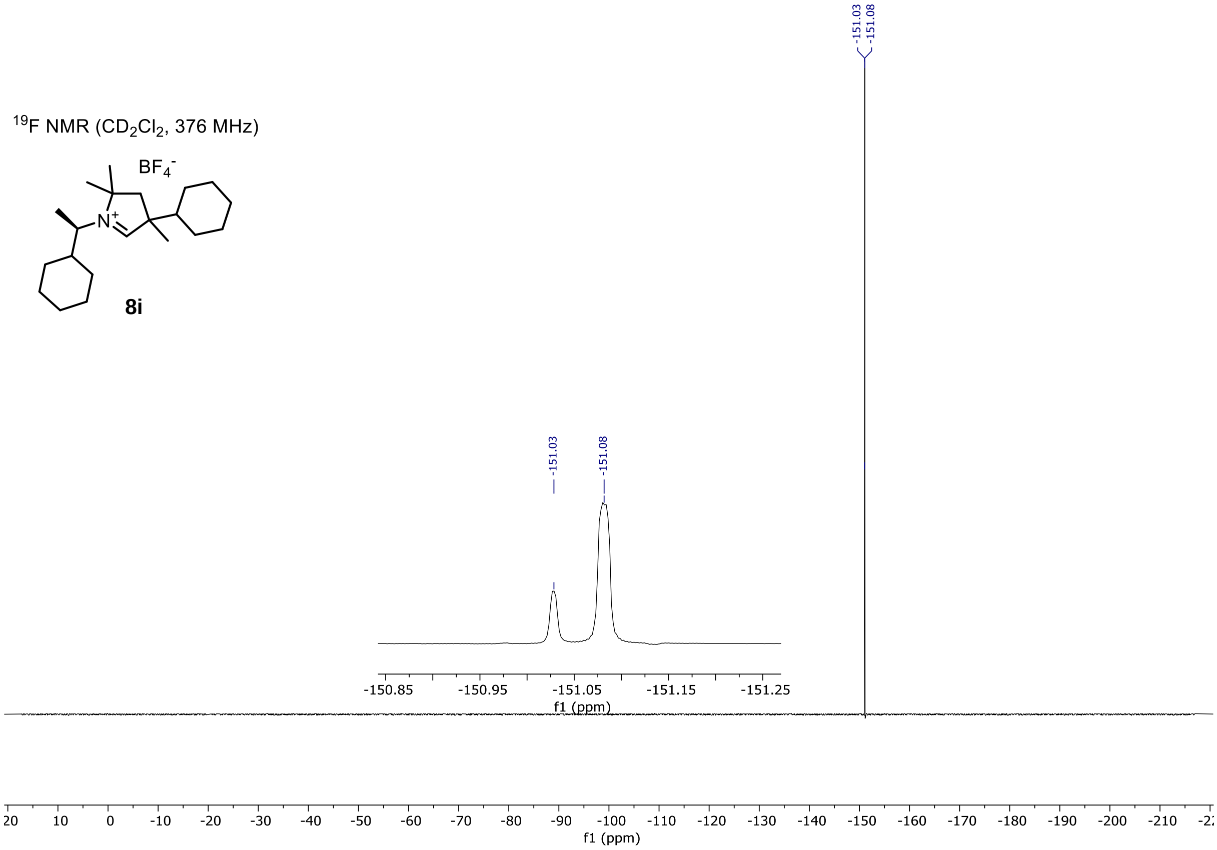

8i

^{1}H NMR (CD_2Cl_2, 400 MHz)

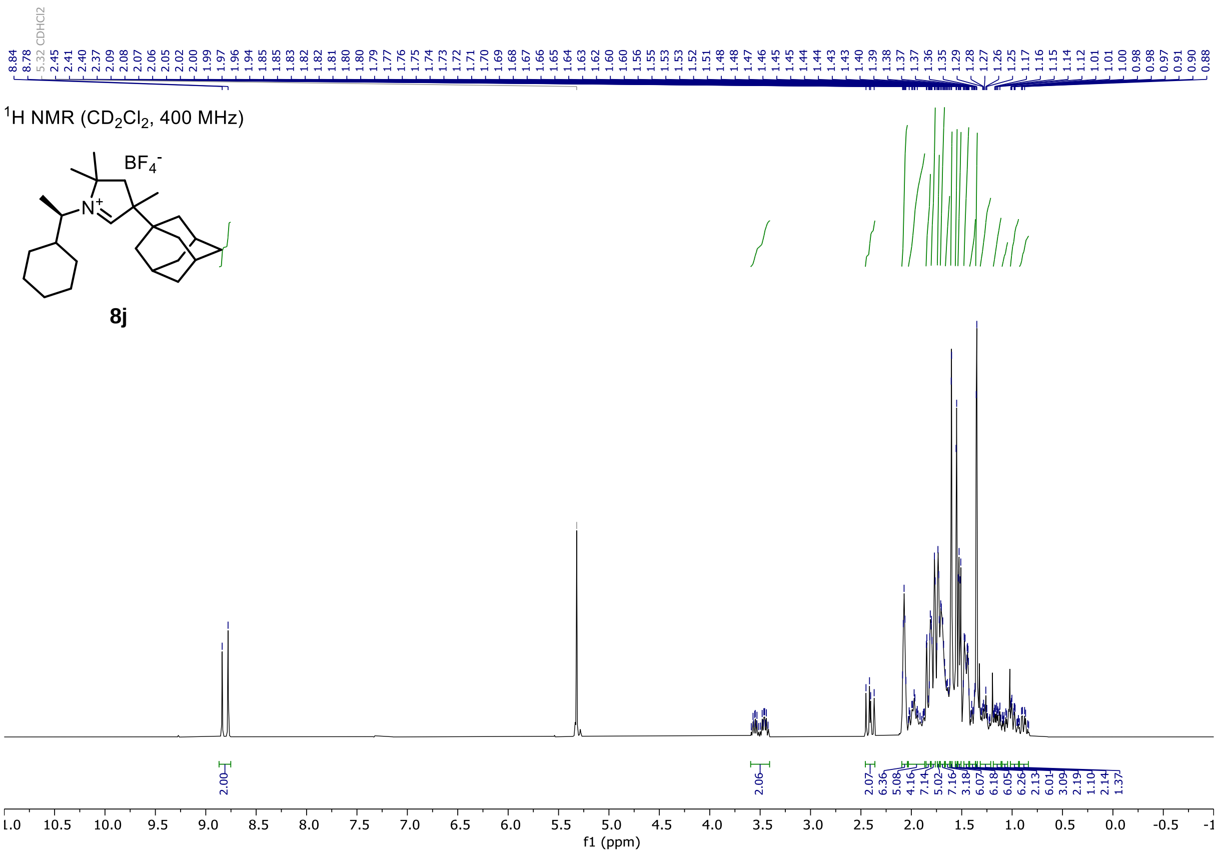

8j

^{13}C NMR (CD_2Cl_2, 101 MHz)

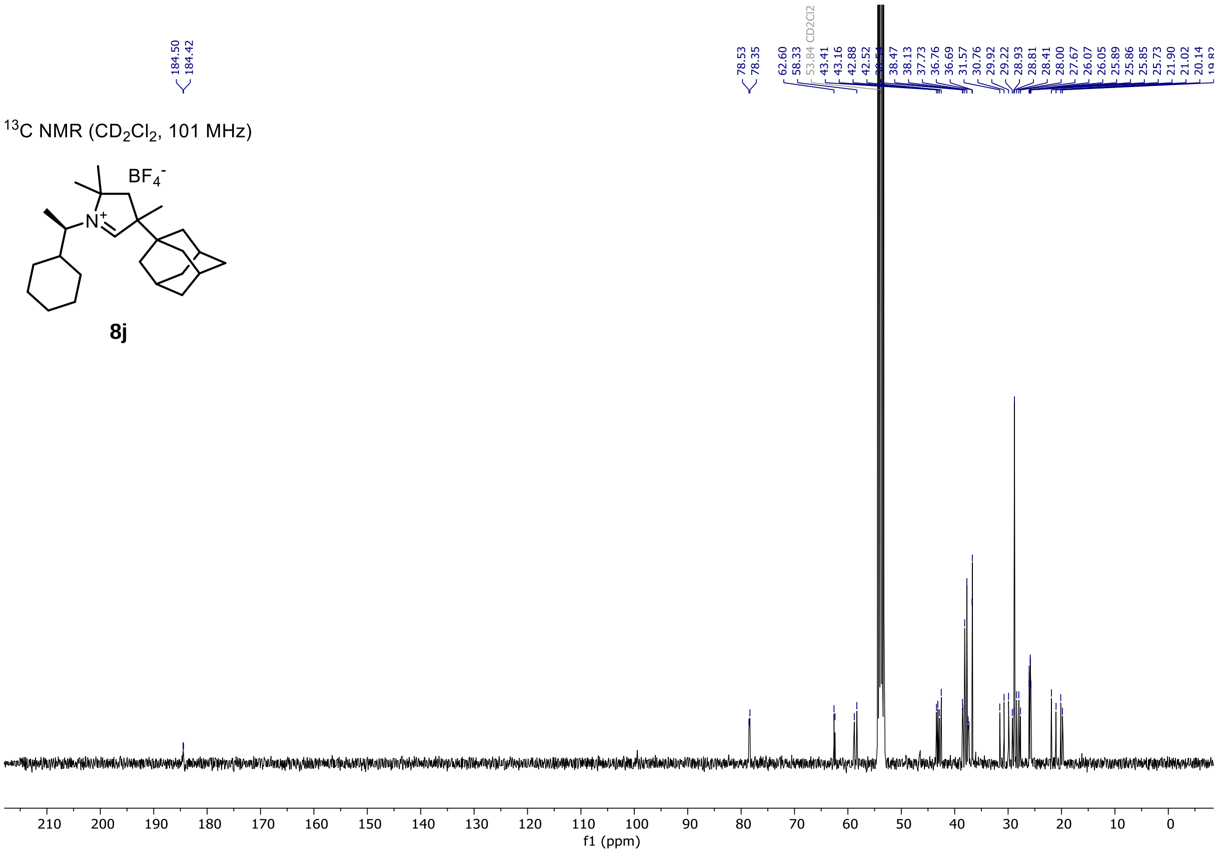

8j

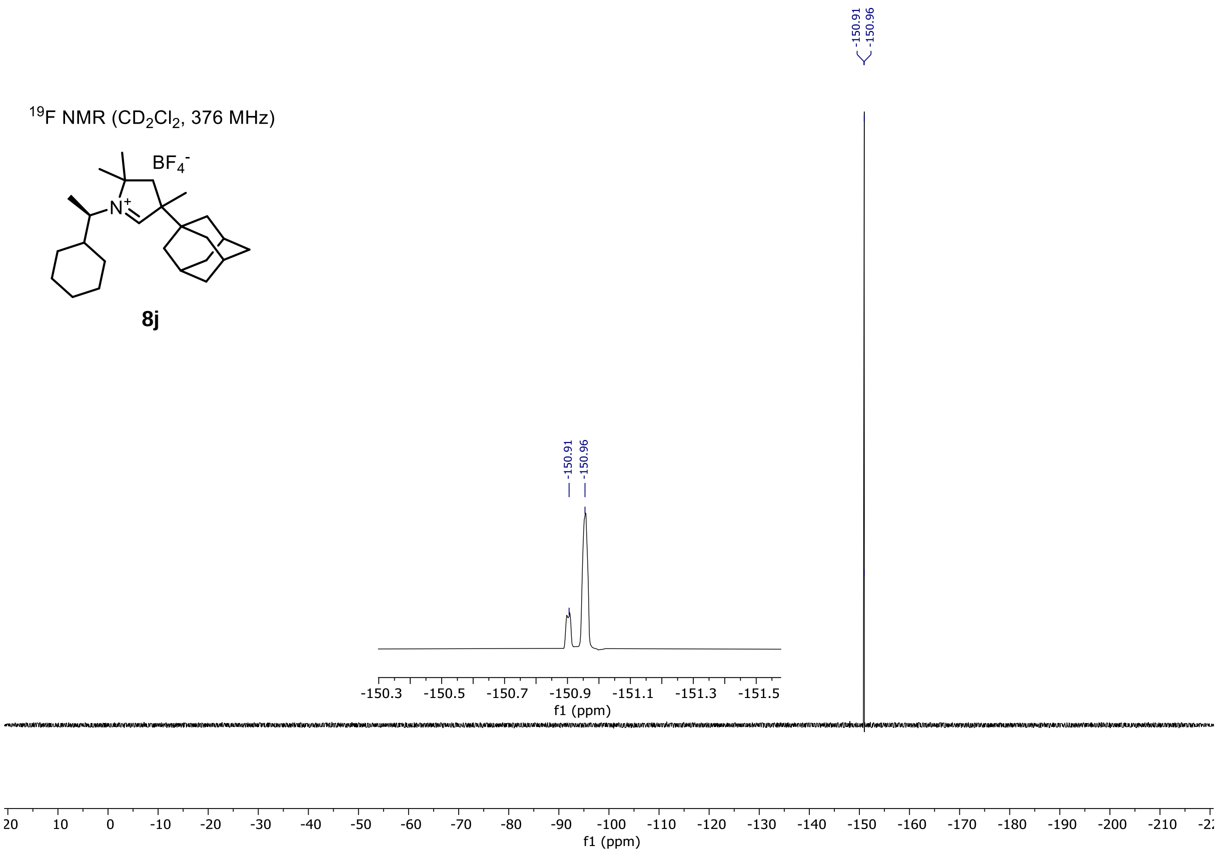

^{19}F NMR (CD$_2$Cl$_2$, 376 MHz)
BF$_4^-$
N+
8j
-150.91
-150.96
-150.3
-150.5
-150.7
-150.9
-151.1
-151.3
-151.5
f1 (ppm)
20 10 0 -10 -20 -30 -40 -50 -60 -70 -80 -90 -100 -110 -120 -130 -140 -150 -160 -170 -180 -190 -200 -210
f1 (ppm)

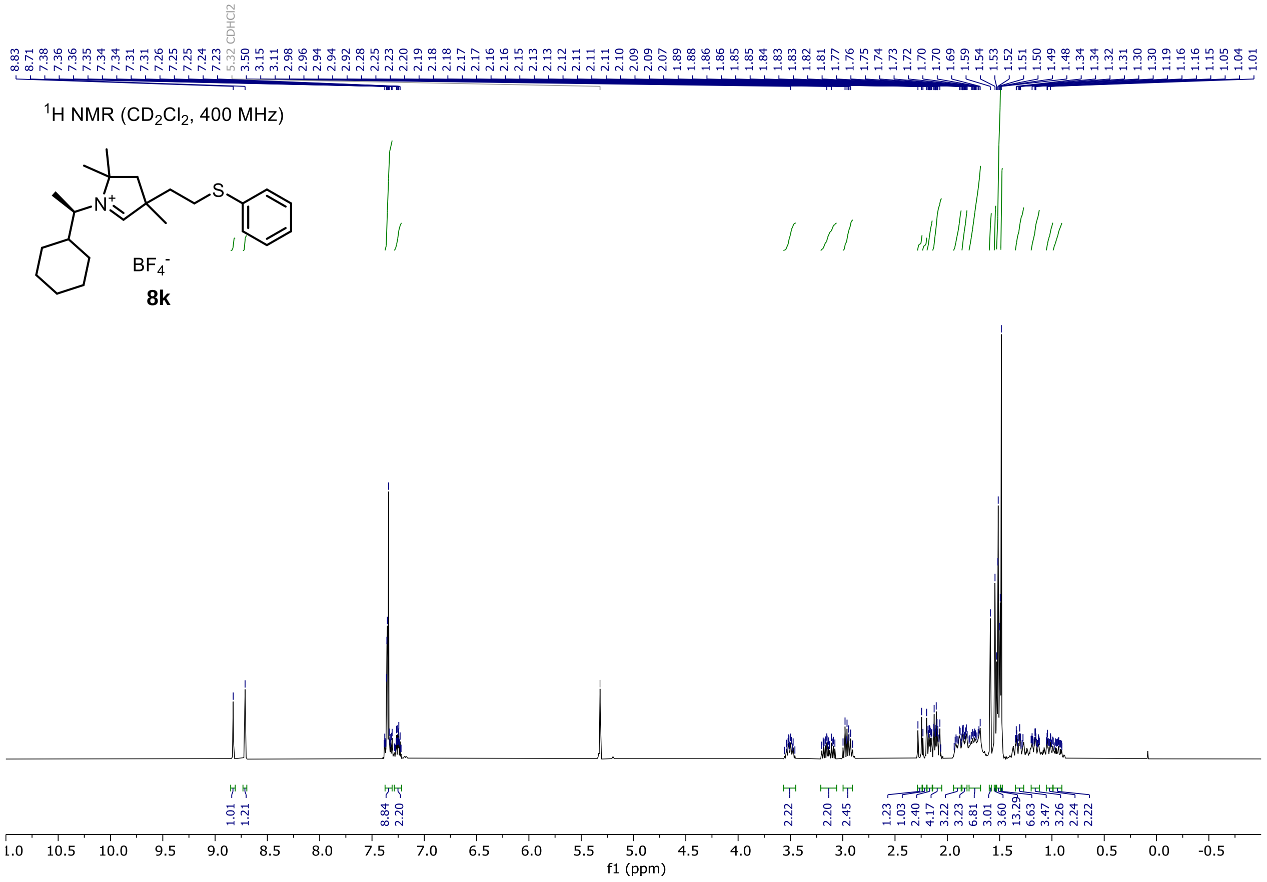

1H NMR (CD_2Cl_2, 400 MHz)

BF_4^-

8k

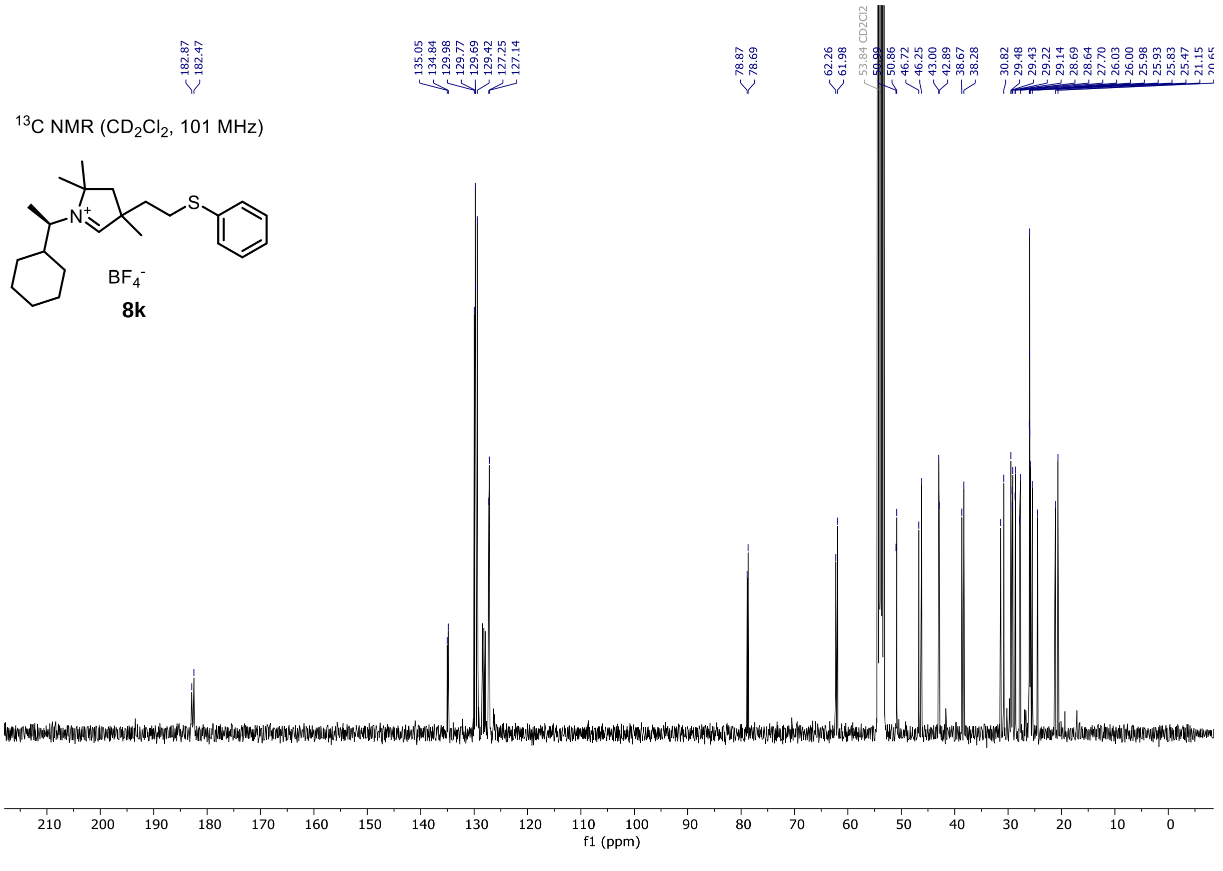

13C NMR (CD_2Cl_2, 101 MHz)

BF_4^-

8k

^{19}F NMR (CD_2Cl_2, 376 MHz)

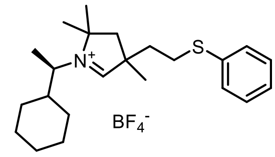

8k

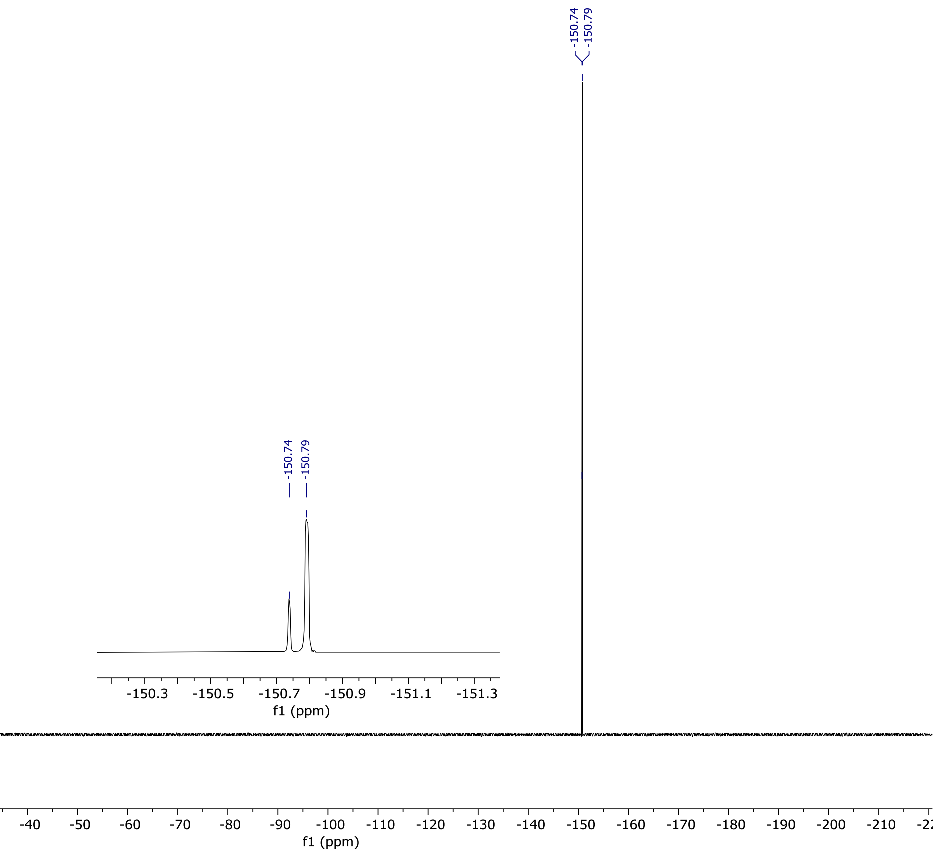

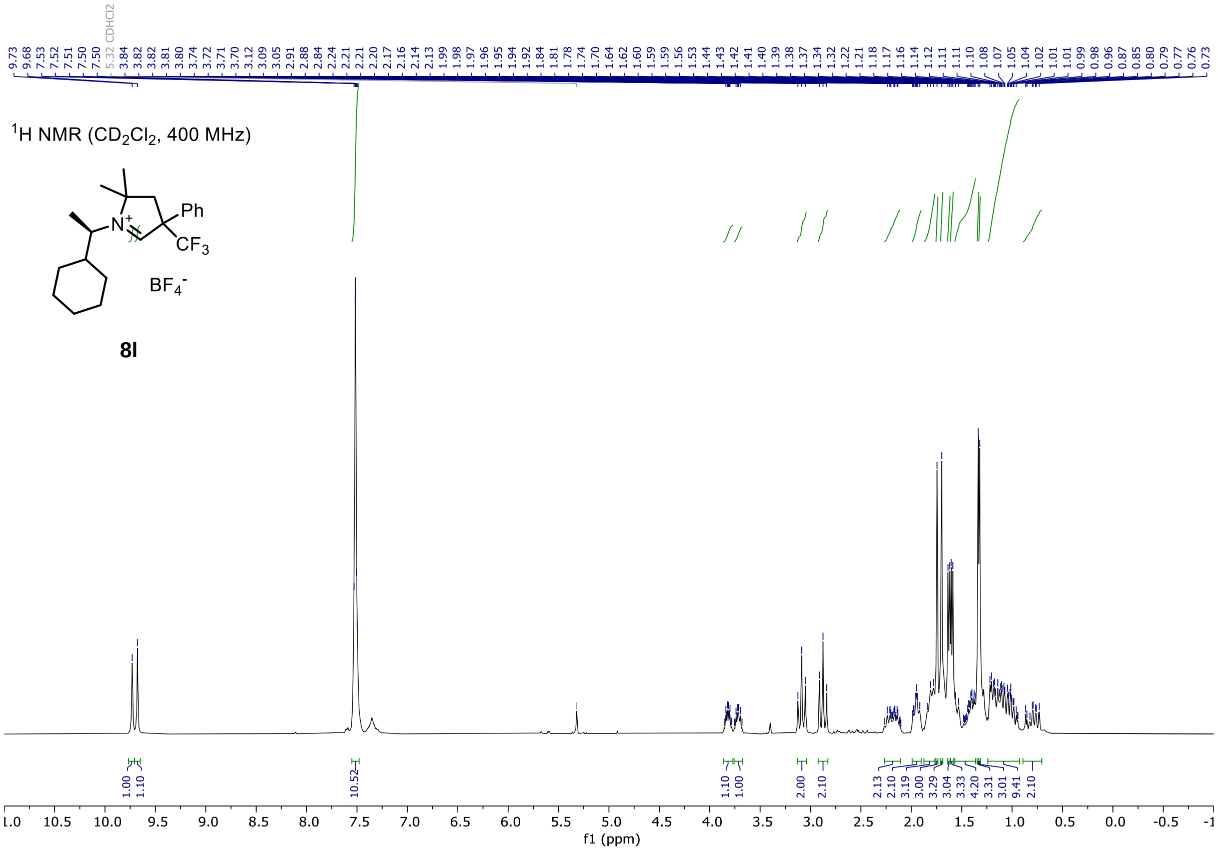
9.73 9.68 7.53 7.52 7.51 7.50 7.50 5.32 CDHCl2 3.84 3.82 3.82 3.81 3.80 3.74 3.72 3.71 3.70 3.12 3.09 3.05 2.91 2.88 2.84 2.24 2.21 2.21 2.20 2.17 2.16 2.14 2.13 1.99 1.98 1.97 1.96 1.95 1.94 1.92 1.84 1.81 1.78 1.74 1.70 1.64 1.62 1.60 1.59 1.59 1.56 1.53 1.44 1.43 1.42 1.41 1.40 1.39 1.38 1.37 1.34 1.32 1.22 1.21 1.18 1.17 1.16 1.14 1.12 1.11 1.11 1.10 1.08 1.07 1.05 1.04 1.02 1.01 1.01 0.99 0.98 0.96 0.87 0.85 0.80 0.79 0.76 0.73
1H NMR (CD2Cl2, 400 MHz)
Ph
N+
CF3
BF4-
8l
1.00 1.10 10.52 1.10 1.00 2.00 2.10 2.13 2.10 3.19 3.00 3.29 3.04 3.33 4.20 3.31 3.01 9.41 2.10
f1 (ppm)

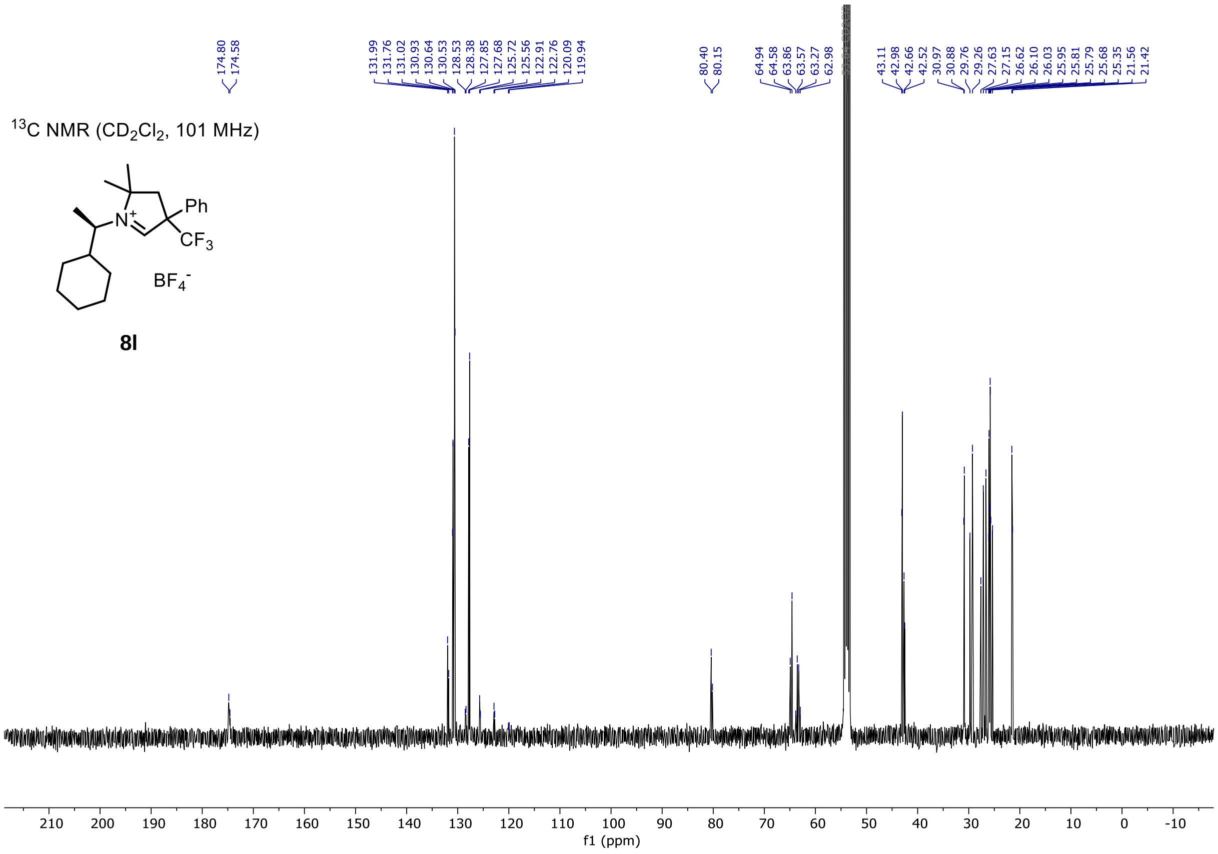
174.80 174.58 131.99 131.76 131.02 130.93 130.64 130.53 128.53 128.38 127.85 127.68 125.72 125.56 122.91 122.76 120.09 119.94 80.40 80.15 64.94 64.58 63.86 63.57 63.27 62.98 43.11 42.98 42.66 42.52 30.97 30.88 29.76 29.26 27.63 27.15 26.62 26.10 26.03 25.95 25.81 25.79 25.68 25.35 21.56 21.42
13C NMR (CD2Cl2, 101 MHz)
Ph
N+
CF3
BF4-
8l
f1 (ppm)

^{19}F NMR (CD_2Cl_2, 376 MHz)

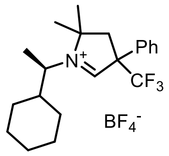

8l

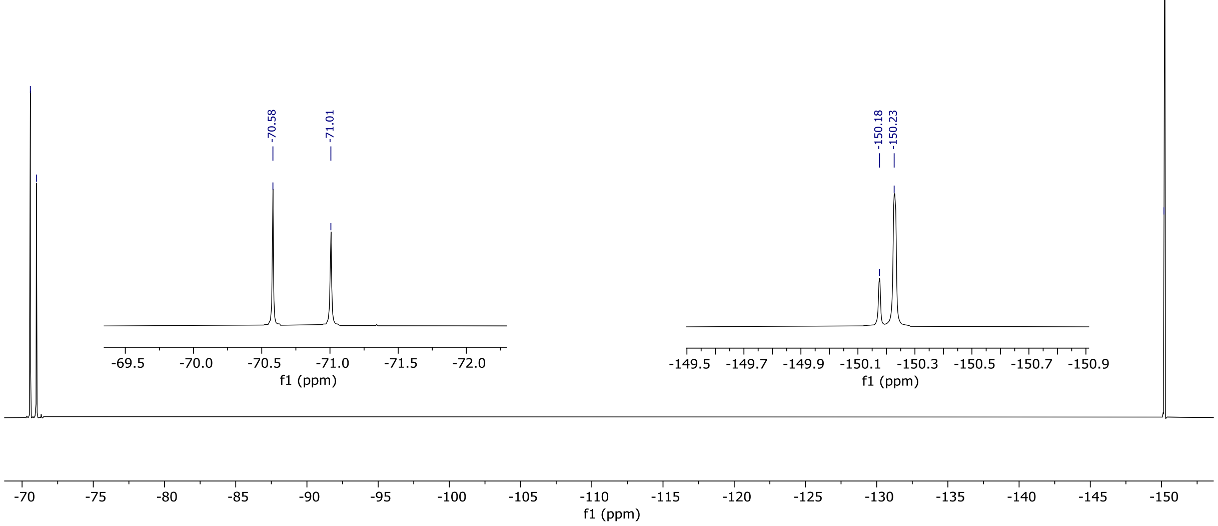

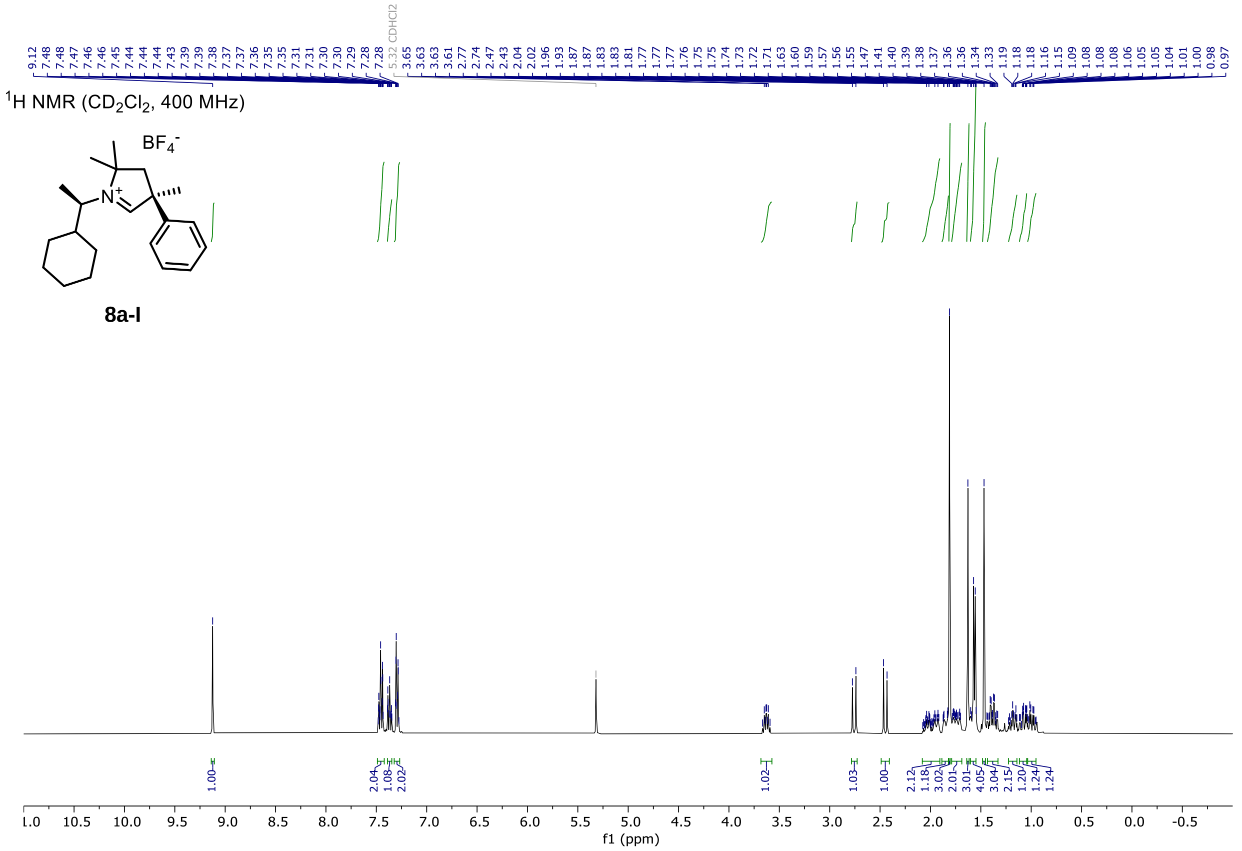

9.12 7.48 7.47 7.46 7.45 7.44 7.44 7.44 7.43 7.39 7.39 7.38 7.37 7.37 7.36 7.35 7.31 7.31 7.30 7.30 7.29 7.28 5.32 CDHCl2 3.65 3.63 3.63 3.61 2.77 2.74 2.47 2.43 2.04 2.02 1.96 1.93 1.87 1.87 1.83 1.83 1.81 1.77 1.77 1.77 1.76 1.75 1.75 1.74 1.73 1.72 1.71 1.63 1.60 1.59 1.57 1.56 1.55 1.47 1.41 1.40 1.39 1.38 1.37 1.36 1.36 1.34 1.33 1.19 1.18 1.18 1.16 1.15 1.09 1.08 1.08 1.08 1.06 1.05 1.05 1.04 1.01 1.00 0.98 0.97
^1H NMR (CD_2Cl_2, 400 MHz)
BF4-
N+
8a-I
1.00 2.04 1.08 2.02 1.02 1.03 1.00 2.12 1.18 3.02 2.01 3.01 4.05 3.04 2.15 1.20 1.24 1.24
f1 (ppm)

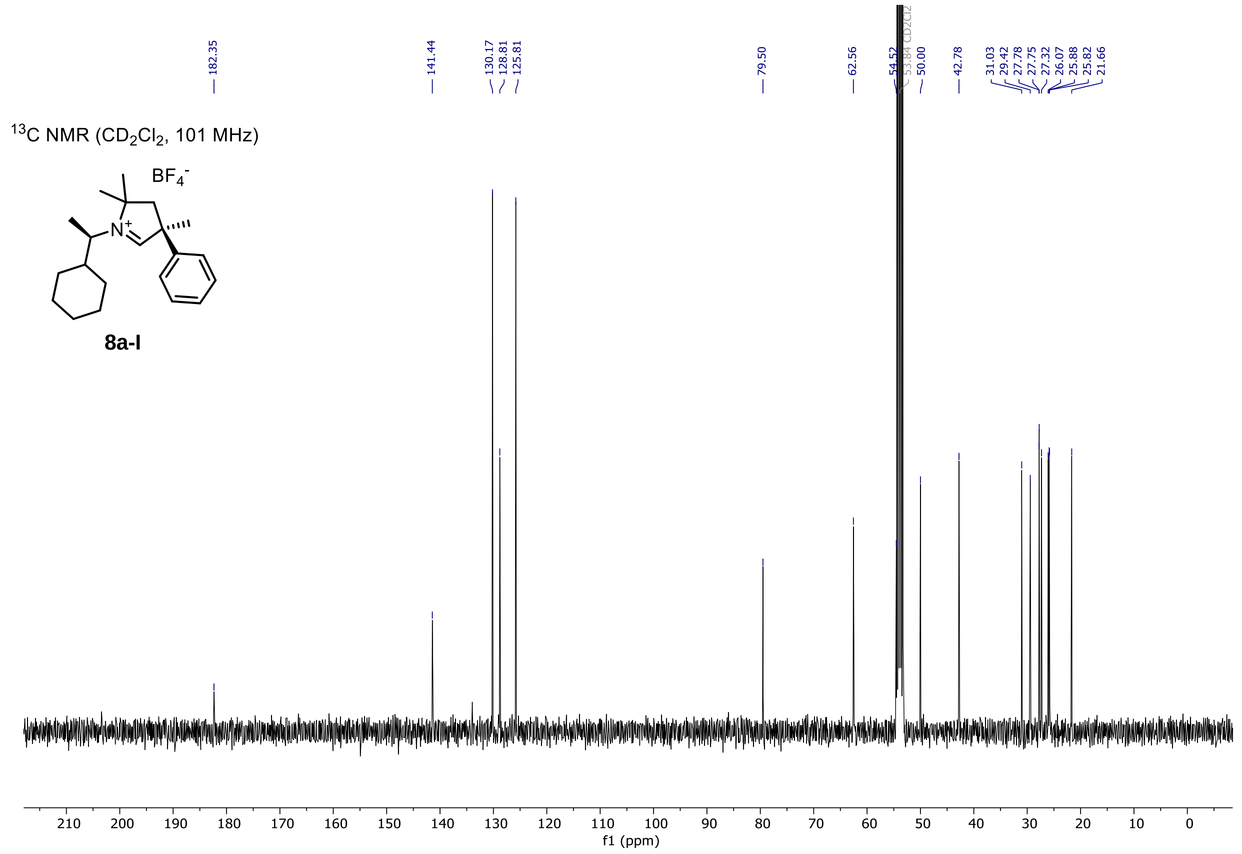

182.35 141.44 130.17 128.81 125.81 79.50 62.56 54.52 53.84 CD2Cl2 50.00 42.78 31.03 29.42 27.78 27.75 27.32 26.07 25.88 25.82 21.66
^{13}C NMR (CD_2Cl_2, 101 MHz)
BF4-
N+
8a-I
f1 (ppm)

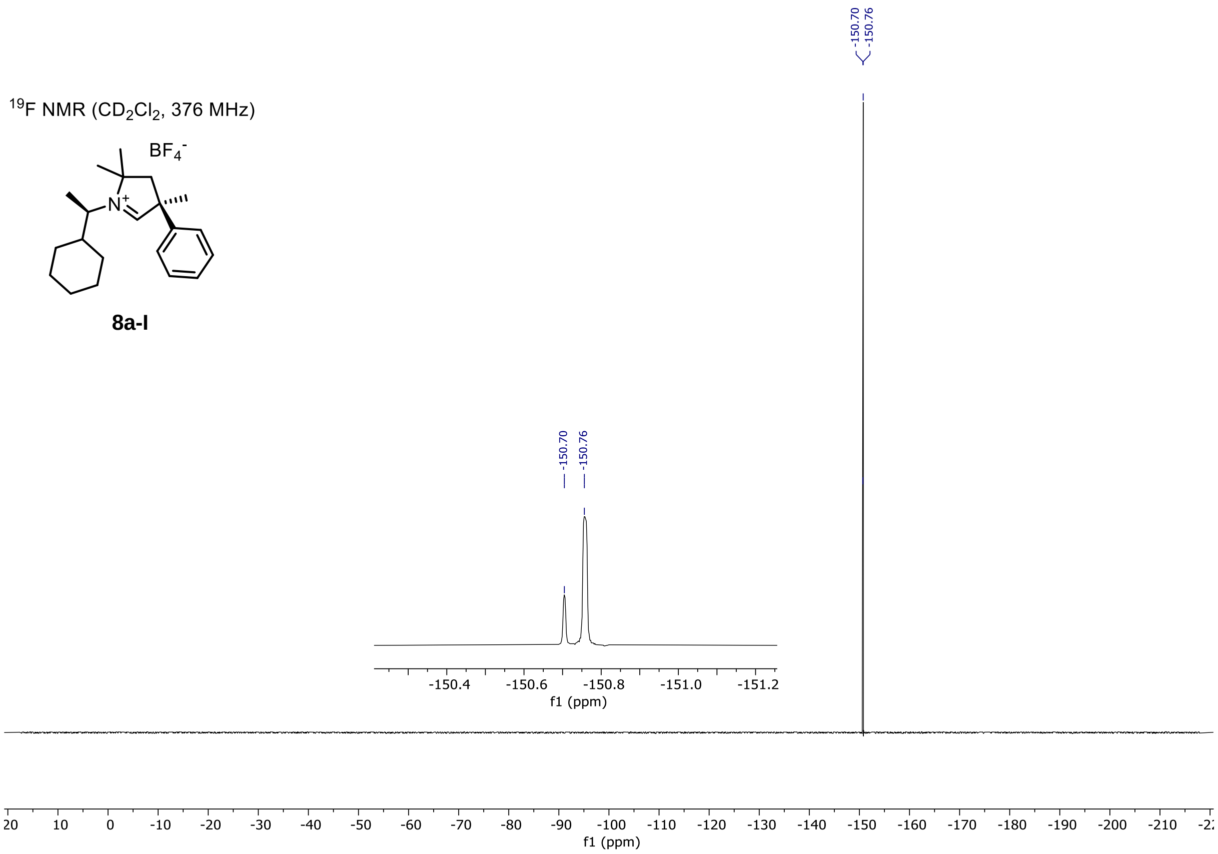
19F NMR (CD2Cl2, 376 MHz)
BF4-
N+
8a-I
-150.70
-150.76
-150.4 -150.6 -150.8 -151.0 -151.2
f1 (ppm)
20 10 0 -10 -20 -30 -40 -50 -60 -70 -80 -90 -100 -110 -120 -130 -140 -150 -160 -170 -180 -190 -200 -210
f1 (ppm)

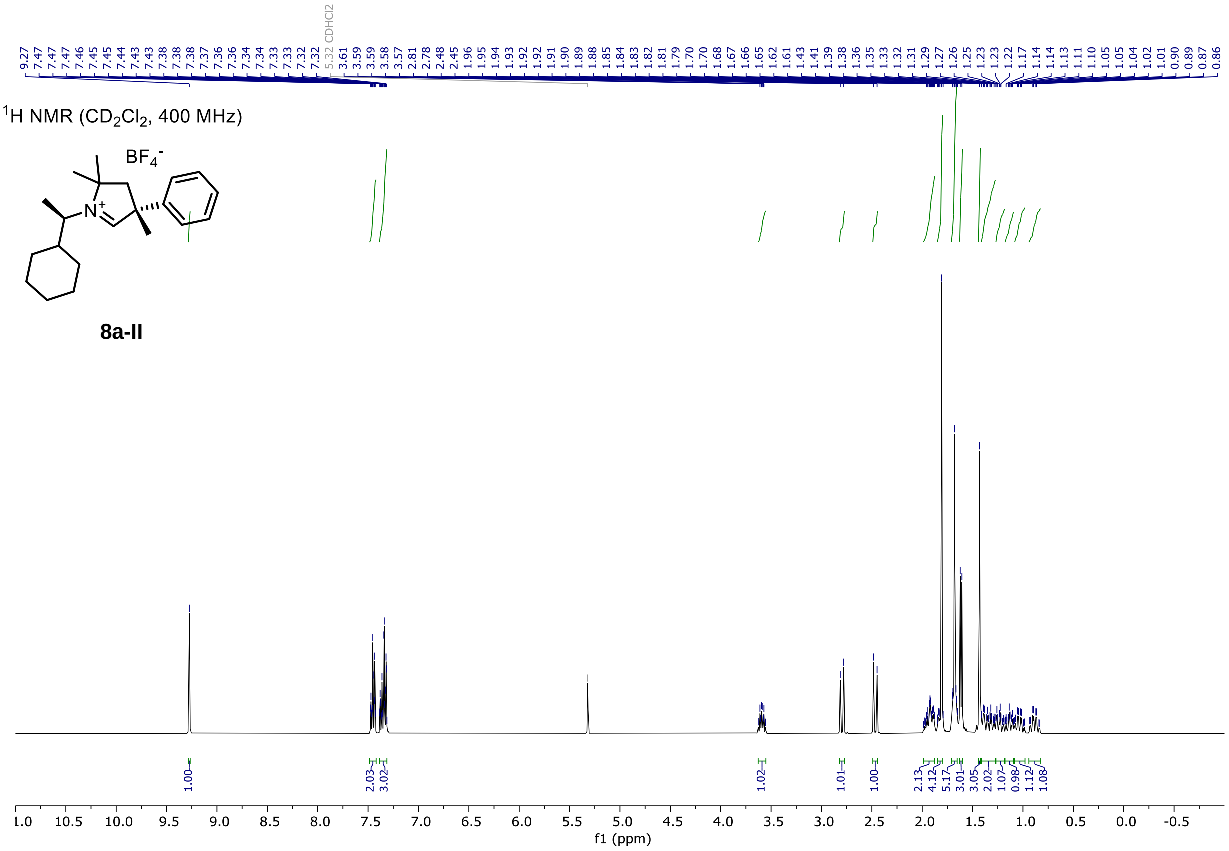

^{1}H NMR (CD_2Cl_2, 400 MHz)
BF$_4^-$
N+
8a-II
5.32 CDHCl2
1.00
2.03
3.02
1.02
1.01
1.00
2.13
4.12
5.17
3.01
3.05
2.02
1.07
0.98
1.12
1.08
f1 (ppm)

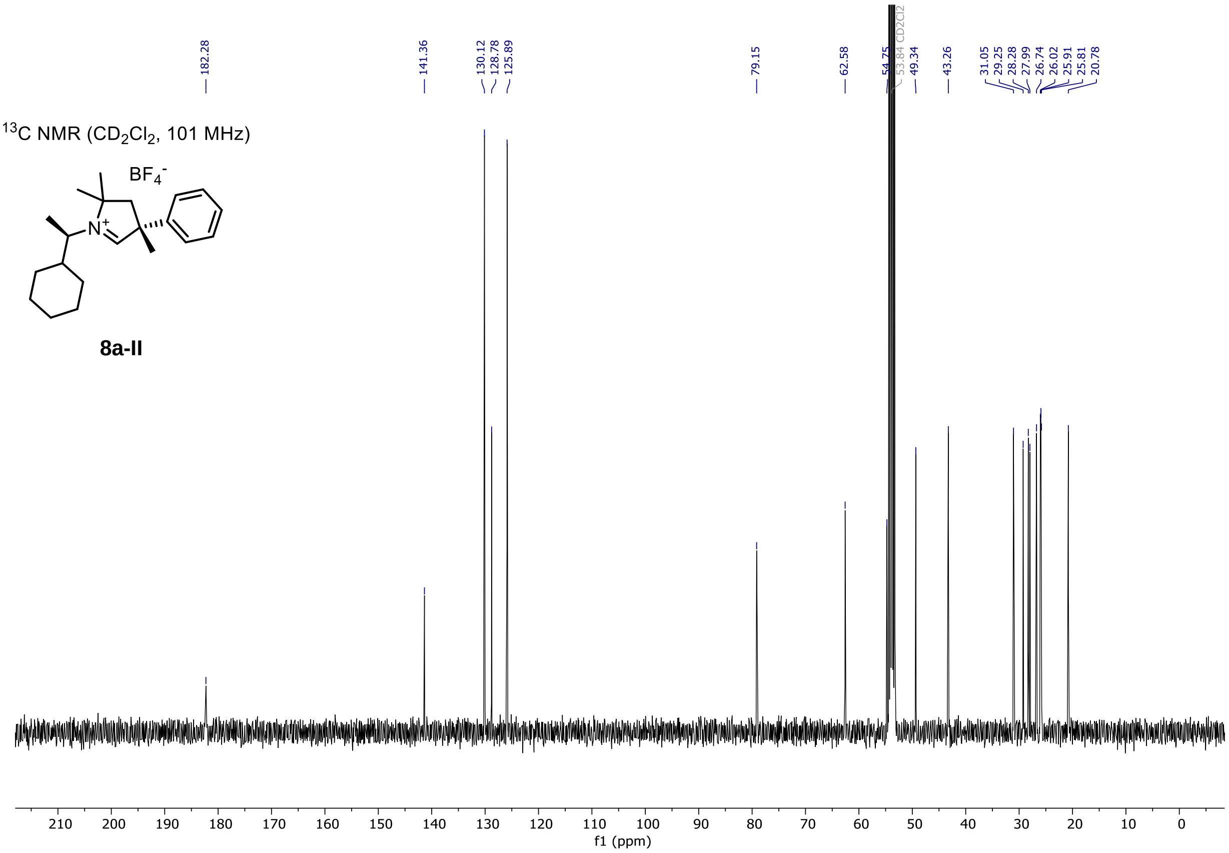

^{13}C NMR (CD_2Cl_2, 101 MHz)
BF$_4^-$
N+
8a-II
182.28
141.36
130.12
128.78
125.89
79.15
62.58
54.75
53.84 CD2Cl2
49.34
43.26
31.05
29.25
28.28
27.99
26.74
26.02
25.91
25.81
20.78
f1 (ppm)

^{19}F NMR (CD_2Cl_2, 376 MHz)

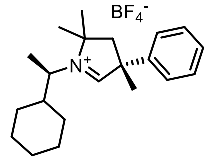

8a-II

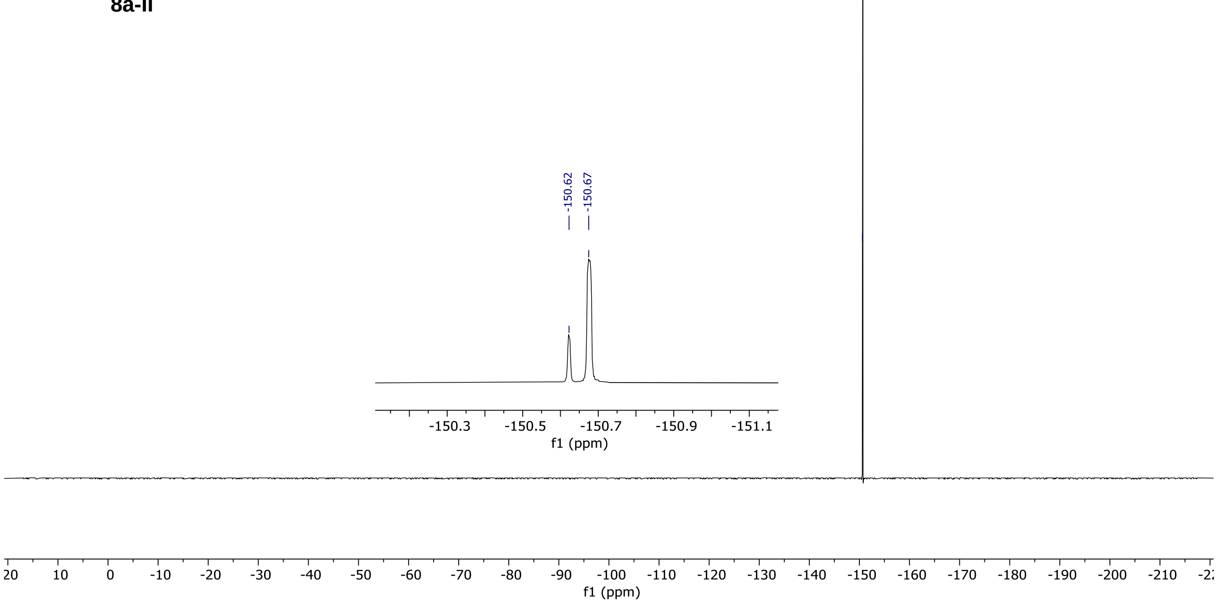

^{1}H NMR (CD_2Cl_2, 400 MHz)

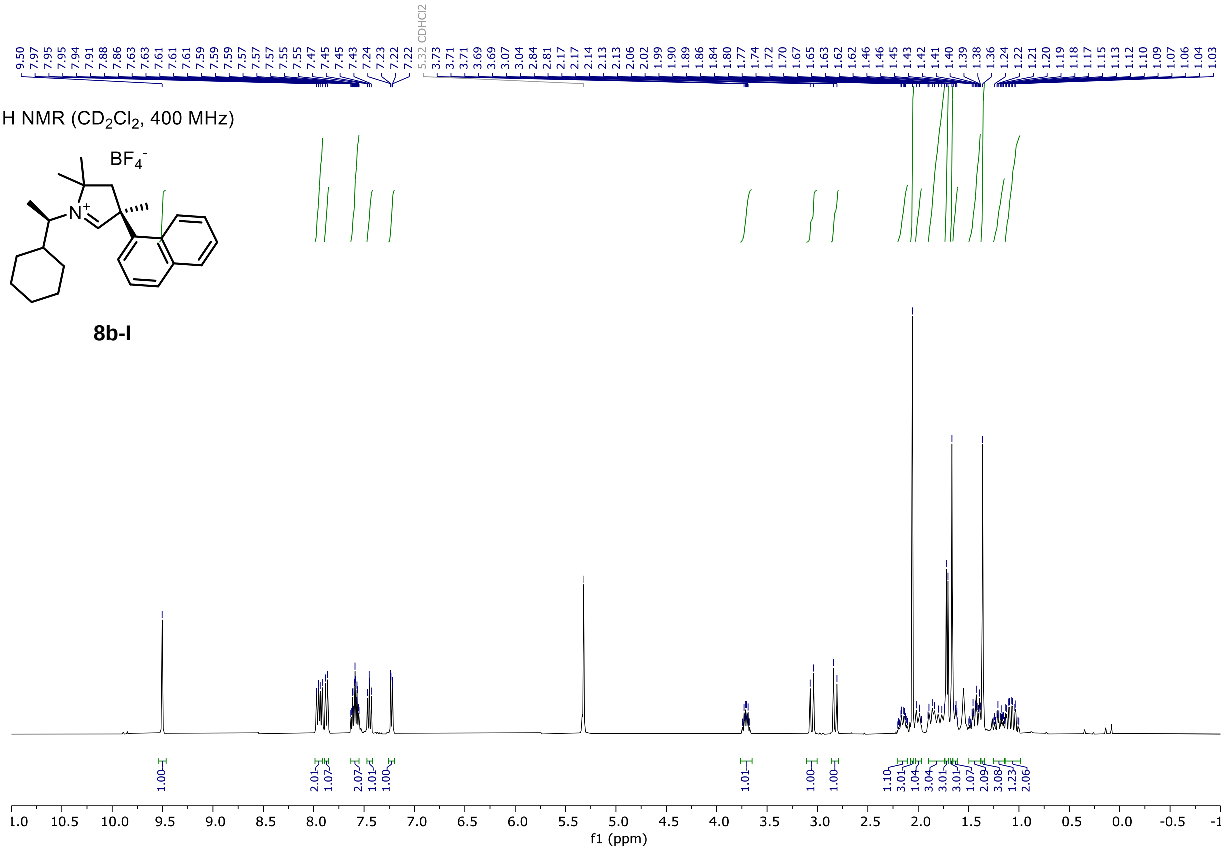

8b-I

^{13}C NMR (CD_2Cl_2, 101 MHz)

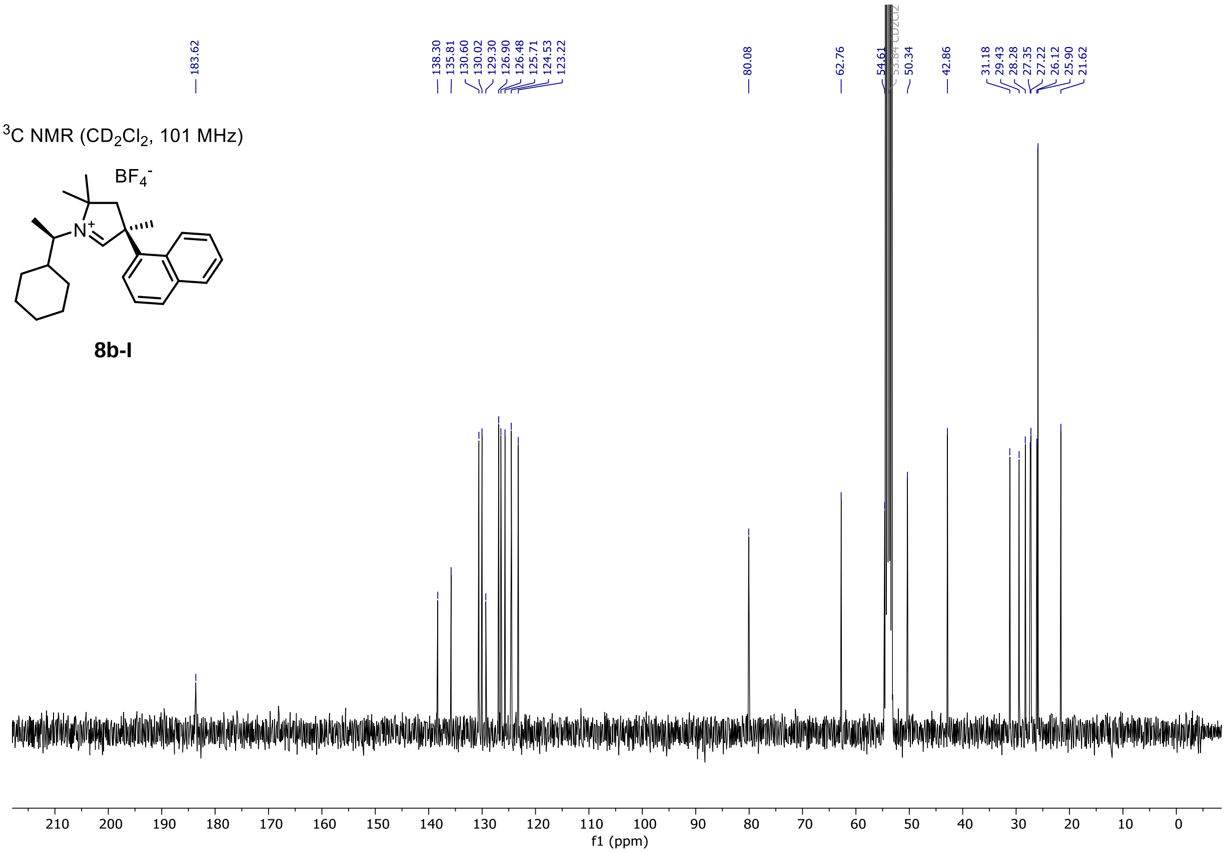

8b-I

^{19}F NMR (CD_2Cl_2, 376 MHz)

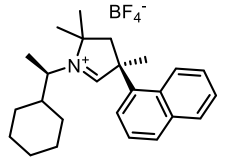

8b-I

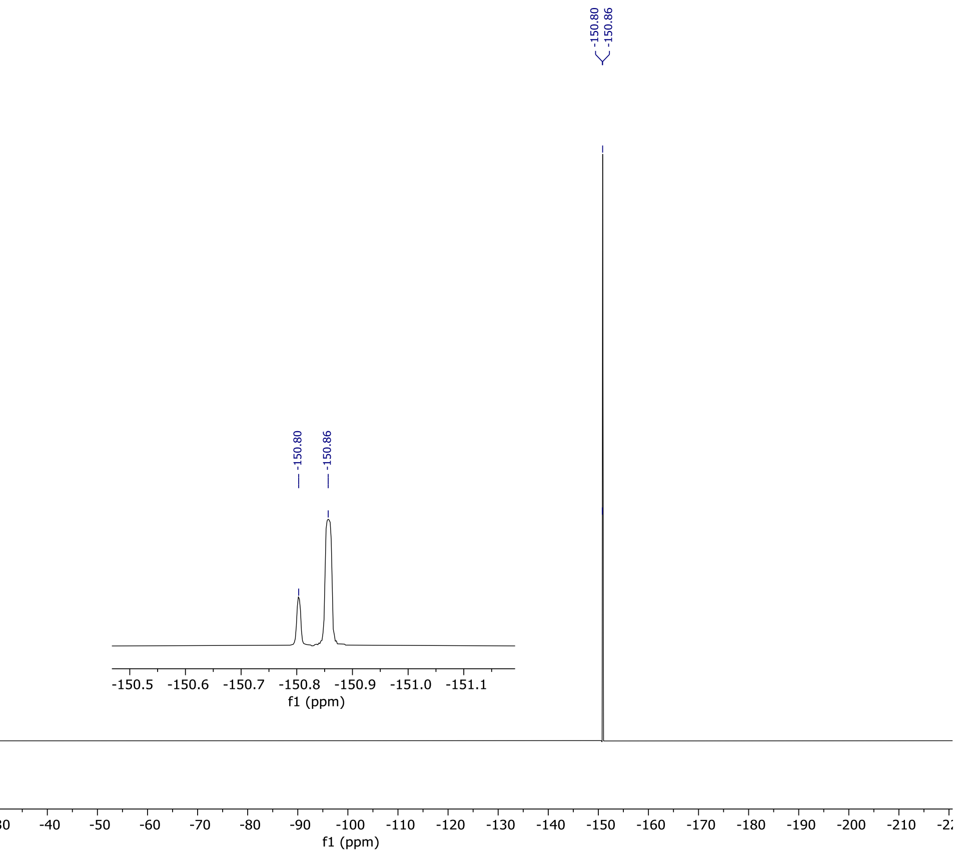

^{1}H NMR (CD_2Cl_2, 400 MHz)

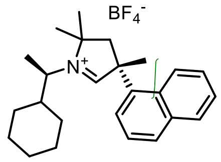

8b-II

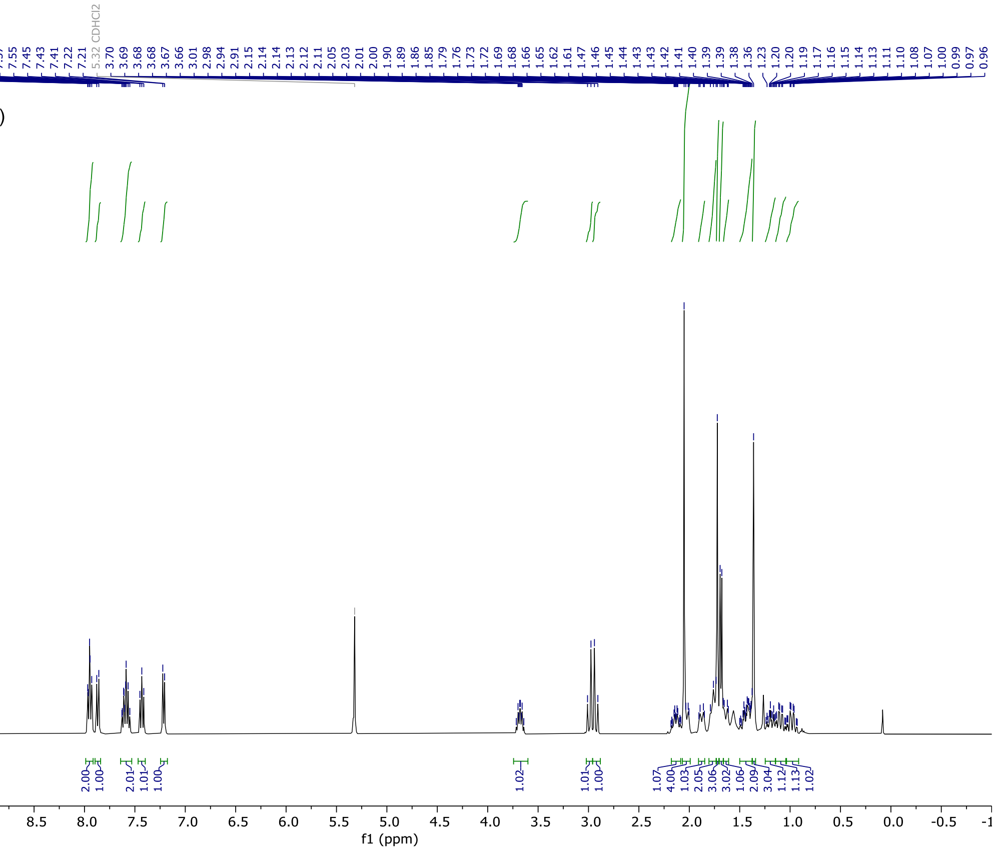

^{13}C NMR (CD_2Cl_2, 101 MHz)

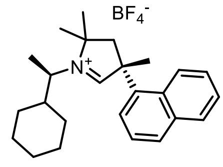

8b-II

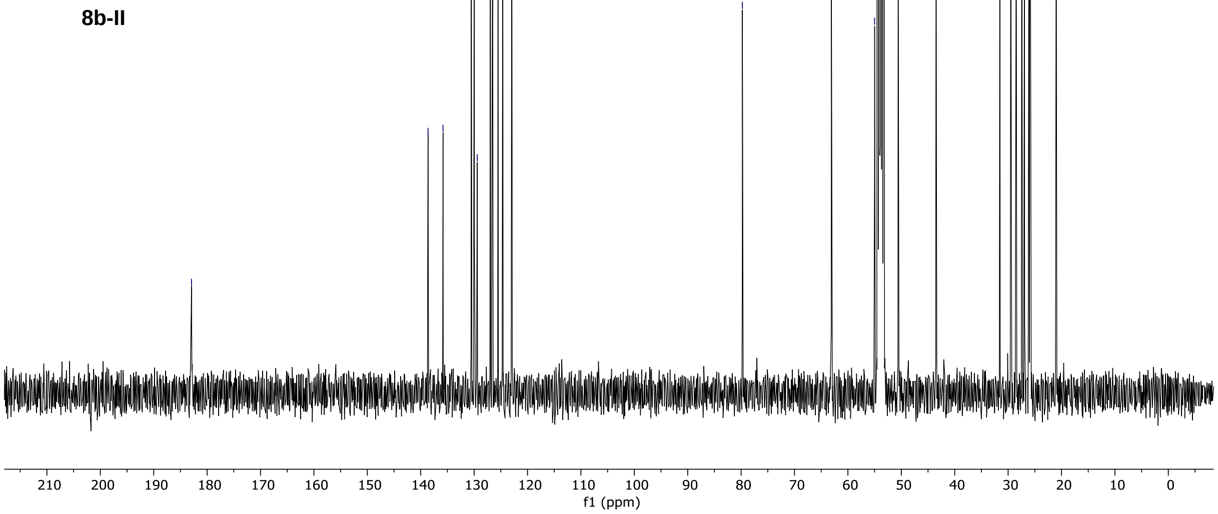

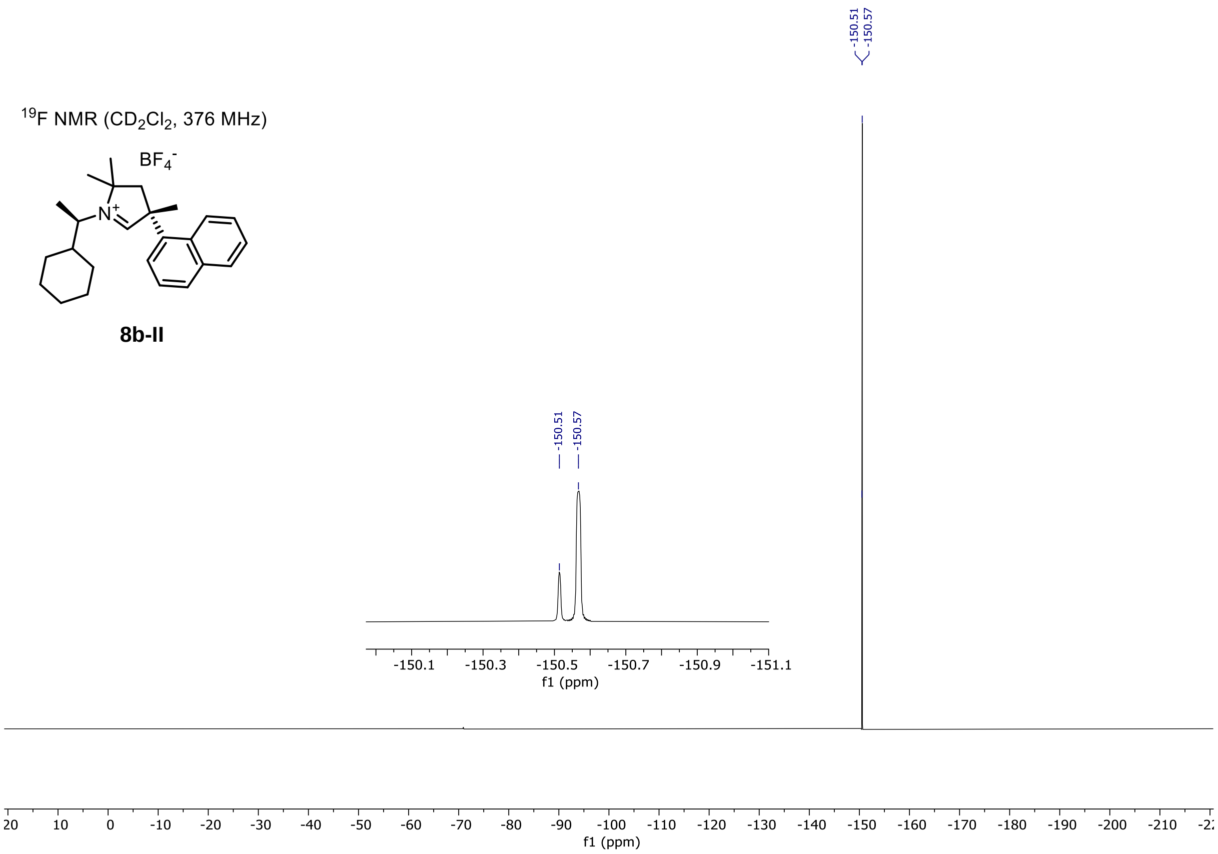

^{19}F NMR (CD_2Cl_2, 376 MHz)
BF_4^-
8b-II
-150.51
-150.57
-150.1 -150.3 -150.5 -150.7 -150.9 -151.1
f1 (ppm)
20 10 0 -10 -20 -30 -40 -50 -60 -70 -80 -90 -100 -110 -120 -130 -140 -150 -160 -170 -180 -190 -200 -210
f1 (ppm)

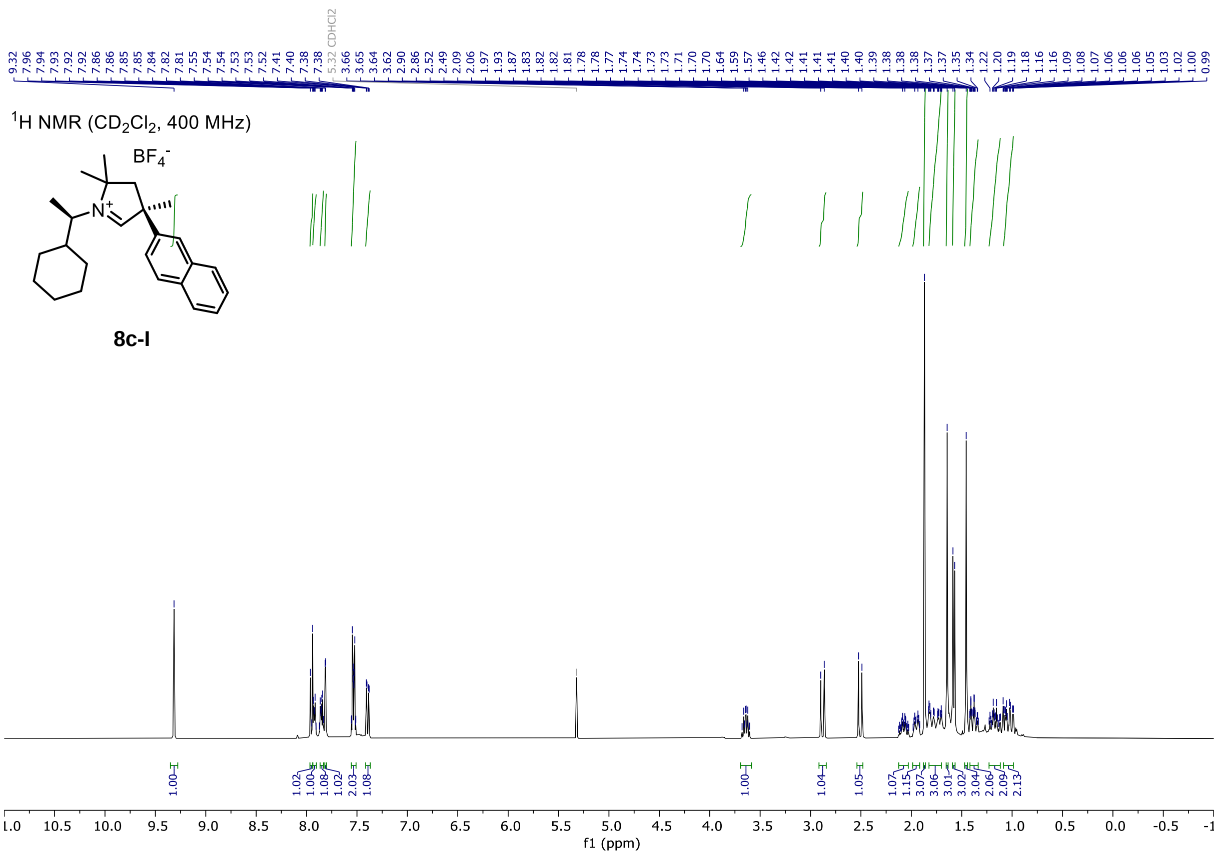
9.32 7.96 7.94 7.93 7.92 7.92 7.86 7.86 7.85 7.84 7.82 7.81 7.55 7.54 7.54 7.53 7.53 7.52 7.41 7.40 7.38 7.38 5.32 CDHCl2 3.66 3.65 3.64 3.62 2.90 2.86 2.52 2.49 2.09 2.06 1.97 1.93 1.87 1.83 1.82 1.82 1.81 1.78 1.78 1.77 1.74 1.74 1.73 1.73 1.71 1.70 1.64 1.59 1.57 1.46 1.42 1.42 1.41 1.41 1.41 1.40 1.40 1.39 1.38 1.38 1.38 1.37 1.37 1.35 1.34 1.22 1.20 1.19 1.18 1.16 1.16 1.09 1.08 1.07 1.06 1.06 1.05 1.03 1.02 1.00 0.99
^{1}H NMR (CD_2Cl_2, 400 MHz)
BF_4^-
N+
8c-I
1.00 1.02 1.00 1.08 1.02 2.03 1.08 1.00 1.04 1.05 1.07 1.15 3.07 3.06 3.01 3.02 3.04 2.06 2.09 2.13
1.0 10.5 10.0 9.5 9.0 8.5 8.0 7.5 7.0 6.5 6.0 5.5 5.0 4.5 4.0 3.5 3.0 2.5 2.0 1.5 1.0 0.5 0.0 -0.5 -1
f1 (ppm)

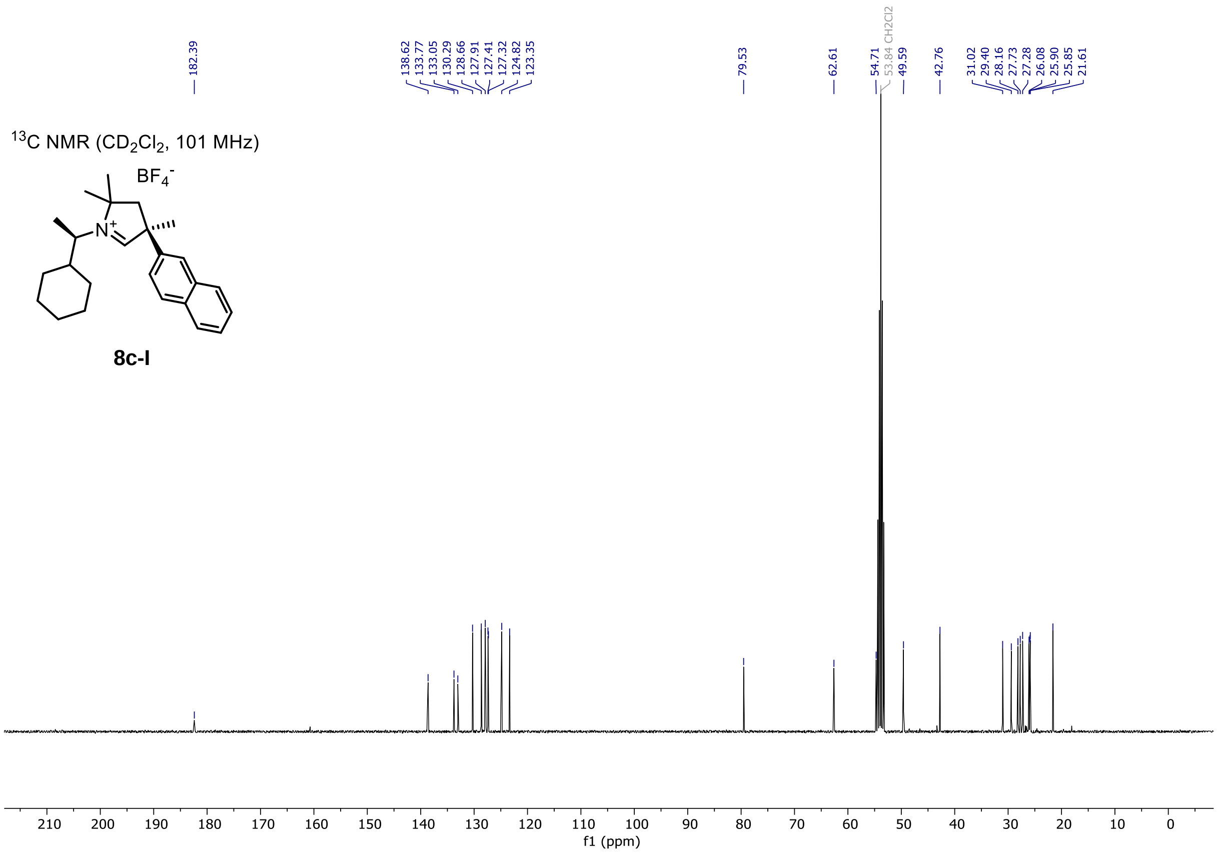
182.39
138.62 133.77 133.05 130.29 128.66 127.91 127.41 127.32 124.82 123.35
79.53
62.61
54.71 53.84 CH2Cl2 49.59
42.76
31.02 29.40 28.16 27.73 27.28 26.08 25.90 25.85 21.61
^{13}C NMR (CD_2Cl_2, 101 MHz)
BF_4^-
N+
8c-I
210 200 190 180 170 160 150 140 130 120 110 100 90 80 70 60 50 40 30 20 10 0
f1 (ppm)

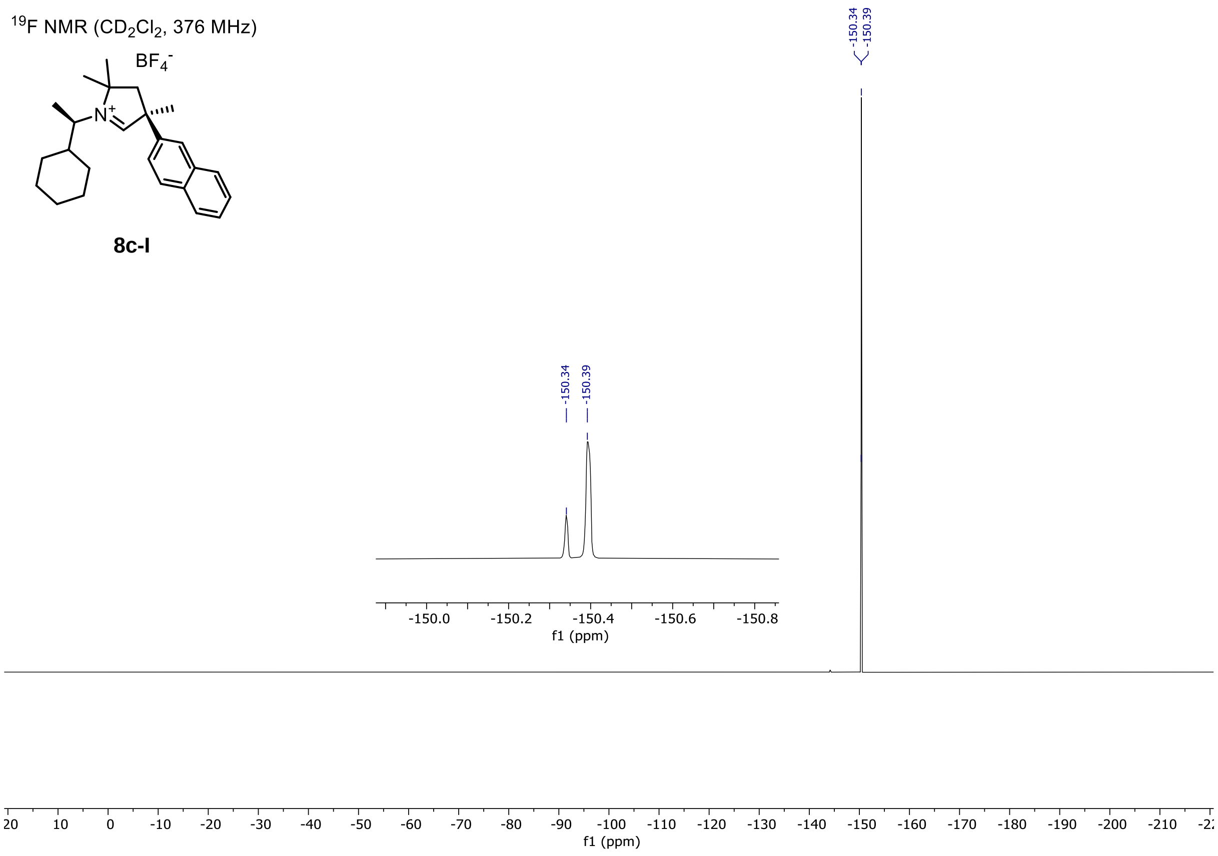

19F NMR (CD2Cl2, 376 MHz)
BF4-
8c-I
-150.34
-150.39
-150.0
-150.2
-150.4
-150.6
-150.8
f1 (ppm)
20
10
0
-10
-20
-30
-40
-50
-60
-70
-80
-90
-100
-110
-120
-130
-140
-150
-160
-170
-180
-190
-200
-210
f1 (ppm)

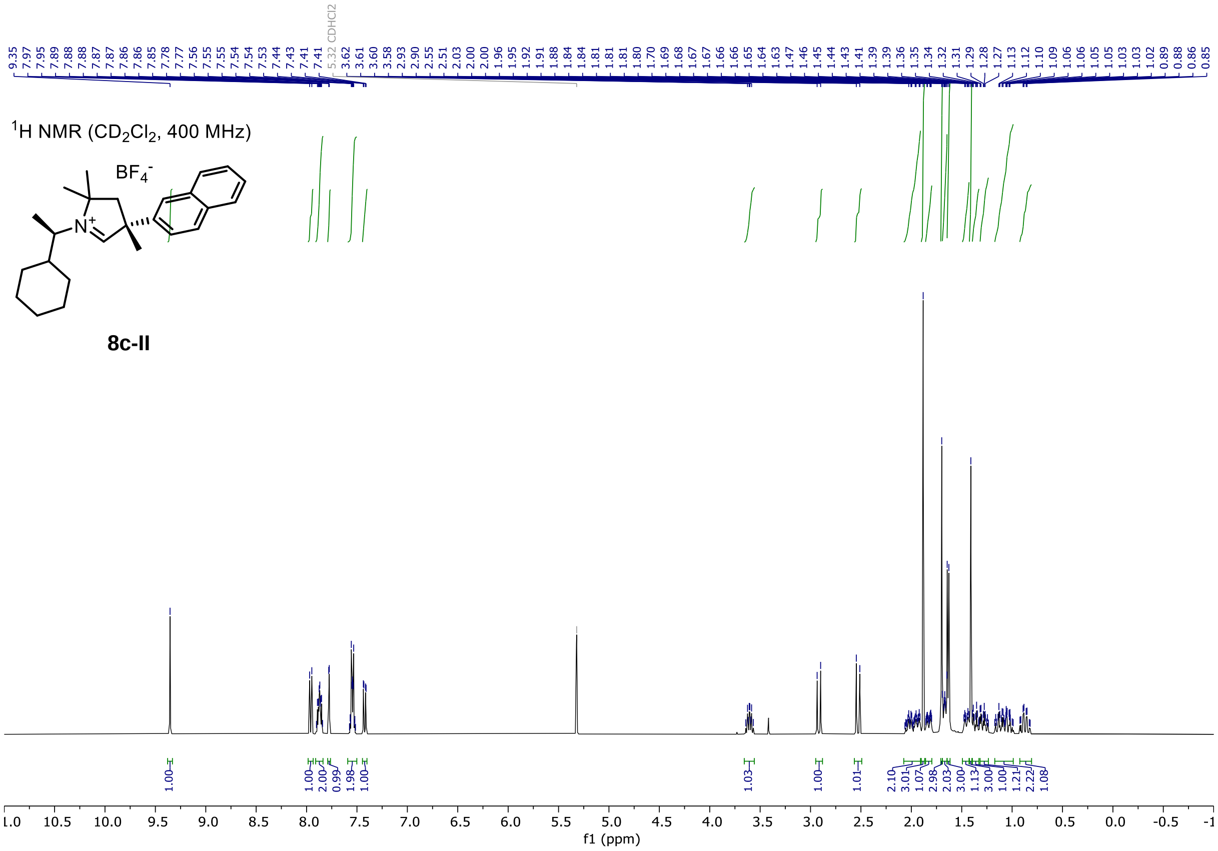
9.35 7.97 7.95 7.89 7.88 7.88 7.87 7.87 7.86 7.86 7.85 7.78 7.77 7.56 7.55 7.55 7.54 7.54 7.53 7.44 7.43 7.41 7.41 5.32 CDHCl2 3.62 3.61 3.60 3.58 2.93 2.90 2.55 2.51 2.03 2.00 1.96 1.95 1.92 1.91 1.88 1.84 1.84 1.81 1.81 1.81 1.80 1.70 1.69 1.68 1.67 1.66 1.66 1.65 1.64 1.63 1.47 1.46 1.45 1.44 1.43 1.41 1.39 1.39 1.36 1.35 1.34 1.32 1.31 1.29 1.28 1.27 1.13 1.12 1.10 1.09 1.06 1.06 1.05 1.05 1.03 1.03 1.02 0.89 0.88 0.86 0.85
1H NMR (CD2Cl2, 400 MHz)
BF4-
8c-II
1.00 1.00 2.00 0.99 1.98 1.00 1.03 1.00 1.01 2.10 3.01 1.07 2.98 2.03 3.00 1.13 3.00 1.00 1.21 2.22 1.08
f1 (ppm)

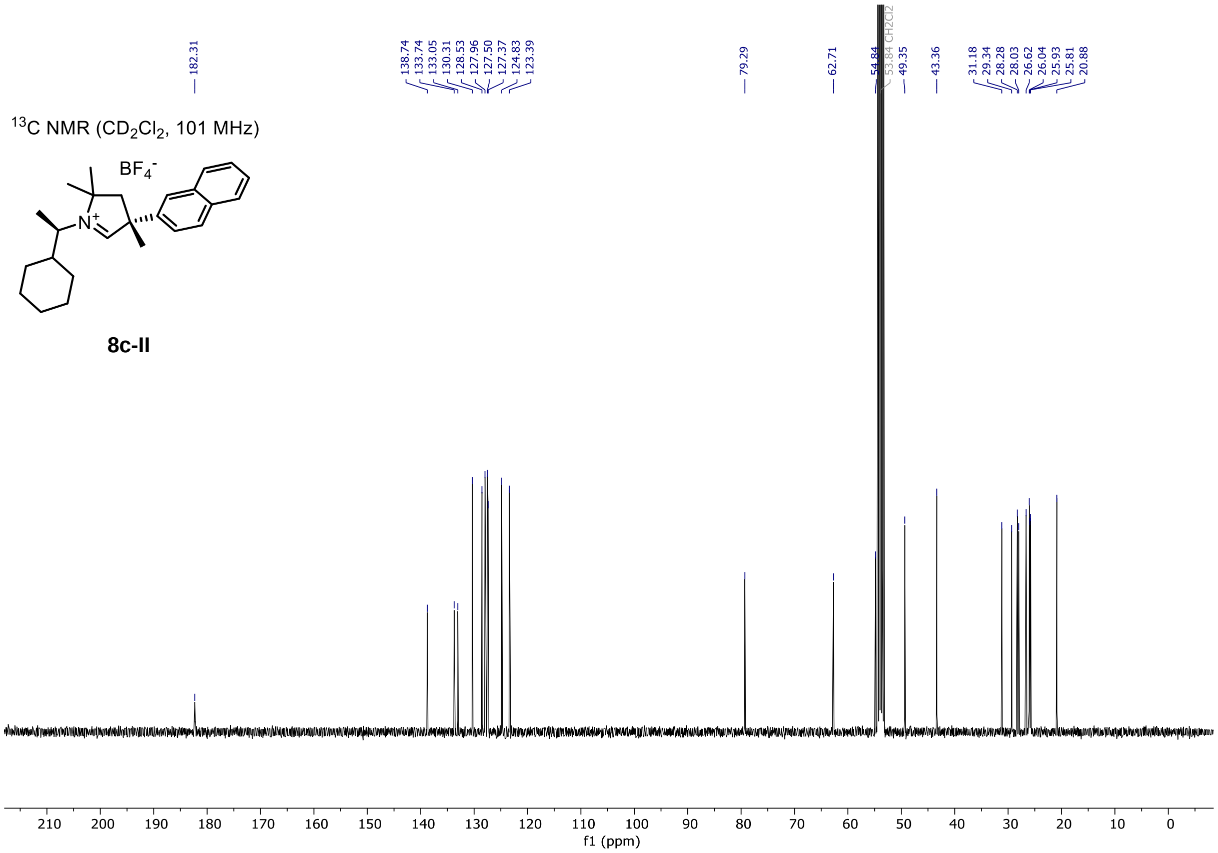
182.31 138.74 133.74 133.05 130.31 128.53 127.96 127.50 127.37 124.83 123.39 79.29 62.71 54.84 53.84 CH2Cl2 49.35 43.36 31.18 29.34 28.28 28.03 26.62 26.04 25.93 25.81 20.88
13C NMR (CD2Cl2, 101 MHz)
BF4-
8c-II
f1 (ppm)

^{19}F NMR (CD_2Cl_2, 376 MHz)

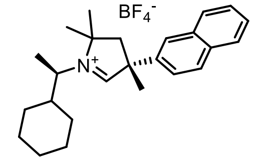

8c-II

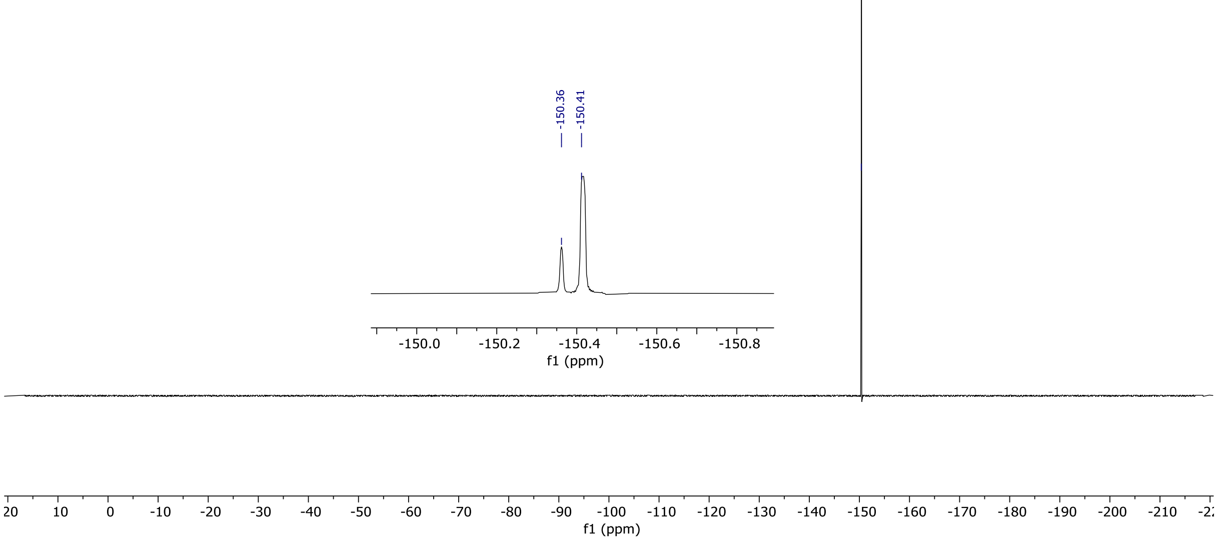

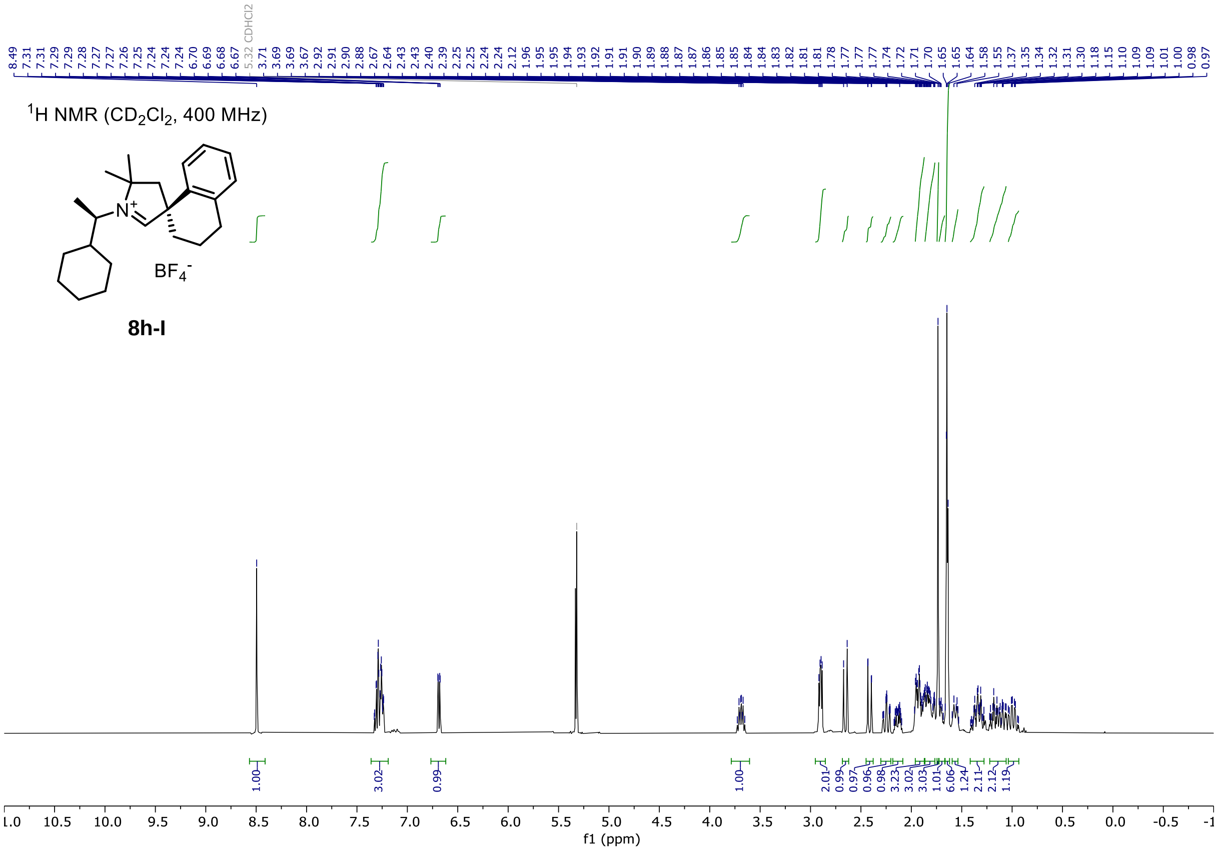
8.49 7.31 7.31 7.29 7.29 7.28 7.27 7.27 7.26 7.25 7.24 7.24 7.24 6.70 6.69 6.68 6.67 5.32 CDHCl2 3.71 3.69 3.69 3.67 2.92 2.91 2.90 2.88 2.67 2.64 2.43 2.43 2.40 2.39 2.25 2.25 2.24 2.24 2.12 1.96 1.95 1.95 1.94 1.93 1.92 1.91 1.91 1.90 1.89 1.88 1.87 1.87 1.86 1.85 1.85 1.84 1.84 1.83 1.82 1.81 1.81 1.78 1.77 1.77 1.77 1.74 1.72 1.71 1.70 1.65 1.65 1.64 1.58 1.55 1.37 1.35 1.34 1.32 1.31 1.30 1.18 1.15 1.10 1.09 1.09 1.01 1.00 0.98 0.97
^{1}H NMR (CD_2Cl_2, 400 MHz)
N+
BF_4^-
8h-I
1.00 3.02 0.99 1.00 2.01 0.99 0.97 0.96 0.98 3.23 3.02 3.03 1.01 6.06 1.24 2.11 2.12 1.19
f1 (ppm)

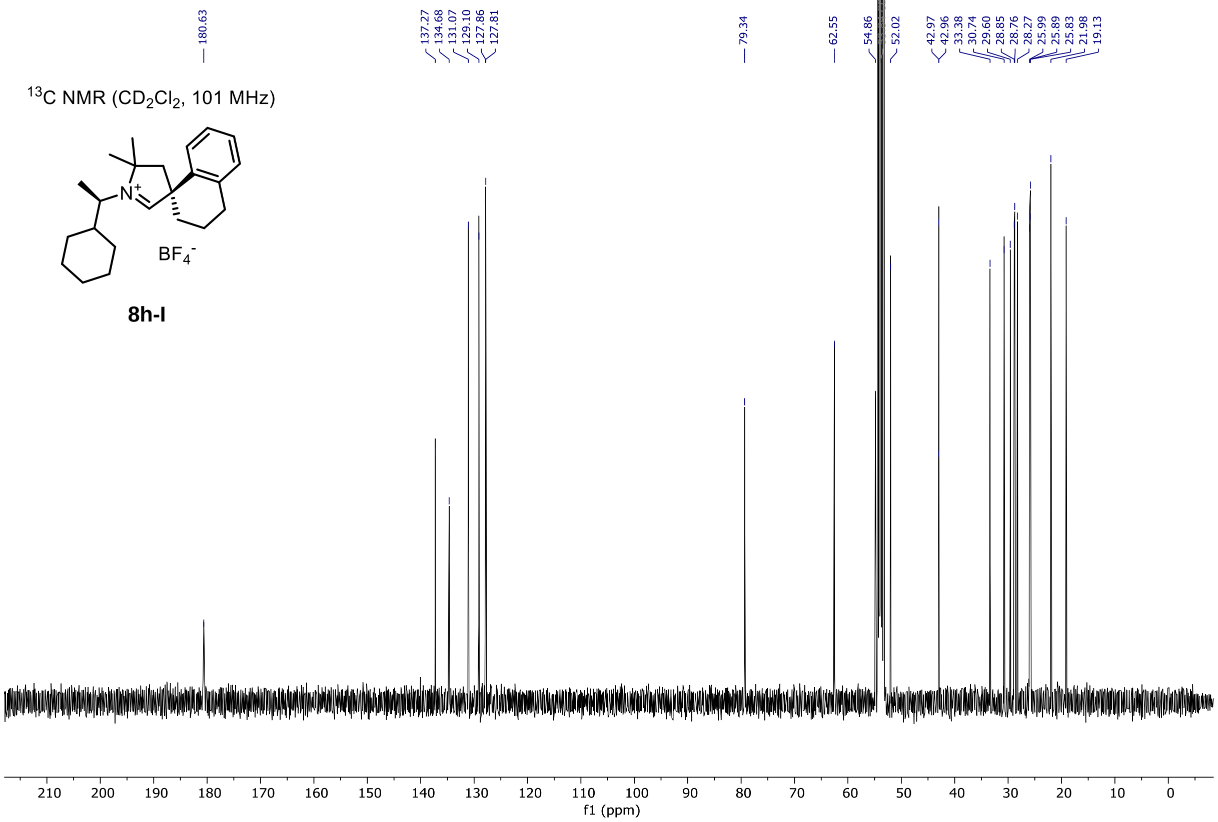
180.63
137.27 134.68 131.07 129.10 127.86 127.81
79.34
62.55
54.86
52.02
42.97 42.96 33.38 30.74 29.60 28.85 28.76 28.27 25.99 25.89 25.83 21.98 19.13
^{13}C NMR (CD_2Cl_2, 101 MHz)
N+
BF_4^-
8h-I
f1 (ppm)

^{19}F NMR (CD_2Cl_2, 376 MHz)

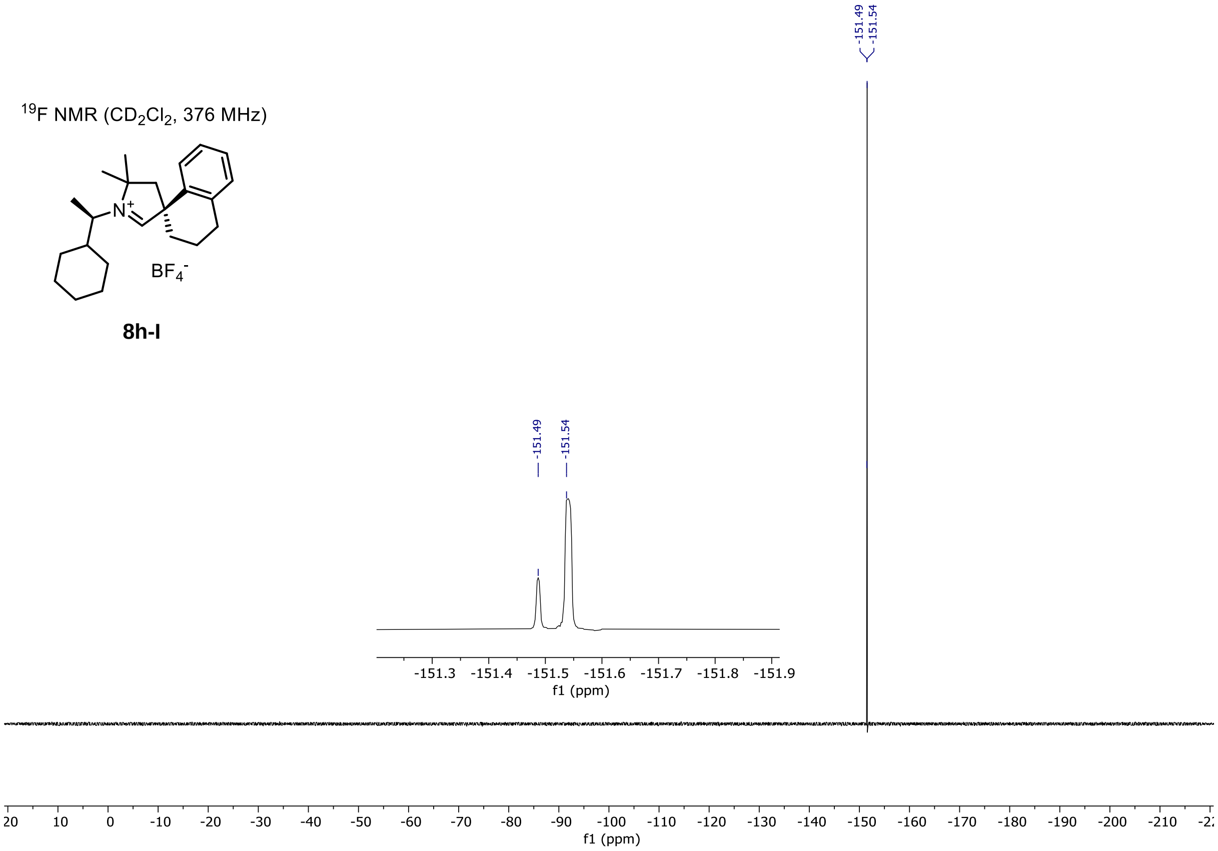

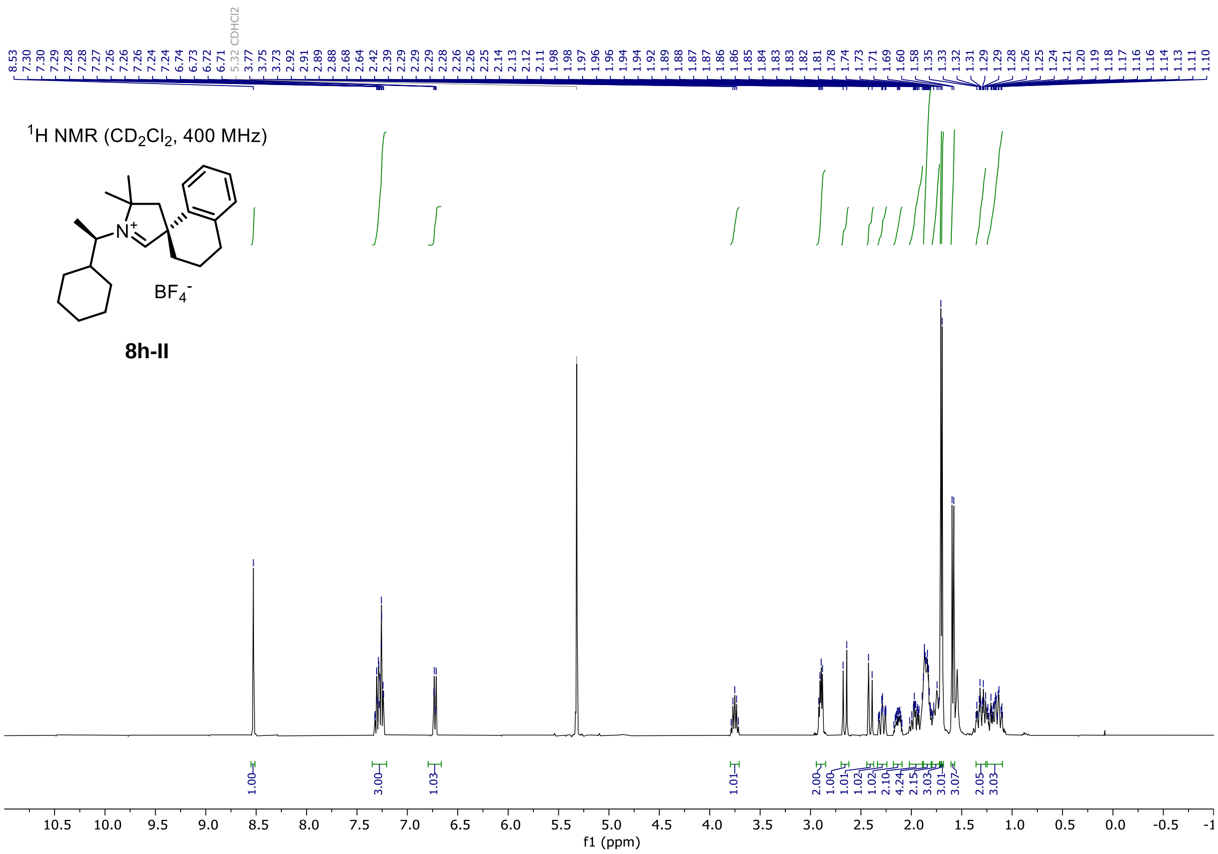
8.53 7.30 7.30 7.29 7.28 7.28 7.27 7.26 7.26 7.26 7.24 7.24 6.74 6.73 6.72 6.71 5.32 CDHCl2 3.77 3.75 3.73 2.92 2.91 2.89 2.88 2.68 2.64 2.42 2.39 2.29 2.29 2.29 2.28 2.26 2.26 2.25 2.14 2.13 2.12 2.11 1.98 1.98 1.97 1.96 1.96 1.94 1.94 1.92 1.89 1.88 1.87 1.87 1.86 1.86 1.85 1.84 1.83 1.83 1.82 1.81 1.78 1.74 1.73 1.71 1.69 1.60 1.58 1.35 1.33 1.32 1.31 1.29 1.29 1.28 1.26 1.25 1.24 1.21 1.20 1.19 1.18 1.17 1.16 1.14 1.13 1.11 1.10
^{1}H NMR (CD_2Cl_2, 400 MHz)
N+
BF_4^-
8h-II
1.00 3.00 1.03 1.01 2.00 1.00 1.01 1.02 1.02 2.10 4.24 2.15 3.03 3.01 3.07 2.05 3.03
f1 (ppm)

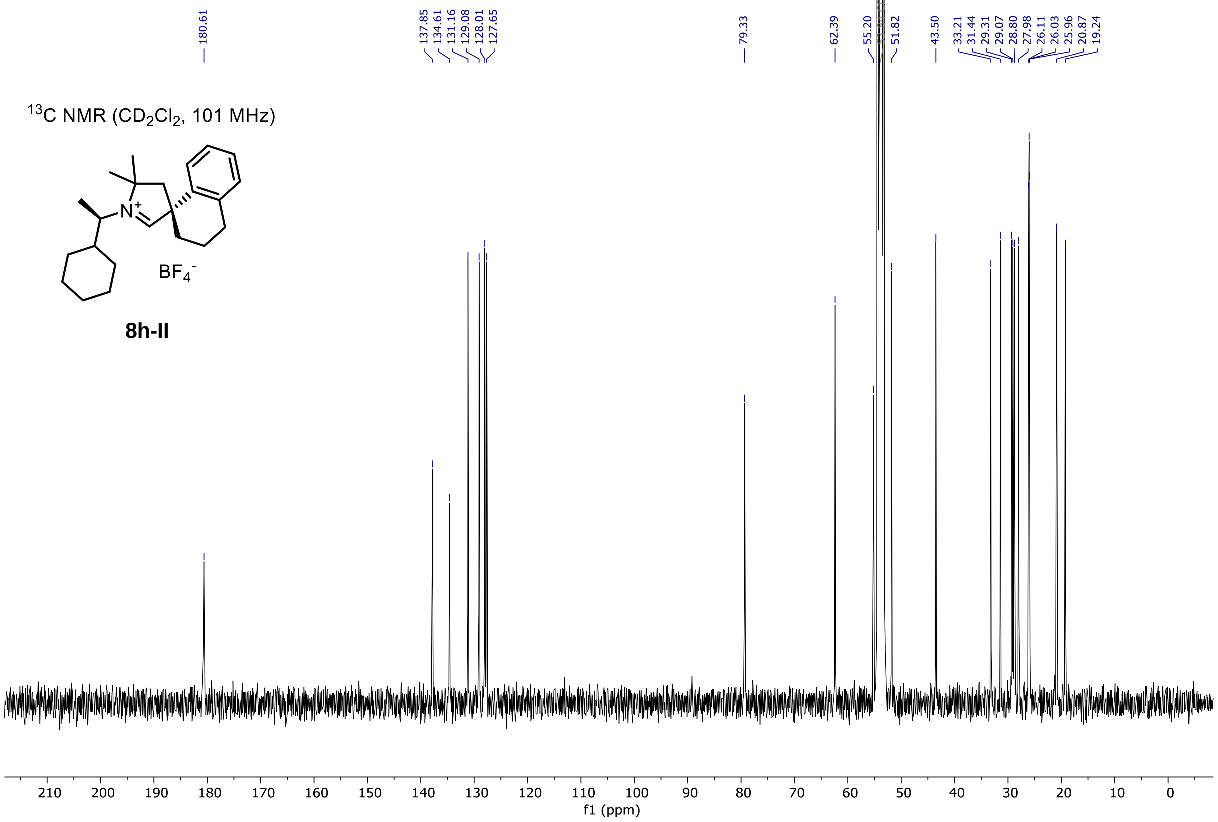
180.61 137.85 134.61 131.16 129.08 128.01 127.65 79.33 62.39 55.20 51.82 43.50 33.21 31.44 29.31 29.07 28.80 27.98 26.11 26.03 25.96 20.87 19.24
^{13}C NMR (CD_2Cl_2, 101 MHz)
N+
BF_4^-
8h-II
f1 (ppm)

^{19}F NMR (CD_2Cl_2, 376 MHz)

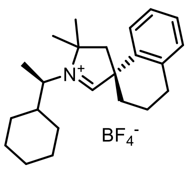

8h-II

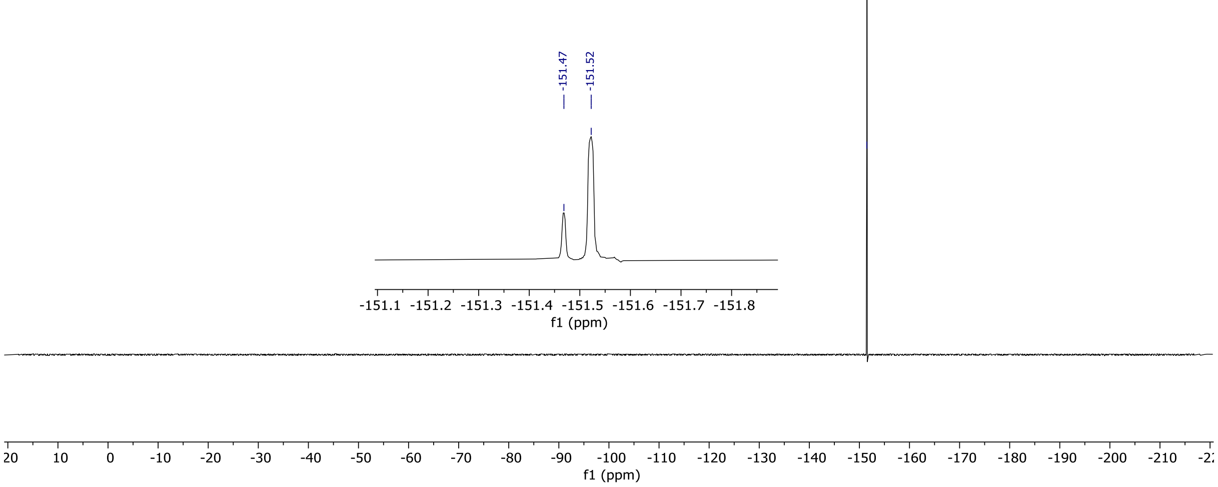

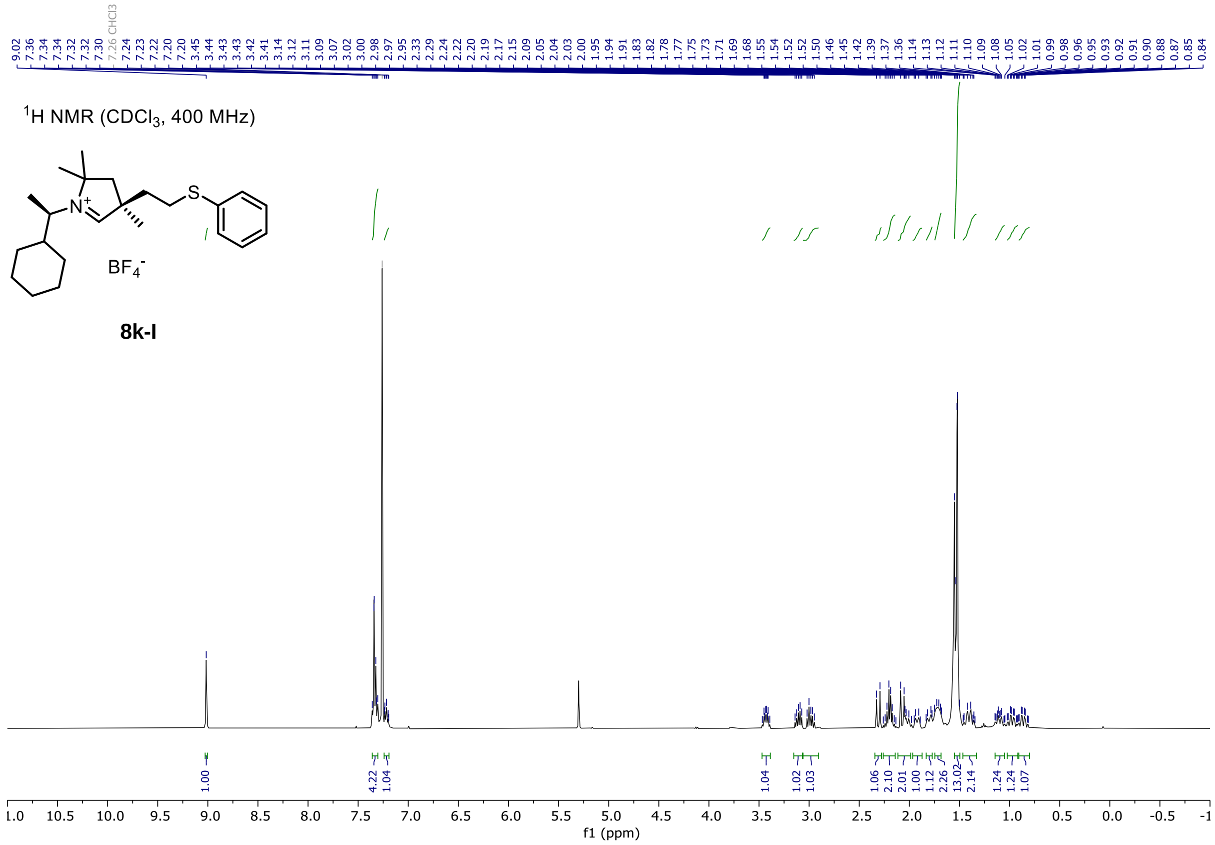
9.02 7.36 7.34 7.34 7.32 7.32 7.30 7.26 CHCl3 7.24 7.23 7.22 7.20 7.20 3.45 3.44 3.43 3.43 3.42 3.41 3.14 3.12 3.11 3.09 3.07 3.02 3.00 2.98 2.97 2.95 2.33 2.29 2.24 2.22 2.20 2.19 2.17 2.15 2.09 2.05 2.04 2.03 2.00 1.95 1.94 1.91 1.83 1.82 1.78 1.77 1.75 1.73 1.71 1.69 1.68 1.55 1.54 1.52 1.52 1.50 1.46 1.45 1.42 1.39 1.37 1.36 1.14 1.13 1.12 1.12 1.11 1.10 1.09 1.08 1.05 1.02 1.01 0.99 0.98 0.96 0.95 0.93 0.92 0.91 0.90 0.88 0.87 0.85 0.84
^{1}H NMR ($CDCl_3$, 400 MHz)
S
N+
BF_4^-
8k-I
1.00 4.22 1.04 1.04 1.02 1.03 1.06 2.10 2.01 1.00 1.12 2.26 13.02 2.14 1.24 1.24 1.07
f1 (ppm)

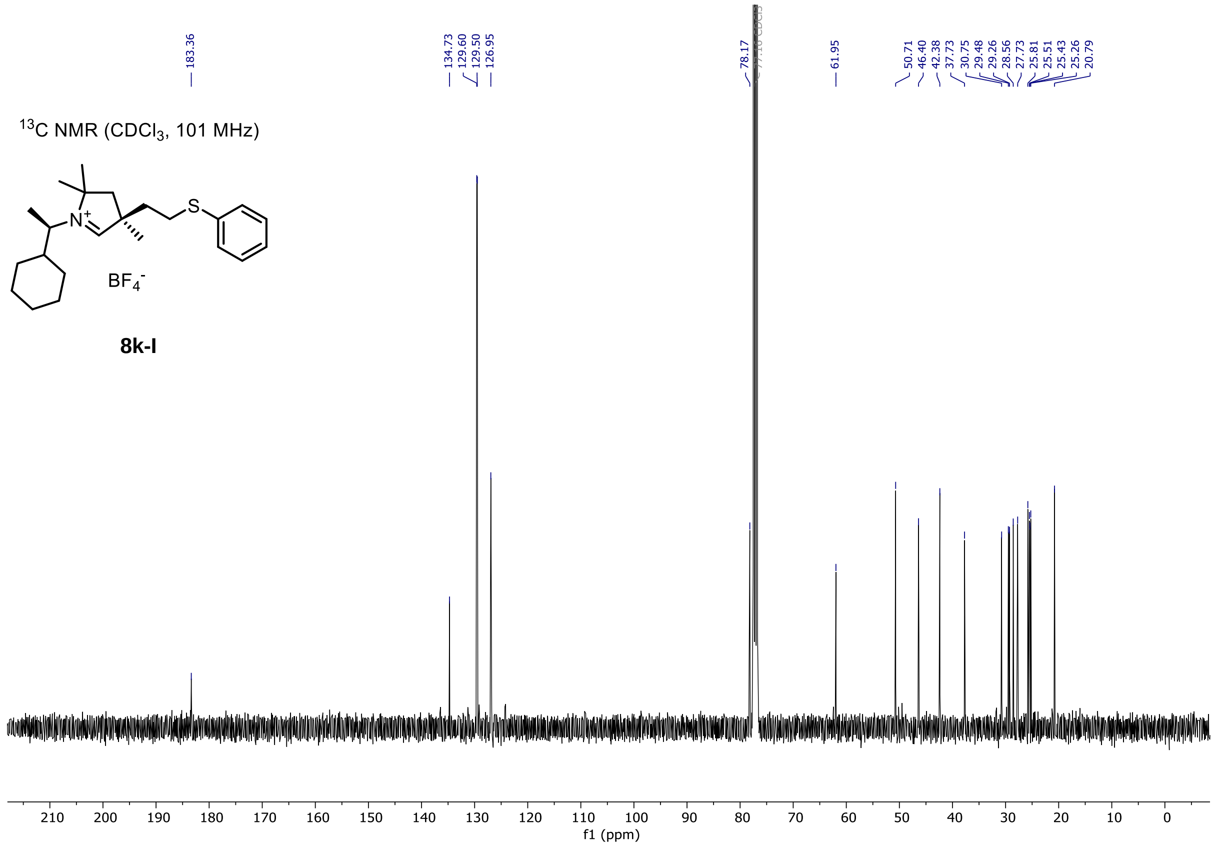
183.36 134.73 129.60 129.50 126.95 78.17 77.16 CDCl3 61.95 50.71 46.40 42.38 37.73 30.75 29.48 29.26 28.56 27.73 25.81 25.51 25.43 25.26 20.79
^{13}C NMR ($CDCl_3$, 101 MHz)
S
N+
BF_4^-
8k-I
f1 (ppm)

^{19}F NMR ($CDCl_3$, 376 MHz)

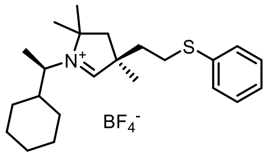

8k-I

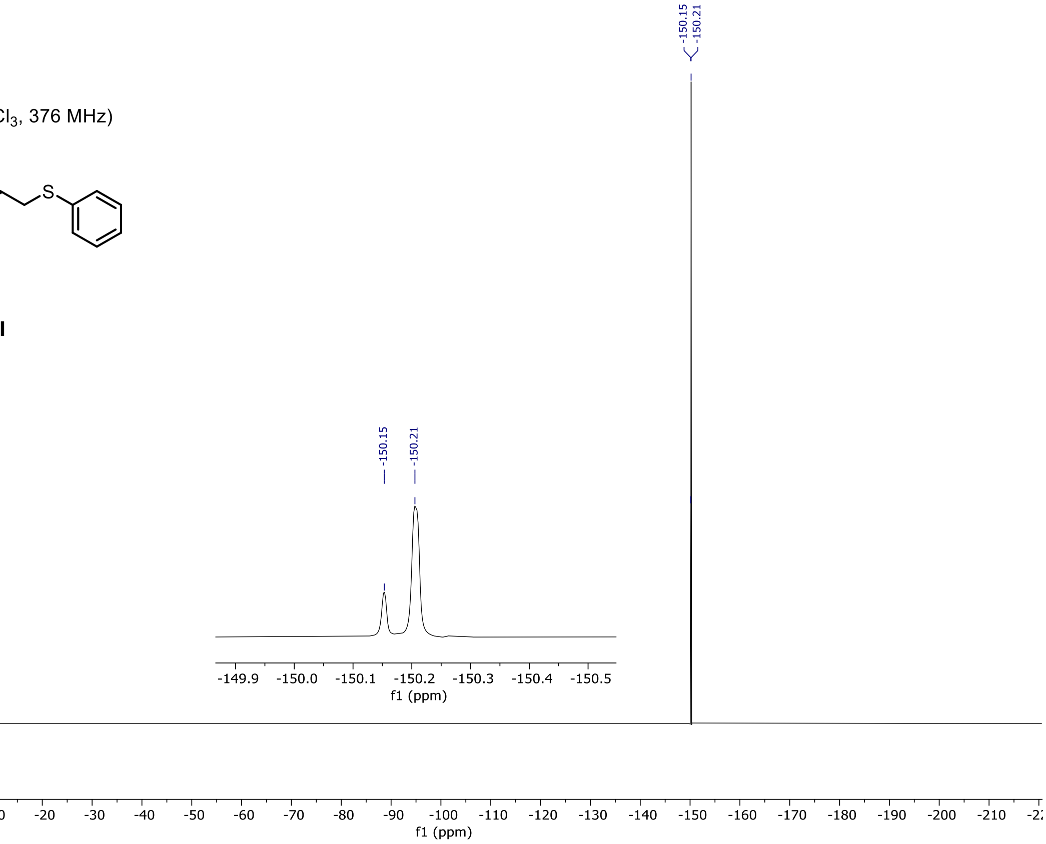

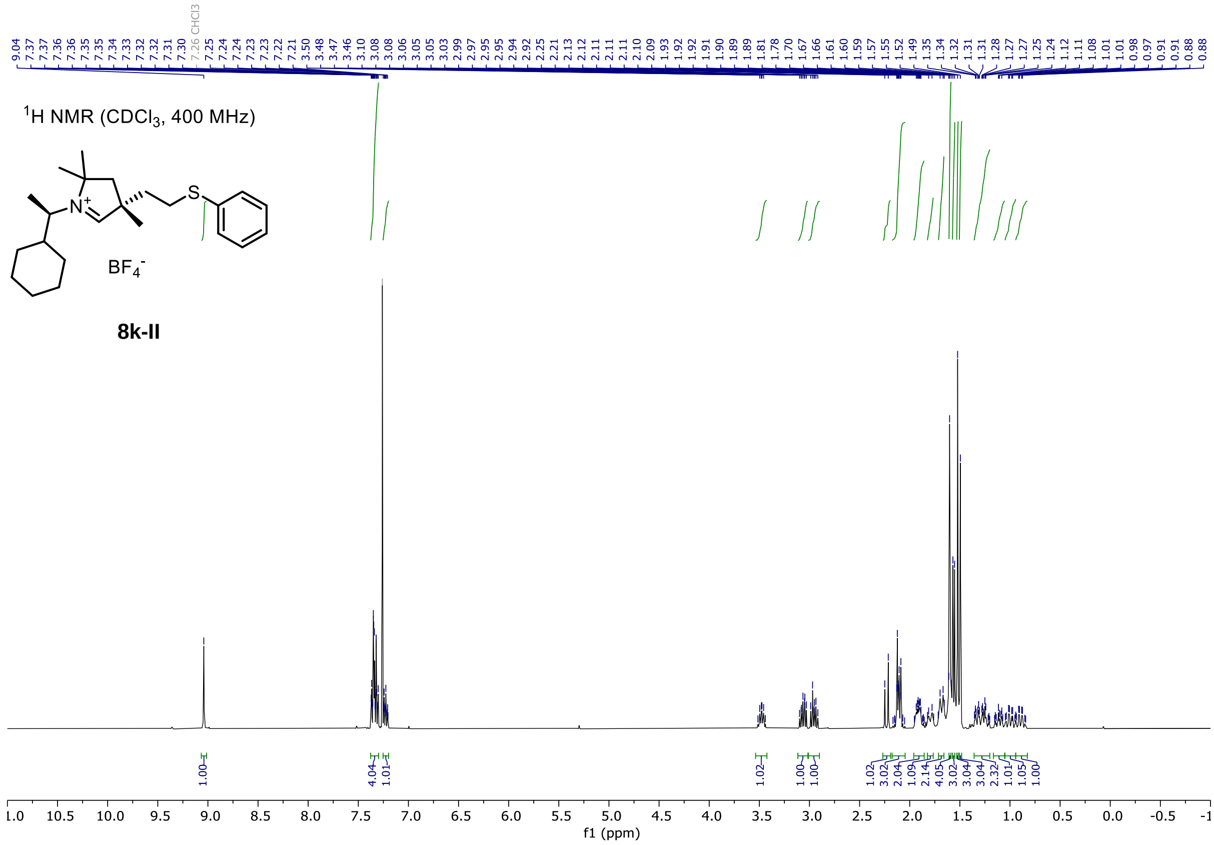

1H NMR (CDCl3, 400 MHz)
BF4-
8k-II
f1 (ppm)

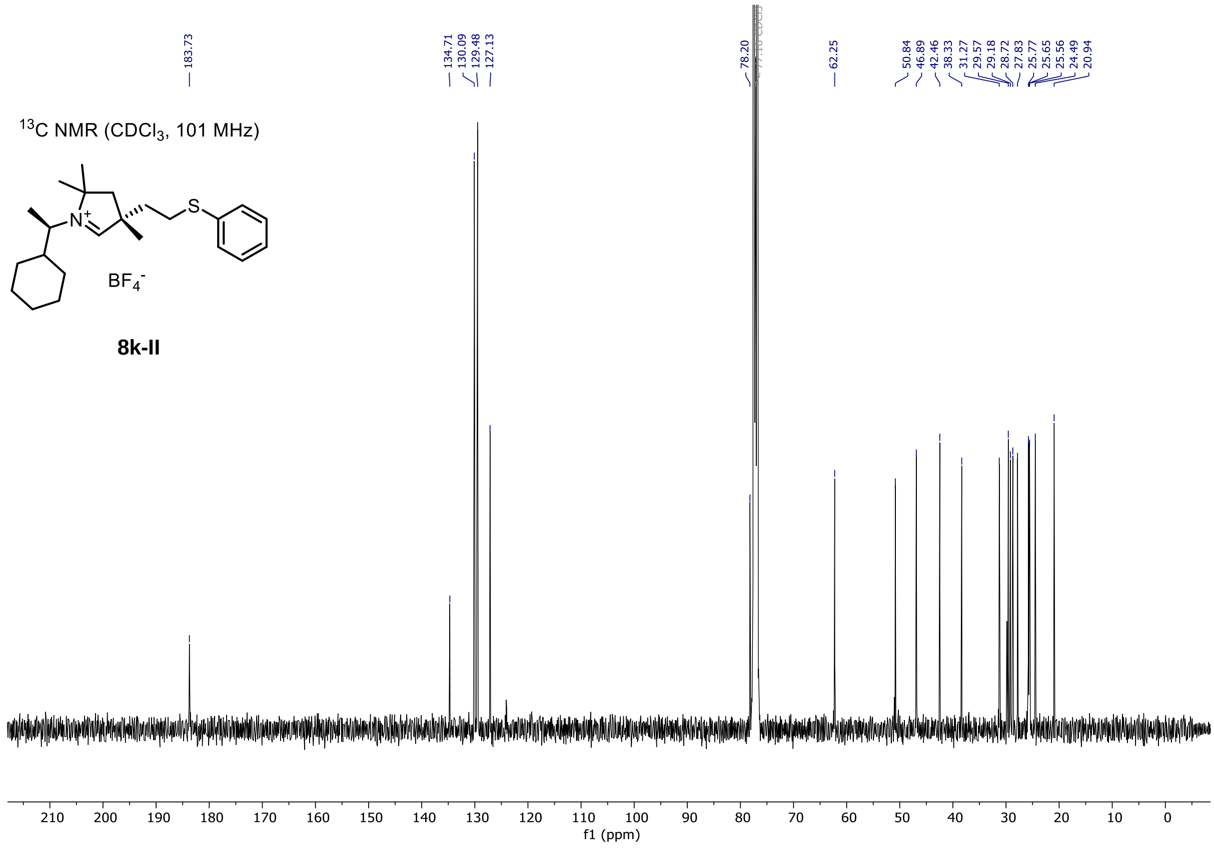

13C NMR (CDCl3, 101 MHz)
BF4-
8k-II
f1 (ppm)

^{19}F NMR ($CDCl_3$, 376 MHz)

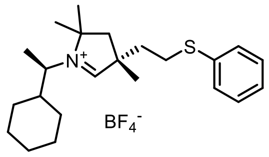

BF_4^-

8k-II

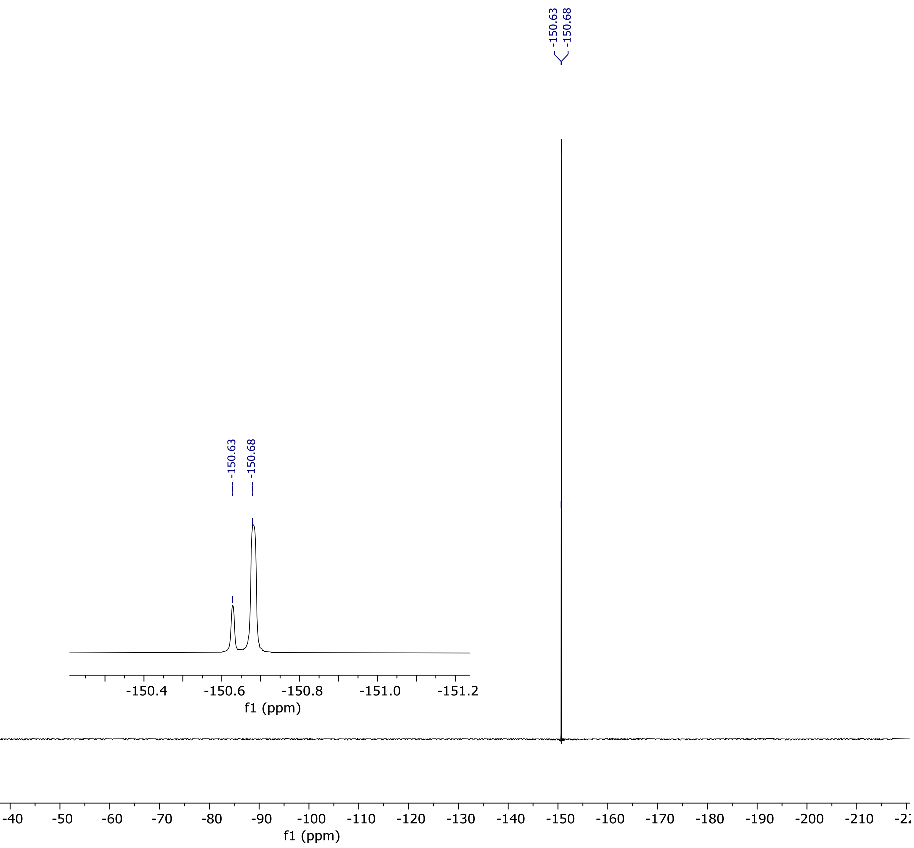

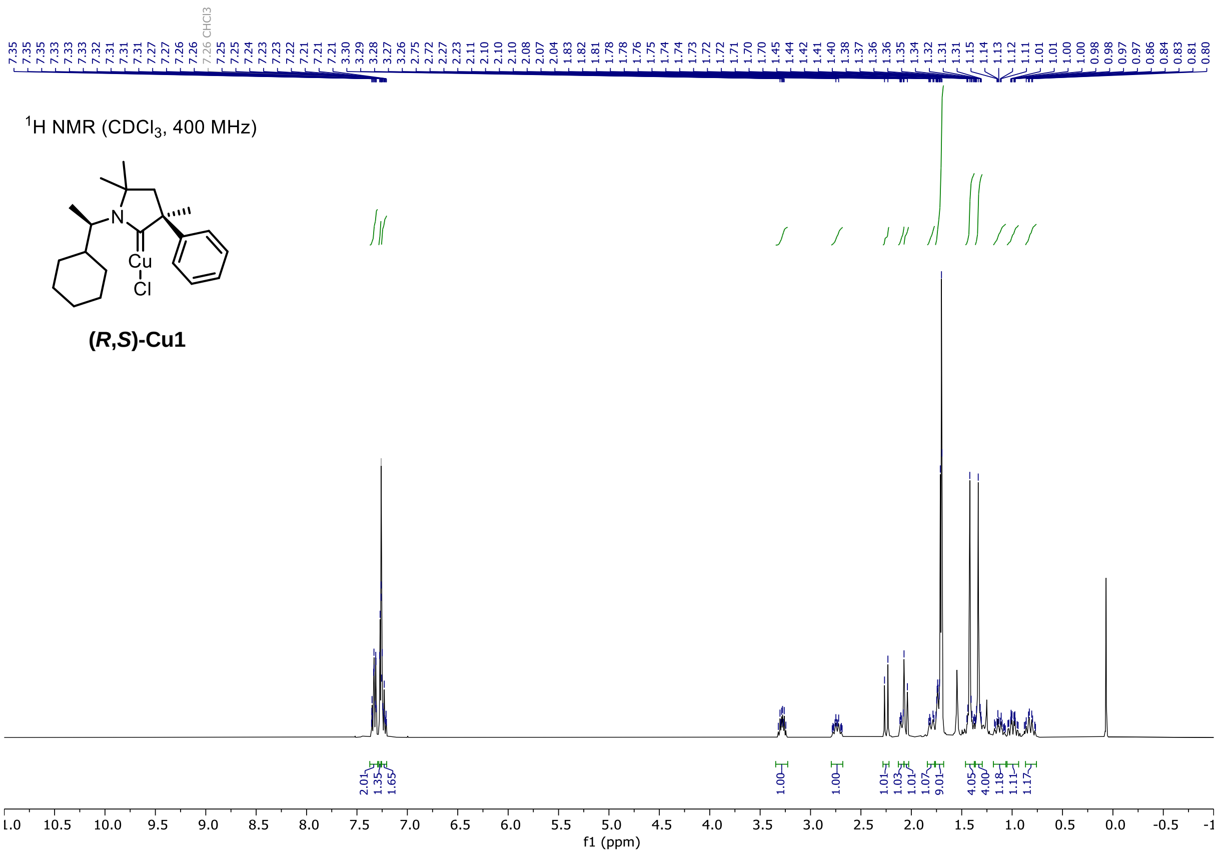

1H NMR (CDCl3, 400 MHz)
(R,S)-Cu1
Cu
Cl
N
f1 (ppm)

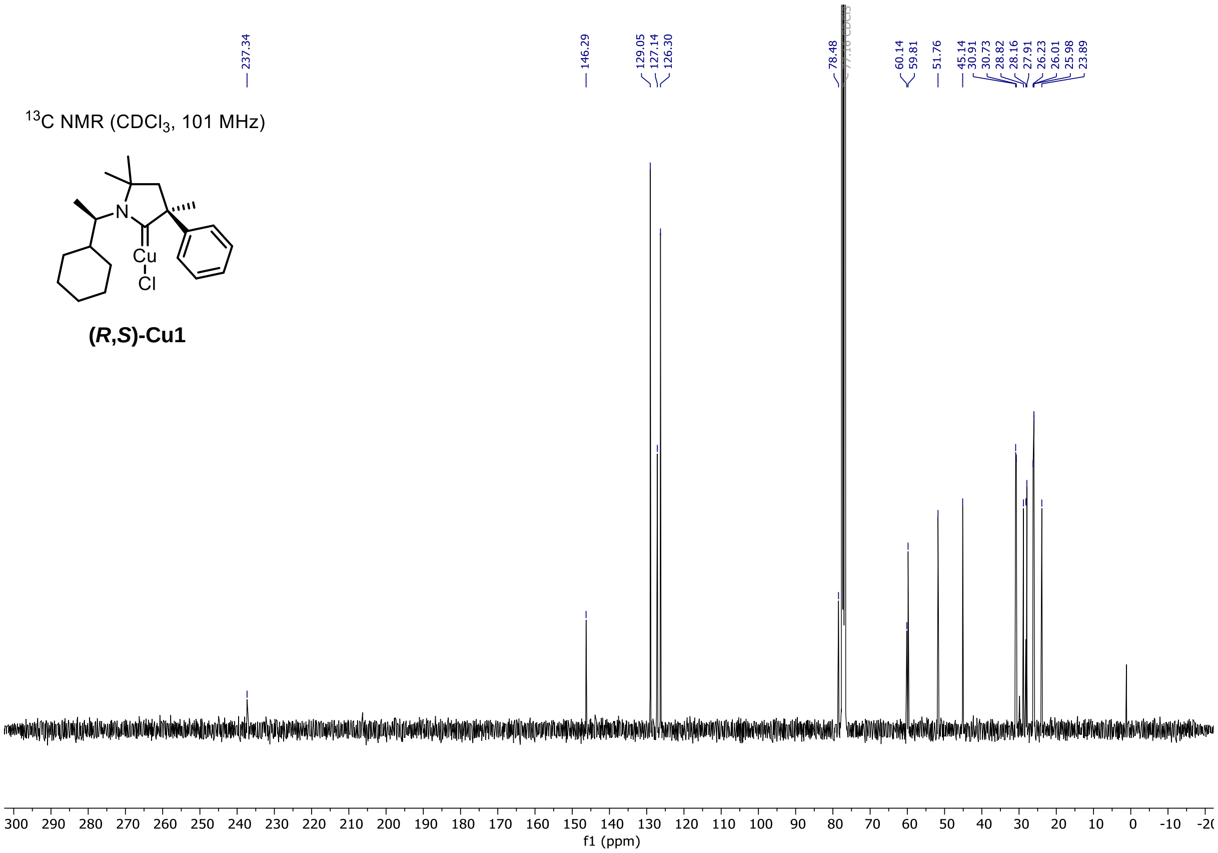

13C NMR (CDCl3, 101 MHz)
(R,S)-Cu1
Cu
Cl
N
237.34
146.29
129.05
127.14
126.30
78.48
60.14
59.81
51.76
45.14
30.91
30.73
28.82
28.16
27.91
26.23
26.01
25.98
23.89
f1 (ppm)

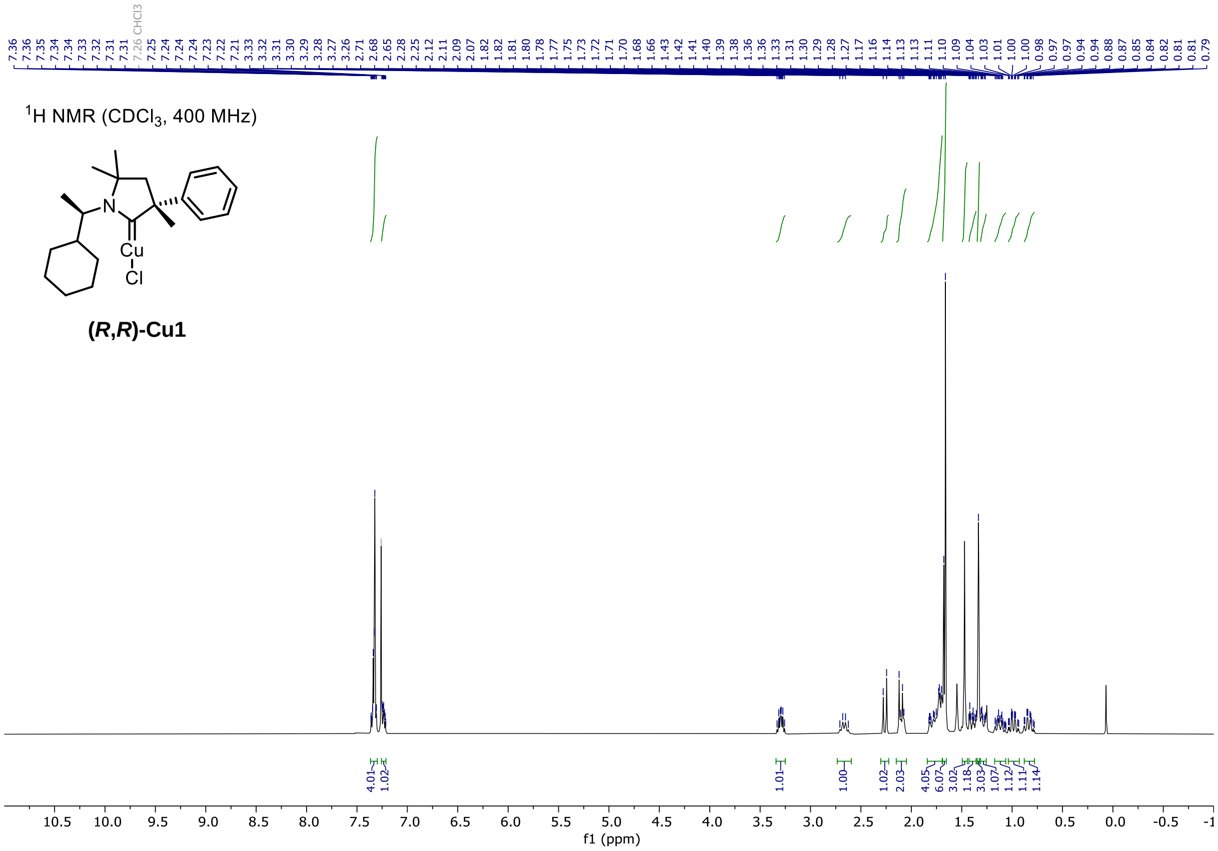
7.36 7.36 7.35 7.34 7.33 7.32 7.31 7.31 7.26 CHCl3 7.25 7.24 7.24 7.24 7.23 7.22 7.21 3.33 3.32 3.31 3.30 3.29 3.28 3.27 3.26 2.71 2.68 2.65 2.28 2.25 2.12 2.11 2.09 2.07 1.82 1.81 1.80 1.78 1.77 1.75 1.73 1.72 1.71 1.70 1.68 1.66 1.43 1.42 1.41 1.40 1.39 1.38 1.36 1.36 1.33 1.31 1.30 1.29 1.28 1.27 1.17 1.16 1.14 1.13 1.13 1.11 1.10 1.09 1.04 1.03 1.01 1.00 1.00 0.98 0.97 0.97 0.94 0.94 0.88 0.87 0.85 0.84 0.82 0.81 0.81 0.79
¹H NMR (CDCl3, 400 MHz)
N
Cu
Cl
(R,R)-Cu1
4.01 1.02 1.01 1.00 1.02 2.03 4.05 6.07 3.02 1.18 3.03 1.07 1.12 1.11 1.14
10.5 10.0 9.5 9.0 8.5 8.0 7.5 7.0 6.5 6.0 5.5 5.0 4.5 4.0 3.5 3.0 2.5 2.0 1.5 1.0 0.5 0.0 -0.5 -1
f1 (ppm)

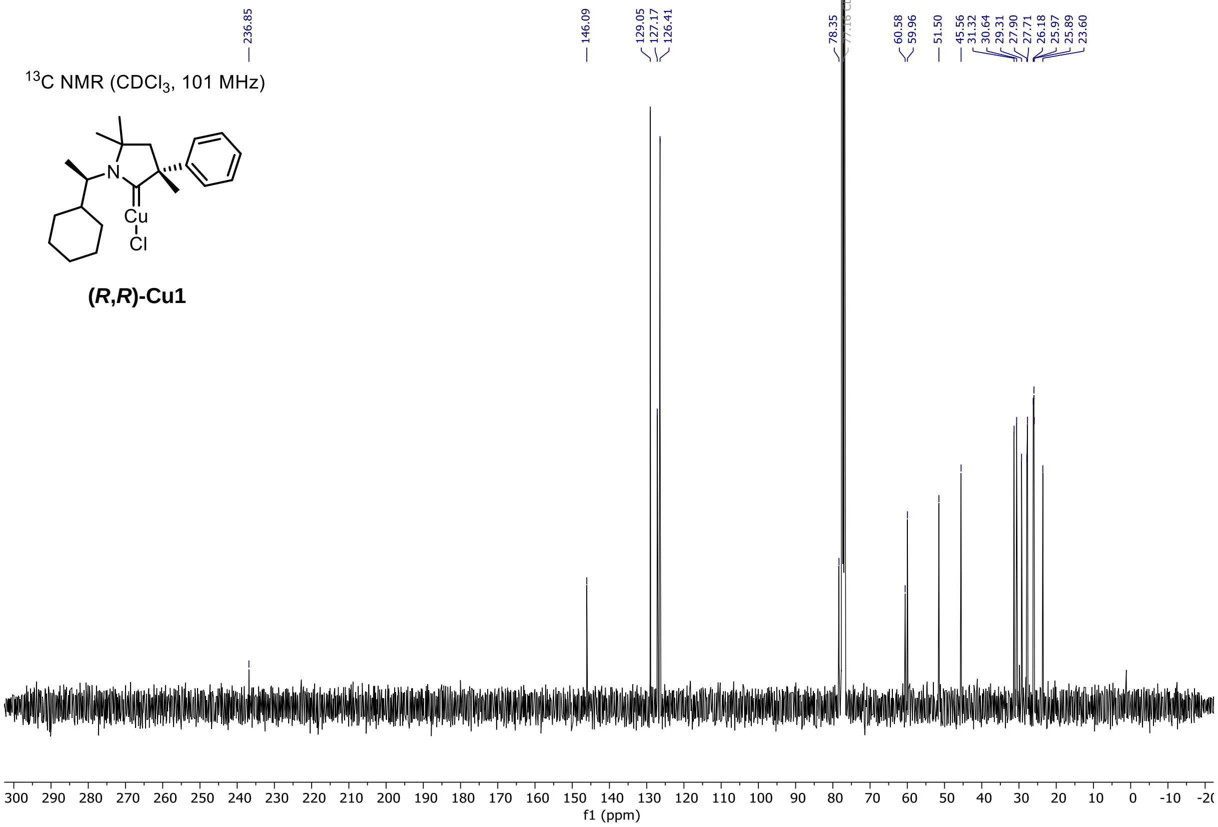
236.85 146.09 129.05 127.17 126.41 78.35 77.16 CDCl3 60.58 59.96 51.50 45.56 31.32 30.64 29.31 27.90 27.71 26.18 25.97 25.89 23.60
¹³C NMR (CDCl3, 101 MHz)
N
Cu
Cl
(R,R)-Cu1
300 290 280 270 260 250 240 230 220 210 200 190 180 170 160 150 140 130 120 110 100 90 80 70 60 50 40 30 20 10 0 -10 -20
f1 (ppm)

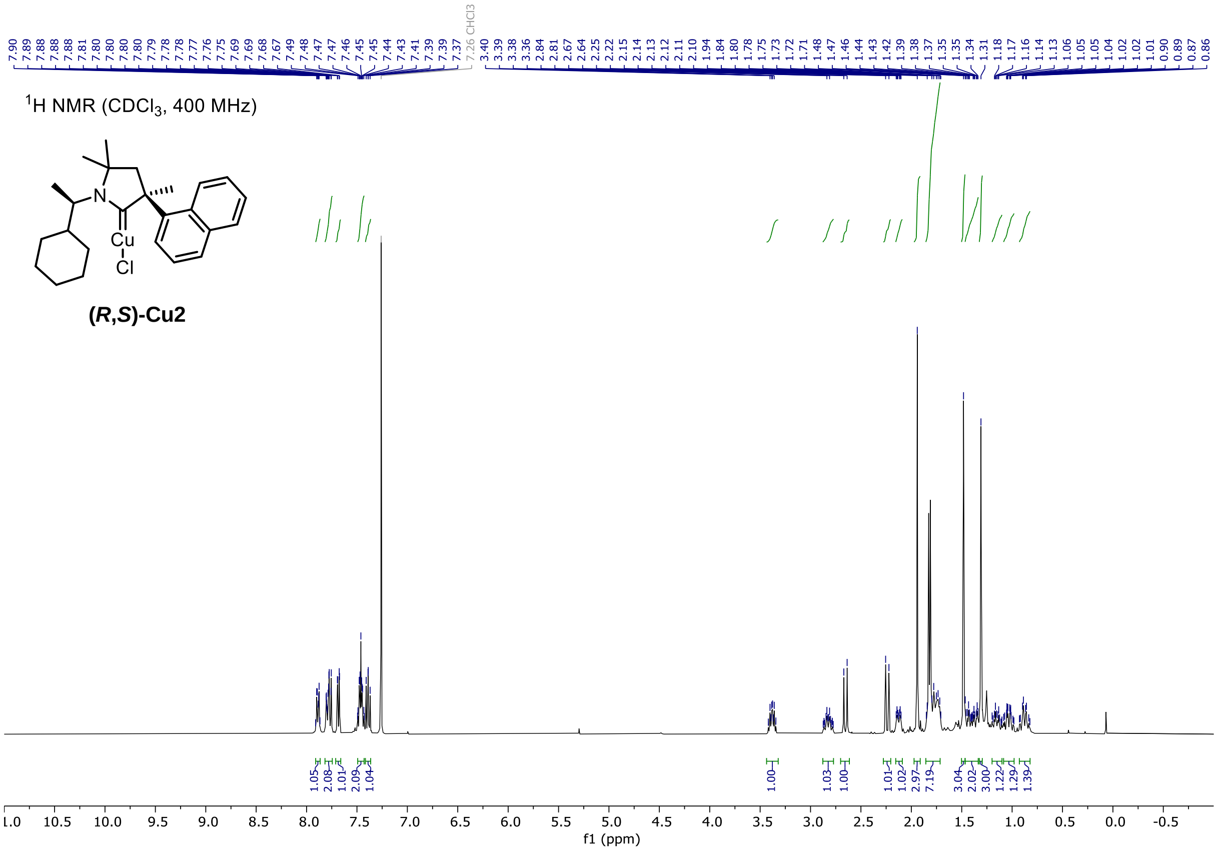

7.90 7.89 7.88 7.88 7.81 7.80 7.80 7.80 7.79 7.78 7.78 7.77 7.76 7.75 7.69 7.69 7.68 7.67 7.49 7.48 7.47 7.47 7.46 7.45 7.45 7.44 7.43 7.41 7.39 7.39 7.37
7.26 CHCl3
3.40 3.39 3.38 3.36 2.84 2.81 2.67 2.64 2.25 2.22 2.15 2.14 2.13 2.12 2.11 2.10 1.94 1.84 1.80 1.78 1.75 1.73 1.72 1.71 1.48 1.47 1.46 1.44 1.43 1.42 1.39 1.38 1.37 1.35 1.35 1.34 1.31 1.18 1.17 1.16 1.14 1.13 1.06 1.05 1.05 1.04 1.02 1.01 0.90 0.89 0.87 0.86
1H NMR (CDCl3, 400 MHz)
N
Cu
Cl
(R,S)-Cu2
1.05 2.08 1.01 2.09 1.04 1.00 1.03 1.00 1.01 1.02 2.97 7.19 3.04 2.02 3.00 1.22 1.29 1.39
f1 (ppm)

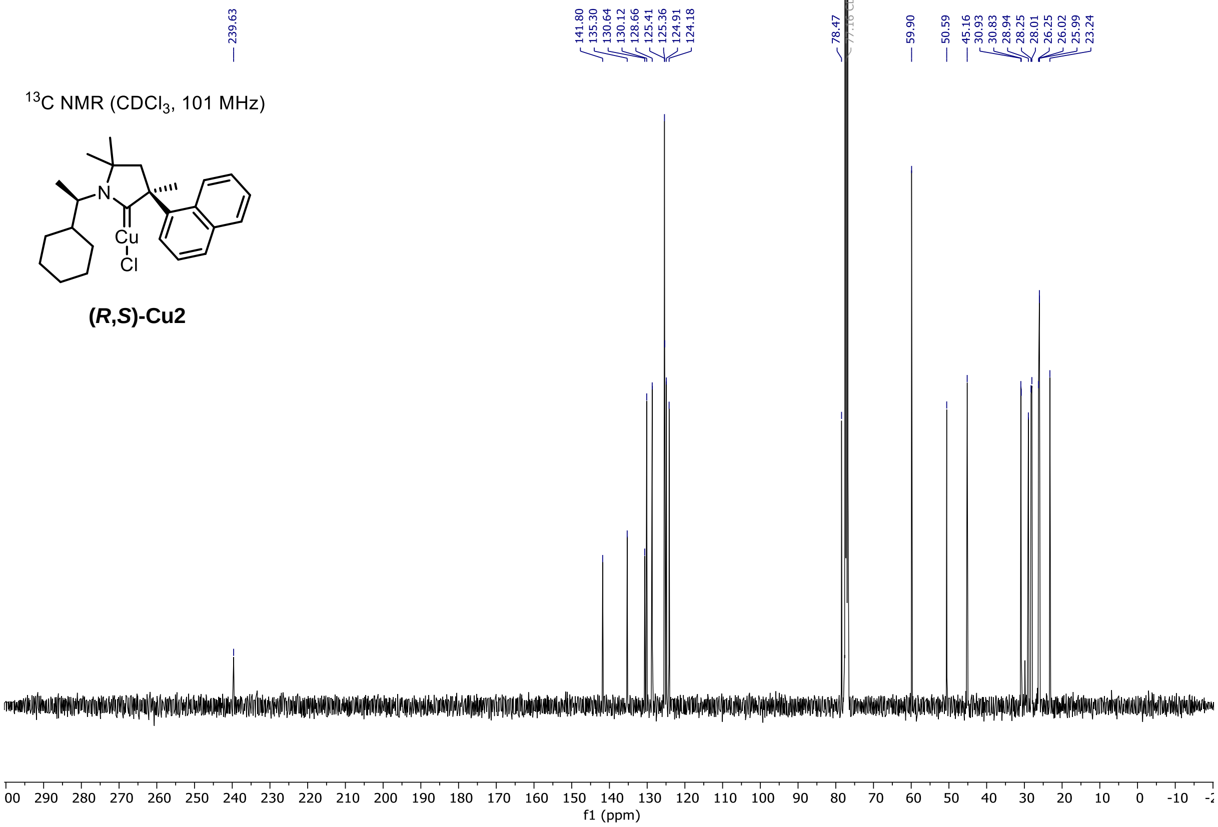

239.63
141.80 135.30 130.64 130.12 128.66 125.41 125.36 124.91 124.18
78.47
77.16 CDCl3
59.90
50.59
45.16 30.93 30.83 28.94 28.25 28.01 26.25 26.02 25.99 23.24
13C NMR (CDCl3, 101 MHz)
N
Cu
Cl
(R,S)-Cu2
f1 (ppm)

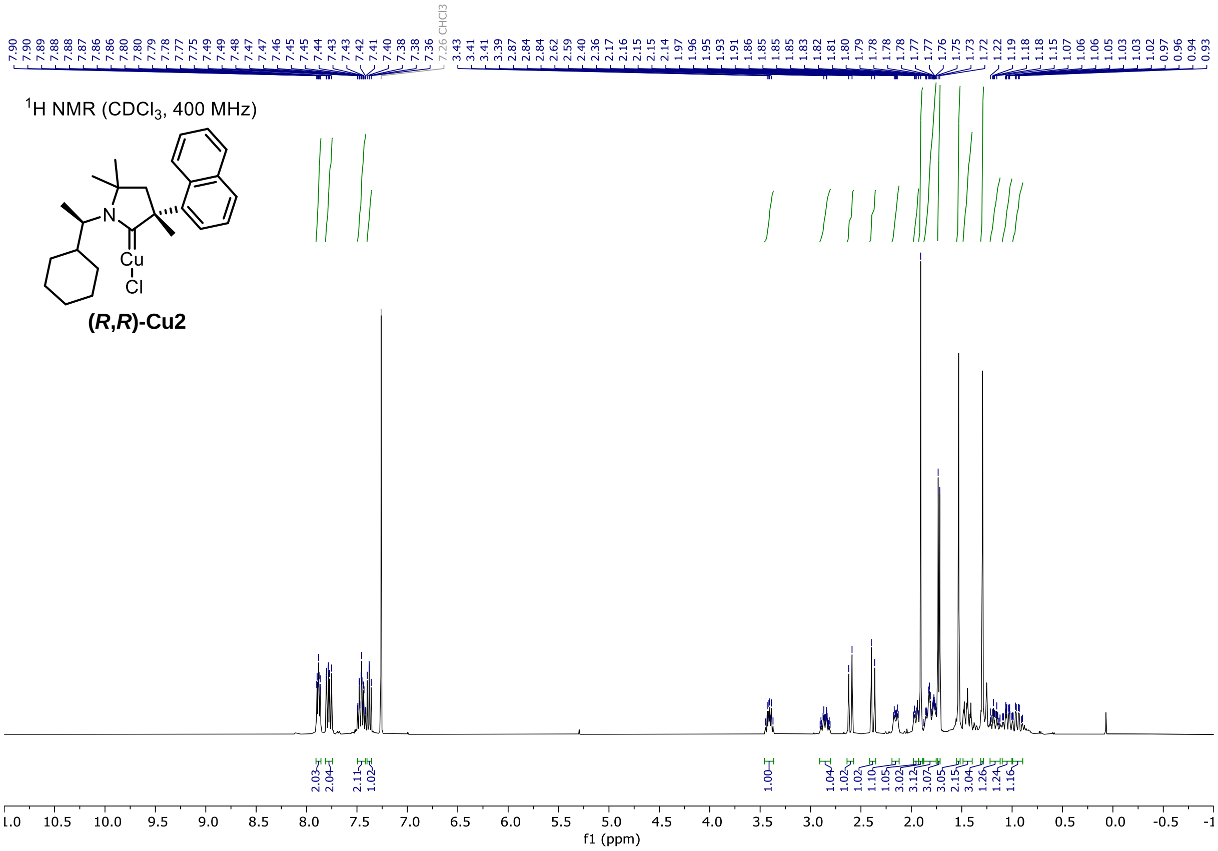
7.90 7.90 7.89 7.88 7.88 7.87 7.86 7.86 7.80 7.80 7.79 7.78 7.77 7.75 7.49 7.49 7.48 7.47 7.47 7.46 7.45 7.45 7.44 7.43 7.43 7.42 7.41 7.40 7.38 7.38 7.36 7.26 CHCl3 3.43 3.41 3.39 2.87 2.84 2.84 2.62 2.59 2.40 2.36 2.17 2.16 2.15 2.15 2.14 1.97 1.96 1.95 1.93 1.91 1.86 1.85 1.85 1.85 1.83 1.82 1.81 1.80 1.79 1.78 1.78 1.78 1.77 1.77 1.76 1.75 1.73 1.72 1.22 1.19 1.18 1.18 1.15 1.07 1.06 1.05 1.03 1.03 1.02 0.97 0.96 0.94 0.93
^{1}H NMR ($CDCl_3$, 400 MHz)
N
Cu
Cl
(*R*,*R*)-Cu2
2.03 2.04 2.11 1.02 1.00 1.04 1.02 1.02 1.10 1.05 3.02 3.12 3.07 3.05 2.15 3.04 1.26 1.24 1.16
f1 (ppm)

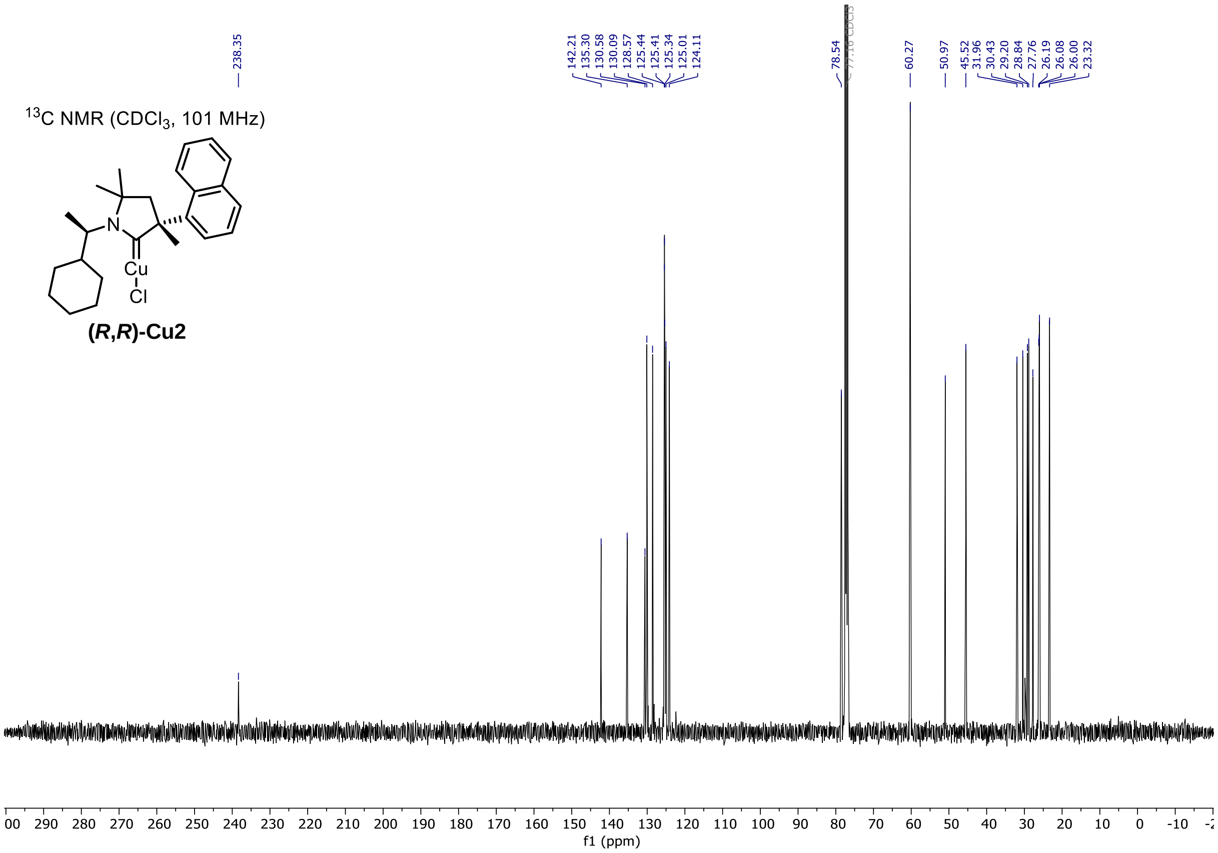
238.35
142.21 135.30 130.58 130.09 128.57 125.44 125.41 125.34 125.01 124.11
78.54 77.16 CDCl3
60.27 50.97 45.52 31.96 30.43 29.20 28.84 27.76 26.19 26.08 26.00 23.32
^{13}C NMR ($CDCl_3$, 101 MHz)
N
Cu
Cl
(*R*,*R*)-Cu2
f1 (ppm)

^{1}H NMR ($CDCl_3$, 400 MHz)

(*R*,*S*)-Cu3

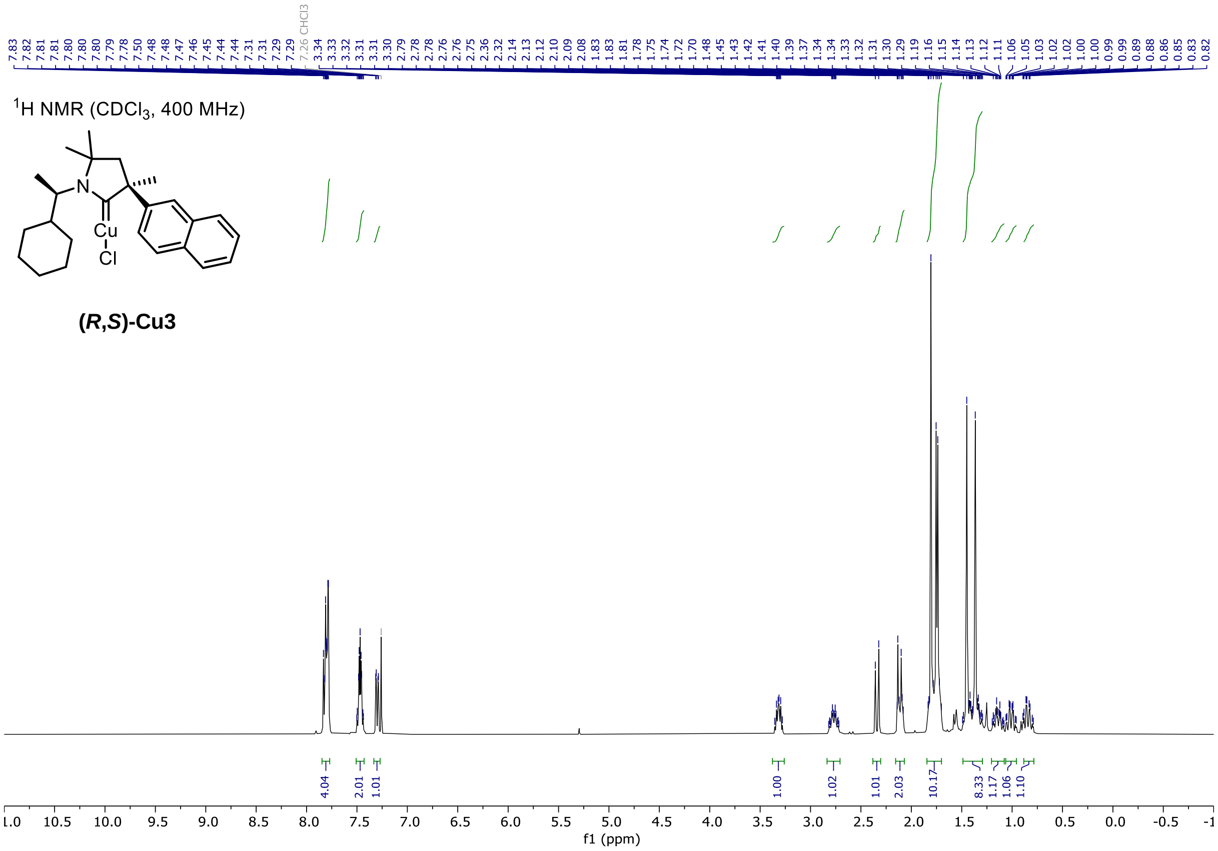

^{13}C NMR ($CDCl_3$, 101 MHz)

(*R*,*S*)-Cu3

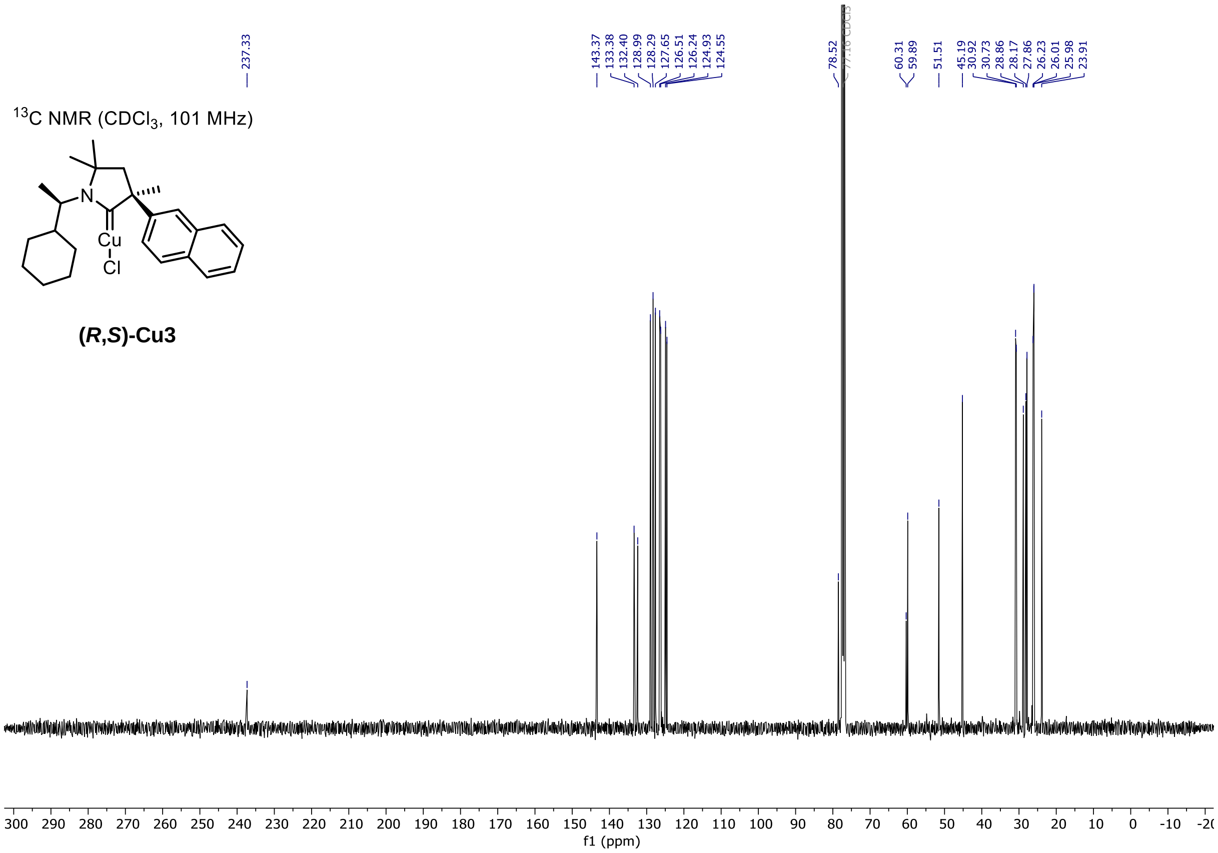

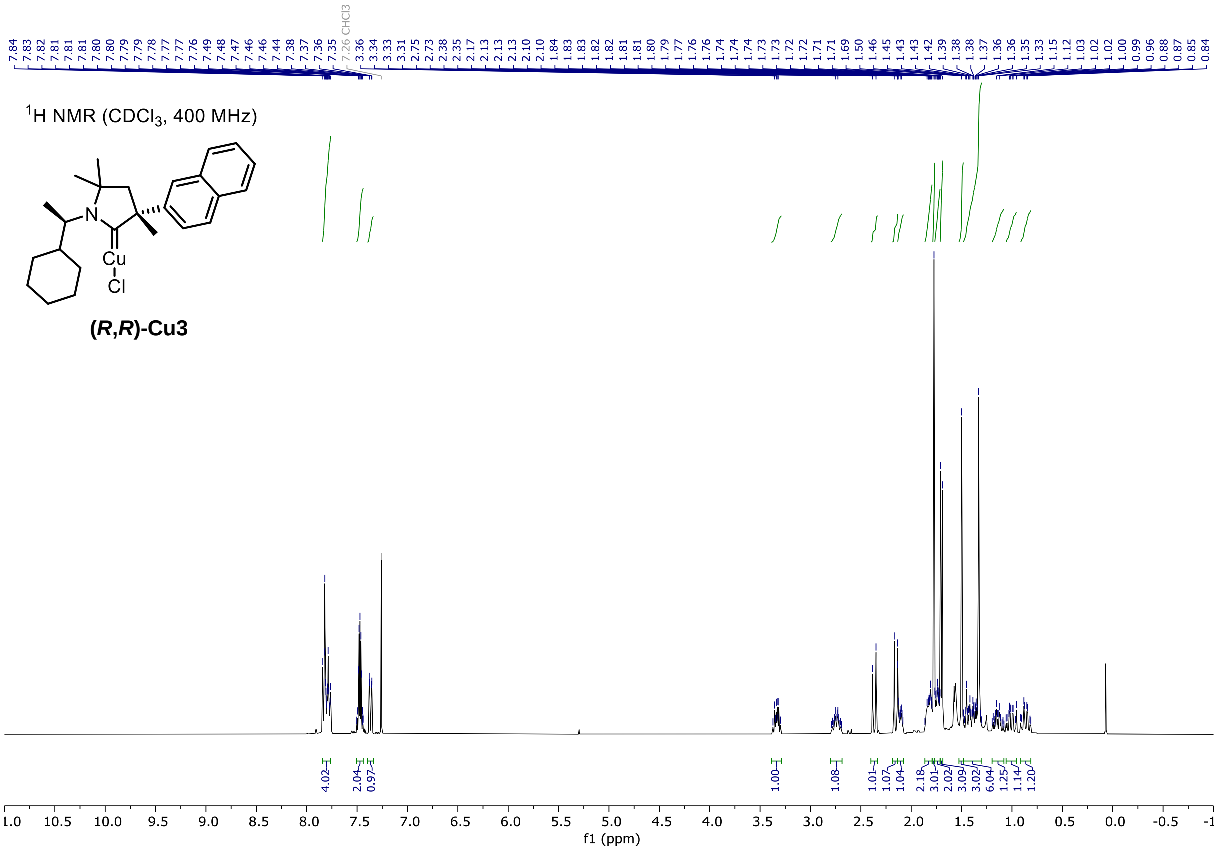
^{1}H NMR ($CDCl_3$, 400 MHz)
(R,R)-Cu3
f1 (ppm)

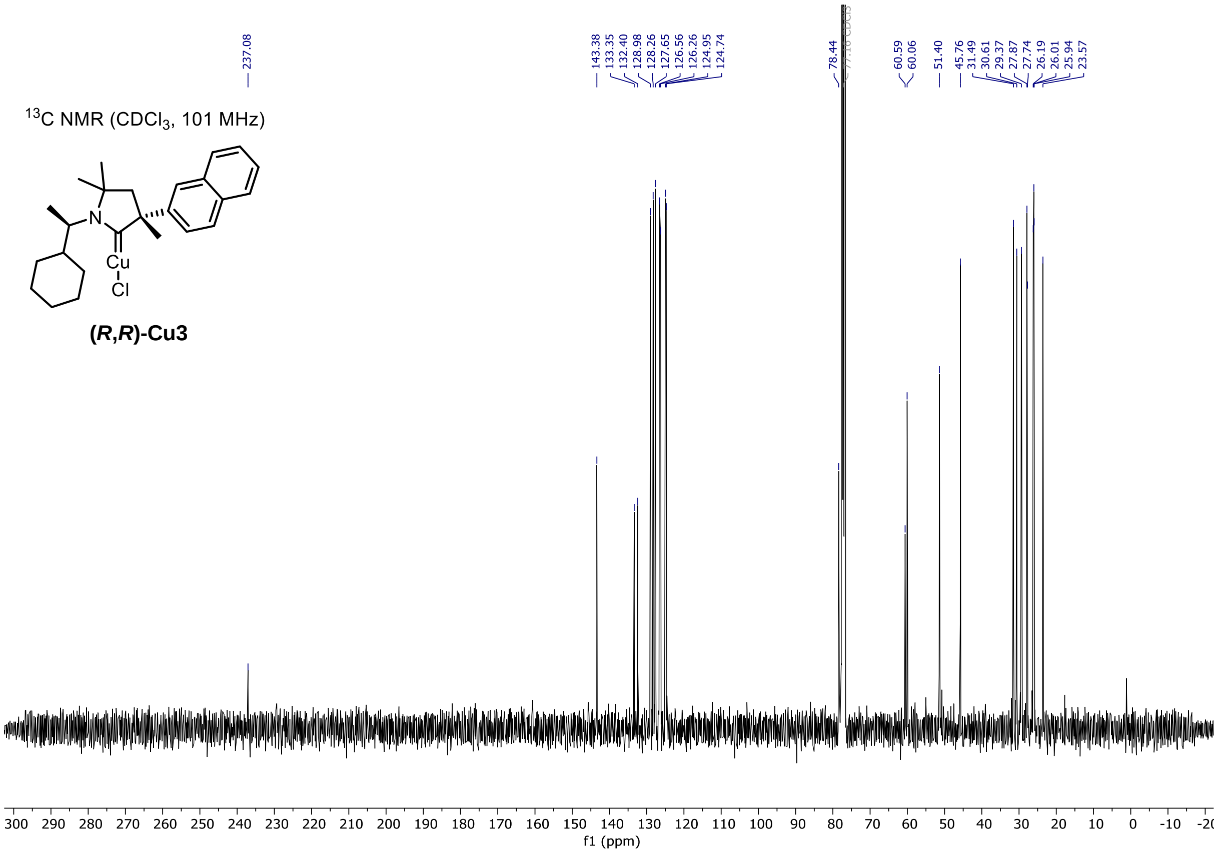
^{13}C NMR ($CDCl_3$, 101 MHz)
(R,R)-Cu3
f1 (ppm)

^{1}H NMR ($CDCl_3$, 400 MHz)

(*R*,*S*)-Cu4

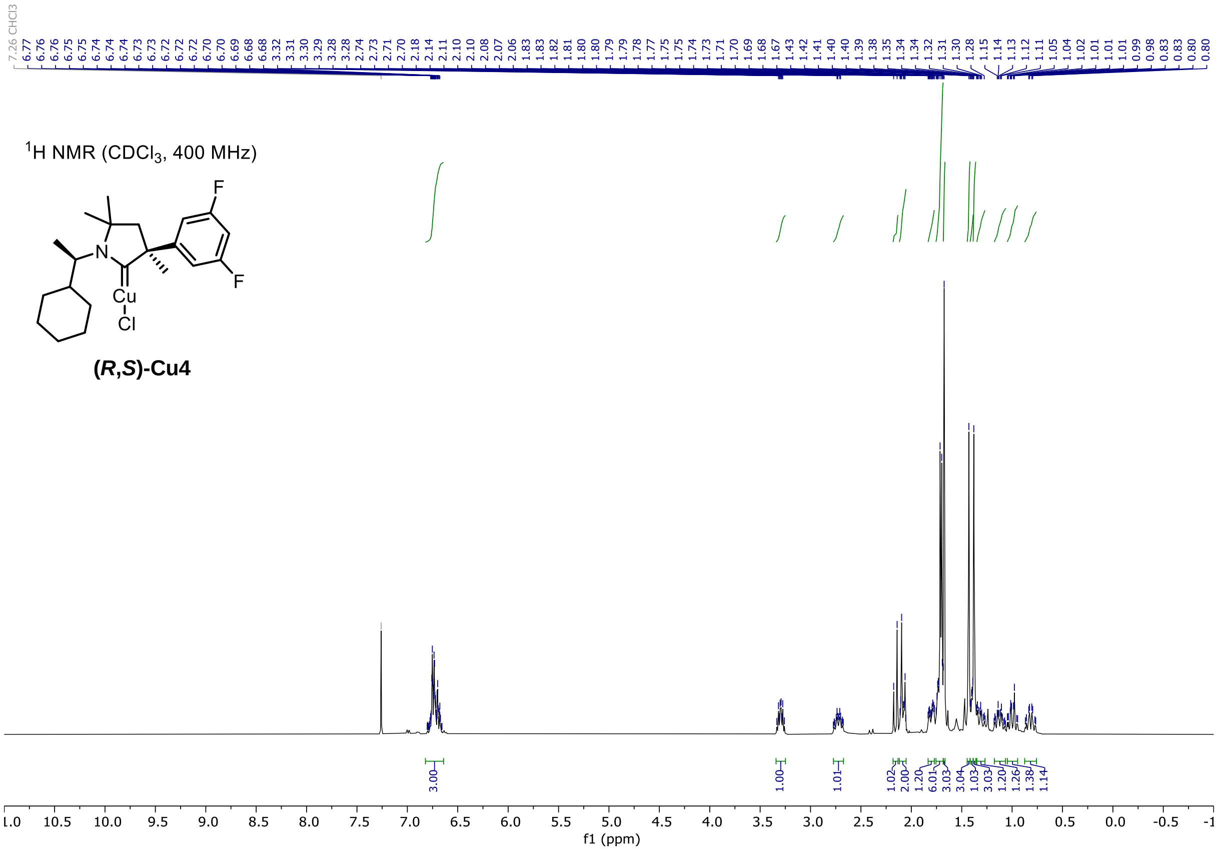

^{13}C NMR ($CDCl_3$, 101 MHz)

(*R*,*S*)-Cu4

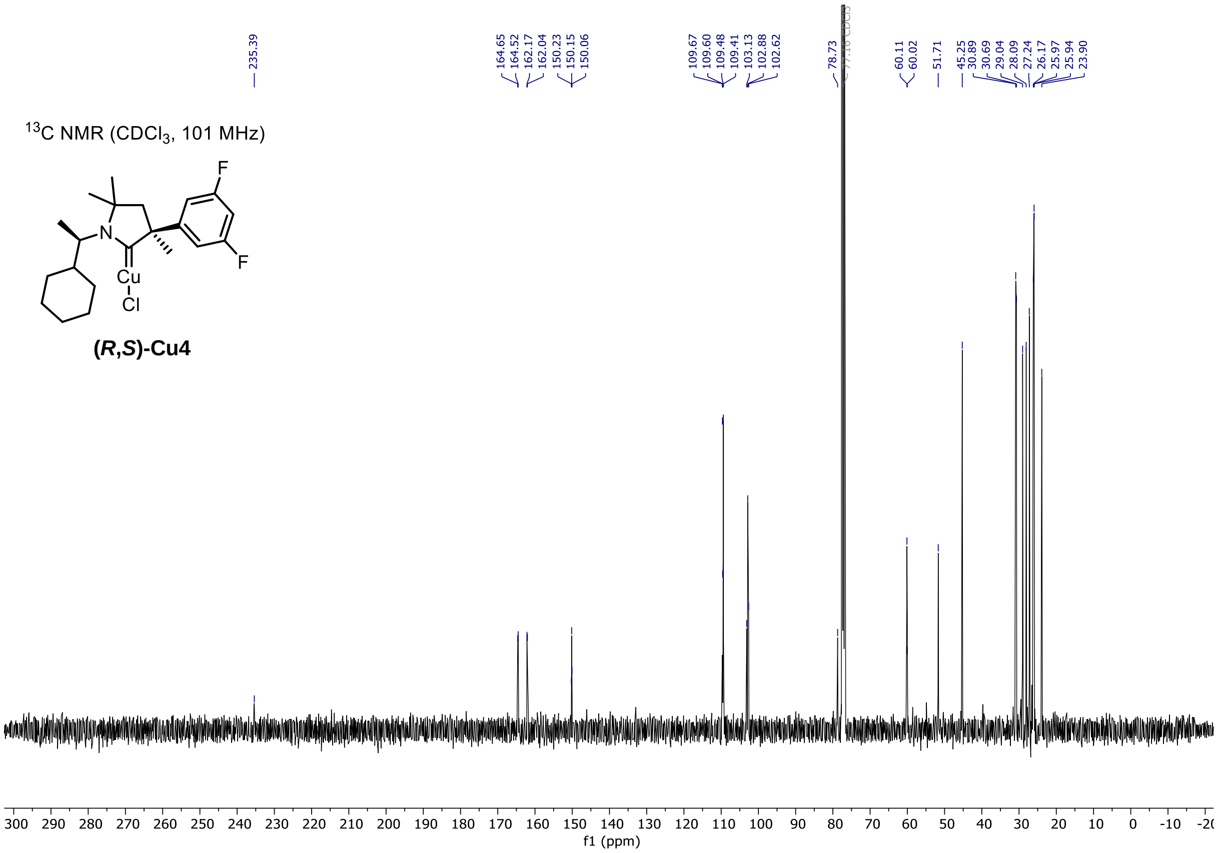

^{19}F NMR ($CDCl_3$, 376 MHz)

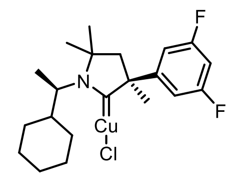

(*R*,*S*)-Cu4

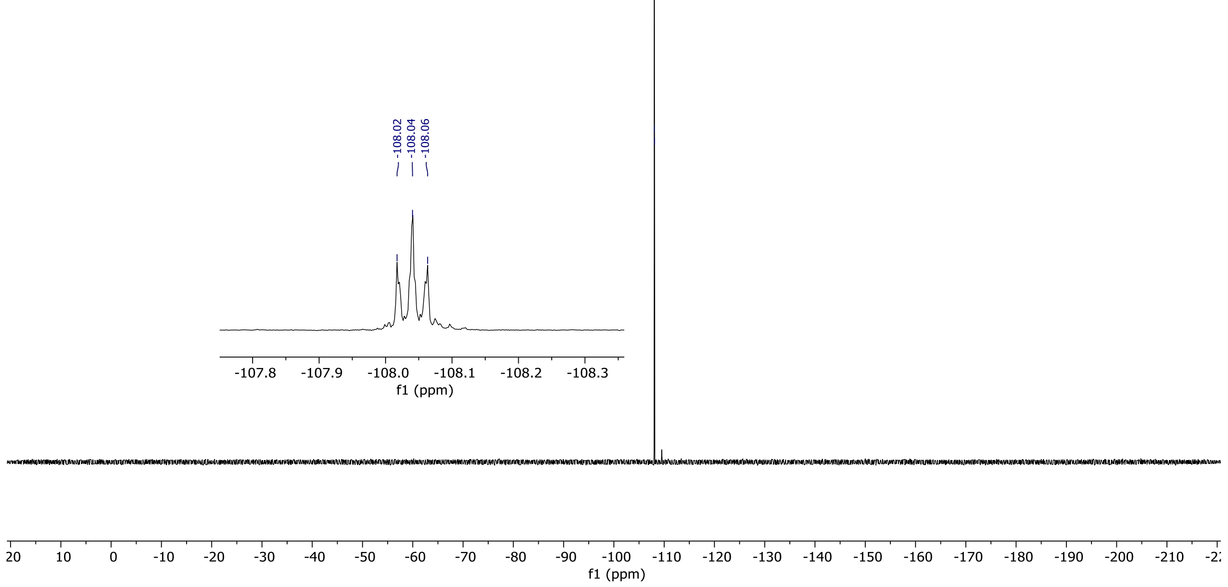

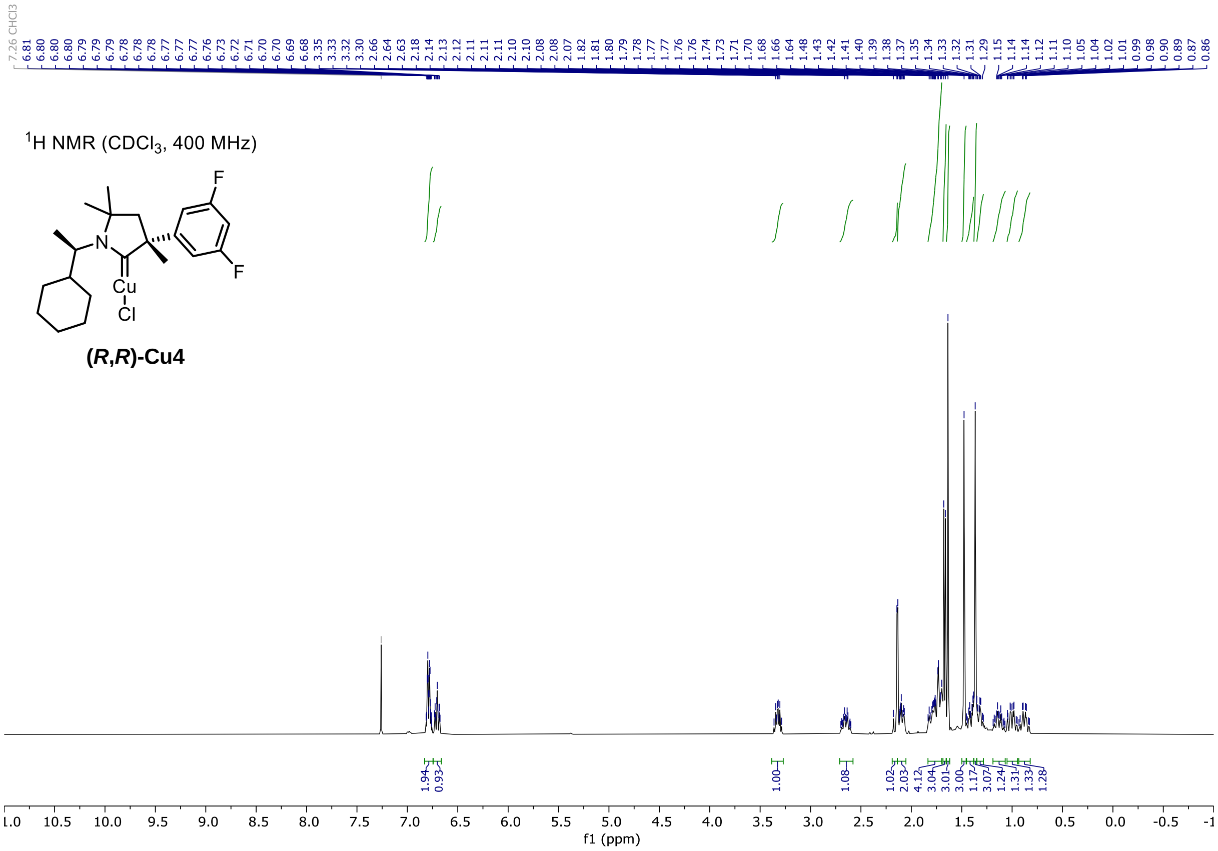

^{1}H NMR (CDCl$_3$, 400 MHz)
F
N
F
Cu
Cl
(R,R)-Cu4
f1 (ppm)

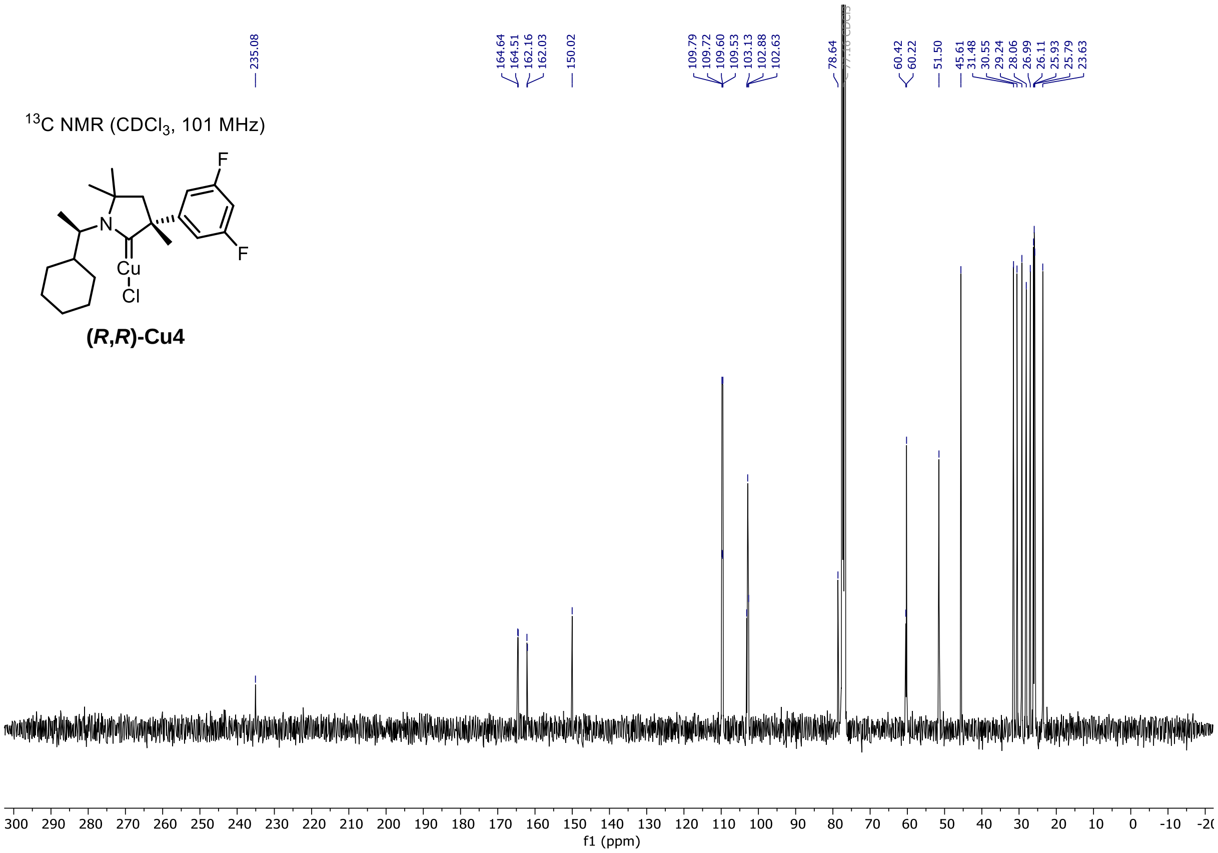

^{13}C NMR (CDCl$_3$, 101 MHz)
F
N
F
Cu
Cl
(R,R)-Cu4
f1 (ppm)

^{19}F NMR ($CDCl_3$, 376 MHz)

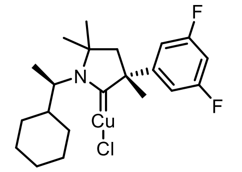

(*R*,*R*)-Cu4

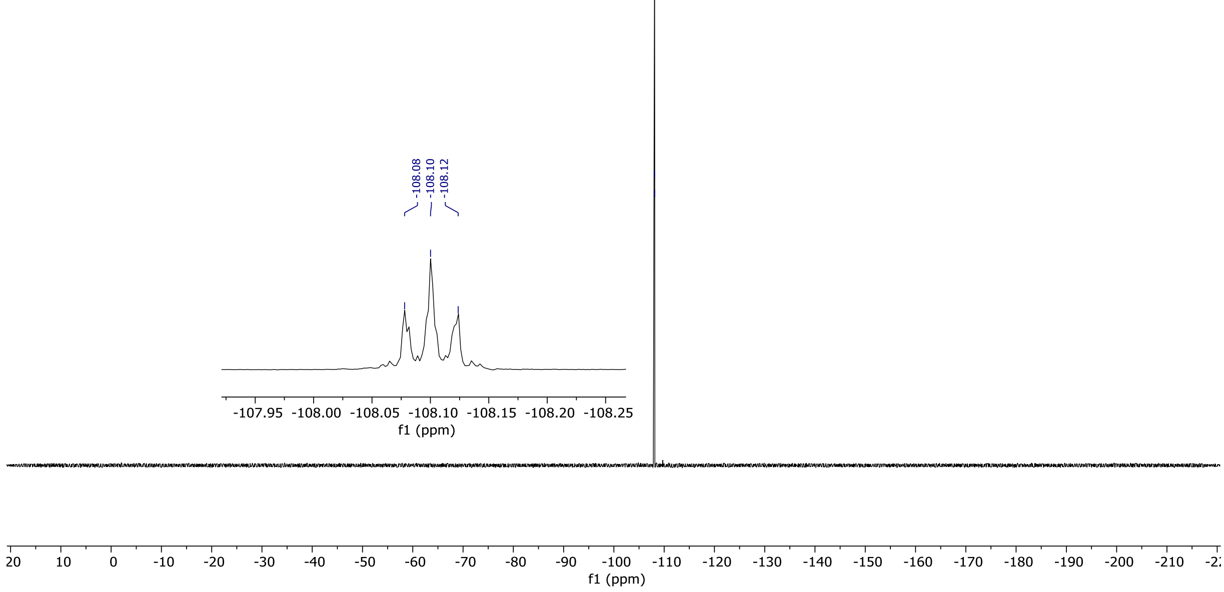

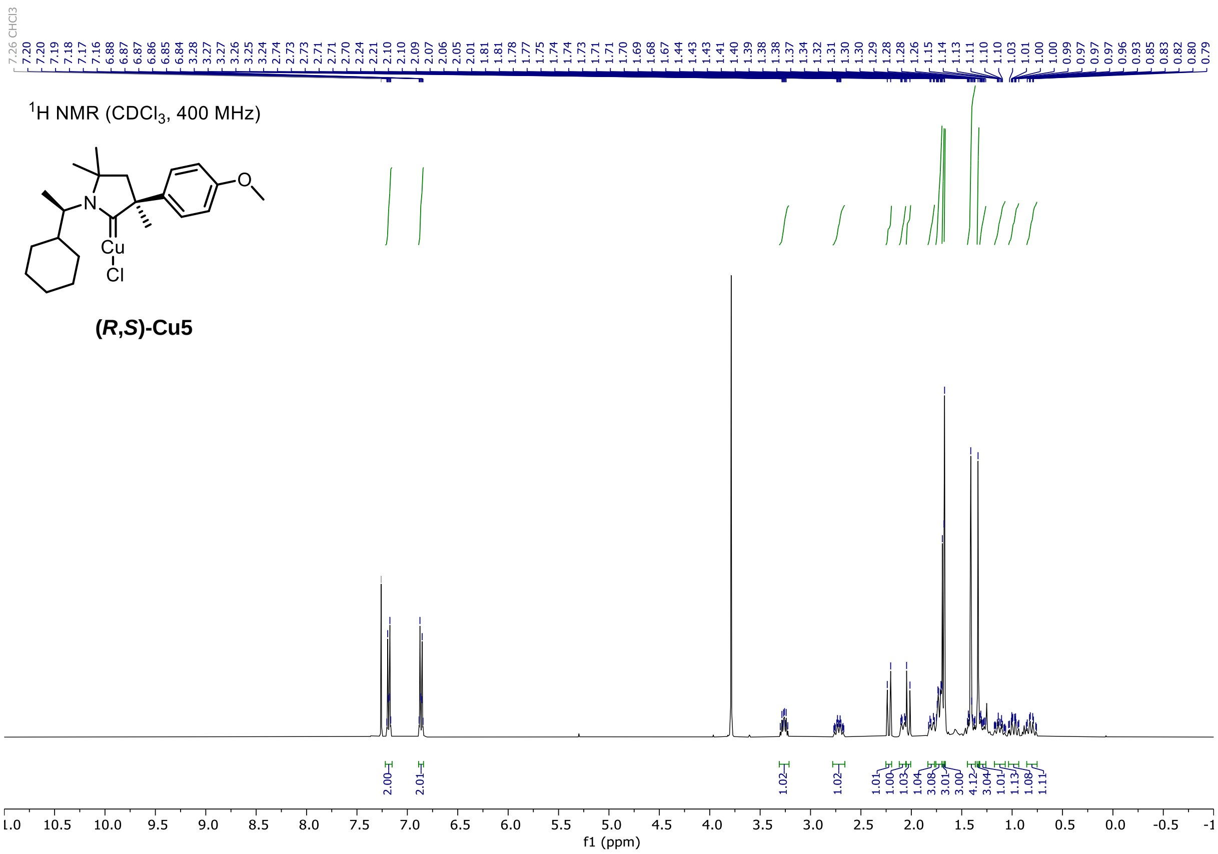

^{1}H NMR ($CDCl_3$, 400 MHz)

(*R*,*S*)-Cu5

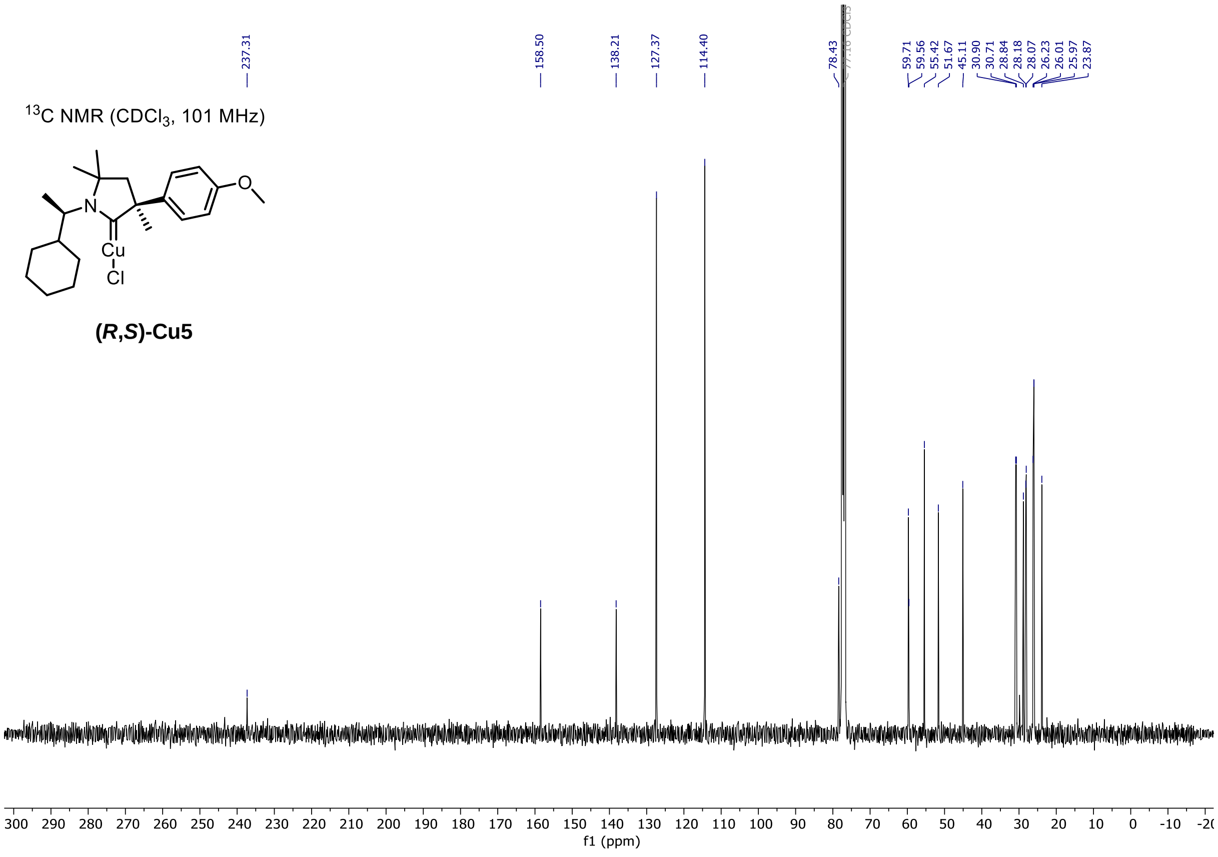

^{13}C NMR ($CDCl_3$, 101 MHz)

(*R*,*S*)-Cu5

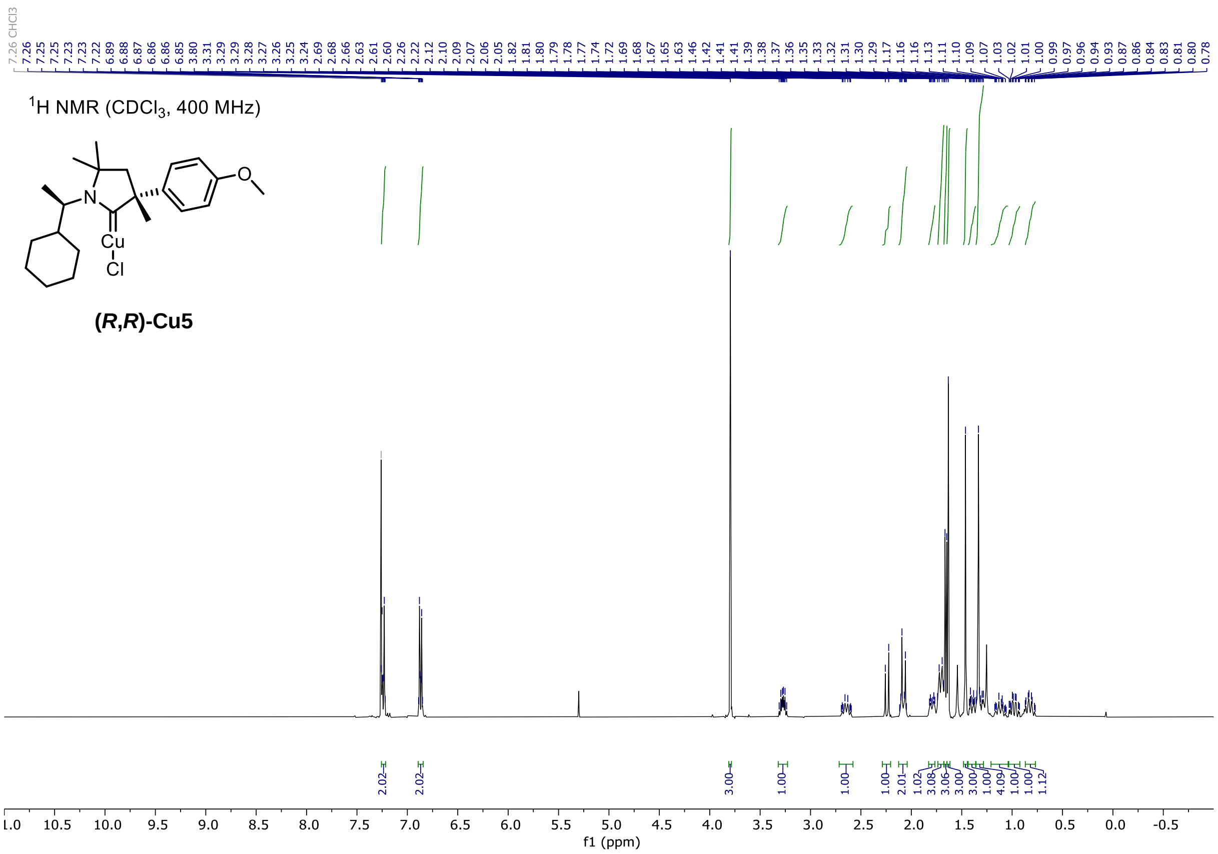
1H NMR (CDCl3, 400 MHz)
(R,R)-Cu5
Cu
Cl
N
O
2.02
2.02
3.00
1.00
1.00
1.00
2.01
1.02
3.08
3.06
3.00
3.00
1.00
4.09
1.00
1.00
1.12
f1 (ppm)

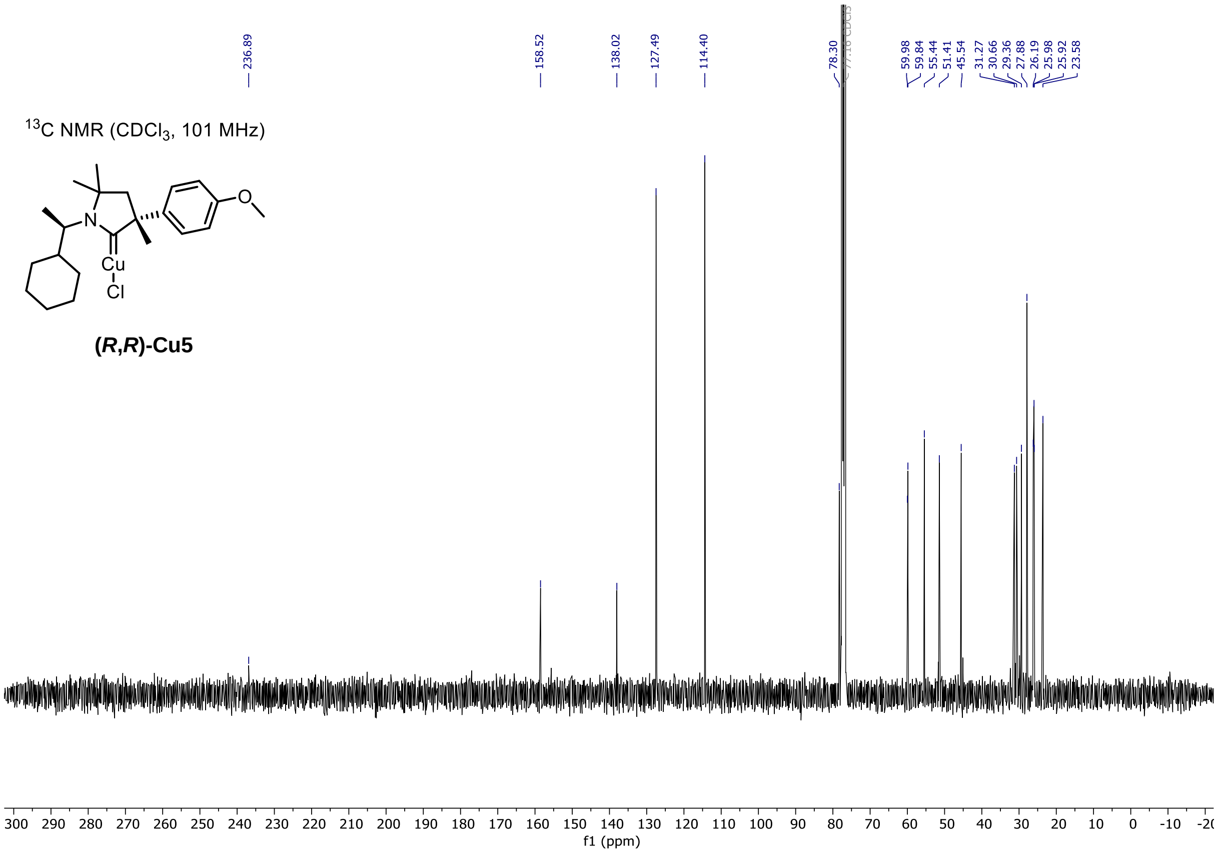
13C NMR (CDCl3, 101 MHz)
(R,R)-Cu5
Cu
Cl
N
O
236.89
158.52
138.02
127.49
114.40
78.30
77.16 CDCl3
59.98
59.84
55.44
51.41
45.54
31.27
30.66
29.36
27.88
26.19
25.98
25.92
23.58
f1 (ppm)

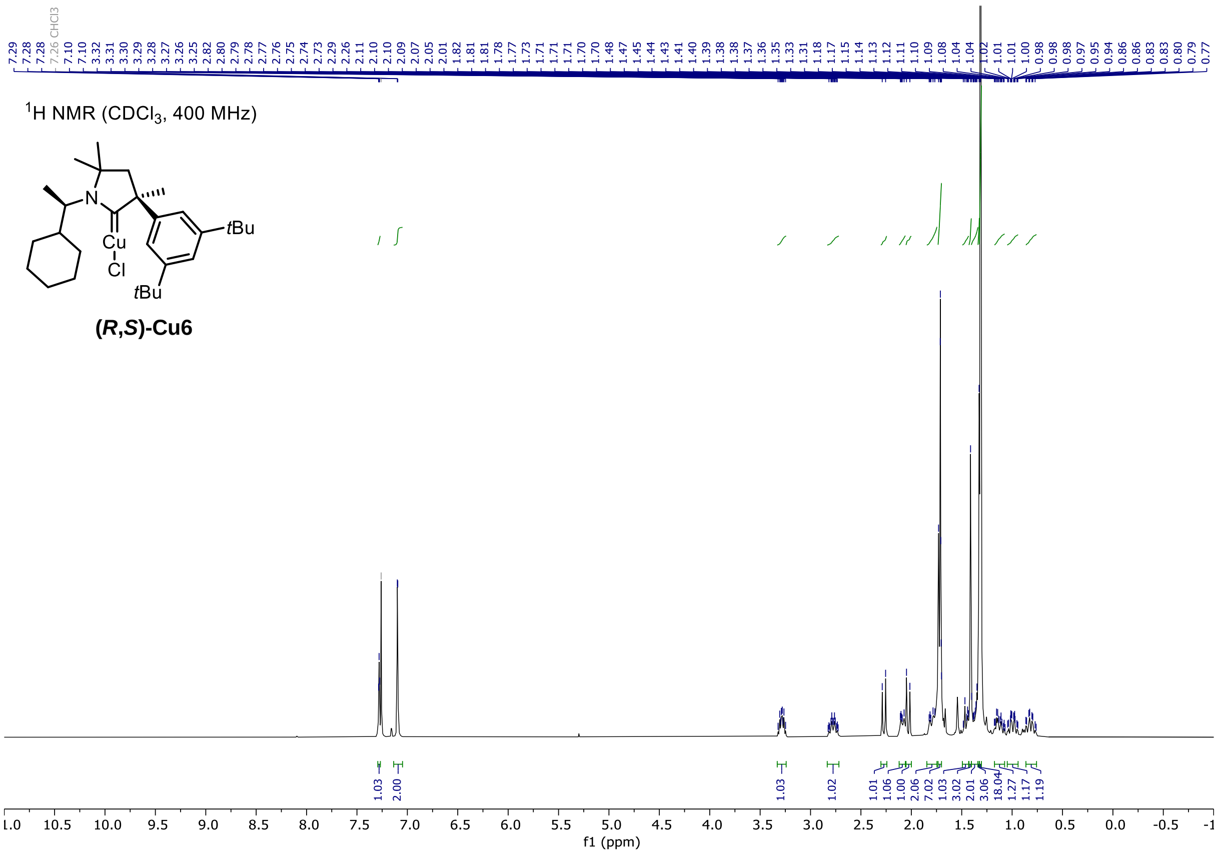
7.29 7.28 7.28 7.26 CHCl3 7.10 7.10 3.32 3.31 3.30 3.29 3.28 3.27 3.26 3.25 2.82 2.80 2.79 2.78 2.77 2.76 2.75 2.74 2.73 2.29 2.26 2.11 2.10 2.10 2.09 2.07 2.05 2.01 1.82 1.81 1.78 1.77 1.73 1.71 1.71 1.71 1.70 1.48 1.47 1.45 1.44 1.43 1.41 1.40 1.39 1.38 1.38 1.37 1.36 1.35 1.33 1.31 1.18 1.17 1.15 1.14 1.13 1.12 1.11 1.10 1.09 1.08 1.04 1.04 1.02 1.01 1.01 1.00 0.98 0.98 0.97 0.95 0.94 0.86 0.86 0.83 0.83 0.80 0.79 0.77
^{1}H NMR (CDCl$_3$, 400 MHz)
N
Cu
Cl
tBu
tBu
(R,S)-Cu6
1.03 2.00 1.03 1.02 1.01 1.06 1.00 2.06 7.02 1.03 3.02 2.01 3.06 18.04 1.27 1.17 1.19
11.0 10.5 10.0 9.5 9.0 8.5 8.0 7.5 7.0 6.5 6.0 5.5 5.0 4.5 4.0 3.5 3.0 2.5 2.0 1.5 1.0 0.5 0.0 -0.5 -1
f1 (ppm)

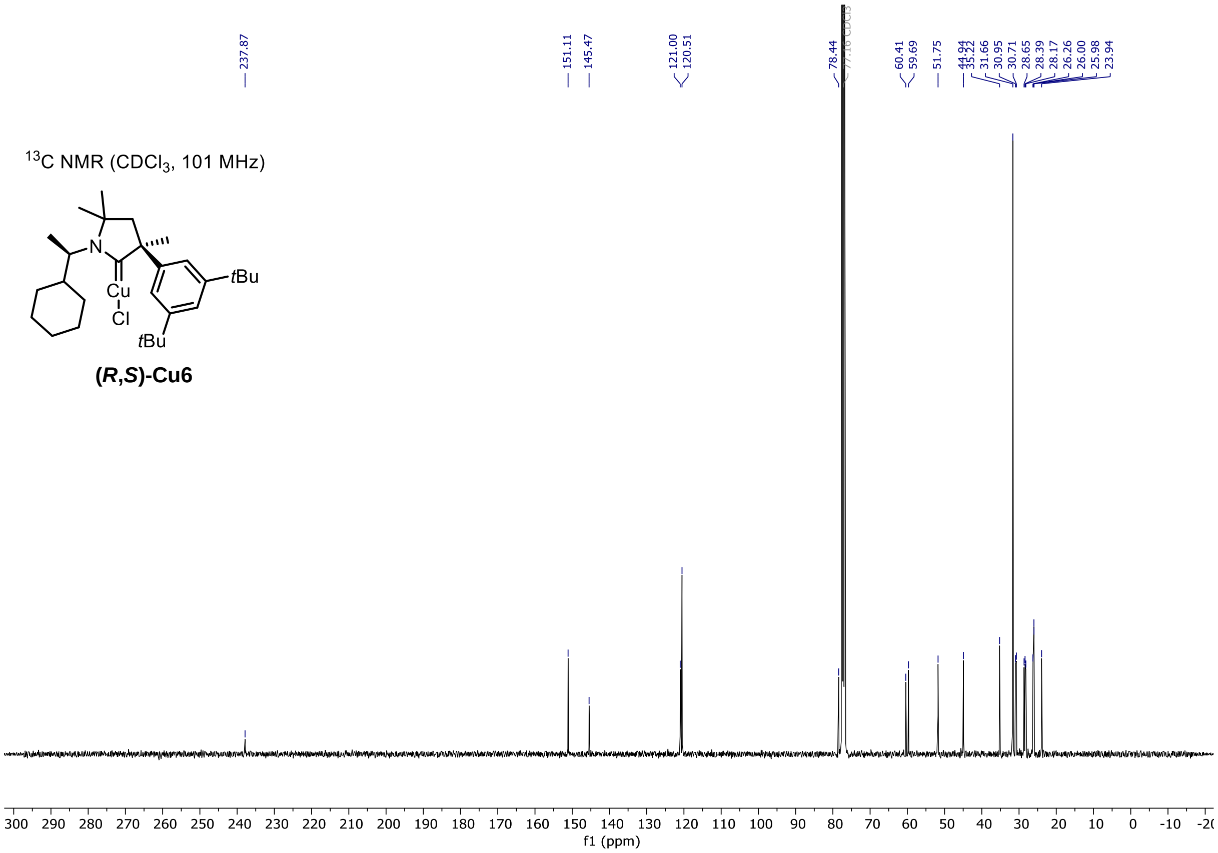
237.87 151.11 145.47 121.00 120.51 78.44 77.16 CDCl3 60.41 59.69 51.75 44.94 35.22 31.66 30.95 30.71 28.65 28.39 28.17 26.26 26.00 25.98 23.94
^{13}C NMR (CDCl$_3$, 101 MHz)
N
Cu
Cl
tBu
tBu
(R,S)-Cu6
300 290 280 270 260 250 240 230 220 210 200 190 180 170 160 150 140 130 120 110 100 90 80 70 60 50 40 30 20 10 0 -10 -20
f1 (ppm)

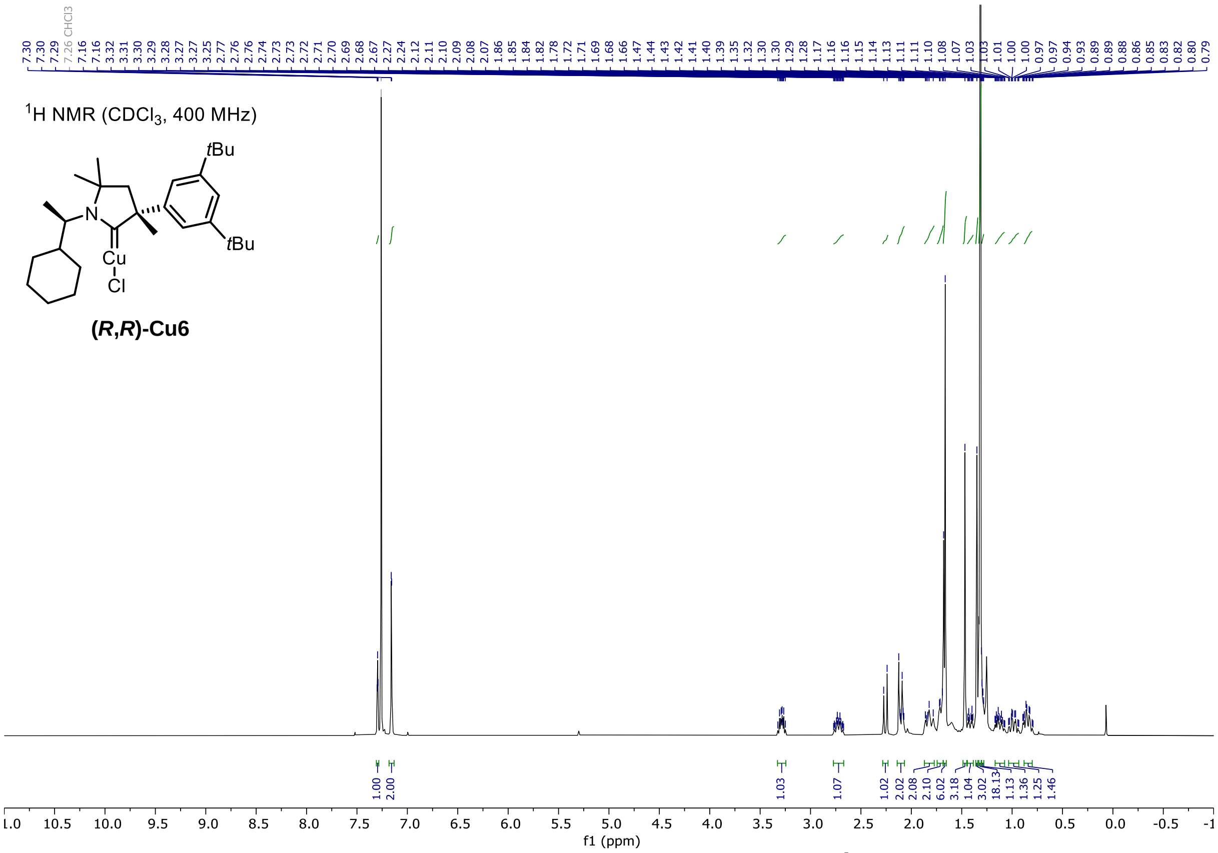

^{1}H NMR (CDCl$_3$, 400 MHz)
tBu
tBu
N
Cu
Cl
(R,R)-Cu6
f1 (ppm)

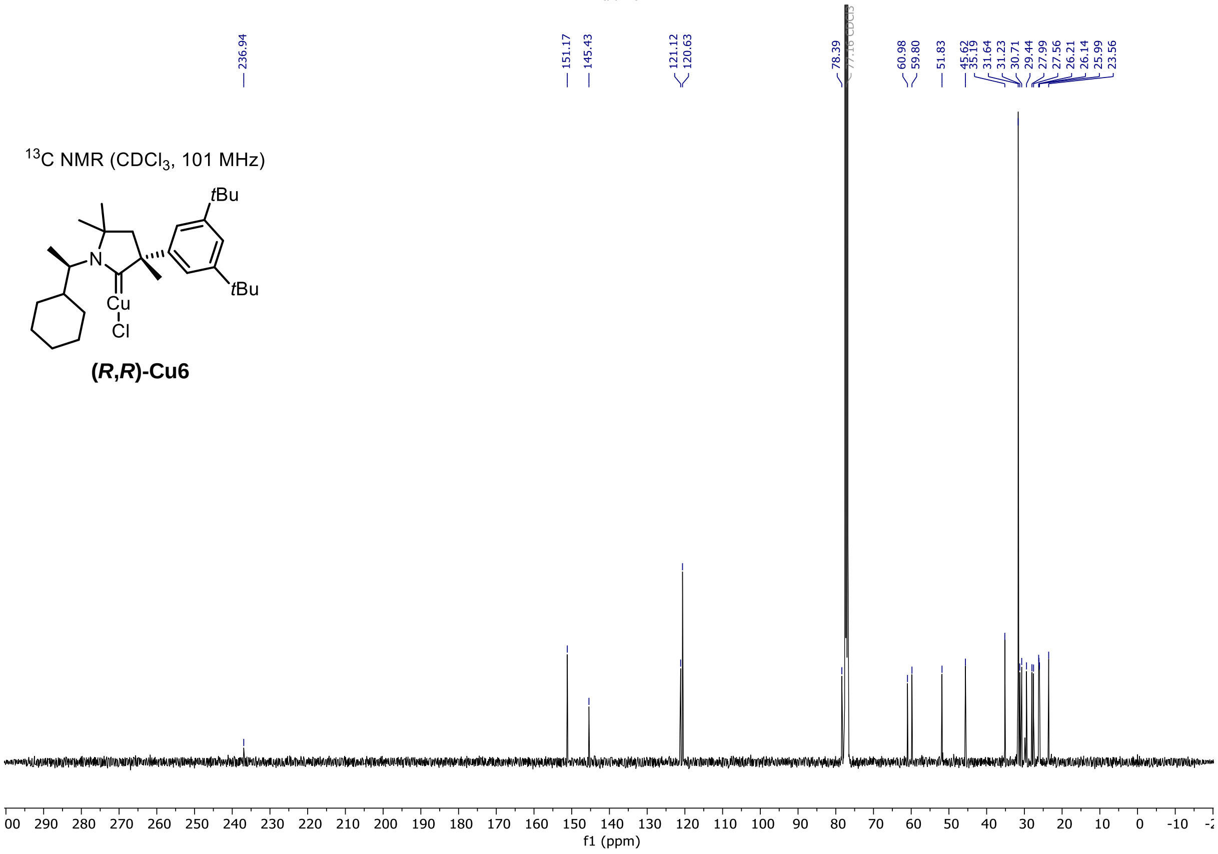

^{13}C NMR (CDCl$_3$, 101 MHz)
tBu
tBu
N
Cu
Cl
(R,R)-Cu6
f1 (ppm)

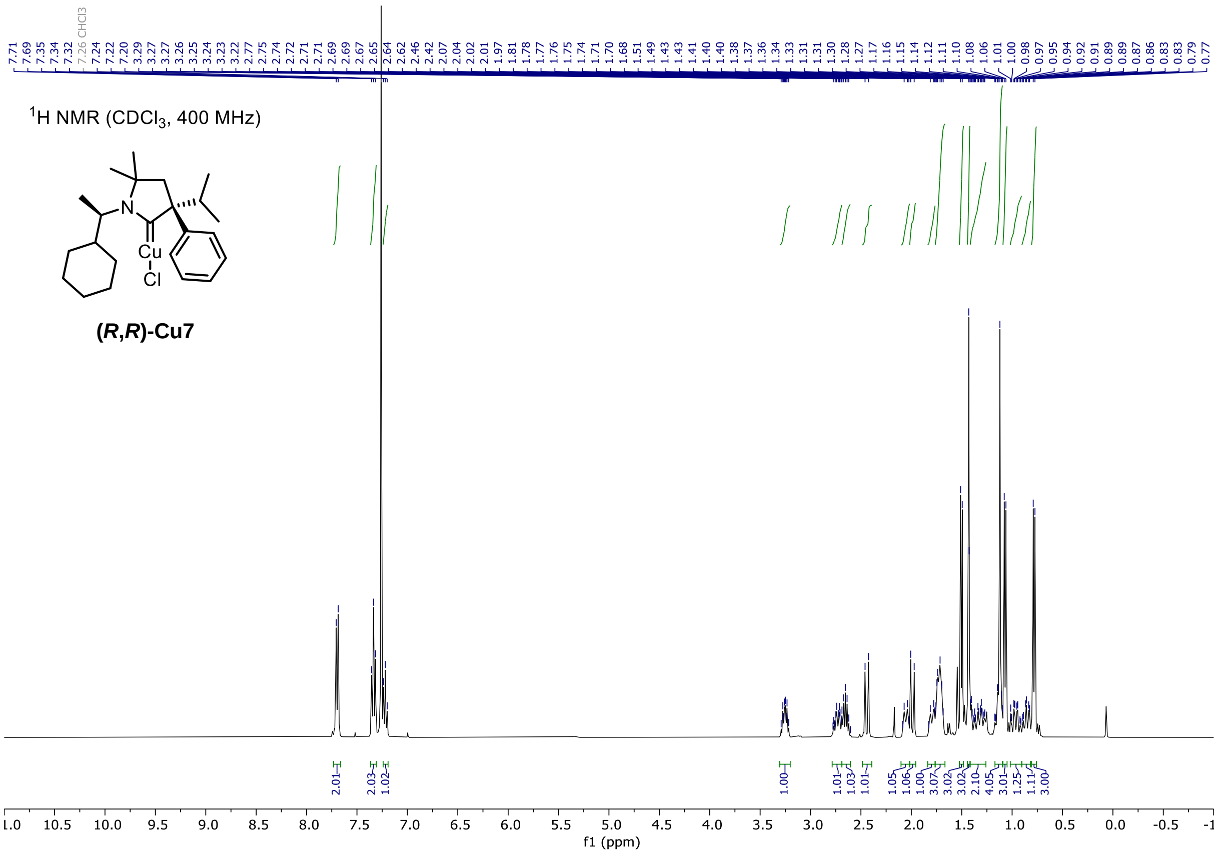

1H NMR (CDCl3, 400 MHz)
(R,R)-Cu7
f1 (ppm)

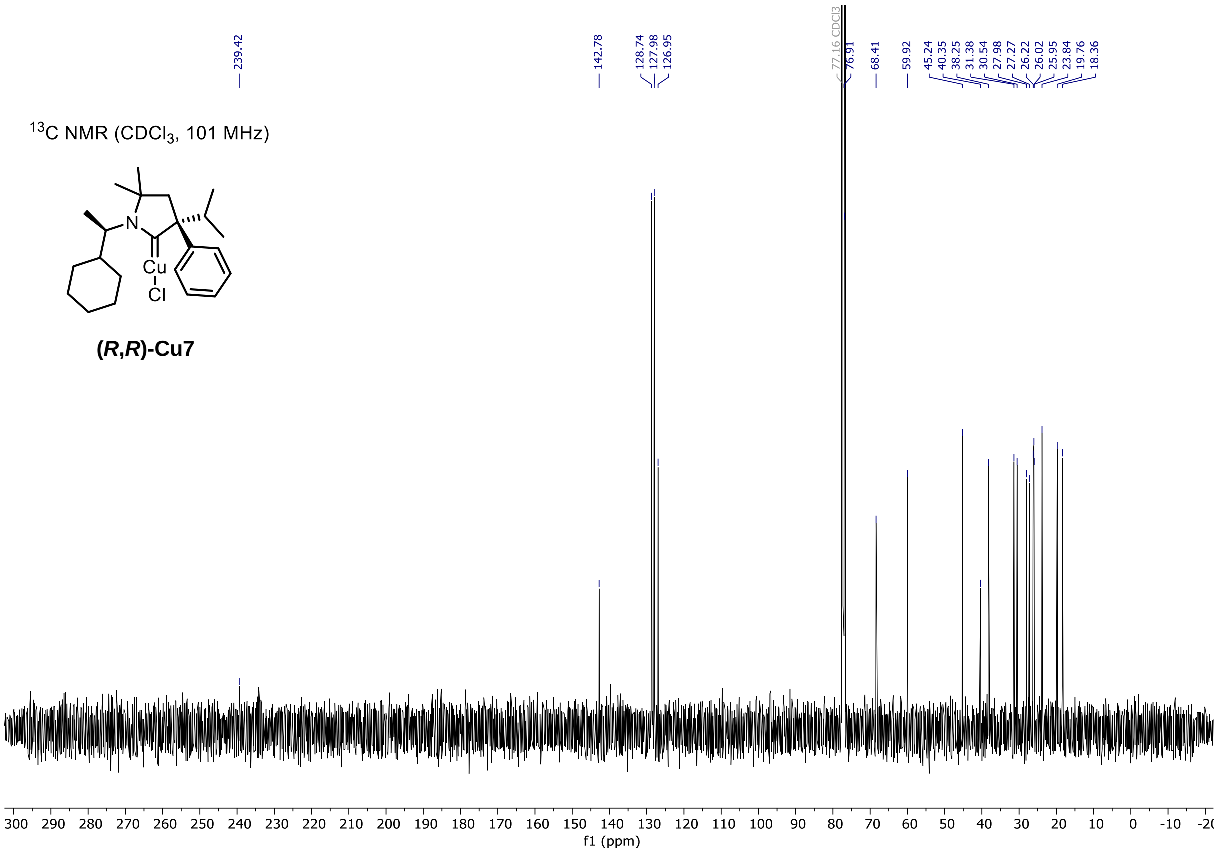

13C NMR (CDCl3, 101 MHz)
(R,R)-Cu7
239.42
142.78
128.74
127.98
126.95
77.16 CDCl3
76.91
68.41
59.92
45.24
40.35
38.25
31.38
30.54
27.98
27.27
26.22
26.02
25.95
23.84
19.76
18.36
f1 (ppm)

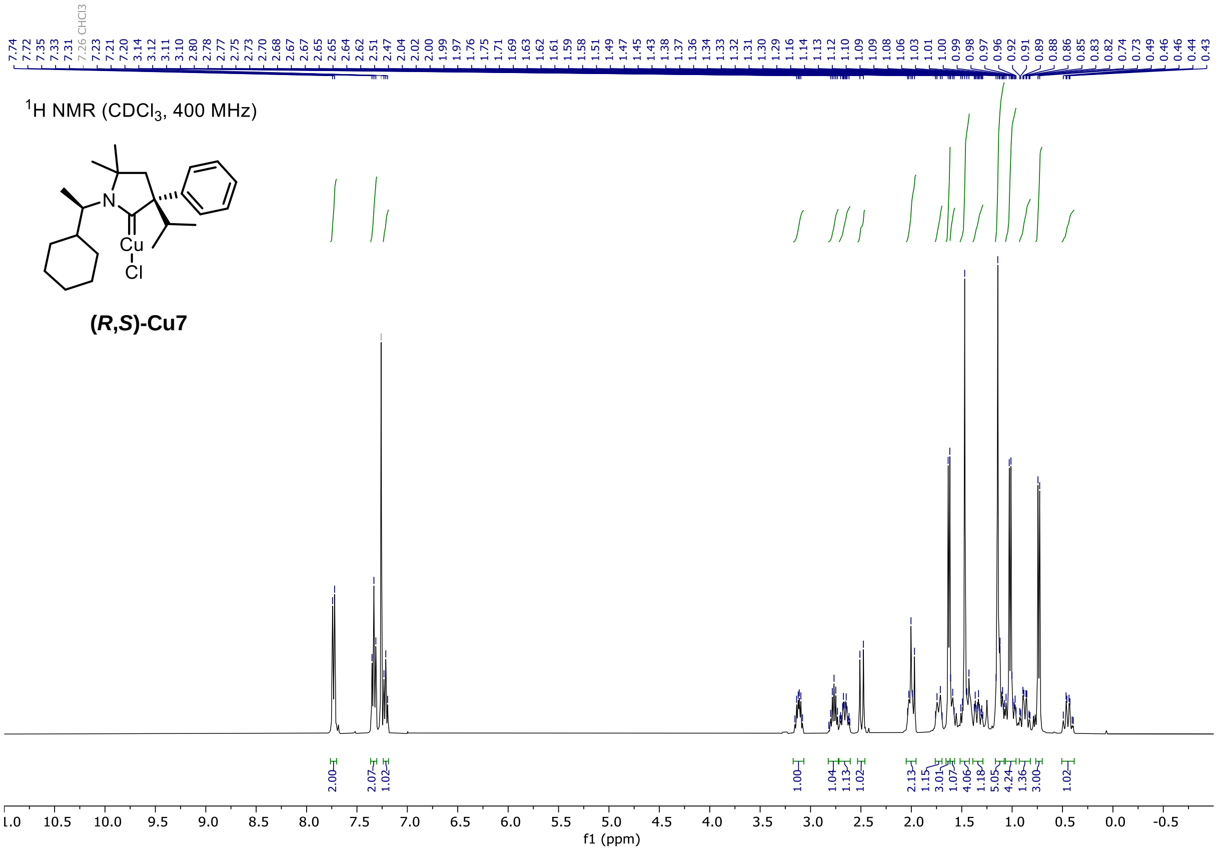

1H NMR (CDCl3, 400 MHz)
Cu
Cl
N
(R,S)-Cu7
f1 (ppm)

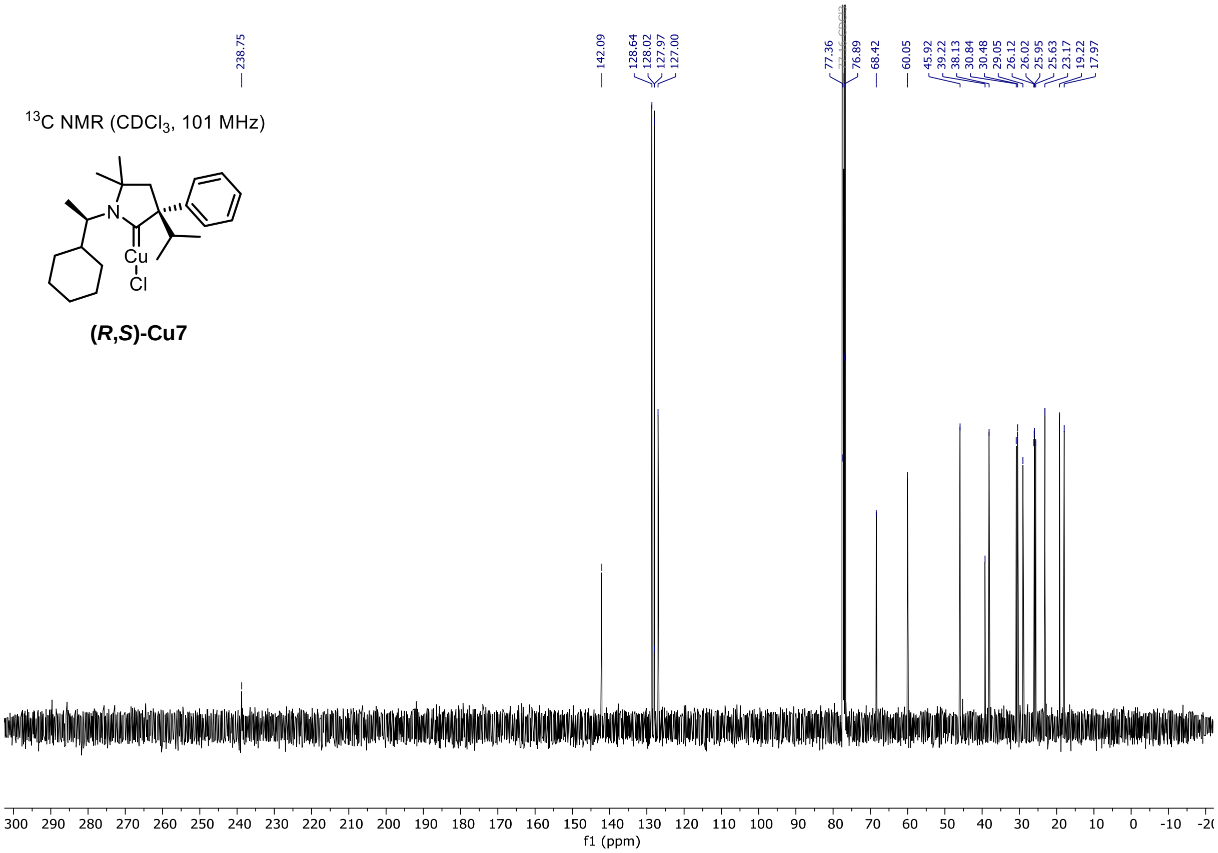

13C NMR (CDCl3, 101 MHz)
Cu
Cl
N
(R,S)-Cu7
238.75
142.09
128.64
128.02
127.97
127.00
77.36
76.89
68.42
60.05
45.92
39.22
38.13
30.84
30.48
29.05
26.12
26.02
25.95
25.63
23.17
19.22
17.97
f1 (ppm)

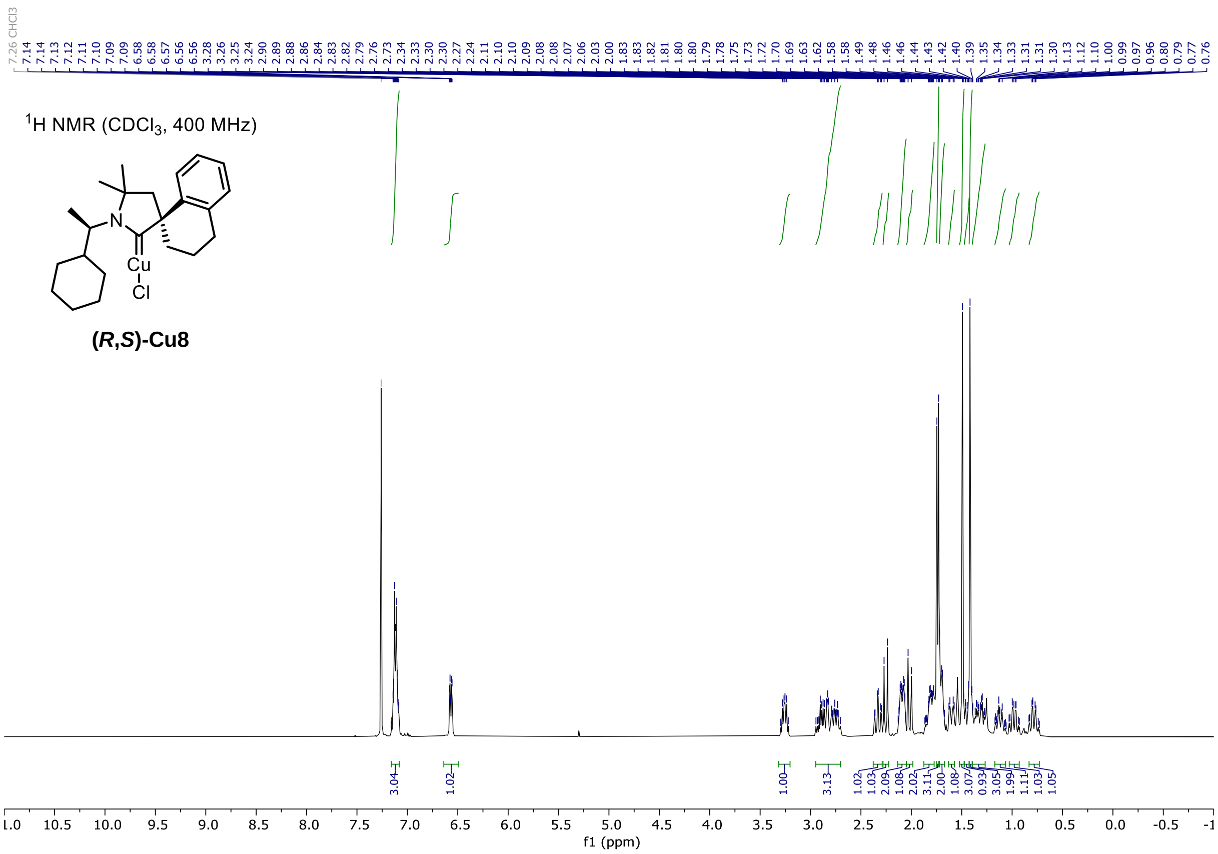

7.26 CHCl3
¹H NMR (CDCl3, 400 MHz)
N
Cu
Cl
(R,S)-Cu8
f1 (ppm)

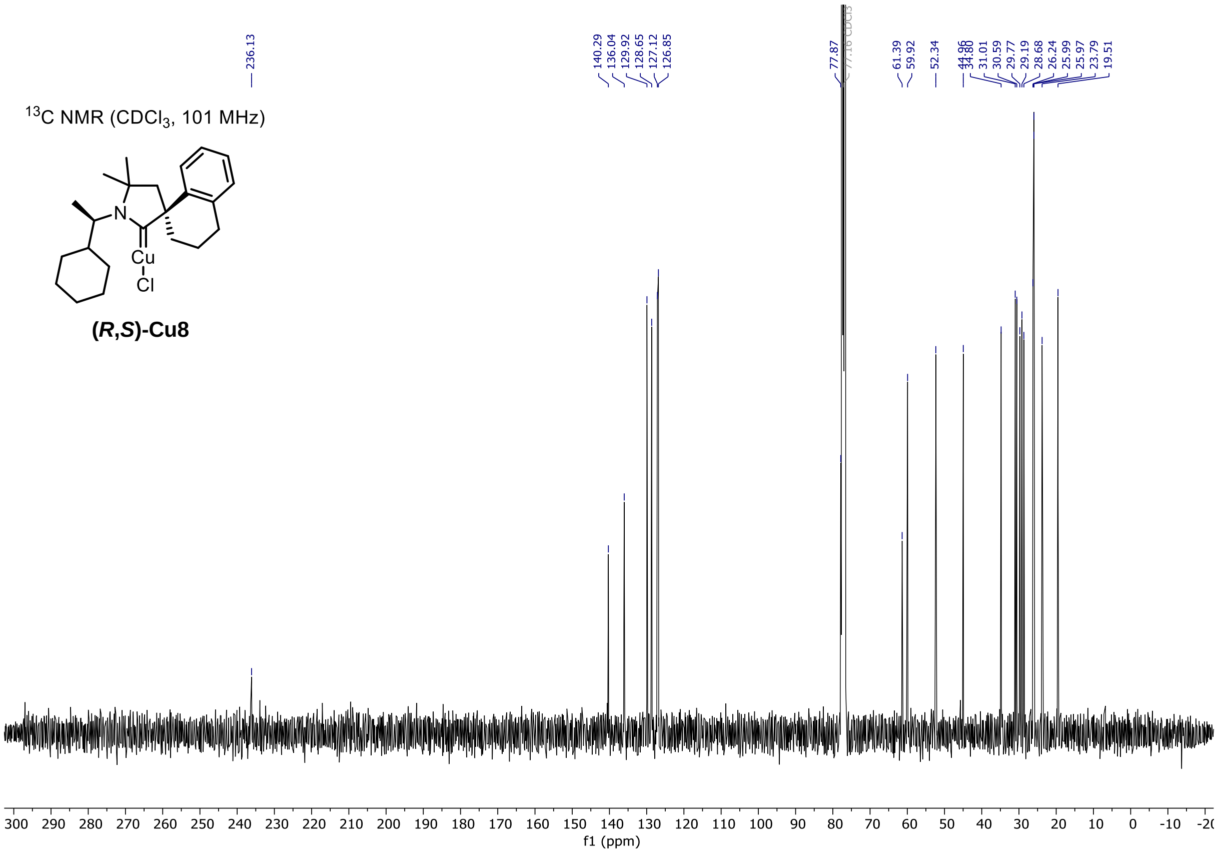

236.13
140.29
136.04
129.92
128.65
127.12
126.85
77.87
77.16 CDCl3
61.39
59.92
52.34
44.96
34.80
31.01
30.59
29.77
29.19
28.68
26.24
25.99
25.97
23.79
19.51
¹³C NMR (CDCl3, 101 MHz)
N
Cu
Cl
(R,S)-Cu8
f1 (ppm)

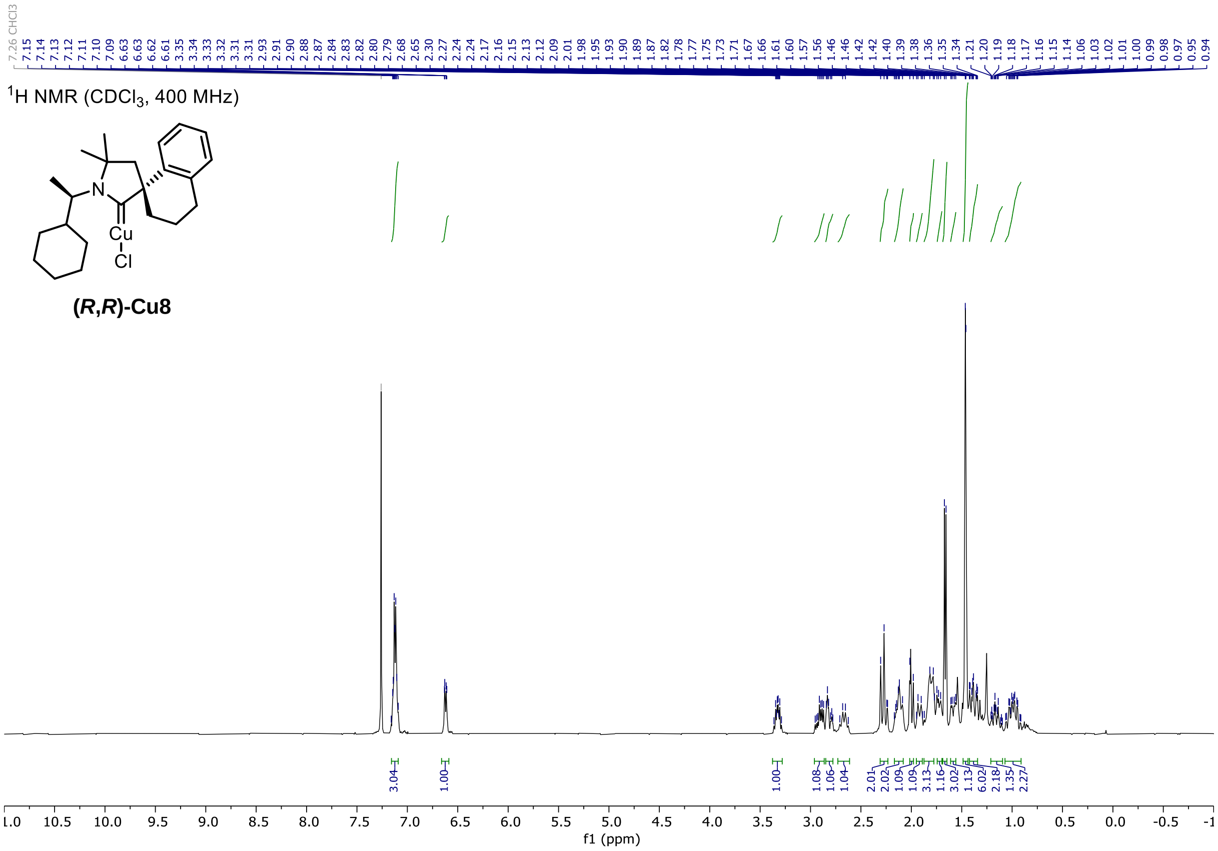
^{1}H NMR (CDCl$_3$, 400 MHz)
Cu
Cl
(*R*,*R*)-Cu8
f1 (ppm)

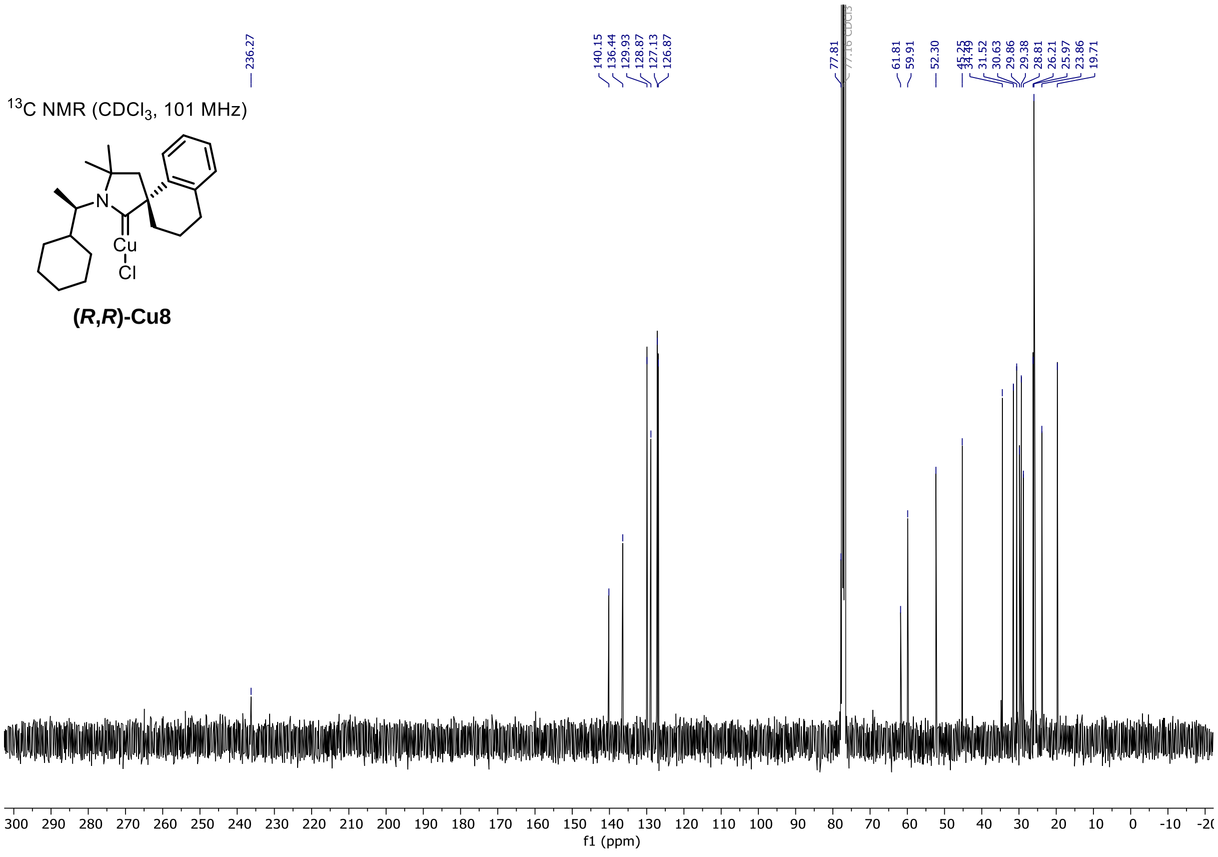
^{13}C NMR (CDCl$_3$, 101 MHz)
Cu
Cl
(*R*,*R*)-Cu8
f1 (ppm)

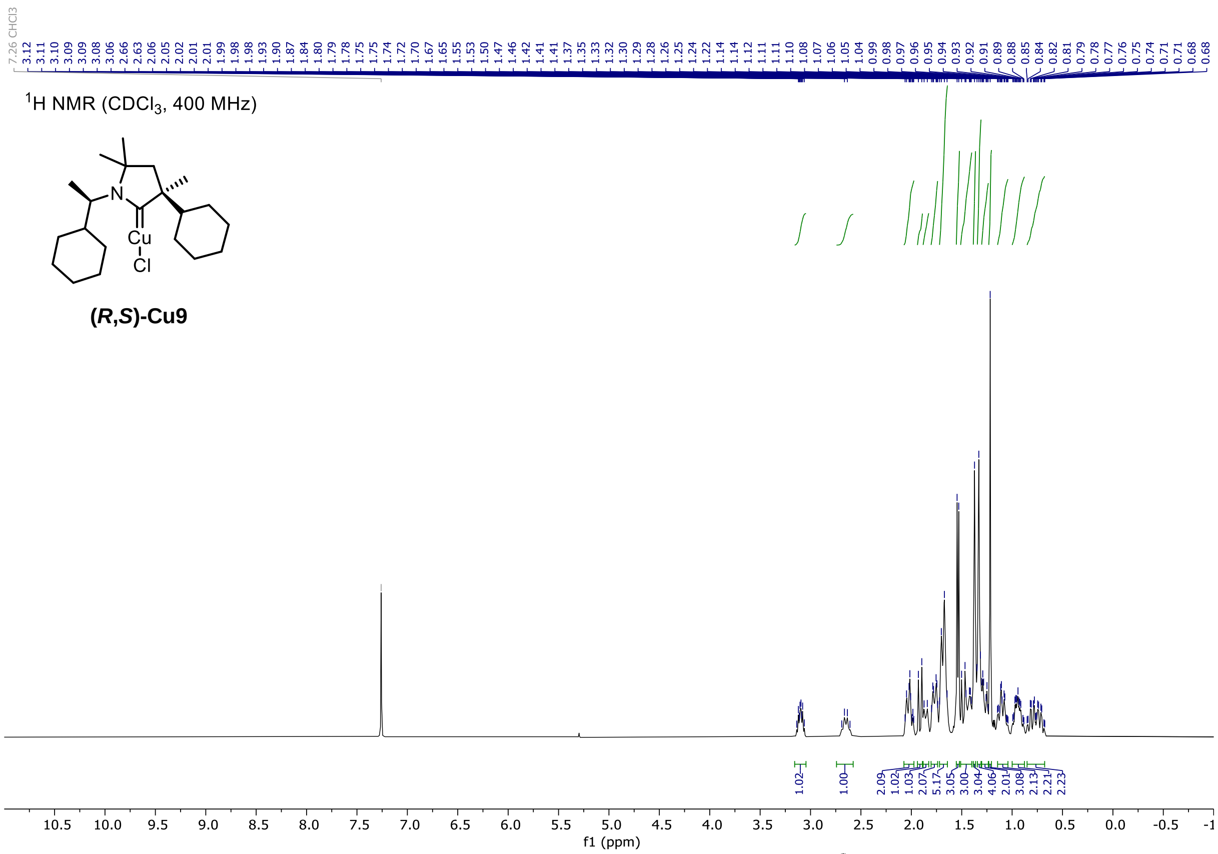

1H NMR (CDCl3, 400 MHz)
Cu
Cl
N
(R,S)-Cu9
f1 (ppm)

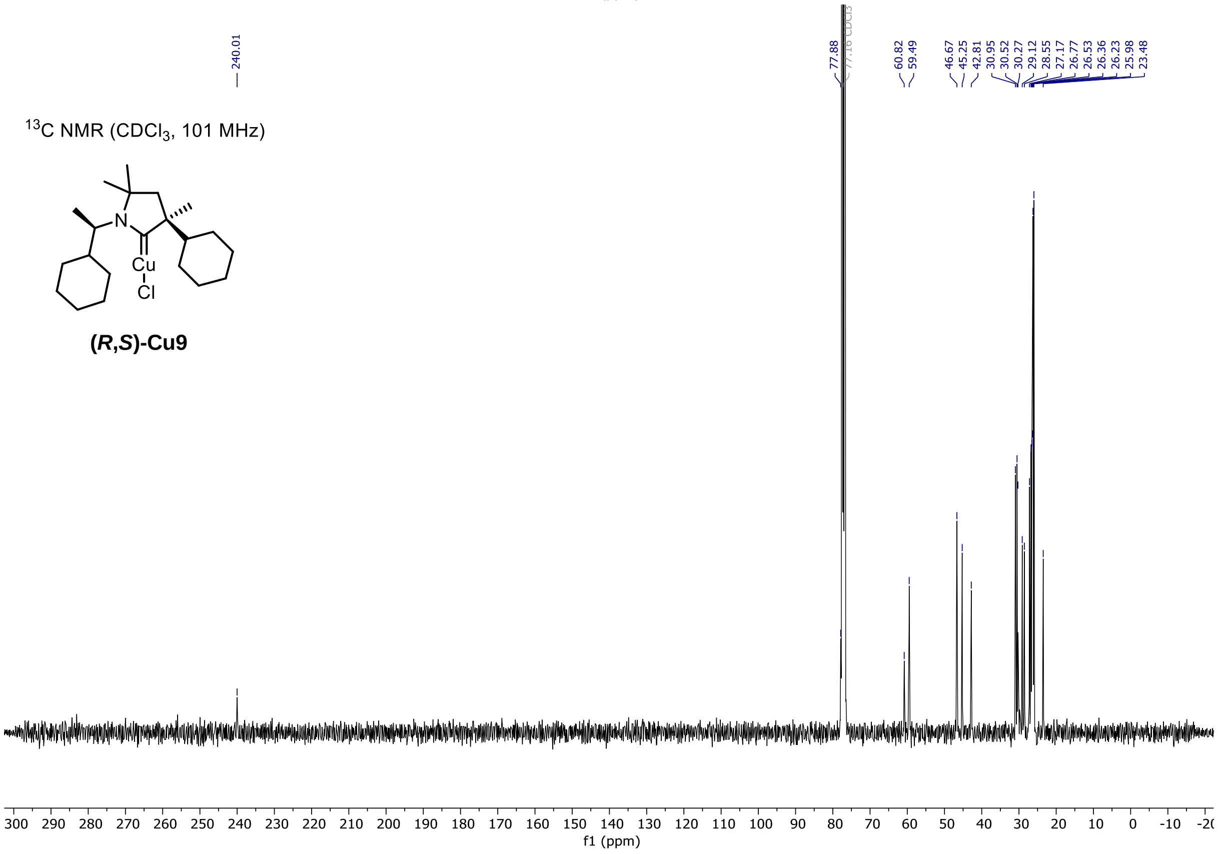

13C NMR (CDCl3, 101 MHz)
240.01
77.88
77.16 CDCl3
60.82
59.49
46.67
45.25
42.81
30.95
30.52
30.27
29.12
28.55
27.17
26.77
26.53
26.36
26.23
25.98
23.48
Cu
Cl
N
(R,S)-Cu9
f1 (ppm)

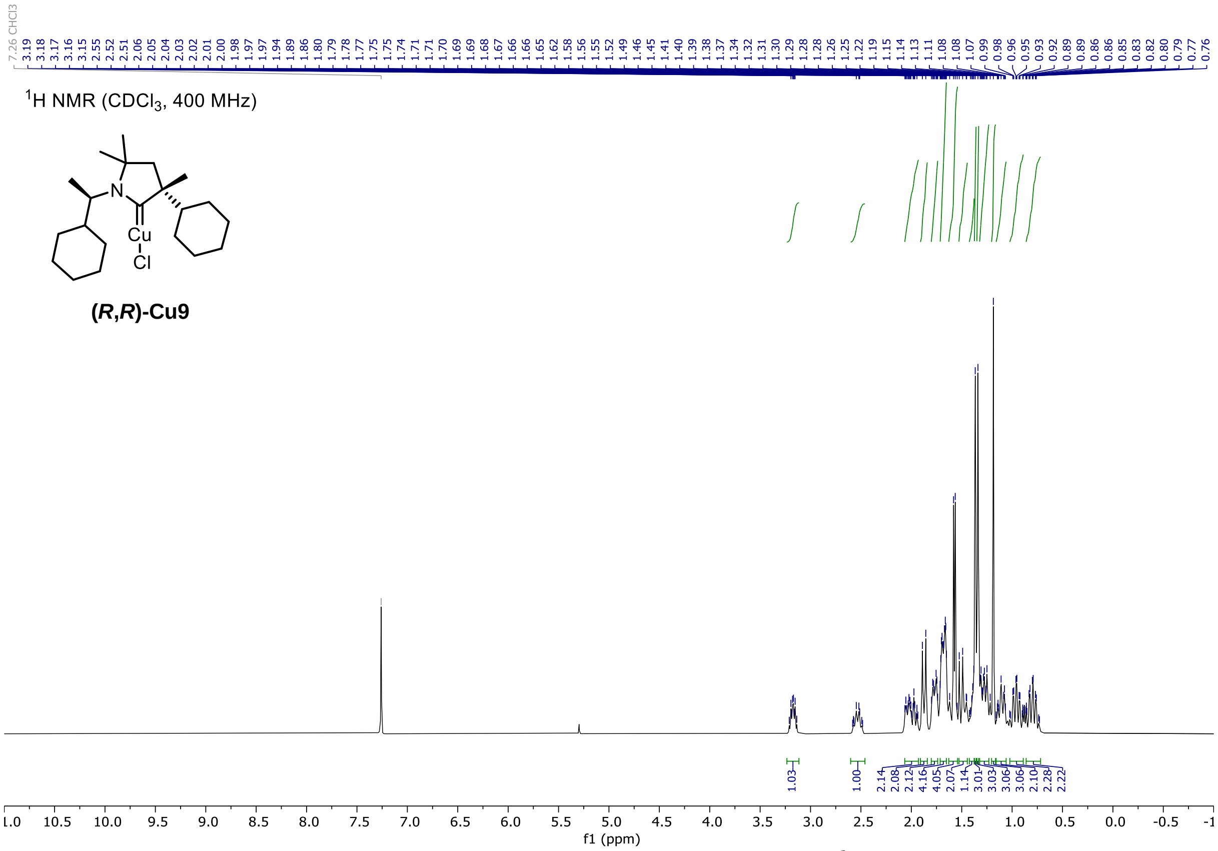

^{1}H NMR (CDCl$_3$, 400 MHz)
Cu
Cl
N
(*R*,*R*)-Cu9
f1 (ppm)

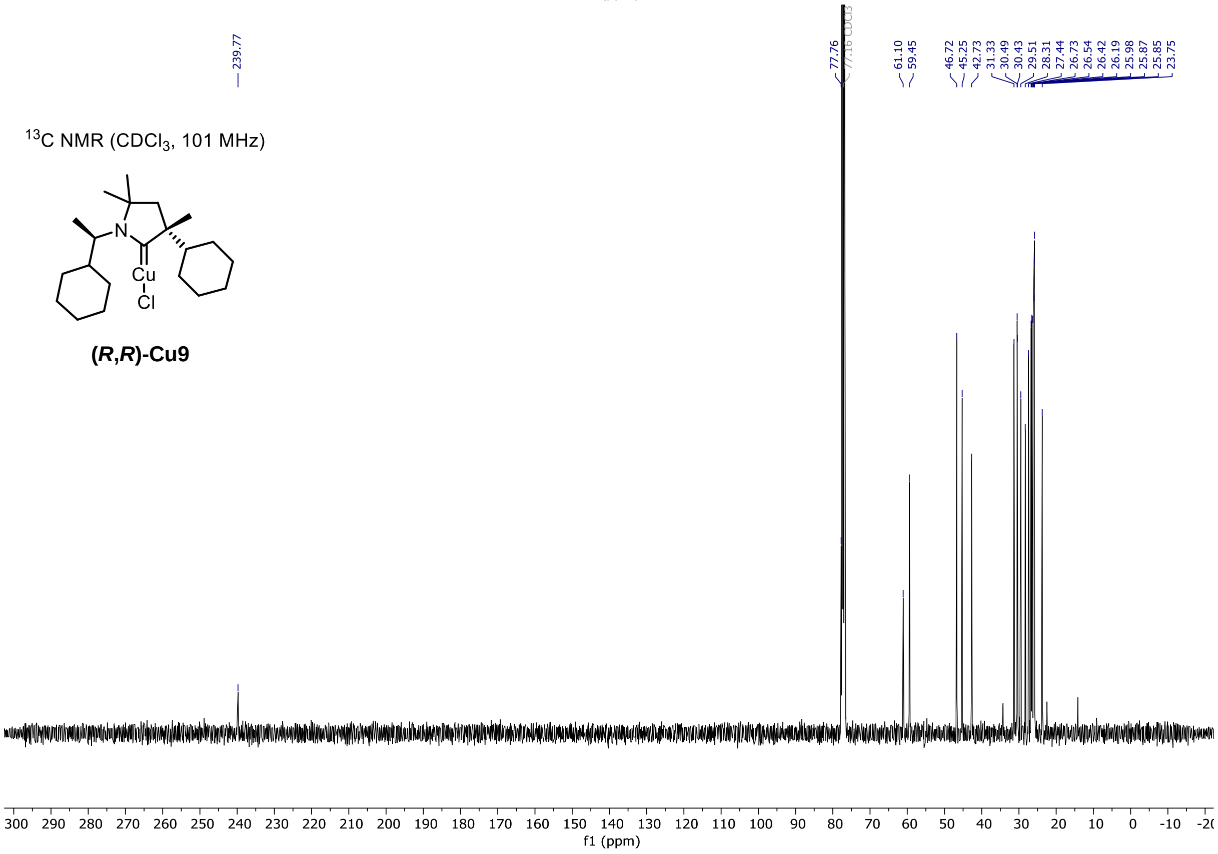

^{13}C NMR (CDCl$_3$, 101 MHz)
Cu
Cl
N
(*R*,*R*)-Cu9
f1 (ppm)

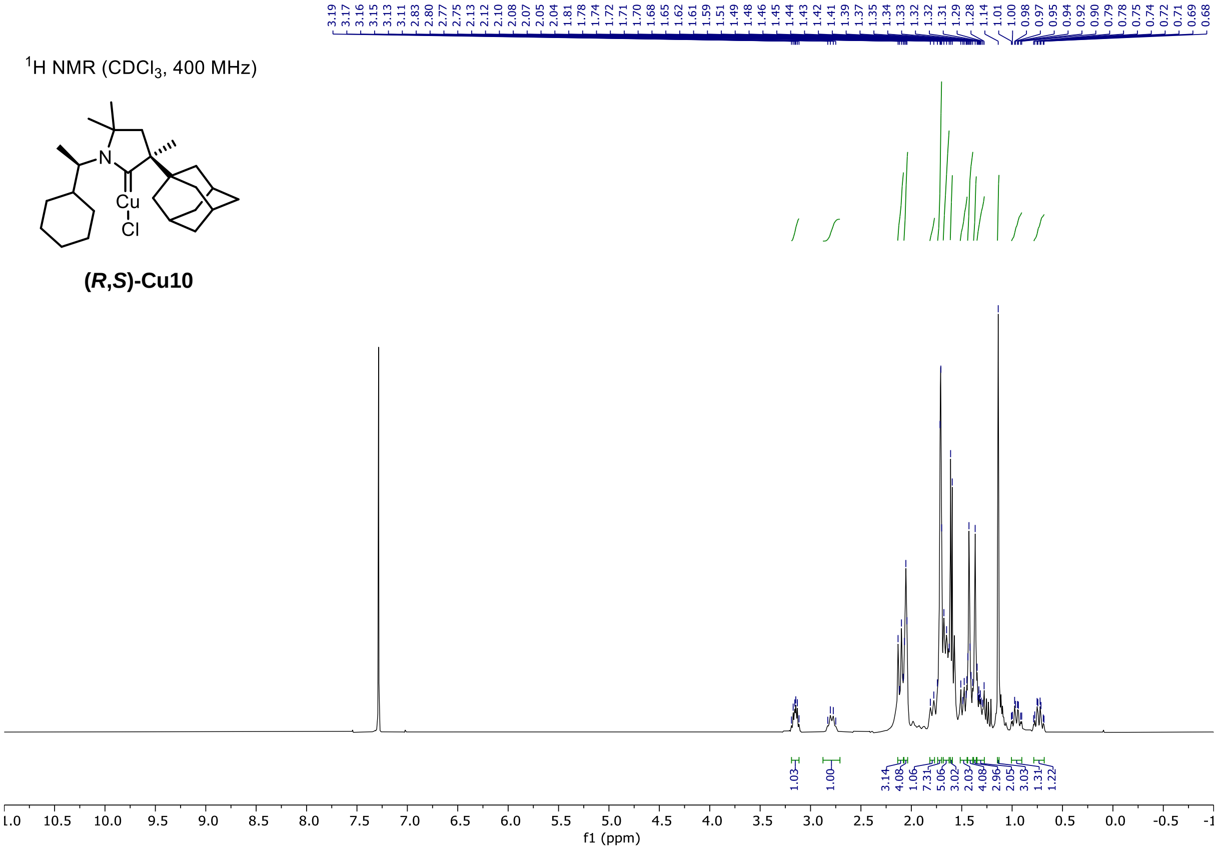

1H NMR (CDCl3, 400 MHz)
(R,S)-Cu10
3.19 3.17 3.16 3.15 3.13 3.11 2.83 2.80 2.77 2.75 2.13 2.12 2.10 2.08 2.07 2.05 2.04 1.81 1.78 1.74 1.72 1.71 1.70 1.68 1.65 1.62 1.61 1.59 1.51 1.49 1.48 1.46 1.45 1.44 1.43 1.42 1.41 1.39 1.37 1.35 1.34 1.33 1.32 1.32 1.31 1.29 1.28 1.14 1.01 1.00 0.98 0.97 0.95 0.94 0.92 0.90 0.79 0.78 0.75 0.74 0.72 0.71 0.69 0.68
1.03 1.00 3.14 4.08 1.06 7.31 5.06 3.02 2.03 4.08 2.96 2.05 3.03 1.31 1.22
f1 (ppm)

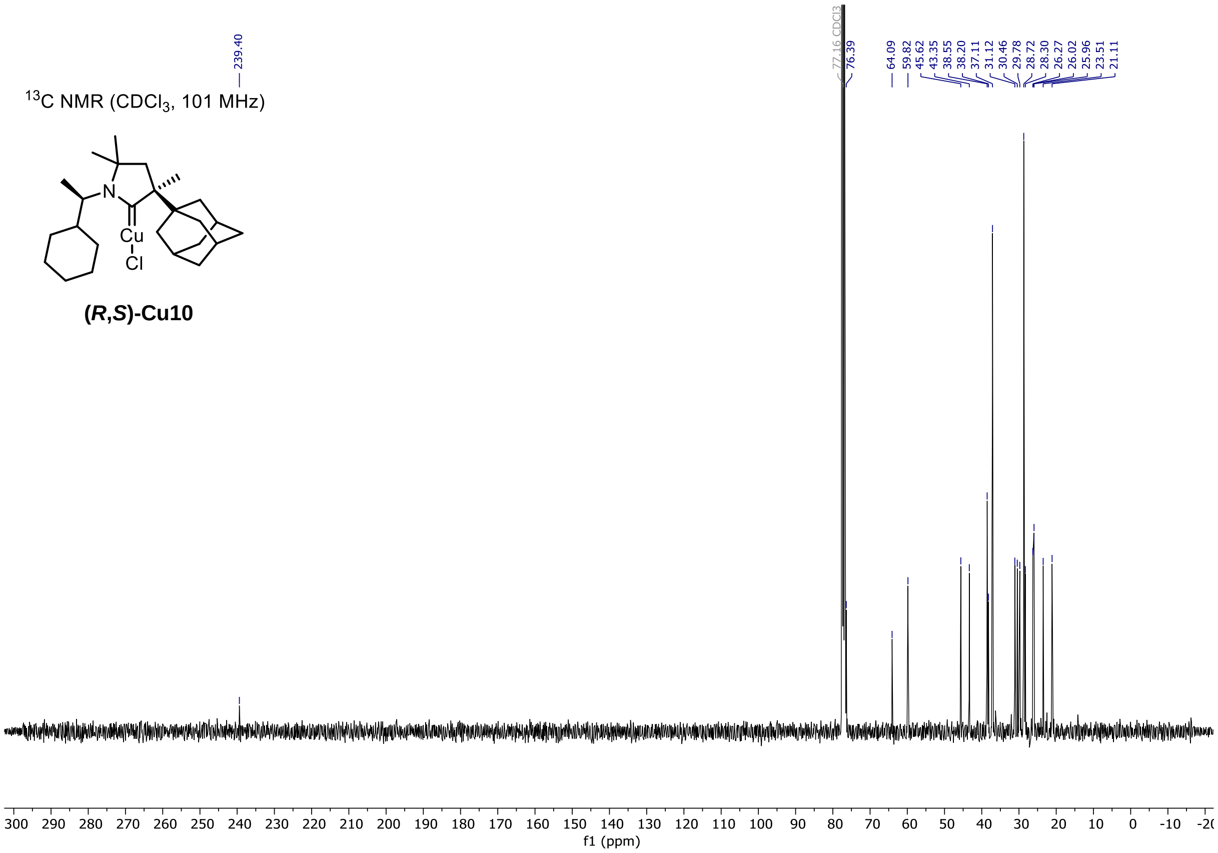

13C NMR (CDCl3, 101 MHz)
(R,S)-Cu10
239.40
77.16 CDCl3
76.39
64.09 59.82 45.62 43.35 38.55 38.20 37.11 31.12 30.46 29.78 28.72 28.30 26.27 26.02 25.96 23.51 21.11
f1 (ppm)

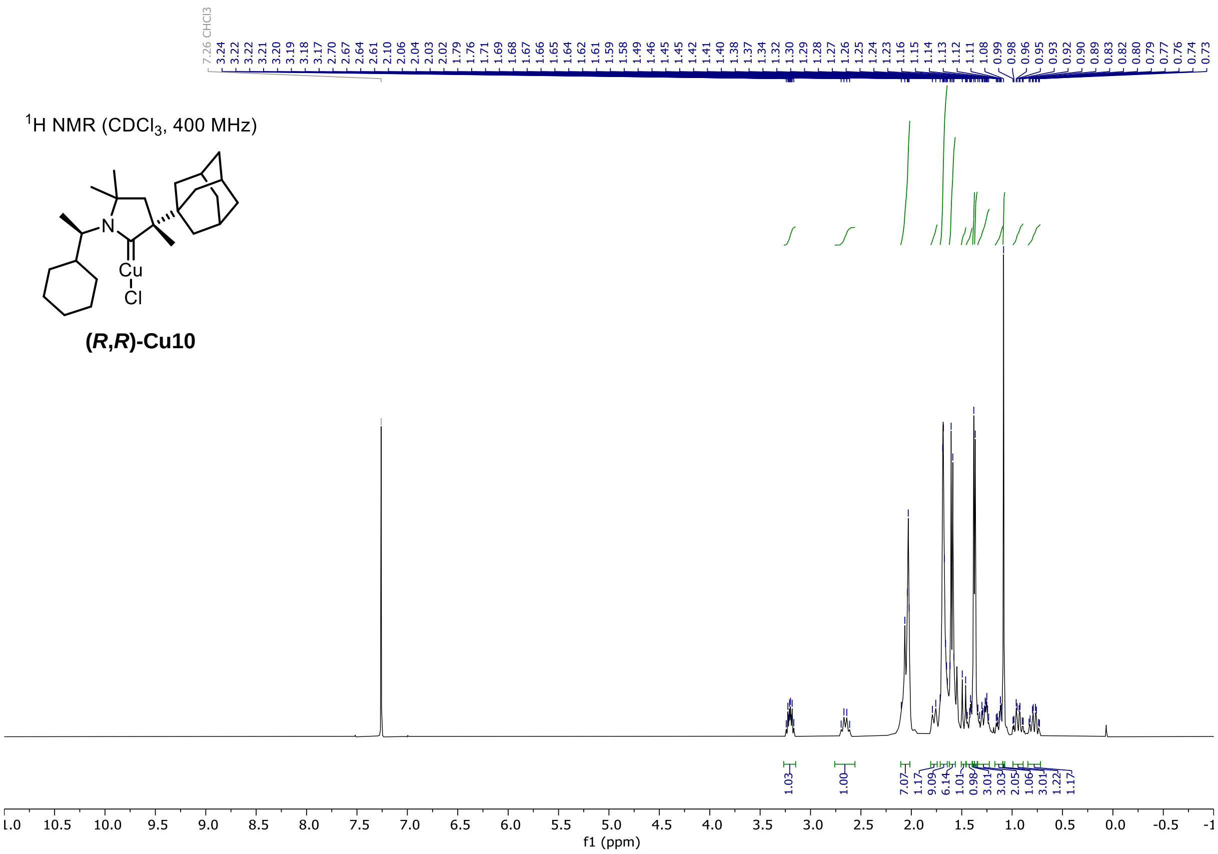
^{1}H NMR ($CDCl_3$, 400 MHz)
(R,R)-Cu10
7.26 CHCl3
3.24 3.22 3.22 3.21 3.20 3.19 3.18 3.17 2.70 2.67 2.64 2.61 2.10 2.06 2.04 2.03 2.02 1.79 1.76 1.71 1.69 1.68 1.67 1.66 1.65 1.64 1.62 1.61 1.59 1.58 1.49 1.46 1.45 1.45 1.42 1.41 1.40 1.38 1.37 1.34 1.32 1.30 1.29 1.28 1.27 1.26 1.25 1.24 1.23 1.16 1.15 1.14 1.13 1.12 1.11 1.08 0.99 0.98 0.96 0.95 0.93 0.92 0.90 0.89 0.83 0.82 0.80 0.79 0.77 0.76 0.74 0.73
1.03 1.00 7.07 1.17 9.09 6.14 1.01 0.98 3.01 3.03 2.05 1.06 3.01 1.22 1.17
f1 (ppm)

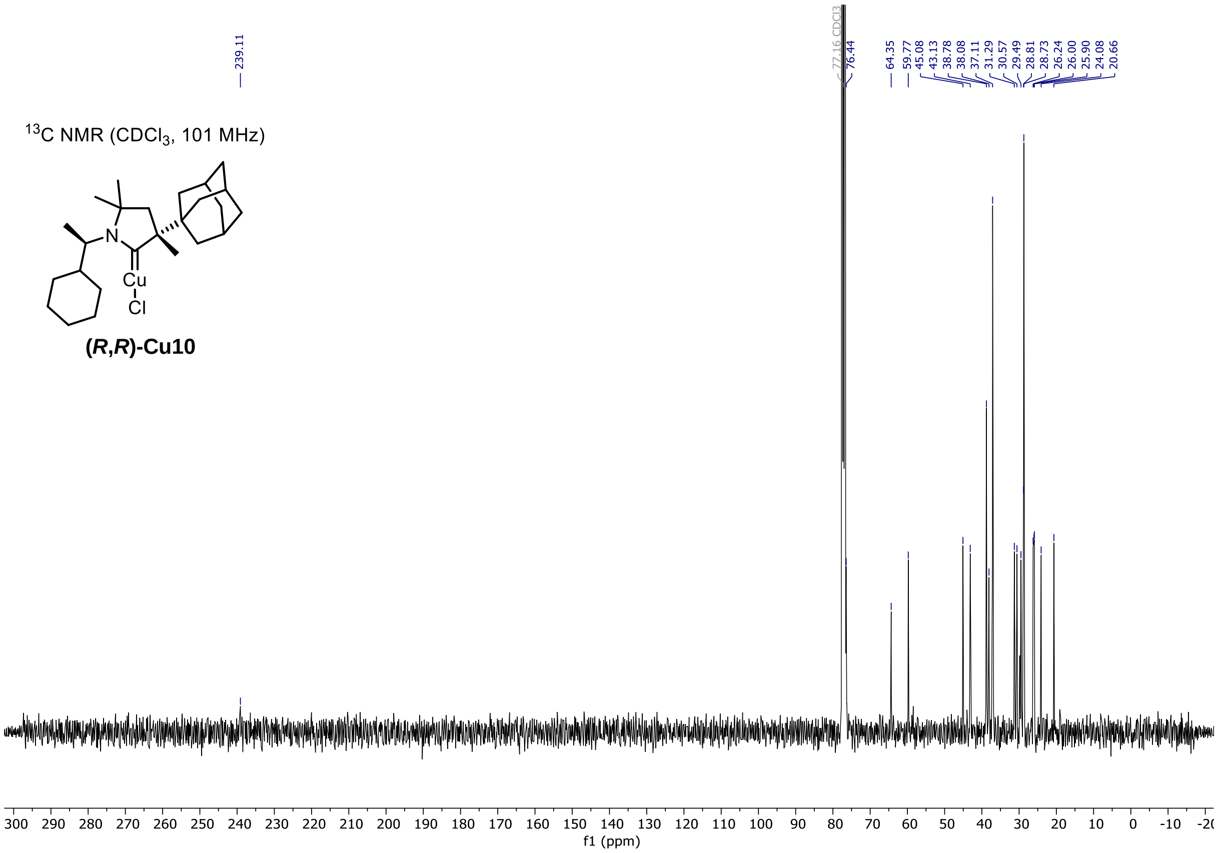
^{13}C NMR ($CDCl_3$, 101 MHz)
(R,R)-Cu10
239.11
77.16 CDCl3
76.44
64.35 59.77 45.08 43.13 38.78 38.08 37.11 31.29 30.57 29.49 28.81 28.73 26.24 26.00 25.90 24.08 20.66
f1 (ppm)

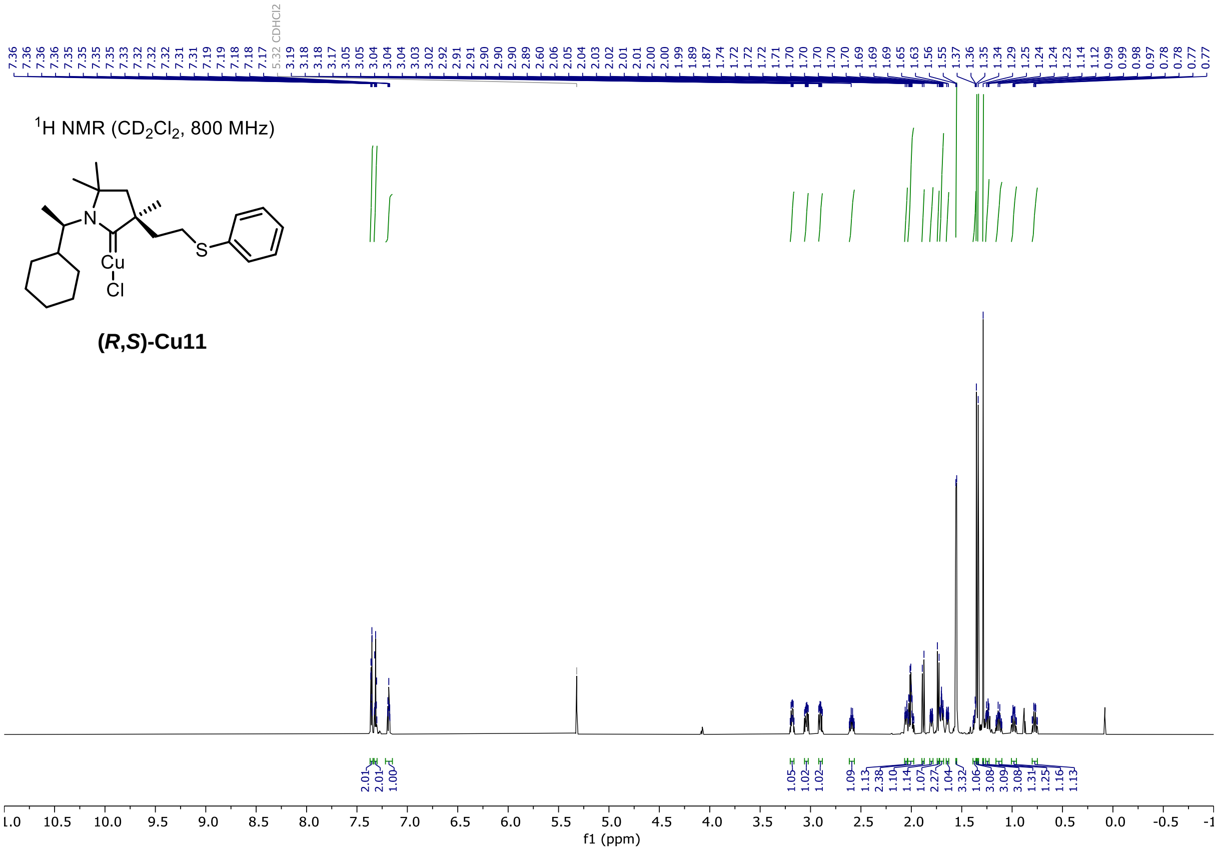
7.36 7.36 7.36 7.35 7.35 7.35 7.33 7.32 7.32 7.32 7.31 7.31 7.19 7.19 7.18 7.17 5.32 CDHCl2 3.19 3.18 3.18 3.17 3.05 3.04 3.04 3.03 3.02 2.92 2.91 2.91 2.90 2.90 2.89 2.60 2.06 2.05 2.04 2.03 2.02 2.01 2.01 2.00 2.00 1.99 1.89 1.87 1.74 1.72 1.72 1.72 1.71 1.70 1.70 1.70 1.70 1.69 1.69 1.69 1.65 1.63 1.56 1.55 1.37 1.36 1.35 1.34 1.29 1.25 1.24 1.24 1.23 1.14 1.12 0.99 0.99 0.98 0.97 0.78 0.77 0.77
1H NMR (CD2Cl2, 800 MHz)
N
Cu
Cl
S
(R,S)-Cu11
2.01 2.01 1.00
1.05 1.02 1.02 1.09 1.13 2.38 1.10 1.14 1.07 2.27 1.04 3.32 1.06 3.08 3.09 3.08 1.31 1.25 1.16 1.13
f1 (ppm)

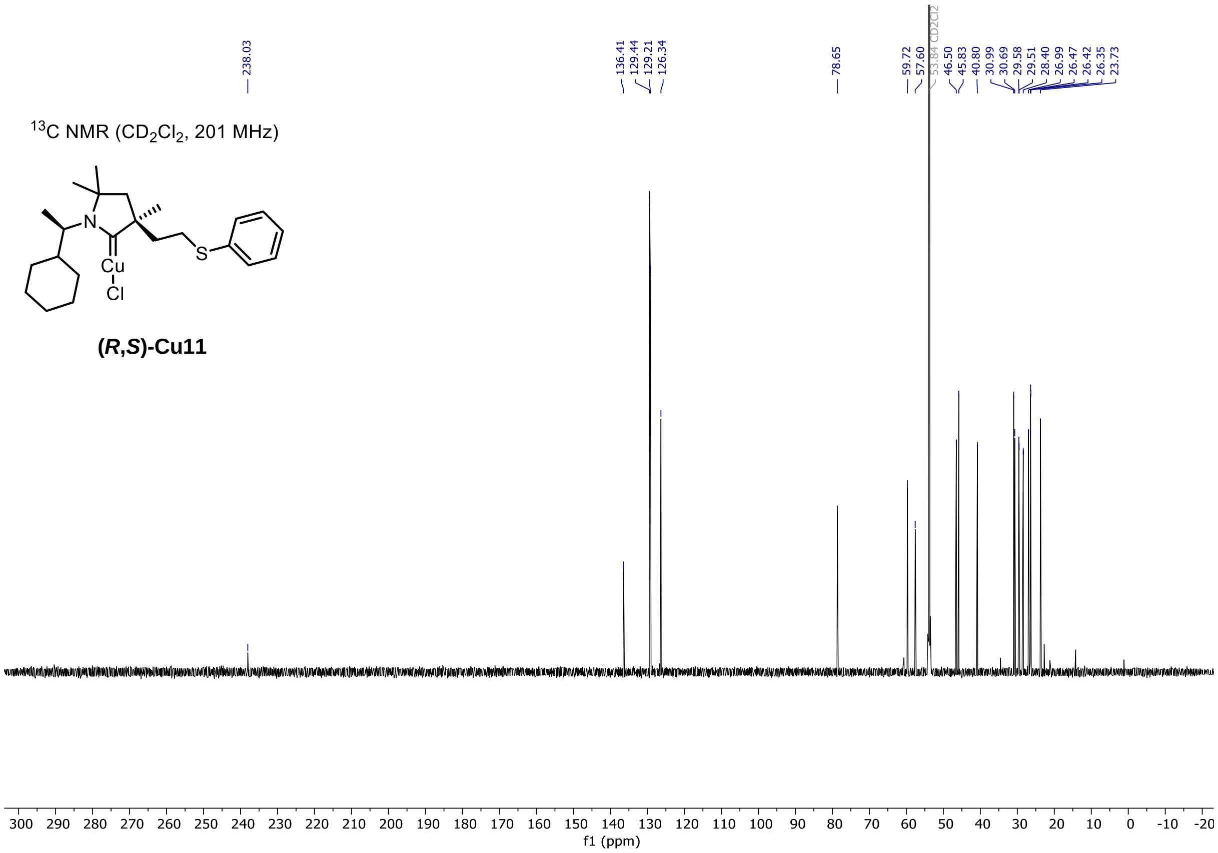
238.03
136.41 129.44 129.21 126.34
78.65
59.72 57.60 53.84 CD2Cl2 46.50 45.83 40.80 30.99 30.69 29.58 29.51 28.40 26.99 26.47 26.42 26.35 23.73
13C NMR (CD2Cl2, 201 MHz)
N
Cu
Cl
S
(R,S)-Cu11
f1 (ppm)

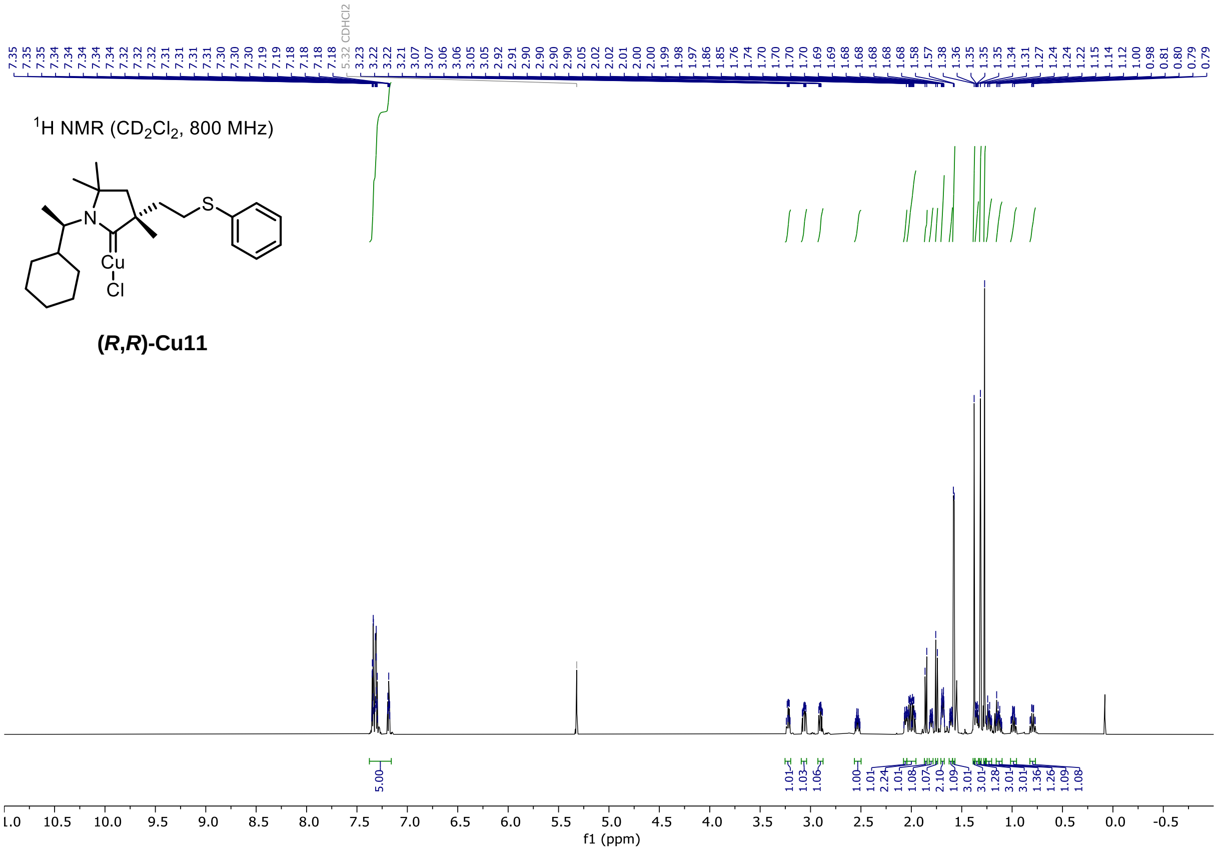

1H NMR (CD2Cl2, 800 MHz)
Cu
Cl
N
S
(R,R)-Cu11
5.32 CDHCl2
f1 (ppm)

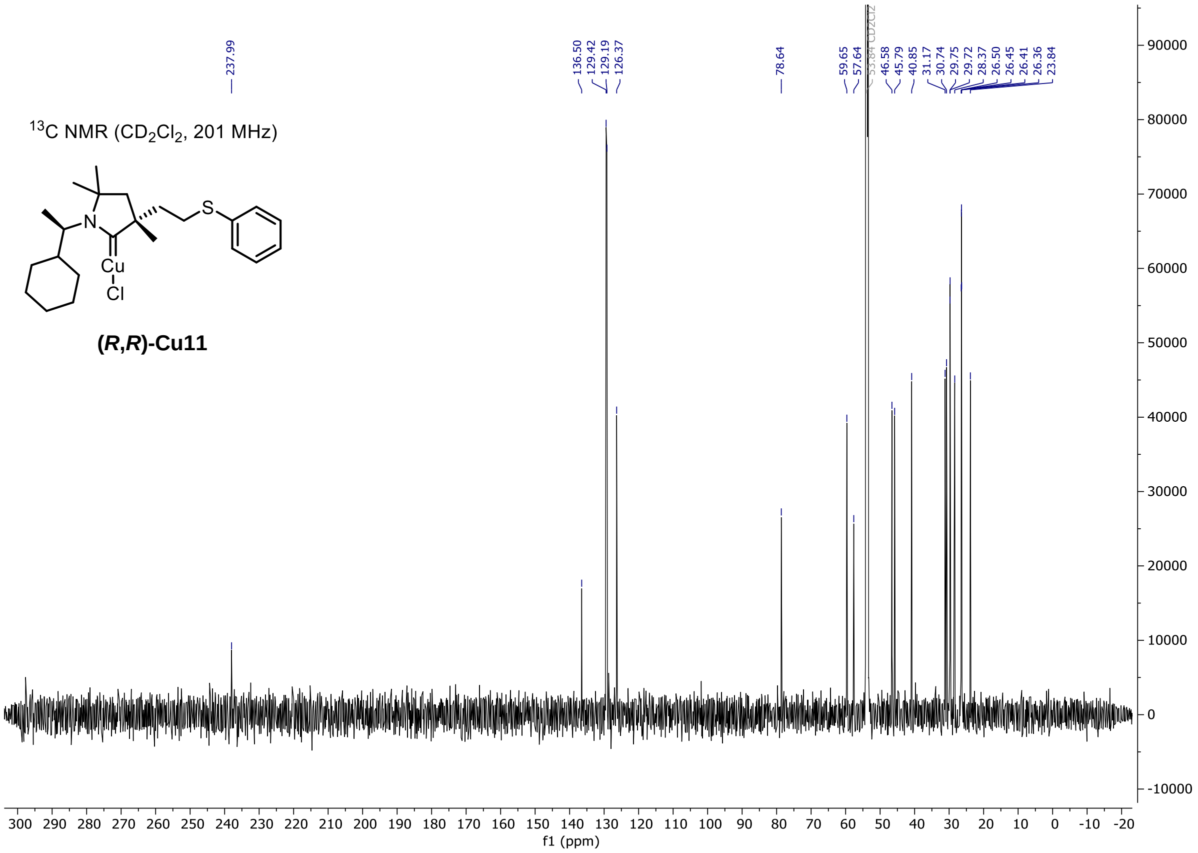

13C NMR (CD2Cl2, 201 MHz)
Cu
Cl
N
S
(R,R)-Cu11
237.99
136.50
129.42
129.19
126.37
78.64
59.65
57.64
53.84 CD2Cl2
46.58
45.79
40.85
31.17
30.74
29.75
29.72
28.37
26.50
26.45
26.41
26.36
23.84
f1 (ppm)

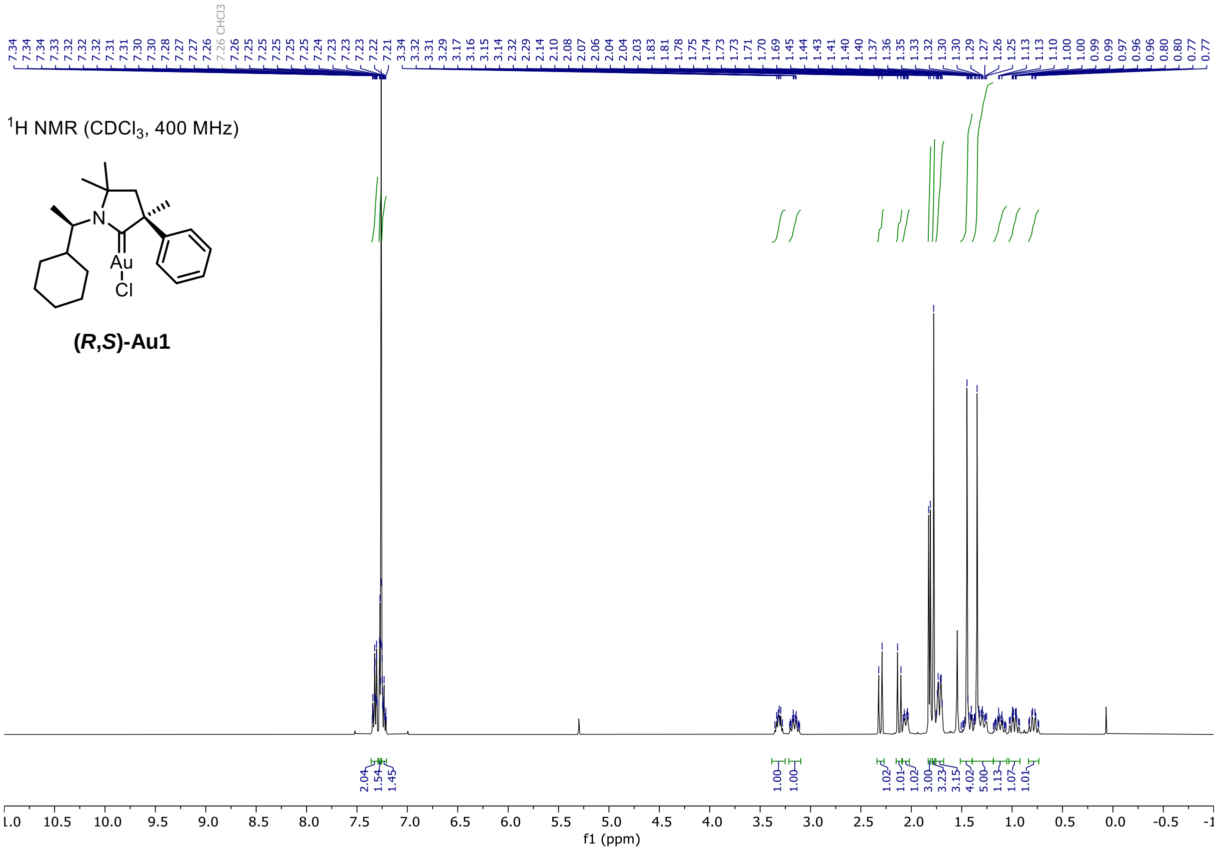

^{1}H NMR (CDCl$_3$, 400 MHz)
(*R*,*S*)-Au1
Au
Cl
N
f1 (ppm)

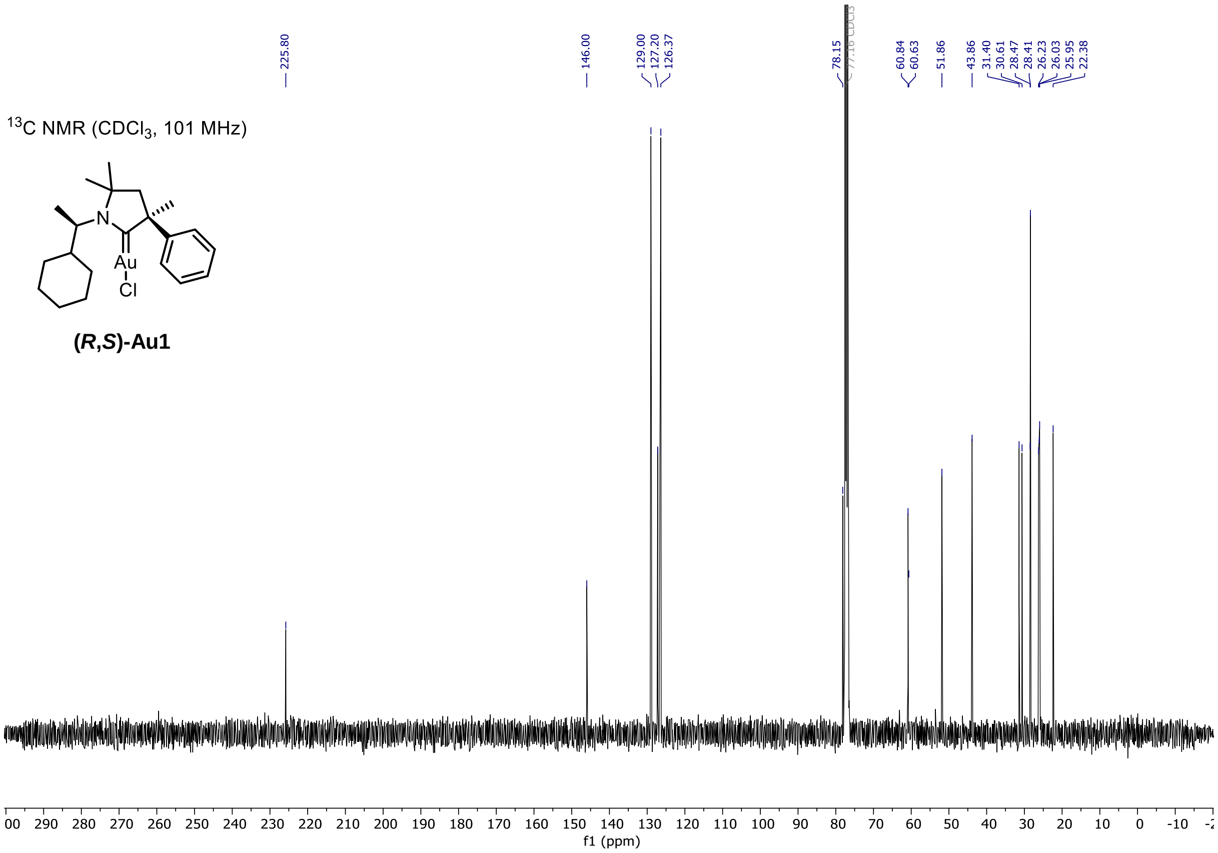

^{13}C NMR (CDCl$_3$, 101 MHz)
(*R*,*S*)-Au1
225.80
146.00
129.00
127.20
126.37
78.15
60.84
60.63
51.86
43.86
31.40
30.61
28.47
28.41
26.23
26.03
25.95
22.38
f1 (ppm)

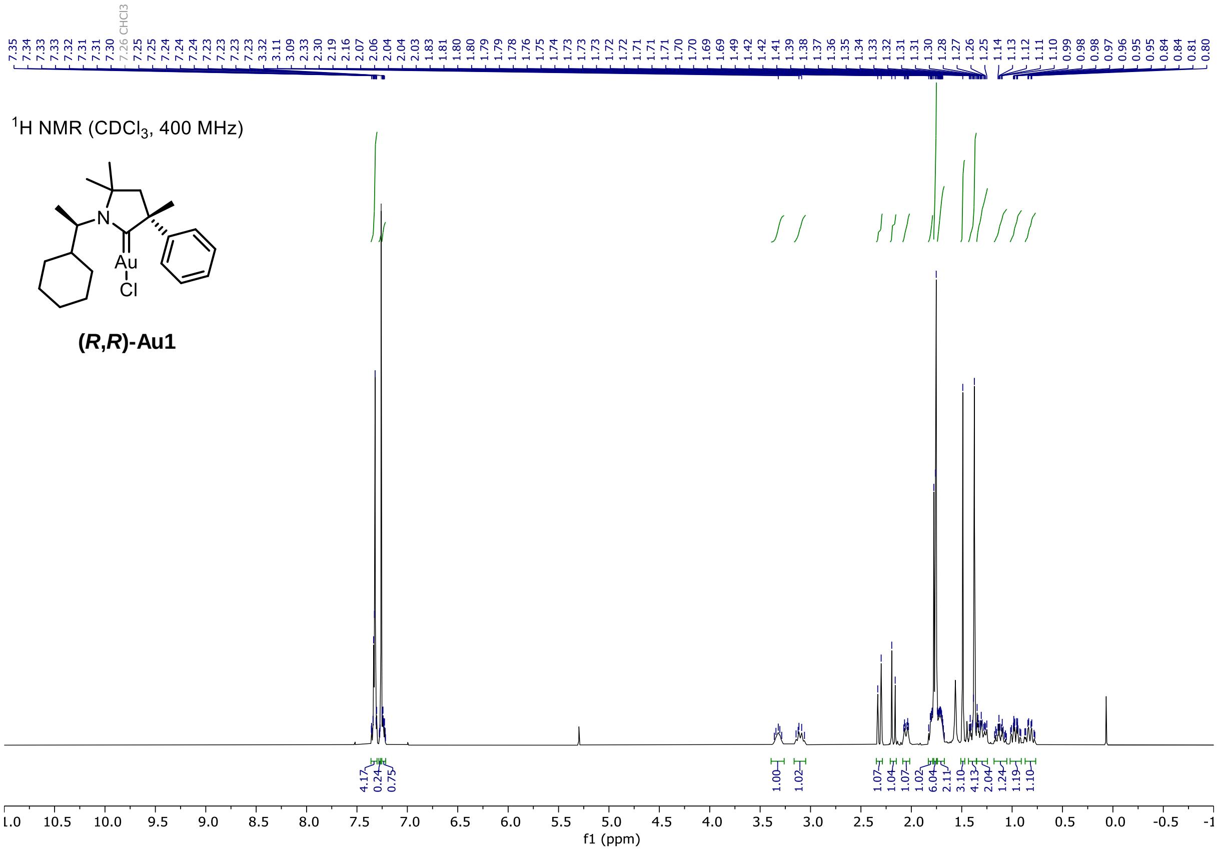

1H NMR (CDCl3, 400 MHz)
Au
Cl
(R,R)-Au1
f1 (ppm)

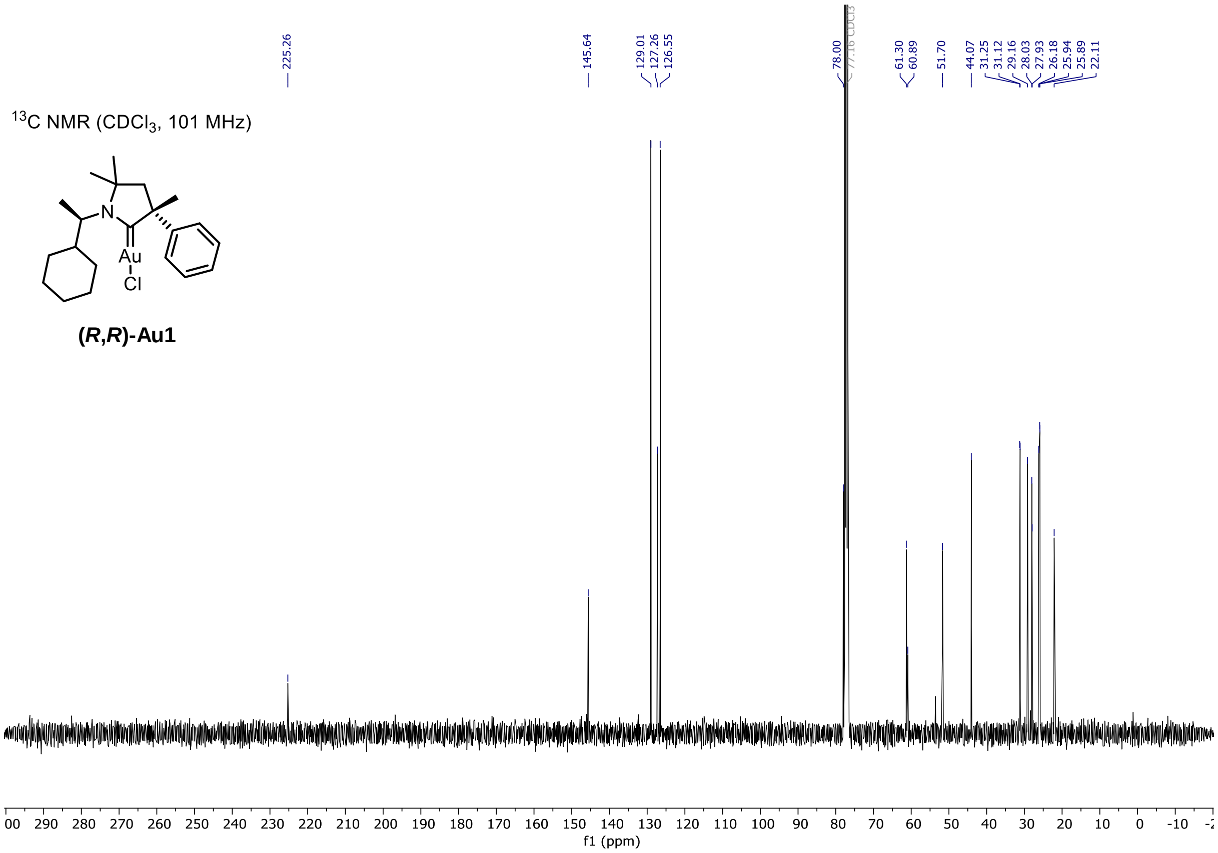

13C NMR (CDCl3, 101 MHz)
225.26
145.64
129.01
127.26
126.55
78.00
77.16 CDCl3
61.30
60.89
51.70
44.07
31.25
31.12
29.16
28.03
27.93
26.18
25.94
25.89
22.11
Au
Cl
(R,R)-Au1
f1 (ppm)

^{1}H NMR (CD_2Cl_2, 400 MHz)

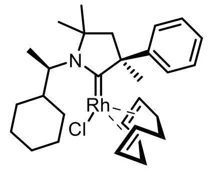

(*R*,*S*)-Rh1

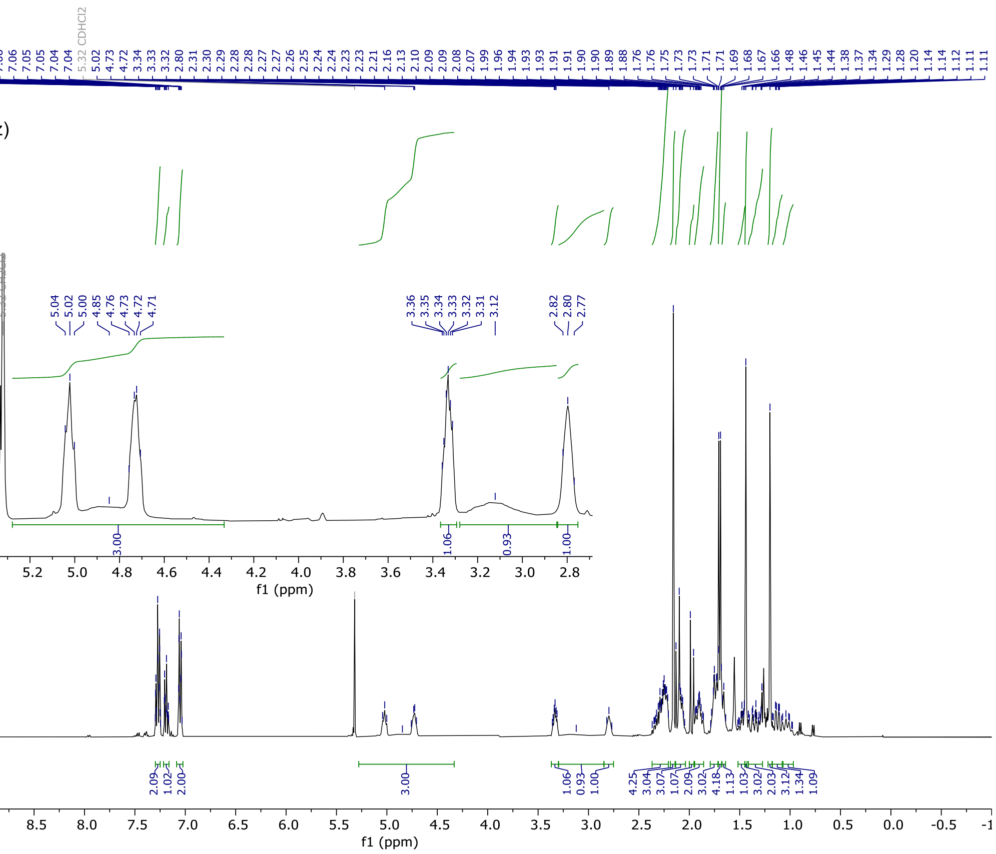

^{13}C NMR (CD_2Cl_2, 101 MHz)

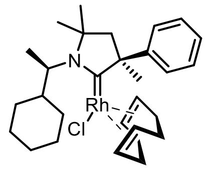

(*R*,*S*)-Rh1

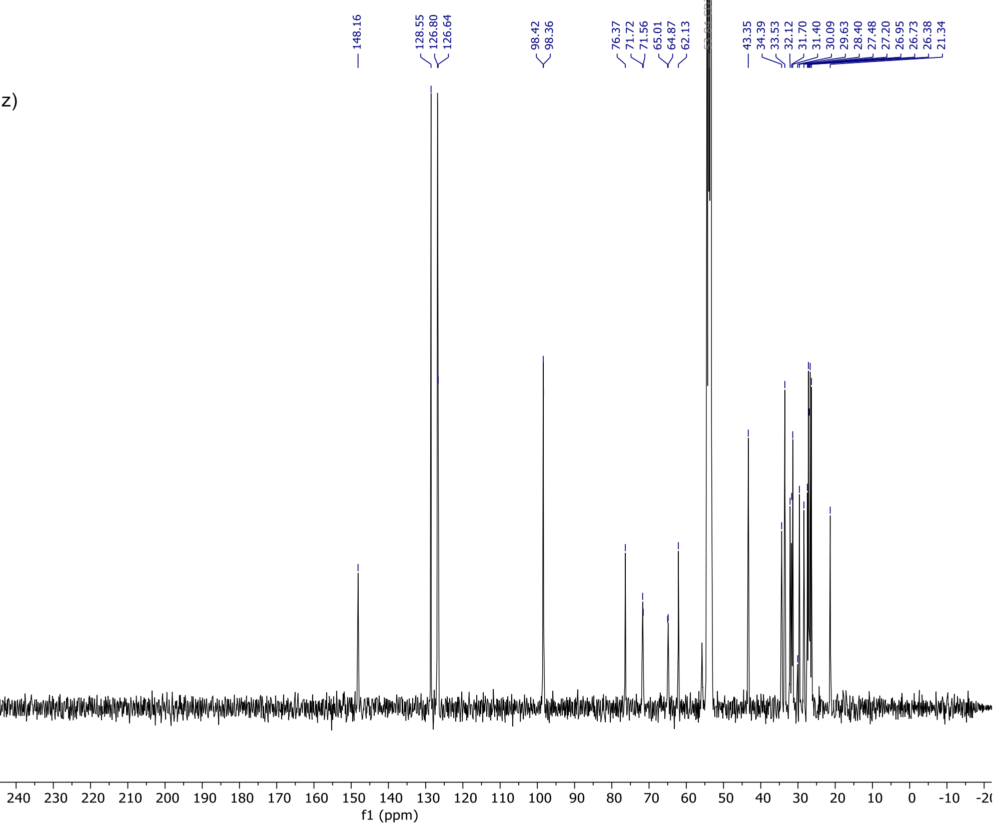

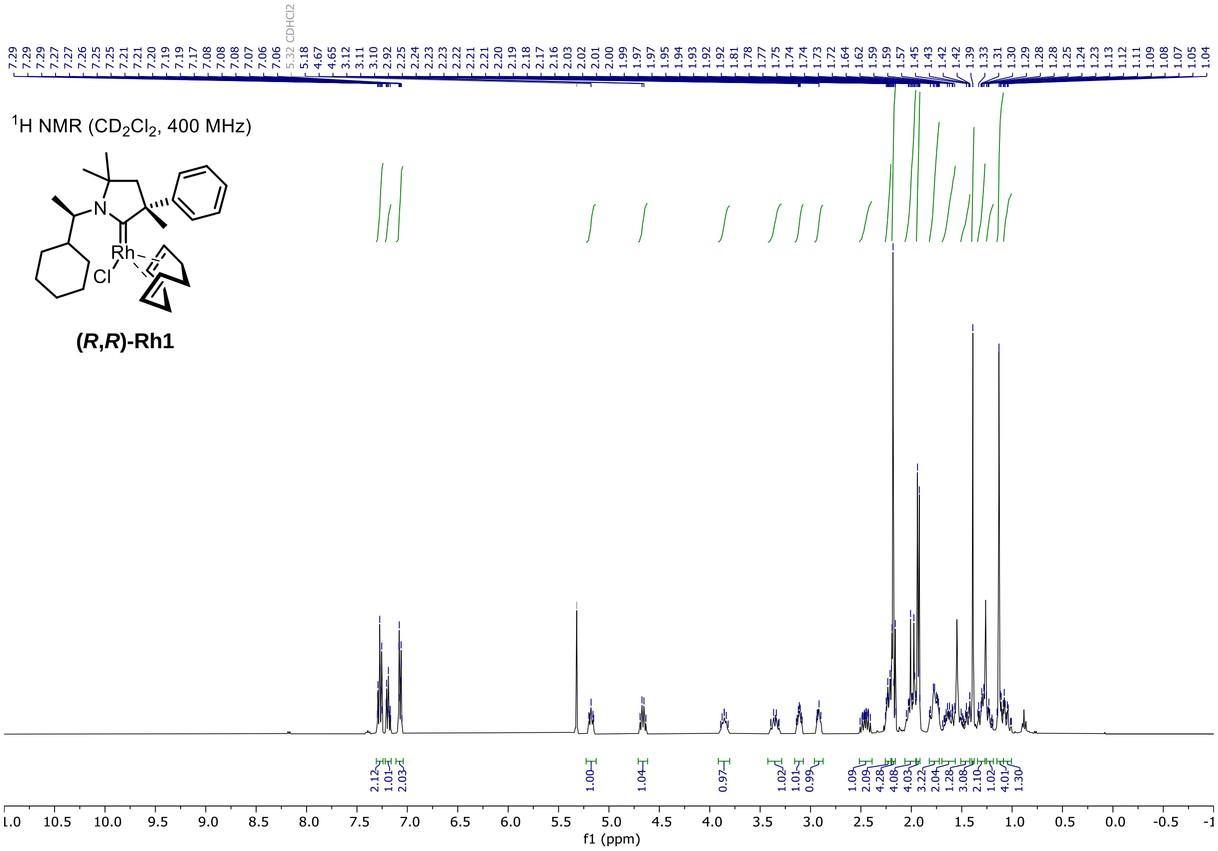
7.29 7.29 7.29 7.27 7.26 7.25 7.25 7.21 7.21 7.20 7.19 7.19 7.17 7.08 7.08 7.08 7.07 7.06 7.06 5.32 CDHCl2 5.18 4.67 4.65 3.12 3.11 3.10 2.92 2.25 2.24 2.23 2.23 2.22 2.21 2.21 2.20 2.19 2.18 2.17 2.16 2.03 2.02 2.01 2.00 1.99 1.97 1.97 1.95 1.94 1.93 1.92 1.92 1.81 1.78 1.77 1.75 1.74 1.74 1.73 1.72 1.64 1.62 1.59 1.59 1.57 1.45 1.43 1.42 1.42 1.39 1.33 1.31 1.30 1.29 1.28 1.25 1.24 1.23 1.13 1.12 1.11 1.09 1.08 1.07 1.05 1.04
^{1}H NMR (CD_2Cl_2, 400 MHz)
N
Rh
Cl
(*R*,*R*)-Rh1
2.12 1.01 2.03 1.00 1.04 0.97 1.02 1.01 0.99 1.09 2.09 4.28 4.08 4.03 3.22 2.04 1.28 3.08 2.10 1.02 4.01 1.30
f1 (ppm)

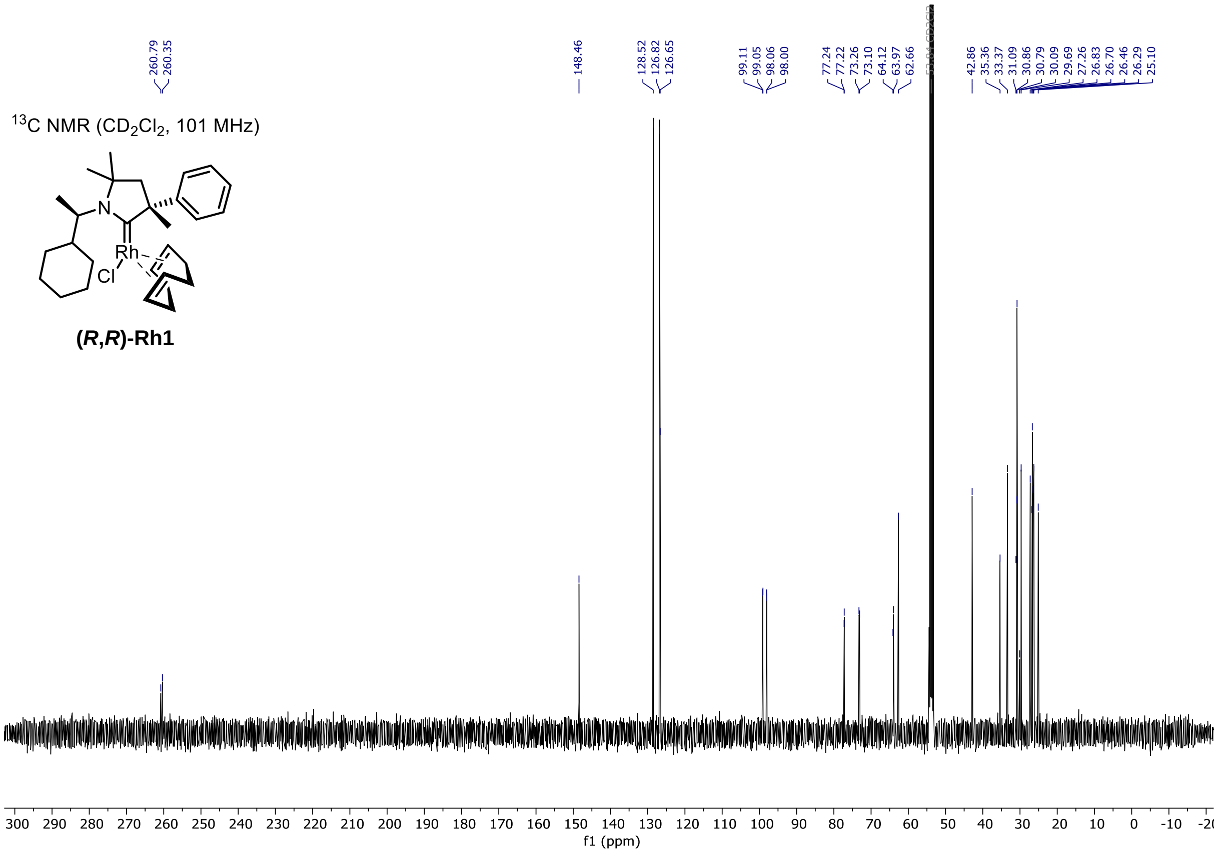
260.79 260.35 148.46 128.52 126.82 126.65 99.11 99.05 98.06 98.00 77.24 77.22 73.26 73.10 64.12 63.97 62.66 42.86 35.36 33.37 31.09 30.86 30.79 30.09 29.69 27.26 26.83 26.70 26.46 26.29 25.10
^{13}C NMR (CD_2Cl_2, 101 MHz)
N
Rh
Cl
(*R*,*R*)-Rh1
f1 (ppm)

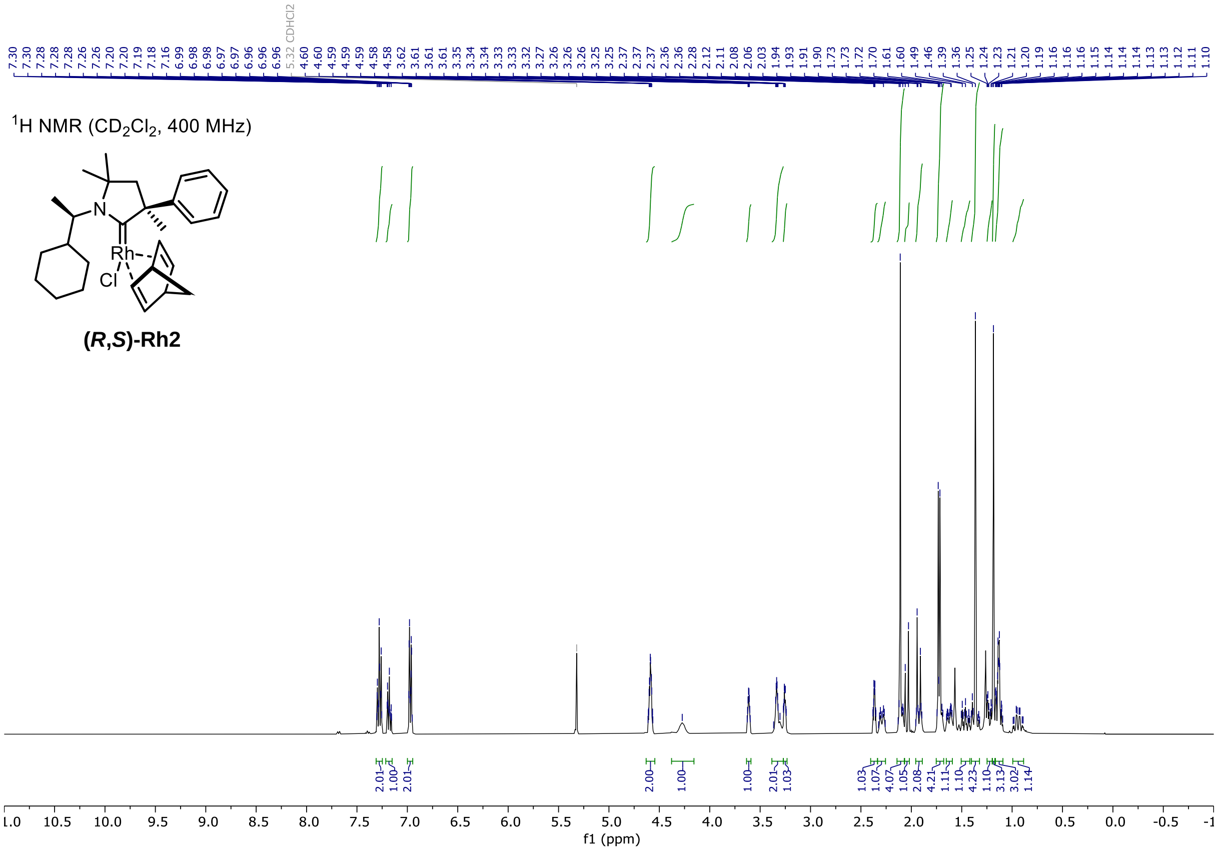

1H NMR (CD2Cl2, 400 MHz)
(R,S)-Rh2
f1 (ppm)

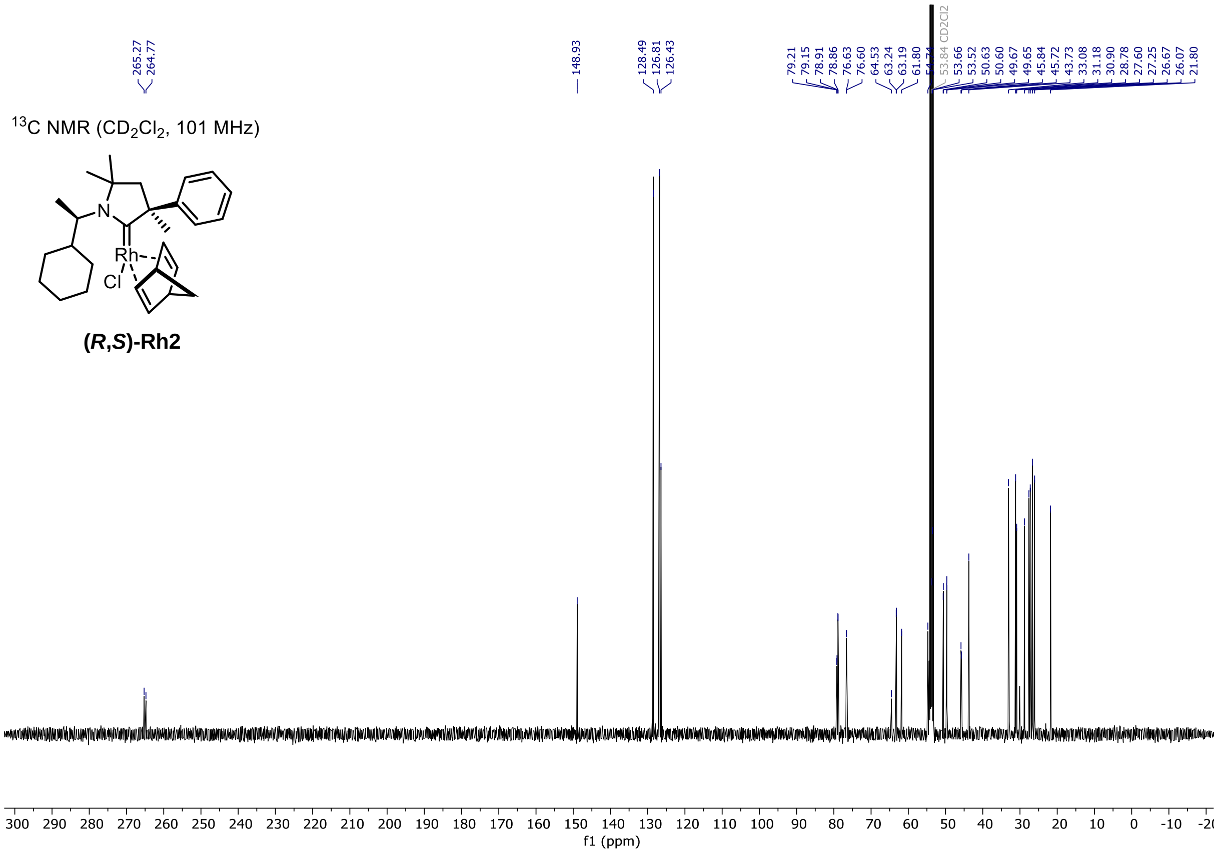

13C NMR (CD2Cl2, 101 MHz)
(R,S)-Rh2
f1 (ppm)

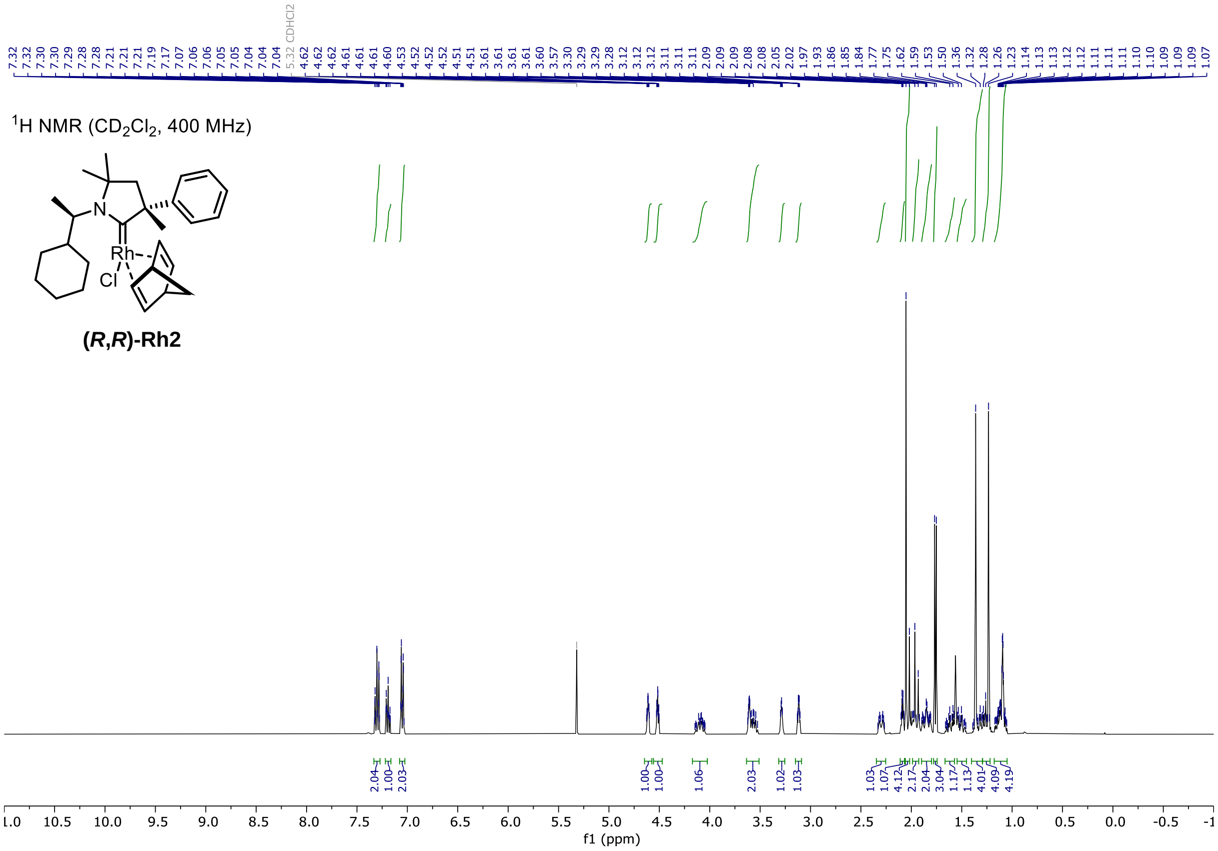
7.32 7.32 7.30 7.30 7.29 7.28 7.28 7.21 7.21 7.21 7.19 7.17 7.07 7.06 7.06 7.05 7.05 7.04 7.04 7.04 5.32 CDHCl2 4.62 4.62 4.62 4.61 4.61 4.61 4.60 4.53 4.52 4.52 4.52 4.51 4.51 3.61 3.61 3.61 3.60 3.57 3.30 3.29 3.29 3.28 3.12 3.12 3.12 3.11 3.11 3.11 2.09 2.09 2.09 2.08 2.08 2.05 2.02 1.97 1.93 1.86 1.85 1.84 1.77 1.75 1.62 1.59 1.53 1.50 1.36 1.32 1.28 1.26 1.23 1.14 1.13 1.13 1.12 1.12 1.11 1.11 1.11 1.10 1.09 1.09 1.09 1.07
^{1}H NMR (CD_2Cl_2, 400 MHz)
N
Rh
Cl
(*R*,*R*)-Rh2
f1 (ppm)

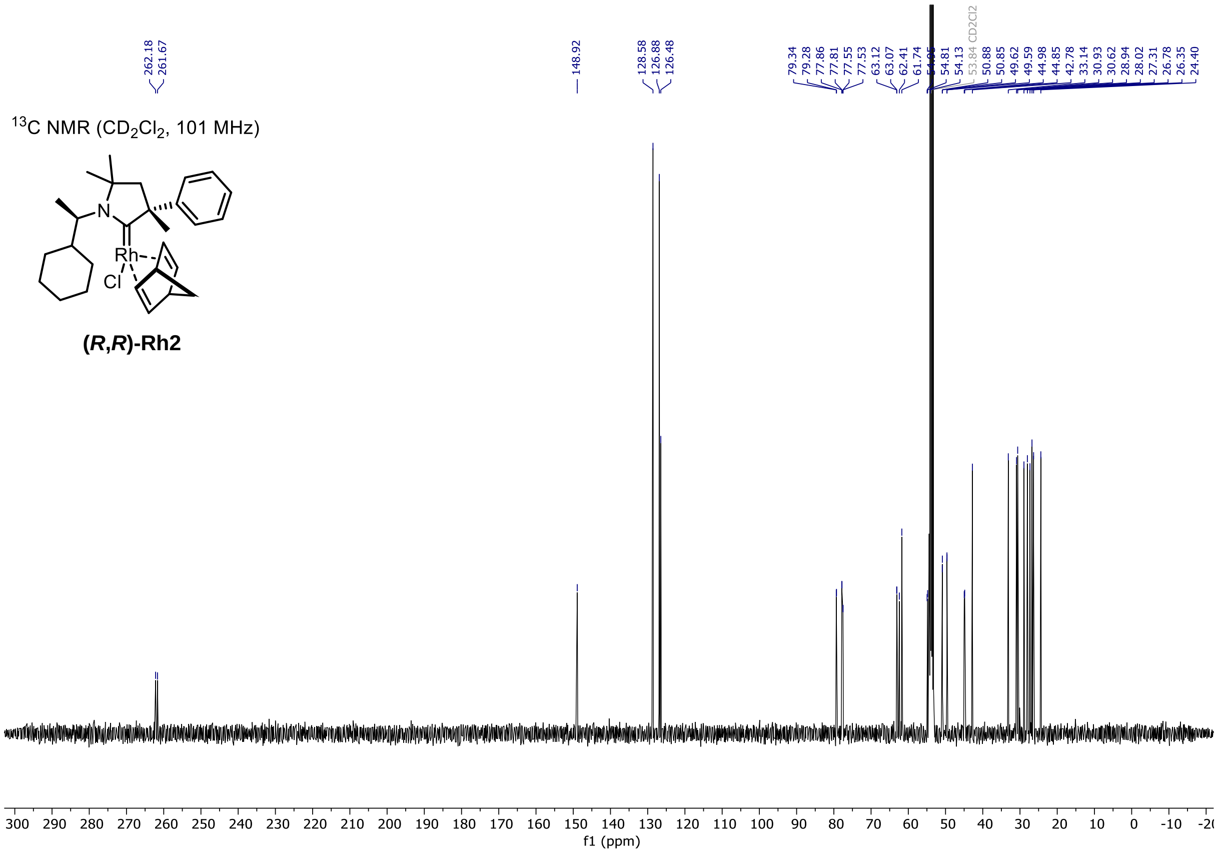
262.18 261.67 148.92 128.58 126.88 126.48 79.34 79.28 77.86 77.81 77.55 77.53 63.12 63.07 62.41 61.74 54.81 54.13 53.84 CD2Cl2 50.88 50.85 49.62 49.59 44.98 44.85 42.78 33.14 30.93 30.62 28.94 28.02 27.31 26.78 26.35 24.40
^{13}C NMR (CD_2Cl_2, 101 MHz)
N
Rh
Cl
(*R*,*R*)-Rh2
f1 (ppm)

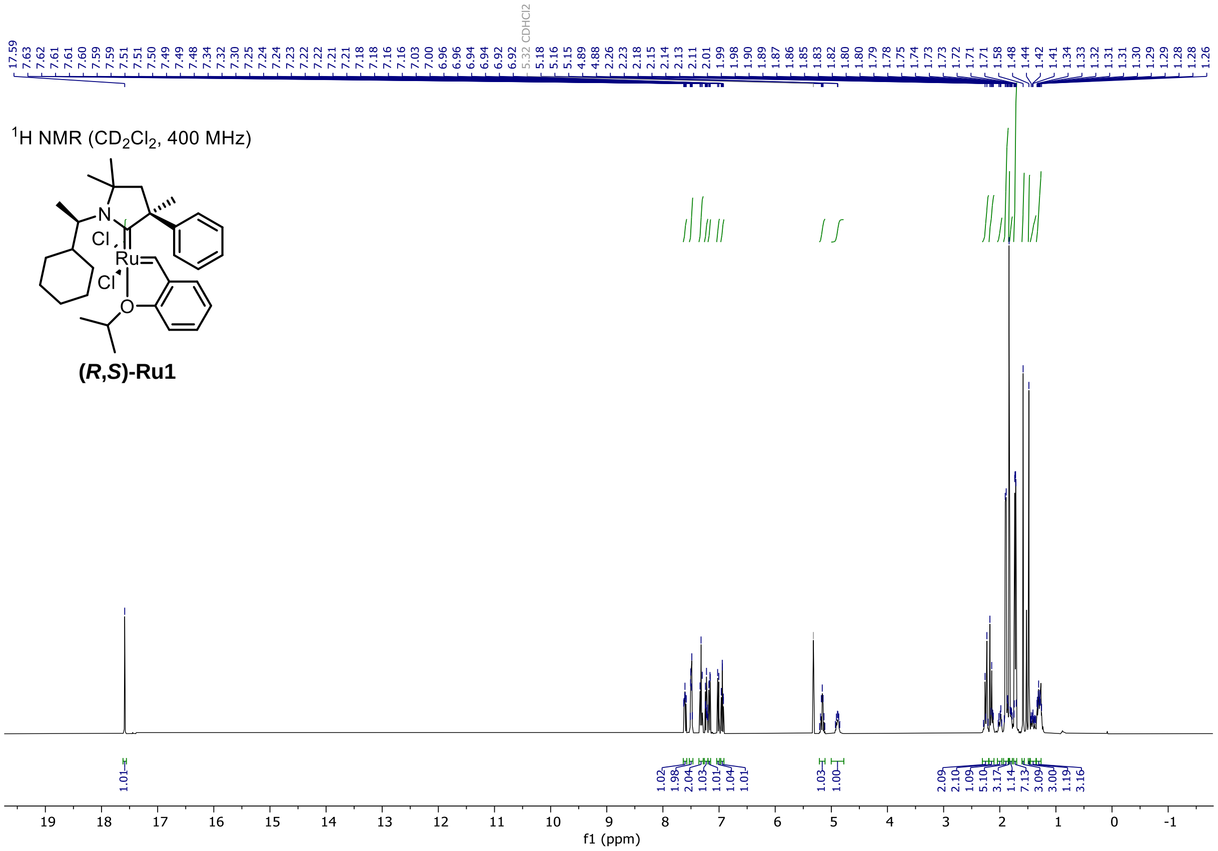
17.59 7.63 7.62 7.61 7.60 7.59 7.59 7.51 7.50 7.49 7.49 7.48 7.34 7.32 7.30 7.25 7.24 7.24 7.23 7.22 7.22 7.21 7.21 7.18 7.18 7.16 7.16 7.03 7.00 6.96 6.96 6.94 6.94 6.92 6.92 5.32 CDHCl2 5.18 5.16 5.15 4.89 4.88 2.26 2.23 2.18 2.15 2.14 2.13 2.11 2.01 1.99 1.98 1.90 1.89 1.87 1.86 1.85 1.83 1.82 1.80 1.80 1.79 1.78 1.75 1.74 1.73 1.73 1.72 1.71 1.71 1.58 1.48 1.44 1.42 1.41 1.34 1.33 1.32 1.31 1.30 1.29 1.29 1.28 1.26
^{1}H NMR (CD_2Cl_2, 400 MHz)
N
Cl
Ru
Cl
O
(*R*,*S*)-Ru1
1.01
1.02 1.98 2.04 1.03 1.01 1.04 1.01
1.03 1.00
2.09 2.10 1.09 5.10 3.17 1.14 7.13 3.09 3.00 1.19 3.16
19 18 17 16 15 14 13 12 11 10 9 8 7 6 5 4 3 2 1 0 -1
f1 (ppm)

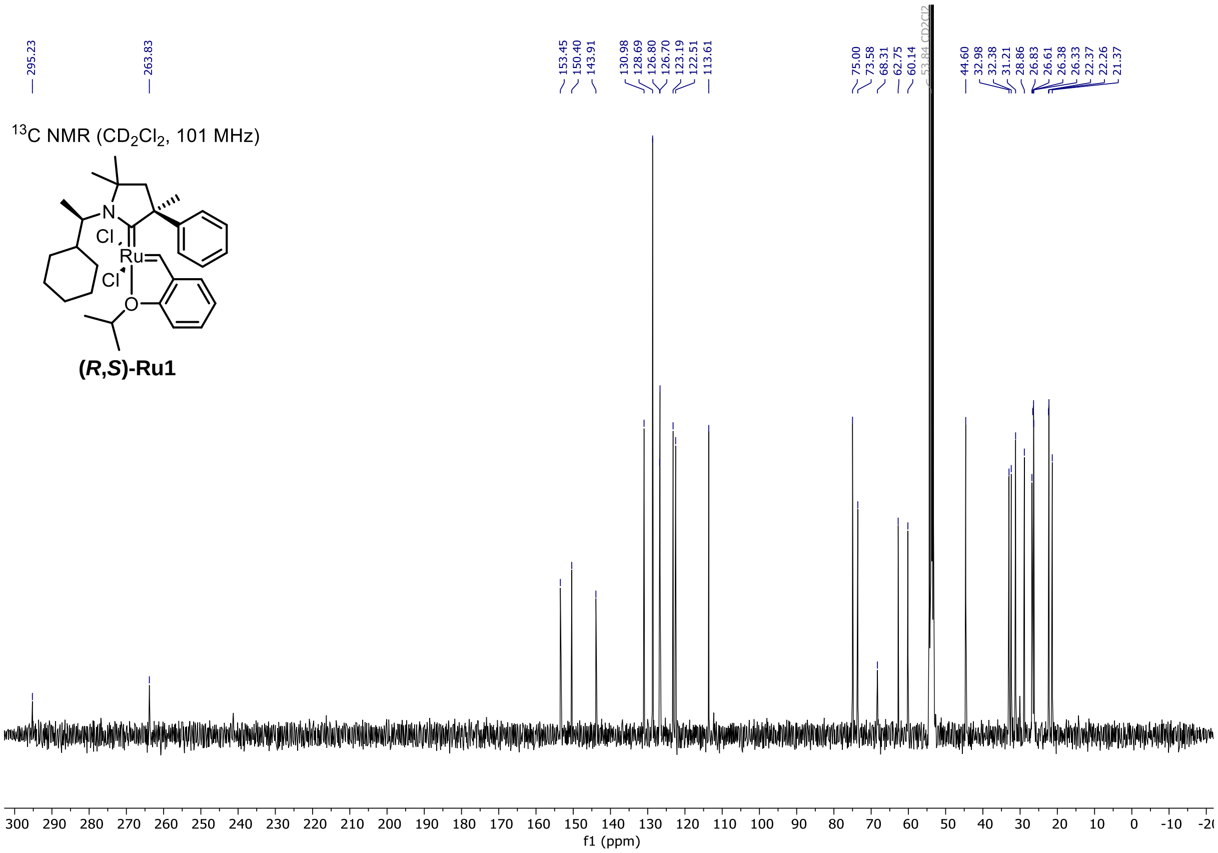
295.23 263.83 153.45 150.40 143.91 130.98 128.69 126.80 126.70 123.19 122.51 113.61 75.00 73.58 68.31 62.75 60.14 53.84 CD2Cl2 44.60 32.98 32.38 31.21 28.86 26.83 26.61 26.38 26.33 22.37 22.26 21.37
^{13}C NMR (CD_2Cl_2, 101 MHz)
N
Cl
Ru
Cl
O
(*R*,*S*)-Ru1
300 290 280 270 260 250 240 230 220 210 200 190 180 170 160 150 140 130 120 110 100 90 80 70 60 50 40 30 20 10 0 -10 -20
f1 (ppm)

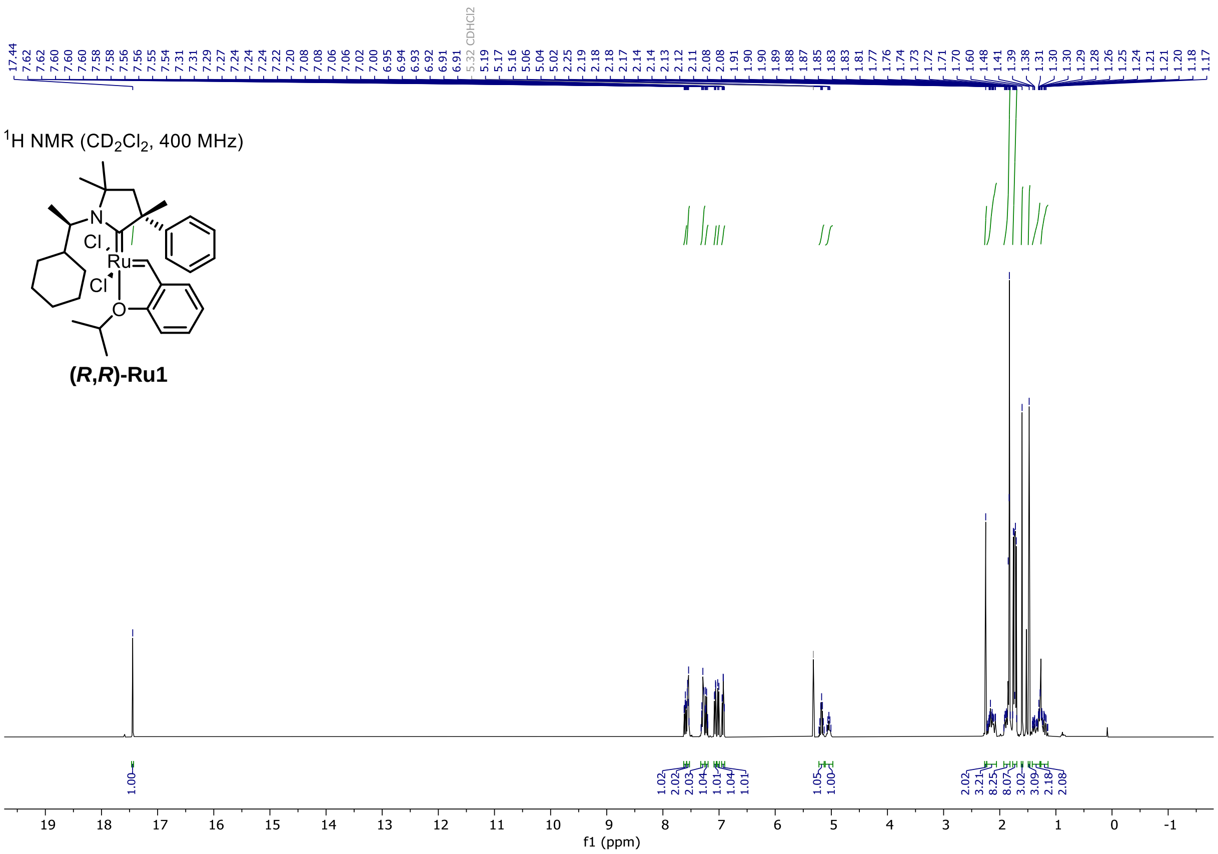
1H NMR (CD2Cl2, 400 MHz)
(R,R)-Ru1
f1 (ppm)

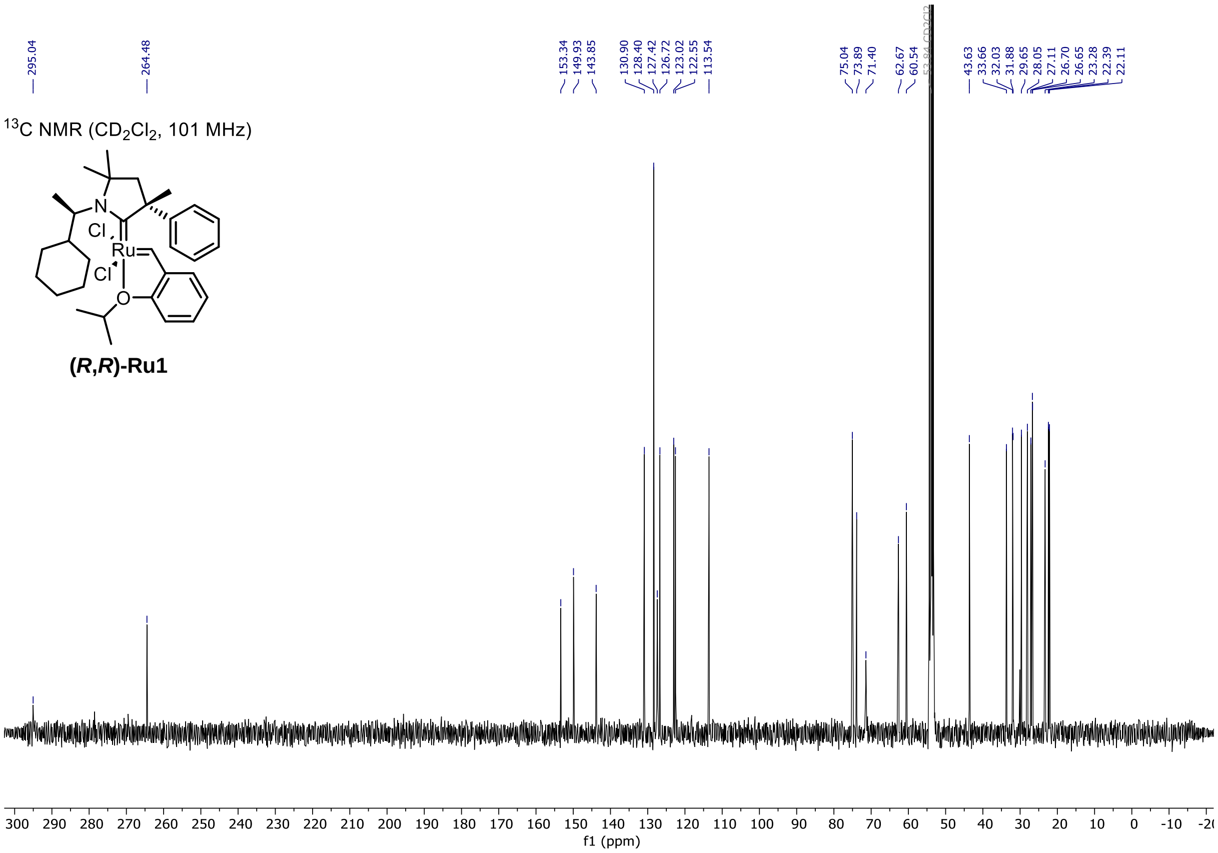
13C NMR (CD2Cl2, 101 MHz)
(R,R)-Ru1
f1 (ppm)

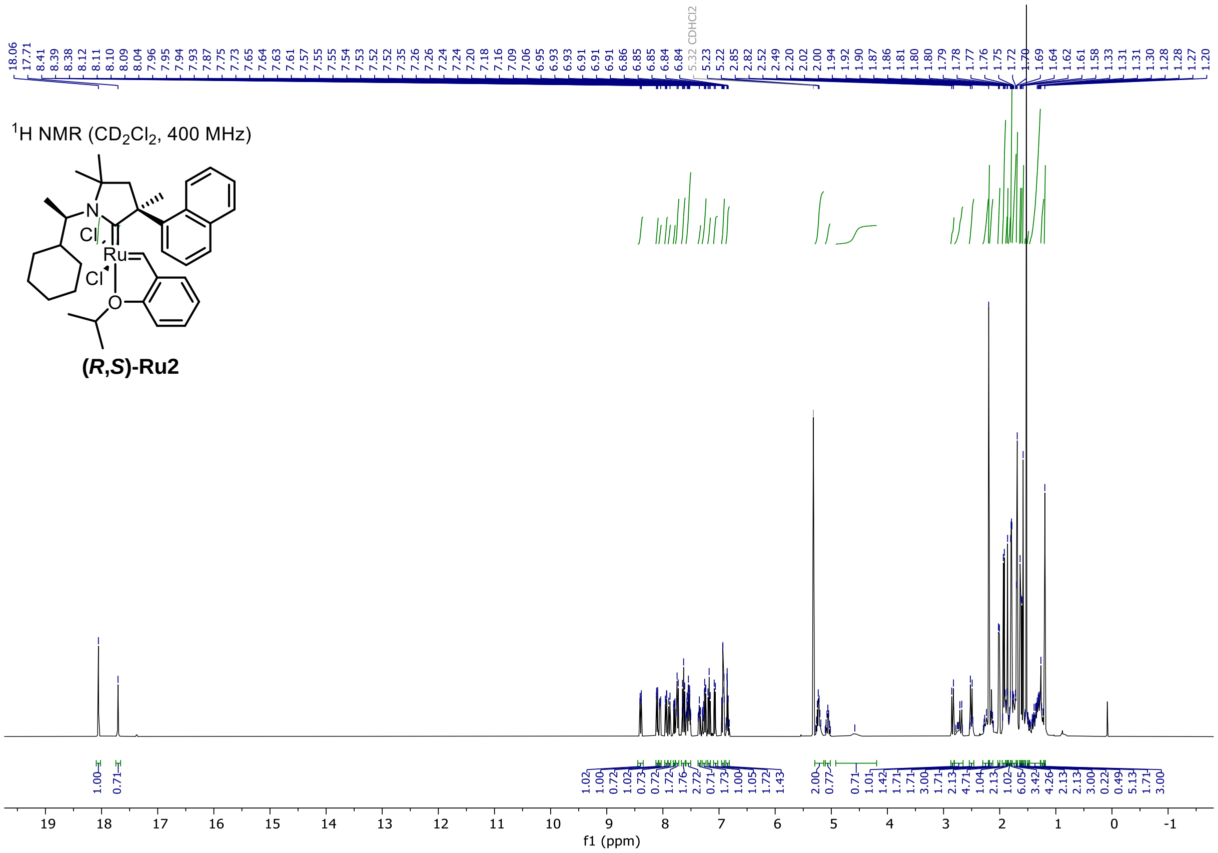

^1H NMR (CD_2Cl_2, 400 MHz)
(R,S)-Ru2
CDHCl2
f1 (ppm)

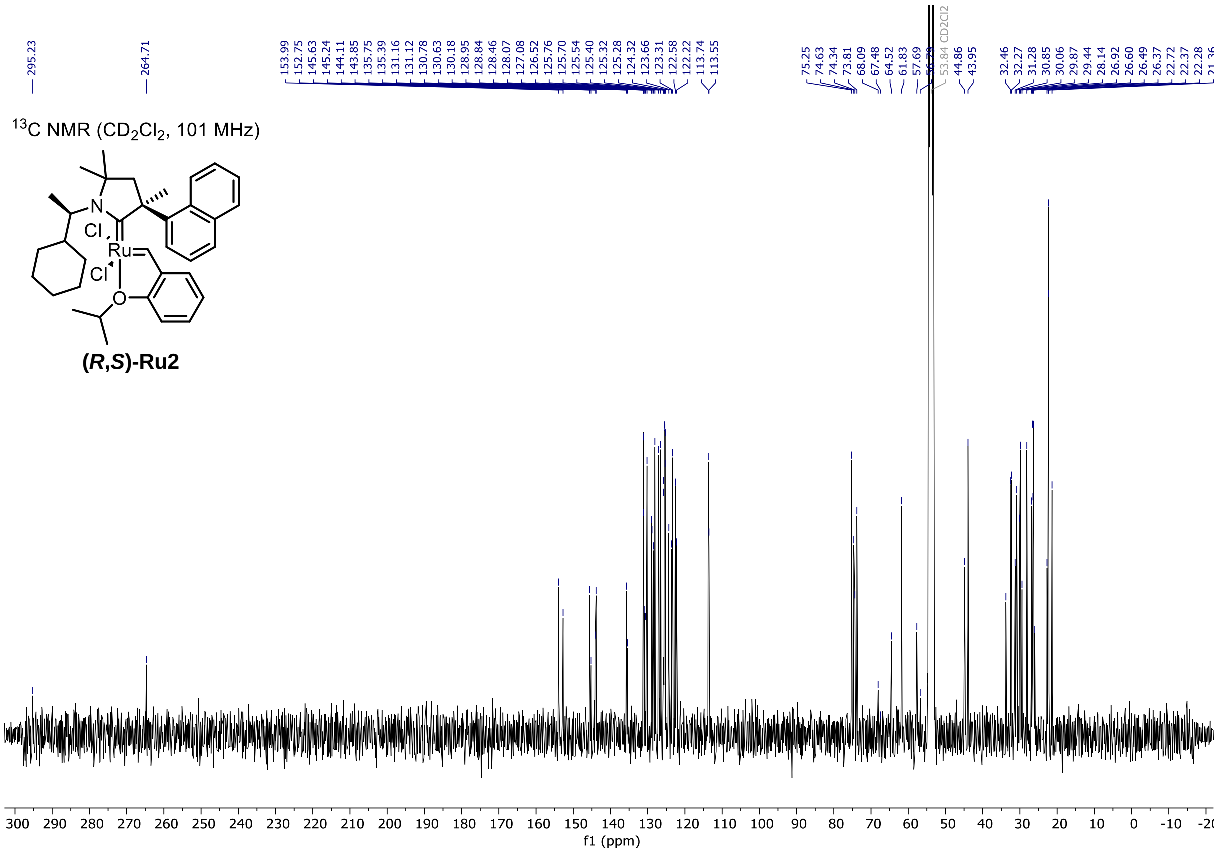

^{13}C NMR (CD_2Cl_2, 101 MHz)
(R,S)-Ru2
CD2Cl2
f1 (ppm)

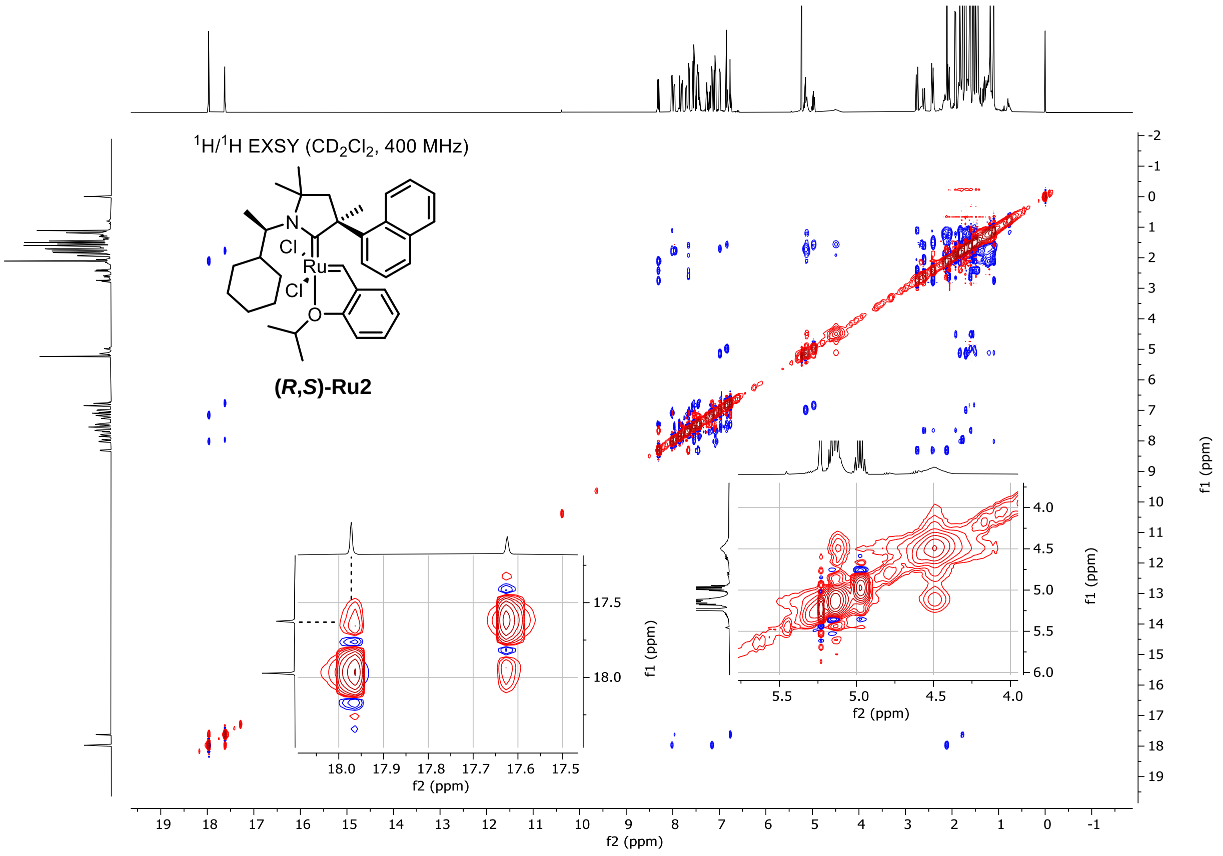

¹H/¹H EXSY (CD₂Cl₂, 400 MHz)
(R,S)-Ru2
f2 (ppm)
f1 (ppm)

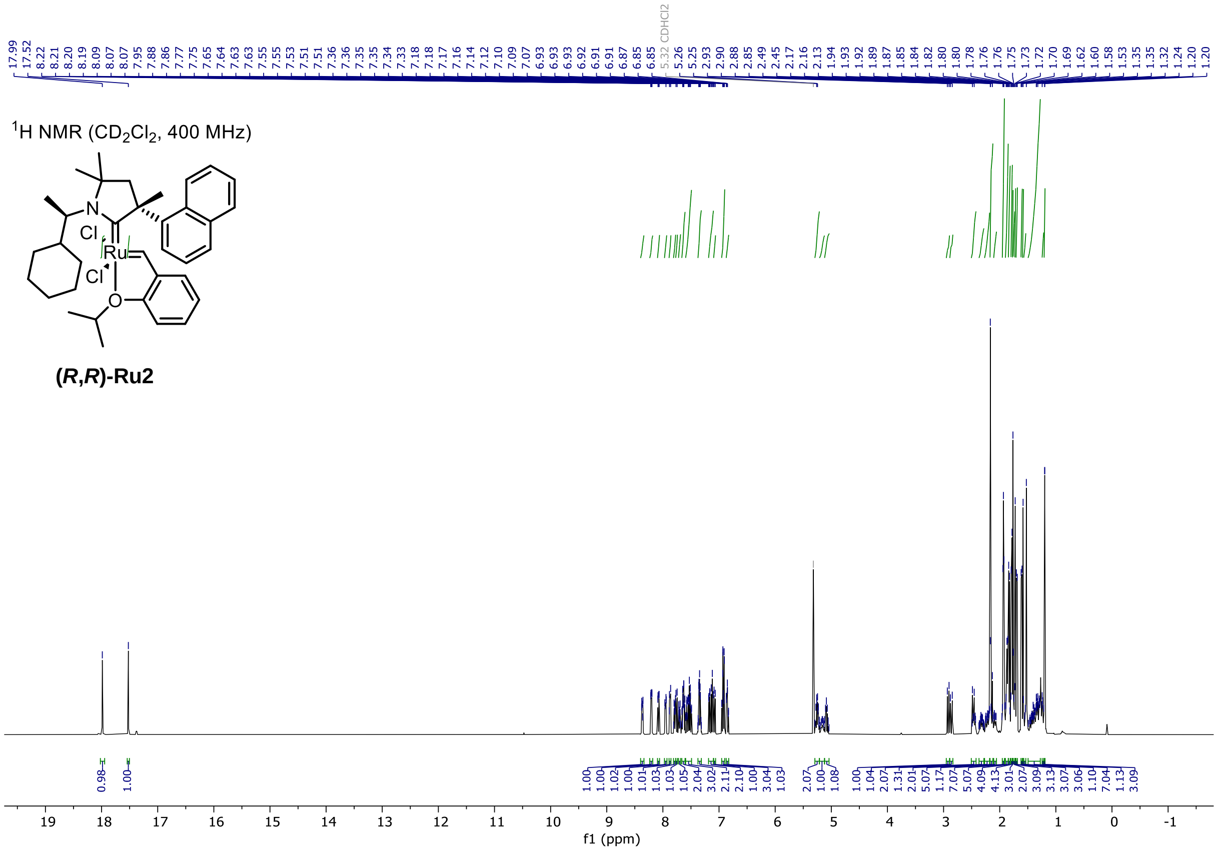
17.99 17.52 8.22 8.21 8.20 8.19 8.09 8.07 8.07 7.95 7.88 7.86 7.77 7.75 7.65 7.64 7.63 7.63 7.55 7.55 7.53 7.51 7.51 7.36 7.36 7.35 7.35 7.34 7.33 7.18 7.18 7.17 7.16 7.14 7.12 7.10 7.09 7.07 6.93 6.93 6.93 6.92 6.91 6.91 6.87 6.85 6.85 5.32 CDHCl2 5.26 5.25 2.93 2.90 2.88 2.85 2.49 2.45 2.17 2.16 2.13 1.94 1.93 1.92 1.89 1.87 1.85 1.84 1.82 1.80 1.80 1.78 1.76 1.76 1.75 1.73 1.72 1.70 1.69 1.62 1.60 1.58 1.53 1.35 1.35 1.32 1.24 1.20 1.20
^{1}H NMR (CD_2Cl_2, 400 MHz)
N
Cl
Ru
Cl
O
(*R*,*R*)-Ru2
0.98 1.00
1.00 1.00 1.02 1.00 1.01 1.03 1.03 1.05 2.04 3.02 2.11 2.10 1.00 3.04 1.03
2.07 1.00 1.08
1.00 1.04 2.07 1.31 2.01 5.07 1.17 7.07 5.07 4.09 4.13 3.01 2.07 3.09 3.13 3.07 3.06 1.10 7.04 1.13 3.09
19 18 17 16 15 14 13 12 11 10 9 8 7 6 5 4 3 2 1 0 -1
f1 (ppm)

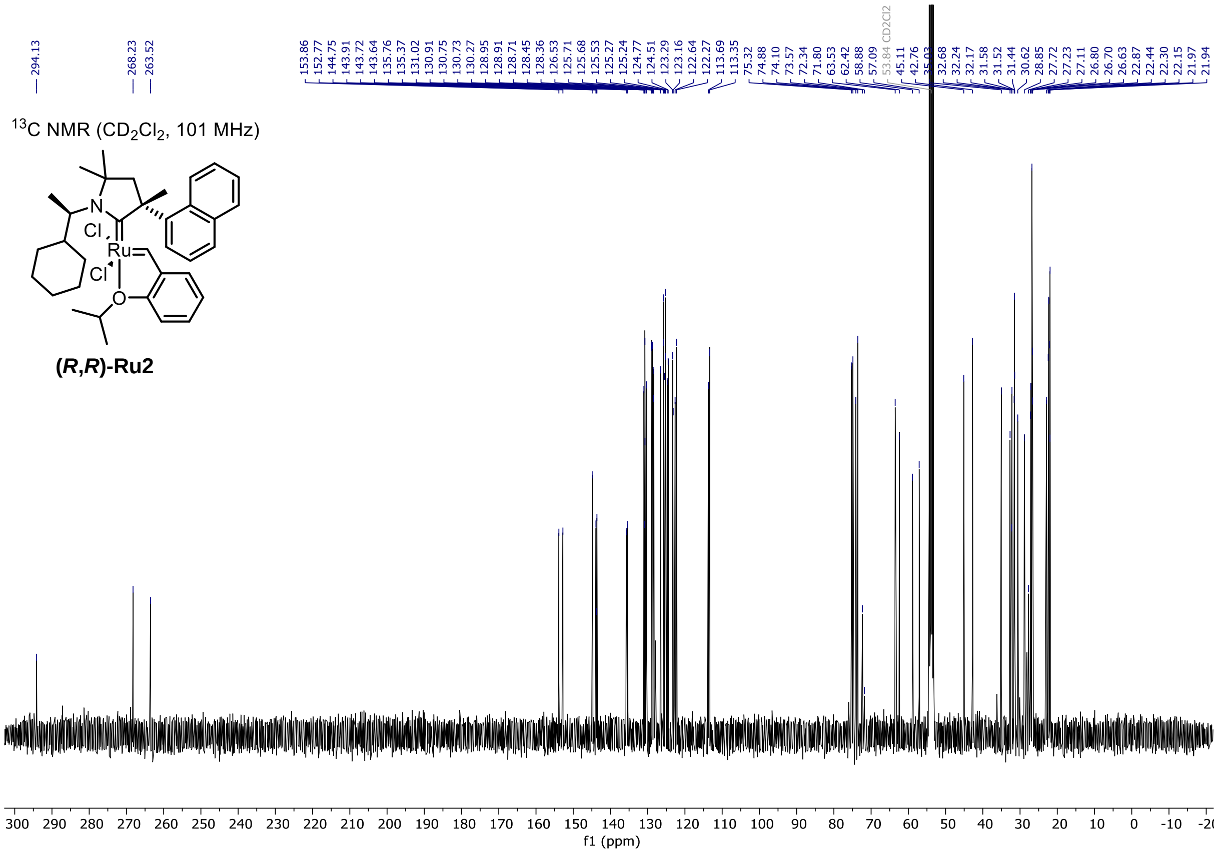
294.13
268.23 263.52
153.86 152.77 144.75 143.91 143.72 143.64 135.76 135.37 131.02 130.91 130.75 130.73 130.27 128.95 128.91 128.71 128.45 128.36 126.53 125.71 125.68 125.53 125.27 125.24 124.77 124.51 123.29 123.16 122.64 122.27 113.69 113.35
75.32 74.88 74.10 73.57 72.34 71.80 63.53 62.42 58.88 57.09 53.84 CD2Cl2 45.11 42.76 32.68 32.24 32.17 31.58 31.52 31.44 30.62 28.85 27.72 27.23 27.11 26.80 26.70 26.63 22.87 22.44 22.30 22.15 21.97 21.94
^{13}C NMR (CD_2Cl_2, 101 MHz)
N
Cl
Ru
Cl
O
(*R*,*R*)-Ru2
300 290 280 270 260 250 240 230 220 210 200 190 180 170 160 150 140 130 120 110 100 90 80 70 60 50 40 30 20 10 0 -10
f1 (ppm)

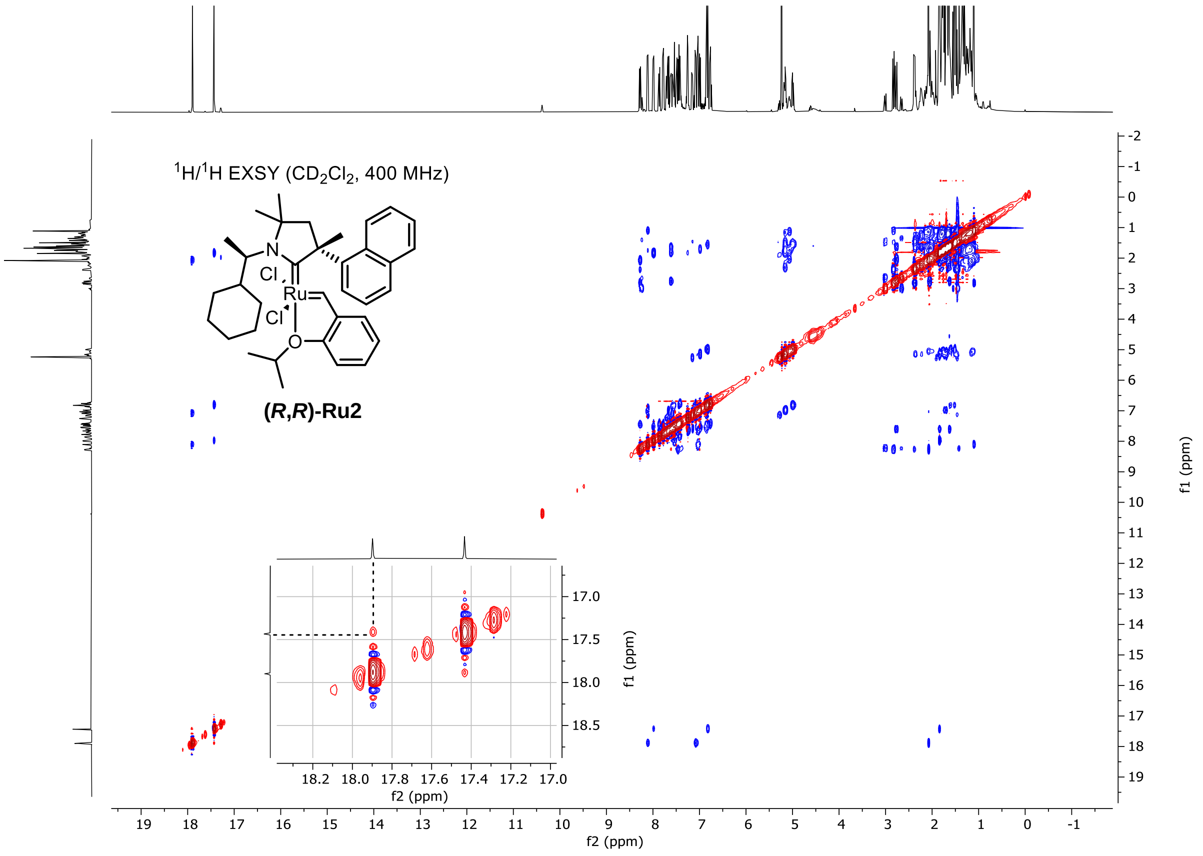

1H/1H EXSY (CD2Cl2, 400 MHz)
N
Cl
Ru
Cl
O
(R,R)-Ru2
f1 (ppm)
f2 (ppm)

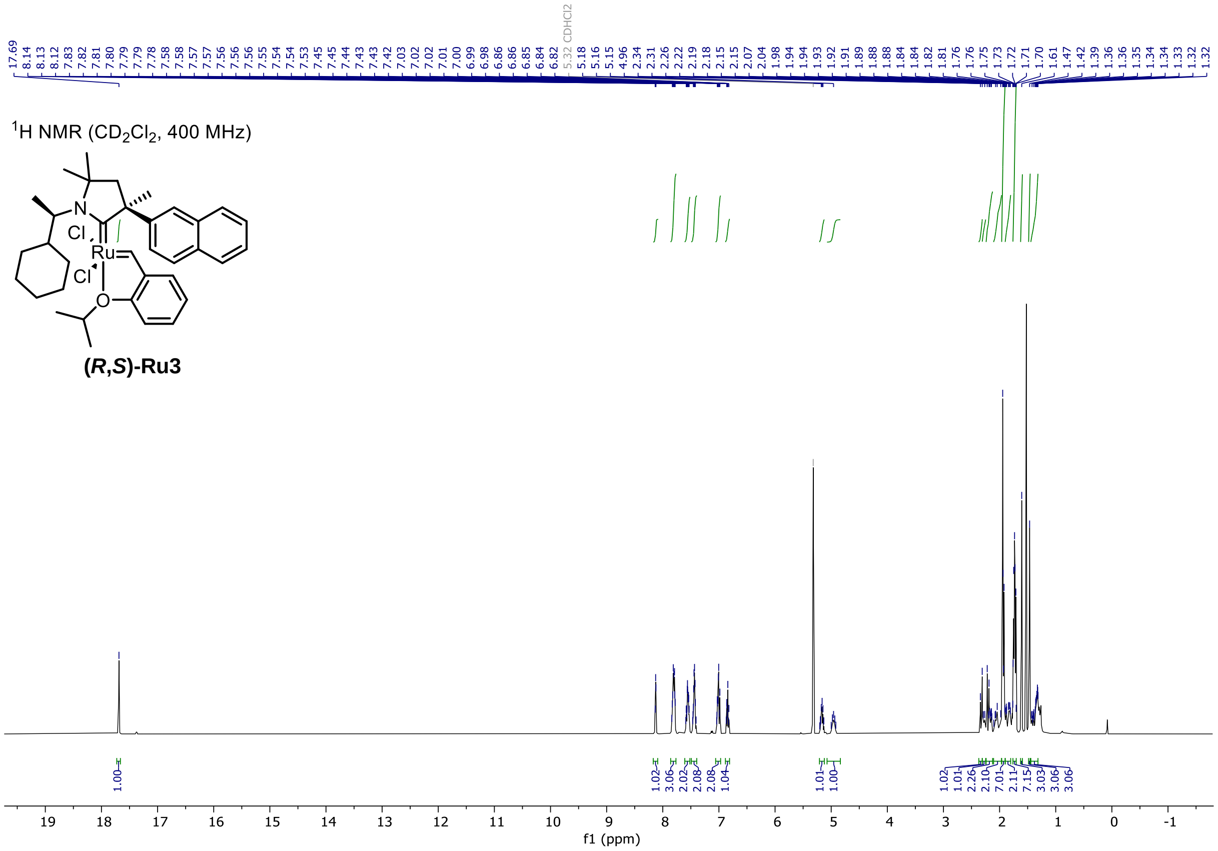
17.69 8.14 8.13 8.12 7.83 7.82 7.81 7.80 7.79 7.79 7.78 7.58 7.58 7.57 7.57 7.56 7.56 7.56 7.55 7.54 7.54 7.53 7.45 7.45 7.44 7.43 7.43 7.42 7.03 7.02 7.02 7.01 7.00 6.99 6.98 6.86 6.86 6.85 6.84 6.82 5.32 CDHCl2 5.18 5.16 5.15 4.96 2.34 2.31 2.26 2.22 2.19 2.18 2.15 2.15 2.07 2.04 1.98 1.94 1.94 1.93 1.92 1.91 1.89 1.88 1.88 1.84 1.84 1.82 1.81 1.76 1.75 1.73 1.72 1.71 1.70 1.61 1.47 1.42 1.39 1.36 1.35 1.34 1.34 1.33 1.32 1.32
^{1}H NMR (CD_2Cl_2, 400 MHz)
N
Cl
Ru
Cl
O
(*R*,*S*)-Ru3
1.00
1.02 3.06 2.02 2.08 2.08 1.04
1.01 1.00
1.02 1.01 2.26 2.10 7.01 2.11 7.15 3.03 3.06 3.06
19 18 17 16 15 14 13 12 11 10 9 8 7 6 5 4 3 2 1 0 -1
f1 (ppm)

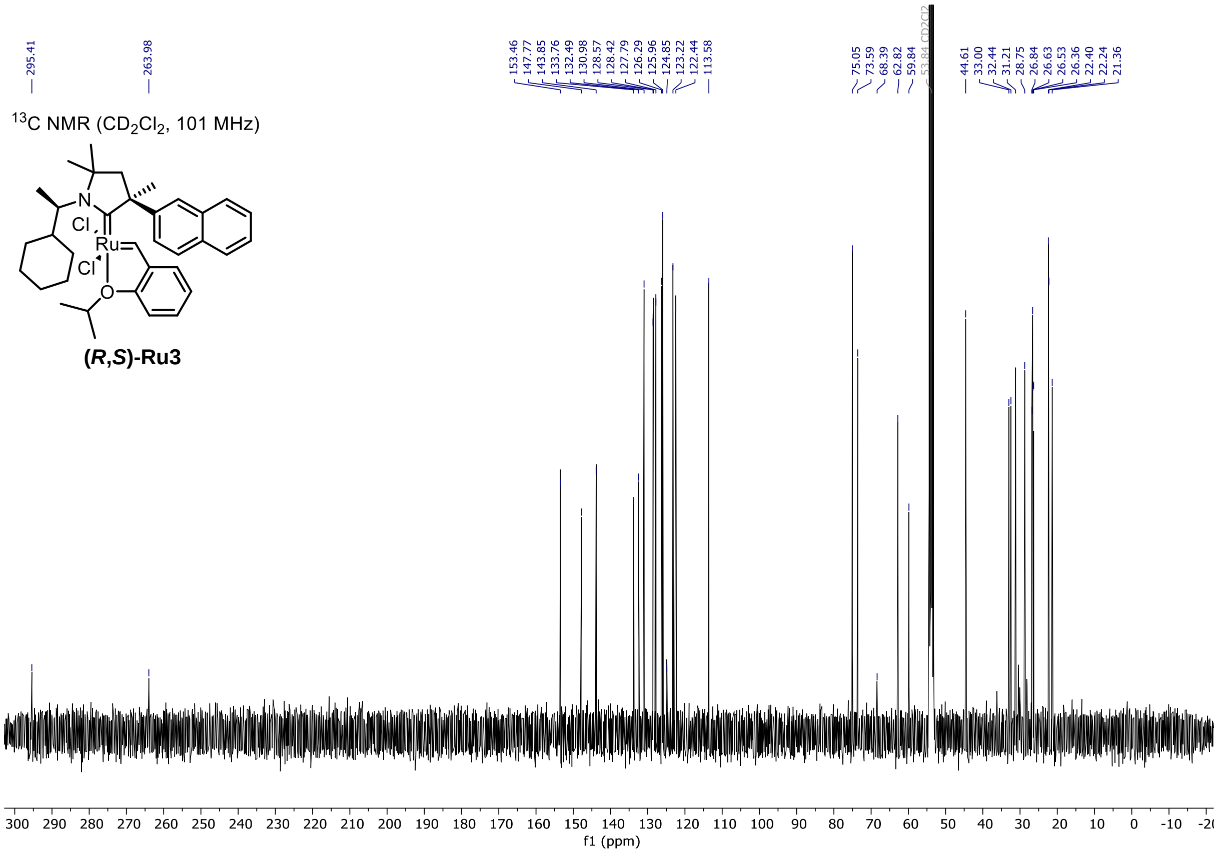
295.41
263.98
153.46 147.77 143.85 133.76 132.49 130.98 128.57 128.42 127.79 126.29 125.96 124.85 123.22 122.44 113.58
75.05 73.59 68.39 62.82 59.84 53.84 CD2Cl2
44.61 33.00 32.44 31.21 28.75 26.84 26.63 26.36 22.40 22.24 21.36
^{13}C NMR (CD_2Cl_2, 101 MHz)
N
Cl
Ru
Cl
O
(*R*,*S*)-Ru3
300 290 280 270 260 250 240 230 220 210 200 190 180 170 160 150 140 130 120 110 100 90 80 70 60 50 40 30 20 10 0 -10 -20
f1 (ppm)

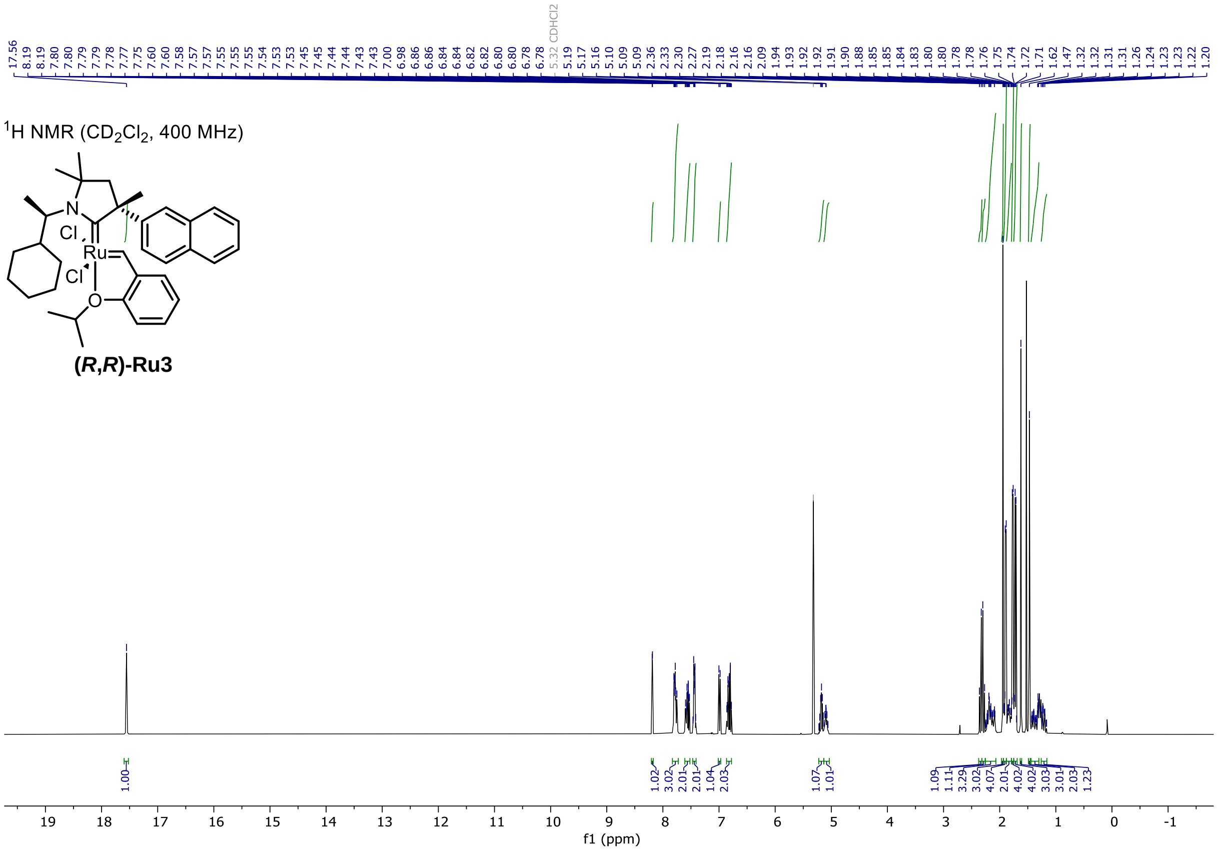
17.56 8.19 8.19 7.80 7.80 7.79 7.79 7.78 7.77 7.75 7.60 7.58 7.57 7.57 7.55 7.55 7.54 7.53 7.53 7.45 7.45 7.44 7.44 7.43 7.43 7.00 6.98 6.86 6.86 6.84 6.84 6.82 6.82 6.80 6.80 6.78 6.78 5.32 CDHCl2 5.19 5.17 5.16 5.10 5.09 5.09 2.36 2.33 2.30 2.27 2.19 2.18 2.16 2.16 2.09 1.94 1.93 1.93 1.92 1.92 1.91 1.90 1.88 1.85 1.85 1.84 1.83 1.80 1.80 1.78 1.76 1.75 1.74 1.72 1.71 1.62 1.47 1.32 1.32 1.31 1.26 1.24 1.23 1.23 1.22 1.20
1H NMR (CD2Cl2, 400 MHz)
Cl
Ru
Cl
N
O
(R,R)-Ru3
1.00 1.02 3.02 2.01 2.01 1.04 2.03 1.07 1.01 1.09 1.11 3.29 3.02 4.07 2.01 4.02 4.02 3.03 3.01 2.03 1.23
19 18 17 16 15 14 13 12 11 10 9 8 7 6 5 4 3 2 1 0 -1
f1 (ppm)

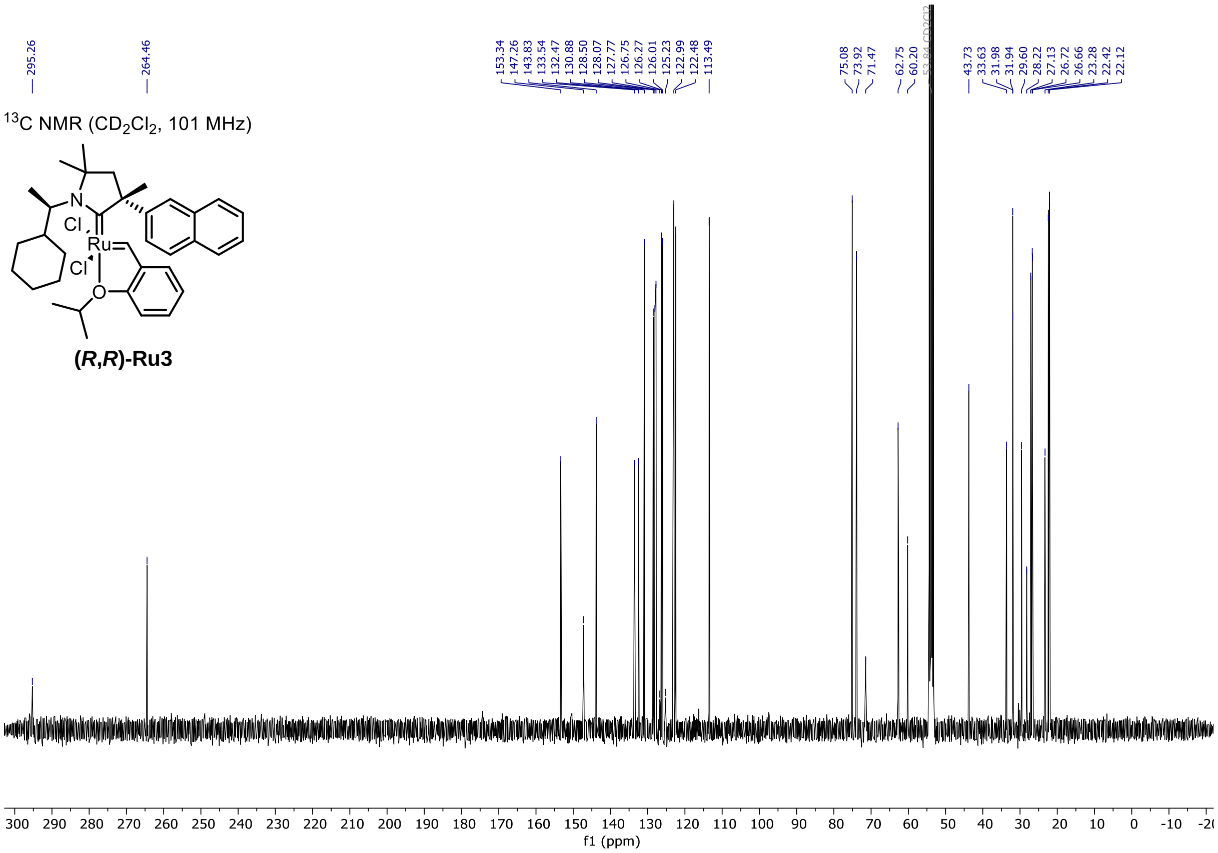
295.26
264.46
153.34 147.26 143.83 133.54 132.47 130.88 128.50 128.07 127.77 126.75 126.27 126.01 125.23 122.99 122.48 113.49
75.08 73.92 71.47 62.75 60.20 53.84 CD2Cl2
43.73 33.63 31.98 31.94 29.60 28.22 27.13 26.72 26.66 23.28 22.42 22.12
13C NMR (CD2Cl2, 101 MHz)
Cl
Ru
Cl
N
O
(R,R)-Ru3
300 290 280 270 260 250 240 230 220 210 200 190 180 170 160 150 140 130 120 110 100 90 80 70 60 50 40 30 20 10 0 -10 -20
f1 (ppm)

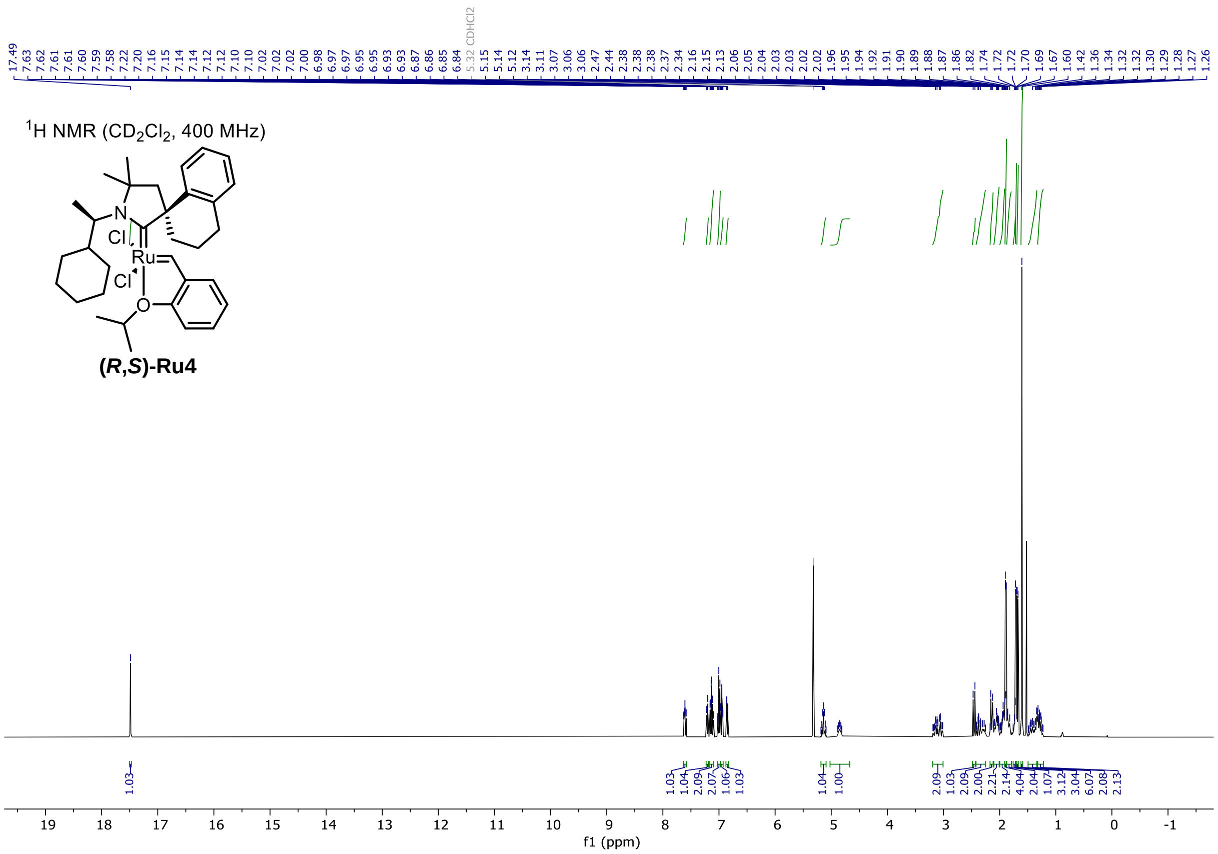

^{1}H NMR (CD_2Cl_2, 400 MHz)
(R,S)-Ru4
f1 (ppm)

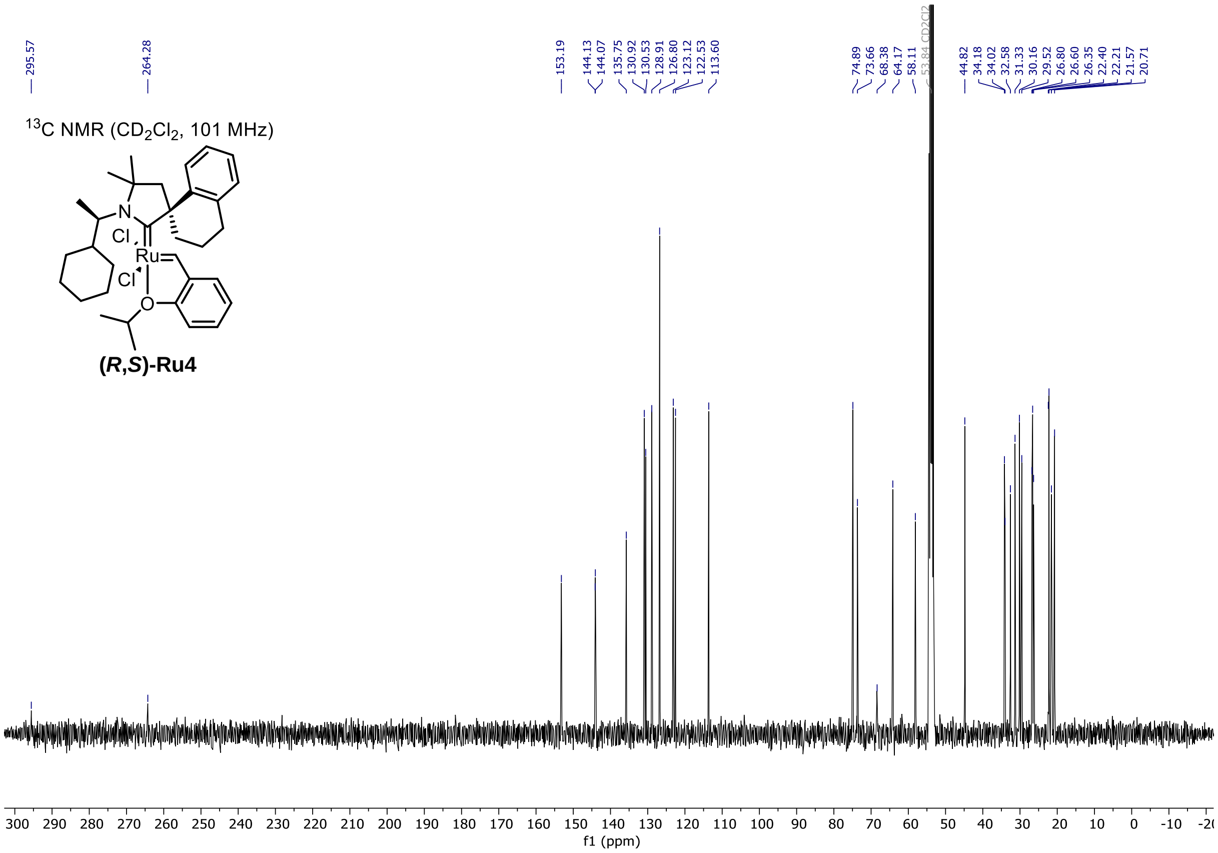

^{13}C NMR (CD_2Cl_2, 101 MHz)
(R,S)-Ru4
f1 (ppm)

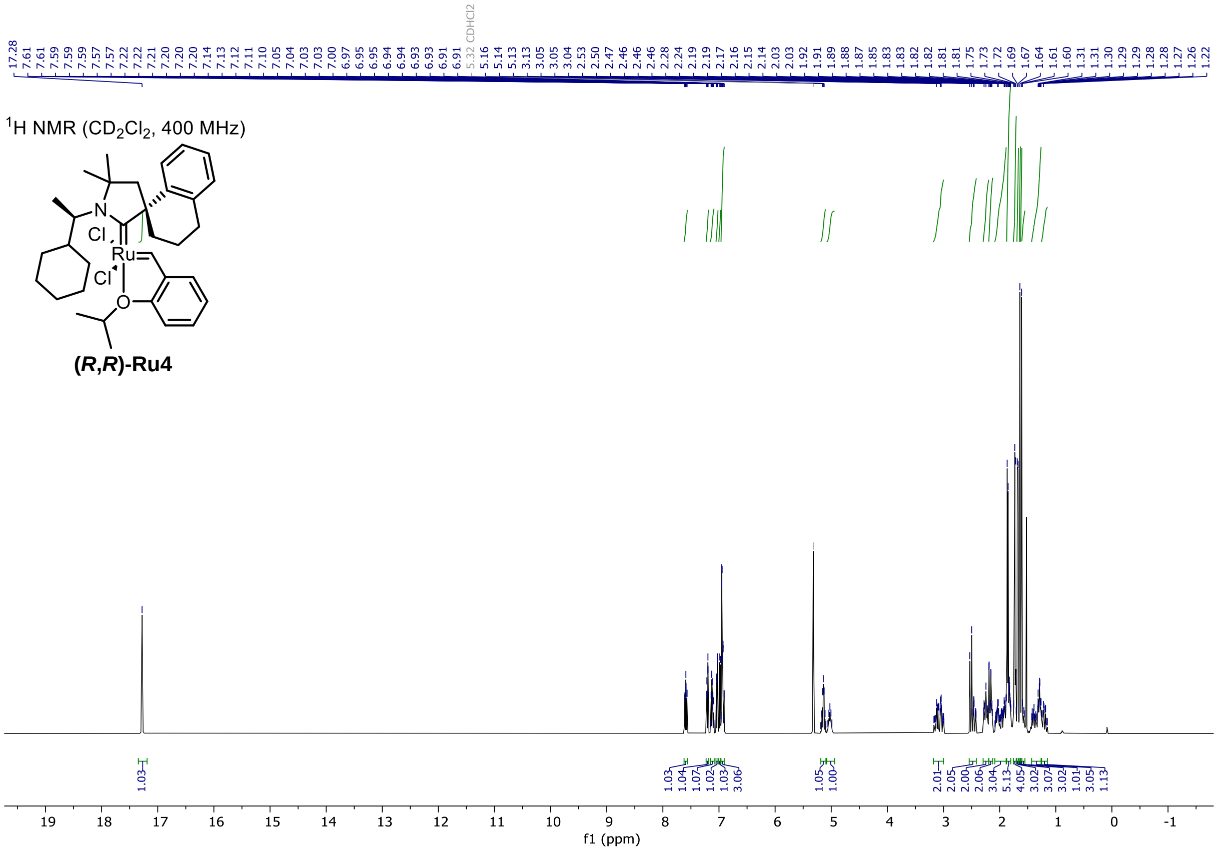
17.28 7.61 7.61 7.59 7.59 7.59 7.57 7.57 7.22 7.22 7.21 7.20 7.20 7.20 7.14 7.13 7.12 7.11 7.10 7.05 7.04 7.03 7.03 7.00 6.97 6.95 6.95 6.94 6.94 6.93 6.93 6.91 6.91 5.32 CDHCl2 5.16 5.14 5.13 3.13 3.05 3.04 2.53 2.50 2.47 2.46 2.46 2.46 2.28 2.24 2.19 2.19 2.17 2.16 2.15 2.14 2.03 2.03 1.92 1.91 1.89 1.88 1.87 1.85 1.83 1.83 1.82 1.82 1.81 1.81 1.75 1.73 1.72 1.69 1.67 1.64 1.61 1.60 1.31 1.31 1.30 1.29 1.29 1.28 1.27 1.26 1.22
1H NMR (CD2Cl2, 400 MHz)
Cl
Ru
Cl
N
O
(R,R)-Ru4
1.03 1.03 1.04 1.07 1.02 1.03 3.06 1.05 1.00 2.01 2.05 2.00 2.06 3.04 5.13 4.05 3.02 3.07 3.02 1.01 3.05 1.13
f1 (ppm)

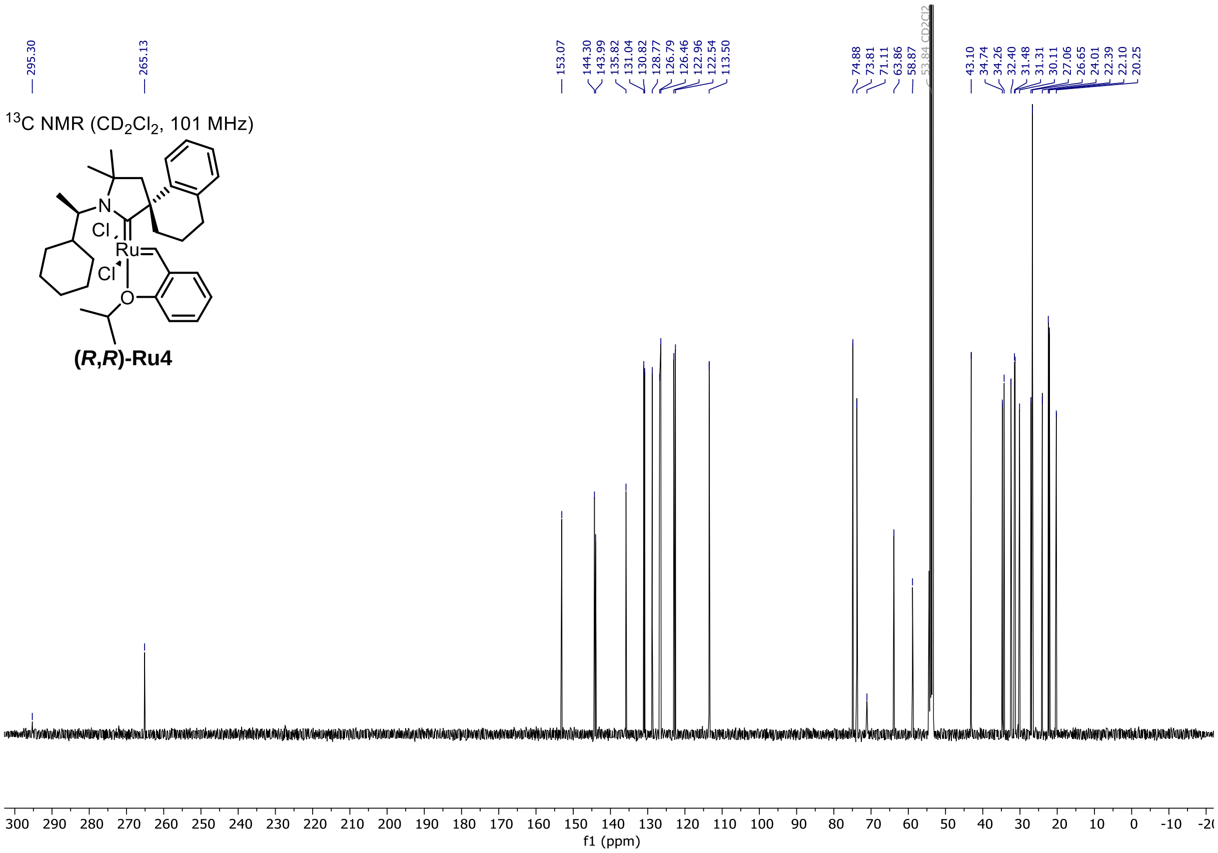
295.30 265.13 153.07 144.30 143.99 135.82 131.04 130.82 128.77 126.79 126.46 122.96 122.54 113.50 74.88 73.81 71.11 63.86 58.87 53.84 CD2Cl2 43.10 34.74 34.26 32.40 31.48 31.31 30.11 27.06 26.65 24.01 22.39 22.10 20.25
13C NMR (CD2Cl2, 101 MHz)
Cl
Ru
Cl
N
O
(R,R)-Ru4
f1 (ppm)

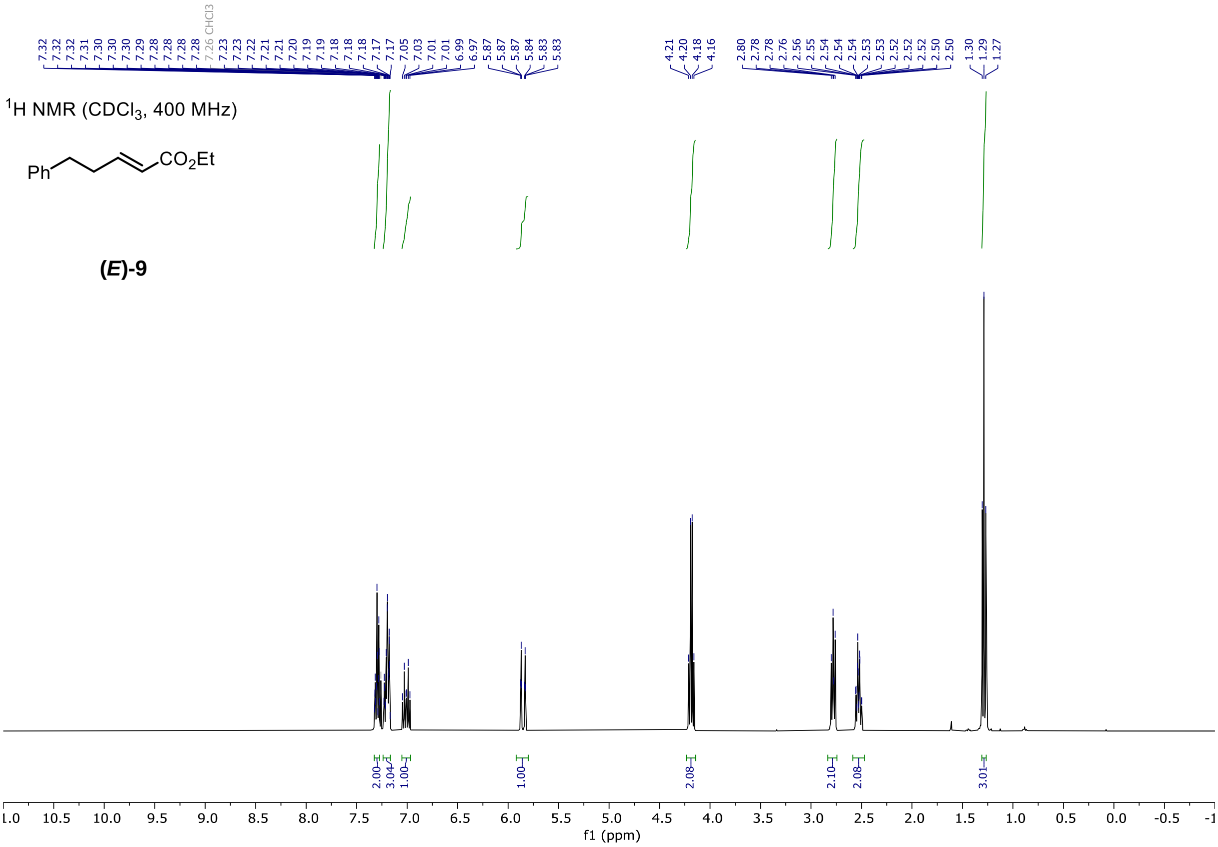

^{1}H NMR ($CDCl_3$, 400 MHz)
Ph
CO_2Et
(*E*)-9
f1 (ppm)

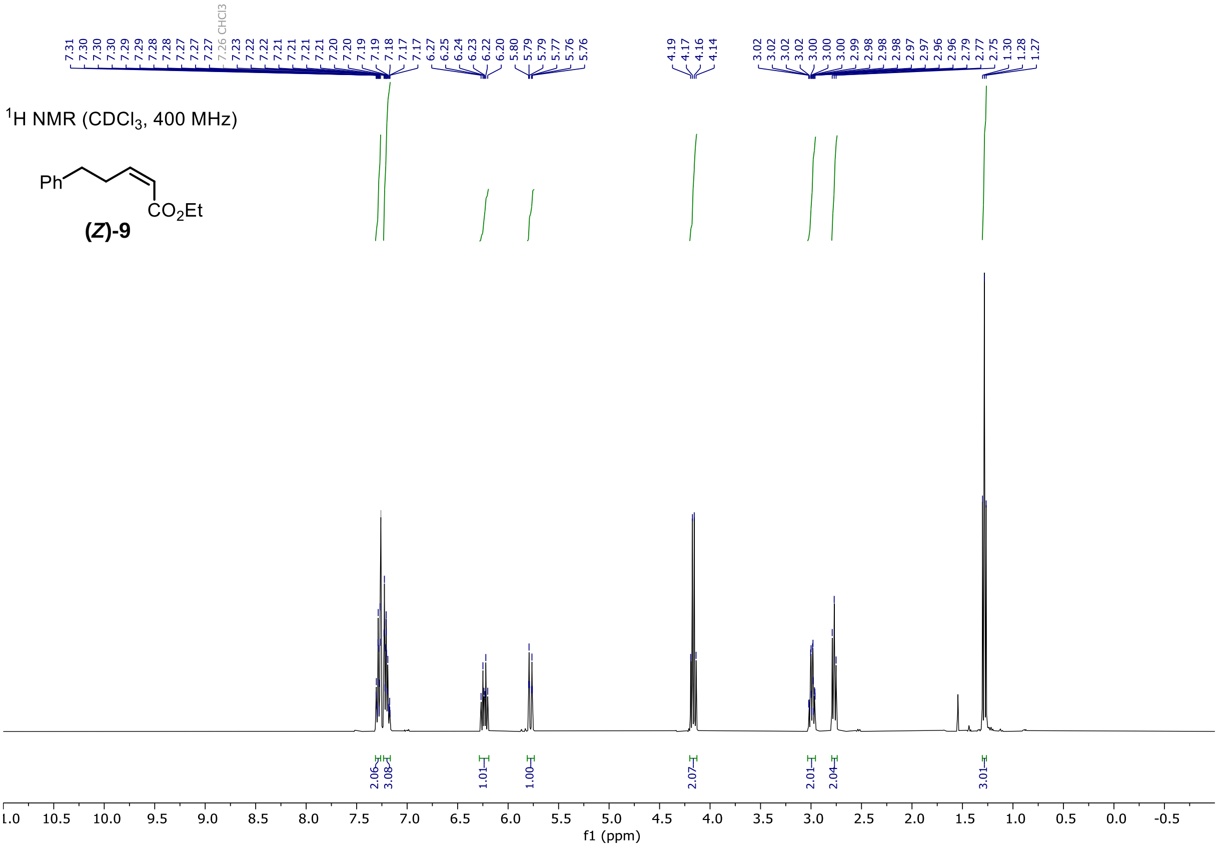

^{1}H NMR ($CDCl_3$, 400 MHz)
Ph
CO_2Et
(*Z*)-9
f1 (ppm)

^{1}H NMR ($CDCl_3$, 400 MHz)

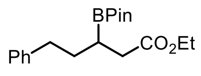

10

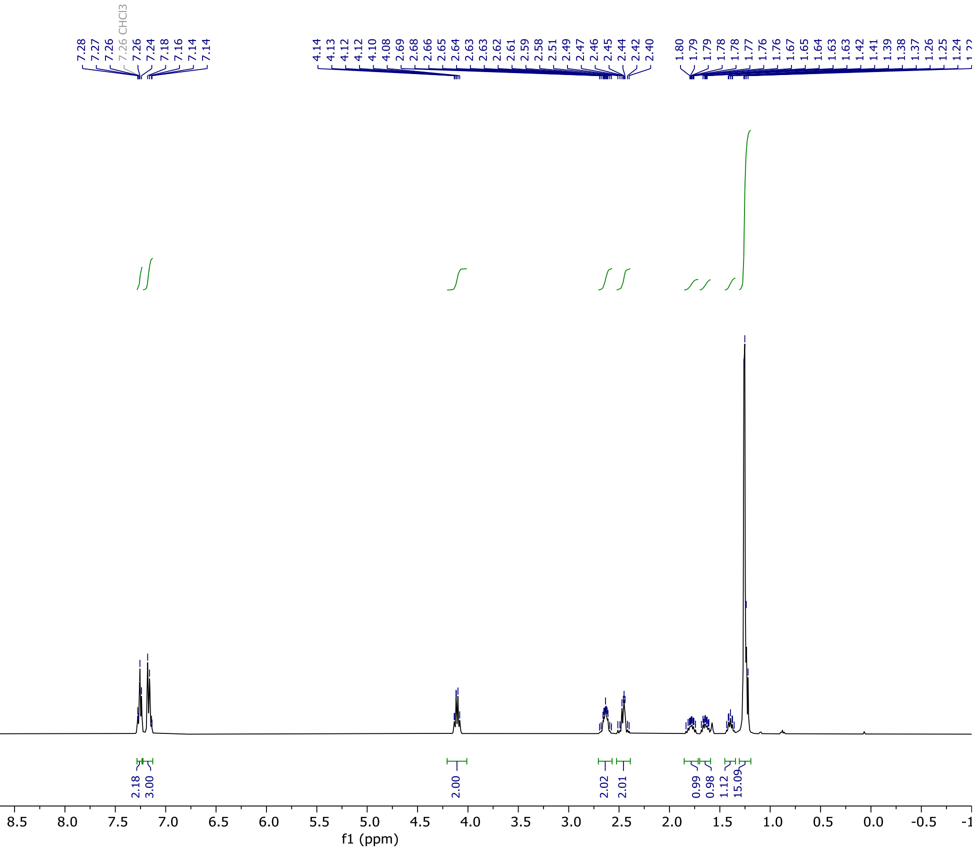

^{1}H NMR ($CDCl_3$, 400 MHz)

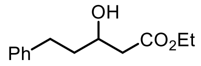

S-V

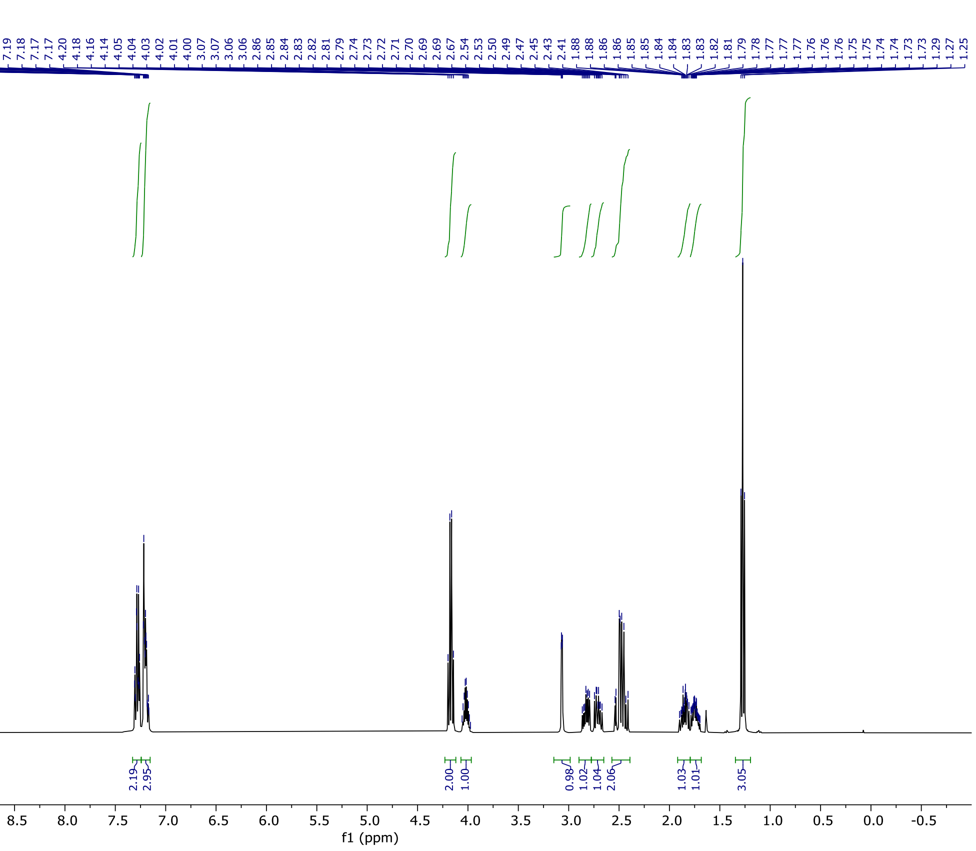

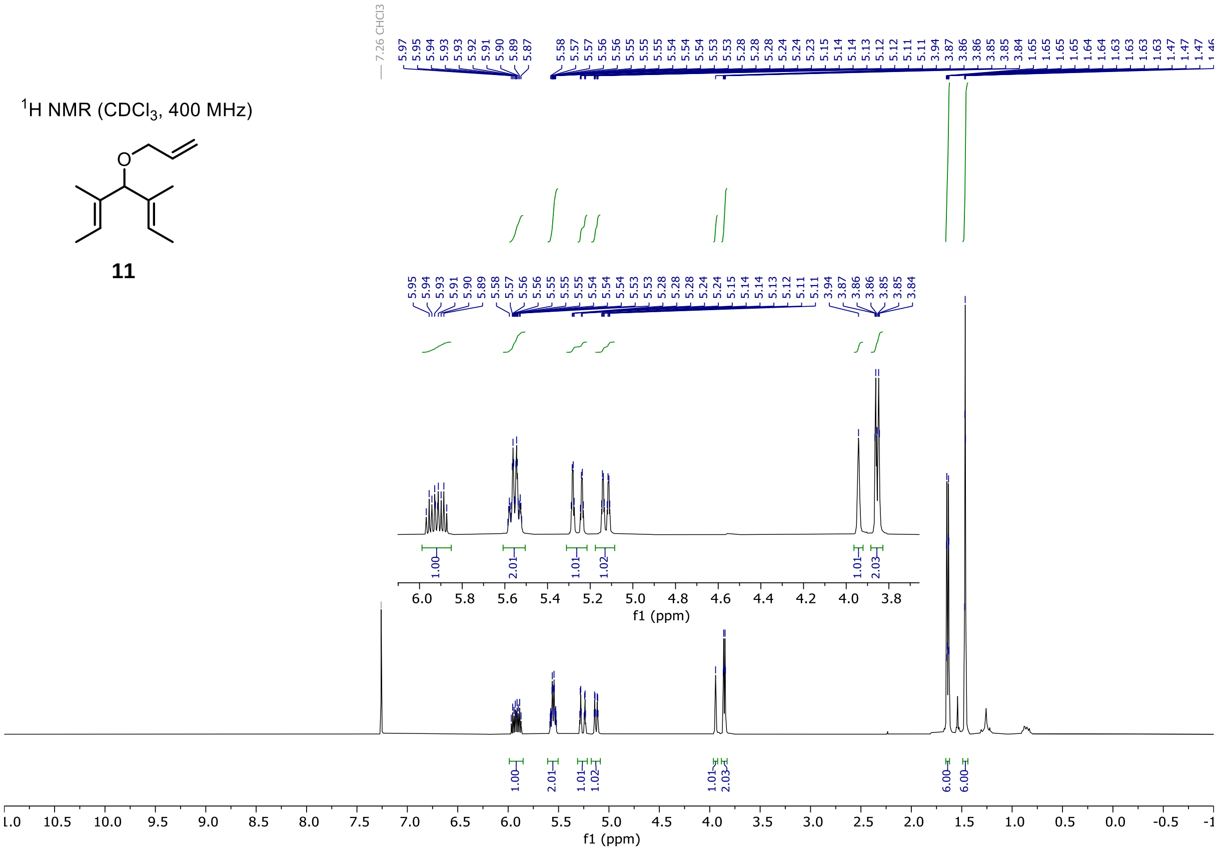

¹H NMR (CDCl3, 400 MHz)
11
7.26 CHCl3
f1 (ppm)

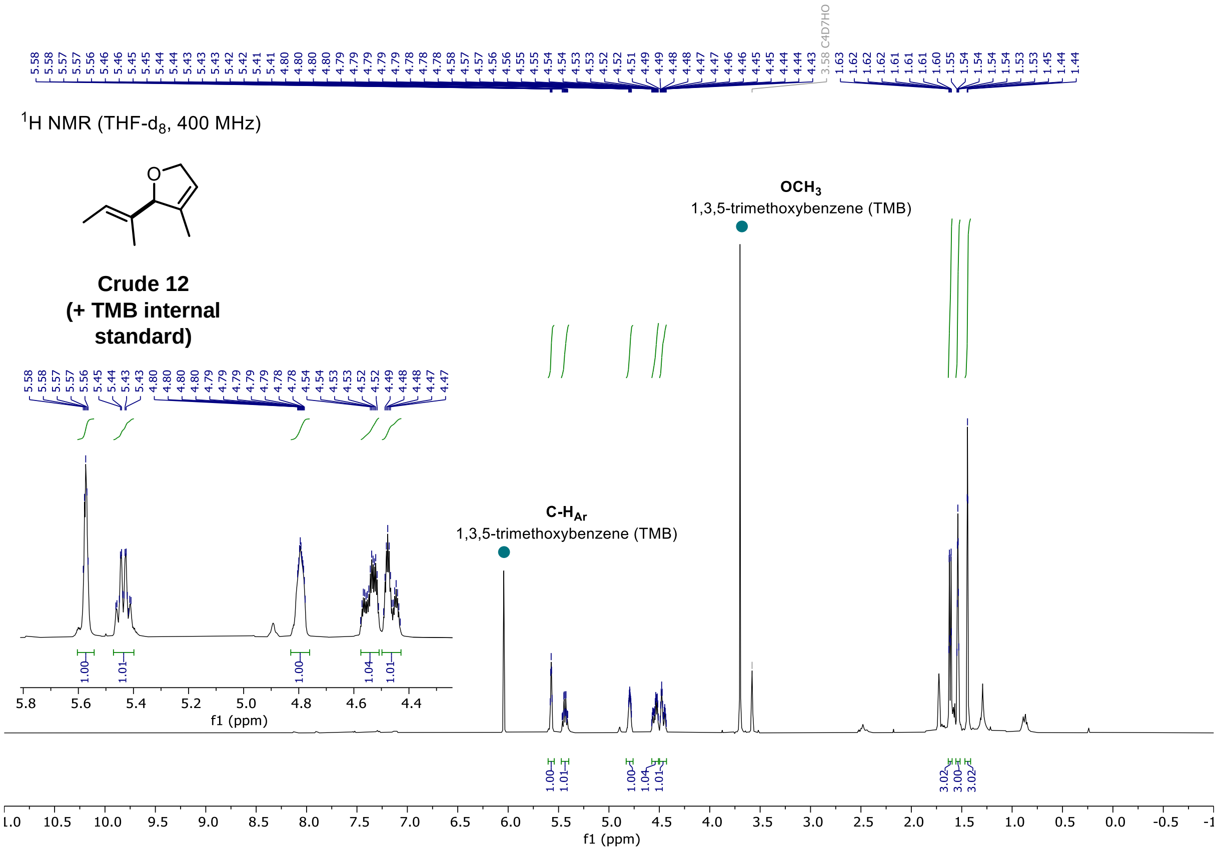

¹H NMR (THF-d8, 400 MHz)
Crude 12
(+ TMB internal standard)
OCH3
1,3,5-trimethoxybenzene (TMB)
C-HAr
1,3,5-trimethoxybenzene (TMB)
3.58 C4D7HO
f1 (ppm)
